# Cemiplimab plus chemotherapy versus chemotherapy alone in non-small cell lung cancer: a randomized, controlled, double-blind phase 3 trial

**DOI:** 10.1038/s41591-022-01977-y

**Published:** 2022-08-25

**Authors:** Miranda Gogishvili, Tamar Melkadze, Tamta Makharadze, Davit Giorgadze, Mikhail Dvorkin, Konstantin Penkov, Konstantin Laktionov, Gia Nemsadze, Marina Nechaeva, Irina Rozhkova, Ewa Kalinka, Christian Gessner, Brizio Moreno-Jaime, Rodolfo Passalacqua, Siyu Li, Kristina McGuire, Manika Kaul, Anne Paccaly, Ruben G. W. Quek, Bo Gao, Frank Seebach, David M. Weinreich, George D. Yancopoulos, Israel Lowy, Giuseppe Gullo, Petra Rietschel

**Affiliations:** 1 High Technology Medical Centre, University Clinic Ltd., Tbilisi, Georgia; 2 Acad. F. Todua Medical Center, Tbilisi, Georgia; 3 LTD High Technology Hospital Med Center, Batumi, Georgia; 4 David Tvildiani Medical University, Tbilisi, Georgia; 5 State Budgetary Healthcare Institution of Omsk Region, Omsk, Russia; 6 Private Medical Institution Euromedservice, Saint Petersburg, Russia; 7 Federal State Budgetary Institution ‘N.N. Blokhin National Medical Research Center of Oncology’ of the Ministry of Health of the Russian Federation, Moscow, Russia; 8 The Institute of Clinical Oncology, Tbilisi, Georgia; 9 Chelyabinsk Regional Clinical Oncology Center, Chelyabinsk, Chelyabinsk, Russia; 10 Kaluga Regional Clinical Oncology Dispensary, Kaluga, Russia; 11 Polish Mother’s Memorial Hospital Research Institute, Łódź, Poland; 12 POIS Leipzig GbR, Leipzig, Germany; 13 Institute of Clinical Immunology, University of Leipzig, Leipzig, Germany; 14 Hospital Regional ISSSTE, León, Mexico; 15 Istituti Ospitalieri Di Cremona, Cremona, Italy; 16 Regeneron Pharmaceuticals, Inc., Tarrytown, NY USA

**Keywords:** Non-small-cell lung cancer, Cancer immunotherapy

## Abstract

First-line cemiplimab (anti-programmed cell death-1 (PD-1)) monotherapy has previously shown significant improvement in overall survival (OS) and progression-free survival (PFS) versus chemotherapy in patients with advanced non-small cell lung cancer (aNSCLC) and PD-ligand 1 (PD-L1) expression ≥50%. EMPOWER-Lung 3 (NCT03409614), a double-blind, placebo-controlled, phase 3 study, examined cemiplimab plus platinum-doublet chemotherapy as first-line treatment for aNSCLC, irrespective of PD-L1 expression or histology. In this study, 466 patients with stage III/IV aNSCLC without *EGFR*, *ALK* or *ROS1* genomic tumor aberrations were randomized (2:1) to receive cemiplimab 350 mg (*n* = 312) or placebo (*n* = 154) every 3 weeks for up to 108 weeks in combination with four cycles of platinum-doublet chemotherapy (followed by pemetrexed maintenance as indicated). In total, 57.1% (266/466 patients) had non-squamous NSCLC, and 85.2% (397/466 patients) had stage IV disease. The primary endpoint was OS. The trial was stopped early per recommendation of the independent data monitoring committee, based on meeting preset OS efficacy criteria: median OS was 21.9 months (95% confidence interval (CI), 15.5–not evaluable) with cemiplimab plus chemotherapy versus 13.0 months (95% CI, 11.9–16.1) with placebo plus chemotherapy (hazard ratio (HR) = 0.71; 95% CI, 0.53–0.93; *P* = 0.014). Grade ≥3 adverse events occurred with cemiplimab plus chemotherapy (43.6%, 136/312 patients) and placebo plus chemotherapy (31.4%, 48/153 patients). Cemiplimab is only the second anti-PD-1/PD-L1 agent to show efficacy in aNSCLC as both monotherapy and in combination with chemotherapy for both squamous and non-squamous histologies.

## Abstract (web summary)

Results from EMPOWER-Lung 3 demonstrate increased overall survival with cemiplimab plus platinum-doublet chemotherapy compared to cemiplimab as first-line treatment in patients with advanced non-small cell lung cancer, irrespective of PD-L1 expression.

## Main

PD-1 and PD-L1 inhibitors are the mainstay of systemic treatment for aNSCLC in patients without therapeutically actionable tumor genomic aberrations, such as epidermal growth factor receptor (*EGFR)* mutations, anaplastic lymphoma kinase (*ALK)* translocations or ROS proto-oncogene 1 (*ROS1)* fusions^1–3^. Pembrolizumab and atezolizumab (anti–PD-L1) are both approved as first-line therapies in combination with platinum-based chemotherapy and other therapies for certain patient populations with metastatic NSCLC, although atezolizumab approval with platinum-doublet chemotherapy is limited to non-squamous histology^2,4,5^. Combinations of checkpoint inhibitors—ipilimumab (anti–cytotoxic T-lymphocyte-associated protein 4) plus nivolumab (anti–PD-1) with or without chemotherapy—are also approved for patients with aNSCLC irrespective of histology, but the clinical utility of these agents compared to single-agent checkpoint treatments alone or in combination with chemotherapy is unclear^6,7^.

After demonstration of superior survival benefit for cemiplimab as monotherapy versus chemotherapy in EMPOWER-Lung 1 (ref. ^8^), cemiplimab was approved in the United States and the European Union as first-line treatment for patients with aNSCLC and PD-L1 ≥ 50% and with no *EGFR*, anaplastic *ALK* or *ROS1* genomic aberrations, and is a preferred treatment for these patients by National Comprehensive Cancer Network (NCCN) guidelines^1,8–11^.

In EMPOWER-Lung 3, we examined first-line cemiplimab in combination with investigator’s choice of platinum-doublet chemotherapy in patients with aNSCLC (metastatic or unresectable locally advanced disease not suitable for definitive chemoradiation), with either squamous or non-squamous histology and any level of PD-L1 expression.

## Results

### Patient characteristics

**Fig. 1:**
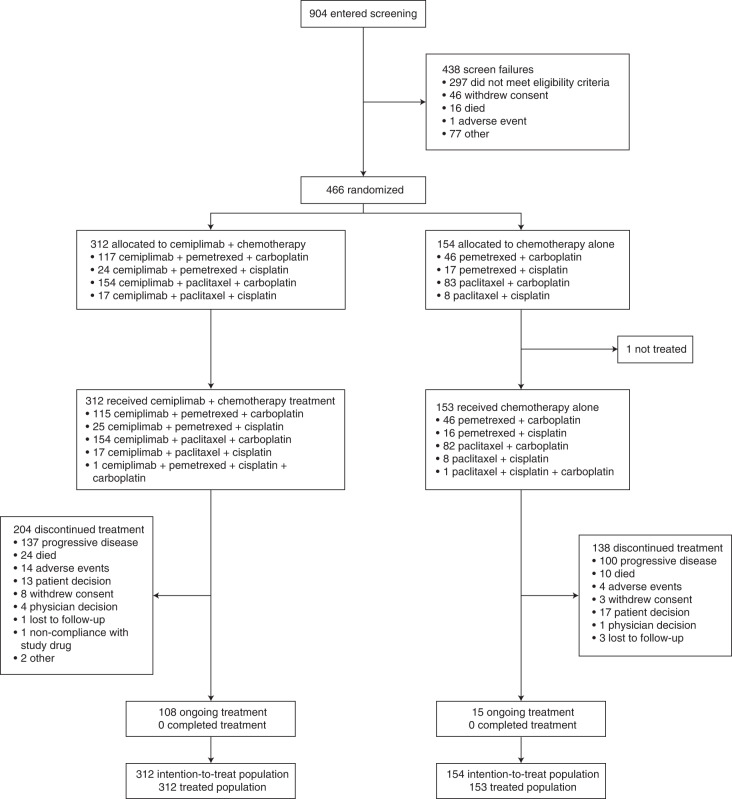
CONSORT diagram of EMPOWER-Lung 3 part two. All randomized patients were included in the efficacy analyses, and all patients who received treatment were included in the safety analyses.

In total, 904 patients were screened for enrollment at 74 sites in ten countries (Supplementary Table 1). Between 17 June 2019 and 30 September 2020, 466 patients were enrolled and randomly assigned 2:1 to cemiplimab plus chemotherapy (*n* = 312) and placebo plus chemotherapy (*n* = 154) treatment arms (Fig. 1). All patients who received cemiplimab plus chemotherapy and 153 patients (99.4%) in the placebo plus chemotherapy arm received at least one dose of study treatment. Baseline characteristics were generally well balanced between both arms; 42.9% (*n* = 200) of patients had squamous histology, 84.3% (*n* = 393) of patients had an Eastern Cooperative Oncology Group performance status (ECOG PS) of 1, and 14.8% (*n* = 69) of patients had locally advanced disease (Table 1).

**Table 1:** Baseline characteristics of the intention-to-treat patient population

Characteristic	Cemiplimab + chemotherapy (*n* = 312)	Placebo + chemotherapy (*n* = 154)	Total (*n* = 466)
Age, years			
Median (IQR)	63.0 (57–68)	63.0 (57–68)	63.0 (57–68)
≥65, *n* (%)	128 (41.0)	60 (39.0)	188 (40.3)
Sex, *n* (%)			
Women	44 (14.1)	31 (20.1)	75 (16.1)
Men	268 (85.9)	123 (79.9)	391 (83.9)
Geographic region, *n* (%)			
Europe	270 (86.5)	138 (89.6)	408 (87.6)
Asia	42 (13.5)	16 (10.4)	58 (12.4)
Histology, *n* (%)			
Non-squamous	179 (57.4)	87 (56.5)	266 (57.1)
Squamous	133 (42.6)	67 (43.5)	200 (42.9)
PD-L1 expression, *n* (%)			
<1%	95 (30.4)	44 (28.6)	139 (29.8)
1–49%	114 (36.5)	61 (39.6)	175 (37.6)
≥50%	103 (33.0)	49 (31.8)	152 (32.6)
ECOG PS, *n* (%)			
﻿ 0	51 (16.3)	18 (11.7)	69 (14.8)
﻿ 1	259 (83.0)	134 (87.0)	393 (84.3)
Brain metastasis, *n* (%)	24 (7.7)	7 (4.5)	31 (6.7)
Cancer stage at screening, *n* (%)			
Metastatic	267 (85.6)	130 (84.4)	397 (85.2)
Locally advanced	45 (14.4)	24 (15.6)	69 (14.8)
Smoking history, *n* (%)			
Current smoker	173 (55.4)	75 (48.7)	248 (53.2)
Past smoker	96 (30.8)	55 (35.7)	151 (32.4)
Never smoker	43 (13.8)	24 (15.6)	67 (14.4)
Previous cancer-related therapy, *n* (%)			
Systemic adjuvant therapy	5 (1.6)	1 (0.6)	6 (1.3)
Systemic other	1 (0.3)	0	1 (0.2)
Radiotherapy	40 (12.8)	11 (7.1)	51 (10.9)

The intention-to-treat population includes all randomized patients.

The trial was stopped early per recommendation of the independent data monitoring committee (IDMC), based on meeting preset criteria for OS efficacy (Methods). At the time of data cutoff (14 June 2021), 108 patients in the cemiplimab plus chemotherapy arm and 15 patients in the placebo plus chemotherapy arm remained on treatment. In the cemiplimab plus chemotherapy arm, 204 (65.4%) patients discontinued treatment, primarily due to progressive disease. In the placebo plus chemotherapy arm, 138 (89.6%) patients discontinued treatment, primarily due to progressive disease. The median duration of follow-up was 16.3 months (interquartile range (IQR), 13.9–19.1) in the cemiplimab plus chemotherapy arm and 16.7 months (IQR, 14.2–19.0) in the placebo plus chemotherapy arm. Treatment exposures are summarized in Supplementary Table 2.

### OS

**Fig. 2:**
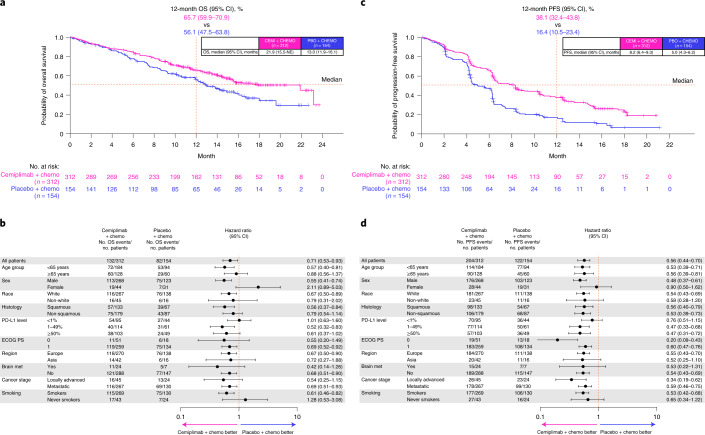
Survival data for the cemiplimab plus chemotherapy and placebo plus chemotherapy arms. **a**, Kaplan–Meier OS curves of all patients. **b**, Forest plots of OS by subgroups. **c**, Kaplan–Meier PFS curves of all patients. **d**, Forest plots of PFS by subgroups. Median OS and PFS and corresponding two-sided 95% CIs were estimated by the Kaplan–Meier method. HRs and corresponding two-sided 95% CIs for OS and PFS were calculated using a stratified Cox proportional hazard model with Efron’s method of tie handling. Cemi, cemiplimab; chemo, chemotherapy; met, metastasis; PBO, placebo.

With 214 deaths, the primary endpoint, median OS with cemiplimab plus chemotherapy, was 21.9 months (95% CI, 15.5–not evaluable (NE)) versus 13.0 months (95% CI, 11.9–16.1) with placebo plus chemotherapy (HR = 0.71; 95% CI, 0.53–0.93; *P* = 0.014; Fig. 2a). In the cemiplimab plus chemotherapy arm, the secondary endpoint of estimated proportion of patients who were alive at 12 months was 65.7% (95% CI, 59.9–70.9) versus 56.1% (95% CI, 47.5–63.8) in the placebo plus chemotherapy arm.

Although the study was not powered to examine efficacy within predefined subgroups, numeric improvements in OS were seen in both the squamous and non-squamous histology subgroups. In the squamous histology subgroup, median OS was 21.9 months (95% CI, 15.6–NE) with cemiplimab plus chemotherapy versus 13.8 months (95% CI, 9.3–18.0) in the placebo arm (HR = 0.56; 95% CI, 0.37–0.84). In the non-squamous histology subgroup, median OS was 15.8 months (95% CI, 13.7–NE) with cemiplimab arm versus 13.0 months (95% CI, 10.0–NE) with placebo plus chemotherapy (HR = 0.79; 95% CI, 0.54–1.14). Of note, due to the capping applied to the enrollment of patients with squamous histology, follow-up was shorter in the non-squamous subset (14.7 months; IQR, 12.5–17.9) versus the squamous subset (18.2 months; IQR, 15.9–20.2).

In other predefined subgroups, OS estimates consistently favored cemiplimab plus chemotherapy except in women, never-smokers and patients with PD-L1 < 1% (Fig. 2b). Of note, there was overlap among these three subgroups, which were generally small, and the discrepancies noted in OS were not observed in the other efficacy endpoints of PFS and objective response rate (ORR), which reflect an earlier treatment effect.

### PFS

With 326 events of progressive disease or death, the key secondary endpoint of median PFS with cemiplimab plus chemotherapy was 8.2 months (95% CI, 6.4–9.3) versus 5.0 months (95% CI, 4.3–6.2) for the placebo plus chemotherapy arm (HR = 0.56; 95% CI, 0.44–0.70; *P* < 0.0001). The estimated proportion of patients receiving cemiplimab plus chemotherapy who were alive and had no disease progression at 12 months was 38.1% (95% CI, 32.4–43.8) versus 16.4% (95% CI, 10.5–23.4) for the placebo plus chemotherapy arm (Fig. 2c). PFS benefits also consistently favored cemiplimab plus chemotherapy in all predefined subgroups (Fig. 2d).

### Tumor response

**Fig. 3:**
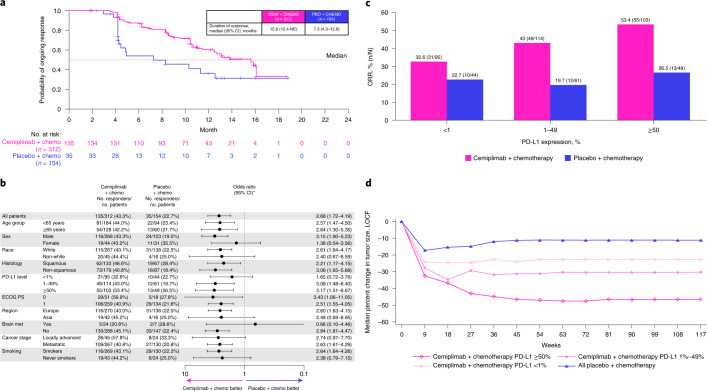
Tumor response data for the cemiplimab plus chemotherapy and placebo plus chemotherapy arms. **a**, Kaplan–Meier curves of DOR in all patients. The median DOR and corresponding two-sided 95% CI were estimated by the Kaplan–Meier method. **b**, Forest plots of objective response in pre-specified subgroups. Odds ratios and corresponding two-sided 95% CIs were calculated using the Cochran–Mantel–Haenszel method. **c**, ORR in correlation with baseline PD-L1 levels. ORR and the corresponding two-sided 95% CI were calculated using the Clopper–Pearson method. **d**, Tumor response in correlation with baseline PD-L1 levels. Median percent change in tumor size over time was calculated descriptively (LOCF). Cemi, cemiplimab; chemo, chemotherapy; LOCF, last observation carried forward; met, metastasis; PBO, placebo.

**Extended Data Fig. 1:**
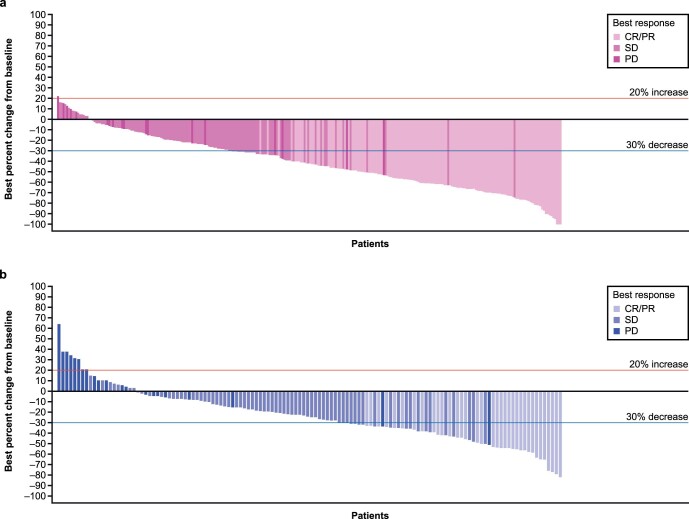
Clinical activity of tumor response in patients with evaluable post-baseline tumor assessment per RECIST 1.1 by independent review committee. **a,** Waterfall plot of tumor response in patients who received cemiplimab plus chemotherapy (n = 312). **b,** Waterfall plot of tumor response in patients who received placebo plus chemotherapy (n = 154). PD, progressive disease; SD, stable disease.

The key secondary endpoint of ORR per independent central review was 43.3% (95% CI, 37.7–49.0) with cemiplimab plus chemotherapy treatment, with complete response (CR) observed in 2.6% of patients (8/312) and partial response (PR) observed in 40.7% of patients (127/312) (Table 2 and Extended Data Fig. 1a). With placebo plus chemotherapy treatment, the ORR was 22.7% (95% CI, 16.4–30.2), and all responses were PR (Table 2 and Extended Data Fig. 1b). The median duration of response (DOR) with cemiplimab plus chemotherapy was 15.6 months (95% CI, 12.4–NE) versus 7.3 months (95% CI, 4.3–12.6) for the placebo plus chemotherapy arm (Fig. 3a). ORR results consistently favored cemiplimab plus chemotherapy in predefined subgroups (Fig. 3b). In the cemiplimab plus chemotherapy group, there was a consistent relationship between ORR and baseline PD-L1 expression (Fig. 3c), with benefits versus placebo plus chemotherapy seen across all levels of baseline PD-L1 expression; there was also a clear association between continuous measure of changes in tumor size over time and baseline PD-L1 expression (Fig. 3d).

**Table 2:** Summary of tumor response per RECIST 1.1 by independent review committee in the intention-to-treat patient population

Response	Cemiplimab + chemotherapy (*n* = 312)	Placebo + chemotherapy (*n* = 154)
Objective response		
Patients, *n*	135	35
% (95% CI)	43.3 (37.7–49.0)	22.7 (16.4–30.2)
Odds ratio (95% CI)	2.68 (1.72–4.19); *P* < 0.0001	
Best overall response, *n* (%)		
Complete response	8 (2.6)	0
Partial response	127 (40.7)	35 (22.7)
Stable disease	121 (38.8)	74 (48.1)
Progressive disease	22 (7.1)	24 (15.6)
NE	30 (9.6)	20 (13.0)
Kaplan–Meier estimated DOR, median (95% CI), months	15.6 (12.4–NE)	7.3 (4.3–12.6)
Observed time to response, median (IQR), months	2.1 (2.0–2.3)	2.1 (2.1–3.9)

Objective response and the corresponding two-sided 95% CI were calculated using the Clopper–Pearson method. The odds ratio and corresponding two-sided 95% CI of the objective response were calculated by the Cochran–Mantel–Haenszel method. The median DOR and corresponding two-sided 95% CI were estimated by the Kaplan–Meier method. Observed time to response and the corresponding IQR were summarized descriptively.

### Patient-reported outcomes

**Extended Data Fig. 2:**
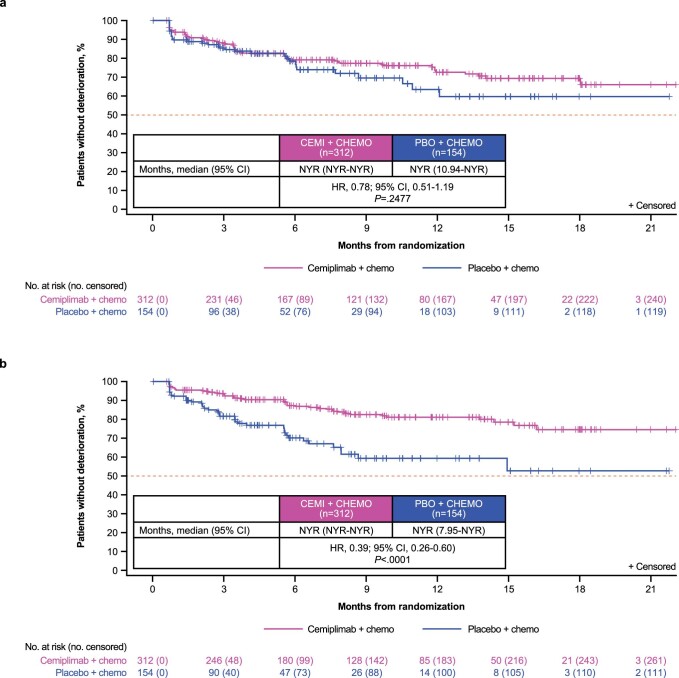
Time to definitive clinically meaningful deterioration in **a**, GHS/QoL and **b**, pain symptoms. Median time to clinically meaningful deterioration in GHS/QoL and pain symptoms, and corresponding two-sided 95% CIs for each, were estimated using the Kaplan–Meier method. Hazard ratios and corresponding two-sided 95% CIs for time to definitive clinically meaningful deterioration were calculated using a stratified Cox proportional hazard model with Efron’s method of tie handling. Cemi, cemiplimab; chemo, chemotherapy; NYR, not yet reached; PBO, placebo.

A significant improvement in the secondary endpoints of overall change from baseline in global health status (GHS)/quality of life (QoL) on the European Organization for Research and Treatment of Cancer Quality of Life-Core 30 (EORTC QLQ-C30) questionnaire was observed in the cemiplimab plus chemotherapy arm (least squares mean change: 1.69; 95% CI, 0.20–3.19) compared to a non-significant overall change in the placebo plus chemotherapy arm (1.08; 95% CI, –1.34 to 3.51). The overall difference between treatment groups was not significant (0.61; 95% CI, –2.23 to 3.45; *P* = 0.673). Compared to placebo plus chemotherapy, cemiplimab plus chemotherapy treatment resulted in a trend toward a delay in the onset of definitive clinically meaningful deterioration according to the GHS/QoL scale (HR = 0.78; 95% CI, 0.51–1.19; *P* = 0.248) (Extended Data Fig. 2a).

There was also a significant overall improvement from baseline in pain symptoms (EORTC QLQ-C30) with cemiplimab plus chemotherapy (–4.52; 95% CI, –6.32 to –2.73) compared to a non-significant overall change with placebo plus chemotherapy (0.46; 95% CI, –2.42 to 3.34). Significant overall difference between treatment groups favored the cemiplimab plus chemotherapy arm (–4.98; 95% CI, –8.36 to –1.60; *P* = 0.004). Compared to placebo plus chemotherapy, cemiplimab plus chemotherapy treatment resulted in a significant delay in the onset of definitive clinically meaningful deterioration according to pain symptoms scale (HR = 0.39; 95% CI, 0.26–0.60; *P* < 0.0001) (Extended Data Fig. 2b).

### Pharmacokinetics and immunogenicity

Cemiplimab concentrations in serum in patients from the cemiplimab plus chemotherapy arm were similar, irrespective of tumor histology type and baseline PD-L1 expression level, and in agreement with those reported for cemiplimab monotherapy. At steady state (week 24; *n* = 177), mean C_max_ (s.d.) was 129 (46.9) mg L, and mean C_trough_ (s.d.) was 48.6 (25.0) mg L.

Immunogenicity was low, with treatment-emergent anti-drug antibodies (ADAs) in 3.5% (7/200) of patients who received cemiplimab plus chemotherapy, at low titer (<1,000) and negative in the neutralizing ADA assay; this did not affect cemiplimab concentrations in serum.

### Safety

The median duration of treatment exposure was 38.5 weeks (IQR, 20.7–63.9) for cemiplimab plus chemotherapy and 21.3 weeks (IQR, 12.0–38.4) for placebo plus chemotherapy (Supplementary Table 2).

Treatment-emergent adverse events (TEAEs) occurred in 95.8% of patients receiving cemiplimab plus chemotherapy; 43.6% of patients experienced grade ≥3 TEAEs, with the most common grade ≥3 TEAEs being anemia (9.9%) and neutropenia (5.8%) (Table 3 and full list in Supplementary Table 3). TEAEs of any grade occurred in 94.1% of patients receiving placebo plus chemotherapy. Grade ≥3 TEAEs occurred in 31.4% of patients, with the most common being anemia (6.5%) and neutropenia (5.9%) (Table 3 and full list in Supplementary Table 3). TEAEs that led to treatment discontinuation occurred in 16 (5.1%) patients in the cemiplimab plus chemotherapy arm and four patients (2.6%) in the placebo plus chemotherapy arm (Table 3). TEAEs of any grade that led to death occurred in 19 patients (6.1%) treated with cemiplimab plus chemotherapy and in 12 patients (7.8%) treated with placebo plus chemotherapy (Table 3).

**Table 3:** TEAEs regardless of attribution

	Cemiplimab + chemotherapy (*n* = 312)		Placebo + chemotherapy (*n* = 153)	
Event, n (%)	Any grade	Grade ≥ 3	Any grade	Grade ≥ 3
Any	299 (95.8)	136 (43.6)	144 (94.1)	48 (31.4)
Led to discontinuation	16 (5.1)	13 (4.2)	4 (2.6)	4 (2.6)
Led to death	19 (6.1)	19 (6.1)	12 (7.8)	12 (7.8)
Events that occurred in ≥10% of patients in either group^a^				
Anemia	136 (43.6)	31 (9.9)	61 (39.9)	10 (6.5)
Alopecia	115 (36.9)	0	66 (43.1)	0
Nausea	78 (25.0)	0	25 (16.3)	0
Hyperglycemia	55 (17.6)	6 (1.9)	18 (11.8)	0
Decreased appetite	53 (17.0)	3 (1.0)	18 (11.8)	0
Alanine aminotransferase increased	51 (16.3)	7 (2.2)	22 (14.4)	3 (2.0)
Arthralgia	48 (15.4)	2 (0.6)	20 (13.1)	0
Neutropenia	48 (15.4)	18 (5.8)	19 (12.4)	9 (5.9)
Aspartate aminotransferase increased	46 (14.7)	1 (0.3)	18 (11.8)	3 (2.0)
Constipation	43 (13.8)	1 (0.3)	17 (11.1)	0
Thrombocytopenia	41 (13.1)	8 (2.6)	19 (12.4)	2 (1.3)
Dyspnea	39 (12.5)	7 (2.2)	10 (6.5)	1 (0.7)
Asthenia	38 (12.2)	6 (1.9)	18 (11.8)	2 (1.3)
Fatigue	38 (12.2)	7 (2.2)	11 (7.2)	1 (0.7)
Vomiting	38 (12.2)	0	15 (9.8)	0
Weight decreased	35 (11.2)	4 (1.3)	13 (8.5)	0
Insomnia	34 (10.9)	0	11 (7.2)	0
Diarrhea	33 (10.6)	4 (1.3)	10 (6.5)	0
Hypoalbuminemia	32 (10.3)	2 (0.6)	9 (5.9)	0

The safety population includes all randomized patients who received at least one dose of any study drug. The events are listed in descending order of frequency in the cemiplimab plus chemotherapy arm. Events were coded according to the Preferred Terms of the Medical Dictionary for Regulatory Activities version 22.1. Severity of adverse events was graded according to the National Cancer Institute Common Terminology Criteria for Adverse Events version 4.03.

^a^The events are listed in descending order of frequency in the cemiplimab + chemotherapy group.

Treatment-related adverse events (TRAEs) occurred in 88.1% of patients treated with cemiplimab plus chemotherapy and in 84.3% of patients treated with placebo plus chemotherapy. TRAEs are summarized in Supplementary Table 4.

Sponsor-identified immune-related adverse events (irAEs) occurred in 18.9% of patients treated with cemiplimab plus chemotherapy, with grade ≥3 irAEs occurring in 2.9% of patients (Supplementary Table 5). Three patients (1.0%) discontinued cemiplimab plus chemotherapy due to an irAE, and one patient (0.3%) died due to immune-mediated pneumonitis (Supplementary Table 5).

## Discussion

EMPOWER-Lung 3 part two was stopped early per recommendation of the IDMC, based on meeting preset OS efficacy criteria, resulting in a primary analysis in which cemiplimab plus chemotherapy showed superior efficacy versus placebo plus chemotherapy in first-line treatment of aNSCLC as measured by OS (primary endpoint) and PFS and ORR (key secondary endpoints). A median OS of 21.9 months was achieved after cemiplimab plus chemotherapy, with a reduced the risk of death by 29% versus placebo plus chemotherapy. Cemiplimab plus chemotherapy was also associated with higher median PFS (8.2 months versus 5.0 months), ORR (43.3% versus 22.7%) and DOR (15.6 months versus 7.3 months) versus placebo plus chemotherapy. Overall change from baseline and time to definitive clinically meaningful deterioration in patient-reported pain symptoms were superior with cemiplimab plus chemotherapy. Cemiplimab is only the second anti–PD-1/PD-L1 agent to show efficacy in advanced NSCLC as both monotherapy and in combination with chemotherapy for both squamous and non-squamous histologies^6,12^.

EMPOWER-Lung 3 part two was designed to efficiently evaluate the efficacy of cemiplimab in combination with chemotherapy in a single phase 3 study of patients with both squamous and non-squamous NSCLC. This obviated the need to conduct separate clinical trials for these two main tumor histology categories, avoiding a distinction that is not rooted in clinical practice. Using this practical design, the study was powered to detect statistically robust results in the overall population irrespective of histology and PD-L1 expression levels. In addition, the patient eligibility criteria of this study were designed to closely resemble the real-world patient population undergoing first-line treatment for advanced NSCLC, including patients with unresectable locally advanced disease not suitable for definitive chemoradiation; patients with previously treated and controlled brain metastases (symptoms were controlled without immunosuppressive doses of steroids, as is most often done in clinical practice, without mandatory radiological evidence of response to treatment); patients with known controlled viral infections (for example, hepatitis B virus, hepatitis C virus or HIV); and patients who were never smokers. Consistent with these broad inclusion criteria, most patients enrolled in this study had an ECOG PS of 1 (84.3%), which was higher than those enrolled in trials in similar settings^2,3^.

In this study, most patients were enrolled in Central and Eastern Europe, where smoking is more common than in the United States, especially among men. Consequently, the number of men enrolled was higher than women, consistent with the men-to-women ratio (2:1) of lung cancer incidence in Eastern Europe^13^. The absence of enrollment in the United States and in Western Europe was due to the availability of an approved anti–PD-1 therapy in combination with platinum-doublet chemotherapy for patients with aNSCLC, irrespective of PD-L1 expression, at the time of the present study^4,14^. Despite differing geographic areas of enrollment, patient characteristics were similar and results were generalizable. Median PFS and OS in the control arm and the safety profile observed in this study were consistent with those observed in studies conducted across various geographies, including those conducted predominantly in Western Europe and the United States^3,15^.

Median OS was greater with cemiplimab plus chemotherapy than with placebo plus chemotherapy in the overall population and across most subgroups, except for patients with PD-L1 < 1%, never smokers and women. These three subgroups were relatively small, overlapping and underpowered for OS assessment; additionally, in these subgroups, HR point estimates for earlier endpoints such as PFS and ORR were all less than 1, and ORR was consistently superior to chemotherapy alone. Given that cemiplimab plus chemotherapy showed consistently superior PFS and ORR in all subgroups, longer-term follow-up data are awaited to further inform OS results.

Cemiplimab plus chemotherapy demonstrated a favorable benefit–risk profile. The incidence of TEAEs was similar between treatment arms, although incidence of grade ≥3 TEAEs was higher with cemiplimab plus chemotherapy (43.6%, 136/312 patients) versus placebo plus chemotherapy (31.4%, 48/153 patients). Low rates of adverse events leading to discontinuation were seen in both treatment arms, and the safety profile was generally consistent with what has been reported for cemiplimab as monotherapy and for platinum-based chemotherapy^8^. Maintenance in GHS and QoL as well as improvements in pain symptoms indicated that cemiplimab plus chemotherapy demonstrated a good benefit–risk profile that does not impose toxicities that interfere with QoL in patients with aNSCLC.

EMPOWER-Lung 3 addresses an unmet clinical need for patients with locally advanced disease who are not candidates for surgical resection or definitive chemoradiation. NCCN guidelines recommend that patients with unresectable stage IIIA and IIIB NSCLC receive definitive concurrent chemoradiation followed by consolidation with durvalumab (anti-PD-L1)^1^. However, for patients who are not candidates for concurrent chemoradiation, platinum-based chemotherapy remains the only standard of care available. EMPOWER-Lung 3 included patients with locally advanced disease who are not candidates for definitive chemoradiation (14.8% of the total patient population), thus providing prospective data to guide treatment for these patients rather than extrapolating from stage IV disease, as is often done in practice^2,6^. Therefore, this study fills a gap in the available evidence that is important for clinical practice and establishes a potential new standard-of-care treatment option for these patients.

The results of EMPOWER-Lung 3 demonstrate that cemiplimab in combination with platinum-doublet chemotherapy is a potential first-line treatment option for patients with advanced squamous and non-squamous NSCLC, regardless of PD-L1 expression level and without *EGFR*, *ALK* or *ROS1* aberrations.

## Methods

### Patients

Adult patients with squamous or non-squamous NSCLC and any PD-L1 expression level were enrolled. The number of patients with squamous histology was capped per protocol at 50%. PD-L1 subgroups were also capped to ensure a homogenous representation of all PD-L1 levels. PD-L1 levels were capped as follows: at least 30% but no more than 40% of patients enrolled must have tumors that express PD-L1 in ≥50% of tumor cells; enrollment of patients whose tumors express PD-L1 in <1% of tumor cells will be capped at 30%; and enrollment of patients with tumors that express PD-L1 in <50% of tumor cells will be capped at 70%.

Inclusion criteria included men and women ≥18 years of age (≥20 years of age for Japanese patients); availability of an archival or on-study-obtained formalin-fixed, paraffin-embedded tumor tissue sample; at least one radiographically measurable lesion per Response Evaluation Criteria in Solid Tumors version 1.1 (RECIST 1.1); histologically or cytologically confirmed squamous or non-squamous stage IIIB/C (if deemed not candidates for treatment with definitive chemoradiation) or stage IV NSCLC; ECOG PS ≤1; anticipated life expectancy of at least 3 months; adequate organ and bone marrow function; willingness and ability to comply with clinic visits and study-related procedures; provided signed informed consent; and ability to understand and complete study-related questionnaires.

Exclusion criteria included active or untreated brain metastases or spinal cord compression (patients with adequately treated and clinically stable brain metastases were eligible); tumors positive for *EGFR* mutations, *ALK* translocations or *ROS1* fusions; encephalitis, meningitis or uncontrolled seizures in the year before enrollment; history of interstitial lung disease or of active, non-infectious pneumonitis that required immunosuppressive doses of glucocorticoids to assist with management or of pneumonitis within the last 5 years; ongoing or recent evidence of substantial autoimmune disease that required treatment with systemic immunosuppressive treatments; active or suspected autoimmune disease that required systemic treatment; corticosteroid therapy within 14 days of randomization; another malignancy that is progressing or requires treatment (exception of non-melanoma skin cancer that has undergone potentially curative therapy or any other localized tumor that has been treated, and the patient was deemed to be in complete remission for at least 2 years before enrollment); active hepatitis B or C; prior anti–PD-1/PD-L1 therapy; treatment-related immune-mediated adverse events from immune-modulatory agents; receipt of an investigational drug or device within 30 days of enrollment; receipt of a live vaccine within 30 days of planned start of study drug; major surgery or substantial traumatic injury within 4 weeks before the first dose; documented allergic or acute hypersensitivity reaction attributed to antibody treatments; known psychiatric or substance abuse disorder; pregnant or breastfeeding women; and sexually active adults of childbearing potential who were unwilling to practice highly effective contraception before the start of treatment. Of note, never smokers, defined as individuals who had smoked fewer than 100 cigarettes in their lifetime, were allowed in the study.

### Study design and treatment

EMPOWER-Lung 3 (NCT03409614) is a two-part, randomized, phase 3 study (see Supplementary Information for full study protocol). This manuscript reports results from EMPOWER-Lung 3 part two, which compared cemiplimab plus chemotherapy versus placebo plus chemotherapy in patients with aNSCLC and any PD-L1 expression level. The time point for primary analysis has been reached for part two, and the results are reported here. Part one is considered a separate study evaluating cemiplimab plus abbreviated chemotherapy and ipilimumab or cemiplimab plus chemotherapy compared to platinum-doublet chemotherapy alone in patients with aNSCLC whose tumors express PD-L1 in <50% of tumor cells (part one is ongoing, and results will be reported separately).

Patients were randomly assigned 2:1 via an interactive web response system and stratified by histology and PD-L1 expression (<1%, 1–49% and ≥50% as measured using a PD-L1 immunohistochemistry assay) to receive either cemiplimab 350 mg once every 3 weeks or placebo every 3 weeks in combination with four cycles of chemotherapy. Investigators’ choice of histology-specific chemotherapy options included paclitaxel plus carboplatin, paclitaxel plus cisplatin, pemetrexed plus carboplatin and pemetrexed plus cisplatin (Supplementary Table 6). Patients were treated for a maximum of 108 weeks, or until disease progression or unacceptable toxicity. Maintenance pemetrexed was mandatory for patients with non-squamous histology assigned to a pemetrexed-containing regimen.

Patients reserved the right to withdraw from the study at any time for any reason. Criteria for discontinuation of treatment included toxicity that did not resolve within 84 days of last treatment infusion; any severe or life-threatening event; pregnancy; and a grade ≥3 infusion reaction during or directly after treatment infusions.

Major protocol amendments that occurred during the study (EMPOWER-Lung 3 part two) included updating PFS to be a key secondary endpoint instead of being a primary endpoint; adding an interim analysis at 50% OS events in addition to the planned interim analysis at 70% OS events; and adding a clarification that the superiority or futility of cemiplimab treatment will be decided at the interim analysis if the statistical boundary is crossed, because a two-sided test is used.

### Endpoints

The primary endpoint, OS, was defined as the time from randomization to the date of patient death. Key secondary endpoints included PFS, defined as the time from randomization to the date of the first documented tumor progression (as determined by the blinded independent review committee) or death, whichever is earlier, and ORR, defined as the proportion of patients with a best overall response of confirmed CR or PR, per blinded independent review committee. Patient-reported outcomes (PROs) were measured as predefined secondary endpoints using multiple instruments, including the EORTC QLQ-C30. Secondary endpoints also included pharmacokinetics (PK), as measured by concentrations of cemiplimab in serum over time, and immunogenicity, as measured by ADAs.

All efficacy endpoints were assessed in the intention-to-treat population. Safety was assessed in all randomized patients who received at least one dose of the assigned treatment.

Clinical data were captured in the clinical database using the Medidata Rave Electronic Capture Data system (version 2021.2.0).

### Trial oversight

The protocol and all amendments were approved by the appropriate institutional review board or independent ethics committee at each participating study site (Supplementary Table 7). The study was conducted in accordance with the principles of the Declaration of Helsinki and the International Conference on Harmonization Good Clinical Practice guidelines.

All patients provided written informed consent before enrollment. The study was sponsored by Regeneron Pharmaceuticals and Sanofi and was designed by employees of Regeneron Pharmaceuticals in collaboration with the investigators.

Treatment for part two of the study was double-blinded except for an unblinded pharmacist at each site. Patients, the principal investigators and study site personnel (apart from the unblinded investigative site pharmacist) remained blinded to all randomization assignments throughout the study.

A blinded independent review committee, independent of the sponsor and the study investigators, reviewed all tumor assessments to determine tumor response per RECIST 1.1. An IDMC reviewed unblinded safety and efficacy data, including all available tumor assessments, for efficacy analyses.

The data were collected by investigators, analyzed by statisticians employed by the sponsors, and interpreted by the authors, including employees of the sponsors. Authors had full access to the data and were responsible for all content and editorial decisions.

### Assessments

Baseline assessments included collection of tumor tissue samples for evaluation of PD-L1 expression. Formalin-fixed, paraffin-embedded tumor samples were assessed at a central laboratory using the SP263 assay^16^. Tumor tissue samples were also tested for *EGFR*, *ALK* and *ROS1* genomic tumor aberrations by a central laboratory.

Radiographic tumor assessments were performed every 9 weeks for the first year, beginning at week 9, and then every 12 weeks beginning at week 55 (during the second year) until disease progression, withdrawal of consent, death or initiation of another anti-cancer treatment. Adverse events and laboratory abnormalities were graded according to the National Cancer Institute Common Terminology Criteria for Adverse Events version 4.03. The full assessment schedule is available in the study protocol. Responses were assessed by RECIST 1.1 criteria.

PROs were measured with multiple instruments including the EORTC QLQ-C30 questionnaire at baseline, at the beginning of each treatment cycle for the first six doses and then at the start of every three cycles and at the end of treatment. EORTC QLQ-C30 is a 30-item questionnaire composed of five multiple-item functional subscales, three multiple-item symptom scales, a GHS/QoL subscale and six single-item symptom scales assessing other cancer-related symptoms. Responses to all items are converted to a 0–100 scale with a standard scoring algorithm. For GHS/QoL, higher scores indicate better QoL. For the pain symptoms scale, higher scores indicate greater severity of symptoms. Hence, a negative change from baseline in pain symptoms score reflects an improvement, and a positive change reflects a deterioration. Conversely, a negative change from baseline in GHS/QoL score reflects a deterioration, and a positive change reflects an improvement.

Cemiplimab concentrations (PK), immunogenicity (ADAs), and neutralizing anti-cemiplimab antibodies (NAbs) were measured in serum from blood samples collected pre-dose and at various times throughout the treatment and follow-up periods. A validated ELISA with a lower limit of quantitation (LLOQ) of 0.078 mg L was used to measure cemiplimab concentrations in serum. Immunogenicity was assessed using a validated electrochemiluminescence bridging immunoassay for ADAs and a validated competitive ligand-binding assay for NAbs measured in ADA-positive serum samples only.

### Statistical analysis

We estimated that a sample size of 450 randomized patients would yield approximately 93% power to detect a statistically significant difference in OS at a two-sided type 1 error level of 0.05 between the two treatment arms. Timing of the second interim analysis was pre-specified to occur when approximately 204 deaths (70% of total OS events) were observed. An IDMC reviewed the results of this second interim analysis based on a Lan–DeMets approach to the O’Brien–Fleming alpha-spending function and concluded that statistical significance was demonstrated for OS. The IDMC recommended that the study be unblinded, and the sponsor accepted the recommendation, concluding the study at this second interim analysis and designating these data as the primary analysis. All statistical analyses were performed using SAS software version 9.4 or above.

OS was analyzed by stratified log-rank test using histology and PD-L1 expression level as stratification factors. HRs and 95% CIs were estimated by a stratified Cox regression model using the treatment as covariate and adjusted by the same stratification factors.

PFS was analyzed using the same statistical method as in the OS analysis. ORR was analyzed using the Cochran–Mantel–Haenszel test stratified by histology and PD-L1 expression. DOR was analyzed using the Kaplan–Meier method for each treatment arm.

The primary endpoint of OS and secondary endpoints PFS and ORR were tested hierarchically, in the order of OS, PFS and ORR.

Pre-specified PRO analyses included overall change from baseline, estimated by a mixed-effects model for repeated measures, and the time to definitive clinically meaningful deterioration, analyzed by a stratified log-rank test and summarized by Kaplan–Meier estimation. Time to definitive clinically meaningful deterioration for the GHS/QoL scale was defined as the time from randomization to the first observation with a ≥10-point decrease and no subsequent observations with a decrease of <10 points from baseline^17^ or if patient dropout resulted in missing data. Time to definitive clinically meaningful deterioration for the pain symptoms scale was defined as the time from randomization to the first observation with a ≥10-point increase from baseline and no subsequent observations with an increase of <10 points from baseline^17^ or if patient dropout resulted in missing data.

### Reporting summary

Further information on research design is available in the Nature Research Reporting Summary linked to this article.

## Online content

Any methods, additional references, Nature Research reporting summaries, source data, extended data, supplementary information, acknowledgements, peer review information; details of author contributions and competing interests; and statements of data and code availability are available at 10.1038/s41591-022-01977-y.

## Supplementary information

Supplementary Information Supplementary Tables 1–7.

Reporting Summary

## Data Availability

Qualified researchers may request access to study documents (including the clinical study report, study protocol with any amendments, blank case report form and statistical analysis plan) that support the methods and findings reported in this manuscript. Individual anonymized participant data will be considered for sharing once the product and indication has been approved by major health authorities (for example, the US Food & Drug Administration, the European Medicines Agency and the Pharmaceuticals and Medical Devices Agency), if there is legal authority to share the data and there is not a reasonable likelihood of participant re-identification. Submit requests to https://vivli.org/.
