# Supplementary material for: Cemiplimab plus chemotherapy versus chemotherapy alone in non-small cell lung cancer: a randomized, controlled, double-blind phase 3 trial
Source: Nat Med. 2022 Aug 25;28(11):2374–80. doi: 10.1038/s41591-022-01977-y (PMC9671806; doi:10.1038/s41591-022-01977-y)
Supplement: Supplementary file 1 — Supplementary Tables 1–7. [file 41591_2022_1977_MOESM1_ESM.pdf]

nature portfolio

https://doi.org/10.1038/s41591-022-01977-y

Supplementary information

Cemiplimab plus chemotherapy versus chemotherapy alone in non-small cell lung cancer: a randomized, controlled, double-blind phase 3 trial

In the format provided by the authors and unedited

Supplementary Information

Supplementary Table 1. List of investigators 2

Supplementary Table 2. Summary of treatment exposure in the safety population. .. 9

Supplementary Table 3. Treatment-emergent adverse events (TEAEs) regardless of attribution. 10

Supplementary Table 4. Treatment-related adverse events in the safety population. 29

Supplementary Table 5. Sponsor-identified immune-related adverse events. 41

Supplementary Table 6. Investigator's choice of chemotherapy options. 43

Supplementary Table 7. List of institutional review boards and independent ethics committees that approved the clinical trial protocol 44

Original Protocol 53

Protocol Amendment 5 (current) 189

Summary of Protocol Changes 341

Statistical Analysis Plan Primary Analysis (current) 359

Supplementary Table 1. List of investigators

Country	Site name	Principal investigator	Patients enrolled, n
China	Shanghai Pulmonary Hospital	Zhou, Caicun	6
	Beijing YouYi Hospital-Beijing Friendship Hospital Capital Medical University	Cao, Bangwei	3
	Shandong University - Jinan Central Hospital	Sun, Meili (current)<br>Sun, Yuping (previous)	2
	Affiliated Hospital of Hebei University	Zang, Aimin	3
	The Second Affiliated Hospital of Zhejiang University School of Medicine	Shen, Hong	1
	Huadong Hospital	Zhu, Huili	1
	Henan Provincial People's Hospital	Cang, Shundong	2
	Xiangyang Central Hospital	Yi, Tienan	1
	The First Affiliated Hospital, School of Clinical Medicine of Guangdong Pharmaceutical University	Wang, Xicheng	2
	Henan Cancer Hospital	Zhao, Yanqiu	1

	Hangzhou First People's Hospital - Hangzhou Cancer Hospital (The Fourth People's Hospital of Hangzhou)	Ma, Shenglin	2
	Liaoning Cancer Hospital & Institute	Ma, Rui	1
	Zhejiang Hospital	Zhang, Yu	1
Georgia	High Technology Medical Center, University Clinic Tbilisi	Gogishvili, Miranda	52
	LTD Cancer Center of Adjara Autonomic Republic	Makharadze, Tamta	34
	JSC Neo Medi	Shavdia, Mikheil	8
	LTD Institute of Clinical Oncology	Nemsadze, Gia	8
	LTD Acad. F.Todua Medical Center-LTD Research Institute of Clinical Medicine	Melkadze, Tamar	46
	Multiprofile Clinic Consilium Medulla	Giorgadze, Davit	23
Greece	Sotiria General Hospital of Athens	Syrigos, Konstantinos	5
	Euromedica General Clinic	Fountzilas, George	3
	Ongology Theageneio Thessaloniki Al.	Tsiouda, Theodora	4

	University General Hospital of Larissa	Kotsakis, Athanasios	1
Malaysia	Hospital Sultan Ismail	Lim, Chun Sen	5
	Pantai Hospital Kuala Lumpur	Yusof, Mastura	2
	Hospital Pulau Pinang	Ai Lian, Tan	1
	Hospital Tengku Ampuan Afzan (HTAA) - International Islamic University Malaysia (IIUM)	Soon Hin, How	7
	University Malaya Medical Centre (UMMC)	Gwo Fuang, Ho	4
	Mount Miriam Cancer Hospital	Raman, Rakesh	2
Poland	Instytut MSF Sp. z o.o	Kalinka-Warzocha, Ewa	5
	Medpolonia Sp. z o.o.	Ramlau, Rodryg	2
	Mandziuk Slawomir - Specjalistyczna Praktyka Lekarska	Mandziuk, Slawomir	2
	Wojewodzki Szpital Zespolony im. L. Rydygiera w Toruniu Szpital Obserwacyjno-Zakazny Oddzial Chemioterapii Nowotworow	Sawrycki, Piotr	7
	Wojewodzki Szpital Chorob Pluc im. dr Alojzego Pawelca	Gabrys, Jacek	1

	Szpitale Pomorskie Sp. z o.o.	Danielewicz, Iwona	8
	Mazowieckie Centrum Leczenia Chorob Pluc i Gruzlicy	Szczesna, Aleksandra	13
	MRUKMED. Lekarz Beata Madej Mruk i Partner. Sp. p. Oddzial nr 1 w Rzeszowie	Mruk, Andrzej	2
	Samodzielny Publiczny Zespol Gruzlicy i Chorob Pluc w Olsztynie	Kazarnowicz, Andrzej	3
Romania	Onco Clinic Consult SA	Visan, Patricia	1
	Centrul de Oncologie Sf. Nectarie S.R.L	Schenker, Michael	7
	S.C. Medisprof S.R.L	Udrea, Adrian	1
Russia	Ekaterinburg Regional Oncology Center	Bentsion, Dmitriy	1
	RBHI Kursk Oncological Research and Clinical Center named after G. E. Ostroverkhova Health Committee of the Kursk region	Lifirenko, Igor	4
	City Clinical Oncology Dispensary	Orlova, Rashida	3
	Arkhangelsk Regional Clinical Oncology Dispensary	Nechaeva, Marina	12

	"FSBI" "Scientific Research Institute of Oncology n. a. N. N. Petrov"	Protsenko, Svetlana	3
	National Research Mordoviz State University	Skopin, Pavel	5
	State Healthcare Institution Oncology Dispensary 2 Ministry of Healthcare of Krasnodar Region	Kirtbaya, Dmitry (current)<br><br>Udovitsa, Dmitriy (previous)	3
	Siberian State Medical University	Kirillova, Natalia	2
	State Budgetary Healthcare Institution of Kaluga Region Kaluga Regional Clinical Oncology Dispensary	Rozhkova, Irina	11
	FSBSI Russian Cancer Research Center n.a. N.N.Blokhin	Laktionov, Konstantin	14
	EVIMED LLC	Gladkov, Oleg	3
	Private medical institution Euromedservice	Penkov, Konstantin	17
	State Autonomous Healthcare Institution Republican Clinical Oncological Dispensary of the Ministry of Healthcare of the Republic	Sakaeva, Dina Damirovna	3

	of Bashkortostan (GAUZ RKOD MINZDRAVARB)		
	Leningrad Regional Oncological Dispensary	Smagina, Maria	3
	Republican Clinical Oncology Dispensary	Mukhametshina, Guzel	9
	Piatigorsky Oncology Center	Vladimirov, Vladimir	2
	Belgorod Regional Oncology Dispensary	Kuzina, Ludmila	5
	Budgetary Healthcare Institution of Omsk Region Clinical Oncology Dispensary	Dvorkin, Mikhail	38
	University Headache Clinic LLC	Ledin, Evgeny	3
Thailand	Udonthani Cancer Hospital	Butthongkomvong, Kritiya	1
	Lopburi Cancer Hospital	Pornpraserthsuk, Piti	1
	Phramongkutklao College of Medicine	Prasongsook, Naiyarat	5
	Lampang Cancer Hospital	Sorrarittichingchai, Sirikul	1

	Prince of Songkla Hospital, Prince Of Songkhla University	Sunpaweravong, Patrapim	2
	Chiangrai Prachanukroh Hospital	Tharavichitkul, Ekkapong	1
Turkey	Ministry of Health Göztepe Prof. Dr. Süleyman Yalçın City Hospital	Gumus, Mahmut	6
	Hacettepe Universitesi Tip Fakultesi Onkoloji Hastanesi	Turker, Fatma (current)<br>Kilickap, Saadettin (previous)	5
	Baskent Universitesi Tip Fakultesi Adana Hastanesi	Sezer, Ahmet	8
	Bezmialem Vakif Universitesi Tip Fakultesi	Turk, Haci Mehmet	3
Ukraine	Dnepropetrovsk State Medical Academy	Bondarenko, Igor	1
	Private Enterprise of Private Manufacturing Company Acinus, Medical and Diagnostic Center	Ursol, Grygorii	1
	Municipal Non-Profit Enterprise Regional Oncology Center	Kobziev, Oleg	5
	Vinnitsa Regional Clinical Oncological Center	Kostiuk, Oleksandr	2

Supplementary Table 2. Summary of treatment exposure in the safety population.

The safety population includes all randomized patients who received at least one dose of any study drug.

	**Cemiplimab + chemotherapy (n=312)**	**Placebo + chemotherapy (n=153)**
Duration of exposure, median (range), weeks	38.5 (1.4–102.6)	21.3 (0.6–95.0)
Duration of exposure, n (%)		
<6 weeks	22 (7.1)	12 (7.8)
6–12 weeks	22 (7.1)	25 (16.3)
12–18 weeks	20 (6.4)	18 (11.8)
18–36 weeks	69 (22.1)	52 (34.0)
36–54 weeks	72 (23.1)	25 (16.3)
54–72 weeks	58 (18.6)	13 (8.5)
72–96 weeks	45 (14.4)	8 (5.2)
96–108 weeks	4 (1.3)	0
≥108 weeks	0	0

Supplementary Table 3. Treatment-emergent adverse events (TEAEs) regardless of attribution.

The safety population includes all randomized patients who received at least one dose of any study drug. Events were coded according to the Preferred Terms of the Medical Dictionary for Regulatory Activities (MedDRA), version 22.1. Severity of adverse events was graded according to the National Cancer Institute Common Terminology Criteria for Adverse Events, version 4.03.

	Cemiplimab + chemotherapy (n=312)		Placebo + chemotherapy (n=153)	
**Event, n (%)**	**Any grade**	**Grade ≥3**	**Any grade**	**Grade ≥3**
Any	299 (95.8)	136 (43.6)	144 (94.1)	48 (31.4)
Led to discontinuation	16 (5.1)	13 (4.2)	4 (2.6)	4 (2.6)
Led to death	19 (6.1)	19 (6.1)	12 (7.8)	12 (7.8)
Anemia	136 (43.6)	31 (9.9)	61 (39.9)	10 (6.5)
Alopecia	115 (36.9)	0	66 (43.1)	0
Nausea	78 (25.0)	0	25 (16.3)	0
Hyperglycemia	55 (17.6)	6 (1.9)	18 (11.8)	0
Decreased appetite	53 (17.0)	3 (1.0)	18 (11.8)	0
Alanine aminotransferase increased	51 (16.3)	7 (2.2)	22 (14.4)	3 (2.0)
Arthralgia	48 (15.4)	2 (0.6)	20 (13.1)	0
Neutropenia	48 (15.4)	18 (5.8)	19 (12.4)	9 (5.9)
Aspartate aminotransferase increased	46 (14.7)	1 (0.3)	18 (11.8)	3 (2.0)
Constipation	43 (13.8)	1 (0.3)	17 (11.1)	0

Thrombocytopenia	41 (13.1)	8 (2.6)	19 (12.4)	2 (1.3)
Dyspnea	39 (12.5)	7 (2.2)	10 (6.5)	1 (0.7)
Asthenia	38 (12.2)	6 (1.9)	18 (11.8)	2 (1.3)
Fatigue	38 (12.2)	7 (2.2)	11 (7.2)	1 (0.7)
Vomiting	38 (12.2)	0	15 (9.8)	0
Weight decreased	35 (11.2)	4 (1.3)	13 (8.5)	0
Insomnia	34 (10.9)	0	11 (7.2)	0
Diarrhea	33 (10.6)	4 (1.3)	10 (6.5)	0
Hypoalbuminemia	32 (10.3)	2 (0.6)	9 (5.9)	0
Blood creatinine increased	31 (9.9)	1 (0.3)	8 (5.2)	0
Hypothyroidism	28 (9.0)	1 (0.3)	4 (2.6)	0
Peripheral sensory neuropathy	28 (9.0)	0	15 (9.8)	0
Pneumonia	25 (8.0)	9 (2.9)	7 (4.6)	5 (3.3)
Weight increased	24 (7.7)	0	2 (1.3)	0
White blood cell count decreased	23 (7.4)	10 (3.2)	6 (3.9)	3 (2.0)
Amylase increased	22 (7.1)	3 (1.0)	5 (3.3)	0
Blood lactate dehydrogenase increased	22 (7.1)	0	6 (3.9)	0
Hemoptysis	22 (7.1)	1 (0.3)	12 (7.8)	0
Blood urea increased	21 (6.7)	0	7 (4.6)	0
Cough	20 (6.4)	1 (0.3)	8 (5.2)	0
Hypokalemia	20 (6.4)	3 (1.0)	7 (4.6)	2 (1.3)
Rash	20 (6.4)	2 (0.6)	6 (3.9)	0
Hypocalcemia	19 (6.1)	1 (0.3)	11 (7.2)	1 (0.7)
Pyrexia	19 (6.1)	1 (0.3)	8 (5.2)	0

Hyperthyroidism	18 (5.8)	1 (0.3)	4 (2.6)	0
Leukopenia	18 (5.8)	3 (1.0)	10 (6.5)	2 (1.3)
Neuropathy peripheral	18 (5.8)	0	6 (3.9)	0
Blood alkaline phosphatase increased	17 (5.4)	0	14 (9.2)	0
Platelet count decreased	17 (5.4)	3 (1.0)	6 (3.9)	0
Back pain	15 (4.8)	0	12 (7.8)	0
Blood thyroid-stimulating hormone increased	15 (4.8)	0	1 (0.7)	0
Blood uric acid increased	15 (4.8)	0	7 (4.6)	1 (0.7)
Dizziness	15 (4.8)	0	2 (1.3)	0
Hyperkalemia	15 (4.8)	0	7 (4.6)	3 (2.0)
Hyperuricemia	15 (4.8)	1 (0.3)	3 (2.0)	0
Hyponatremia	15 (4.8)	9 (2.9)	3 (2.0)	2 (1.3)
Lipase increased	15 (4.8)	1 (0.3)	2 (1.3)	0
Pain in extremity	15 (4.8)	0	15 (9.8)	0
Paresthesia	15 (4.8)	0	4 (2.6)	0
Hypertension	14 (4.5)	2 (0.6)	4 (2.6)	1 (0.7)
Noncardiac chest pain	14 (4.5)	1 (0.3)	9 (5.9)	0
Pruritus	14 (4.5)	0	3 (2.0)	0
Hypomagnesemia	13 (4.2)	0	1 (0.7)	1 (0.7)
Pneumonitis	13 (4.2)	1 (0.3)	1 (0.7)	0
Neutrophil count decreased	12 (3.8)	8 (2.6)	5 (3.3)	3 (2.0)
Lymphocyte count decreased	11 (3.5)	3 (1.0)	4 (2.6)	0
Myalgia	11 (3.5)	0	4 (2.6)	0
Bone pain	10 (3.2)	2 (0.6)	6 (3.9)	0

Headache	10 (3.2)	0	2 (1.3)	0
Mood altered	10 (3.2)	0	1 (0.7)	0
Edema peripheral	10 (3.2)	1 (0.3)	4 (2.6)	0
Stomatitis	10 (3.2)	2 (0.6)	1 (0.7)	0
Abdominal pain	9 (2.9)	0	2 (1.3)	0
COVID-19	9 (2.9)	2 (0.6)	2 (1.3)	0
Hypercalcemia	9 (2.9)	1 (0.3)	5 (3.3)	2 (1.3)
Abdominal pain upper	8 (2.6)	1 (0.3)	1 (0.7)	0
Acute kidney injury	7 (2.2)	0	2 (1.3)	1 (0.7)
Blood thyroid-stimulating hormone decreased	7 (2.2)	0	4 (2.6)	0
Dysphagia	7 (2.2)	0	2 (1.3)	0
Respiratory tract infection	7 (2.2)	0	2 (1.3)	0
Thrombocytosis	7 (2.2)	0	1 (0.7)	0
Vertigo	7 (2.2)	0	3 (2.0)	0
Blood bilirubin increased	6 (1.9)	1 (0.3)	4 (2.6)	0
Dry skin	6 (1.9)	0	4 (2.6)	0
Lacrimation increased	6 (1.9)	0	2 (1.3)	0
Peripheral swelling	6 (1.9)	0	2 (1.3)	0
Blood pressure increased	5 (1.6)	0	0	0
Death	5 (1.6)	5 (1.6)	0	0
Dyspepsia	5 (1.6)	0	2 (1.3)	0
Hyperbilirubinemia	5 (1.6)	0	1 (0.7)	0
Hypoproteinemia	5 (1.6)	0	1 (0.7)	0
Leukocytosis	5 (1.6)	0	4 (2.6)	0
Polyneuropathy	5 (1.6)	0	4 (2.6)	0

Rash maculopapular	5 (1.6)	1 (0.3)	0	0
Urinary tract infection	5 (1.6)	2 (0.6)	1 (0.7)	0
Blood potassium increased	4 (1.3)	1 (0.3)	1 (0.7)	1 (0.7)
Bronchitis chronic	4 (1.3)	1 (0.3)	0	0
COVID-19 pneumonia	4 (1.3)	3 (1.0)	1 (0.7)	1 (0.7)
Conjunctivitis	4 (1.3)	0	2 (1.3)	0
Febrile neutropenia	4 (1.3)	4 (1.3)	4 (2.6)	4 (2.6)
Gastritis	4 (1.3)	0	0	0
Hypoesthesia	4 (1.3)	0	0	0
Hypotension	4 (1.3)	1 (0.3)	1 (0.7)	0
Muscular weakness	4 (1.3)	1 (0.3)	3 (2.0)	0
Productive cough	4 (1.3)	0	5 (3.3)	0
Pulmonary embolism	4 (1.3)	2 (0.6)	2 (1.3)	2 (1.3)
Sinus tachycardia	4 (1.3)	0	0	0
Tachycardia	4 (1.3)	0	1 (0.7)	0
Upper respiratory tract infection	4 (1.3)	0	4 (2.6)	0
Abdominal distension	3 (1.0)	0	1 (0.7)	0
Atrial fibrillation	3 (1.0)	1 (0.3)	1 (0.7)	1 (0.7)
Bilirubin conjugated increased	3 (1.0)	0	0	0
Bronchitis	3 (1.0)	1 (0.3)	1 (0.7)	1 (0.7)
Chronic kidney disease	3 (1.0)	1 (0.3)	2 (1.3)	1 (0.7)
Colitis	3 (1.0)	1 (0.3)	0	0
Dysphonia	3 (1.0)	0	0	0
Hepatic function abnormal	3 (1.0)	1 (0.3)	0	0
Hiccups	3 (1.0)	0	0	0

Hyperhidrosis	3 (1.0)	0	1 (0.7)	0
Hypersensitivity	3 (1.0)	1 (0.3)	0	0
Hypophosphatemia	3 (1.0)	1 (0.3)	2 (1.3)	0
Memory impairment	3 (1.0)	0	2 (1.3)	0
Musculoskeletal pain	3 (1.0)	0	1 (0.7)	0
Myocardial infarction	3 (1.0)	3 (1.0)	0	0
Neck pain	3 (1.0)	0	0	0
Pelvic pain	3 (1.0)	0	0	0
Pericarditis	3 (1.0)	0	0	0
Pleural effusion	3 (1.0)	2 (0.6)	1 (0.7)	0
Pleurisy	3 (1.0)	0	1 (0.7)	0
Renal failure	3 (1.0)	2 (0.6)	2 (1.3)	1 (0.7)
Tinnitus	3 (1.0)	0	1 (0.7)	0
Toothache	3 (1.0)	0	1 (0.7)	0
Ventricular extrasystoles	3 (1.0)	0	0	0
Vision blurred	3 (1.0)	0	0	0
Activated partial thromboplastin time prolonged	2 (0.6)	0	0	0
Acute respiratory distress syndrome	2 (0.6)	2 (0.6)	0	0
Anxiety	2 (0.6)	0	0	0
Arthritis	2 (0.6)	0	0	0
Asthma	2 (0.6)	0	2 (1.3)	1 (0.7)
Atrial flutter	2 (0.6)	1 (0.3)	0	0
Balance disorder	2 (0.6)	0	0	0
Bronchospasm	2 (0.6)	1 (0.3)	0	0

Bundle branch block left	2 (0.6)	0	0	0
Cardiac failure	2 (0.6)	1 (0.3)	2 (1.3)	2 (1.3)
Chest discomfort	2 (0.6)	0	1 (0.7)	0
Chest pain	2 (0.6)	0	0	0
Chronic gastritis	2 (0.6)	0	2 (1.3)	0
Coordination abnormal	2 (0.6)	0	1 (0.7)	0
Depression	2 (0.6)	0	0	0
Dermatitis	2 (0.6)	0	1 (0.7)	0
Diabetes mellitus	2 (0.6)	0	0	0
Dry mouth	2 (0.6)	0	1 (0.7)	0
Edema	2 (0.6)	0	0	0
Epistaxis	2 (0.6)	0	1 (0.7)	0
Gamma-glutamyl transferase increased	2 (0.6)	1 (0.3)	3 (2.0)	1 (0.7)
Gastroenteritis	2 (0.6)	0	0	0
General physical health deterioration	2 (0.6)	1 (0.3)	0	0
Gynecomastia	2 (0.6)	0	0	0
Hematocrit decreased	2 (0.6)	0	0	0
Hematuria	2 (0.6)	0	1 (0.7)	0
Hemoglobin decreased	2 (0.6)	0	0	0
Hyperamylasemia	2 (0.6)	0	0	0
Hypermagnesemia	2 (0.6)	0	0	0
Hypophagia	2 (0.6)	1 (0.3)	0	0
Laryngeal hemorrhage	2 (0.6)	0	0	0
Lung abscess	2 (0.6)	1 (0.3)	0	0
Mucosal inflammation	2 (0.6)	0	0	0

Myelosuppression	2 (0.6)	2 (0.6)	0	0
Neutrophil count increased	2 (0.6)	0	0	0
Pain	2 (0.6)	1 (0.3)	0	0
Palpitations	2 (0.6)	0	0	0
Pancreatic enzymes increased	2 (0.6)	1 (0.3)	0	0
Peripheral sensorimotor neuropathy	2 (0.6)	0	0	0
Psoriasis	2 (0.6)	1 (0.3)	0	0
Rash papular	2 (0.6)	0	1 (0.7)	0
Renal impairment	2 (0.6)	0	0	0
Rhinitis atrophic	2 (0.6)	0	0	0
Seizure	2 (0.6)	2 (0.6)	1 (0.7)	0
Sleep disorder	2 (0.6)	0	0	0
Sudden death	2 (0.6)	2 (0.6)	0	0
Syncope	2 (0.6)	1 (0.3)	0	0
Thyroxine free increased	2 (0.6)	0	0	0
Thyroxine increased	2 (0.6)	0	0	0
Transaminases increased	2 (0.6)	1 (0.3)	0	0
Tremor	2 (0.6)	0	0	0
Tubulointerstitial nephritis	2 (0.6)	0	0	0
Type 2 diabetes mellitus	2 (0.6)	0	0	0
Urticaria	2 (0.6)	0	0	0
Viral infection	2 (0.6)	0	0	0
Xerophthalmia	2 (0.6)	0	0	0
Acute sinusitis	1 (0.3)	0	0	0

Alanine aminotransferase abnormal	1 (0.3)	0	0	0
Alanine aminotransferase decreased	1 (0.3)	0	0	0
Anesthesia	1 (0.3)	0	0	0
Angina pectoris	1 (0.3)	0	0	0
Arachnoid cyst	1 (0.3)	0	0	0
Arterial occlusive disease	1 (0.3)	0	0	0
Arteriosclerosis	1 (0.3)	0	1 (0.7)	1 (0.7)
Arthropod sting	1 (0.3)	0	0	0
Ascites	1 (0.3)	0	1 (0.7)	1 (0.7)
Ataxia	1 (0.3)	0	1 (0.7)	0
Autoimmune arthritis	1 (0.3)	0	0	0
Autoimmune thyroiditis	1 (0.3)	0	0	0
Basedow’s disease	1 (0.3)	0	1 (0.7)	0
Benign prostatic hyperplasia	1 (0.3)	0	0	0
Blood albumin decreased	1 (0.3)	0	1 (0.7)	0
Blood calcium decreased	1 (0.3)	0	1 (0.7)	0
Blood chloride decreased	1 (0.3)	0	0	0
Blood corticotrophin decreased	1 (0.3)	0	0	0
Blood corticotrophin increased	1 (0.3)	1 (0.3)	0	0
Blood glucose increased	1 (0.3)	0	0	0
Blood lactic acid increased	1 (0.3)	0	0	0
Blood potassium decreased	1 (0.3)	0	0	0
Blood sodium decreased	1 (0.3)	1 (0.3)	0	0

Body temperature decreased	1 (0.3)	0	0	0
Body temperature increased	1 (0.3)	0	2 (1.3)	0
Body tinea	1 (0.3)	0	0	0
Brachial plexopathy	1 (0.3)	0	0	0
Bronchiolitis	1 (0.3)	0	0	0
Bronchopleural fistula	1 (0.3)	0	0	0
Bundle branch block right	1 (0.3)	0	0	0
Burning sensation	1 (0.3)	0	0	0
Cardiac failure chronic	1 (0.3)	0	0	0
Cellulitis	1 (0.3)	0	0	0
Cerebrovascular accident	1 (0.3)	0	0	0
Chills	1 (0.3)	0	1 (0.7)	0
Chronic obstructive pulmonary disease	1 (0.3)	0	2 (1.3)	0
Clavicle fracture	1 (0.3)	0	0	0
Coagulopathy	1 (0.3)	0	1 (0.7)	0
Conduction disorder	1 (0.3)	0	0	0
Coronary artery disease	1 (0.3)	0	0	0
Cystatin C increased	1 (0.3)	0	0	0
Cystitis	1 (0.3)	0	0	0
Deafness neurosensory	1 (0.3)	0	0	0
Dermatitis allergic	1 (0.3)	0	0	0
Dizziness postural	1 (0.3)	0	0	0
Drug hypersensitivity	1 (0.3)	0	3 (2.0)	0
Drug-induced liver injury	1 (0.3)	1 (0.3)	0	0

Dry eye	1 (0.3)	0	1 (0.7)	0
Dysarthria	1 (0.3)	0	0	0
Dysgeusia	1 (0.3)	0	0	0
Dyslexia	1 (0.3)	0	0	0
Dyslipidemia	1 (0.3)	0	0	0
Dyspnea exertional	1 (0.3)	0	0	0
Electrocardiogram QT prolonged	1 (0.3)	0	0	0
Embolism	1 (0.3)	1 (0.3)	1 (0.7)	1 (0.7)
Eosinophilia	1 (0.3)	0	0	0
Eructation	1 (0.3)	0	0	0
Erythema	1 (0.3)	0	1 (0.7)	0
Erythropenia	1 (0.3)	0	0	0
Exostosis	1 (0.3)	0	0	0
Face edema	1 (0.3)	0	0	0
Femur fracture	1 (0.3)	0	0	0
Fibroma	1 (0.3)	0	0	0
Fluid overload	1 (0.3)	0	0	0
Flushing	1 (0.3)	0	0	0
Food poisoning	1 (0.3)	0	0	0
Gallbladder polyp	1 (0.3)	0	0	0
Gastrointestinal hemorrhage	1 (0.3)	1 (0.3)	0	0
Gastroesophageal reflux disease	1 (0.3)	0	1 (0.7)	0
Gingival bleeding	1 (0.3)	0	0	0
Gliosis	1 (0.3)	0	0	0

Glossitis	1 (0.3)	0	0	0
Glossodynia	1 (0.3)	0	0	0
Glucose tolerance impaired	1 (0.3)	0	0	0
Glycolic acid increased	1 (0.3)	0	0	0
Gout	1 (0.3)	1 (0.3)	0	0
Hematoma	1 (0.3)	0	1 (0.7)	0
Hemorrhoidal hemorrhage	1 (0.3)	0	0	0
Hemorrhoids	1 (0.3)	0	0	0
Hand dermatitis	1 (0.3)	0	0	0
Hemiparesis	1 (0.3)	0	0	0
Hepatic enzyme increased	1 (0.3)	0	0	0
Hepatic hemorrhage	1 (0.3)	0	0	0
Hepatitis	1 (0.3)	0	1 (0.7)	0
Hepatitis B	1 (0.3)	0	0	0
Hepatitis acute	1 (0.3)	1 (0.3)	0	0
Hepatotoxicity	1 (0.3)	0	1 (0.7)	0
Herpes zoster	1 (0.3)	1 (0.3)	1 (0.7)	0
Hot flush	1 (0.3)	0	0	0
Hyperemia	1 (0.3)	0	0	0
Hyperlipidemia	1 (0.3)	0	0	0
Hypernatremia	1 (0.3)	1 (0.3)	0	0
Hyperphosphatemia	1 (0.3)	0	0	0
Hypertriglyceridemia	1 (0.3)	0	0	0
Hypochloremia	1 (0.3)	0	0	0
Hypoglycemia	1 (0.3)	0	0	0

Hypoxia	1 (0.3)	1 (0.3)	0	0
Iliac artery stenosis	1 (0.3)	0	0	0
Immune-mediated hepatitis	1 (0.3)	0	0	0
Immune-mediated nephritis	1 (0.3)	0	0	0
Immune-mediated pneumonitis	1 (0.3)	1 (0.3)	0	0
Immune-mediated thyroiditis	1 (0.3)	0	0	0
Infection	1 (0.3)	1 (0.3)	0	0
Infectious pleural effusion	1 (0.3)	1 (0.3)	0	0
Influenza	1 (0.3)	0	0	0
Influenza-like illness	1 (0.3)	0	0	0
Infusion-related reaction	1 (0.3)	0	1 (0.7)	0
Iron deficiency anemia	1 (0.3)	0	0	0
Ischemic cerebral infarction	1 (0.3)	1 (0.3)	0	0
Jaundice cholestatic	1 (0.3)	0	1 (0.7)	1 (0.7)
Lethargy	1 (0.3)	0	0	0
Libido decreased	1 (0.3)	0	0	0
Lichen planus	1 (0.3)	0	0	0
Liver function test abnormal	1 (0.3)	0	0	0
Loss of consciousness	1 (0.3)	0	0	0
Lung disorder	1 (0.3)	0	0	0
Lung infiltration	1 (0.3)	0	0	0
Lung opacity	1 (0.3)	0	0	0
Lymphocytosis	1 (0.3)	1 (0.3)	0	0
Lymphopenia	1 (0.3)	0	1 (0.7)	1 (0.7)
Malaise	1 (0.3)	0	1 (0.7)	0

Melena	1 (0.3)	1 (0.3)	0	0
Mesenteric artery thrombosis	1 (0.3)	1 (0.3)	0	0
Metastases to central nervous system	1 (0.3)	1 (0.3)	0	0
Mouth swelling	1 (0.3)	0	0	0
Mouth ulceration	1 (0.3)	0	0	0
Mucosal infection	1 (0.3)	0	0	0
Multiple injuries	1 (0.3)	0	0	0
Musculoskeletal chest pain	1 (0.3)	0	4 (2.6)	0
Myocardial ischemia	1 (0.3)	0	0	0
Nasal dryness	1 (0.3)	0	0	0
Nasopharyngitis	1 (0.3)	0	3 (2.0)	0
Neuralgia	1 (0.3)	0	0	0
Neutropenic sepsis	1 (0.3)	1 (0.3)	0	0
Neutrophilia	1 (0.3)	0	1 (0.7)	0
Obesity	1 (0.3)	0	0	0
Obstructive pancreatitis	1 (0.3)	0	0	0
Oliguria	1 (0.3)	1 (0.3)	0	0
Oral herpes	1 (0.3)	0	2 (1.3)	0
Oral pain	1 (0.3)	1 (0.3)	0	0
Oropharyngeal pain	1 (0.3)	0	0	0
Orthostatic hypotension	1 (0.3)	0	0	0
Osteomyelitis	1 (0.3)	0	0	0
Pain in jaw	1 (0.3)	0	0	0
Pancytopenia	1 (0.3)	1 (0.3)	1 (0.7)	1 (0.7)
Paracancerous pneumonia	1 (0.3)	0	0	0

Partial seizures	1 (0.3)	1 (0.3)	0	0
Pathological fracture	1 (0.3)	0	0	0
Pericardial effusion	1 (0.3)	0	0	0
Periorbital edema	1 (0.3)	0	0	0
Peripheral arterial occlusive disease	1 (0.3)	0	0	0
Pharyngeal inflammation	1 (0.3)	0	0	0
Physical deconditioning	1 (0.3)	1 (0.3)	0	0
Pneumonia aspiration	1 (0.3)	1 (0.3)	0	0
Pneumonia viral	1 (0.3)	1 (0.3)	1 (0.7)	0
Polyneuropathy in malignant disease	1 (0.3)	0	0	0
Poor quality sleep	1 (0.3)	0	0	0
Prealbumin increased	1 (0.3)	0	0	0
Proctitis	1 (0.3)	0	0	0
Protein urine present	1 (0.3)	0	0	0
Prothrombin time prolonged	1 (0.3)	0	0	0
Pulmonary hemorrhage	1 (0.3)	1 (0.3)	0	0
Pulmonary edema	1 (0.3)	1 (0.3)	0	0
Pulmonary vascular disorder	1 (0.3)	0	0	0
Rash macular	1 (0.3)	0	0	0
Rash pruritic	1 (0.3)	0	0	0
Respiratory failure	1 (0.3)	1 (0.3)	3 (2.0)	2 (1.3)
Respiratory tract infection viral	1 (0.3)	1 (0.3)	2 (1.3)	0
Rhinitis allergic	1 (0.3)	0	0	0
Road traffic accident	1 (0.3)	1 (0.3)	0	0

Salivary hypersecretion	1 (0.3)	0	0	0
Scleritis	1 (0.3)	0	0	0
Septic shock	1 (0.3)	1 (0.3)	0	0
Sialic acid increased	1 (0.3)	0	0	0
Sinusitis	1 (0.3)	0	0	0
Skin exfoliation	1 (0.3)	0	1 (0.7)	0
Skin fissures	1 (0.3)	0	0	0
Skin hyperpigmentation	1 (0.3)	0	0	0
Skin reaction	1 (0.3)	0	0	0
Skin toxicity	1 (0.3)	0	0	0
Soft tissue infection	1 (0.3)	0	0	0
Somnolence	1 (0.3)	0	1 (0.7)	0
Spinal pain	1 (0.3)	0	1 (0.7)	0
Superior vena cava occlusion	1 (0.3)	1 (0.3)	0	0
Taste disorder	1 (0.3)	0	1 (0.7)	0
Thrombophlebitis	1 (0.3)	0	0	0
Thrombophlebitis superficial	1 (0.3)	0	0	0
Thrombosis mesenteric vessel	1 (0.3)	1 (0.3)	0	0
Thyroxine free decreased	1 (0.3)	0	0	0
Tinea infection	1 (0.3)	0	0	0
Toxic neuropathy	1 (0.3)	0	0	0
Toxic nodular goiter	1 (0.3)	0	0	0
Transplant rejection	1 (0.3)	0	0	0
Tri-iodothyronine free increased	1 (0.3)	0	0	0

Vascular encephalopathy	1 (0.3)	0	0	0
Venous thrombosis	1 (0.3)	0	0	0
Visual impairment	1 (0.3)	0	0	0
White matter lesion	1 (0.3)	0	0	0
Abdominal wall wound	0	0	1 (0.7)	0
Acute coronary syndrome	0	0	1 (0.7)	1 (0.7)
Ageusia	0	0	1 (0.7)	0
Angioedema	0	0	1 (0.7)	0
Back disorder	0	0	1 (0.7)	0
Bacteriuria	0	0	1 (0.7)	0
Blood bicarbonate decreased	0	0	1 (0.7)	0
Blood creatinine decreased	0	0	1 (0.7)	0
Blood iron decreased	0	0	1 (0.7)	0
Bursitis	0	0	1 (0.7)	0
Cardiac failure acute	0	0	1 (0.7)	1 (0.7)
Cardiomyopathy	0	0	1 (0.7)	1 (0.7)
Cardiopulmonary failure	0	0	1 (0.7)	1 (0.7)
Carotid artery thrombosis	0	0	1 (0.7)	0
Cataract	0	0	1 (0.7)	0
Creatinine renal clearance decreased	0	0	1 (0.7)	0
Daydreaming	0	0	1 (0.7)	0
Deep vein thrombosis	0	0	1 (0.7)	0
Dermatitis acneiform	0	0	2 (1.3)	0
Diplopia	0	0	1 (0.7)	0
Dysuria	0	0	1 (0.7)	0

Ear infection	0	0	1 (0.7)	0
Eating disorder	0	0	1 (0.7)	0
Empyema	0	0	1 (0.7)	0
Enterocolitis	0	0	1 (0.7)	1 (0.7)
Epigastric discomfort	0	0	1 (0.7)	0
Essential hypertension	0	0	1 (0.7)	0
Food aversion	0	0	1 (0.7)	0
Functional gastrointestinal disorder	0	0	1 (0.7)	0
Gastritis erosive	0	0	1 (0.7)	0
Gastrointestinal infection	0	0	1 (0.7)	0
Gastrointestinal inflammation	0	0	1 (0.7)	0
Glaucoma	0	0	1 (0.7)	0
Granulocytopenia	0	0	1 (0.7)	1 (0.7)
Hematotoxicity	0	0	1 (0.7)	0
Hepatitis C	0	0	1 (0.7)	0
Herpes simplex	0	0	1 (0.7)	0
Hydrothorax	0	0	1 (0.7)	0
Hypercoagulation	0	0	1 (0.7)	0
Hyperthermia	0	0	1 (0.7)	0
Intestinal perforation	0	0	1 (0.7)	1 (0.7)
Ischemic stroke	0	0	1 (0.7)	1 (0.7)
Limb discomfort	0	0	1 (0.7)	0
Lower respiratory tract infection	0	0	1 (0.7)	1 (0.7)
Neurotoxicity	0	0	2 (1.3)	0

Onychomadesis	0	0	1 (0.7)	0
Osteonecrosis	0	0	1 (0.7)	1 (0.7)
Otitis externa	0	0	1 (0.7)	0
Peripheral nerve injury	0	0	1 (0.7)	0
Platelet count increased	0	0	1 (0.7)	0
Pleural fistula	0	0	1 (0.7)	0
Pneumonia serratia	0	0	1 (0.7)	0
Prostate cancer	0	0	1 (0.7)	1 (0.7)
Prostatitis	0	0	1 (0.7)	1 (0.7)
Protein total decreased	0	0	1 (0.7)	0
Reticulocyte count increased	0	0	1 (0.7)	0
Rhinitis	0	0	1 (0.7)	0
Rib fracture	0	0	1 (0.7)	0
Skin induration	0	0	1 (0.7)	1 (0.7)
Spinal osteoarthritis	0	0	1 (0.7)	0
Swelling face	0	0	1 (0.7)	0
Thyroxine decreased	0	0	1 (0.7)	0
Tumor associated fever	0	0	1 (0.7)	0
Urinary tract discomfort	0	0	1 (0.7)	0
Vitamin D deficiency	0	0	1 (0.7)	0
Xeroderma	0	0	1 (0.7)	0

The events are listed in descending order of frequency in the cemiplimab plus chemotherapy arm. The events were coded according to the Preferred Terms of the Medical Dictionary for Regulatory Activities, version 22.1. The severity of adverse events was graded according to the National Cancer Institute Common Terminology Criteria for Adverse Events, version 4.03.

COVID-19, coronavirus disease 19.

Supplementary Table 4. Treatment-related adverse events in the safety population.

The safety population includes all randomized patients who received at least one dose of any study drug. Events were coded according to the Preferred Terms of the MedDRA version 22.1. Severity of adverse events was graded according to the National Cancer Institute Common Terminology Criteria for Adverse Events, version 4.03.

	**Cemiplimab + chemotherapy (n=312)**		**Placebo + chemotherapy (n=153)**	
**Event, n (%)**	**Any grade**	**Grade ≥3**	**Any grade**	**Grade ≥3**
Any	275 (88.1)	90 (28.8)	129 (84.3)	28 (18.3)
Led to discontinuation	10 (3.2)	7 (2.2)	1 (0.7)	1 (0.7)
Led to death	4 (1.3)	4 (1.3)	1 (0.7)	1 (0.7)
Anemia	127 (40.7)	30 (9.6)	52 (34.0)	10 (6.5)
Alopecia	114 (36.5)	0	65 (42.5)	0
Nausea	71 (22.8)	0	25 (16.3)	0
Alanine aminotransferase increased	45 (14.4)	6 (1.9)	19 (12.4)	1 (0.7)
Neutropenia	45 (14.4)	17 (5.4)	19 (12.4)	9 (5.9)
Decreased appetite	42 (13.5)	1 (0.3)	16 (10.5)	0
Aspartate aminotransferase increased	39 (12.5)	1 (0.3)	15 (9.8)	1 (0.7)
Thrombocytopenia	39 (12.5)	7 (2.2)	19 (12.4)	1 (0.7)
Hyperglycemia	33 (10.6)	2 (0.6)	12 (7.8)	0
Vomiting	33 (10.6)	0	14 (9.2)	0

Arthralgia	29 (9.3)	1 (0.3)	11 (7.2)	0
Asthenia	28 (9.0)	2 (0.6)	10 (6.5)	1 (0.7)
Constipation	28 (9.0)	0	12 (7.8)	0
Peripheral sensory neuropathy	28 (9.0)	0	15 (9.8)	0
Blood creatinine increased	27 (8.7)	0	7 (4.6)	0
Insomnia	27 (8.7)	0	8 (5.2)	0
Diarrhea	26 (8.3)	3 (1.0)	4 (2.6)	0
Fatigue	26 (8.3)	3 (1.0)	9 (5.9)	1 (0.7)
Hypothyroidism	24 (7.7)	1 (0.3)	3 (2.0)	0
White blood cell count decreased	23 (7.4)	10 (3.2)	5 (3.3)	2 (1.3)
Blood urea increased	21 (6.7)	0	6 (3.9)	0
Amylase increased	18 (5.8)	1 (0.3)	4 (2.6)	0
Blood lactate dehydrogenase increased	18 (5.8)	0	5 (3.3)	0
Hypoalbuminemia	18 (5.8)	0	4 (2.6)	0
Leukopenia	18 (5.8)	3 (1.0)	10 (6.5)	2 (1.3)
Hypokalemia	17 (5.4)	3 (1.0)	4 (2.6)	1 (0.7)
Neuropathy peripheral	17 (5.4)	0	6 (3.9)	0
Hyperthyroidism	16 (5.1)	0	2 (1.3)	0
Platelet count decreased	16 (5.1)	3 (1.0)	6 (3.9)	0
Rash	16 (5.1)	1 (0.3)	4 (2.6)	0
Weight decreased	16 (5.1)	0	6 (3.9)	0
Paresthesia	15 (4.8)	0	3 (2.0)	0
Blood alkaline phosphatase increased	14 (4.5)	0	10 (6.5)	0
Blood uric acid increased	14 (4.5)	0	6 (3.9)	1 (0.7)

Weight increased	14 (4.5)	0	0	0
Blood thyroid-stimulating hormone increased	13 (4.2)	0	1 (0.7)	0
Hyperkalemia	13 (4.2)	0	1 (0.7)	0
Hypomagnesemia	12 (3.8)	0	0	0
Neutrophil count decreased	12 (3.8)	8 (2.6)	5 (3.3)	3 (2.0)
Pneumonitis	12 (3.8)	1 (0.3)	0	0
Hyperuricemia	11 (3.5)	0	3 (2.0)	0
Lipase increased	11 (3.5)	0	2 (1.3)	0
Lymphocyte count decreased	11 (3.5)	3 (1.0)	4 (2.6)	0
Pruritus	11 (3.5)	0	2 (1.3)	0
Mood altered	10 (3.2)	0	1 (0.7)	0
Stomatitis	10 (3.2)	2 (0.6)	1 (0.7)	0
Myalgia	9 (2.9)	0	1 (0.7)	0
Dyspnea	8 (2.6)	1 (0.3)	0	0
Pain in extremity	8 (2.6)	0	8 (5.2)	0
Bone pain	7 (2.2)	1 (0.3)	2 (1.3)	0
Hypocalcemia	6 (1.9)	0	4 (2.6)	0
Blood bilirubin increased	5 (1.6)	1 (0.3)	2 (1.3)	0
Blood thyroid-stimulating hormone decreased	5 (1.6)	0	2 (1.3)	0
Hypercalcemia	5 (1.6)	0	1 (0.7)	0
Lacrimation increased	5 (1.6)	0	1 (0.7)	0
Polyneuropathy	5 (1.6)	0	4 (2.6)	0
Rash maculopapular	5 (1.6)	1 (0.3)	0	0
Acute kidney injury	4 (1.3)	0	0	0

Febrile neutropenia	4 (1.3)	4 (1.3)	4 (2.6)	4 (2.6)
Gastritis	4 (1.3)	0	0	0
Hyperbilirubinemia	4 (1.3)	0	0	0
Hyponatremia	4 (1.3)	3 (1.0)	2 (1.3)	2 (1.3)
Edema peripheral	4 (1.3)	0	0	0
Pyrexia	4 (1.3)	0	4 (2.6)	0
Thrombocytosis	4 (1.3)	0	1 (0.7)	0
Abdominal distension	3 (1.0)	0	0	0
Bilirubin conjugated increased	3 (1.0)	0	0	0
Blood potassium increased	3 (1.0)	1 (0.3)	0	0
Colitis	3 (1.0)	1 (0.3)	0	0
Conjunctivitis	3 (1.0)	0	0	0
Dry skin	3 (1.0)	0	4 (2.6)	0
Hypersensitivity	3 (1.0)	1 (0.3)	0	0
Hypoesthesia	3 (1.0)	0	0	0
Hypophosphatemia	3 (1.0)	1 (0.3)	1 (0.7)	0
Leukocytosis	3 (1.0)	0	0	0
Muscular weakness	3 (1.0)	1 (0.3)	0	0
Noncardiac chest pain	3 (1.0)	0	2 (1.3)	0
Tachycardia	3 (1.0)	0	1 (0.7)	0
Tinnitus	3 (1.0)	0	0	0
Abdominal pain upper	2 (0.6)	0	0	0
Arthritis	2 (0.6)	0	0	0
Chronic kidney disease	2 (0.6)	1 (0.3)	2 (1.3)	1 (0.7)
Cough	2 (0.6)	0	1 (0.7)	0

Dermatitis	2 (0.6)	0	0	0
Dry mouth	2 (0.6)	0	0	0
Dyspepsia	2 (0.6)	0	2 (1.3)	0
Gamma-glutamyl transferase increased	2 (0.6)	1 (0.3)	2 (1.3)	0
Hematocrit decreased	2 (0.6)	0	0	0
Hemoglobin decreased	2 (0.6)	0	0	0
Hemoptysis	2 (0.6)	1 (0.3)	0	0
Hepatic function abnormal	2 (0.6)	1 (0.3)	0	0
Hiccups	2 (0.6)	0	0	0
Hyperamylasemia	2 (0.6)	0	0	0
Hypermagnesemia	2 (0.6)	0	0	0
Hypophagia	2 (0.6)	1 (0.3)	0	0
Lung abscess	2 (0.6)	1 (0.3)	0	0
Memory impairment	2 (0.6)	0	1 (0.7)	0
Mucosal inflammation	2 (0.6)	0	0	0
Musculoskeletal pain	2 (0.6)	0	1 (0.7)	0
Myelosuppression	2 (0.6)	2 (0.6)	0	0
Pain	2 (0.6)	1 (0.3)	0	0
Pericarditis	2 (0.6)	0	0	0
Peripheral sensorimotor neuropathy	2 (0.6)	0	0	0
Peripheral swelling	2 (0.6)	0	1 (0.7)	0
Pneumonia	2 (0.6)	0	2 (1.3)	1 (0.7)
Psoriasis	2 (0.6)	1 (0.3)	0	0
Rash papular	2 (0.6)	0	1 (0.7)	0
Renal impairment	2 (0.6)	0	0	0

Sinus tachycardia	2 (0.6)	0	0	0
Thyroxine free increased	2 (0.6)	0	0	0
Thyroxine increased	2 (0.6)	0	0	0
Transaminases increased	2 (0.6)	1 (0.3)	0	0
Tremor	2 (0.6)	0	0	0
Urticaria	2 (0.6)	0	0	0
Abdominal pain	1 (0.3)	0	1 (0.7)	0
Activated partial thromboplastin time prolonged	1 (0.3)	0	0	0
Acute sinusitis	1 (0.3)	0	0	0
Alanine aminotransferase abnormal	1 (0.3)	0	0	0
Alanine aminotransferase decreased	1 (0.3)	0	0	0
Anxiety	1 (0.3)	0	0	0
Ascites	1 (0.3)	0	0	0
Asthma	1 (0.3)	0	0	0
Atrial flutter	1 (0.3)	1 (0.3)	0	0
Autoimmune arthritis	1 (0.3)	0	0	0
Autoimmune thyroiditis	1 (0.3)	0	0	0
Back pain	1 (0.3)	0	4 (2.6)	0
Blood calcium decreased	1 (0.3)	0	0	0
Blood chloride decreased	1 (0.3)	0	0	0
Blood corticotrophin decreased	1 (0.3)	0	0	0
Blood corticotrophin increased	1 (0.3)	1 (0.3)	0	0
Blood potassium decreased	1 (0.3)	0	0	0

Blood pressure increased	1 (0.3)	0	0	0
Blood sodium decreased	1 (0.3)	1 (0.3)	0	0
Bronchiolitis	1 (0.3)	0	0	0
Bronchitis chronic	1 (0.3)	1 (0.3)	0	0
Bronchospasm	1 (0.3)	0	0	0
Burning sensation	1 (0.3)	0	0	0
Cystatin C increased	1 (0.3)	0	0	0
Deafness neurosensory	1 (0.3)	0	0	0
Death	1 (0.3)	1 (0.3)	0	0
Depression	1 (0.3)	0	0	0
Dermatitis allergic	1 (0.3)	0	0	0
Diabetes mellitus	1 (0.3)	0	0	0
Dizziness	1 (0.3)	0	0	0
Drug-induced liver injury	1 (0.3)	1 (0.3)	0	0
Dry eye	1 (0.3)	0	1 (0.7)	0
Dyslexia	1 (0.3)	0	0	0
Dyslipidemia	1 (0.3)	0	0	0
Dysphagia	1 (0.3)	0	0	0
Dysphonia	1 (0.3)	0	0	0
Electrocardiogram QT prolonged	1 (0.3)	0	0	0
Embolism	1 (0.3)	1 (0.3)	0	0
Eosinophilia	1 (0.3)	0	0	0
Epistaxis	1 (0.3)	0	1 (0.7)	0
Eructation	1 (0.3)	0	0	0
Erythema	1 (0.3)	0	1 (0.7)	0

Erythropenia	1 (0.3)	0	0	0
Fluid overload	1 (0.3)	0	0	0
Flushing	1 (0.3)	0	0	0
Gastroenteritis	1 (0.3)	0	0	0
Gastroesophageal reflux disease	1 (0.3)	0	0	0
General physical health deterioration	1 (0.3)	1 (0.3)	0	0
Glossitis	1 (0.3)	0	0	0
Glossodynia	1 (0.3)	0	0	0
Glycolic acid increased	1 (0.3)	0	0	0
Hematuria	1 (0.3)	0	1 (0.7)	0
Hand dermatitis	1 (0.3)	0	0	0
Hepatic hemorrhage	1 (0.3)	0	0	0
Hepatitis	1 (0.3)	0	1 (0.7)	0
Hepatitis B	1 (0.3)	0	0	0
Hepatotoxicity	1 (0.3)	0	1 (0.7)	0
Hyperemia	1 (0.3)	0	0	0
Hyperhidrosis	1 (0.3)	0	1 (0.7)	0
Hypernatremia	1 (0.3)	1 (0.3)	0	0
Hypertension	1 (0.3)	0	2 (1.3)	0
Hypochloremia	1 (0.3)	0	0	0
Hypoglycemia	1 (0.3)	0	0	0
Hypoproteinemia	1 (0.3)	0	1 (0.7)	0
Hypotension	1 (0.3)	0	0	0
Iliac artery stenosis	1 (0.3)	0	0	0
Immune-mediated hepatitis	1 (0.3)	0	0	0

Immune-mediated nephritis	1 (0.3)	0	0	0
Immune-mediated pneumonitis	1 (0.3)	1 (0.3)	0	0
Immune-mediated thyroiditis	1 (0.3)	0	0	0
Infection	1 (0.3)	1 (0.3)	0	0
Infusion-related reaction	1 (0.3)	0	1 (0.7)	0
Lethargy	1 (0.3)	0	0	0
Lichen planus	1 (0.3)	0	0	0
Liver function test abnormal	1 (0.3)	0	0	0
Lung infiltration	1 (0.3)	0	0	0
Lymphocytosis	1 (0.3)	1 (0.3)	0	0
Lymphopenia	1 (0.3)	0	1 (0.7)	1 (0.7)
Malaise	1 (0.3)	0	1 (0.7)	0
Mesenteric artery thrombosis	1 (0.3)	1 (0.3)	0	0
Mouth swelling	1 (0.3)	0	0	0
Mouth ulceration	1 (0.3)	0	0	0
Mucosal infection	1 (0.3)	0	0	0
Myocardial infarction	1 (0.3)	1 (0.3)	0	0
Nasal dryness	1 (0.3)	0	0	0
Neuralgia	1 (0.3)	0	0	0
Neutropenic sepsis	1 (0.3)	1 (0.3)	0	0
Neutrophil count increased	1 (0.3)	0	0	0
Obesity	1 (0.3)	0	0	0
Oral pain	1 (0.3)	1 (0.3)	0	0
Palpitations	1 (0.3)	0	0	0

Pancreatic enzymes increased	1 (0.3)	1 (0.3)	0	0
Pancytopenia	1 (0.3)	1 (0.3)	1 (0.7)	1 (0.7)
Pericardial effusion	1 (0.3)	0	0	0
Periorbital edema	1 (0.3)	0	0	0
Pharyngeal inflammation	1 (0.3)	0	0	0
Pleurisy	1 (0.3)	0	0	0
Poor quality sleep	1 (0.3)	0	0	0
Prealbumin increased	1 (0.3)	0	0	0
Productive cough	1 (0.3)	0	1 (0.7)	0
Pulmonary embolism	1 (0.3)	1 (0.3)	0	0
Rash macular	1 (0.3)	0	0	0
Rash pruritic	1 (0.3)	0	0	0
Renal failure	1 (0.3)	0	1 (0.7)	1 (0.7)
Respiratory tract infection	1 (0.3)	0	0	0
Rhinitis atrophic	1 (0.3)	0	0	0
Salivary hypersecretion	1 (0.3)	0	0	0
Seizure	1 (0.3)	1 (0.3)	0	0
Skin fissures	1 (0.3)	0	0	0
Skin hyperpigmentation	1 (0.3)	0	0	0
Skin reaction	1 (0.3)	0	0	0
Skin toxicity	1 (0.3)	0	0	0
Sleep disorder	1 (0.3)	0	0	0
Somnolence	1 (0.3)	0	0	0
Taste disorder	1 (0.3)	0	1 (0.7)	0
Thrombophlebitis	1 (0.3)	0	0	0

Thrombophlebitis superficial	1 (0.3)	0	0	0
Thyroxine free decreased	1 (0.3)	0	0	0
Toxic neuropathy	1 (0.3)	0	0	0
Tri-iodothyronine free increased	1 (0.3)	0	0	0
Tubulointerstitial nephritis	1 (0.3)	0	0	0
Urinary tract infection	1 (0.3)	1 (0.3)	0	0
Xerophthalmia	1 (0.3)	0	0	0
Ageusia	0	0	1 (0.7)	0
Atrial fibrillation	0	0	1 (0.7)	1 (0.7)
Blood bicarbonate decreased	0	0	1 (0.7)	0
Blood creatinine decreased	0	0	1 (0.7)	0
Body temperature increased	0	0	1 (0.7)	0
Bronchitis	0	0	1 (0.7)	1 (0.7)
Chills	0	0	1 (0.7)	0
Coagulopathy	0	0	1 (0.7)	0
Coordination abnormal	0	0	1 (0.7)	0
Creatinine renal clearance decreased	0	0	1 (0.7)	0
Deep vein thrombosis	0	0	1 (0.7)	0
Dermatitis acneiform	0	0	2 (1.3)	0
Drug hypersensitivity	0	0	3 (2.0)	0
Dysuria	0	0	1 (0.7)	0
Enterocolitis	0	0	1 (0.7)	1 (0.7)
Food aversion	0	0	1 (0.7)	0

Glaucoma	0	0	1 (0.7)	0
Granulocytopenia	0	0	1 (0.7)	1 (0.7)
Hematotoxicity	0	0	1 (0.7)	0
Headache	0	0	1 (0.7)	0
Herpes simplex	0	0	1 (0.7)	0
Herpes zoster	0	0	1 (0.7)	0
Hyperthermia	0	0	1 (0.7)	0
Limb discomfort	0	0	1 (0.7)	0
Nasopharyngitis	0	0	1 (0.7)	0
Neurotoxicity	0	0	2 (1.3)	0
Onychomadesis	0	0	1 (0.7)	0
Oral herpes	0	0	1 (0.7)	0
Osteonecrosis	0	0	1 (0.7)	1 (0.7)
Pleural effusion	0	0	1 (0.7)	0
Pneumonia serratia	0	0	1 (0.7)	0
Protein total decreased	0	0	1 (0.7)	0
Respiratory failure	0	0	1 (0.7)	1 (0.7)
Respiratory tract infection viral	0	0	1 (0.7)	0
Spinal pain	0	0	1 (0.7)	0

The events are listed in descending order of frequency in the cemiplimab plus chemotherapy arm. The events were coded according to the Preferred Terms of the Medical Dictionary for Regulatory Activities, version 22.1. The severity of adverse events was graded according to the National Cancer Institute Common Terminology Criteria for Adverse Events, version 4.03.

Supplementary Table 5. Sponsor-identified immune-related adverse events.

The study sponsor used a customized query to identify potential immune-related adverse events (irAEs) among all patients who received at least one dose of cemiplimab plus chemotherapy treatment, according to Preferred Terms of the MedDRA version 22.1. Treatment-related TEAEs on this list were considered potential irAEs. Identified irAEs were defined as potential irAEs requiring treatment with systemic corticosteroids or immunosuppressants or were immune-related endocrinopathies. Severity of adverse events was graded according to the National Cancer Institute Common Terminology Criteria for Adverse Events, version 4.03.

	**Cemiplimab + chemotherapy (n=312)**	
**Event, n (%)**	**Any grade**	**Grade ≥3**
Any	59 (18.9)	9 (2.9)
Led to discontinuation	3 (1.0)	3 (1.0)
Led to death	1 (0.3)	1 (0.3)
Hypothyroidism	24 (7.7)	1 (0.3)
Hyperthyroidism	16 (5.1)	0
Blood thyroid-stimulating hormone increased	13 (4.2)	0
Pneumonitis	5 (1.6)	1 (0.3)
Alanine aminotransferase increased	2 (0.6)	2 (0.6)
Dermatitis	2 (0.6)	0
Pruritus	2 (0.6)	0
Rash	2 (0.6)	1 (0.3)
Aspartate aminotransferase increased	1 (0.3)	0

Autoimmune arthritis	1 (0.3)	0
Autoimmune thyroiditis	1 (0.3)	0
Blood alkaline phosphatase increased	1 (0.3)	0
Blood bilirubin increased	1 (0.3)	1 (0.3)
Blood creatinine increased	1 (0.3)	0
Colitis	1 (0.3)	1 (0.3)
Diabetes mellitus	1 (0.3)	0
Gamma-glutamyl transferase increased	1 (0.3)	1 (0.3)
Immune-mediated nephritis	1 (0.3)	0
Immune-mediated pneumonitis	1 (0.3)	1 (0.3)
Immune-mediated thyroiditis	1 (0.3)	0
Psoriasis	1 (0.3)	1 (0.3)
Rash maculopapular	1 (0.3)	1 (0.3)

As irAEs are associated with cemiplimab exposure, irAEs were not summarized in the placebo plus chemotherapy arm.

irAE, immune-related adverse event.

Supplementary Table 6. Investigator's choice of chemotherapy options.

Option	Chemotherapy	Dosing frequency	Maintenance therapy
1	Paclitaxel 200 mg/m^2 IV plus carboplatin; AUC of 5 or 6 mg/mL/minute IV	Day 1 every 21 days for 4 cycles; calculate dose of carboplatin using the Calvert formula	No maintenance therapy
2	Paclitaxel 200 mg/m^2 IV plus cisplatin 75 mg/m^2 IV	Day 1 every 21 days for 4 cycles	No maintenance therapy
3	Pemetrexed 500 mg/m^2 IV plus carboplatin; AUC of 5 or 6 mg/mL/minute IV	Day 1 every 21 days for 4 cycles; calculate dose of carboplatin using the Calvert formula	Mandatory pemetrexed maintenance 500 mg/m^2 IV day 1 every 21 days; pemetrexed maintenance according to local prescribing information and practice guidelines
4	Pemetrexed 500 mg/m^2 IV plus cisplatin 75 mg/m^2 IV	Day 1 every 21 days for 4 cycles	Mandatory pemetrexed maintenance 500 mg/m^2 IV day 1 every 21 days; pemetrexed maintenance according to local prescribing information and practice guidelines

AUC, area under the curve; IV, intravenous.

Supplementary Table 7. List of institutional review boards and independent ethics committees that approved the clinical trial protocol

Country	Study site no.	Local institutional review boards and independent ethics committees	Central institutional review boards and independent ethics committees
China	156001	Ethics Committee of Shanghai Pulmonary Hospital, No.507 Zhengmin Road, Yangpu District, Shanghai, 200433, China	
China	156002	Life Ethics Committee of Beijing Friendship Hospital, Capital Medical University, No.95 YongAn Road, Xicheng District, Beijing, 100050, China	
China	156003	Ethics Committee of Jinan Central Hospital, No.105 Jiefang Road, Jinan, Shandong, 250013, China	
China	156004	Ethics Committee of Affiliated Hospital of Hebei University, No. 648 Dongfeng East Road, Lianchi Qu, Baoding Shi, Hebei Sheng, 071105, China	
China	156005	The Ethics Committee of Cancer Hospital of Xinjiang Medical University, No.789, Suzhou East Street, New Urban Area, Urumqi, Xinjiang, 830000, China	
China	156006	Ethics Committee of Clinical Trials of the First Affiliated Hospital, College of Medicine, Zhejiang University, No.79 Qingchun Road, Hangzhou, Zhejiang, 310003, China	
China	156007	Ethics Committee of the Second Affiliated Hospital of Zhejiang University School of Medicine, No.88 Jiefang Road, Shangcheng District, Hangzhou, Zhejiang, 310009, China	
China	156008	Institutional Review Board of Huadong Hospital, No.168 Yan’an Road West, Jing’an District, Shanghai,200040, China	
China	156011	Clinical Trial Ethics Committee of the Henan Provincial Peoples Hospital, No.7, Weiwu Road, Zhengzhou, Henan, 450003, China	

China	156012	Ethics Committee of The Second Affiliated Hospital of Nanjing Medical University, No.121 Jiangjiayuan Road, Gulou district, Nanjing, Jiangsu, 210011, China	
China	156014	The First Affiliated Hospital of Guangzhou Medical University, No. 151, Yanjiang West Road, Yuexiu District, Guangzhou City, Guangdong Province, 510030, China	
China	156015	Ethics Committee of Xiangyang Central Hospital, No.136 Jinzhou Street, Xiangcheng, Xiangyang City, Hubei, 441021, China	
China	156018	Ethics Committee of the First Affiliated Hospital of Guangdong Pharmaceutical University, No. 19, Nonglinxia Road, Yuexiu District, Guangzhou, Guangdong, 510000, China	
China	156019	Ethics Committee of Henan Cancer Hospital, No.127 Dongming Road, Zhengzhou, Henan, 450008, China	
China	156022	Good Clinical Practice Office of Hanghou First Peoples Hospital, Hangzhou Cancer Hospital, No.34, Yanguan Xiang,Shangcheng district, Hangzhou City, Zhejiang Province, 310002, China	
China	156023	Anhui Provincial Cancer Hospital Clinical Trial Ethics Committee, No.107, Huanhu East Road, Shushan District, Hefei, Anhui, 230031, China	
China	156024	Ethics Committee of Fuzhou Pulmonary Hospital of Fujian, No.2 Hubian, Cangshan District, Fuzhou, Fujian, 350008, China	
China	156026	Medical Ethics Committee of Liaoning Cancer Hospital and Institute, No. 44, Xiaoheyan Road, Dadong District, Shenyang, Liaoning, 110042, China	
China	156028	Ethics Committee of Hunan Cancer Hospital, No.283, Tongzipo Road, Yuelu District, Changsha, Hunan, 410013, China	

China	156029	Ethics Committee of Linyi Cancer Hospital, Linyi Cancer Hospital, Intersection between Zhongsheng Street and Zhicheng Road, Hedong District, Linyi City, Shangdong Province, 276000, China	
China	156030	Ethics Review Committee of Zhejiang Hospital, No. 12, Lingyin Road, Xihu District, Hangzhou, Zhejiang Province, 310013, China	
Georgia	268001	Local Ethics Committee of High Technology Medical Center University Clinic, 9 Tsinandali Street, 0144 Tbilisi, Georgia	
Georgia	268002	Local Ethics Committee of Cancer Center of Adjara Autonomic Republic, 118 Pushkin Street, 6000 Batumi, Georgia	
Georgia	268003	Local Ethics Committee of JSC “Neo Medi”, 12 Kristine Sharashidze Street, 0159 Tbilisi, Georgia	
Georgia	268004	Local Ethics Committee of Institute of Clinical Oncology, 5, Lubliana Street, 0156 Tbilisi, Georgia	
Georgia	268005	Local Ethics Committee of Research Institute of Clinical Medicine, 13 Tevdore Mgvdeli Street, 0112 Tbilisi, Georgia	
Georgia	268006	Local Ethics Committee of “Multi-Profile Clinic Consilium Medulla”, 6g Politkovskaia Street, 0186 Tbilisi, Georgia	
Greece	300001<br>300003<br>300005<br>300006<br>300007<br>300008<br>300009<br>300010<br>300011<br>300012		Hellenic Republic, Ministry of Health, National Ethic Committee, 284 Mesogion Avenue, 155 62 Cholargos, Greece
Malaysia	458001<br>458003<br>458004<br>458005<br>458006<br>458007<br>458010		Medical Research & Ethics Committee c/o Kompleks Institut Kesihatan Negara Blok A, No 1, Jalan Setia Murni U13/52, Seksyen U13, Bandar Setia Alam, 40170 Shah Alam, Selangor, Malaysia

Malaysia	458008	Clinical Investigation Centre, 5th Floor, East Tower, University Malaya Medical Centre, 59100, Lembah Pantai, Kuala Lumpur, Malaysia	
Malaysia	458009	Research Ethics Committee, The National University of Malaysia, 1st Floor, Clinical Block, Hospital Canselor Tuanku Muhriz, Universiti Kebangsaan Malaysia Medical Centre, Jalan Yaacob Latif, Bandar Tun Razak, 56000, Cheras, Kuala Lumpur, Malaysia	
Poland	616001<br>616002<br>616003<br>616004<br>616006<br>616007<br>616008<br>616009<br>616010<br>616011		Komisja Bioetyczna przy Okręgowej Izbie Lekarskiej w Lublinie ul. Chmielna 4, 20-079 Lublin, Poland
Romania	642001<br>642002<br>642003<br>642004<br>642005<br>642006<br>642007<br>642008<br>642009		Romania Academy of Medical Sciences, National Bioethics Committee for Medicines and Medical Devices, Sos. Stefan cel Mare nr. 19-21, Sector 2, Bucharest, Romania
Russia	643001	Ethics Committee of State Budgetary Healthcare Institution of Sverdlovsk Region “Sverdlovsk Regional Oncology Dispensary”, 29, Soboleva Street, Yekaterinburg, 620036, Russian Federation	Ethics Council at the Ministry of Healthcare of the Russian Federation, Rakhmanovskiy pereulok, 3, Moscow, 127994, Russian Federation
Russia	643002	Ethics Committee at the Regional Budget Healthcare Institution “Kursk Regional Scientific Clinical Centra n.a. G. E. Ostroverkhov”, Ul. Eliseeva, 1, Kislino, Kursk Region, Kursk District, 305524, Russian Federation	Ethics Council at the Ministry of Healthcare of the Russian Federation, Rakhmanovskiy pereulok, 3, Moscow, 127994, Russian Federation
Russia	643003	Ethics Committee at City Clinical Oncologic Dispensary of Saint Petersburg, 3/5, 2-ya Berezovaya alleya, Saint Petersburg, Russian Federation / 56, Prospekt Veteranov, Saint Petersburg, 197022, Russian Federation	Ethics Council at the Ministry of Healthcare of the Russian Federation, Rakhmanovskiy pereulok, 3, Moscow, 127994, Russian Federation

Russia	643004	Ethics Committee of Arkhangelsk Clinical Oncology Dispensary, Bld.1, 145, Obvodniy Kanal Prospekt, Arkhangelsk, 163045, Russian Federation	Ethics Council at the Ministry of Healthcare of the Russian Federation, Rakhmanovskiy pereulok, 3, Moscow, 127994, Russian Federation
Russia	643005	Federal State Budgetary Institution “National Medical Research Centre of Oncology named after N.N. Petrov” of the Ministry of Healthcare of the Russian Federation, 68, Leningradskaya Street, pos. Pesochny, Saint Petersburg, 197758, Russian Federation	Ethics Council at the Ministry of Healthcare of the Russian Federation, Rakhmanovskiy pereulok, 3, Moscow, 127994, Russian Federation
Russia	643006	Federal State Budgetary Educational Institution of Higher Education “National Research Ogarev Mordovia State University”, Medical Institute, 26a, Ulyanov Street, Saransk, Republic of Mordovia, 430032, Russian Federation	Ethics Council at the Ministry of Healthcare of the Russian Federation, Rakhmanovskiy pereulok, 3, Moscow, 127994, Russian Federation
Russia	643007	Independent Ethics Committee of “Arte Med Assistance” LLC, Office 44, Lit. Ts, 27 Prospekt Engelsa Saint Petersburg, 194156, Russian Federation	Ethics Council at the Ministry of Healthcare of the Russian Federation, Rakhmanovskiy pereulok, 3, Moscow, 127994, Russian Federation
Russia	643008	Federal State Budgetary Educational Institution of Higher Education “Siberian State Medical University” of Ministry of Healthcare of Russia, 15, Kotovskogo Street, Tomsk, 634034, Russian Federation	Ethics Council at the Ministry of Healthcare of the Russian Federation, Rakhmanovskiy pereulok, 3, Moscow, 127994, Russian Federation
Russia	643009	Ethics Committee at the State Budgetary Healthcare Institution of Kaluga Region “Kaluga Regional Clinical Oncology Dispensary”, 2, Vishnevskogo Street, Kaluga, Kaluga Region, 248007, Russian Federation	Ethics Council at the Ministry of Healthcare of the Russian Federation, Rakhmanovskiy pereulok, 3, Moscow, 127994, Russian Federation
Russia	643011	Committee for Biomedical Ethics at the Research Institute of Oncology of Tomsk National Research Medical Centre ul. Savinykh, 12/1, Tomsk Region, Tomsk, 634028, Russian Federation	Ethics Council at the Ministry of Healthcare of the Russian Federation, Rakhmanovskiy pereulok, 3, Moscow, 127994, Russian Federation
Russia	643012	Ethics Committee of State Budgetary Healthcare Institution of Kemerovo Region “Regional Clinical Oncology Dispensary” 35, Volgogradskaya Steet, Kemerovo, Kemerovo Region, 650036, Russian Federation	Ethics Council at the Ministry of Healthcare of the Russian Federation, Rakhmanovskiy pereulok, 3, Moscow, 127994, Russian Federation

Russia	643013	Ethics Committee at the Private Institution Educational Organisation of Higher Education Reaviz Medical University, ul. Chkalova, d. 100, Samara, 443030, Russian Federation	Ethics Council at the Ministry of Healthcare of the Russian Federation, Rakhmanovskiy pereulok, 3, Moscow, 127994, Russian Federation
Russia	643014	Ethics Committee of Federal State Budgetary Institution “N. N. Blokhin National Medical Research Center of Oncology” of the Ministry of Health of the Russian Federation, 24, Kashirskoe Shosse, Moscow, 115478, Russian Federation	Ethics Council at the Ministry of Healthcare of the Russian Federation, Rakhmanovskiy pereulok, 3, Moscow, 127994, Russian Federation
Russia	643015	Local Ethics Committee of "EVIMED" LLC, Offices 10, 22, 9-v, Blyukhera Street., Chelyabinsk, 454048, Russian Federation	Ethics Council at the Ministry of Healthcare of the Russian Federation, Rakhmanovskiy pereulok, 3, Moscow, 127994, Russian Federation
Russia	643016	Ethics Committee of "Komanda" LLC, Room 37-N, Lit. A, Bld. 2, 19 Frunze Street, Saint-Petersburg, 196135, Russian Federation	Ethics Council at the Ministry of Healthcare of the Russian Federation, Rakhmanovskiy pereulok, 3, Moscow, 127994, Russian Federation
Russia	643017	Federal State Budgetary Educational Institution of Higher Education Bashkir State Medical University of the Ministry of Healthcare of the Russian Federation ul. Lenina 3, Ufa, Republic of Bashkortostan, 450008, Russian Federation	Ethics Council at the Ministry of Healthcare of the Russian Federation, Rakhmanovskiy pereulok, 3, Moscow, 127994, Russian Federation
Russia	643018	Independent Interdisciplinary Ethics Committee on Ethical Review for Clinical Studies, 51, Leningradskiy Avenue, Moscow, 125468, Russian Federation	Ethics Council at the Ministry of Healthcare of the Russian Federation, Rakhmanovskiy pereulok, 3, Moscow, 127994, Russian Federation
Russia	643019	Ethics Committee at the State Budgetary Healthcare Institution “Leningrad Regional Clinical Oncology Dispensary”, Liteyny Prospect 37-39, Saint Petersburg, 191014, Russian Federation	Ethics Council at the Ministry of Healthcare of the Russian Federation, Rakhmanovskiy pereulok, 3, Moscow, 127994, Russian Federation
Russia	643020	State Autonomous Healthcare Institution Republican Clinical Oncology Center of the Ministry of Healthcare of the Republic of Tatarstan, Sibirskiy Trakt, 29, Kazan, 420029, Russian Federation	Ethics Council at the Ministry of Healthcare of the Russian Federation, Rakhmanovskiy pereulok, 3, Moscow, 127994, Russian Federation

Russia	643021	State Budgetary Healthcare Institution of Stavropol Region “Pyatigorsk Interdistrict Oncology Dispensary”, 31, Kalinina Prospect, Pyatigorsk, Stavropol Territory, 357502, Russian Federation	Ethics Council at the Ministry of Healthcare of the Russian Federation, Rakhmanovskiy pereulok, 3, Moscow, 127994, Russian Federation
Russia	643022	Committee on Ethics Expertise of Clinical Studies of Regional Budgetary Healthcare Institution “Belgorod Oncology Dispensary” 1, Kuybysheva Street, Belgorod, 308010, Russian Federation	Ethics Council at the Ministry of Healthcare of the Russian Federation, Rakhmanovskiy pereulok, 3, Moscow, 127994, Russian Federation
Russia	643023	Ethics Committee of Budgetary Healthcare Institution of Omsk Region “Clinical Oncology Dispensary”, Bld. 1, 9, Zavertyayeva Street, Omsk, 644013, Russian Federation	Ethics Council at the Ministry of Healthcare of the Russian Federation, Rakhmanovskiy pereulok, 3, Moscow, 127994, Russian Federation
Russia	643024	Independent Interdisciplinary Ethics Committee on Ethical Review for Clinical Studies, 51, Leningradskiy Avenue, Moscow, 125468, Russian Federation	Ethics Council at the Ministry of Healthcare of the Russian Federation, Rakhmanovskiy pereulok, 3, Moscow, 127994, Russian Federation
Thailand	764001	Ethics Committee in Human Research, Udonthani Cancer Hospital, 36 Moo 1, Udon-Khon Kean Road, Muang, Udonthani, 41330, Thailand	Central Research Ethics Committee, 5th Floor, Building 2, The National Research Council of Thailand, Paholyothin Road, Lad Yao Sub-district, Chatuchak District, Bangkok, 10900, Thailand
Thailand	764002	Naresuan University Institutional Review Board, 99 Moo 9 Tha Pho, Muang, Phitsanulok, 65000, Thailand	Central Research Ethics Committee, 5th Floor, Building 2, The National Research Council of Thailand, Paholyothin Road, Lad Yao Sub-district, Chatuchak District, Bangkok, 10900, Thailand
Thailand	764003	Lopburi Cancer Hospital Ethics Committee for Human Research, 11/1 Paholyothin Road, Thalae Chup Son, Mueang, Lopburi, 15000, Thailand	Central Research Ethics Committee, 5th Floor, Building 2, The National Research Council of Thailand, Paholyothin Road, Lad Yao Sub-district, Chatuchak District, Bangkok, 10900, Thailand
Thailand	764004	Institution Review Board, Royal Thai Army Medical Department, 5th floor Phramongkutklao wejvitya Building, Phramongkutklao College of Medicine, 317/5 Rajavithi Road, Rajathevee, Bangkok, 10400, Thailand	Central Research Ethics Committee, 5th Floor, Building 2, The National Research Council of Thailand, Paholyothin Road, Lad Yao Sub-district, Chatuchak District, Bangkok, 10900, Thailand

Thailand	764005	The Ethics Committee of Lampang Cancer Hospital, 199 Moo 12, Pichai, Muang, Lampang, 52000, Thailand	Central Research Ethics Committee, 3rd Floor. Building 3, The National Research Council of Thailand, 196 Moo 5, Phaholyothin Road, Lad Yao, Chatuchak, Bangkok, 10900, Thailand
Thailand	764007	Human Research Ethics Committee Faculty of Medicine, Prince of Songkla University, 15 Karnjanavanit Road, Hat Yai, Songkhla, 90110, Thailand	Central Research Ethics Committee, 5th Floor, Building 2, The National Research Council of Thailand, Paholyothin Road, Lad Yao Sub-district, Chatuchak District, Bangkok, 10900, Thailand
Thailand	764008	The Ethical Committee for Research in Human Subject, Chiangrai Prachanukroh Hospital, 1039 Sathanpayaban Road, Muang, Chiang Rai, 57000, Thailand	Central Research Ethics Committee, 5th Floor, Building 2, The National Research Council of Thailand, Paholyothin Road, Lad Yao Sub-district, Chatuchak District, Bangkok, 10900, Thailand
Thailand	764009	Khon Kaen Hospital Institute Review Board in Human Research, 54, 56 Sri Chan Road, Naimaung Sub-District, Muang District, Khon Kaen, 40000, Thailand	Central Research Ethics Committee, 5th Floor, Building 2, The National Research Council of Thailand, Paholyothin Road, Lad Yao Sub-district, Chatuchak District, Bangkok, 10900, Thailand
Turkey	792002<br>792004<br>792012<br>792014		Ankara University Faculty of Medicine Clinical Trials Ethics Committee, Ankara Üniversitesi Tıp Fakültesi Morfoloji Binası, Sıhhiye/Ankara, 6100, Turkey
Ukraine	804002	Local Ethics Committee of MI “City Dnipropetrovsk Multi-Field Clinical Hospital #4 of DRC”, 31, Blizhniaya Street, Dnipro, 49102, Ukraine	
Ukraine	804004	Ethics Committee of Medical and Diagnostic Center of Private Enterprise of Private Production Company “Acinus”, 65, Velyka Perspektyvna Street, Kropyvnytskyi, 25006, Ukraine	
Ukraine	804006	Ethics Committee of Municipal Non-Profit Enterprise "Regional Oncology Center", 4, Lisoparkivska Street, Kharkiv, 61070, Ukraine	
Ukraine	804007	Ethics Committee of Podilsky Regional Center of Oncology, 84, Khmelnytske Highway, Vinnytsia, 21029, Ukraine	
Ukraine	804008	Ethics Committee of National Cancer Institute, 33/43, Lomonosova Street, Kyiv, 03022, Ukraine	

Ukraine	804013	Ethics Committee at Municipal Non-profit Enterprise “Transcarpathian Antitumor Center” of the Transcarpathian Regional Council, 2, Brodlakovych Street, Uzhorod, 88014, Ukraine	

EudraCT Number: 2017-001311-36
IND Number: 134016

Regeneron Pharmaceuticals, Inc.

Clinical Study Protocol

A RANDOMIZED, PHASE 3, OPEN-LABEL STUDY OF COMBINATIONS OF REGN2810 (ANTI-PD-1 ANTIBODY), IPILIMUMAB (ANTI-CTLA-4 ANTIBODY), AND PLATINUM-BASED DOUBLET CHEMOTHERAPY IN FIRST-LINE TREATMENT OF PATIENTS WITH ADVANCED OR METASTATIC NON-SMALL CELL LUNG CANCER WITH TUMORS EXPRESSING PD-L1 <50%

**Compound:**	REGN2810 (anti-PD-1 mAb)
**Clinical Phase:**	3
**Protocol Number:**	R2810-ONC-16113
**Date of Issue:**	*See appended electronic signature page*
**Scientific/Medical Monitor:**	[redacted]<br>Regeneron Pharmaceuticals, Inc.<br>777 Old Saw Mill River Road<br>Tarrytown, NY 10591

[redacted]

VV-RIM-00023209-1.0 Approved - 29 Aug 2017 GMT-5:00

CLINICAL STUDY PROTOCOL SYNOPSIS

**Title**	A Randomized, Phase 3, Open-label Study of Combinations of REGN2810 (Anti-PD-1 Antibody), Ipilimumab (Anti-CTLA-4 Antibody), and Platinum-based Doublet Chemotherapy in First-line Treatment of Patients with Advanced or Metastatic Non-Small Cell Lung Cancer With Tumors Expressing PD-L1 <50%
**Site Locations**	Patients will be randomized at approximately 200 global study sites.
**Objectives**	The primary objective of the study is to compare the progression-free survival (PFS) of REGN2810 plus 4 to 6 cycles of standard-of-care platinum-based doublet chemotherapy combination therapy (REGN2810/chemo-f) and REGN2810 plus 2 cycles only of standard-of-care platinum-based doublet chemotherapy plus ipilimumab combination therapy (REGN2810/chemo-l/ipi) with standard-of-care platinum-based doublet chemotherapy in the first-line treatment of patients with advanced squamous or non-squamous non-small cell lung cancer (NSCLC) in the subgroup of patients whose tumors express programmed cell death ligand 1 (PD-L1) in 1% to <50% of tumor cells and in the overall population of study patients whose tumors express PD-L1 in <50% of tumor cells.<br><br>The key secondary objectives of the study are the following:<br><br>• To compare the overall survival (OS) of REGN2810/chemo-f and REGN2810/chemo-l/ipi versus standard-of-care platinum-based doublet chemotherapy in the first-line treatment of patients with advanced squamous or non-squamous NSCLC in the subgroup of patients whose tumors express PD-L1 in 1% to <50% of tumor cells and in the overall population of study patients whose tumors express PD-L1 in <50% of tumor cells<br><br>• To compare the objective response rate (ORR) of REGN2810/chemo-f and REGN2810/chemo-l/ipi versus standard-of-care platinum-based doublet chemotherapy in the first-line treatment of patients with advanced squamous or non-squamous NSCLC in the subgroup of patients whose tumors express PD-L1 in 1% to <50% of tumor cells and in the overall population of study patients whose tumors express PD-L1 in <50% of tumor cells<br><br>The other secondary objectives are the following:<br><br>• To evaluate the safety and tolerability of REGN2810/chemo-f and REGN2810/chemo-l/ipi compared to standard-of-care platinum-based doublet chemotherapy<br><br>• To compare the OS at 12 and 18 months of REGN2810/chemo-f or REGN2810/chemo-l/ipi versus standard-of-care platinum-based doublet chemotherapy in the first-line treatment of patients with

	advanced squamous or non-squamous NSCLC in the subgroup of patients whose tumors express PD-L1 in 1% to <50% of tumor cells and in the overall population of study patients whose tumors express PD-L1 in <50% of tumor cells<br>• To compare quality of life (QOL) in patients with advanced squamous or non-squamous NSCLC whose tumors express PD-L1 in <50% of tumor cells receiving REGN2810/chemo-f or REGN2810/chemo-l/ipi versus those receiving standard-of-care platinum-based doublet chemotherapy<br>• To assess immunogenicity as measured by anti-drug antibodies (ADAs) for REGN2810<br>• To assess the predictive utility of baseline PD-L1 tumor expression levels on indicators of clinical response using PD-L1 assays other than the clinical trial assay<br>• To characterize the pharmacokinetics (PK) of REGN2810 when administered in combination with ipilimumab or in combination with platinum-based doublet chemotherapy<br>• To conduct exposure-response (E-R) analyses for relevant biomarkers (exploratory PK/pharmacodynamic analyses) and E-R analyses for safety and efficacy endpoints, as appropriate
**Study Design**	This is a phase 3, randomized, global, open-label, efficacy and safety study of REGN2810/chemo-f versus REGN2810/chemo-l/ipi versus standard-of-care platinum-based doublet chemotherapy in the first-line treatment of patients with stage IIIB or stage IV squamous or non-squamous NSCLC, whose tumors express PD-L1 in <50% of tumor cells and who have received no prior systemic treatment for their advanced disease.<br>The study will consist of the following 3 periods: screening, treatment, and follow-up.<br>Patients will undergo a screening evaluation to determine their eligibility within 28 days prior to randomization. Eligible patients will be randomized 1:1:1 to one of the following treatment arms:<br>• Treatment Arm A: standard-of-care platinum-based doublet chemotherapy every 3 weeks (Q3W) for 4 to 6 cycles (followed by optional pemetrexed maintenance for those patients initially assigned to receive a pemetrexed-containing regimen)<br>• Treatment Arm B: REGN2810 350 mg Q3W for 108 weeks plus standard-of-care platinum-based doublet chemotherapy for 4 to 6 cycles (referred to as "REGN2810/chemo-f" hereinafter)<br>• Treatment Arm C: REGN2810 350 mg Q3W for 108 weeks plus standard-of-care platinum-based doublet chemotherapy for 2 cycles

VV-RIM-00023209-1.0 Approved - 29 Aug 2017 GMT-5:00

	and ipilimumab 50 mg every 6 weeks (Q6W) for up to 4 doses (referred to as "REGN2810/chemo-l/ipi" hereinafter)<br><br>Patients will receive their assigned treatment for the treatment period (as noted above). Treatment may be discontinued early due to Response Evaluation Criteria in Solid Tumors Version 1.1 (RECIST 1.1)-defined progressive disease, unacceptable toxicity, withdrawal of consent, death, initiation of another anti-cancer treatment, or, for patients in Treatment Arms B and C, in specific instances of confirmed complete response (CR) or partial response (PR).<br><br>Patients who experience RECIST 1.1-defined progressive disease on therapy may continue treatment if the investigator judges the patient to be experiencing clinical benefit and if the patient has not completed the 108-week treatment period. If further progressive disease (defined as an additional 10% increase in tumor burden from the time of initial progressive disease) is confirmed, therapy must be discontinued and other anti-cancer therapy considered, if appropriate. A similar approach to treatment beyond first evidence of progression may be offered to patients receiving pembrolizumab in Treatment Arm A.<br><br>After discontinuing study treatment, patients will enter the follow-up period.<br><br>Each patient will have the first follow-up visit 14 to 30 days (±7 days) after the last study treatment, if treatment is discontinued early due to progressive disease, toxicity, or for another reason, or 14 to 30 days (±7 days) after the last cycle visit. Follow-up visit 2 through follow-up visit 7 will occur 28 days (±7 days) from the previous visit. Survival data will then be collected by phone or at an office visit every 3 months, until death, loss to follow-up, or withdrawal of study consent.<br><br>Because this is the first study of REGN2810/chemo-l/ipi, safety data from the first 10 patients treated with REGN2810/chemo-l/ipi in Treatment Arm C will be reviewed after these patients have had 4 weeks of follow-up following the first dose of REGN2810/chemo-l/ipi. The data will be reviewed at a meeting of the Independent Data Monitoring Committee (IDMC). If 2 or more dose-limiting toxicities occur in the first 10 patients treated in Treatment Arm C, enrollment for this treatment arm will be stopped temporarily and will be restarted only after a formal safety review.
**Study Duration**	The approximate duration of the active study assessments for each patient, excluding screening, will be 24 to 32 months. For Treatment Arms B and C, this encompasses 25 months of study treatment plus 7 months of follow-up. For patients in Treatment Arm A, this encompasses 4 to 6 cycles of study treatment (and pemetrexed maintenance, if appropriate) and radiographic tumor assessments for up to 2 years. After the active study assessments are complete, all patients will be followed for survival.

**Population**	
**Sample Size:**	Approximately 690 patients will be randomized.
**Target Population:**	Patients in this study will include men and women ≥18 years of age, diagnosed with stage IIIB or stage IV non-squamous or squamous NSCLC, whose tumors express PD-L1 in <50% of tumor cells (measured using the PD-L1 immunohistochemistry 22C3 pharmDx assay [Dako, North America, Inc.]) and who have received no prior systemic treatment for their advanced disease.
**Treatments**	
**Study Drug Dose/Route/Schedule:**	REGN2810 administered at 350 mg as an intravenous (IV) infusion Q3W for 108 weeks in combination with standard-of-care platinum-based doublet chemotherapy Q3W administered IV for 4 to 6 cycles.
**Study Drug Dose/Route/Schedule:**	REGN2810 administered at 350 mg as an IV infusion Q3W for 108 weeks in combination with standard-of-care platinum-based doublet chemotherapy Q3W administered IV for 2 cycles and ipilimumab administered IV over approximately 90 minutes at 50 mg Q6W for up to 4 doses.
**Reference Drug Dose/Route/Schedule:**	Standard-of-care platinum-based doublet chemotherapy administered IV Q3W for 4 to 6 cycles (followed by optional pemetrexed maintenance for those patients initially assigned to receive a pemetrexed-containing regimen).
**Endpoints**	
**Primary:**	The primary endpoint is PFS as assessed by a blinded Independent Review Committee (IRC) based on RECIST 1.1 assessments.
**Secondary:**	The key secondary endpoints in the study are OS and ORR.<br>Other secondary endpoints will include the following:<br>• The safety and tolerability of REGN2810/chemo-l-ipi and REGN2810/chemo-f measured by the incidence of treatment-emergent adverse events (TEAEs), dose-limiting toxicities (DLTs), serious adverse events (SAEs), deaths, and laboratory abnormalities<br>• Overall survival at 12 months, 18 months, and end of treatment<br>• Quality of life as measured by the European Organization for Research and Treatment of Cancer Quality of Life Questionnaire Core 30 (EORTC QLQ-C30) and Quality of Life Questionnaire Lung Cancer 13 (EORTC QLQ-LC13)

VV-RIM-00023209-1.0 Approved - 29 Aug 2017 GMT-5:00

**Procedures and Assessments**	Procedures to be performed at screening will include informed consent; assessment of inclusion/exclusion criteria; recording of medical, oncology, and concomitant medications histories; recording of demographics; collection and testing of tumor tissue samples for PD-L1 assessment and for epidermal growth factor receptor and anaplastic lymphoma kinase mutations and C-ros oncogene receptor tyrosine kinase fusions; radiographic tumor assessment; tumor burden assessment; chest X-ray; serum pregnancy testing; 12-lead electrocardiogram; adverse event (AE) recording; physical examination, including vital signs, height, and weight assessments; Eastern Cooperative Oncology Group (ECOG) performance status assessment; and laboratory testing. Samples for an optional genomic sub-study may also be obtained.<br><br>During the treatment period, the following procedures will be performed to assess efficacy and safety: QOL measurement using validated patient questionnaires, physical examination, ECOG performance status assessment; vital signs; laboratory testing, including pregnancy testing for women of childbearing potential; recording of AEs and concomitant medications. Computed tomography or magnetic resonance imaging (or positron emission tomography) for radiographic tumor burden assessment and tumor burden assessment based on RECIST 1.1 criteria will be performed at pre-specified time points throughout the study.<br><br>Other assessments will include REGN2810 concentration measurement, REGN2810 ADA assessment, and biomarker assessments. Biomarker procedures will include the use of tumor tissue samples for validation of additional PD-L1 assays.<br><br>Survival data will then be collected by phone or at an office visit every 3 months, until death, loss to follow-up, or withdrawal of study consent.
**Statistical Plan**	The primary statistical hypotheses are that REGN2810/chemo-f or REGN2810/chemo-l/ipi will prolong PFS as compared with platinum-based standard-of-care doublet chemotherapy in the subgroup of patients whose tumors express PD-L1 in 1% to <50% of tumor cells and in the overall population of study patients whose tumors express PD-L1 in <50% of tumor cells. The secondary hypotheses are that REGN2810/chemo-f or REGN2810/chemo-l/ipi will improve OS in the subgroup of patients whose tumors express PD-L1 in 1% to <50% of tumor cells and in the overall population of study patients whose tumors express PD-L1 in <50% of tumor cells.<br><br>The study assumes a median PFS of 6 months for patients treated with chemotherapy alone and a median PFS of 10 months for patients treated with each of the REGN2810 combination therapies. The assumptions correspond to a 66.7% increase in median PFS and a hazard ratio (HR) of 0.6. Under these assumptions, and for each REGN2810 combination treatment arm versus the standard-of-care chemotherapy comparison, 190 PFS events are

VV-RIM-00023209-1.0 Approved - 29 Aug 2017 GMT-5:00

	needed to yield approximately 90% power to detect statistical significance at 2-sided 0.025 level.<br><br>Considering a uniform enrollment rate and a combined enrollment and PFS follow-up duration of approximately 18 months (12 months for enrollment and 6 months follow-up for PFS) and 10% dropout rate per year, enrollment of approximately 477 randomized patients (159 patients per arm) in the subgroup of patients whose tumors express PD-L1 in 1% to <50% of tumor cells is needed to generate enough PFS events to yield approximately 90% power for each REGN2810 combination treatment arm versus the standard-of-care chemotherapy comparison treatment arm.<br><br>It is projected that approximately 70% of the patients whose tumors express PD-L1 in <50% of tumor cells will have tumors that express PD-L1 in 1% to <50% of tumor cells. Therefore, approximately 690 patients will be randomized into this study in order to separately test and have power for the subgroup of patients whose tumors express PD-L1 in 1% to <50% of tumor cells.<br><br>In the subgroup of patients whose tumors express PD-L1 in 1% to <50% of tumor cells (as determined by the PD-L1 IHC 22C3 pharmDx assay), it is projected that an observed HR of 0.72 or lower, which corresponds to an increase in median PFS of 2.3 months or more (6 months versus 8.3 months), would result in a statistically significant improvement in PFS.<br><br>The primary and secondary endpoints will be tested in the following order: PFS, OS, and ORR.<br><br>The primary endpoint of PFS will be analyzed first in the subgroup of patients whose tumors express PD-L1 in 1% to <50% of tumor cells and will be performed when approximately 110 PFS events are observed in the subgroup and in the standard-of-care chemotherapy arm. If the primary analysis of PFS for a REGN2810 combination versus standard-of-care chemotherapy is statistically significant at a 2-sided alpha=0.025 level in the subgroup of patients whose tumors express PD-L1 in 1% to <50% tumor cells, the primary analysis of PFS for that REGN2810 combination versus standard-of-care chemotherapy will then be performed in the overall study population (patients whose tumors express PD-L1 in <50% of tumor cells).<br><br>In the subgroup of patients whose tumors express PD-L1 in 1% to <50% of tumor cells and in the overall study population, the primary endpoint of PFS will be analyzed by stratified log-rank test using the status of histology (non-squamous versus squamous) and levels of PD-L1 expression (<1% versus 1% to <25% versus 25% to <50%) as stratification factors. The HR and the corresponding 95% confidence interval will be estimated by a stratified Cox regression model using treatment as covariate.<br><br>For the 2 REGN2810 combinations versus standard of care chemotherapy comparisons, familywise type-I error rate of 0.05 is controlled by Bonferroni

	approach (split alpha into 0.025 for each comparison).<br>Safety observations and measurements, including drug exposure, AEs, laboratory data, vital signs, and ECOG performance status, will be summarized and presented in tables and listings.<br>REGN2810 concentrations in serum will be reported over time as individual values with descriptive statistics.<br>For continuous variables, descriptive statistics will include the following information: the number of patients reflected in the calculation (n), mean, median, standard deviation, minimum, and maximum. Baseline characteristics and AEs will be summarized using descriptive statistics.

CONFIDENTIAL

VV-RIM-00023209-1.0 Approved - 29 Aug 2017 GMT-5:00

LIST OF ABBREVIATIONS AND DEFINITIONS OF TERMS

ACTH	Adrenocorticotropic hormone
ADA(s)	Anti-drug antibody(ies)
ADL	Activities of daily living
AE	Adverse event
AESI	Adverse event of special interest
ALK	Anaplastic lymphoma kinase
ALT	Alanine aminotransferase
Anti-CTLA-4	Anti-cytotoxic T-lymphocyte-associated antigen 4
Anti-PD-1	Anti-programmed death-1
Anti-PD-L1	Anti-programmed death ligand 1
AST	Aspartate aminotransferase
ATC	Anatomical therapeutic chemical
AUC	Area under the curve
BOR	Best overall response
BUN	Blood urea nitrogen
C2P1	Cell Line 2 Process 1
CBC	Complete blood count
CI	Confidence interval
C_{eoi}	Concentration at end of infusion
CHO	Chinese hamster ovary
CNS	Central nervous system
CR	Complete response
CRF	Case report form
CRO	Contract research organization
CSCC	Cutaneous squamous cell carcinoma
CT	Computed tomography
CTLA-4	Cytotoxic T-lymphocyte-associated protein 4
C_{trough}	Pre-infusion concentration
CTX	Cyclophosphamide
CV%	Variability in exposure
DLT	Dose-limiting toxicity
DNA	Deoxyribonucleic acid
DOR	Duration of response
EC	Ethics Committee

ECG	Electrocardiogram
ECOG	Eastern Cooperative Oncology Group
EDC	Electronic data capture
EGFR	Epidermal growth factor receptor
EORTC QLQ-C30	European Organization for Research and Treatment of Cancer Quality of Life Questionnaire Core 30
EORTC QLQ-LC13	European Organization for Research and Treatment of Cancer Quality of Life Questionnaire Lung Cancer 13
E-R	Exposure-response
EU	European Union
FAS	Full analysis set
FDA	Food and Drug Administration
FIH	First-in-human
GCP	Good Clinical Practice
GFR	Glomerular filtration rate
hfRT	Hypofractionated radiation therapy
HR	Hazard ratio
ICF	Informed consent form
ICH	International Council for Harmonisation
IDMC	Independent Data Monitoring Committee
IHC	Immunohistochemistry
irAE	Immune-related adverse event
IRB	Institutional Review Board
IRC	Independent Review Committee
irTEAE	Immune-related treatment-emergent adverse event
IV	Intravenous
IVRS	Interactive voice response system
IWRS	Interactive web response system
LDH	Lactate dehydrogenase
MedDRA	Medical Dictionary for Regulatory Activities
MRI	Magnetic resonance imaging
MTD	Maximum tolerated dose
N/A	Not applicable
NAb	Neutralizing anti-drug antibody
NCI-CTCAE	National Cancer Institute: Common Terminology Criteria for Adverse Events
NE	Not evaluable
NSCLC	Non-small cell lung cancer

ORR	Objective response rate
OS	Overall survival
PD	Progressive disease
PD-1	Programmed cell death-1
PD-L1	Programmed cell death ligand 1
PD-L2	Programmed cell death ligand 2
PE	Physical examination
PET	Positron emission tomography
PFS	Progression-free survival
PK	Pharmacokinetic(s)
PR	Partial response
PT	Preferred Term
PT/PTT	Prothrombin time/Partial thromboplastin time
QOL	Quality of life
Q2W	Every 2 weeks
Q3W	Every 3 weeks
Q6W	Every 6 weeks
Q9W	Every 9 weeks
Q12W	Every 12 weeks
Q18W	Every 18 weeks
Q24W	Every 24 weeks
RBC	Red blood cell
RECIST 1.1	Response Evaluation Criteria in Solid Tumors version 1.1
Regeneron	Regeneron Pharmaceuticals, Inc.
RNA	Ribonucleic acid
ROS1	C-ros oncogene receptor tyrosine kinase
SAE	Serious adverse event
SAF	Safety analysis set
SAP	Statistical analysis plan
SD	Stable disease
SmPC	Summary of Product Characteristics
SOC	System organ class
T4	Thyroxine
TEAE	Treatment-emergent adverse event
t_{eoi}	Time of end of infusion
TSH	Thyroid-stimulating hormone

ULN	Upper limit of normal
US	United States
WBC	White blood cell

VV-RIM-00023209-1.0 Approved - 29 Aug 2017 GMT-5:00

TABLE OF CONTENTS

CLINICAL STUDY PROTOCOL SYNOPSIS....2
LIST OF ABBREVIATIONS AND DEFINITIONS OF TERMS....9
TABLE OF CONTENTS....13
LIST OF TABLES....18
LIST OF FIGURES....19
LIST OF APPENDICES....19
1. INTRODUCTION....20
2. STUDY OBJECTIVES....24
2.1. Primary Objective....24
2.2. Secondary Objectives....24
2.2.1. Key Secondary Objectives....24
2.2.2. Other Secondary Objectives....24
3. HYPOTHESIS AND RATIONALE....25
3.1. Hypothesis....25
3.2. Rationale....26
3.2.1. Rationale for Study Design....26
3.2.2. Rationale for Endpoints and Objectives....26
3.2.3. Rationale for Choice of Patient Population Based on the <50% PD-L1 Expression Cut-off....27
3.2.4. Rationale for Combination of REGN2810 and Ipilimumab and Limited (2 Cycles) Platinum-Based Doublet Chemotherapy....27
3.2.5. Rationale for REGN2810 Dose Selection....29
3.2.6. Rationale for Ipilimumab Dose Selection....30
3.2.7. Rationale for Standard-of-Care Platinum-Based Chemotherapy as Comparator....30
4. STUDY VARIABLES....31
4.1. Demographic and Baseline Characteristics....31
4.2. Primary and Secondary Endpoints....32
4.2.1. Primary Endpoint....32
4.2.2. Key Secondary Endpoints....32
4.2.3. Other Secondary Endpoints....32
4.3. Pharmacokinetic Variables....32

4.4. Anti-Drug Antibody Variables 33
5. STUDY DESIGN 33
5.1. Study Description and Duration 33
5.1.1. Screening 35
5.1.2. Treatment Period 35
5.1.3. Follow-Up Period 38
5.1.4. Description of Study Stopping Rules 38
5.1.5. Dose-Limiting Toxicities....39
5.1.6. Maximum Tolerated Dose 40
5.1.7. End of Study Definition....40
5.2. Planned Interim Analysis....40
5.3. Study Committees....40
5.3.1. Independent Review Committee....41
5.3.2. Independent Data Monitoring Committee 41
6. SELECTION, WITHDRAWAL, AND REPLACEMENT OF PATIENTS....41
6.1. Number of Patients Planned 41
6.2. Study Population....41
6.2.1. Inclusion Criteria 41
6.2.2. Exclusion Criteria 42
6.3. Premature Withdrawal from the Study 44
6.4. Replacement of Patients 45
7. STUDY TREATMENTS....45
7.1. Investigational and Reference Treatments....45
7.1.1. REGN2810 45
7.1.2. Ipilimumab....46
7.1.3. Platinum-based Doublet Chemotherapy 46
7.2. Pre-treatments 49
7.3. Dose Modification and Study Treatment Discontinuation Rules 49
7.3.1. REGN2810 Plus Ipilimumab and Chemotherapy Combination Therapy 49
7.3.2. REGN2810 as Combination Therapy With Chemotherapy 52
7.3.3. Immune-Related Adverse Events 54
7.3.4. Ipilimumab....56
7.3.5. Platinum-Based Doublet Chemotherapy 56

CONFIDENTIAL

VV-RIM-00023209-1.0 Approved - 29 Aug 2017 GMT-5:00

7.3.6. Permanent Study Drug Discontinuation 57
7.4. Management of Acute Reactions 57
7.4.1. Acute Infusion Reactions 57
7.4.1.1. Interruption of the Infusion 58
7.4.1.2. Termination of the Infusion 59
7.5. Method of Treatment Assignment 59
7.5.1. Blinding/Masking 60
7.6. Treatment Logistics and Accountability 60
7.6.1. Packaging, Labeling, and Storage 60
7.6.2. Supply and Disposition of Treatments 60
7.6.3. Treatment Accountability 61
7.6.4. Treatment Compliance 61
7.7. Concomitant Medications 61
7.7.1. Prohibited Medications and Procedures 61
7.7.2. Permitted Medications and Procedures 62
7.8. Disease Progression While Receiving REGN2810 62
7.9. Post-study Treatments and Procedures 63
8. STUDY SCHEDULE OF EVENTS AND PROCEDURES 63
8.1. Schedule of Events 63
8.1.1. Footnotes for the Schedule of Events Table 76
8.1.2. Early Termination Visit 76
8.1.3. Unscheduled Visits 76
8.2. Study Procedures 76
8.2.1. Procedures Performed Only at the Screening/Baseline Visit 76
8.2.2. Efficacy Procedures 77
8.2.2.1. Radiographic Tumor Assessments 77
8.2.2.2. Tumor Burden Assessments 78
8.2.2.3. Quality of Life Questionnaires 78
8.2.2.4. Survival Data Collection 79
8.2.3. Safety Procedures 79
8.2.3.1. Physical Examination 79
8.2.3.2. ECOG Performance Status 79
8.2.3.3. Weight and Height 79

VV-RIM-00023209-1.0 Approved - 29 Aug 2017 GMT-5:00

8.2.3.4. Vital Signs 79
8.2.3.5. 12-Lead Electrocardiogram 80
8.2.3.6. Laboratory Testing....................80
8.2.3.7. Concomitant Medication Recording....................81
8.2.3.8. Adverse Event Recording 81
8.2.4. Pharmacokinetic and Anti-Drug Antibody Procedures 81
8.2.4.1. Drug Concentration Measurements and Samples....................81
8.2.4.2. Anti-Drug Antibody Measurements and Samples....................82
8.2.5. Biomarker Procedures 82
8.2.6. Future Biomedical Research....................83
8.2.6.1. Genomics Sub-Study - Optional....................83
9. SAFETY DEFINITIONS, REPORTING, AND MONITORING 84
9.1. Obligations of Investigator 84
9.2. Obligations of Sponsor 84
9.3. Definitions 84
9.3.1. Adverse Event....................84
9.3.2. Serious Adverse Event....................85
9.3.3. Adverse Events of Special Interest 85
9.3.3.1. Immune-Related Adverse Events 86
9.3.4. Infusion Reactions 86
9.4. Recording and Reporting Adverse Events....................86
9.4.1. Adverse Events 86
9.4.2. Serious Adverse Events 87
9.4.3. Other Events that Require Accelerated Reporting to Sponsor 87
9.4.4. Reporting Adverse Events Leading to Withdrawal from the Study 87
9.4.5. Abnormal Laboratory, Vital Signs, or Electrocardiogram Results....................87
9.4.6. Follow-up....................88
9.5. Evaluation of Severity and Causality 88
9.5.1. Evaluation of Severity 88
9.5.2. Evaluation of Causality....................89
9.6. Safety Monitoring....................89
9.7. Investigator Alert Notification....................89
10. STATISTICAL PLAN....................90

CONFIDENTIAL

VV-RIM-00023209-1.0 Approved - 29 Aug 2017 GMT-5:00

10.1. Statistical Hypothesis....90
10.2. Justification of Sample Size....90
10.3. Analysis Sets....91
10.3.1. Efficacy Analysis Sets....91
10.3.2. Safety Analysis Set....91
10.3.3. Other Analysis Sets....91
10.4. Statistical Methods....91
10.4.1. Patient Disposition....92
10.4.2. Demography and Baseline Characteristics....92
10.4.3. Medical History....92
10.4.4. Prior Medications/Concomitant Medications....92
10.4.5. Efficacy Analyses....93
10.4.5.1. Primary Efficacy Analyses....93
10.4.5.2. Secondary Efficacy Analyses....93
10.4.5.3. Subgroup Analyses....94
10.4.6. Safety Analyses....94
10.4.6.1. Adverse Events....94
10.4.6.2. Other Safety Analyses....95
10.4.6.3. Treatment Exposure....95
10.4.6.4. Treatment Compliance....95
10.4.7. Analysis of Drug Concentration Data....96
10.4.8. Analysis of Anti-Drug Antibody Data....96
10.4.9. Analysis of Biomarker Data....96
10.5. Multiplicity Considerations....96
10.6. Interim Analysis....97
10.7. Additional Statistical Data Handling Conventions....97
10.8. Statistical Considerations Surrounding the Premature Termination of a Study....98
11. DATA MANAGEMENT AND ELECTRONIC SYSTEMS....98
11.1. Data Management....98
11.2. Electronic Systems....98
12. STUDY MONITORING....99
12.1. Monitoring of Study Sites....99
12.2. Source Document Requirements....99

CONFIDENTIAL

VV-RIM-00023209-1.0 Approved - 29 Aug 2017 GMT-5:00

12.3. Case Report Form Requirements 99

13. AUDITS AND INSPECTIONS 99

14. ETHICAL AND REGULATORY CONSIDERATIONS 100

14.1. Good Clinical Practice Statement 100

14.2. Informed Consent 100

14.3. Patients' Confidentiality and Data Protection 101

14.4. Institutional Review Board/Ethics Committee 101

15. PROTOCOL AMENDMENTS 101

16. PREMATURE TERMINATION OF THE STUDY OR CLOSE-OUT OF A SITE 102

16.1. Premature Termination of the Study 102

16.2. Close-out of a Site 102

17. STUDY DOCUMENTATION 102

17.1. Certification of Accuracy of Data 102

17.2. Retention of Records 102

18. CONFIDENTIALITY 103

19. FINANCING AND INSURANCE 103

20. PUBLICATION POLICY 103

21. REFERENCES 104

22. INVESTIGATOR'S AGREEMENT 110

Signature of Sponsor's Responsible Officers 135

LIST OF TABLES

Table 1: Guidelines for Platinum-Based Doublet Chemotherapy Regimens 48

Table 2: REGN2810 Guidelines for Temporary and Permanent Discontinuations for Toxicity 53

Table 3: General REGN2810 Treatment Hold Guidelines for Immune-Related Adverse Events 56

Table 4: Schedule of Events: Screening Visit Assessments and Procedures 64

Table 5: Schedule of Events: Treatment Period Assessments and Procedures 66

Table 6: Schedule of Events: Follow-Up Period Assessments and Procedures 74

Table 7: Response According to Revised Response Evaluation Criteria in Solid Tumors (Version 1.1) in Patients with Target (and Non-Target) Lesions 117

LIST OF FIGURES

Figure 1: Study Flow Diagram 34
Figure 2: Hierarchical Testing Strategy for Primary and Key Secondary Endpoints 97

LIST OF APPENDICES

Appendix 1. Factors to Consider in Assessing the Relationship of Adverse Events to Study Drug and Study Conduct 111
Appendix 2. Response Evaluation Criteria in Solid Tumors: RECIST Guideline (Version 1.1) 112
Appendix 3. Recommended Dose Modification or Discontinuation and Supportive Care Guidelines for Specific REGN2810 Drug-Related Adverse Events 119
Appendix 4. REGN2810 Pharmacokinetic, Immunogenicity, and Biomarker Sampling Schedule 133

CONFIDENTIAL

VV-RIM-00023209-1.0 Approved - 29 Aug 2017 GMT-5:00

1. INTRODUCTION

Lung cancer is one of the most commonly diagnosed cancers and is the leading cause of cancer-related mortality worldwide (Siegel 2016, Bray 2013). Non-small cell lung cancer (NSCLC) accounts for 80% to 85% of all lung cancers and is composed of several histopathological subtypes, the most common of which include adenocarcinoma (40% to 60%) and squamous cell carcinoma (30%). The majority of patients with NSCLC are found to have advanced cancer at the time of diagnosis (Leighl 2012). With chemotherapy, these patients have a median overall survival (OS) of up to 12 to 18 months and a 5-year survival rate of approximately 18% (Leighl 2012, Siegel 2016).

Systemic therapy with platinum-based doublet chemotherapy regimens, with or without maintenance therapy, has been, until recently, the standard first-line treatment for all patients with advanced NSCLC whose tumors do not have an epidermal growth factor receptor (EGFR) mutation, an anaplastic lymphoma kinase (ALK) translocation, or a C-ros oncogene receptor tyrosine kinase (ROS1) mutation (Besse 2014, Ettinger 2016, Reck 2014). Despite initial therapy with platinum-based doublet chemotherapy regimens, the disease often progresses, and additional treatment options have been limited.

In recent years, immunotherapies have been investigated as potential therapeutic approaches that will improve long-term survival and quality of life (QOL) in patients with advanced NSCLC. A complex cross-talk between cancer cells and the host immune system that can both inhibit and enhance tumor growth has recently been clarified (Vinay 2015). Tumors modulate and evade the host immune response through a number of mechanisms, including formation of an immune-suppressive environment within the tumor. Programmed cell death-1 (PD-1) is a co-receptor expressed on the surface of activated T cells that mediates immunosuppression. The binding of PD-1 to one of its ligands, programmed cell death ligand 1 (PD-L1) or programmed cell death ligand 2 (PD-L2), results in inhibition of a cytotoxic T-cell response. Increased expression of PD-L1 in the tumor microenvironment facilitates escape from the immune-surveillance mechanism (T-cell-induced anti-tumor activity). In contrast, blockade of this interaction results in an enhanced T-cell response with anti-tumor activity.

A number of diagnostic PD-L1 assays have been developed to inform treatment decisions (reviewed in Ratcliffe 2017). The assays demonstrate that there is a broad range of PD-L1 expression levels in tumor cells from NSCLC tumor samples (D'Incecco 2015, Kerr 2015). A high level of expression has been correlated with poor patient prognosis and resistance to standard-of-care (chemotherapy) treatment (Creelan 2014). Blockade of the PD-1/PD-L1 T-cell checkpoint pathway has been shown to be an effective and well-tolerated approach to stimulating the immune response and has achieved significant objective responses in patients with NSCLC (Topalian 2012). Among patients with advanced, previously treated squamous and non-squamous NSCLC, OS was significantly improved in patients treated with the PD-1 inhibitor nivolumab compared to the OS in those treated with docetaxel, regardless of PD-L1 expression (Borghaei 2015, Brahmer 2015). Similarly, patients with previously treated PD-L1-positive NSCLC who were treated with the PD-1 inhibitor pembrolizumab had improved OS compared to those treated with docetaxel (PD-L1 expression in at least 1% of tumor cells) (Herbst 2016). These data established the use of PD-1 inhibitors in the second-line treatment paradigm of patients with NSCLC (KEYTRUDA® [pembrolizumab] Package Insert,

KEYTRUDA European Union [EU] Summary of Product Characteristics [SmPC], OPDIVO® [nivolumab] Package Insert, OPDIVO EU SmPC, Ettinger 2016).

A third PD-L1 inhibitor, TECENTRIQ® (atezolizumab), was recently approved in the US for the treatment of metastatic NSCLC in patients with disease progression during or after treatment with platinum-based chemotherapy. Approval was based on 2 studies that demonstrated improved efficacy over docetaxel (Fehrenbacher 2016, Rittmeyer 2017).

Programmed cell death-1/PD-L1 inhibitors are currently being investigated both as monotherapy (eg, KEYNOTE-024 and CheckMate 026) and in combination with standard-of-care chemotherapy regimens (eg, Rizvi 2016, Antonia 2014, Gadgeel 2016) or other immunotherapies (eg, CheckMate 012 [Hellmann 2017]) in the first-line treatment of patients with advanced NSCLC. Results from clinical studies, described in detail below, have been encouraging, and results of KEYNOTE-024 led to the approval of pembrolizumab as first-line treatment in metastatic NSCLC patients whose tumors express PD-L1 in ≥50% of tumor cells.

In the phase 1 multicohort study, CheckMate 012, 56 patients with advanced squamous or non-squamous, treatment-naïve NSCLC received nivolumab in combination with 4 cycles of platinum-based chemotherapy. Response rates ranged from 33% to 47% with 1-year OS rates of 50% to 87% (Rizvi 2016). In patients treated with a PD-1/PD-L1 inhibitor in combination with chemotherapy, response rates ranging from 33% to 77% and median progression-free survival (PFS) ranging from 4.8 to 10 months have been observed in PD-L1 unselected tumors (Antonia 2014, Papadimitrakopoulou 2015, Liu 2015). In tumors expressing high levels of PD-L1, even greater anti-tumor responses have been observed with response rates of 44% to 75% (Liu 2015, Gadgeel 2016) and a median PFS of 15 months (Gadgeel 2016). These limited data suggest that while monotherapy may be effective in the tumors with high PD-L1 expression, combination chemotherapy may be equally or more effective in patients with any degree of PD-L1 expression.

KEYNOTE-024 was an open-label, phase 3 study that included 305 patients with previously untreated, advanced NSCLC whose tumors expressed PD-L1 in ≥50% of tumor cells. The patients were randomized to receive either pembrolizumab or the investigator's choice of platinum-based chemotherapy. Crossover from the chemotherapy group to the pembrolizumab group was permitted in the event of disease progression. Median PFS was 10.3 months (95% confidence interval [CI]: 6.7 months to not reached) in the pembrolizumab group versus 6.0 months (95% CI: 4.2 to 6.2 months) in the chemotherapy group (hazard ratio [HR] for disease progression or death: 0.50; 95% CI: 0.37 to 0.68; p<0.001). The response rate was higher in the pembrolizumab group than in the chemotherapy group (44.8% versus 27.8%) (Reck 2016). Furthermore, compared to the chemotherapy group, for the pembrolizumab group: the median duration of response (DOR) was longer (not reached [range: 1.9+ to 14.5+ months] versus 6.3 months [range: 2.1+ to 12.6+ months]); and treatment-emergent adverse events (TEAEs) of any grade were less frequent (occurring in 73.4% versus 90.0% of patients), as were TEAEs of grade 3, 4, or 5 (26.6% versus 53.3%) (Reck 2016). The results of this study led to approval of pembrolizumab in the United States (US), Japan, and some European countries for first-line treatment of advanced NSCLC in patients whose tumors express PD-L1 in ≥50% of tumor cells as determined by the PD-L1 immunohistochemistry (IHC) 22C3 pharmDx assay (PD-L1 IHC 22C3 pharmDx Package Insert; Roach 2016). This was the first US Food and Drug Administration (FDA) approval of a checkpoint inhibitor for first-line treatment of lung

cancer. This approval also expanded the pembrolizumab indication in second-line treatment of lung cancer to include all patients with NSCLC expressing PD-L1 ≥1%.

In contrast to KEYNOTE-024, the phase 3 CheckMate 026 study investigating the efficacy of first-line treatment with nivolumab compared to platinum-based doublet chemotherapy in patients with advanced PD-L1-positive NSCLC (defined as present in 1% or more tumor cells) did not meet its primary endpoint (Socinski 2016). Even in a subset analysis of those patients with PD-L1 expression on ≥50% tumor cells, nivolumab failed to demonstrate any improvement over chemotherapy. There is currently no proven explanation for this discrepancy between the two studies. A total of 541 patients were randomized 1:1 to the nivolumab or chemotherapy treatment group. Patients who progressed on chemotherapy could cross over to nivolumab as second-line treatment. In the 423 patients whose tumors express PD-L1 in 5% or greater of tumor cells, PFS was 4.2 months with nivolumab and 5.9 months with chemotherapy (HR: 1.15; 95% CI: 0.91 to 1.45; p=0.25). Overall survival was 14.4 months for nivolumab versus 13.2 months for chemotherapy (HR: 1.02; 95% CI: 0.80 to 1.30).

Programmed cell death-1/PD-L1 inhibitors have also being investigated in combination with standard-of-care chemotherapy regimens. Most recently, results with pembrolizumab in chemotherapy-naïve patients with non-squamous NSCLC in combination with carboplatin and pemetrexed (KEYNOTE-021, Cohort G [n=123]) have shown a median PFS of 10 months and a median OS that was not reached (Langer 2016). Mean confirmed response rates ranged from 54% (PD-L1 expression ≥1% to 49%) to 60% (PD-L1 expression ≥50%) and median PFS from 14 to 15 months. Patients whose tumors expressed PD-L1 <1% showed a median PFS of 6 months, which is historically observed with first-line chemotherapy regimens alone.

A second type of immuno-oncology agent, anti-cytotoxic T-lymphocyte-associated antigen 4 (anti-CTLA-4), has also been demonstrated to be clinically active in advanced cancers. Ipilimumab, a fully human monoclonal anti-CTLA-4 agent, has demonstrated clinical activity as monotherapy in melanoma (Wolchok 2010, O’Day 2010) and prostate cancer (Slovin 2013) and is now approved in the US and European Union (EU) for treatment of unresectable or metastatic melanoma and for the adjuvant treatment of melanoma (Yervoy Package Insert, Yervoy SmPC).

In addition to immuno-oncology agents as monotherapy, the potentially additive or synergistic effects of immuno-oncology therapeutics with different mechanisms of action are now being evaluated in NSCLC (reviewed in Buchbinder 2016). Nivolumab as combination immunotherapy with ipilimumab was evaluated in CheckMate 012, a phase 1 study in treatment-naïve patients with advanced NSCLC (Hellmann 2017). Patients received nivolumab plus ipilimumab at 1 of 3 dose regimens. Confirmed response rates were 47% (95% CI: 31% to 64%) in patients receiving nivolumab every 2 weeks (Q2W) plus ipilimumab every 12 weeks (Q12W) and 38% (95% CI: 23% to 55%) in patients receiving nivolumab Q2W plus ipilimumab every 6 weeks (Q6W). The median DOR was not reached in either treatment group (median follow-up times of 12.8 months in the patients receiving nivolumab Q2W plus ipilimumab Q12W and 11.8 months in the patients receiving nivolumab Q2W plus ipilimumab Q6W). The greatest percentage of responses was noted in patients with tumors that expressed PD-L1. In patients whose tumors expressed PD-L1 in ≥1% of tumor cells, confirmed objective responses were achieved in 12 of 21 (57%) patients in the ipilimumab Q12W treatment group and 13 of 23 (57%) patients in the ipilimumab Q6W treatment group (Hellmann 2017). The 1-year survival rate in patients treated with nivolumab 3 mg/kg Q2W plus ipilimumab 1 mg/kg Q6W or

Q12W was 100% in patients with tumors that expressed PD-L1 in ≥50% of tumor cells (n=13) and was 76% in all-comers (all patients regardless of PD-L1 status [n=77]), compared to 73% for patients receiving nivolumab monotherapy (n=52). One-year survival in patients with tumors that expressed PD-L1 in >1% of tumor cells was 91% in the nivolumab 3 mg/kg plus ipilimumab 1 mg/kg Q12W treatment group and 83% in the nivolumab 3 mg/kg plus ipilimumab 1 mg/kg Q6W treatment group (n=23 in each treatment group) compared to 73% in the nivolumab monotherapy group (n=32) (Gettinger 2016). The improved efficacy in NSCLC patients whose tumors expressed PD-L1 contrasts to the phase 3 study in patients with melanoma (Larkin 2015), where the addition of anti-CTLA-4 to anti-programmed death-1 (anti-PD-1) provided benefit predominantly to those patients whose tumors had low baseline PD-L1 expression.

Combination immunotherapy with durvalumab (anti-programmed death ligand 1 [anti-PD-L1]) plus tremelimumab (anti-CTLA-4) is currently being evaluated in multiple cancer types, including urothelial carcinoma (NCT02516241), squamous cell carcinoma of the head and neck (NCT02551159), and renal cell carcinoma (NCT02762006). In patients with NSCLC, the durvalumab plus tremelimumab combination demonstrated clinical activity, regardless of PD-L1 expression levels. Investigator-reported confirmed responses occurred in 23% of patients (95% CI: 9% to 44%) in the durvalumab plus tremelimumab combination therapy group, including 22% (95% CI: 3% to 60%) of patients with PD-L1-positive tumors (tumors that express PD-L1 on ≥25% of tumor cells) and 29% (95% CI: 8% to 58%) of patients with PD-L1-negative tumors (tumors that express PD-L1 on <25% of tumor cells) (Antonia 2016). Recently, however, a phase 3 trial of durvalumab in combination with tremelimumab failed to significantly improve PFS over standard-of-care chemotherapy in PD-L1-positive patients with advanced NSCLC (AstraZeneca Press Release, 27 July 2017).

Ipilimumab plus chemotherapy also demonstrated improved median OS over chemotherapy alone (12.2 versus 8.3 months; p=0.23) in previously untreated patients with NSCLC when chemotherapy was given first followed by ipilimumab ("phased ipilimumab"). Median immune-related PFS, the primary endpoint, was significantly improved in patients receiving phased ipilimumab over chemotherapy alone (5.7 versus 4.6 months; p=0.05) (Lynch 2012).

REGN2810 is a high-affinity, fully human, hinge-stabilized IgG4P antibody directed to the PD-1 receptor that potently blocks the interaction of PD-1 with its ligands, PD-L1 and PD-L2. Additional studies underway with REGN2810 are described in the Investigator's Brochure. Pre-clinical data indicate that REGN2810 has a similar efficacy to other approved anti-PD-1 antibodies; clinical data to date confirm the pre-clinical data. Regeneron Pharmaceuticals, Inc. (Regeneron) is developing REGN2810 as a foundational immuno-oncology agent to be combined with maximal flexibility with other anti-cancer immunotherapies currently in pre-clinical development.

Please refer to the latest edition of the Investigator's Brochure for an overview of relevant safety data (including patient exposure and immune-related adverse events [irAEs]) and efficacy data from clinical studies with REGN2810.

Study R2810-ONC-16113 is a randomized, open-label, phase 3 study of REGN2810 plus standard-of-care platinum-based doublet chemotherapy (REGN2810/chemo-f) or REGN2810 plus 2 cycles of initial standard-of-care platinum-based doublet chemotherapy plus ipilimumab combination therapies (REGN2810/chemo-l/ipi) versus standard-of-care platinum-based doublet chemotherapy for the treatment of patients with advanced NSCLC whose tumors express PD-L1

VV-RIM-00023209-1.0 Approved - 29 Aug 2017 GMT-5:00

in <50% of tumor cells and who have received no prior systemic treatment for their advanced disease. The main objective of the study is to determine if REGN2810/chemo-f or REGN2810/chemo-l/ipi improves PFS over standard-of-care platinum-based doublet chemotherapy in this patient population. Additional objectives include characterization of OS, objective response rate (ORR), safety, pharmacokinetics (PK), and QOL.

2. STUDY OBJECTIVES

2.1. Primary Objective

The primary objective of the study is to compare the PFS of REGN2810/chemo-f and REGN2810/chemo-l/ipi with standard-of-care platinum-based doublet chemotherapy in the first-line treatment of patients with advanced squamous or non-squamous NSCLC in the subgroup of patients whose tumors express PD-L1 in 1% to <50% of tumor cells and in the overall population of study patients whose tumors express PD-L1 in <50% of tumor cells.

2.2. Secondary Objectives

2.2.1. Key Secondary Objectives

The key secondary objectives of the study are the following:

- To compare the OS of REGN2810/chemo-f and REGN2810/chemo-l/ipi versus standard-of-care platinum-based doublet chemotherapy in the first-line treatment of patients with advanced squamous or non-squamous NSCLC in the subgroup of patients whose tumors express PD-L1 in 1% to <50% of tumor cells and in the overall population of study patients whose tumors express PD-L1 in <50% of tumor cells
- To compare the ORR of REGN2810/chemo-f and REGN2810/chemo-l/ipi versus standard-of-care platinum-based doublet chemotherapy in the first-line treatment of patients with advanced squamous or non-squamous NSCLC in the subgroup of patients whose tumors express PD-L1 in 1% to <50% of tumor cells and in the overall population of study patients whose tumors express PD-L1 in <50% of tumor cells

2.2.2. Other Secondary Objectives

The other secondary objectives are the following:

- To evaluate the safety and tolerability of REGN2810/chemo-f and REGN2810/chemo-l/ipi compared to standard-of-care platinum-based doublet chemotherapy
- To compare the OS at 12 and 18 months of REGN2810/chemo-f and REGN2810/chemo-l/ipi versus standard-of-care platinum-based doublet chemotherapy in the first-line treatment of patients with advanced squamous or non-squamous NSCLC in the subgroup of patients whose tumors express PD-L1 in 1% to <50% of tumor cells and in the overall population of study patients whose tumors express PD-L1 in <50% of tumor cells

VV-RIM-00023209-1.0 Approved - 29 Aug 2017 GMT-5:00

- To compare QOL in patients with advanced squamous or non-squamous NSCLC whose tumors express PD-L1 in <50% of tumor cells receiving REGN2810/chemo-f or REGN2810/chemo-l/ipi versus those receiving standard-of-care platinum-based doublet chemotherapy
- To assess immunogenicity as measured by anti-drug antibodies (ADAs) for REGN2810
- To assess the predictive utility of baseline PD-L1 tumor expression levels on indicators of clinical response using PD-L1 assays other than the clinical trial assay
- To characterize the PK of REGN2810 when administered in combination with ipilimumab or in combination with standard-of-care platinum-based doublet chemotherapy
- To conduct exposure-response (E-R) analyses for relevant biomarkers (exploratory PK/pharmacodynamic analyses) and E-R analyses for safety and efficacy endpoints, as appropriate

3. HYPOTHESIS AND RATIONALE

3.1. Hypothesis

The primary hypotheses of this study are that REGN2810/chemo-f or REGN2810/chemo-l/ipi will prolong PFS compared to standard-of-care platinum-based doublet chemotherapy in the subgroup of patients whose tumors express PD-L1 in 1% to <50% of tumor cells and in the overall population of study patients whose tumors express PD-L1 in <50% of tumor cells. The secondary hypotheses are that REGN2810/chemo-f or REGN2810/chemo-l/ipi will improve OS in the subgroup of patients whose tumors express PD-L1 in 1% to <50% of tumor cells and in the overall population of study patients whose tumors express PD-L1 in <50% of tumor cells.

Historically, median PFS in patients with stage IIIB/IV squamous and non-squamous NSCLC treated with platinum-based doublet chemotherapy has ranged from approximately 2.7 to 6.4 months (El-Shenshawy 2012, Kelly 2001, Rosell 2002, Scagliotti 2002, Schiller 2002, Shimizu 2013, Reck 2016). With the emergence of immunotherapy and the recognition that NSCLC tumors express PD-L1, the effects of a variety of PD-1/PD-L1 inhibitors as monotherapy and in combination with chemotherapy are being investigated. Accumulating clinical data suggest that anti-PD-1 monotherapy may prolong PFS and OS in NSCLC, with the greatest clinical benefit observed in tumors expressing PD-L1, especially at high levels.

Anti-PD-1 monotherapy as first-line treatment in patients with advanced NSCLC has demonstrated efficacy (Gettinger 2016, Garon 2015) and a significant improvement in PFS compared to the chemotherapy (Reck 2016). Combination immunotherapies for NSCLC have also demonstrated clinical activity (Hellmann 2017).

REGN2810 clinical study data (Section 1) in addition to the known activity of standard-of-care platinum-based doublet chemotherapy regimens and the demonstrated clinical activity of ipilimumab support the hypothesis that REGN2810/chemo-l/ipi and REGN2810/chemo-f may

VV-RIM-00023209-1.0 Approved - 29 Aug 2017 GMT-5:00

prolong PFS in patients with advanced or metastatic, squamous or non-squamous NSCLC whose tumors express in <50% of tumor cells.

3.2. Rationale

3.2.1. Rationale for Study Design

This study is a randomized, global, open-label, phase 3 study comparing REGN2810/chemo-f or REGN2810/chemo-l/ipi to standard-of-care platinum-based doublet chemotherapy in the first-line treatment of patients with advanced or metastatic, squamous or non-squamous NSCLC whose tumors express PD-L1 in <50% of tumor cells and who have received no prior systemic treatment for their advanced disease. This study will be open-label because the differences in administration and known distinct toxicities of the therapies do not lend themselves to blinding.

Because this is the first study to evaluate the combination of REGN2810 and ipilimumab, there will be an early review of data to ensure patient safety after the first 10 patients in the REGN2810/chemo-l/ipi treatment arm have completed 4 weeks of follow-up following the first dose of REGN2810/chemo-l/ipi.

Additionally, pemetrexed maintenance therapy will be optional for patients in the standard-of-care platinum-based doublet chemotherapy arm randomized to a regimen containing pemetrexed, whereas no maintenance option will be given to patients in the REGN2810 plus platinum-based doublet chemotherapy combination therapy treatment arm. Imposing a double-blind design would require patients in this treatment arm to receive placebo until Response Evaluation Criteria in Solid Tumors version 1.1 (RECIST 1.1)-defined progressive disease, and this was not viewed as acceptable given the nature of the disease.

3.2.2. Rationale for Endpoints and Objectives

The primary objective of this study is to compare the PFS of REGN2810/chemo-f or REN2810/chemo-l/ipi to standard-of-care platinum-based doublet chemotherapy in the subgroup of patients whose tumors express PD-L1 in 1% to <50% of tumor cells and in the overall population of study patients whose tumors express PD-L1 in <50% of tumor cells. Overall survival is clearly the ultimate endpoint of REGN2810 combination therapy benefit, but an improvement in OS may be obscured due to patients who progress and elect to begin second-line treatment with pembrolizumab, nivolumab, or atezolizumab as recommended in the recent National Comprehensive Cancer Network recommendations (Ettinger 2016). Therefore, OS will be a secondary endpoint. This approach is consistent with the 2015 FDA Guidance for Industry on Clinical Trial Endpoints for the Approval of NSCLC Drugs and Biologics (FDA 2015) and the December 2015 European Medical Agency Guideline on the Evaluation of Anticancer Medicinal Products in Man (EMA 2015), which contains specific guidance on NSCLC.

Progression-free survival, defined as the time to tumor progression (based on RECIST 1.1 criteria [Eisenhauer 2009]) or death, was chosen as the primary endpoint because PFS is recognized as a marker of clinical benefit. Progression-free survival is an approvable endpoint in the US and is also acceptable in the EU for studies where the experimental therapy is likely to be well tolerated. Progressive disease will be determined based on the RECIST 1.1 criteria (Eisenhauer 2009). The first radiographic tumor assessment will occur after 9 weeks of study treatment and subsequent assessments will occur every 9 weeks (Q9W) during year 1, Q12W

VV-RIM-00023209-1.0 Approved - 29 Aug 2017 GMT-5:00

during year 2, and Q12W during follow-up or until Independent Review Committee (IRC)-assessed RECIST 1.1-defined progressive disease or early treatment discontinuation for another reason. Further, to diminish bias in the assessment of disease progression in this open-label study, an IRC, which will be blinded to treatment assignment, will be utilized to adjudicate tumor responses. Overall survival at 12 months, 18 months, and end of treatment will be assessed as secondary endpoints, despite the acknowledged limitations. Additionally, efficacy responses to be assessed will include assessment of ORR. From a patient perspective, preservation of QOL is important; therefore, QOL will be assessed through the use of 2 validated questionnaires.

3.2.3. Rationale for Choice of Patient Population Based on the <50% PD-L1 Expression Cut-off

Data suggest that tumor cell PD-L1 expression may correlate with anti-PD-1 clinical activity in both squamous and non-squamous NSCLC (Gadgeel 2016, Gettinger 2015, Hellmann 2017) and that high levels of PD-L1 expression were previously correlated with poor patient prognosis and resistance to treatment in NSCLC (Creelan 2014).

Anti-PD-L1 monotherapy studies have been conducted in patients with NSCLC. For example, in an exploratory analysis of an ongoing study, nivolumab monotherapy in chemotherapy-naïve patients with advanced NSCLC resulted in higher response rates and more prolonged PFS and 1-year OS rates as the level of PD-L1 expression increased (1%, 5%, 10%, 25%, and 50%) (Gettinger 2015). A study of pembrolizumab monotherapy (KEYNOTE-021) in patients with NSCLC showed a higher response rate and longer PFS and OS rates in patients expressing ≥50% PD-L1 than in untreated and previously treated patients expressing <50% PD-L1 (Garon 2015). Pembrolizumab is also approved in the US for first-line therapy in patients expressing ≥50% PD-L1. A phase 1 study of nivolumab monotherapy or nivolumab combined with ipilimumab in patients with advanced NSCLC (CheckMate 012) demonstrated the greatest benefit in patients expressing PD-L1 ≥50% with an ORR of 92% (Hellmann 2017, Gettinger2016). A phase 3 study (CheckMate 026) investigating the efficacy of first-line treatment with nivolumab monotherapy compared to platinum-based doublet chemotherapy in patients with advanced NSCLC and PD-L1-positive tumors failed to meet its primary endpoint of PFS. Even in a subset analysis of those patients with PD-L1 expression in ≥50% of tumor cells, nivolumab failed to demonstrate any improvement over chemotherapy (Socinski 2016).

Generally, anti-PD-1 monotherapy has been shown to be efficacious in NSCLC patients whose tumors express PD-L1 in ≥50% of tumor cells but not in patients whose tumors express PD-L1 in <50% of tumor cells. In order to treat patients whose tumors express lower levels of PD-L1, a combination immunotherapy regimen will be required; thus, patients whose tumors express PD-L1 in <50% of tumor cells (as determined by the PD-L1 IHC 22C3 pharmDx assay) are the focus of this study. Additionally, a non-validated 25% cutoff will be used for exploratory stratification and analyses.

3.2.4. Rationale for Combination of REGN2810 and Ipilimumab and Limited (2 Cycles) Platinum-Based Doublet Chemotherapy

Combination immunotherapies that include an anti-CTLA-4 agent and an anti-PD-1/PD-L1 agent have the potential for additive or synergistic effects (reviewed in Buchbinder 2016).

Clinical activity of the combination of anti-PD-L1 and anti-CTLA-4 has been demonstrated in studies of durvalumab plus tremelimumab (Antonia 2016; Section 1) and nivolumab plus ipilimumab (described in detail below).

Ipilimumab has shown promise in multiple tumor types and is approved as monotherapy and in combination with nivolumab for advanced melanoma. Ipilimumab plus nivolumab combination therapy was approved for the treatment of advanced melanoma based on the results of CheckMate 067 that demonstrated significantly improved PFS in patients treated with combination therapy (11.5 months; 95% CI: 8.9 to 16.7 months) compared with ipilimumab monotherapy (2.9 months; 95% CI: 2.8 to 3.4 months; HR for death or disease progression, 0.42; 99.5% CI: 0.31 to 0.57; p<0.001) and nivolumab monotherapy (6.9 months; 95% CI: 4.3 to 9.5 months; HR for the comparison with ipilimumab, 0.57; 99.5% CI: 0.43 to 0.76; p<0.001) (Larkin 2015).

Ipilimumab plus nivolumab combination therapy has also demonstrated clinical activity in NSCLC (Section 1) as first-line treatment in advanced NSCLC. In the CheckMate 012 study, confirmed response rates were 47% (95% CI: 31% to 64%) in patients receiving nivolumab Q2W plus ipilimumab Q12W and 38% (95% CI: 23% to 55%) in patients receiving nivolumab Q2W plus ipilimumab Q6W. The greatest percentage of responses was noted in patients with tumors that expressed PD-L1 (Hellmann 2017), which is in contrast with the phase 3 study in melanoma (Larkin 2015), in which the addition of an anti-CTLA-4 agent to an anti-PD-1 agent provided a benefit predominantly in patients with low baseline PD-L1 positivity. The 1-year survival rate in patients with tumors that expressed PD-L1 in ≥50% of tumor cells (n=13) was 100% and in all-comers (n=77) was 76%, compared to 73% for patients receiving nivolumab monotherapy (n=52) (Gettinger 2016; see Section 1 for additional details).

Recently, unpublished data have indicated that conducting 2 preliminary cycles of standard-of-care platinum-based doublet chemotherapy before the introduction of a secondary anti-CTLA-4 and/or anti-PD-1/PD-L1 agent may improve the overall efficacy and, therefore, this regimen has also been adopted in this study.

REGN2810 and ipilimumab are immuno-oncology agents with different mechanisms of action. REGN2810 is a monoclonal antibody to the PD-1 receptor that blocks PD-1/PD-L1-mediated T-cell inhibition, whereas ipilimumab is an inhibitor of CTLA-4. Based on the unique mechanisms of action, the anti-PD-1/anti-PD-L1 action of REGN2810 and the anti-CTLA-4 action of ipilimumab have the potential for additive or synergistic effects (Buchbinder 2016).

Blockade of the PD-1/PD-L1 T-cell checkpoint pathway is an effective and well tolerated approach to stimulating the immune response, and has achieved significant objective responses in advanced melanoma, RCC, and NSCLC (Topalian 2012). However, optimal therapy will likely require combining immuno-oncology agents with conventional therapies and/or novel immunotherapy approaches. Combinatorial approaches of immuno-oncology agents plus chemotherapy have demonstrated encouraging results in NSCLC (reviewed in Gainor 2016). For example, CheckMate 012, described in Section 1, evaluated nivolumab in combination with 4 cycles of platinum-based doublet chemotherapy. Response rates ranged from 33% to 47% with 1-year OS rates of 50% to 87% (Rizvi 2016). Evaluation of the combination of REGN2810 plus platinum-based doublet chemotherapy will allow comparison of the safety and potential benefits in the population of NSCLC patients whose tumors express PD-L1 in <50% of tumor cells.

The recent trial of durvalumab in combination with tremelimumab failed to achieve statistically significant improvement in PFS over standard-of-care chemotherapy in PD-L1-positive patients with advanced NSCLC (AstraZeneca Press Release, 27 July 2017). This raises some concern whether the initial promising results of the combination of nivolumab and ipilimumab seen in CM-012 (Hellmann 2017) will bear out in larger trials. The time required to mobilize an effective anti-tumor response may sometimes take weeks to months, and in the setting of a rapidly growing tumor, may not arise before progression has occurred. As the recent data studying various combinations of immunotherapy and chemotherapy have shown, concomitant chemotherapy may not appreciably interfere with immunotherapy benefit. Therefore, in this study the combination of REGN2810 and ipilimumab will be accompanied by administration of the first 2 cycles of standard platinum doublet chemotherapy (on an every 3 weeks [Q3W] schedule). The rationale is that initial tumor control mediated by chemotherapy will provide more time for an effective immune response to mature, and may, in fact, be enhanced by chemotherapy-induced cell death and augmented antigen presentation. The rationale for limiting the chemotherapy to 2 cycles is to limit the risk of triple combination-related toxicities and to limit the interference of longer administration of the cytotoxic regimens with the immuno-oncology combination therapy.

3.2.5. Rationale for REGN2810 Dose Selection

The proposed REGN2810 dose of 350 mg Q3W given by intravenous (IV) route was selected in this study for patients with NSCLC to better align with the dosing schedule of planned concurrent therapies, namely with ipilimumab (50 mg Q6W for 4 doses) and with platinum-based doublet chemotherapy (day 1 or days 1 and 8, Q3W). The REGN2810 dose of 350 mg Q3W was also selected for an ongoing pivotal phase 3 study in patients with NSCLC.

In the ongoing first-in-human (FIH) study (study R2810-ONC-1423) the 3 mg/kg Q2W IV dose has shown anti-tumor activity and acceptable safety in NSCLC patients among other patient types; efficacy was also observed at the 1 mg/kg Q2W dose. As many standard chemotherapy treatments for NSCLC are dosed on a Q3W schedule, the clinical development strategy of REGN2810 coalesced around a Q3W treatment interval. The ongoing clinical development of REGN2810 also seeks to incorporate a flat dosing paradigm. As such, a Q3W flat dose regimen has been selected that is expected to provide a similar clinical efficacy and safety profile to that observed for the 3 mg/kg Q2W regimen.

The preference of a flat dose over a body weight adjusted dose for anti-PD-1 monoclonal antibodies is supported by a wide safety margin (no maximum tolerated dose [MTD] observed), a flat E-R relationship for safety and efficacy over the therapeutic dosing range, and similar variability in exposure (CV%) after flat and body weight adjusted dose (Freshwater 2017, Zhao 2017a). In fact, pembrolizumab and nivolumab were initially approved at a body-weight adjusted dose of 2 mg/kg Q3W and 3 mg/kg Q2W, respectively, and were recently approved by US FDA at flat doses of 200 mg Q3W and 240 mg Q2W, respectively, for the treatment of melanoma and NSCLC (KEYTRUDA Package Insert, OPDIVO Package Insert).

A flat IV REGN2810 dose of 350 mg Q3W was selected, based on population PK modeling and simulation since this dose is expected to provide exposure that closely replicates the exposure observed in patients (mean weight: 80 kg) for the 3 mg/kg Q2W IV regimen in

study R2810-ONC-1423 (NCT02383212). Simulations of REGN2810 exposure in 1000 patients using population PK analyses have demonstrated the following:

1. The variability in REGN2810 exposure was similar for body weight-adjusted doses as compared to flat doses, therefore supporting flat dose selection.
2. A 350 mg Q3W dose in patients with an expected mean body weight of 80 kg resulted in similar (≤20% difference) C_{trough}, area under the curve from time 0 to week 12 (AUC_{12W}), and peak concentration (C_{max}) as compared to the 3 mg/kg Q2W dose used in the FIH study. REGN2810 concentrations with 350 mg Q3W exceeded those observed with 1 mg/kg Q2W, a dose that demonstrated clinical efficacy in the FIH study, and C_{trough} values with 350 mg Q3W exceeded concentrations of ~5 to 20 mg/L, above which saturation of PD-1 target occupancy is expected to occur based on linearity assessment on single-dose pharmacokinetics in cynomolgus monkeys.

The 350 mg Q3W dose of REGN2810 is, therefore, being proposed as the optimal dose in the phase 3 studies in patients with NSCLC and across the REGN2810 program.

There are some populations (eg, in Japan or other countries in Asia-Pacific Rim where, on average, the body weight in the population is slightly lower as compared to Western patients (with body weight distribution around a mean of 60 kg versus 80 kg) (Shimizu 2016), and body weight is a known covariate of exposure for monoclonal antibodies. The average body weight of the patient population in the FIH study R2810-ONC-1423 was approximately 80 kg. As supported by population PK modeling and simulation of REGN2810 exposure in 1000 patients, when REGN2810 is administered as a flat dose, a small (approximately 16%) but clinically unimportant increase in exposure of REGN2810 in serum is predicted on average in Asian and/or Japanese patients versus the existing data in Western patients. With the lack of any added safety signal in the existing clinical data at doses up to 10 mg/kg, the existing data support the use of the 350 mg Q3W treatment regimen for the global development of REGN2810.

3.2.6. Rationale for Ipilimumab Dose Selection

Recent data presented by Bristol-Meyers Squibb, Inc. demonstrated higher tumor shrinkage in patients treated with nivolumab at 3 mg/kg Q2W plus ipilimumab at 1 mg/kg Q6W or Q12W compared to nivolumab monotherapy (Zhao 2017b). The incidence of AEs was similar for nivolumab monotherapy and nivolumab plus ipilimumab at 1 mg/kg Q6W or Q12W, but was higher in treatment groups with more frequent and/or higher ipilimumab dosing. Based on the presented risk-benefit assessment (Zhao 2017b) and exposure-response analyses, the recommended dose of ipilimumab in NSCLC is 1 mg/kg Q6W, which is equivalent to approximately 50 mg, the dose proposed in the present study.

3.2.7. Rationale for Standard-of-Care Platinum-Based Chemotherapy as Comparator

Platinum-based doublet chemotherapy is currently recommended as first-line treatment for advanced or metastatic NSCLC in patients with PD-L1 expression <50% (Ettinger 2016) and will therefore serve as the active comparator in this study. There is no single "best" standard platinum-based doublet chemotherapy for squamous or non-squamous NSCLC. Randomized studies that have compared various regimens have not shown any difference in survival (Fossella 2003, Scagliotti 2002, Schiller 2002). Pemetrexed-based doublets are restricted to non-

CONFIDENTIAL

VV-RIM-00023209-1.0 Approved - 29 Aug 2017 GMT-5:00

squamous NSCLC (ALIMTA® US Package Insert, ALIMTA European Union Summary of Product Characteristics).

The PARAMOUNT study is a recent large study of pemetrexed in advanced non-squamous NSCLC (Paz-Ares 2013). In this study, 939 patients with advanced non-squamous NSCLC were given 4 cycles of pemetrexed-cisplatin induction therapy. Of these patients, 539 patients with no disease progression and Eastern Cooperative Oncology Group (ECOG) performance statuses of 0 or 1 were then randomly assigned in a 2:1 ratio to receive maintenance pemetrexed (500 mg/m^2 on day 1 of each 21-day cycle; n=359) or placebo (n=180). The results demonstrated that maintenance pemetrexed resulted in a statistically significant 22% reduction in the risk of death (HR: 0.78; 95% CI: 0.64 to 0.96; p=0.0195; median OS: pemetrexed: 13.9 months, placebo: 11.0 months). Survival on pemetrexed consistently improved for all patient subgroups, including the induction response subgroups: complete/partial responders (n=234) OS HR: 0.81; 95% CI: 0.59 to 1.11; SD (n=285) OS HR: 0.76; 95% CI: 0.57 to 1.01. However, drug-related grade 3 to 4 toxicities of anemia, fatigue, and neutropenia were significantly higher in pemetrexed-treated patients. As such, the investigator in this study will decide, in consultation with an eligible patient, whether or not to include pemetrexed maintenance in the treatment regimen. A decision not to include pemetrexed maintenance will be documented in the case report form (CRF) (throughout this protocol, CRF refers to either a paper CRF or an electronic CRF [eCRF]).

Both cisplatin and carboplatin are used as standard-of-care chemotherapy agents, although carboplatin may be associated with fewer side effects. Given this and that clinical practice preferences will vary in this global study, investigators will be given the option to choose from several approved treatment regimens.

Administration of 4 cycles of chemotherapy is standard, but up to 6 cycles may be given to patients who are not progressing and who are tolerating treatment. For this study, patients will be administered chemotherapy for at least 4 cycles and up to 6 cycles, depending on patient tolerability and disease assessment.

4. STUDY VARIABLES

4.1. Demographic and Baseline Characteristics

Baseline characteristics will include standard demography (eg, age, race, ethnicity, weight, gender, and height) and disease characteristics, including PD-L1 status and medical and oncology history.

4.2. Primary and Secondary Endpoints

4.2.1. Primary Endpoint

The primary endpoint is PFS as assessed by a blinded IRC based on RECIST 1.1 assessments. Progression-free survival will be defined as the time from randomization to the date of the first documented tumor progression, as determined by the IRC (based on RECIST 1.1 assessments [Eisenhauer 2009; see Appendix 2]), or death due to any cause. Patients will be censored according to the rules listed below:

1. Patients who do not have a documented tumor progression or death will be censored on the date of their last evaluable tumor assessment.
2. Patients who do not have any evaluable tumor assessments after randomization and did not die will be censored on the date of randomization.

Rationale for PFS as the primary endpoint is provided in Section 3.2.2.

4.2.2. Key Secondary Endpoints

The key secondary endpoints in the study will be OS and ORR.

Overall survival will be defined as the time from randomization to the date of death. A patient who has not died will be censored at the last known date of contact.

Objective response rate will be defined as the number of patients with a best overall response (BOR) of confirmed CR or PR divided by the number of patients in the efficacy analysis set.

Best overall response will be defined as the BOR, as determined by the IRC per RECIST 1.1, between the date of randomization and the date of the first objectively documented progression or the date of subsequent anti-cancer therapy, whichever comes first.

4.2.3. Other Secondary Endpoints

Other secondary endpoints will include the following:

- The safety and tolerability of REGN2810/chemo-l/ipi and REGN2810/chemo-f measured by the incidence of TEAEs, dose-limiting toxicities (DLTs), serious adverse events (SAEs), deaths, and laboratory abnormalities
- Overall survival at 12 months, 18 months, and end of treatment
- Quality of life as measured by the European Organization for Research and Treatment of Cancer Quality of Life Questionnaire Core 30 (EORTC QLQ-C30) and Quality of Life Questionnaire Lung Cancer 13 (EORTC QLQ-LC13)

See Section 4.4 for immunogenicity variables.

4.3. Pharmacokinetic Variables

REGN2810 concentration in the sera of patients randomized to the REGN2810/chemo-f or REGN2810/chemo-l/ipi treatment arms (Treatment Arms B and C, respectively) will be assessed at multiple time points throughout the treatment and follow-up periods.

Pharmacokinetic variables may include, but are not limited to, the following:

- C_{eoi} – concentration at end of infusion
- C_{trough} – pre-infusion concentration
- t_{eoi} – time of end of infusion

4.4. Anti-Drug Antibody Variables

The ADA variables will be measured in samples from patients randomized to the REGN2810/chemo-f or REGN2810/chemo-l/ipi treatment arms (Treatment Arms B and C, respectively). Anti-drug antibody variables include status of ADA response and titer as follows:

- Treatment-boosted ADA response - defined as a positive response in the ADA assay after the first dose that is greater than or equal to 9-fold over baseline titer levels, when baseline results are positive
- Treatment-emergent ADA response - defined as a positive response in the ADA assay after the first dose when baseline results are negative or missing

The treatment-emergent ADA responses will be further categorized into persistent, indeterminate, and transient responses.

- Titer category is defined based on values as (titer value category):
  - Low (titer <1000)
  - Moderate (1000≤ titer ≤10,000)
  - High (titer >10,000)
- Neutralizing anti-drug antibody (NAb) activities for samples that are confirmed positive in the ADA assay

5. STUDY DESIGN

5.1. Study Description and Duration

This is a phase 3, randomized, global, open-label, efficacy and safety study of REGN2810/chemo-f versus REGN2810/chemo-l/ipi versus standard-of-care platinum-based doublet chemotherapy in the first-line treatment of patients with advanced squamous or non-squamous NSCLC, whose tumors express PD-L1 in <50% of tumor cells (measured using the PD-L1 IHC 22C3 pharmDx assay) and who have received no prior systemic treatment for their advanced disease. A study flow diagram is presented in Figure 1.

VV-RIM-00023209-1.0 Approved - 29 Aug 2017 GMT-5:00

Figure 1: Study Flow Diagram

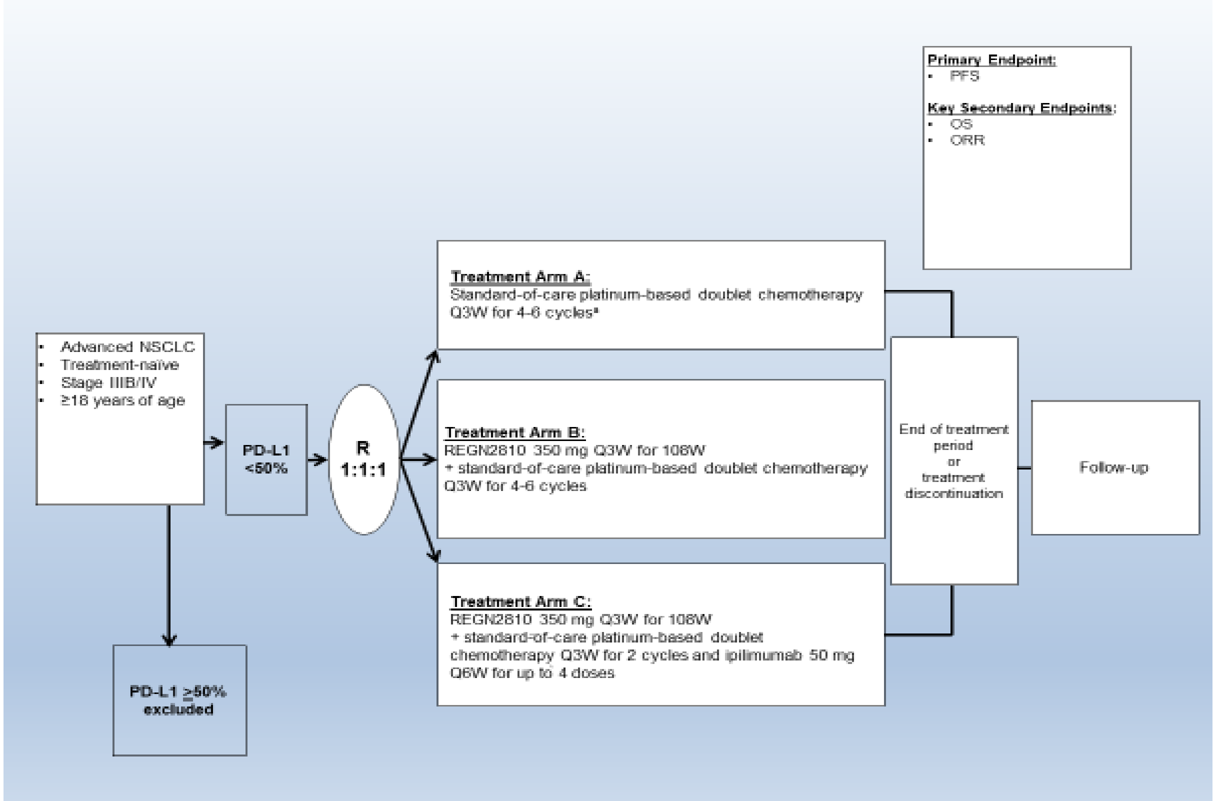

Abbreviations: NSCLC=non-small cell lung cancer; ORR=objective response rate; OS=overall survival; PD-L1=programmed death ligand 1; PFS=progression-free survival; Q3W=every 3 weeks; Q6W=every 6 weeks; R=randomized; W=weeks

[a] For Treatment Arm A, patients with non-squamous NSCLC who are randomized to receive pemetrexed may continue with optional pemetrexed maintenance.

The study will consist of the following 3 periods: screening, treatment, and follow-up. After screening, eligible patients will be randomized to Treatment Arm A (standard-of-care platinum-based chemotherapy), Treatment Arm B (REGN2810/chemo-f), or Treatment Arm C (REGN2810/chemo-l/ipi). The length of the treatment period will determined by the treatment arm to which a patient is randomized. Treatment may be discontinued early due to RECIST 1.1-defined progressive disease, withdrawal of consent, death, unacceptable toxicity, initiation of another anti-cancer treatment, or, for patients in Treatment Arms B and C, in specific instances of confirmed CR or PR (see below). After discontinuing study treatment, patients will enter the follow-up period.

The approximate duration of the active study assessments for each patient, excluding screening, will be 24 to 32 months. For Treatment Arms B and C, this encompasses 25 months of study treatment plus 7 months of follow-up. For patients in Treatment Arm A, this encompasses 4 to 6 cycles of treatment (and pemetrexed maintenance, if appropriate) and radiographic tumor assessments for up to 2 years. After the active portion of the study is complete, all patients will be followed for survival.

VV-RIM-00023209-1.0 Approved - 29 Aug 2017 GMT-5:00

5.1.1. Screening

Patients will undergo a screening evaluation to determine their eligibility within 28 days prior to randomization (Table 4). Program cell death ligand 1 expression in a tumor tissue sample (archival tissue, if ≤5 months old, or recently obtained on-study biopsy collected during screening; for collection instructions, refer to the laboratory manual) will be assessed using the PD-L1 IHC 22C3 pharmDx assay by a central laboratory (Section 8.2.5). Patients whose tumors express PD-L1 in <50% of tumor cells will continue in screening, while those whose tumors express PD-L1 in ≥50% of tumor cells will be excluded from the study. Given that non-squamous (specifically adenocarcinoma) histology is more prevalent than squamous histology, it is predicted that approximately 70% of patients enrolling in the study will have non-squamous NSCLC and 30% of patients will have squamous NSCLC. Tumor tissue samples will also be tested for EGFR mutations and ALK translocations as well as for ROS1 fusions, unless this testing has already been performed and the results are available. Patients whose tumors are positive for any of these mutations/fusions (by testing at screening or by previous results) will not be eligible for the study.

Baseline radiographic tumor assessments should also be performed within 28 days prior to randomization (Table 4). These assessments will not be reviewed by the IRC for eligibility assessment.

Informed consent must be obtained prior to any study-related procedures. Assessments performed as part of standard-of-care that fall within the screening window (28 days prior to randomization) but before informed consent is obtained may be used for screening and need not be repeated for enrollment eligibility.

5.1.2. Treatment Period

A total of 690 patients will be randomized. Eligible patients with advanced, treatment-naïve NSCLC will be randomized 1:1:1 to one of the following treatment arms:

Treatment Period: Treatment Arm A

- Treatment Arm A: standard-of-care platinum-based doublet chemotherapy Q3W for 4 to 6 cycles (followed by optional pemetrexed maintenance for those patients initially assigned to receive a pemetrexed-containing regimen)

Patients randomized to Treatment Arm A will receive a standard-of-care platinum-based doublet chemotherapy Q3W for 4 to 6 cycles (depending on patient tolerability and disease assessment) or until RECIST 1.1-defined progressive disease or early treatment discontinuation for another reason, including unacceptable toxicity, withdrawal of consent, death, or initiation of another anti-cancer treatment.

Following 4 to 6 cycles of chemotherapy, patients randomized to receive pemetrexed will have the option of pemetrexed maintenance according to local prescribing information and practice guidelines. Patients will enter the follow-up period after the last cycle of chemotherapy or pemetrexed maintenance therapy.

Treatment Period: Treatment Arm B

- Treatment Arm B: REGN2810 350 mg Q3W for 108 weeks plus standard-of-care platinum-based doublet chemotherapy Q3W for 4 to 6 cycles (REGN2810/chemo-f)

CONFIDENTIAL

VV-RIM-00023209-1.0 Approved - 29 Aug 2017 GMT-5:00

Patients randomized to Treatment Arm B will receive the following treatments in combination:

- REGN2810 350 mg as an IV infusion on day 1 of every treatment cycle (Q3W) for 108 weeks or until RECIST 1.1-defined progressive disease or early treatment discontinuation for another reason, including unacceptable toxicity, withdrawal of consent, death, or initiation of another anti-cancer treatment, or in specific instances of confirmed CR or PR
- Platinum-based doublet chemotherapy Q3W for 4 to 6 cycles (depending on patient tolerability and disease assessment)
  - The choice of platinum-based doublet chemotherapy will be at the discretion of the investigator from 1 of the options listed in Section 7.1.3 and is to be decided and documented prior to randomization.
  - Patients in this arm will not be permitted to begin pemetrexed maintenance.

Treatment Period: Treatment Arm C

- Treatment Arm C: REGN2810 350 mg Q3W for 108 weeks plus standard-of-care platinum-based doublet chemotherapy for 2 cycles and ipilimumab 50 mg Q6W for up to 4 doses (REGN2810/chemo-l/ipi)

Patients randomized to Treatment Arm C will receive the following treatments in combination:

- REGN2810 350 mg as an IV infusion on day 1 of every treatment cycle (Q3W) for 108 weeks or until RECIST 1.1-defined progressive disease or early treatment discontinuation for another reason, including unacceptable toxicity, withdrawal of consent, death, or initiation of another anti-cancer treatment, or in specific instances of confirmed CR or PR
- Platinum-based doublet chemotherapy Q3W for 2 cycles (depending on patient tolerability and disease assessment)
  - The choice of platinum-based doublet chemotherapy will be at the discretion of the investigator from one of the options listed in Section 7.1.3 and is to be decided and documented prior to randomization.
  - Patients in this arm will not be permitted to begin pemetrexed maintenance.
- Ipilimumab 50 mg flat dose administered IV on day 1 of every other treatment cycle (ie, every 42 days or Q6W) for up to 4 doses.

Treatment Period: All Treatment Arms

Randomization will be stratified by histology (non-squamous versus squamous) and levels of PD-L1 expression (<1% versus 1% to 24% versus 25% to <50%).

Treatment should begin within 3 days of randomization. Details of the treatment regimens are provided in Section 7.1.

For the purposes of this study, a treatment cycle will be defined as 21 days or 3 weeks.

Laboratory results for safety assessments must be available prior to dosing on day 1 of each dosing cycle (Table 5).

VV-RIM-00023209-1.0 Approved - 29 Aug 2017 GMT-5:00

Details of the treatment regimens are provided in Section 7.1.1, and details of dose modifications and study drug permanent and temporary discontinuation criteria are discussed in Section 7.3.

Radiographic tumor assessments will be obtained Q9W beginning at week 9 (day 63 ± 5 days) during year 1 and Q12W beginning at week 55 (first radiographic tumor assessment in year 2 performed at end of week 54) during year 2, until IRC-assessed RECIST 1.1-defined progressive disease, withdrawal of consent, death, or initiation of another anti-cancer treatment. See Section 8.2.2.1 for detailed timing of radiographic tumor assessments. Patients who discontinue for reasons other than progression who are not attending treatment visits may have radiographic tumor assessments between Q9W and Q12W until RECIST 1.1-defined progressive disease, withdrawal of consent, death, or initiation of another anti-cancer treatment.

Progressive disease will be defined using RECIST 1.1 criteria (Appendix 2). Investigators and the blinded IRC (Section 5.3.1) will assess response to therapy using RECIST 1.1 criteria. RECIST 1.1-defined progressive disease determined by the investigator will be used for clinical management of the patient. RECIST 1.1-based tumor burden assessments by the blinded IRC will be used for evaluation of efficacy endpoints.

Patients who experience RECIST 1.1-defined progressive disease on therapy may continue treatment if the investigator judges the patient to be experiencing clinical benefit and if the patient has not completed the 108-week treatment period (Section 7.8). Alternatively, these patients may opt to initiate a new anti-cancer treatment. If a patient continues treatment beyond the initial determination of progressive disease, study assessments should continue as per Table 5. If on the next scheduled radiographic tumor assessment, RECIST 1.1-defined further progressive disease is confirmed (Section 7.8), therapy will be discontinued. Further progression will be defined as an additional 10% increase in tumor burden from the time of initial progressive disease.

Safety will be assessed through the occurrence of AEs, recording of concomitant medications, vital sign evaluation, physical examination, ECOG performance status, and laboratory analyses (Table 5).

To assess disease-related symptoms, patients will be asked to complete QOL questionnaires at time points specified in Table 5.

Details of the treatment regimens are provided in Section 7.1, and details of dose modifications and study drug permanent and temporary discontinuation criteria are discussed in Section 7.3.

Blood samples will be collected from patients in treatment arms B and C to measure serum concentrations of REGN2810 and REGN2810 ADA titers. Blood samples will be collected to measure biomarkers associated with clinical response to REGN2810 including cytokines, circulating nucleic acids, and other potential biomarkers of interest (Table 5).

After at least 6 months (24 weeks) of treatment, a patient with confirmed CR may choose to stop REGN2810 treatment early and be followed for the duration of the study. A patient with a PR that has stabilized after 6 months and is no longer changing after 3 successive tumor assessments may also choose to stop REGN2810 treatment early and be followed for the duration of the study.

CONFIDENTIAL

VV-RIM-00023209-1.0 Approved - 29 Aug 2017 GMT-5:00

5.1.3. Follow-Up Period

Patients who discontinue study treatment due to progressive disease should return to the clinic 14 to 30 days after the last study treatment to complete the end of study assessments (follow-up visit1).

Patients who discontinue study treatment for a reason other than progressive disease should return to the clinic 14 to 30 days (±7 days) after the last cycle visit for follow-up visit 1 and then continue with follow-up visit 2 through follow-up visit 7.

Survival data will then be collected by phone or at an office visit every 3 months, until death, loss to follow-up, or withdrawal of study consent.

Follow-up Period: Treatment Arm A

- Patients in Treatment Arm A will enter follow-up after completion of the last dose of chemotherapy (completion of 4 to 6 cycles) or completion of pemetrexed maintenance therapy.
- Patients who do not start pemetrexed maintenance and enter follow-up after 4 to 6 cycles of chemotherapy without RECIST 1.1-defined progressive disease will continue to have radiographic tumor assessments according to the same schedule as the other treatment arms, as outlined in Section 8.2.2.1.
- Patients that opt to be treated with other anti-cancer treatments will be expected to complete all follow-up assessments as specified in Table 6.

Follow-up Period: Treatment Arms B and C

- Patients in Treatment Arms B and C will enter the follow-up period after completion of the 108-week treatment period, at the time of RECIST 1.1-defined progressive disease, or when the decision is made to discontinue REGN2810 treatment.
- Patients who completed the treatment period without RECIST 1.1-defined progressive disease or who discontinued study treatment early for reasons other than RECIST 1.1-defined progressive disease should continue to have radiographic tumor assessments Q12W.
- Patients who discontinued REGN2810 treatment early (but after at least 6 months [24 weeks] of treatment) due to CR, PR, or stable disease (SD) and entered follow-up at that time who then have RECIST 1.1-defined progressive disease while in follow-up may be offered the option to begin retreatment with REGN2810 350 mg Q3W in order to complete a total of 108 weeks of REGN2810 treatment. Study assessments for these patients will be performed as specified in Table 5.
- Follow-up study assessments will be performed as specified in Table 6. Patients assigned to Treatment Arms B and C will have blood samples taken for PK and ADA testing as specified in Table 6.

5.1.4. Description of Study Stopping Rules

More than 353 patients have dosed as of April 2017 with REGN2810 with no DLTs observed. Because this is the first study of the REGN2810/chemo-l/ipi combination, safety data

from the first 10 patients treated with REGN2810/chemo-l/ipi in Treatment Arm C will be reviewed after completing 4 weeks of follow-up following the first dose of REGN2810/chemo-l/ipi. The data will be reviewed at a meeting of the Independent Data Monitoring Committee (IDMC). If 2 or more DLTs occur in the first 10 patients treated with REGN2810/chemo-l/ipi, enrollment for this treatment arm will be stopped temporarily. Enrollment in Treatment Arm C will be restarted only after a formal early safety review. The outcome of the early safety review will be a decision to do one of the following:

- Continue the study as planned after discussions with investigators and regulatory authorities
- Continue the study without the second cycle of chemotherapy
- Increase the interval for ipilimumab administration from 6 to 12 weeks in Treatment Arm C
- Discontinue Treatment Arm C

5.1.5. Dose-Limiting Toxicities

The DLT observation period for determination of safety is defined as 28 days starting with cycle 1 day 1, with the intent to monitor safety and tolerability of REGN2810/chemo-l/ipi.

Any of the below outlined events occurring during the DLT observation period and considered to be at least possibly related to REGN2810/chemo-l/ipi will qualify as a DLT.

A DLT is defined as follows:

Non-Hematologic Toxicity

1. Grade ≥2 uveitis (considered as a potential irAE)
2. Any grade ≥3 non-hematologic toxicity, with the exception of the following:
    a. Grade 3 nausea, vomiting, or diarrhea unless persistent (>7 days of duration) despite maximal supportive care measures, as prescribed by the treating physician
    b. Grade ≥3 laboratory abnormalities that are considered clinically insignificant and do not meet criteria for an AE
    c. Grade 3 infusion-related reactions that respond to medical management
    d. Grade 3 irAE (as defined by experience with other immunomodulatory drugs; see Section 7.3.3 for the description of common irAEs) other than uveitis that improves within 14 days to grade 2 or lower with medical management (including treatment with steroids)

Hematologic Toxicity

1. Grade 4 neutropenia lasting more than 7 days
2. Grade 4 thrombocytopenia
3. Grade 3 thrombocytopenia with bleeding
4. Grade ≥3 febrile neutropenia (fever ≥38.5°C with absolute neutrophil count [ANC] <1.0 × 10^9/L) or grade ≥3 neutropenia with documented infection

The frequency, time to onset, and severity of toxicities, as well as the success of standard medical management and dosing interruptions/delays (Section 7.3.1), will be analyzed to determine if a given toxicity should be considered a DLT. Both irAEs and non-irAEs that meet the definition of a DLT will be considered to be DLTs.

In general, because there is no clinical experience with the REGN2810/chemo-l/ipi combination, any AE that has been clearly described for other agents that block the PD-1/PD-L1 and/or CTLA-4 pathways or the combination will be treated initially as unexpected in this study. Such TEAEs will be monitored and especially considered on an ongoing basis to assess expectedness, including possible differences in event frequency or severity from that observed with other PD-1/PD-L1 and CTLA-4 blockers.

The TEAEs that appear to meet the DLT definition will be discussed between the sponsor and the investigator. The final decision of whether or not the TEAE meets the DLT definition will be based on a careful review of all relevant data and consensus between the Medical Monitor, Clinical Development Lead, and the designated Risk Management Lead from the Pharmacovigilance and Risk Management department. The investigator may also be consulted.

Regardless of whether a patient remains on study treatment and/or continues to participate in study procedures, such an event will count as a DLT, if the event occurs during the DLT observation period.

5.1.6. Maximum Tolerated Dose

If 2 or more of the first 10 patients from Treatment Arm C who are dosed with REGN2810/chemo-l/ipi experience a DLT during the DLT monitoring period, the dosing interval for ipilimumab could be increased from Q6W to Q12W or the number of cycles of platinum doublet chemotherapy reduced from 2 to 1. If 2 or more DLTs occur in the first 10 patients treated in Treatment Arm C, enrollment to this treatment arm will be stopped temporarily and will be restarted only after the formal early safety review.

The MTD is defined as the dose level immediately below the level at which dosing is stopped due to the occurrence of 2 or more DLTs. If the study is not stopped due to the occurrence of a DLT, it will be considered that the MTD has not been determined.

Based on data with REGN2810 and other anti-PD-1 investigational compounds, it is possible that an MTD may not be defined in this study.

5.1.7. End of Study Definition

The end of study is defined as the last visit of the last patient.

5.2. Planned Interim Analysis

No interim analysis is planned. There will be an early safety review by the IDMC as detailed in Section 5.1.4.

5.3. Study Committees

Two independent study committees will be utilized: an IRC (Section 5.3.1) and an IDMC (Section 5.3.2).

CONFIDENTIAL

VV-RIM-00023209-1.0 Approved - 29 Aug 2017 GMT-5:00

5.3.1. Independent Review Committee

A blinded IRC composed of members who are independent from the sponsor and the study investigators will review all available (de-identified) radiographic tumor assessments to determine tumor response based on RECIST 1.1 criteria. The IRC-determined tumor response will be used in the analysis of the PFS and ORR endpoints. Details of the IRC responsibilities and procedures will be specified in the IRC charter.

5.3.2. Independent Data Monitoring Committee

An IDMC, composed of members who are independent from the sponsor and the study investigators, will monitor patient safety by conducting formal reviews of accumulated safety data that will be blinded by treatment arm; if requested, the IDMC may have access to the treatment allocation code or any other requested data for the purposes of a risk-benefit assessment.

The IDMC will provide the sponsor with appropriate recommendations on the conduct of the clinical study to ensure the protection and safety of the patients enrolled in the study. The IDMC will also institute any measures that may be required for ensuring the integrity of the study results during the study execution.

All activities and responsibilities of the IDMC are described in the IDMC charter.

6. SELECTION, WITHDRAWAL, AND REPLACEMENT OF PATIENTS

6.1. Number of Patients Planned

Approximately 690 patients will be randomized at approximately 200 global sites. Of the 690 patients, it is predicted that approximately 70% of the patients enrolling in the study will have non-squamous NSCLC and 30% of the patients will have squamous NSCLC histology.

6.2. Study Population

Patients in this study will include men and women ≥18 years of age, who are diagnosed with stage IIIB or stage IV non-squamous or squamous NSCLC, whose tumors express PD-L1 in <50% of tumor cells (measured using the PD-L1 IHC 22C3 pharmDx assay), and who have received no prior systemic treatment for their advanced disease.

To be eligible for the study, patients must meet all of the inclusion criteria (Section 6.2.1) and none of the exclusion criteria (Section 6.2.2).

6.2.1. Inclusion Criteria

A patient must meet the following criteria to be eligible for inclusion in the study:

1. Men and women ≥18 years of age.
2. Patients with histologically or cytologically documented squamous or non-squamous NSCLC with stage IIIB or stage IV disease, who received no prior systemic treatment for recurrent or metastatic NSCLC

- Patients who received adjuvant or neoadjuvant platinum-based doublet chemotherapy (after surgery and/or radiation therapy) and developed recurrent or metastatic disease more than 6 months after completing therapy.

3. Availability of an archival (≤5 months) or on-study obtained formalin-fixed, paraffin-embedded tumor tissue sample from a metastatic/recurrent site, which has not previously been irradiated.

4. Expression of PD-L1 in <50% of tumor cells determined by the commercially available PD-L1 IHC 22C3 pharmDx assay performed by the central laboratory.

5. At least 1 radiographically measureable lesion by computed tomography (CT) or magnetic resonance imaging (MRI) per RECIST 1.1 criteria (see Appendix 2). Target lesions may be located in a previously irradiated field if there is documented (radiographic) disease progression in that site.

6. ECOG performance status of ≤1.

7. Anticipated life expectancy of at least 3 months.

8. Adequate organ and bone marrow function as defined below:

   a. Hemoglobin ≥10.0 g/dL
   b. Absolute neutrophil count ≥1.5 × 10^9/L
   c. Platelet count ≥100,000/mm^3
   d. Glomerular filtration rate (GFR) >30 mL/min/1.73m^2
   e. Total bilirubin ≤1.5 × upper limit of normal (ULN) (if liver metastases ≤3 × ULN), with the exception of patients diagnosed with clinically confirmed Gilbert's syndrome
   f. Aspartate aminotransferase (AST) and alanine aminotransferase (ALT) ≤3 × ULN or ≤5 × ULN, if liver metastases
   g. Alkaline phosphatase ≤2.5 × ULN (or ≤5.0 × ULN, if liver or bone metastases)
   h. Not meeting criteria for Hy's law (ALT >3 × ULN and bilirubin >2 × ULN).

9. Willing and able to comply with clinic visits and study-related procedures.

10. Provide signed informed consent.

11. Able to understand and complete study-related questionnaires.

6.2.2. Exclusion Criteria

A patient who meets any of the following criteria will be excluded from the study:

1. Patients who have never smoked, defined as smoking ≤100 cigarettes in a lifetime.

2. Active or untreated brain metastases or spinal cord compression. Patients are eligible if central nervous system (CNS) metastases are adequately treated and patients have neurologically returned to baseline (except for residual signs or symptoms related to the CNS treatment) for at least 2 weeks prior to enrollment. Patients must be off (immunosuppressive doses of) corticosteroid therapy (see exclusion criteria 7) for details on timing of discontinuation of steroids).

3. Patients with tumors tested positive for EGFR gene mutations, ALK gene translocations, or ROS1 fusions.

VV-RIM-00023209-1.0 Approved - 29 Aug 2017 GMT-5:00

4. Encephalitis, meningitis, or uncontrolled seizures in the year prior to informed consent.
5. History of interstitial lung disease (eg, idiopathic pulmonary fibrosis or organizing pneumonia), of active, noninfectious pneumonitis that required immune-suppressive doses of glucocorticoids to assist with management, or of pneumonitis within the last 5 years. A history of radiation pneumonitis in the radiation field is permitted.
6. Ongoing or recent evidence of significant autoimmune disease that required treatment with systemic immunosuppressive treatments, which may suggest risk of immune-related treatment-emergent adverse events (irTEAEs). The following are not exclusionary: vitiligo, childhood asthma that has resolved, residual hypothyroidism that required only hormone replacement, or psoriasis that does not require systemic treatment.
7. Patients with a condition requiring corticosteroid therapy (>10 mg prednisone/day or equivalent) within 14 days of randomization. Physiologic replacement doses are allowed even if they are >10 mg of prednisone/day or equivalent, as long as they are not being administered for immunosuppressive intent. Inhaled or topical steroids are permitted, provided that they are not for treatment of an autoimmune disorder.
8. Previous treatment with idelalisib at any time (ZYDELIG®).
9. Another malignancy that is progressing or requires treatment, with the exception of nonmelanomatous skin cancer that has undergone potentially curative therapy, in situ cervical carcinoma, or any other localized tumor that has been treated, and the patient is deemed to be in complete remission for at least 2 years prior to study entry, and no additional therapy is required during the study period.
10. Known active hepatitis B (known positive result) or hepatitis C (known positive result) and known quantitative HCV RNA results greater than the lower limits of detection of the assay).
11. Known history of human immunodeficiency virus or known acquired immunodeficiency syndrome indicating uncontrolled active infection. Patients on highly active antiretroviral therapy with undetectable RNA levels and CD4 counts above 350 are permitted.
12. Active infection requiring systemic therapy within 14 days prior to randomization.
13. Prior therapy with anti-PD-1 or anti-PD-L1. Prior exposure to other immunomodulatory or vaccine therapies such as anti-CTLA-4 antibodies is permitted, but the last dose of such an antibody should have been at least 6 months prior to the first dose of study drug.
14. Treatment-related immune-mediated AEs from immune-modulatory agents (including but not limited to anti-PD1/PD-L1monoclonal antibodies, anti-CTLA4 monoclonal antibodies, and phosphatidylinositide 3-kinase inhibitors) that have not resolved to baseline at least 3 months prior to initiation of treatment with study therapy. Patients are excluded from treatment with REGN2810 if they experienced immune-mediated AEs related to prior treatment with a blocker of the PD-1/PD-L1 pathway that were grade 3 or 4 in severity and/or required discontinuation of the agent, regardless of time of occurrence.

VV-RIM-00023209-1.0 Approved - 29 Aug 2017 GMT-5:00

15. Receipt of an investigational drug or device within 30 days of screening or within 5 half-lives of the investigational drug or therapy being studied (whichever is longer).
16. Receipt of a live vaccine within 30 days of planned start of study medication.
17. Major surgery or significant traumatic injury within 4 weeks prior to first dose.
18. Documented allergic or acute hypersensitivity reaction attributed to antibody treatments in general or to agents specifically used in the study.
19. Known psychiatric or substance abuse disorder that would interfere with participation with and/or the requirements of the study, including current use of any illicit drugs.
20. Pregnant or breastfeeding women.
21. Women of childbearing potential* who are unwilling to practice highly effective contraception prior to the initial dose/start of the first treatment, during the study, and for at least 6 months after the last dose. Highly effective contraceptive measures include stable use of combined (estrogen and progestogen containing) hormonal contraception (oral, intravaginal, transdermal) or progestogen-only hormonal contraception (oral, injectable, implantable) associated with inhibition of ovulation initiated 2 or more menstrual cycles prior to screening; intrauterine device (IUD); intrauterine hormone-releasing system (IUS); bilateral tubal ligation; vasectomized partner; and or sexual abstinence†, ‡.

    *Postmenopausal women must be amenorrheic for at least 12 months in order not to be considered of childbearing potential. Pregnancy testing and contraception are not required for women with documented hysterectomy or tubal ligation.

    †Sexual abstinence is considered a highly effective method only if defined as refraining from heterosexual intercourse during the entire period of risk associated with the study treatments.

    ‡Periodic abstinence (calendar, symptothermal, post-ovulation methods), withdrawal (coitus interruptus), spermicides only, and lactational amenorrhea method (LAM) are not acceptable methods of contraception. Female condom and male condom should not be used together.

    Sexually active men and their partners must use highly effective contraception as described above. Contraception is not required for men with documented vasectomy.
22. Patients who are committed to an institution by virtue of an order issued either by the judicial or the administrative authorities will be excluded from this study.
23. Member of the clinical site study team and/or his/her immediate family, unless prior approval granted by the Sponsor.

6.3. Premature Withdrawal from the Study

A patient has the right to withdraw from the study at any time, for any reason, and without repercussion.

The investigator and/or sponsor have the right to withdraw a patient from the study if it is no longer in the interest of the patient to continue in the study, or if the patient's continuation in the study places the scientific outcome of the study at risk (eg, if a patient does not or cannot follow study procedures). An excessive rate of withdrawals would render the study uninterpretable; therefore, unnecessary withdrawal of patients should be avoided.

Patients who are withdrawn prematurely from the study will be asked to complete study assessments, as described in Section 8.1.2.

Rules for discontinuation of study treatment (permanent or temporary) are discussed in Section 7.3.6.

6.4. Replacement of Patients

Patients prematurely discontinued from the study or study treatment will not be replaced.

7. STUDY TREATMENTS

7.1. Investigational and Reference Treatments

Patients will be randomized to receive one of the following treatment regimens:

- Treatment Arm A: Standard-of-care platinum-based doublet chemotherapy Q3W for 4 to 6 cycles as an IV infusion (followed by optional pemetrexed maintenance for those patients initially assigned to receive a pemetrexed-containing regimen)
- Treatment Arm B: REGN2810 350 mg Q3W for 108 weeks as an IV infusion and standard-of-care platinum-based doublet chemotherapy Q3W for 4 to 6 cycles as an IV infusion (REGN2810/chemo-f)
- Treatment Arm C: REGN2810 350 mg Q3W for 108 weeks as an IV infusion standard-of-care platinum-based doublet chemotherapy Q3W for 2 cycles and ipilimumab 50 mg Q6W for up to 4 doses as an IV infusion (REGN2810/chemo-l/ipi)

Patients will receive their assigned treatment for the treatment period (as noted above) or until RECIST 1.1-defined progressive disease or early treatment discontinuation for another reason.

Study treatment will be administered by the investigator or other designated study personnel.

7.1.1. REGN2810

REGN2810 is a covalent heterotetramer consisting of 2 disulfide-linked human heavy chains, each of which is covalently bonded through disulfide linkages to a human kappa light chain. The antibody possesses an approximate molecular weight of 143.6 kDa based on the primary sequence. There is a single N-linked glycosylation site on each heavy chain, located within the constant region in the Fc portion of the molecule. The REGN2810 heavy chain possesses an IgG4 isotype constant region. The variable domains of the heavy and light chains combine to form PD-1 binding site within the antibody.

REGN2810 is manufactured by Regeneron Pharmaceuticals, Inc. REGN2810 is manufactured at 25 and 50 mg/mL.

REGN2810 (50 mg/mL) is manufactured with Cell Line 2 Process 1 (C2P1) and formulated in an aqueous buffered solution at pH 6.0 containing 10 mM histidine, 5% (w/v) sucrose, 1.5% (w/v) L-proline, and 0.2% (w/v) polysorbate 80. REGN2810 C2P1 drug product is supplied as a sterile liquid solution of 5.6 or 7.4 mL in a 10 or 20 mL glass vial for IV administration.

REGN2810 will be supplied as a liquid in sterile, single-use vials. Each vial will contain a volume sufficient to withdraw 5 mL or 7 mL of REGN2810 at a concentration of 50 mg/mL. See Section 7.6 for details on packaging, labeling, and storage.

REGN2810 will be administered in an outpatient setting as a 30-minute (±10 minutes) IV infusion.

Instructions on dose preparation are provided in the pharmacy manual. Instructions on management of acute infusion reactions are provided in Section 7.4.1.

REGN2810 will be administered in combination with either platinum-based doublet chemotherapy (administered Q3W 4 to 6 cycles; REGN2810/chemo-f) or with platinum-based doublet chemotherapy (administered Q3W for 2 cycles) and ipilimumab (administered Q6W for up to 4 doses; REGN2810/chemo-f/ipi) and then alone for the remainder of the treatment period.

When administered in combination with ipilimumab and chemotherapy, infuse chemotherapy, then REGN2810, then ipilimumab on the same day. Use separate infusion bags and filters for each infusion.

When administered in combination with ipilimumab, infuse REGN2810 first followed by ipilimumab on the same day. Use separate infusion bags and filters for each infusion.

When administered in combination with platinum-based doublet chemotherapy agents, the chemotherapy agents should be infused first, followed by REGN2810, on the same day. Use separate infusion bags and filters for each infusion.

7.1.2. Ipilimumab

Ipilimumab should be procured by the study sites as local commercial products in some countries and where allowed by local regulations; for other countries, Regeneron may provide the ipilimumab to the study sites. See Section 7.6 for details on packaging, handling, and storage.

Ipilimumab 50 mg flat dose will be administered IV over approximately 90 minutes on day 1 every 42 days (Q6W) for up to 4 doses in combination with REGN2810. Administration should adhere to the local prescribing information and practice guidelines.

Instructions on dose preparation are provided in the pharmacy manual. Instructions on management of acute infusion reactions are provided in Section 7.4.1.

Ipilimumab should be infused after REGN2810 on the same day. Use separate infusion bags and filters for each infusion.

7.1.3. Platinum-based Doublet Chemotherapy

Chemotherapy will be administered Q3W as outlined in Table 1 for 4 to 6 cycles or 2 cycles (depending on randomized treatment arm, patient tolerability, and disease assessment) or until RECIST 1.1-defined progressive disease or early treatment discontinuation for another reason.

VV-RIM-00023209-1.0 Approved - 29 Aug 2017 GMT-5:00

Chemotherapy will be administered and according to local prescribing information and practice guidelines.

The choice of chemotherapy will be one of the regimens shown in Table 1. The investigator may choose from one of these regimens provided that it is consistent with the local standard-of-care (Table 1). Assignment of the chemotherapy choice must be made prior to randomization. Chemotherapy should be procured by the study sites as local commercial products in some countries and where allowed by local regulations; for other countries, Regeneron may provide the chemotherapy to the study sites. Preference should be given to regimens that are allowed by local regulations.

The suggested sequence of drug administration is platinum-based doublet chemotherapy agents followed by REGN2810.

VV-RIM-00023209-1.0 Approved - 29 Aug 2017 GMT-5:00

Table 1: Guidelines for Platinum-Based Doublet Chemotherapy Regimens

Option	Chemotherapy Regimen	Dosing Frequency	Maintenance Therapy
1	Gemcitabine 1250 mg/m^2 IV plus carboplatin AUC of 5 or 6 mg/mL/minute IV	Day 1 (both gemcitabine and carboplatin) and day 8 (gemcitabine only) every 21 days (Q3W) for 2 cycles or 4 to 6 cycles<br>Calculate dose of carboplatin using the Calvert formula.	N/A
2	Gemcitabine 1250 mg/m^2 IV plus cisplatin 100 mg/m^2 IV	Day 1 (both gemcitabine and cisplatin) and day 8 (gemcitabine only) every 21 days (Q3W) for 2 cycles or 4 to 6 cycles	N/A
3	Paclitaxel 200 mg/m^2 IV plus carboplatin AUC of 5 or 6 mg/mL/minute IV	Day 1 every 21 days (Q3W) for 2 cycles or 4 to 6 cycles<br>Calculate dose of carboplatin using the Calvert formula.	N/A
4	Paclitaxel 200 mg/m^2 IV plus cisplatin 75 mg/m^2 IV	Day 1 every 21 days (Q3W) for 2 cycles or 4 to 6 cycles	N/A
5	Pemetrexed 500 mg/m^2 IV plus carboplatin AUC of 5 or 6 mg/mL/minute IV	Day 1 every 21 days (Q3W) for 2 cycles or 4 to 6 cycles<br>Calculate dose of carboplatin using the Calvert formula.	**For Treatment Arm A only:** Optional pemetrexed maintenance 500 mg/m^2 IV day 1 every 21 days; pemetrexed maintenance according to local prescribing information and practice guidelines.
6	Pemetrexed 500 mg/m^2 IV plus cisplatin 75 mg/m^2 IV	Day 1 every 21 days (Q3W) for 2 cycles or 4 to 6 cycles	**For Treatment Arm A only:** Optional pemetrexed maintenance 500 mg/m^2 IV day 1 every 21 days; pemetrexed maintenance according to local prescribing information and practice guidelines.

Abbreviations: AUC=area under the curve; IV=intravenous; N/A=not applicable

These are general guidelines. The dosages should be according to the local prescribing information and practice guidelines.

Note that patients with GFR <50 mL/min/1.73m^2 may NOT receive cisplatin-containing regimens.

7.2. Pre-treatments

Premedications should be procured by the study sites as local commercial products in some countries and where allowed by local regulations; for other countries, Regeneron may provide the premedications. Preference should be given to regimens that are allowed by local regulations.

No premedications are to be administered prior to the first administration of REGN2810. Premedications will be allowed at subsequent doses depending on the need to manage any observed low grade infusion reactions (Section 7.4.1.1).

For standard-of-care chemotherapy, pre-medications should be administered in accordance with the local prescribing information and practice guidelines. In general, it is recommended that patients receive corticosteroids, diphenhydramine, and an H_2-receptor blocker prior to receipt of paclitaxel. It is recommended that patients receiving pemetrexed receive vitamin supplementation (folic acid and vitamin B12) and corticosteroids. Patients receiving cisplatin should be adequately hydrated prior to the infusion of therapy and receive highly effective combination antiemetic therapy.

7.3. Dose Modification and Study Treatment Discontinuation Rules

7.3.1. REGN2810 Plus Ipilimumab and Chemotherapy Combination Therapy

Dosing Delay Rules

Patients in Treatment Arm C who experience protocol-defined DLTs (see Section 5.1.5; either during or outside of the DLT observation period) will be required to temporarily discontinue treatment with REGN2810/chemo-l/ipi.

In addition to DLTs, during the REGN2810/chemo-l/ipi therapy period, administration of REGN2810 (and of ipilimumab, if an AE occurs on the day of a planned ipilimumab dosing), must be delayed due to the following AEs:

- Either febrile neutropenia or neutropenia <500 cells/mm³ for >1 week despite the use of growth factors
- Any grade ≥2 non-skin, drug-related AE, except for fatigue and laboratory abnormalities and except for AEs that require study treatment discontinuation (as listed below)
- Any grade 3 drug-related laboratory abnormality (except for lymphopenia, AST, ALT, or total bilirubin or asymptomatic lipase or amylase)
  - Grade 3 lymphopenia will not require a dose delay
  - If the patient had a baseline AST, ALT, or total bilirubin level that was within normal limits, dosing should be delayed for a drug-related grade ≥2 toxicity

  - If the patient had a baseline AST, ALT, or total bilirubin level that was within the grade 1 toxicity range, dosing should be delayed for a drug-related grade ≥3 toxicity
  - Any grade 3 drug-related amylase or lipase abnormality that is not associated with symptoms or clinical manifestations of pancreatitis will not require a dose delay
- Any grade 3 skin drug-related AE
- Any AE, laboratory abnormality, or intercurrent illness that, in the judgment of the investigator, warrants delaying the dose of study treatment

Resumption of treatment may be at the initial dose regimen or the dosing interval for ipilimumab could be increased from Q6W to Q12W or the number of cycles of platinum-based doublet chemotherapy reduced from 2 to 1, based upon the discretion of the investigator and the sponsor. Dose modification of REGN2810 will not be permitted.

A repeat occurrence of the same DLT after resumption of treatment will require permanent discontinuation of REGN2810/chemo-l/ipi.

Criteria for Restarting REGN2810 Plus Ipilimumab and Chemotherapy Dosing

Patients may resume treatment with REGN2810/chemo-l/ipi when the drug-related AE(s) resolve to grade ≤1 or baseline value, with the following exceptions:

- Patients may resume study treatment in the presence of grade 2 fatigue.
- Patients who have not experienced a grade 3 drug-related skin AE may resume study treatment in the presence of grade 2 skin toxicity.
- Patients with baseline grade 1 AST, ALT, or total bilirubin level who require dose delays for reasons other than a 2-grade shift in AST, ALT, or total bilirubin may resume study treatment in the presence of grade 2 AST, ALT, OR total bilirubin.
- Patients with AST, ALT, and/or total bilirubin values meeting discontinuation criteria should have study treatment permanently discontinued.
- Drug-related pulmonary toxicity, diarrhea, or colitis must resolve to baseline before study treatment is resumed.
- Drug-related endocrinopathies adequately controlled with only physiologic hormone replacement may resume study treatment.
- Any AE that meets the discontinuation rules below requires that the patient discontinue study treatment.

Dosing Discontinuation Rules

The following categories require permanent discontinuation of REGN2810/chemo-l/ipi:

- Any grade 2 drug-related uveitis, eye pain, or blurred vision that does not respond to topical therapy and does not improve to grade 1 severity within the retreatment period, or that requires systemic treatment require study treatment discontinuation.

- Any grade 3 non-skin, drug-related AE lasting >7 days require study treatment discontinuation, with the following exceptions for laboratory abnormalities, drug-related uveitis, pneumonitis, bronchospasm, colitis, diarrhea, hypersensitivity reactions, infusion reactions, and endocrinopathies:
  - Grade 3 drug-related uveitis, pneumonitis, bronchospasm, colitis, diarrhea, hypersensitivity reaction, or infusion reaction of any duration require study treatment discontinuation
  - Grade 3 drug-related endocrinopathies adequately controlled with only physiologic hormone replacement do not require study treatment discontinuation
  - Grade 3 drug-related laboratory abnormalities do not require study treatment discontinuation except for the following:
    - Grade 3 drug-related thrombocytopenia >7 days or associated with bleeding requires study treatment discontinuation
    - Any drug-related liver function test abnormality that meets the following criteria requires study treatment discontinuation:
      - AST or ALT >5 to 10 × ULN for >2 weeks
      - AST or ALT >10 × ULN
      - Total bilirubin >5 × ULN
      - Concurrent AST or ALT >3 × ULN and total bilirubin >2 × ULN
  - Any grade 4 drug-related AE or laboratory abnormality requires study treatment discontinuation, except for the following events, which do not require study treatment discontinuation:
    - Grade 4 neutropenia ≤7 days
    - Grade 4 lymphopenia or leukopenia
    - Isolated grade 4 amylase or lipase abnormalities that are not associated with symptoms or clinical manifestations of pancreatitis
    - Isolated grade 4 electrolyte imbalances/abnormalities that are not associated with clinical sequelae and are corrected with supplementation/appropriate management within 72 hours of onset
    - Grade 4 drug-related endocrinopathy AEs, such as adrenal insufficiency, adrenocorticotropic hormone (ACTH) deficiency, hyperthyroidism, hypothyroidism, or glucose intolerance, that resolve or are adequately controlled with physiologic hormone replacement (corticosteroids, thyroid hormones) or glucose controlling agents, as applicable, may not require study treatment discontinuation per approval from the Medical Monitor
- Any study treatment delay resulting in no dosing for >6 weeks (REGN2810), >12 weeks (ipilimumab Q6W), or >18 weeks (ipilimumab Q12W, if dose was

VV-RIM-00023209-1.0 Approved - 29 Aug 2017 GMT-5:00

revised to Q12W due to a toxicity or another reason) requires study treatment discontinuation, with the exception of dosing delays to manage drug-related AEs, such as prolonged steroid tapers and with the exception of delays noted in the next bulleted item

- Study treatment delays resulting in no dosing for >6 weeks (REGN2810), >12 weeks (ipilimumab Q6W), or >18 weeks (ipilimumab Q12W, if dose was revised to Q12W due to a toxicity or another reason) that occur for non-drug-related reasons are permitted and may not require study treatment discontinuation, if approved by the Medical Monitor
- Any AE, laboratory abnormality, or intercurrent illness, which, in the judgment of the investigator, presents a substantial clinical risk to the patient with continued REGN2810 or ipilimumab dosing, requires study treatment discontinuation.

If the investigator determines that an AE is definitely related to ipilimumab based on the Reference Safety Information in the Investigator's Brochure or based on local prescribing information and practice guidelines, the patient may discontinue treatment with ipilimumab and continue treatment with only REGN2810.

Additional guidelines for discontinuation of ipilimumab are provided in Section 7.3.4.

7.3.2. REGN2810 as Combination Therapy With Chemotherapy

The following REGN2810 treatment hold guidelines should be followed for patients in Treatment Arm B throughout the course of the study and for patients in Treatment Arm C following completion of ipilimumab dosing (when REGN2810 will be used alone).

REGN2810 treatment may be held upon occurrence of a treatment-related AE at any time on the study. Resumption of REGN2810 therapy after resolution or stabilization of the condition is allowed at the discretion of the investigator and sponsor if resuming treatment is thought to be in the best interest of the patient, with the exception of the following categories:

- Patients with events that require REGN2810 to be discontinued permanently or held for more than 84 days from the last scheduled dose
- Patients with grade ≥2 uveitis. Patients with grade 2 uveitis will generally be discontinued from REGN2810 treatment unless there is resolution to grade ≤1 as outlined in Appendix 3 AND discussion with and approval by the Medical Monitor. All patients with grade ≥3 uveitis will be permanently discontinued from REGN2810

Dose modification of REGN2810 will not be permitted.

Guidelines for REGN2810 temporary discontinuations, including delays and interruptions, criteria for restarting, and permanent discontinuations for toxicity are outlined in Table 2 and Appendix 3.

VV-RIM-00023209-1.0 Approved - 29 Aug 2017 GMT-5:00

Table 2: REGN2810 Guidelines for Temporary and Permanent Discontinuations for Toxicity

Toxicity	Grade	Hold Treatment?	Restarting Criteria	Restarting Dose/Schedule	Discontinuation Criteria
**Hematological toxicity** (other than grade 3 thrombocytopenia >7 days or associated with bleeding)	1, 2, 3	No	N/A	N/A	N/A
	4	Yes	Toxicity resolves to grade 0 to 1 or baseline	Same dose and schedule	Toxicity does not resolve within 84 days of last infusion.<br>*Permanent discontinuation should be considered for any severe or life-threatening event.*
Grade 3 thrombocytopenia >7 days or associated with bleeding	3	Yes	Toxicity resolves to grades 0 to 1 or baseline	Same dose and schedule	Toxicity does not resolve within 84 days of last infusion.<br>*Permanent discontinuation should be considered for any severe or life-threatening event.*
**Nonhematological toxicity**<br>*Note:* Exceptions to be treated as for grade 1 toxicity:<br>• Grade 2 alopecia<br>• Grade 2 fatigue<br>• Clinically insignificant lab abnormality not meeting AE criteria	1	No	N/A	N/A	N/A
	2	Consider withholding for persistent symptoms	Toxicity resolves to grades 0 to 1 or baseline	*Clinical AE resolves within 4 weeks*: Same dose and schedule<br>*Clinical AE does not resolve within 4 weeks:* Discontinue	Toxicity does not resolve within 84 days of last infusion.
	3	Yes	Toxicity resolves to grades 0 to 1 or baseline	Same dose and schedule	Toxicity does not resolve within 84 days of last infusion.
	4	Yes	N/A	N/A	Patient must be discontinued.

Abbreviations: AE=adverse event; irAE=immune-related adverse event; N/A=not applicable
For additional information regarding potential irAEs with a potential for irAEs, see Table 3 and Appendix 3.

Appendix 3 includes recommendations on the management of specific treatment-related AEs and when to delay and/or discontinue REGN2810. These guidelines are intended to be applied when the investigator determines the events to be treatment-related.

Additional reasons for REGN2810 permanent discontinuation include the following:

- REGN2810 dosing will be permanently discontinued in the event of pregnancy.
- In the event of an infusion reaction of grade 3 or greater severity during or directly following REGN2810 infusions, dosing should be stopped and the patient must be permanently discontinued from REGN2810 treatment. Infusion reactions are defined in Section 9.3.4.
- In addition, REGN2810 for any patient may be discontinued for other safety reasons or compliance issues at the discretion of the investigator or sponsor. A patient may choose to discontinue REGN2810 or study participation at any time for any reason.

After at least 6 months (24 weeks) of treatment, patients in Treatment Arm C with confirmed CR may choose to stop REGN2810 treatment early and be followed for the duration of the study. A patient with a PR that has stabilized after 6 months and is no longer changing after 3 successive tumor assessments may also choose to stop REGN2810 treatment early and be followed for the duration of the study.

A patient who permanently discontinues REGN2810 treatment should continue follow-up in the study without additional REGN2810 treatment until RECIST 1.1-defined progressive disease, completion of all study assessments, or closure of the study (Section 6.3 and Section 8.1.2).

Guidelines for dose delays and discontinuation of chemotherapy are provided in Section 7.3.5.

7.3.3. Immune-Related Adverse Events

Investigators must be extremely vigilant and be ready to intervene early in the management of irAEs because the onset of symptoms of irAEs (eg, pneumonitis) may be subtle. Immune-related TEAEs have been reported with REGN2810 and with other anti-PD-1 antibodies; these are considered consistent with the mechanism of action of anti-PD-1 antibodies.

An irTEAE can occur shortly after the first dose or several months after the last dose of treatment. All AEs of unknown etiology associated with drug exposure should be evaluated for a possible immune etiology. Efforts should be made to rule out neoplastic, infectious, metabolic, toxin or other etiologic causes prior to labeling an AE as an irTEAE. Suggested management guidelines are provided in Appendix 3 for certain anti-PD-1 irTEAEs including but not limited to:

- **Gastrointestinal events** (colitis, colitis microscopic, enterocolitis, enterocolitis hemorrhagic, gastrointestinal perforation, diarrhoea, stomatitis)
- **Pneumonitis events** (pneumonitis, acute interstitial pneumonitis)
- **Hepatic events** (ALT/AST increased, autoimmune hepatitis, transaminases increased)
- **Endocrine events** (autoimmune thyroiditis, blood thyroid stimulating hormone [TSH] increased, diabetic ketoacidosis, diabetes mellitus, hyperthyroidism,

hypophysitis, hypopituitarism, hypothyroidism, thyroid disorder, thyroiditis, adrenal insufficiency, Type 1 diabetes mellitus)

- **Uveitis** (iritis, iridocyclitis, uveitis)
- **Renal events (**nephritis, autoimmune nephritis, tubulointerstitial nephritis)
- **Skin events** (dermatitis, dermatitis acneiform, dermatitis bullous, dermatitis exfoliative, dermatitis exfoliative generalized, exfoliative rash, pruritus, pruritus generalized, rash, rash erythematous, rash generalized, rash macular, rash maculopapular, rash maculovesicular, rash morbilliform, rash papular, rash pruritic, rash rubelliform, rash scarlatiniform, rash vesicular, vitiligo)
- **Nervous System Events** (encephalitis, paraneoplastic encephalomyelitis, myasthenia gravis)

Based on the emerging safety profile of REGN2810 and other antibodies targeting the PD-1/PD-L1 axis (Weber 2015, Naidoo 2015), the following working case definitions are provided to help investigators distinguish irTEAEs from non-immune AEs. These case definitions pertain to the more commonly reported irTEAEs associated with PD-1 inhibition (Weber 2015, Naidoo 2015), and is not exhaustive of all possible irAEs. Clinical presentations of less common irAEs, including neurologic, musculoskeletal, cardiac, renal, and ocular events (Zimmer 2016, Hofmann 2016), should be reviewed in patients with concerning presentations.

The investigator should refer to the latest version of the Investigator's Brochure for further details and guidance. The case definitions have not been validated, and are intended only as guidance for investigators to help distinguish irTEAEs from non-immune AEs. Investigators' clinical judgment may include other factors when determining immune-relatedness. The case definitions for irAEs may evolve as clinical experience increases with REGN2810 and other antibodies targeting the PD-1/PD-L1 axis.

VV-RIM-00023209-1.0 Approved - 29 Aug 2017 GMT-5:00

Table 3: General REGN2810 Treatment Hold Guidelines for Immune-Related Adverse Events

Severity	Withhold/Discontinue REGN2810 Treatment?	Supportive Care
Grade 1	No action.	Provide symptomatic treatment.
Grade 2	May withhold treatment.	Consider systemic corticosteroids in addition to appropriate symptomatic treatment.
Grade 3<br>Grade 4	Withhold treatment.<br>Discontinue if unable to reduce corticosteroid dose to <10 mg per day prednisone equivalent within 12 weeks of toxicity.	For any severe (grade 3-4) irAE, if symptoms worsen or do not improve on adequate corticosteroids within 48 to 72 hours, consider adding additional immunosuppressive agents (to be selected from agents such as: infliximab, CTX, cyclosporine, and mycophenolate mofetil). Referral of the patient to a specialized unit for assessment and treatment should be considered.

Abbreviations: CTX=cyclophosphamide; irAE=immune-related adverse event

7.3.4. Ipilimumab

Ipilimumab-related toxicities should be managed in accordance with local prescribing information and practice guidelines.

Ipilimumab should also be discontinued for the following reasons:

- Ipilimumab dosing will be permanently discontinued in the event of pregnancy.
- In the event of an infusion reaction of grade 3 or greater severity during or directly following infusions, dosing should be stopped and the patient must be permanently discontinued from ipilimumab treatment. Infusion reactions are defined in Section 9.3.4.
- Ipilimumab may be discontinued for other safety reasons or compliance issues at the discretion of the investigator or sponsor. A patient may choose to discontinue REGN2810 plus ipilimumab combination therapy or study participation at any time for any reason.
- Any reason listed in the local prescribing information and practice guidelines.

If a patient experiences a toxicity that is known to be associated only with ipilimumab therapy, ipilimumab will be discontinued but REGN2810 may be continued.

7.3.5. Platinum-Based Doublet Chemotherapy

Dose modification/reduction or temporary cessation of a given chemotherapy should be managed in accordance with the local prescribing guidelines for the specific chemotherapy agent.

Chemotherapy should be permanently discontinued for safety reasons, compliance issues, intolerance due to toxicity, or other reasons as provided by local prescribing information and practice guidelines and standard-of-care.

If a patient experiences a toxicity that is known to be associated with chemotherapy, chemotherapy treatment will be discontinued but REGN2810 treatment may be continued.

VV-RIM-00023209-1.0 Approved - 29 Aug 2017 GMT-5:00

A patient who permanently discontinues from chemotherapy should continue follow-up in the study without additional chemotherapy treatment until RECIST 1.1-defined progressive disease, completion of all study assessments, or closure of the study (see Section 6.3 and Section 8.1.2).

7.3.6. Permanent Study Drug Discontinuation

Patients who permanently discontinue from study drug and who do not withdraw from the study will be asked to return to the clinic for all remaining study visits per the visit schedule.

Patients who permanently discontinue from study drug and who opt to withdraw from the study will be asked to complete study assessments, per Section 8.1.2.

7.4. Management of Acute Reactions

7.4.1. Acute Infusion Reactions

Emergency equipment and medication for the treatment of infusion reactions must be available for immediate use. Vital signs should be closely monitored according to Table 4, Table 5, and Table 6. All infusion reactions must be reported as AEs (as defined in Section 9.4.1) and graded using the grading scales as instructed in Section 9.5.1.

Acute infusion reactions are defined as any AE that occurs during the infusion or within 2 hours after the infusion is completed. Emergency equipment and medication for the treatment of these potential adverse effects (eg, antihistamines, bronchodilators, IV saline, corticosteroids, acetaminophen, and/or epinephrine) must be available for immediate use. Infusion reactions must be graded according to the current version of the National Cancer Institute Common Terminology Criteria for Adverse Events (NCI-CTCAE) grading scale (Section 9.5.1).

In the event of an infusion reaction of grade 3 or greater severity during or directly following REGN2810, ipilimumab, or chemotherapy infusion, dosing must be stopped and the patient must be permanently discontinued from treatment. Infusion reactions are defined in Section 9.3.4.

Acute infusion reactions can include cytokine release syndrome, angioedema, or anaphylaxis, and differ from allergic/hypersensitive reactions, although some of the manifestations are common to both AEs. Signs and symptoms usually develop during or shortly after drug infusion and generally resolve completely within 24 hours of the completion of infusion.

Signs/symptoms may include:

- Allergic reaction/hypersensitivity (including drug fever)
- Arthralgia (joint pain)
- Bronchospasm
- Cough
- Dizziness
- Dyspnea (shortness of breath)
- Fatigue (asthenia, lethargy, malaise)
- Headache

VV-RIM-00023209-1.0 Approved - 29 Aug 2017 GMT-5:00

- Hypertension
- Hypotension
- Myalgia (muscle pain)
- Nausea
- Pruritus/itching
- Rash/desquamation
- Rigors/chills
- Diaphoresis (sweating)
- Tachycardia
- Tumor pain (onset or exacerbation of tumor pain due to treatment)
- Urticaria (hives, welts, wheals)
- Vomiting

7.4.1.1. Interruption of the Infusion

The infusion should be interrupted if any of the following AEs are observed:

- Cough
- Rigors/chills
- Rash, pruritus (itching)
- Urticaria (hives, welts, wheals)
- Diaphoresis (sweating)
- Hypotension
- Dyspnea (shortness of breath)
- Vomiting
- Flushing

The reaction(s) should be treated symptomatically, and the infusion may be restarted at 50% of the original rate.

If investigators feel there is a medical need for treatment or discontinuation of the infusion other than described above, they should use clinical judgment to provide the appropriate response according to typical clinical practice.

For patients who experience infusion-related hypersensitivity reactions that are less than grade 3 and who plan to continue treatment, pre-medication will be required for retreatment.

For grade 1 symptoms (mild reaction; infusion interruption not indicated; intervention not indicated), the following prophylactic medications are recommended for future infusions:

VV-RIM-00023209-1.0 Approved - 29 Aug 2017 GMT-5:00

diphenhydramine 50 mg (or equivalent) and/or acetaminophen/paracetamol 325 to 1000 mg at least 30 minutes prior to subsequent REGN2810 infusions.

For grade 2 symptoms (moderate reaction that requires therapy or infusion interruption but for which symptoms resolve promptly with appropriate treatment such as antihistamines, nonsteroidal anti-inflammatory drugs, narcotics, corticosteroids, and/or IV fluids; prophylactic medications indicated ≤24 hours), the following prophylactic medications are recommended for future infusions: diphenhydramine 50 mg (or equivalent) and/or acetaminophen/paracetamol 325 to 1000 mg at least 30 minutes prior to subsequent REGN2810 infusions. If necessary, corticosteroids (up to 25 mg of hydrocortisone or equivalent) may be used.

See the local prescribing information and practice guidelines for management of infusion interruptions for ipilimumab and chemotherapy agents.

7.4.1.2. Termination of the Infusion

The infusion should be terminated and NOT restarted if any of the following AEs occur:

- Anaphylaxis
- Laryngeal/pharyngeal edema
- Severe bronchospasm
- Chest pain
- Seizure
- Severe hypotension
- Other neurological symptoms (confusion, loss of consciousness, paresthesia, paralysis, etc.)
- Any other symptom or sign that, in the opinion of the investigator, warrants discontinuation of the infusion

In the event of an infusion reaction of grade 3 or greater severity during or directly following REGN2810, ipilimumab, or pembrolizumab, dosing must be stopped and the patient must be permanently discontinued from treatment.

See the local prescribing information and practice guidelines for management of infusion termination for ipilimumab and chemotherapy agents.

7.5. Method of Treatment Assignment

Each patient who signs the informed consent form (ICF) will be assigned a patient number and tracked centrally as described in the interactive voice response system (IVRS)/interactive web response system (IWRS) manual. Approximately 690 patients will be randomized in a 1:1:1 ratio according to a central randomization scheme provided by an IVRS/IWRS to the designated study pharmacist (or authorized designee). Patients will be randomized after providing informed consent, after completing screening assessments, and after the investigator has verified patient eligibility. Randomization will be stratified by histology (non-squamous versus squamous) and levels of PD-L1 expression (<1% versus 1% to <25% versus 25% to <50%).

Patients will be randomized 1:1:1 to receive standard-of-care platinum-based doublet chemotherapy, REGN2810/chemo-f, or REGN2810/chemo-l/ipi.

The choice of chemotherapy will be at the discretion of the investigator and is to be decided and documented prior to randomization.

7.5.1. Blinding/Masking

This is an open-label study. To reduce bias, endpoint assessments will be performed by an IRC blinded to treatment assignment.

7.6. Treatment Logistics and Accountability

7.6.1. Packaging, Labeling, and Storage

REGN2810

Open-label REGN2810 will be supplied as a liquid in sterile, single-use vials that will display the product lot number on the label. Each vial will contain a withdrawable volume of 5 mL or 7 mL of REGN2810 at a concentration of 50 mg/mL. REGN2810 will be refrigerated at the site at a temperature of 2°C to 8°C. The temperature of the storage refrigerator should be checked and recorded at least daily as prescribed in the pharmacy manual. Further storage instructions will be provided in the pharmacy manual.

A pharmacist or other qualified individual will be identified at each site to prepare REGN2810 for administration. Details on storage and preparation for drug product for IV administration will be provided in the pharmacy manual.

Platinum-Based Doublet Chemotherapy

Instructions on storage for chemotherapy agents will be provided in the pharmacy manual. A pharmacist or other qualified individual will be identified at each site to prepare platinum-based doublet chemotherapy for administration. Detailed preparation and administration instructions will be provided to the sites in the pharmacy manual.

Ipilimumab

Instructions on storage will be provided in the pharmacy manual. A pharmacist or other qualified individual will be identified at each site to prepare ipilimumab for administration. Detailed preparation and administration instructions will be provided to the sites in the pharmacy manual.

Ipilimumab will be refrigerated at the site at a temperature of 2°C to 8°C, and refrigerator temperature will be logged daily.

7.6.2. Supply and Disposition of Treatments

REGN2810

Study drug will be shipped at a temperature of 2ºC to 8ºC to the investigator or designee at regular intervals or as needed during the study. At specified time points during the study (eg, interim site monitoring visits), at the site close-out visit, and following drug reconciliation and

documentation by the site monitor, all opened and unopened study drug will be destroyed or returned to the sponsor or designee.

Ipilimumab

Open-label ipilimumab will be supplied locally or may be provided by Regeneron.

Platinum-Based Doublet Chemotherapy

Open-label platinum-based doublet chemotherapy agents will be supplied locally or may be provided by Regeneron.

7.6.3. Treatment Accountability

All drug accountability records must be kept current.

The investigator must be able to account for all opened and unopened study drug. These records should contain the dates, quantity, and study medication

- Dispensed to each patient,
- Returned from each patient (if applicable), and
- Disposed of at the site or returned to the sponsor or designee.

All accountability records must be made available for inspection by the sponsor and regulatory agency inspectors; anonymized photocopies must be provided to the sponsor at the conclusion of the study.

7.6.4. Treatment Compliance

All treatments will be administered at the study site, and administration will be recorded on the eCRF. All dosing records for each patient will be kept by the site.

All drug compliance records must be kept current and made available for inspection by the sponsor and regulatory agency inspectors.

7.7. Concomitant Medications

Any treatment administered from the time of informed consent until 30 days after the last study treatment will be considered concomitant medication. This includes medications that were started before the study and are ongoing during the study, as well as any therapies started in the follow-up period to treat a study-drug-related AE. All concomitant treatments must be recorded in the study eCRF with the generic name, dose, dose unit, frequency, indication, and start/stop date, as appropriate.

7.7.1. Prohibited Medications and Procedures

While participating in this study, a patient may not receive any investigational drug or treatment for treatment of a tumor other than REGN2810/chemo-l/ipi or study specified chemotherapy regimens.

Treatment with idelalisib, bevacizumab, or necitumumab is not one of the protocol–defined treatment options. If the treating physician believes that treatment with one of these 3

medications is required for a patient considering enrollment to the study and study-specified treatment options are not sufficient, they should not enroll in the study.

Any other medication that is considered necessary for the patient's welfare and is not expected to interfere with the evaluation of the REGN2810 may be given at the discretion of the investigator.

7.7.2. Permitted Medications and Procedures

It is recommended that patients do not receive concomitant systemic corticosteroids such as hydrocortisone, prednisone, prednisolone (SOLU MEDROL®), or dexamethasone (DECADRON®) at any time throughout the study, except in the case of a life-threatening emergency and/or to treat an irAE.

Physiologic replacement doses of systemic corticosteroids are permitted, even if >10 mg/day prednisone equivalents. A brief course of corticosteroids for prophylaxis (eg, contrast dye allergy) or for treatment of non-autoimmune conditions (eg, delayed-type hypersensitivity reaction caused by contact allergen) is permitted.

Treatments for bone metastases (eg, bisphosphonates, denosumab) are permitted.

Pemetrexed maintenance therapy is only permitted for patients with non-squamous histology randomized to receive pemetrexed with carboplatin or pemetrexed with cisplatin in Treatment Arm A. Pemetrexed maintenance therapy should be given according to local prescribing information and practice guidelines.

7.8. Disease Progression While Receiving REGN2810

It is recognized that a minority of patients treated with immunotherapy may derive clinical benefit despite initial evidence of progressive disease. Patients treated with REGN2810 350 mg will be permitted to continue treatment beyond initial RECIST 1.1-defined progressive disease if the investigator perceives the patient to be experiencing clinical benefit and the patient has not completed the 108-week treatment period and provided that he/she meets the following criteria:

- Investigator assessed no rapid disease progression.
- Patient continues to meet all other study eligibility criteria.
- Patient is tolerant of REGN2810 and has a stable performance status.
- Treatment beyond progression will not delay an imminent intervention to prevent serious complications of disease progression.

Imaging should be performed within 9 weeks of the initial assessment of progressive disease to determine whether there has been a decrease in the original tumor size or continued progressive disease. In these patients, further progression will be defined as an additional 10% increase in tumor burden from the time of initial progressive disease; this includes an increase in the sum of all target lesions and/or the development of new target lesions. If further progressive disease is confirmed, REGN2810 must be discontinued and other anti-cancer therapy considered, if appropriate.

If a patient continues treatment with REGN2810 beyond the initial determination of progressive disease, study assessments should continue as per Table 5.

7.9. Post-study Treatments and Procedures

Patients will be contacted quarterly by telephone for survival status, if available, until death, loss to follow-up, or study termination by the sponsor.

8. STUDY SCHEDULE OF EVENTS AND PROCEDURES

8.1. Schedule of Events

Study assessments and procedures are presented in Table 4 for screening, in Table 5 for the treatment period, and in Table 6 for the follow-up period.

CONFIDENTIAL

VV-RIM-00023209-1.0 Approved - 29 Aug 2017 GMT-5:00

Table 4: Schedule of Events: Screening Visit Assessments and Procedures

Procedure	Screening Visit (within 28 days prior to randomization)	Notes
**Eligibility Assessments**		
Informed Consent	X	Informed consent must be obtained prior to any study-related procedures. Assessments performed as part of standard-of-care that fall within the screening window (28 days prior to randomization) but before informed consent is obtained may be used for screening and need not be repeated for enrollment eligibility.
Inclusion/Exclusion Criteria	X	Eligibility of the patient must be confirmed prior to randomization. See Section 6.2 .
Collection of Tumor Tissue Sample for PD-L1 Assessment	X	• Samples should be collected as described in the laboratory manual. See Section 8.2.5.<br>• Samples (archival tissue, if ≤5 months old, or recently obtained on-study biopsy collected during screening) will be tested for PD-L1 by a central laboratory.<br>• Samples will also be tested for EGFR mutations, ALK translocations, and ROS1 fusions, unless this testing has been performed and the test results are available.
Medical/Oncology History	X	
Demographics	X	
**Efficacy Assessments**		
Baseline Radiographic Tumor Assessment	X	• CT or MRI (or PET) should be performed within 28 days of randomization.<br>• CT or MRI of the brain with contrast (unless contraindicated) should be performed in patients with a known history of treated brain metastasis, if not performed in the prior 60 days. Additional sites of known disease (including CNS) should be imaged at screening.<br>• The same imaging modality should be used throughout the study. If PET is used at baseline, it should be used throughout the study.
Baseline Tumor Burden Assessment	X	Tumor burden assessment using RECIST 1.1 criteria
**Safety Assessments**		Assessments performed as part of standard-of-care treatment that fall within the screening window (28 days prior to randomization) but before informed consent is obtained may be used for screening, and need not be repeated for enrollment eligibility.
Complete Physical Examination	X	
ECOG Performance Status	X	

**Procedure**	**Screening Visit (within 28 days prior to randomization)**	**Notes**
Weight	X	
Height	X	
Vital Signs	X	• Vital signs include temperature, seated BP, heart rate, respiration rate.<br>• BP and heart rate should be measured prior to obtaining any blood samples.
12-Lead ECG	X	A 12-lead ECG should be acquired at screening and as clinically indicated thereafter, per the discretion of the investigator.
Chest X-ray	X	
Laboratory Tests:<br>CBC with differential<br>Serum Chemistry<br>PT/PTT<br>TSH	X	• Measure free T4 if TSH is outside the normal range.<br>• TSH (and free T4 if TSH is abnormal) must be tested ≤72 hours prior to dosing ipilimumab, and the results must be reviewed prior to dosing ipilimumab.
Serum Pregnancy Test	X	Women of childbearing potential must have a serum pregnancy test performed within the 72 hours prior to administration of the first dose of study drug.
Prior/Concomitant Medication Recording	X	
Adverse Event Recording	X	Assess using current version of NCI-CTCAE.
**Genomics Sub-study**		
Genomics Sub-Study Consent (Optional)	X	DNA consent should be obtained during the screening period but may be obtained any time throughout the study

Abbreviations: ALK=anaplastic lymphoma kinase; BP=blood pressure; CBC=complete blood count; CNS=central nervous system; CT=computerized tomography; DNA=deoxyribonucleic acid; ECG=electrocardiogram; EGFR=epidermal growth factor receptor; ECOG=Eastern Cooperative Oncology Group; MRI=magnetic resonance imaging; PD-L1=programmed cell death ligand 1; PET=positron emission tomography; PT=prothrombin time; PTT=partial thromboplastin time; RECIST 1.1=Response Evaluation Criteria in Solid Tumors version 1.1; ROS1=C-ros oncogene receptor tyrosine kinase; T4= thyroxine; TSH=thyroid-stimulating hormone

Table 5: Schedule of Events: Treatment Period Assessments and Procedures

Study Procedure	Year 1				Year 2 (Starting at Cycle 19)			Notes
	Day 1, Cycle 1	Day 1, Each Cycle (Every 21 Days ±3 Days)	Day 1, Every Other Cycle (Every 42 Days ±3 Days)	Every 3 Cycles (9 Weeks [63 Days] ±5 Days)	Day 1, Each Cycle (Every 21 Days ±3 Days)	Every 3 Cycles (9 Weeks [63 Days] ±5 Days)	Every 4 Cycles (12 Weeks ±5 Days)	
Randomization	X							
**Study Treatment Administration**								Treatment should be initiated within 3 days of randomization.
**Treatment Arm A - One of the of the following platinum-based doublet chemotherapy**								
Gemcitabine 1250 mg/m² IV plus carboplatin AUC of 5 or 6 mg/mL/min IV<br>OR<br>Gemcitabine 1250 mg/m² IV plus cisplatin 100 mg/m² IV<br>OR<br>Paclitaxel 200 mg/m² IV plus carboplatin AUC of 5 or 6 mg/mL/min IV<br>OR<br>Paclitaxel 200 mg/m² IV plus cisplatin 75 mg/m² IV<br>OR<br>Pemetrexed 500 mg/m² IV plus carboplatin AUC of 5 or 6 mg/mL/min IV (non-squamous histology only)<br>OR<br>Pemetrexed 500 mg/m² IV plus cisplatin 75 mg/m² IV (non-squamous histology only)	X	X						• Record start and stop infusion times.<br>• Administer 4 to 6 cycles.<br>• Administer on day 1 of each 21-day cycle (Q3W). For patients receiving gemcitabine and cisplatin, administer on day 1 of each 21-day cycle (both gemcitabine and cisplatin) and day 8 (gemcitabine only) of each 21-day cycle (Q3W).<br>• Calculate dose of carboplatin using the Calvert formula.<br>• Note that patients with GFR <50 mL/min/1.73m² may NOT receive cisplatin-containing regimens.<br>• Pemetrexed maintenance is optional and for Treatment Arm A only; pemetrexed maintenance should be according to local prescribing information and practice guidelines.

CONFIDENTIAL

VV-RIM-00023209-1.0 Approved - 29 Aug 2017 GMT-5:00

<table>
<tr><th rowspan="2">Study Procedure</th><th colspan="4">Year 1</th><th colspan="3">Year 2 (Starting at Cycle 19)</th><th rowspan="2">Notes</th></tr>
<tr><th>Day 1, Cycle 1</th><th>Day 1, Each Cycle (Every 21 Days ±3 Days)</th><th>Day 1, Every Other Cycle (Every 42 Days ±3 Days)</th><th>Every 3 Cycles (9 Weeks [63 Days] ±5 Days)</th><th>Day 1, Each Cycle (Every 21 Days ±3 Days)</th><th>Every 3 Cycles (9 Weeks [63 Days] ±5 Days)</th><th>Every 4 Cycles (12 Weeks ±5 Days)</th></tr>
<tr><td colspan="9">Treatment Arm B - REGN2810/chemo-f</td></tr>
<tr><td>REGN2810 350 mg</td><td>X</td><td>X</td><td></td><td></td><td>X</td><td></td><td></td><td>• Record start and stop infusion times.
• Infuse REGN2810 after chemotherapy.</td></tr>
<tr><td>Gemcitabine 1250 mg/m^2 IV plus carboplatin AUC of 5 or 6 mg/mL/min IV
OR
Gemcitabine 1250 mg/m^2 IV plus cisplatin 100 mg/m^2 IV
OR
Paclitaxel 200 mg/m^2 IV plus carboplatin AUC of 5 or 6 mg/mL/min IV
OR
Paclitaxel 200 mg/m^2 IV plus cisplatin 75 mg/m^2 IV
OR
Pemetrexed 500 mg/m^2 IV plus carboplatin AUC of 5 or 6 mg/mL/min IV (non-squamous histology only)
OR
Pemetrexed 500 mg/m^2 IV plus cisplatin 75 mg/m^2 IV (non-squamous histology only)</td><td>X</td><td>X</td><td colspan="6">• Record start and stop infusion times.
• Administer 4 to 6 cycles.
• Administer on day 1 (both gemcitabine and carboplatin) and day 8 (gemcitabine only) of each 21-day cycle (Q3W).
• Calculate dose of carboplatin using the Calvert formula.
• Note that patients with GFR <50 mL/min/1.73m^2 may NOT receive cisplatin-containing regimens.</td></tr>
</table>

CONFIDENTIAL

VV-RIM-00023209-1.0 Approved - 29 Aug 2017 GMT-5:00

Study Procedure	Year 1				Year 2 (Starting at Cycle 19)			Notes
	Day 1, Cycle 1	Day 1, Each Cycle (Every 21 Days ±3 Days)	Day 1, Every Other Cycle (Every 42 Days ±3 Days)	Every 3 Cycles (9 Weeks [63 Days] ±5 Days)	Day 1, Each Cycle (Every 21 Days ±3 Days)	Every 3 Cycles (9 Weeks [63 Days] ±5 Days)	Every 4 Cycles (12 Weeks ±5 Days)	
**Treatment Arm C - REGN2810/chemo-l/ipi**								
REGN2810 350 mg	X	X			X			• Record start and stop infusion times.<br>• Infuse chemotherapy first, then REGN2810 followed by ipilimumab on the same day.
Gemcitabine 1250 mg/m^2 IV plus carboplatin AUC of 5 or 6 mg/mL/min IV<br>OR<br>Gemcitabine 1250 mg/m^2 IV plus cisplatin 100 mg/m^2 IV<br>OR<br>Paclitaxel 200 mg/m^2 IV plus carboplatin AUC of 5 or 6 mg/mL/min IV<br>OR<br>Paclitaxel 200 mg/m^2 IV plus cisplatin 75 mg/m^2 IV<br>OR<br>Pemetrexed 500 mg/m^2 IV plus carboplatin AUC of 5 or 6 mg/mL/min IV (non-squamous histology only)<br>OR<br>Pemetrexed 500 mg/m^2 IV plus cisplatin 75 mg/m^2 IV (non-squamous histology only)	X	X	• Record start and stop infusion times.<br>• Administer 2 cycles.<br>• Administer on day 1 (both gemcitabine and carboplatin) and day 8 (gemcitabine only) of each 21-day cycle (Q3W).<br>• Calculate dose of carboplatin using the Calvert formula.<br>• Note that patients with GFR <50 mL/min/1.73m^2 may NOT receive cisplatin-containing regimens.					

Study Procedure	Year 1				Year 2 (Starting at Cycle 19)			Notes
	Day 1, Cycle 1	Day 1, Each Cycle (Every 21 Days ±3 Days)	Day 1, Every Other Cycle (Every 42 Days ±3 Days)	Every 3 Cycles (9 Weeks [63 Days] ±5 Days)	Day 1, Each Cycle (Every 21 Days ±3 Days)	Every 3 Cycles (9 Weeks [63 Days] ±5 Days)	Every 4 Cycles (12 Weeks ±5 Days)	
Ipilimumab 50 mg	X		X					• Record start and stop infusion times.<br>• Administer Q6W for up to 4 doses.<br>• Infuse after REGN2810 on the same day.
**Efficacy Assessments**								
Radiographic Tumor Assessment				X			X	• For schedule, see Section 8.2.2.1.<br>• Image with contrast (unless contraindicated) the chest/abdomen/pelvis and other areas being monitored.<br>• Regardless of when patients in Treatment Arm A enter the follow-up period, the radiographic tumor assessment schedule outlined in Section 8.2.2.1 should be followed, until RECIST 1.1-defined progressive disease, withdrawal of consent, death, or initiation of another anti-cancer treatment.<br>• Brain scans during the treatment and follow-up periods should be performed as clinically indicated except for patients with a history of metastases, who should have surveillance imaging approximately Q18W for year 1 and Q24W for year 2 or sooner, if indicated.<br>• For patients who have RECIST 1.1-defined progressive disease while receiving REGN2810 or after completion of REGN2810

CONFIDENTIAL

VV-RIM-00023209-1.0 Approved - 29 Aug 2017 GMT-5:00

**Study Procedure**	**Year 1**				**Year 2 (Starting at Cycle 19)**			**Notes**
	**Day 1, Cycle 1**	**Day 1, Each Cycle (Every 21 Days ±3 Days)**	**Day 1, Every Other Cycle (Every 42 Days ±3 Days)**	**Every 3 Cycles (9 Weeks [63 Days] ±5 Days)**	**Day 1, Each Cycle (Every 21 Days ±3 Days)**	**Every 3 Cycles (9 Weeks [63 Days] ±5 Days)**	**Every 4 Cycles (12 Weeks ±5 Days)**	
								treatment, imaging should be performed within 9 weeks of the original tumor progression.
Tumor Burden Assessment				X			X	Tumor burden assessment per RECIST 1.1 criteria
Quality of Life Questionnaires	X	X		X	X	X		Complete prior to any study procedures. Complete on day 1 of every cycle for the first 6 doses and then on day 1 of every 3 cycles.
**Safety Assessments**								
Physical Examination	X	X			X			A PE may be performed ≤72 hours prior to dosing on the day 1 visit of each cycle. A complete PE is to be performed prior to the first dose. A limited PE should be performed at all other visits, but a complete PE may be performed, if indicated. PE definitions are provided in Section 8.2.3.1.
ECOG Performance Status	X	X			X			
Vital Signs (Seated Blood Pressure, Heart Rate, Respiratory Rate, Temperature)	X	X			X			At cycle 1 day 1 and on all subsequent treatment days, vital signs will be collected prior to infusion of treatment. Vital signs must also be obtained approximately 15 minutes (±10 minutes) after completion of the infusion. See Section 8.2.3.4.
12-Lead ECG	(X)	(X)			(X)			A 12-lead ECG should be acquired as clinically indicated, per the discretion of the investigator.
Hematology (CBC With Differential)	X	X			X			• Blood samples may be collected ≤72 hours prior to dosing on the

CONFIDENTIAL

VV-RIM-00023209-1.0 Approved - 29 Aug 2017 GMT-5:00

Study Procedure	Year 1				Year 2 (Starting at Cycle 19)			Notes
	Day 1, Cycle 1	Day 1, Each Cycle (Every 21 Days ±3 Days)	Day 1, Every Other Cycle (Every 42 Days ±3 Days)	Every 3 Cycles (9 Weeks [63 Days] ±5 Days)	Day 1, Each Cycle (Every 21 Days ±3 Days)	Every 3 Cycles (9 Weeks [63 Days] ±5 Days)	Every 4 Cycles (12 Weeks ±5 Days)	
								day 1 visit of each cycle.<br>• Results must be obtained/reviewed prior to dosing.<br>• Screening laboratory examinations performed within 7 days of cycle 1 day 1 do not need to be repeated for this visit, unless clinically indicated.
Serum Chemistry	X	X	X (ACTH must be tested in Treatment Arm C; only prior to ipilimumab dosing)		X			• Blood sample may be collected ≤72 hours prior to dosing on the day 1 visit of each cycle.<br>• Results must be obtained/reviewed prior to dosing.<br>• Screening laboratory examinations performed within 7 days of cycle 1 day 1 do not need to be repeated for this visit, unless clinically indicated.
Coagulation Testing	(X)	(X)			(X)			As clinically indicated
Pregnancy Testing	X	X			X			Women of childbearing potential must have a negative serum pregnancy test within 72 hours prior to study treatment administration on cycle 1 day 1 and a negative urine pregnancy test prior to study treatment administration on day 1 of each subsequent treatment cycle and Q6W, or more frequently per local standard.
TSH	X		X (Treatment Arm C; only prior to ipilimumab dosing)					• For **Treatment Arm C**, TSH (and free T4 if TSH is abnormal) **must** be tested ≤72 hours prior to dosing ipilimumab and the results must be reviewed prior to dosing ipilimumab.

CONFIDENTIAL

VV-RIM-00023209-1.0 Approved - 29 Aug 2017 GMT-5:00

Study Procedure	Year 1				Year 2 (Starting at Cycle 19)			Notes
	Day 1, Cycle 1	Day 1, Each Cycle (Every 21 Days ±3 Days)	Day 1, Every Other Cycle (Every 42 Days ±3 Days)	Every 3 Cycles (9 Weeks [63 Days] ±5 Days)	Day 1, Each Cycle (Every 21 Days ±3 Days)	Every 3 Cycles (9 Weeks [63 Days] ±5 Days)	Every 4 Cycles (12 Weeks ±5 Days)	
								• For all treatment arms, may be obtained again, as clinically indicated.<br>• Measure free T4 if TSH is abnormal.
Concomitant Medication Recording	X	X			X			
Adverse Event Recording	X	X			X			Assess using current version of NCI-CTCAE.
**PK Drug Concentration/ Anti-Drug Antibody Procedures**								For footnotes, see Section 8.1.1
**Treatment Arms B and C:** Pharmacokinetic Drug Concentration Measurements and Samples	X	X					X	Collect pre-dose[1] and at the end of infusion on day 1 of cycle 1; collect pre-infusion[2] and at the end of infusion on day 1 of cycle 2 through cycle 6, cycle 9, cycle 12, cycle 15, and cycle 18, then every 4 cycles in year 2, and at end of treatment (see Appendix 4).
**Treatment Arms B and C:** Anti-Drug Antibody Measurements and Samples	X	X					X	Collect pre-dose[1] on day 1 of cycle 1 and pre-infusion[2] on day 1 of cycle 6, cycle 9, and cycle 18, then every 8 cycles in year 2 (see Appendix 4).
**Biomarker Procedures**								
Serum Biomarker Sample	X	X						Samples will be collected prior to drug administration on day 1 of cycle 1, cycle 2, and cycle 4, and at the time of RECIST 1.1-defined progressive disease (see Appendix 4).
Plasma Biomarker Sample	X	X						Samples will be collected prior to drug administration on day 1 of cycle 1,

CONFIDENTIAL

VV-RIM-00023209-1.0 Approved - 29 Aug 2017 GMT-5:00

**Study Procedure**	**Year 1**				**Year 2 (Starting at Cycle 19)**			**Notes**
	**Day 1, Cycle 1**	**Day 1, Each Cycle (Every 21 Days ±3 Days)**	**Day 1, Every Other Cycle (Every 42 Days ±3 Days)**	**Every 3 Cycles (9 Weeks [63 Days] ±5 Days)**	**Day 1, Each Cycle (Every 21 Days ±3 Days)**	**Every 3 Cycles (9 Weeks [63 Days] ±5 Days)**	**Every 4 Cycles (12 Weeks ±5 Days)**	
								cycle 2, and cycle 4, and at the time of RECIST 1.1-defined progressive disease (see Appendix 4).
Genomics Sub-Study: Blood Sample for Germline DNA (Optional)	X							Collect the blood sample for DNA at day 1 of cycle 1. If consent is not obtained during screening, it can be obtained at any other visit prior to collection of the sample.
**Tumor Biopsy**								
Tumor Biopsy (Optional)		(X)			(X)			A tumor biopsy should be collected at the time of RECIST 1.1-defined progressive disease (optional) as described in the laboratory manual.

Parentheses indicate assessments which are optional.
Abbreviations: REGN2810/chemo-f=REGN2810 in combination with standard-of-care platinum-based doublet chemotherapy; REGN2810/chemo-l/ipi=REGN2810 in combination with initial standard-of-care platinum-based doublet chemotherapy and ipilimumab; ACTH=adrenocorticotropic hormone; ADA=anti-drug antibody; CBC=complete blood count; AUC=area under the curve; DNA=deoxyribonucleic acid; ECG=electrocardiogram; ECOG=Eastern Cooperative Oncology Group; GFR=glomerular filtration rate; IV=intravenous; NCI-CTCAE=National Cancer Institute Common Terminology Criteria for Adverse Events; PD=progressive disease; PE=physical examination; PK=pharmacokinetic; Q3W=every 3 weeks; Q6W=every 6 weeks; Q9W=every 9 weeks; Q12W=every 12 weeks; Q18W=every 18 weeks; Q24W=every 24 weeks; RECIST 1.1=Response Evaluation Criteria in Solid Tumors version 1.1; T4=thyroxine; TSH=thyroid-stimulating hormone

Table 6: Schedule of Events: Follow-Up Period Assessments and Procedures

Study Procedure	Follow-up Visit 1/End of study visit for patients who discontinued treatment due to PD	Follow-up Visits 2-7	Notes
	**14 to 30 Days (±7 days) after the last study treatment if treatment is discontinued due to PD as an end of study visit OR Last Cycle Visit + 14 to 30 Days (±7 Days) if treatment is discontinued for any other reason**	**Prior Follow-up Visit + 28 Days (± 7 Days)**	
**Efficacy Assessments**			
Radiographic Tumor Assessment		X	• Radiographic assessments for patients in **Treatment Arms B and C** should be according to the schedule outlined in Section 8.2.2.1.<br>• Regardless of when patients in Treatment Arm A enter the follow-up period, the radiographic tumor assessment schedule outlined in Section 8.2.2.1 should be followed, until RECIST 1.1-defined progressive disease, withdrawal of consent, death, or initiation of another anti-cancer treatment.
Tumor Burden Assessment		X	Tumor burden assessment using RECIST 1.1 criteria
Quality of Life Questionnaires	X		Complete prior to any study procedures.
Survival Data Collection	X	X	Every 3 months, until death, loss to follow-up, or withdrawal of study consent. May be performed by phone contact or office visit.
**Safety Assessments**			
Physical Examination	X	X	A limited PE may be performed but a complete PE should be performed when clinically indicated
ECOG Performance Status	X	X	
Weight	X		
Vital Signs	X	X	• Vital signs including temperature, seated BP, RR, heart rate<br>• BP and heart rate should be measured prior to obtaining any blood samples.
Hematology (CBC With Differential)	X		Collect at follow-up visit 1 and then as clinically indicated.
Serum Chemistry	X		Collect at follow-up visit 1 and then as clinically indicated.
Coagulation Testing			Collect as clinically indicated.
Pregnancy Test (urine)	X		Women of childbearing potential only.
Concomitant Medication Recording	X	X	

CONFIDENTIAL

VV-RIM-00023209-1.0 Approved - 29 Aug 2017 GMT-5:00

Study Procedure	Follow-up Visit 1/End of study visit for patients who discontinued treatment due to PD	Follow-up Visits 2-7	Notes
	**14 to 30 Days (±7 days) after the last study treatment if treatment is discontinued due to PD as an end of study visit OR Last Cycle Visit + 14 to 30 Days (±7 Days) if treatment is discontinued for any other reason**	**Prior Follow-up Visit + 28 Days (± 7 Days)**	
Adverse Event Recording	X	X	Assess using current version of NCI-CTCAE. All AEs after initiation of study treatment and until 90 days after the last study treatment, regardless of relationship to study treatment, will be reported on the AE eCRF. See Section 9.4.1.
**PK Drug Concentration/ Anti-Drug Antibody Samples (blood)**			
**Treatment Arms B and C:** Pharmacokinetic Drug Concentration Measurements and Samples	X	X	Samples will be collected at follow-up visits 1 and 3.
**Treatment Arms B and C:** Anti-Drug Antibody Measurements and Samples	X	X	Samples will be collected at follow-up visits 1 and 3.
**Biomarker Samples**			
Serum Biomarker Sample	X		Samples will be collected at follow-up visit 1 and at the time of progressive disease.
Plasma Biomarker Sample	X		Samples will be collected at follow-up visit 1 and at the time of progressive disease.
**Tumor Biopsy**			
Tumor Biopsy (Optional)			A tumor biopsy should be collected at the time of RECIST 1.1-defined progressive disease (optional) as described in the laboratory manual.

Abbreviations: CBC=complete blood count; ECOG=Eastern Cooperative Oncology Group; NCI-CTCAE=National Cancer Institute Common Terminology Criteria for Adverse Events; PD=progressive disease; PE=physical examination; PK=pharmacokinetic; Q12W=every 12 weeks; RECIST 1.1=Response Evaluation Criteria in Solid Tumors version 1.1

CONFIDENTIAL

VV-RIM-00023209-1.0 Approved - 29 Aug 2017 GMT-5:00

8.1.1. Footnotes for the Schedule of Events Table

Footnotes for the Schedule of Events Tables are as follows:

1. Pre-dose is defined as before the start of the first REGN2810 infusion (specific to PK drug concentration and ADA samples in Table 5). Pre-dose samples may be collected ≤72 hours prior to day 1 dosing.
2. Pre-infusion is defined as before the start of subsequent REGN2810 infusions (specific to PK drug concentration and ADA samples in Table 5).

8.1.2. Early Termination Visit

Patients who withdraw from the study during the treatment period will be asked to return to the clinic to complete follow-up visit 1 study assessments (Table 6) as an early termination visit. Patients who withdraw from the study during the follow-up period will be asked to return to the clinic to complete visits of the follow-up period as indicated in Table 6.

Patients who discontinue study treatment due to progressive disease should return to the clinic 14 to 30 days after the last study treatment to complete the end of study assessments (follow-up visit1).

Patients who discontinue study treatment for a reason other than progressive disease should return to the clinic 14 to 30 days (±7 days) after the last cycle visit for follow-up visit 1 and then continue with follow-up visit 2 through follow-up visit 7.

8.1.3. Unscheduled Visits

All attempts should be made to keep patients on the study schedule. Unscheduled visits may be necessary to repeat testing following abnormal laboratory results, for follow-up of AEs, or for any other reason, as warranted. In response to adverse event of special interests (AESIs), such as anaphylaxis or hypersensitivity, ADA samples may be collected closer to the event, based on the judgment of the investigator and/or Medical Monitor.

8.2. Study Procedures

8.2.1. Procedures Performed Only at the Screening/Baseline Visit

Informed consent must be obtained prior to any study-related procedures. Assessments performed as part of standard-of-care that fall within the screening window (28 days prior to randomization) but before informed consent is obtained may be used for screening and need not be repeated for enrollment eligibility.

The following procedures will be performed for the sole purpose of determining study eligibility or characterizing the baseline population:

- Collection of tumor tissue sample for PD-L1 assessment by the central laboratory
  - A formalin-fixed, paraffin-embedded tissue block or unstained slide of tumor tissue sample (archival tissue, if ≤5 months old, or recently obtained, on-study tumor biopsy collected at screening) must be provided. Tumor biopsies should be of sufficient size to ensure an adequate amount of tissue for analysis (excisional,

VV-RIM-00023209-1.0 Approved - 29 Aug 2017 GMT-5:00

incisional, or core needle; fine needle aspirates are not acceptable). Complete instructions on the collection, processing, handling, and shipment of all samples will be provided in the laboratory manual.

  - Tumor tissue samples will also be tested for EGFR mutations and ALK translocations as well as for ROS1 fusions, unless these testing has already been performed and the results are available.

- Baseline radiographic tumor assessment of the chest, abdomen, pelvis, and all other known or suspected sites of disease by CT or MRI (or positron emission tomography). The same imaging modality should be used throughout the study. If PET is used at baseline, it should be used throughout the study.
- Baseline tumor burden assessment
- Baseline weight and height
- Serum pregnancy test in women of childbearing potential within 72 hours prior to administration of the first study treatment administration
- Baseline 12-Lead ECG and chest X-ray
- Sample for a genomics sub-study (optional)

For the complete list of procedures performed at screening to determine eligibility, including those that are used throughout the study (ie, not only for determining eligibility), see Table 4.

8.2.2. Efficacy Procedures

8.2.2.1. Radiographic Tumor Assessments

High-resolution CT with contrast and contrast-enhanced MRI are the preferred imaging modalities for assessing radiographic tumor response. In patients whom contrast is strictly contraindicated, non-contrast scans will suffice. The chest, abdomen, and pelvis must be imaged along with any other known or suspected sites of disease. If more than 1 imaging modality is used at screening, the most accurate imaging modality according to RECIST 1.1 should be used when recording data. The same imaging modality used at screening should be used for all subsequent assessments.

At screening, CT or MRI of the brain with contrast (unless contraindicated) should be performed in patients with a known history of treated brain metastasis, if not performed in the prior 60 days. Additional sites of known disease (including CNS) should be imaged at screening.

After the baseline tumor assessment, radiographic tumor assessments will be obtained in all patients Q9W beginning at week 9 (day 63 ± 5 days) during year 1 and Q12W beginning at week 55 (first radiographic tumor assessment in year 2 performed at end of week 54) during year 2, until IRC-assessed RECIST 1.1-defined progressive disease, withdrawal of consent, death, or initiation of another anti-cancer treatment. Patients in Treatment Arm A are treated with 4 to 6 cycles of chemotherapy and optional pemetrexed maintenance. However, regardless of when patients in Treatment Arm A enter the follow-up period, the following radiographic tumor assessment schedule should be followed, until RECIST 1.1-defined progressive disease, withdrawal of consent, death, or initiation of another anti-cancer treatment. Patients who

VV-RIM-00023209-1.0 Approved - 29 Aug 2017 GMT-5:00

discontinue for reasons other than progression who are not attending treatment visits may have radiographic tumor assessments between Q9W and Q12W until RECIST 1.1-defined progressive disease, withdrawal of consent, death, or initiation of another anti-cancer treatment.

Radiographic tumor assessments will occur at the following time points:

End of week 9 ± 5 days (end of cycle 3)
End of week 18 ± 5 days (end of cycle 6)
End of week 27 ± 5 days (end of cycle 9)
End of week 36 ± 5 days (end of cycle 12)
End of week 45 ± 5 days (end of cycle 15)
End of week 54 ± 5 days (end of cycle 18)
End of week 66 ± 5 days (end of cycle 22)
End of week 78 ±5 days (end of cycle 26)
End of week 90 ± 5 days (end of cycle 30)
End of week 102 ± 5 days (end of cycle 34)

In the follow-up period, radiographic assessments for all patients who have not experienced progressive disease should be performed Q12W or until RECIST 1.1-defined progressive disease, withdrawal of consent, death, or initiation of another anti-cancer treatment.

Tumor assessments should be performed even if dosing is interrupted. Weeks are in reference to the calendar week and should not be adjusted due to dosing delays/interruptions.

Brain scans during the treatment and follow-up periods should be performed as clinically indicated except for patients with a history of metastases, who should have surveillance imaging approximately every 18 weeks for year 1 and every 24 weeks for year 2 or sooner, if indicated.

For patients identified as having RECIST 1.1-defined progressive disease while receiving or after completion of study treatment, subsequent imaging should be performed within 9 weeks of the initial tumor assessment of progression.

8.2.2.2. Tumor Burden Assessments

Tumor measurements will be performed in accordance with RECIST 1.1 criteria (Eisenhauer 2009; Appendix 2) and should be done by the same investigator or radiologist for each assessment, to the extent feasible.

Investigators and the blinded IRC (Section 5.3.1) will assess response to therapy using RECIST 1.1 criteria. RECIST 1.1-defined progressive disease determined by the investigator will be used for clinical management of the patient. RECIST 1.1-based tumor burden assessments by the blinded IRC will be used for evaluation of efficacy endpoints.

8.2.2.3. Quality of Life Questionnaires

Patient-reported outcomes will be measured at the frequency indicated in Table 5 and Table 6 using the following validated patient self-administered questionnaires: EORTC QLQ-C30 and EORTC QLQ-LC13 (Bergman 1994, Bjordal 2000). Patients will be asked to complete these questionnaires prior to any study procedures being performed at a given study visit (during the treatment and follow-up periods).

8.2.2.4. Survival Data Collection

Every effort will be made to collect survival data on all patients, including patients who withdraw from the study for any reason but have not withdrawn consent to collect survival information, as indicated in Table 6. If the death of a patient is not reported, the date of the last patient contact in this study will be used in the determination of the patient's last known date alive. This will be completed by telephone contact or an office visit.

8.2.3. Safety Procedures

8.2.3.1. Physical Examination

A complete or limited PE will be performed at the visits specified in Table 4, Table 5, and Table 6. Care should be taken to examine and assess any abnormalities that may be present, as indicated by the patient's medical history.

Complete PE will be performed prior to the first dose on cycle 1 day 1, or in other visits if indicated, and will include examination of the skin, head, eyes, nose, throat, neck, joints, lungs, heart, pulse, abdomen (including liver and spleen), lymph nodes, and extremities, as well as a brief neurologic examination.

Limited physical examination will include, at least, examination of the lungs, heart, abdomen, and skin.

8.2.3.2. ECOG Performance Status

Eastern Cooperative Oncology Group performance status will be measured at a frequency indicated in Table 4, Table 5, and Table 6.

8.2.3.3. Weight and Height

Body weight measurements will be obtained at screening and at follow-up according to Table 4 and Table 6. Weight should be obtained with the patient wearing undergarments or very light clothing, with no shoes, and with an empty bladder. The same scale should be used throughout the study. The use of calibrated balance scales is recommended, if possible. Self-reported weights are not acceptable.

Height should be measured at screening; self-reported heights are not acceptable.

8.2.3.4. Vital Signs

Vital signs, including temperature, seated blood pressure, heart rate, and respiratory rate, will be collected at time points according to Table 4, Table 5, and Table 6. Vital signs should be performed before blood is drawn during visits requiring blood draws.

Blood pressure should be measured in the same arm at all study visits (when feasible) and after the patient has been resting quietly in the seated position for at least 5 minutes.

At cycle 1 day 1 and on all subsequent treatment days, vital signs will be collected prior to infusion of treatment. Vital signs should also be obtained approximately 15 minutes (± 10 minutes) after completion of the infusion.

8.2.3.5. 12-Lead Electrocardiogram

A standard 12-lead ECG will be performed at screening and when clinically indicated, per the discretion of the investigator, while during the active treatment period (Table 4 and Table 5). Electrocardiograms (ECGs) should be performed before blood is drawn during visits requiring blood draws.

The patient should be relaxed and in a recumbent position for at least 5 minutes before recording an ECG. The ECG will be reviewed by the investigator or an authorized designee at the site and will be available for comparison with subsequent ECGs. Heart rate will be recorded from the ventricular rate, and the PR, QRS, RR, QT, and QT corrected for Bazett's formula intervals will also be recorded. The ECG tracing will be retained with the source.

Any ECG finding that is judged by the investigator as a clinically significant change (worsening) compared with the baseline value will be considered an AE, recorded, and monitored.

8.2.3.6. Laboratory Testing

Hematology, chemistry, and pregnancy testing samples will be analyzed by the site's local laboratory.

Samples for laboratory testing will be collected at time points according to according to Table 4, Table 5, and Table 6. Tests will include the following:

Hematology (CBC with Differential)

Hemoglobin	Differential:
Hematocrit	Neutrophils
Red blood cells	Lymphocytes
White blood cells	Monocytes
Red cell indices	Basophils
Platelet count	Eosinophils

For hematology, blood samples may be collected ≤72 hours prior to dosing on the day 1 visit of each cycle. Results must be obtained/reviewed prior to dosing.

Serum Chemistry

Sodium	Total protein, serum	Total bilirubin
Potassium	Creatinine	Magnesium
Chloride	Blood urea nitrogen [a]	Phosphorus
Bicarbonate [b]	Aspartate aminotransferase	Uric acid
Calcium	Alanine aminotransferase	Adrenocorticotropic hormone[c]
Glucose	Alkaline phosphatase	
Albumin	Lactate dehydrogenase	

[a] The urea test is acceptable instead of blood urea nitrogen at centers where this is commonly used instead of the blood urea nitrogen.

[b] The partial pressure of carbon dioxide (PCO_2) test is an acceptable test at centers where this is commonly used instead of the bicarbonate test.

[c] Adrenocorticotropic hormone (ACTH) will be included only for the 4 cycles when ipilimumab is administered and only in Treatment Arm C.

For chemistry, blood sample may be collected ≤72 hours prior to dosing on the day 1 visit of each cycle. Results must be obtained/reviewed prior to dosing. For Treatment Arm C, ACTH should be tested during the 4 cycles ipilimumab is administered.

VV-RIM-00023209-1.0 Approved - 29 Aug 2017 GMT-5:00

Other Laboratory Tests

Coagulation Tests: Prothrombin time/partial thromboplastin time will be analyzed by the site's local laboratory. Testing will be performed at screening and then as clinically indicated.

Thyroid Function Tests: Thyroid-stimulating hormone will be analyzed by the site's local laboratory. For Treatment Arm C, TSH (and free thyroxine [T4] if TSH is abnormal) must be tested ≤72 hours prior to dosing ipilimumab, and the results must be reviewed prior to dosing ipilimumab. For Treatment Arm C, on cycles when ipilimumab is not dosed, and all Treatment Arms A and B patients, TSH is tested as clinically indicated. If TSH is abnormal, a free T4 should be measured at the investigative site's local laboratory.

Pregnancy Testing

Women of childbearing potential must have a negative serum pregnancy test within the 72 hours prior to study treatment administration on cycle 1 day 1 and a negative urine pregnancy test prior to study treatment administration on day 1 of each subsequent treatment cycle and Q6W, or more frequently, per local standard.

Abnormal Laboratory Values and Laboratory Adverse Events

- All laboratory values must be reviewed by the investigator or an authorized designee.
- Significantly abnormal test results that occur after start of treatment must be repeated to confirm the nature and degree of the abnormality. When necessary, appropriate ancillary investigations should be initiated. If the abnormality fails to resolve or cannot be explained by events or conditions unrelated to the study medication or its administration, the Medical Monitor must be consulted.
- The clinical significance of an abnormal test value, within the context of the disease under study, must be determined by the investigator.

Criteria for reporting laboratory values as an AE are provided in Section 9.4.5.

8.2.3.7. Concomitant Medication Recording

Concomitant medication will be collected at time points according to Table 4, Table 5, and Table 6. See Section 7.7 for details on recording concomitant medications.

8.2.3.8. Adverse Event Recording

Adverse events will be collected at time points according to according to Table 4, Table 5, and Table 6. See Section 9.4 for details on recording and reporting AEs.

8.2.4. Pharmacokinetic and Anti-Drug Antibody Procedures

In addition to the procedures detailed below, blood samples will also be taken to measure drug concentrations/ADA, as appropriate, in case of AESIs.

8.2.4.1. Drug Concentration Measurements and Samples

REGN2810 concentrations in the sera of patients randomized to Treatment Arms B and C will be measured using a validated enzyme-linked immunosorbent assay method at visits and time points indicated in Table 5, Table 6, and Appendix 4. The actual time of each blood draw must be

recorded. Pre-dose is defined as before the start of the first REGN2810 infusion. Pre-dose samples may be collected ≤72 hours prior to day 1 dosing. Pre-infusion is defined as before the start of subsequent REGN2810 infusions.

In addition, measurement of ipilimumab concentrations in serum may be considered in the future in the PK samples of patients randomized to Treatment Arm C.

Any unused samples collected for drug concentration measurements may be used for exploratory biomarker research.

8.2.4.2. Anti-Drug Antibody Measurements and Samples

Anti-drug antibody samples for REGN2810 immunogenicity assessments will be collected from patients randomized to the Treatment Arms B and C prior to dosing at time points listed in Table 5, during the follow-up period as shown in Table 6, and as indicated in Appendix 4. Any unused samples collected for immunogenicity assessments may be used for exploratory research or to investigate unexpected AEs.

8.2.5. Biomarker Procedures

For biomarker assessments, a formalin-fixed, paraffin-embedded tissue block or unstained slides of tumor tissue biopsy samples (archival tissue, if ≤5 months old, or recently obtained biopsy collected during on-study) must be provided. Tumor tissue biopsy samples should be of sufficient size to ensure an adequate amount of tissue for analysis (excisional, incisional, or core needle; fine needle aspirates are not acceptable). Complete instructions on the collection, processing, handling, and shipment of all samples will be provided in the laboratory manual.

With the use of the collected tumor tissue samples, the following biomarker-based stratification strategies will be implemented in this study to determine study eligibility:

- A PD-L1 IHC 22C3 pharmDx assay will be utilized as the clinical trial assay to assess PD-L1 expression levels in recently obtained tumor tissue samples (on-study biopsy collected at screening visit or archival tissue, if ≤5 months old) in order to qualify patients for enrollment. The ≥50% PD-L1 positivity cut-off will be used as an inclusion criterion for this study.
- The PD-L1 IHC 22C3 pharmDx assay is a validated, automated, in vitro diagnostic assay that was developed as a companion diagnostic for pembrolizumab (Roach 2016). During development, the PD-L1 IHC 22C3 pharmDx assay was analytically validated for repeatability and reproducibility at 3 independent Clinical Laboratory Improvement Amendments (CLIA)-certified laboratories and clinically validated in KEYNOTE-001, a clinical study of pembrolizumab in patients with NSCLC. Based on these studies, the PD-L1 IHC 22C3 pharmDx assay was demonstrated to be a robust PD-L1 assay and was approved by the FDA as a companion diagnostic for pembrolizumab. Further details on the assay are provided in the package insert (Dako, PD-L1 IHC 22C3 pharmDx Package Insert).

Tumor tissue biopsy samples will also be tested for EGFR mutations, ALK translocations, as well as for ROS1 fusions for determination of study eligibility. This testing does not need to be repeated if it has been done previously and the results are available. The results must indicate

that the patient is negative for EGFR mutations, ALK translocations, and ROS1 fusions for enrollment in this study.

Additional testing may be employed to determine PD-L1 expression levels by utilizing another PD-L1 IHC assay in the same tissue specimens. This approach may provide a better understanding of the performance of PD-L1 expression level as a predictive biomarker of response to REGN2810. Of special interest is PD-L1 expression across different cell types including tumor cells, stroma cells, and infiltrating immune cells.

After completion of PD-L1 expression analysis, the remaining tumor tissue samples may be used to study the biomarkers associated with clinical response to REGN2810 including but not limited to whole exome sequencing of tumor genome and tumor mutational load.

Biomarker serum and plasma samples will be collected from all patients enrolled in this study at multiple visits and time points indicated in Table 5, Table 6, and Appendix 4 to study the potential pharmacodynamics or predictive biomarkers of response to REGN2810 including, but not limited to, cytokines and circulating tumor nucleic acids (refer to the laboratory manual).

A tumor tissue biopsy sample should be obtained at the time of RECIST 1.1-defined progressive disease.

8.2.6. Future Biomedical Research

The biomarker samples unused for study-related research, as well as unused PK and ADA samples, will be stored for up to 15 years after the final date of the database lock. The unused samples may be utilized for future biomedical research of lung carcinoma and other diseases. No additional samples will be collected for future biomedical research. After 15 years, any residual samples will be destroyed.

8.2.6.1. Genomics Sub-Study - Optional

Patients who agree to participate in the genomics sub-study will be required to sign a separate genomics sub-study ICF before collection of the samples. Patients are not required to participate in the genomics sub-study in order to enroll in the primary study. Samples for DNA extraction should be collected on day 1/baseline (predose), but may be collected at any study visit.

DNA samples for the genomics sub-study will be double-coded as defined by the International Council for Harmonisation (ICH) guideline E15. Sub-study samples will be stored for up to 15 years after the final date of the database lock and may be used for research purposes. The purpose of the genomic analyses is to identify genomic associations with clinical or biomarker response, other clinical outcome measures and possible AEs. In addition, associations between genomic variants and prognosis or progression of other diseases may also be studied. These data may be used or combined with data collected from other studies to identify and validate genomic markers related to the study drug or other diseases. Analyses may include sequence determination or single nucleotide polymorphism studies of candidate genes and surrounding genomic regions. Other methods, including whole-exome sequencing, whole-genome sequencing, and DNA copy number variation may also be performed. The list of methods may be expanded to include novel methodology that may be developed during the course of this study or sample storage period.

CONFIDENTIAL

VV-RIM-00023209-1.0 Approved - 29 Aug 2017 GMT-5:00

9. SAFETY DEFINITIONS, REPORTING, AND MONITORING

For the purposes of this section, study treatment refers to REGN2810, platinum-based doublet chemotherapy agents and ipilimumab.

9.1. Obligations of Investigator

The investigator must promptly report to the Institutional Review Board (IRB)/Ethics Committee (EC) all unanticipated problems involving risks to patients according to local regulations.

9.2. Obligations of Sponsor

During the course of the study, the sponsor will report in an expedited manner all SAEs that are both unexpected and at least reasonably related to the study drug (suspected, unexpected, serious adverse reactions), to the health authorities, IRB/ECs as appropriate, and to the investigators.

Any AE not listed as an expected event in the Reference Safety Information section of the REGN2810 Investigator's Brochure or in the reference safety documents of the other study drugs will be considered as unexpected.

In addition, the sponsor will report all other SAEs to the health authorities, according to local regulations, if this is applicable to the country requirements.

At the completion of the study, the sponsor will report all safety observations made during the conduct of the study in the clinical study report to health authorities and IRB/ECs, as appropriate.

9.3. Definitions

9.3.1. Adverse Event

An AE is any untoward medical occurrence in a patient administered a study drug which may or may not have a causal relationship with the study drug. Therefore, an AE is any unfavorable and unintended sign (including abnormal laboratory finding), symptom, or disease which is temporally associated with the use of a study drug, whether or not considered related to the study drug.

An AE also includes any worsening (ie, any clinically significant change in frequency and/or intensity) of a pre-existing condition that is temporally associated with the use of the study drug.

Progression of underlying malignancy will not be considered an AE if it is clearly consistent with the typical progression pattern of the underlying cancer (including time course, affected organs, etc.). Clinical symptoms of progression may be reported as AEs if the symptom cannot be determined as exclusively due to the progression of the underlying malignancy, or does not fit the expected pattern of progression for the disease under study.

If there is any uncertainty about an AE being due only to progression of the underlying malignancy, it should be reported as an AE or SAE as outlined in Section 9.3.2.

VV-RIM-00023209-1.0 Approved - 29 Aug 2017 GMT-5:00

9.3.2. Serious Adverse Event

An SAE is any untoward medical occurrence that at any dose:

- Results in **death** – includes all deaths, even those that appear to be completely unrelated to study drug (eg, a car accident in which a patient is a passenger).
- Is **life-threatening** – in the view of the investigator, the patient is at immediate risk of death at the time of the event. This does not include an AE that had it occurred in a more severe form, might have caused death.
- Requires in-patient **hospitalization** or **prolongation of existing hospitalization**. In-patient hospitalization is defined as admission to a hospital or an emergency room for longer than 24 hours. Prolongation of existing hospitalization is defined as a hospital stay that is longer than was originally anticipated for the event, or is prolonged due to the development of a new AE as determined by the investigator or treating physician.
- Results in persistent or significant **disability/incapacity** (substantial disruption of one's ability to conduct normal life functions).
- Is a **congenital anomaly/birth defect**.
- Is an **important medical event -** Important medical events may not be immediately life-threatening or result in death or hospitalization, but may jeopardize the patient or may require intervention to prevent one of the other serious outcomes listed above (eg, intensive treatment in an emergency room or at home for allergic bronchospasm; blood dyscrasias or convulsions that do not result in hospitalization; or development of drug dependency or drug abuse).

Hospitalization or death due solely to manifestations consistent with typical progression of underlying malignancy will not be considered as an SAE.

Serious adverse events must be reported as directed in Section 9.4.2.

9.3.3. Adverse Events of Special Interest

An AESI (serious or non-serious) is one of scientific and medical concern specific to the sponsor's product or program, for which ongoing monitoring and rapid communication by the investigator to the sponsor can be appropriate. Such an event might warrant further investigation in order to characterize and understand it. Depending on the nature of the event, rapid communication by the study sponsor to other parties (eg, regulators) might also be warranted. All AESI, serious and non-serious, must be reported within 24 hours of identification using the same reporting process as for SAE reporting, per Section 9.4.2. Adverse events of special interest for this study include the following:

- Any AE that meets the DLT criteria (defined in Section 5.1.5)
- Grade 2 or greater infusion-related reactions
- Grade 2 or greater allergic/hypersensitivity reactions
- Grade 3 or greater irAEs

VV-RIM-00023209-1.0 Approved - 29 Aug 2017 GMT-5:00

Note: An irAE can occur shortly after the first dose or several months after the last dose of study treatment. All AEs of unknown etiology associated with REGN2810 exposure should be evaluated to determine possible immune etiology. If an irAE is suspected, efforts should be made to rule out neoplastic, infectious, metabolic, toxin, or other etiologic causes prior to labeling an AE as an irAE. Detailed guidance of management of irAEs is provided in Section 7.3.3 and Appendix 3. The recommendations in Section 7.3.3 and Appendix 3 should be seen as guidelines, and the treating physician should exercise clinical judgment based on the symptoms and condition of the individual patient. For any AE that is of a type known to be potentially immune-related (eg, rash, colitis, elevated transaminases, endocrine, or pneumonitis) but is deemed not to be an irAE by the investigator, the sponsor may request additional information.

9.3.3.1. Immune-Related Adverse Events

Detailed guidance of management of irAEs is provided in Section 7.3.3 and Appendix 3.

Note regarding irAEs: For any AE that is of a type known to be potentially immune-related (eg, rash, colitis, elevated transaminases, endocrine, or pneumonitis) but is deemed not to be an irAE by the investigator, the sponsor may request additional information.

9.3.4. Infusion Reactions

Infusion-related reactions are known to occur with protein therapeutic infusions and have been observed in REGN2810 studies. Acute infusion reactions are defined as any AEs that occur during the infusion or within 2 hours after the infusion is completed. Signs and symptoms usually develop during or shortly after drug infusion and generally resolve completely within 24 hours of completion of infusion.

All infusion reactions must be reported as AEs (defined in Section 9.4.1) and graded using the grading scales as instructed in Section 9.5.1.

9.4. Recording and Reporting Adverse Events

9.4.1. Adverse Events

The investigator (or designee) will seek information on AEs at each patient contact, and record all AEs that occur from the time the informed consent is signed until 105 days after the end of study treatment. After informed consent has been obtained but prior to initiation of study treatment, only the following categories of AEs should be reported on the AE eCRF:

- SAEs
- Non-SAEs associated with a protocol-mandated intervention (eg, AEs related to an invasive procedure such as a biopsy)

Other AEs that occur prior to first treatment should be reported on the medical history eCRF.

All AEs after initiation of study treatment and until 90 days after the last study treatment, regardless of relationship to study treatment, will be reported on the AE eCRF. Additionally, any SAE or other AE of concern that the investigator believes may be related to study treatment and that occurs later than 90 days after last study treatment should be reported.

Information on follow-up for AEs is provided in Section 9.4.6. Laboratory, vital signs, or ECG abnormalities are to be recorded as AEs as outlined in Section 9.4.5.

9.4.2. Serious Adverse Events

All SAEs, regardless of assessment of causal relationship to study drug, must be reported to the sponsor (or designee) within 24 hours of becoming aware of the event.

Information not available at the time of the initial report must be documented in a follow-up report. Substantiating data such as relevant hospital or medical records and diagnostic test reports may also be requested.

In the event that the investigator is informed of an SAE that occurs more than 90 days after the last dose of study treatment, only those SAEs or other AEs of concern deemed by the investigator to be related to study treatment will be reported to the sponsor. The investigator should make every effort to obtain follow-up information on the outcome of any treatment-related SAE and/or until the event is considered chronic and/or stable.

9.4.3. Other Events that Require Accelerated Reporting to Sponsor

The following events also require reporting to the sponsor (or designee) within 24 hours of learning of the event:

Symptomatic Overdose of Study Drug: Accidental or intentional overdose of at least 2 times the intended dose of study drug within the intended therapeutic window, if associated with an AE.

Pregnancy: Although pregnancy is not considered an AE, it is the responsibility of the investigator to report to the sponsor (or designee), within 24 hours of identification, any pregnancy occurring in a female or female partner of a male, during the study or within 180 days of the last dose of study drug. Any complication of pregnancy affecting a female study patient or female partner of a male study patient, and/or fetus and/or newborn that meets the SAE criteria must be reported as an SAE. Outcome for all pregnancies should be reported to the sponsor.

9.4.4. Reporting Adverse Events Leading to Withdrawal from the Study

All AEs that lead to a patient’s withdrawal from the study must be reported to the sponsor’s Medical Monitor within 30 days.

9.4.5. Abnormal Laboratory, Vital Signs, or Electrocardiogram Results

The criteria for determining whether an abnormal objective test finding should be reported as an AE include:

- the test result is associated with accompanying symptoms, and/or
- the test result requires additional diagnostic testing or medical/surgical intervention, and/or

- the test result leads to a change in dosing (outside of protocol-stipulated dose adjustments), discontinuation from the study, significant additional concomitant drug treatment, or other therapy

Contact the Medical Monitor in the event the investigator feels that an abnormal test finding should be reported as an AE, although it does not meet any of the above criteria.

Repeating an abnormal test, in the absence of any of the above conditions, does not constitute an AE. Any abnormal test result that is determined to be an error does not require reporting as an AE.

Evaluation of severity of laboratory abnormalities will be assessed according to the scale outlined in Section 9.5.1.

9.4.6. Follow-up

Information for any non-SAE that starts during the treatment period or within 90 days after last dose of study treatment will be collected from the time of the event until resolution of the event, or until the patient's last study visit, whichever comes first.

Serious adverse event information will be collected until the event is considered chronic and/or stable.

9.5. Evaluation of Severity and Causality

9.5.1. Evaluation of Severity

The severity of AEs (including test findings classified as AEs) will be graded using the current version of the NCI-CTCAE grading system. Adverse events not listed in the NCI-CTCAE will be graded according to the following scale:

1 (Mild): Mild; asymptomatic or mild symptoms; clinical or diagnostic observations only; intervention not indicated

2 (Moderate): Moderate; minimal, local or noninvasive intervention indicated; limiting age-appropriate instrumental Activities of Daily Living (ADL)*

3 (Severe): Severe or medically significant but not immediately life-threatening; hospitalization or prolongation of hospitalization indicated; disabling; limiting self-care ADL**

4 (Life–threatening): Life-threatening consequences; urgent intervention indicated

5 (Death): Death related to AE

*Instrumental ADL refer to preparing meals, shopping for groceries or clothes, using the telephone, managing money, etc.

**Self-care ADL refer to bathing, dressing and undressing, feeding self, using the toilet, taking medications, and not bedridden.

If a laboratory value is considered as an AE, its severity should be based on the degree of physiological impairment the value indicates.

VV-RIM-00023209-1.0 Approved - 29 Aug 2017 GMT-5:00

9.5.2. Evaluation of Causality

Relationship of Adverse Events to Study Drug:

The relationship of AEs to study drug will be assessed by the investigator and will be a clinical decision based on all available information. The following question will be addressed:

Is there a reasonable possibility that the AE may have been caused by the study drug?

The possible answers are:

Not Related: There is no reasonable possibility that the event may have been caused by the study drug

Related: There is a reasonable possibility that the event may have been caused by the study drug

A list of factors to consider when assessing the relationship of AEs to study drug is provided in Appendix 1.

- The investigator should justify the causality assessment of each SAE.

Relationship of Adverse Events to Study Conduct:

The relationship of AEs to study conduct will be assessed by the investigator and will be a clinical decision based on all available information. The following question will be addressed:

Is there a reasonable possibility that the AE may have been caused by study conduct?

The possible answers are:

Not Related: There is no reasonable possibility that the event may have been caused by study conduct

Related: There is a reasonable possibility that the event may have been caused by study conduct

A list of factors to consider when assessing the relationship of AEs to study conduct is provided in Appendix 1.

The investigator should justify the causality assessment of each SAE.

9.6. Safety Monitoring

The investigator will monitor the safety of the study patient at his/her site(s) as per the requirements of this protocol and consistent with current Good Clinical Practice (GCP). Any questions or concerns should be discussed with the sponsor in a timely fashion. The sponsor will monitor the safety data from across all study sites. The Medical Monitor will have primary responsibility for the emerging safety profile of the compound but will be supported by other departments (eg, Pharmacovigilance and Risk Management and Biostatistics and Data Management). Safety monitoring will be performed on an ongoing basis (eg, individual review of SAEs) and on a periodic cumulative aggregate basis.

9.7. Investigator Alert Notification

Regeneron (or designee) will inform all investigators participating in this clinical study, as well as in any other clinical study using the same investigational drug, of any SAE that meets the

VV-RIM-00023209-1.0 Approved - 29 Aug 2017 GMT-5:00

relevant requirements for expedited reporting (an AE that is serious, unexpected based on the Investigator's Brochure or this protocol, and has a reasonable suspected causal relationship to the medicinal/study drug).

10. STATISTICAL PLAN

This section provides the basis for the statistical analysis plan (SAP) for the study. The SAP may be revised during the study to accommodate amendments to the clinical study protocol and to make changes to adapt to unexpected issues in study execution and data that may affect the planned analyses. The final SAP will be issued before the database is locked.

Analysis variables are listed in Section 4.

Although the study is open-label, any analyses or summaries generated by randomized treatment assignment or actual treatment received, and access to those analyses or summaries, will be limited and documented.

10.1. Statistical Hypothesis

The primary statistical hypotheses are that REGN2810/chemo-f or REGN2810/chemo-l/ipi will prolong PFS as compared with platinum-based standard-of-care doublet chemotherapy in the subgroup of patients whose tumors express PD-L1 in 1% to <50% of tumor cells and in the overall population of study patients whose tumors express PD-L1 in <50% of tumor cells. The secondary hypotheses are that REGN2810/chemo-f or REGN2810/chemo-l/ipi will improve OS in the subgroup of patients whose tumors express PD-L1 in 1% to <50% of tumor cells and in the overall population of study patients whose tumors express PD-L1 in <50% of tumor cells.

10.2. Justification of Sample Size

Historically, in patients with stage IIIB or stage IV NSCLC treated with cisplatin or carboplatin plus paclitaxel Q3W, the median PFS has ranged from approximately 2.7 to 6.4 months (El-Shenshawy 2012, Kelly 2001, Rosell 2002, Scagliotti 2002, Schiller 2002, Shimizu 2013, Socinski 2016).

The study assumes a median PFS of 6 months for patients treated with chemotherapy alone and a median PFS of 10 months for patients treated with each of the REGN2810 combination therapies. The assumptions correspond to a 66.7% increase in median PFS and an HR of 0.6. Under these assumptions, and for each REGN2810 combination treatment arm versus the standard-of-care chemotherapy comparison, 190 PFS events are needed to yield approximately 90% power to detect statistical significance at 2-sided 0.025 level.

Considering a uniform enrollment rate and a combined enrollment and PFS follow-up duration of approximately 18 months (12 months for enrollment and approximately 6 months follow-up for PFS) and 10% dropout rate per year, enrollment of approximately 477 randomized patients (159 patients per arm) in the subgroup of patients whose tumors express PD-L1 in 1% to <50% of tumor cells is needed to generate enough PFS events to yield approximately 90% power for each REGN2810 combination treatment arm versus the standard-of-care chemotherapy comparison treatment arm.

VV-RIM-00023209-1.0 Approved - 29 Aug 2017 GMT-5:00

It is projected that approximately 70% of the patients whose tumors express PD-L1 in <50% of tumor cells will have tumors that express PD-L1 in 1% to <50% of tumor cells. Therefore, approximately 690 patients will be randomized into this study in order to separately test and have power for the subgroup of patients whose tumors express PD-L1 in 1% to <50% of tumor cells.

In the subgroup of patients whose tumors express PD-L1 in 1% to <50% of tumor cells (as determined by the PD-L1 IHC pharmDx assay), it is projected that an observed HR of 0.72 or lower, which corresponds to an increase in median PFS of 2.3 months or more (6 months versus 8.3 months), would result in a statistically significant improvement in PFS.

Assuming OS will be analyzed 12 months after the analysis of PFS, a median OS of 13 months for patients treated with platinum-based standard-of-care chemotherapy alone, a median OS of 18.571 months for patients treated with each of the REGN2810 combination therapies, and a corresponding HR of 0.7 under exponential distribution, the analysis of OS in the subgroup of patients whose tumors express PD-L1 in 1% to <50% of tumor cells will have approximately 58% power at significance level of 2-sided 0.025 for each REGN2810 combination treatment arm versus the standard-of-care chemotherapy arm.

10.3. Analysis Sets

10.3.1. Efficacy Analysis Sets

The full analysis set (FAS) includes all randomized patients and will be the intention-to-treat population. The FAS is based on the treatment allocation (as randomized). All efficacy endpoints will be analyzed using the FAS.

10.3.2. Safety Analysis Set

The safety analysis set (SAF) includes all randomized patients who received any study drug; it is based on the treatment received (as treated). Treatment compliance/administration and all clinical safety variables will be analyzed using the SAF.

10.3.3. Other Analysis Sets

The PK population includes all randomized patients (safety population) who receive REGN2810 and who have at least 1 non-missing REGN2810 concentration assay result following the first dose of REGN2810 up to the end of the study.

The ADA analysis set includes all treated patients who received any study drug and had at least 1 non-missing post-baseline ADA assay result following the first dose of study drug.

The DLT analysis set includes all patients treated with REGN2810/chemo-l/ipi who are DLT evaluable, defined as the patients who completed the DLT observation period and those patients who discontinued early due to the development of a DLT. This population will be used for the assessment of DLTs. The patients will be analyzed as treated.

10.4. Statistical Methods

For continuous variables, descriptive statistics will include the following information: the number of patients reflected in the calculation (n), mean, median, standard deviation, minimum, and maximum.

For categorical or ordinal data, frequencies and percentages will be displayed for each category.

The descriptive summary of time-to-event data will include median time to event and the corresponding 95% CI using the Kaplan-Meier method.

10.4.1. Patient Disposition

The following will be provided:

- The total number of screened patients: met the inclusion criteria regarding the target indication and signed the ICF
- The total number of patients in the FAS
- The total number of patients in the SAF
- The total number of patients who discontinued treatment and the reasons for treatment discontinuation
- The total number of patients who discontinued the study and the reasons for discontinuation
- A listing of patients treated but not randomized, patients randomized but not treated, and patients randomized but not treated as randomized
- A listing of patients prematurely discontinued from treatment and study, along with reasons for discontinuation

10.4.2. Demography and Baseline Characteristics

Demographic and baseline characteristics will be summarized descriptively by treatment arm, and by all patients combined.

10.4.3. Medical History

Medical history will be summarized by primary system organ class (SOC) and preferred term (PT) for each treatment arm, with the table sorted by decreasing frequency of SOC, followed by PT, based on the overall incidence between treatment arms.

10.4.4. Prior Medications/Concomitant Medications

Number and proportion of patients taking prior/concomitant medication will be summarized by decreasing frequency of anatomical therapeutic chemical (ATC) level 2 and ATC level 4 according to the current version of the World Health Organization Drug Dictionary, based on the overall incidence between treatment arms.

Listings of pre-treatment medication and concomitant medications will include generic name, ATC levels 2 and 4, indication, the study day onset, the study end date (defined similarly as for study onset day), ongoing status, dose, frequency, and route.

For medications that are started before treatment, the study day onset is defined as the date of medication start to date of the first dose of treatment; for medications that are started on or after treatment, the study day onset is defined as the date of medication start to date of the first dose + 1.

10.4.5. Efficacy Analyses

The primary and key secondary endpoints will be tested in the following order: PFS, OS, and ORR.

10.4.5.1. Primary Efficacy Analyses

The primary endpoint of PFS will be analyzed first in the subgroup of patients whose tumors express PD-L1 in 1% to <50% of tumor cells and will be performed when approximately 110 PFS events are observed in the subgroup and in the standard-of-care chemotherapy treatment arm. If the primary analysis of PFS for a REGN2810 combination versus standard-of-care chemotherapy is statistically significant at a 2-sided alpha=0.025 level in the subgroup of patients whose tumors express PD-L1 in 1% to <50% of tumor cells, the primary analysis of PFS for that REGN2810 combination versus standard-of-care chemotherapy will then be performed in the overall study population (patients whose tumors express PD-L1 in <50% of tumor cells).

In the subgroup of patients whose tumors express PD-L1 in 1% to <50% of tumor cells and in the overall study population, the primary endpoint of PFS will be analyzed by stratified log-rank test using the status of histology (non-squamous versus squamous) and levels of PD-L1 expression (<1% versus 1% to <25% versus 25% to <50%) as stratification factors. The HR and the corresponding 95% CI will be estimated by a stratified Cox regression model using the treatment as covariate.

Three sensitivity analyses will be performed using different censoring rules and progressive disease event definitions. The first sensitivity analysis is the same as the primary analysis except that it considers initiation of new anti-cancer treatment as a progressive-disease-event for patients without documented progressive disease or death prior to initiation of new anti-tumor treatment. The second sensitivity analysis is the same as the primary analysis except that for the second sensitivity analysis, a patient who has progressive disease or death after missing ≥2 tumor assessments will be censored at the last tumor assessment prior to missing ≥2 tumor assessments. The third sensitivity analysis will be performed based on investigator-determined progressive disease events.

10.4.5.2. Secondary Efficacy Analyses

For a REGN2810 combination versus standard chemotherapy comparison, if the final analysis of PFS is statistically significant in the subgroup of patients whose tumors express PD-L1 in 1% to <50% of tumor cells and in the overall population of study patients whose tumors express PD-L1 in <50% of tumor cells, the analysis of the secondary endpoint of OS will be performed at the time of PFS analysis and approximately 12 months later or when 104 OS deaths are observed in patients receiving standard chemotherapy treatment using the same method as used in the analysis of PFS. The familywise type I error will be controlled at the 2-sided 0.025 level and detailed in the SAP. Descriptive analyses will also be provided for the entire population, including patients who receive second-line treatment with REGN2810 or other PD-1 therapy.

The ORR will be analyzed using the Cochran-Mantel-Haenszel test stratified by status of histology (non-squamous versus squamous). An associated odds ratio and 95% CI will be calculated. Objective response rate and the corresponding exact 95% CI will be calculated by Clopper-Pearson method for each treatment arm.

VV-RIM-00023209-1.0 Approved - 29 Aug 2017 GMT-5:00

Overall survival at 12 months, 18 months, and end of treatment for each treatment arm will be summarized.

The change in EORTC QLQ-C30 and EORTC QLQ-LC13 scores from first assessment to the end of the study will be summarized descriptively at each post-baseline time point and compared using a mixed effects model, if appropriate.

10.4.5.3. Subgroup Analyses

To determine the consistency of treatment effect across various demographic and baseline subgroups, the estimate of the between-group treatment effect (with a nominal 95% CI) for the primary and secondary endpoints will be estimated and plotted within each category of the following subgroup variables:

- Age category (≤65 versus >65 years)
- Gender (female, male)
- Race (white, non-white)
- Histology (squamous, non-squamous)
- PD-L1 expression levels (<1% versus 1% to <25% versus 25% to <50%)
- ECOG status (0 versus 1)
- Geographic region of enrolling site
- Ethnicity

10.4.6. Safety Analyses

Safety observations and measurements, including drug exposure, AEs, laboratory data, vital signs, and ECOG performance status, will be summarized and presented in tables and listings.

Dose-limiting toxicities observed during the DLT evaluation period will be summarized by treatment arm and will be assessed using the DLT analysis set.

10.4.6.1. Adverse Events

Definitions

For safety variables, 3 observation periods are defined:

- The pre-treatment period is defined as the time from signing the ICF to before the first dose of study drug.
- The on-treatment period is defined as the day from the first dose of study drug to the day of the last dose of study drug plus 90 days.
- The post-treatment period is defined as the time after follow-up visit 1.

Treatment-emergent adverse events are defined as those that are not present at baseline or represent the exacerbation of a pre-existing condition during the on-treatment period.

VV-RIM-00023209-1.0 Approved - 29 Aug 2017 GMT-5:00

Analysis

All AEs reported in this study will be coded using the currently available version of the Medical Dictionary for Regulatory Activities (MedDRA). Coding will be to lowest-level terms. The verbatim text, the PT, and the primary SOC will be listed.

Summaries of all TEAEs by treatment arm will include the following:

- The number (n) and percentage (%) of patients with at least 1 TEAE by SOC and PT
- TEAEs by severity (graded according to the current version of the NCI-CTCAE), presented by SOC and PT
- TEAEs by outcome
- TEAEs by relationship to study drug (related, not related), presented by SOC and PT
- AESI

Deaths and other SAEs will be listed and summarized by treatment arm.

Events of NCI-CTCAE grade 3 and grade 4 severity will be summarized by treatment arm.

Treatment-emergent adverse events leading to permanent treatment discontinuation will be listed and summarized by treatment arm.

10.4.6.2. Other Safety Analyses

Vital Signs

Vital signs (temperature, heart rate, seated blood pressure, and respiration rate) will be summarized by baseline and change from baseline to each scheduled assessment time with descriptive statistics.

Laboratory Tests

Laboratory test results will be listed, and the number and percentage of patients with NCI-CTCAE grade 3 or grade 4 laboratory values will be summarized by laboratory test. Shift tables may be generated if applicable.

10.4.6.3. Treatment Exposure

Treatment duration, dose intensity, and number of cycles administered will be summarized by treatment arm.

10.4.6.4. Treatment Compliance

Treatment compliance, including dose, number of doses, timing and applicable study concomitant procedures, will be summarized by treatment arm. The analysis methods will be detailed in the SAP.

CONFIDENTIAL

VV-RIM-00023209-1.0 Approved - 29 Aug 2017 GMT-5:00

10.4.7. Analysis of Drug Concentration Data

REGN2810 concentrations in serum will be reported over time as individual values with descriptive statistics. The PK data in this study may be included in a population PK analysis that will be presented in a separate report.

Blood samples for analysis of ipilimumab concentrations will be stored for possible future analysis, the results of which may be reported in the same way as for REGN2810 concentrations.

10.4.8. Analysis of Anti-Drug Antibody Data

The ADA variables described in Section 4.4 will be summarized using descriptive statistics in the ADA analysis set of the REGN2810 treatment arms (Treatment Arms B and C). Frequency tables of the proportion of patients with treatment-emergent, treatment-boosted, persistent ADA response, and NAb status in the NAb assay will be presented as absolute occurrence (n) and percentage of patients (%), presented by treatment arms.

Plots of REGN2810 concentrations will be examined, and the influence of ADAs on individual concentration-time profiles may be evaluated. Assessment of impact of ADA on safety and efficacy may be provided.

10.4.9. Analysis of Biomarker Data

Biomarker analyses in this study will be exploratory in nature, and results will be summarized in a separate report. Detailed description of statistical methods that will be used for biomarker data analyses will be provided in a separate Biomarker Analytical Plan.

10.5. Multiplicity Considerations

For the 2 REGN2810 combinations versus standard of care chemotherapy comparisons, familywise type-I error rate of 0.05 is controlled by Bonferroni approach (split alpha into 0.025 for each comparison).

Within each REGN2810 combination versus chemotherapy comparison, the type I error for analysis in the subgroup of patients whose tumors express PD-L1 in 1% to <50% of tumor cells and in the overall population of study patients whose tumors express PD-L1 in <50% of tumor cells and between the primary endpoint of PFS and the secondary endpoints of OS and ORR is controlled by hierarchical testing strategy. The order of the hierarchical testing strategy for primary and key secondary endpoints is outlined in Figure 2.

VV-RIM-00023209-1.0 Approved - 29 Aug 2017 GMT-5:00

Figure 2: Hierarchical Testing Strategy for Primary and Key Secondary Endpoints

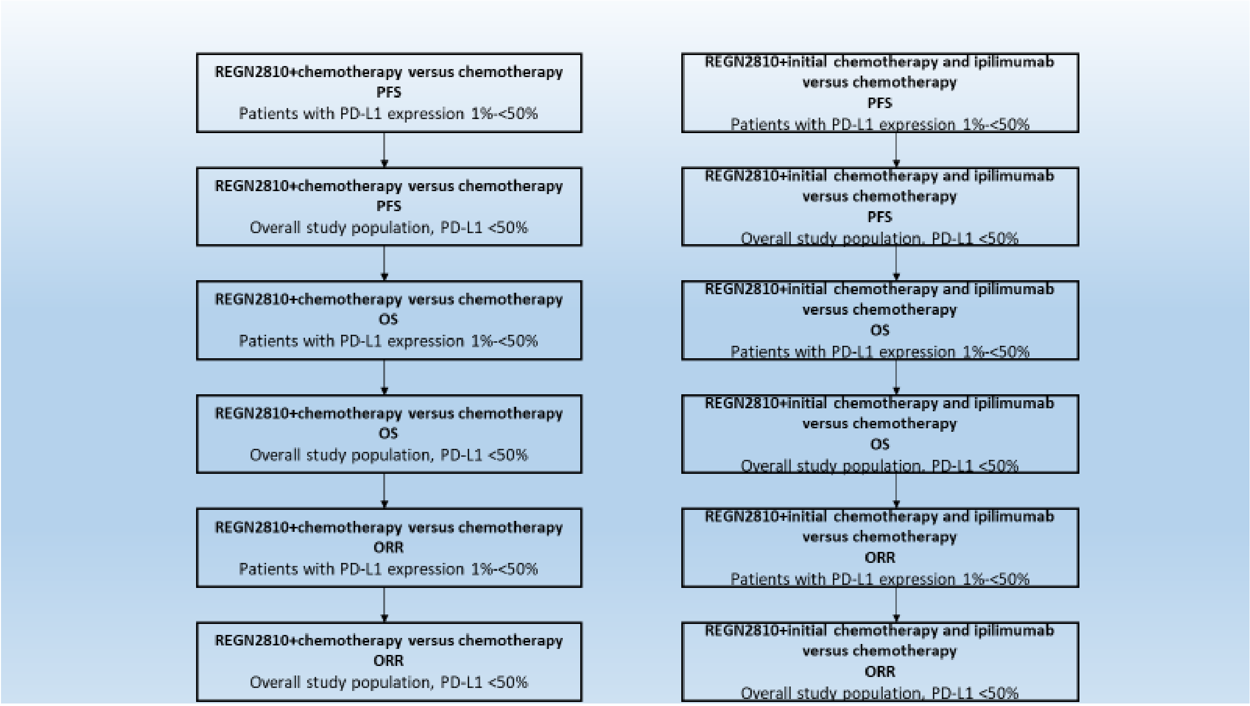

Abbreviations: ORR=objective response rate; OS=overall survival; PD-L1=programmed cell death ligand 1; PFS=progression-free survival

The alpha spending for analysis of OS at the time of PFS analysis and the final analysis will be controlled at a 2-sided 0.025 level and detailed in the SAP. All other statistical comparisons will be exploratory in nature and, therefore, not controlled for multiplicity and should be interpreted accordingly.

10.6. Interim Analysis

No interim analysis is planned for this study. There will be an early safety review conducted by the IDMC as detailed in Section 5.1.4.

10.7. Additional Statistical Data Handling Conventions

The following analysis and data conventions will be followed:

Definition of baseline:

- Unless otherwise specified, the last assessment before the initial administration of REGN2810 will be considered the baseline evaluation.

General rules for handling missing data:

- Unless otherwise specified, there will be no imputations for missing data.
- The pattern of missing data and potential prognostic factors for missing data (QOL, clinical neurologic assessment and mental status) will be examined to guide the use of proper statistical models.

VV-RIM-00023209-1.0 Approved - 29 Aug 2017 GMT-5:00

- If the start date of an AE or concomitant medication is incomplete or missing, it will be assumed to have occurred on or after the intake of study drug except if an incomplete date (eg, month and year) clearly indicates that the event started prior to treatment. If the partial date indicates the same month or year of the intake of study drug date, then the start date by the study drug intake date will be imputed; otherwise, the missing day or month by the first day or the first month will be imputed.

Visit windows:

- Assessments taken outside of protocol allowable windows will be displayed according to the eCRF assessment recorded by the investigator.

Unscheduled assessments:

- Extra assessments (laboratory data or vital signs associated with non-protocol-defined clinical visits or obtained in the course of investigating or managing AEs) will be included in the listings, but not in the summaries. If more than 1 laboratory value is available for a given visit, the first observation will be used in summaries, and all observations will be presented in listings.

10.8. Statistical Considerations Surrounding the Premature Termination of a Study

The study is expected to end after the last visit of the last patient.

If the study is terminated prematurely, only those parameters required for the development program and/or reporting to regulatory authorities will be summarized. Investigator and sponsor responsibilities surrounding the premature termination of a study are presented in Section 16.1.

11. DATA MANAGEMENT AND ELECTRONIC SYSTEMS

11.1. Data Management

A data management plan specifying all relevant aspects of data processing for the study (including data validation, cleaning, correcting, and releasing) will be maintained and stored at Regeneron.

A medical coding plan will specify the processes and the dictionary used for coding. All data coding (eg, AEs, baseline findings, medication, medical history/surgical history) will be done using internationally recognized and accepted dictionaries.

The eCRF data for this study will be collected using an electronic data capture (EDC) tool. User training must be documented before the user is granted access to the EDC system.

11.2. Electronic Systems

Electronic systems that may be used to process and/or collect data in this study will include the following:

- IVRS/IWRS system – randomization, study drug supply

- EDC system – data capture
- Statistical Analysis System (SAS) – statistical review and analysis
- Pharmacovigilance safety database
- ARGUS safety database

12. STUDY MONITORING

12.1. Monitoring of Study Sites

The study monitor and/or designee (eg, contract research organization [CRO] monitor) will visit each site prior to enrollment of the first patient, and periodically during the study.

12.2. Source Document Requirements

Investigators are required to prepare and maintain adequate and accurate patient records (source documents).

The investigator must keep all source documents on file with the case report form (CRF) (throughout this protocol, CRF refers to either a paper CRF or an electronic CRF [eCRF]). Case report forms and source documents must be available at all times for inspection by authorized representatives of the sponsor and regulatory authorities.

12.3. Case Report Form Requirements

Study data obtained in the course of the clinical study will be recorded on eCRFs within the EDC system by trained site personnel. All required eCRFs must be completed for each and every patient enrolled in the study. After review of the clinical data for each patient, the investigator must provide an electronic signature. A copy of each patient eCRF casebook is to be retained by the investigator as part of the study record and must be available at all times for inspection by authorized representatives of the sponsor and regulatory authorities.

Corrections to the eCRF will be entered in the eCRF by the investigator or an authorized designee. All changes, including details regarding the date and person performing corrections, will be available via the audit trail, which is part of the EDC system. For corrections made via data queries, a reason for any alteration must be provided.

13. AUDITS AND INSPECTIONS

This study may be subject to a quality assurance audit or inspection by the sponsor or regulatory authorities. Should this occur, the investigator is responsible for the following:

- Informing the sponsor of a planned inspection by the authorities as soon as notification is received, and authorizing the sponsor's participation in the inspection
- Providing access to all necessary facilities, study data, and documents for the inspection or audit

- Communicating any information arising from inspection by the regulatory authorities to the sponsor immediately
- Taking all appropriate measures requested by the sponsor to resolve the problems found during the audit or inspection

Documents subject to audit or inspection include, but are not limited to, all source documents, eCRFs, medical records, correspondence, ICFs, IRB/EC files, documentation of certification and quality control of supporting laboratories, and records relevant to the study maintained in any supporting pharmacy facilities. Conditions of study material storage are also subject to inspection. In addition, representatives of the sponsor may observe the conduct of any aspect of the clinical study or its supporting activities both within and outside of the investigator's institution.

In all instances, the confidentiality of the data must be respected.

14. ETHICAL AND REGULATORY CONSIDERATIONS

14.1. Good Clinical Practice Statement

It is the responsibility of both the sponsor and the investigator(s) to ensure that this clinical study will be conducted in accordance with the ethical principles that have their origin in the Declaration of Helsinki, and that are consistent with the ICH guidelines for GCP and applicable regulatory requirements.

14.2. Informed Consent

The principles of informed consent are described in ICH guidelines for GCP.

The ICF used by the investigator must be reviewed and approved by the sponsor prior to submission to the appropriate IRB/EC. A copy of the IRB/EC-approved ICF and documentation of approval must be provided to the sponsor before study drug will be shipped to the study site.

It is the responsibility of the investigator or designee (if acceptable by local regulations) to obtain written informed consent from each patient prior to his/her participation in the study and after the aims, methods, objectives, and potential hazards of the study have been explained to the patient in language that he/she can understand. The ICF should be signed and dated by the patient and by the investigator or authorized designee who reviewed the ICF with the patient.

- Patients who can write but cannot read will have the ICF read to them before signing and dating the ICF.
- Patients who can understand but who can neither write nor read will have the ICF read to them in presence of an impartial witness, who will sign and date the ICF to confirm that informed consent was given.

The original ICF must be retained by the investigator as part of the patient’s study record, and a copy of the signed ICF must be given to the patient.

If new safety information results in significant changes in the risk-benefit assessment, the ICF must be reviewed and updated appropriately. All study patients must be informed of the new

information and provide their written consent if they wish to continue in the study. The original signed revised ICF must be maintained in the patient's study record and a copy must be given to the patient.

14.3. Patients' Confidentiality and Data Protection

The investigator must take all appropriate measures to ensure that the anonymity of each study patient will be maintained. Patients should be identified by a patient identification number, only, on eCRFs or other documents submitted to the sponsor. Documents that will not be submitted to the sponsor (eg, signed ICF) must be kept in strict confidence.

The patient's and investigator's personal data, which may be included in the sponsor database, will be treated in compliance with all applicable laws and regulations. The sponsor shall take all appropriate measures to safeguard and prevent access to this data by any unauthorized third party.

14.4. Institutional Review Board/Ethics Committee

An appropriately constituted IRB/EC, as described in ICH guidelines for GCP, must review and approve:

- The protocol, ICF, and any other materials to be provided to the patients (eg, advertising) before any patient may be enrolled in the study
- Any amendment or modification to the study protocol or ICF before implementation, unless the change is necessary to eliminate an immediate hazard to the patient, in which case the IRB/EC should be informed as soon as possible
- Ongoing studies on an annual basis or at intervals appropriate to the degree of risk

In addition, the IRB/EC should be informed of any event likely to affect the safety of patients or the continued conduct of the clinical study.

A copy of the IRB/EC approval letter with a current list of the IRB/EC members and their functions must be received by the sponsor prior to shipment of drug supplies to the investigator. The approval letter should include the study number and title, the documents reviewed, and the date of the review.

Records of the IRB/EC review and approval of all study documents (including approval of ongoing studies) must be kept on file by the investigator.

15. PROTOCOL AMENDMENTS

The sponsor may not implement a change in the design of the protocol or ICF without an IRB/EC-approved amendment. Regulatory approvals will also be sought as required by regulatory guidance.

16. PREMATURE TERMINATION OF THE STUDY OR CLOSE-OUT OF A SITE

16.1. Premature Termination of the Study

The sponsor has the right to terminate the study prematurely. Reasons may include efficacy, safety, or futility, among others. Should the sponsor decide to terminate the study, the investigator(s) will be notified in writing.

16.2. Close-out of a Site

The sponsor and the investigator have the right to close-out a site prematurely.

Investigator's Decision

The investigator must notify the sponsor of a desire to close-out a site in writing, providing at least 30 days' notice. The final decision should be made through mutual agreement with the sponsor. Both parties will arrange the close-out procedures after review and consultation.

Sponsor's Decision

The sponsor will notify the investigator(s) of a decision to close-out a study site in writing. Reasons may include the following, among others:

- The investigator has received all items and information necessary to perform the study, but has not enrolled any patient within a reasonable period of time
- The investigator has violated any fundamental obligation in the study agreement, including but not limited to, breach of this protocol (and any applicable amendments), breach of the applicable laws and regulations, or breach of any applicable ICH guidelines
- The total number of patients required for the study are enrolled earlier than expected

In all cases, the appropriate IRB/EC and Health Authorities must be informed according to applicable regulatory requirements, and adequate consideration must be given to the protection of the patients' interests.

17. STUDY DOCUMENTATION

17.1. Certification of Accuracy of Data

A declaration assuring the accuracy and content of the data recorded on the eCRF must be signed electronically by the investigator. This signed declaration accompanies each set of patient final eCRF that will be provided to the sponsor.

17.2. Retention of Records

The investigator must retain all essential study documents, including ICFs, source documents, investigator copies of eCRFs, and drug accountability records for at least 15 years following the completion or discontinuation of the study, or longer, if a longer period is required by relevant

regulatory authorities. The investigator must consult with the sponsor before discarding or destroying any essential study documents following study completion or discontinuation. Records must be destroyed in a manner that ensures confidentiality.

If the investigator's personal situation is such that archiving can no longer be ensured, the investigator must inform the sponsor and the relevant records will be transferred to a mutually agreed-upon destination.

18. CONFIDENTIALITY

Confidentiality of information is provided as a separate agreement.

19. FINANCING AND INSURANCE

Financing and insurance information is provided as a separate agreement.

20. PUBLICATION POLICY

The publication policy is provided as a separate agreement.

21. REFERENCES

ALIMTA [package insert]. Indianapolis, IN: Eli Lilly and Company; 2013.

ALIMTA [European Union Summary of Product Characteristics]. Indianapolis, IN: Eli Lilly and Company; 2013.

Antonia S, Goldberg SB, Balmanoukian A, Chaft JE, Sanborn RE, Gupta A, et al. Safety and antitumour activity of durvalumab plus tremelimumab in non-small cell lung cancer: a multicentre, phase 1b study. Lancet Oncol. 2016;17(3):299-308.

Antonia SJ, Brahmer JR, Gettinger SN, Quan Man Chow L, Juergens RA, Shepherd FA, et al. 2014 ASCO Annual Meeting: Nivolumab (anti-PD-1; BMS-936558, ONO-4538) in combination with platinum-based doublet chemotherapy (PT-DC) in advanced non-small cell lung cancer (NSCLC). J Clin Oncol. 2014;32:5s(suppl; abstr 8113).

Bergman B, Aaronson NK, Ahmedzai S, Kaasa S, Sullivan M. The EORTC QLQ-LC13: a modular supplement to the EORTC Core Quality of Life Questionnaire (QLQ-C30) for use in lung cancer clinical trials. EORTC Study Group on Quality of Life. Eur J Cancer. 1994;30A(5):635-42.

Besse B, Adjei A, Baas P, Meldgaard P, Nicolson M, Paz-Ares L, et al. 2nd ESMO consensus conference on lung cancer: non-small-cell lung cancer first-line/second and further lines of treatment in advanced disease. Ann Oncol. 2014;25:1475-84.

Bjordal K, de Graeff A, Fayers PM, Hammerlid E, van Pottelsberghe C, Curran D, et al. A 12 country field study of the EORTC QLQ-C30 (version 3.0) and the head and neck cancer specific module (EORTC QLQ-H;N35) in head and neck patients. EORTC Quality of Life Group. Eur J Cancer. 2000;36(14):1796-807.

Borghaei H, Paz-Ares L, Horn L, Spigel DR, Steins M, Ready NE, et al. Nivolumab versus docetaxel in advanced non-squamous non-small-cell lung cancer. N Engl J Med. 2015;373:1627-39.

Brahmer J, Reckamp KL, Baas P, Crinò L, Eberhardt WE, Poddubskaya E, et al. Nivolumab versus docetaxel in advanced squamous-cell non-small-cell lung cancer. N Engl J Med. 2015;373:123-35.

Bray F, Ren JS, Masuyer E, Ferlay J. Estimates of global cancer prevalence for 27 sites in the adult population in 2008. Int J Cancer. 2013;132:1133-45.

Buchbinder EI, Desai A. CTLA-4 and PD-1 pathways: similarities, differences, and implications of their inhibition. Am J Clin Oncol. 2016;39:98-106.

Creelan BC. Update on immune checkpoint inhibitors in lung cancer. Cancer Control. 2014;21:80-9.

D’Incecco A, Andreozzi M, Ludovini V, Rossi E, Capodanno A, Landi L, et al. PD-1 and PD-L1 expression in molecularly selected non-small-cell lung cancer patients. Br J Cancer. 2015;112:95-102.

VV-RIM-00023209-1.0 Approved - 29 Aug 2017 GMT-5:00

Eisenhauer EA, Therasse P, Bogaerts J, Schwartz LH, Sargent D, Ford R, et al. New response evaluation criteria in solid tumours: revised RECIST guideline (version 1.1). Eur J Cancer. 2009;45:228-47.

El-Shenshawy HM, Taema S, El-Zahaf E, El-Beshbeshi W, Eldeen DS, Fathy A. Advanced non-small cell lung cancer in elderly patients: the standard every 3-weeks versus weekly paclitaxel with carboplatin. Chest. 2012;61:485-93.

Ettinger DS, Wood DE, Akerley W, Bazhenova LA, Borghaei H, Camidge DR, et al. NCCN Guidelines insights: non-small cell lung cancer, version 4.2016. J Natl Compr Canc Netw. 2016;14:255-64.

European Medical Agency (EMA) Guideline on the Evaluation of Anticancer Medicinal Products in Man. December 2015. http://www.ema.europa.eu/docs/en_GB/document_library/Scientific_guideline/2016/03/WC500203320.pdf

Fehrenbacher L, Spira A, Ballinger M, Kowanetz M, Vansteenkiste J, Mazieres J, et al. Atezolizumab versus docetaxel for patients with previously treated non-small-cell lung cancer (POPLAR): a multicentre, open-label, phase 2 randomised controlled trial. Lancet. 2016;387(10030):1837-46.

Food and Drug Administration (FDA) Guidance for Industry on Clinical Trial Endpoints for the Approval of Non-Small Cell Lung Cancer Drugs and Biologics. April 2015. https://www.fda.gov/downloads/Drugs/GuidanceComplianceRegulatoryInformation/Guidances/UCM259421.pdf

Fossella F, Pereira JR, von Pawel J, Pluzanska A, Gorbounova V, Kaukel E, et al. Randomized, multinational, phase III study of docetaxel plus platinum combinations versus vinorelbine plus cisplatin for advanced non-small-cell lung cancer: the TAX 326 study group. J Clin Oncol. 2003;21:3016-24.

Freshwater T, Kondic A, Ahamadi M, Li CH, de Greef R, de Alwis D, Stone JA, et al. Evaluation of dosing strategy for pembrolizumab for oncology indications. J Immunother Cancer. 2017;16(5):43.

Gadgeel SM, Stevenson J, Langer C, Gandhi L, Borghaei H, Patnaik A, et al. Pembrolizumab (pembro) plus chemotherapy as front-line therapy for advanced NSCLC: KEYNOTE-021 cohorts A-C. J Clin Oncol. 2016;34(Suppl); abstract 9016.

Gainor J. Moving programmed death-1 inhibitors to the front lines in non-small-cell lung cancer. J Clin Oncol. 2016;34:2953-5.

Garon EB, Rizvi NA, Hui R, Leighl N, Balmanoukian AS, Eder JP, et al. Pembrolizumab for the treatment of non-small-cell lung cancer. N Engl J Med. 2015;372:2018-28.

Gettinger SN, Hellmann MD, Shepherd FA, Antonia SJ, Brahmer JR, Man Chow LQ, et al. First-line monotherapy with nivolumab (NIVO; anti-programmed death-1 [PD-1]) in advanced non-small cell lung cancer (NSCLC): safety, efficacy and correlation of outcomes with PD-1 ligand (PD-L1) expression. J Clin Oncol. 2015;33(Suppl); abstract 8025.

CONFIDENTIAL

VV-RIM-00023209-1.0 Approved - 29 Aug 2017 GMT-5:00

Gettinger S, Rizvi NA, Chow LQ, Borghaei H, Brahmer J, Ready N, et al. First-line nivolumab monotherapy and nivolumab plus ipilimumab in patients with advanced NSCLC: Long-term outcomes from CheckMate 012. IASLC 17th World Congress. 2016;Abstract OA03.01.

Hellmann MD, Rizvi NA, Goldman JW, Gettinger SN, Borghaei H, Brahmer JR, et al. Nivolumab plus ipilimumab as first-line treatment for advanced non-small-cell lung cancer (CheckMate 012): results of an open-label, phase 1, multicohort study. Lancet Oncol. 2017;18:31-41.

Herbst RS, Baas P, Kim DW, Felip E, Pérez-Gracia JL, Han JY, et al. Pembrolizumab versus docetaxel for previously treated, PD-L1-positive, advanced non-small-cell lung cancer (KEYNOTE-010): a randomised controlled trial. Lancet. 2016;387:1540-50.

Hofmann L, Forschner A, Loquai C, Goldinger SM, Zimmer L, Uqurel S, et al. Cutaneous, gastrointestinal, hepatic, endocrine, and renal side-effects of anti-PD-1 therapy. Eur J Cancer 2016;60:190-209.

Kelly WK, Curley T, Slovin S, Heller G, McCaffrey J, Bajorin D, et al. Paclitaxel, estramustine phosphate, and carboplatin in patients with advanced prostate cancer. J Clin Oncol. 2001;19:44-53.

Kerr KM, Tsao MS, Nicholson AG, Yatabe Y, Wistuba II, Hirsch FR; IASLC Pathology Committee. Programmed death-ligand 1 immunohistochemistry in lung cancer: in what state is this art? J Thorac Oncol. 2015;10:985-9.

KEYTRUDA [package insert]. Whitehouse Station, NY: Merck & Co; 2015.

Langer CJ, Gadgeel SM, Borghaei H, Papadimitrakopoulou VA, Patnaik A, Powell SF, et al. Carboplatin and pemetrexed with or without pembrolizumab for advanced, non-squamous non-small-cell lung cancer: a randomised, phase 2 cohort of the open-label KEYNOTE-021 study. Lancet Oncol. 2016;17:1497-508.

Larkin J, Chiarion-Sileni V, Gonzalez R, Grob JJ, Cowey CL, Lao CD, et al. Combined nivolumab and ipilimumab or monotherapy in untreated melanoma. N Engl J Med. 2015;373:23-34.

Leighl NB. Treatment paradigms for patients with metastatic non-small-cell lung cancer: first-, second-, and third-line. Curr Oncol. 2012;19:S52-8.

Liu SV, Powderly JD, Camidge DR, Ready N, Suk Heist R, Hodi FS, et al. 2015 ASCO Annual Meeting. Safety and efficacy of MODL3280A (anti-PDL1) in combination with platinum-based doublet chemotherapy in patients with advanced non-small cell lung cancer (NSCLC). J Clin Oncol. 2015;33:(suppl; abstr 8030).

Lynch TJ, Bondarenko I, Luft A, Serwatowski P, Barlesi F, Chacko R, et al. Ipilimumab in combination with paclitaxel and carboplatin as first-line treatment in stage IIIB/IV non–small cell lung cancer: results from a randomized, double-blind, multicenter phase II study. J Clin Oncol. 2012;30:2046-54.

MYSTIC Press Release, 27 July 2017. https://www.astrazeneca.com/media-centre/press-releases/2017/astrazeneca-reports-initial-results-from-the-ongoing-mystic-trial-in-stage-iv-lung-cancer-27072017.html.

Naidoo J, Page DB, Li BT, Connell LC, Schindler K, Lacouture ME, et al. Toxicities of the anti-PD-1 and anti-PD-L1 immune checkpoint antibodies. Annals of Oncology (2015) 26: 2375-2391.

O'Day SJ, Maio M, Chiarion-Sileni V, Gajewski TF, Pehamberger H, Bondarenko IN, et al. Efficacy and safety of ipilimumab monotherapy in patients with pretreated advanced melanoma: a multicenter single-arm phase II study. Ann Oncol. 2010;21:1712-7.

OPDIVO [package insert]. Princeton, NJ: Bristol-Myers Squibb Company; 2015.

OPDIVO [Summary of Product Characteristics]. Princeton, NJ: Bristol-Myers Squibb Company; 2015.

Papadimitrakopoulou V, Patnaik A, Borghaei H, Stevenson J, Gandhi L, Gubens MA, et al. 2015 ASCO Annual Meeting. Pembrolizumab (pembro; MK-3475) plus platinum doublet chemotherapy (PDC) as front-line therapy for advanced non-small cell lung cancer (NSCLC): KEYNOTE-021 Cohorts A and C. J Clin Oncol. 2015;33:(suppl; abstr 8031).

Paz-Ares LG, de Marinis F, Dediu M, Thomas M, Pujol JL, Bidoli P, et al. PARAMOUNT: Final overall survival results of the phase III study of maintenance pemetrexed versus placebo immediately after induction treatment with pemetrexed plus cisplatin for advanced non-squamous non-small-cell lung cancer. J Clin Oncol. 2013;31:2895-902.

PD-L1 IHC 22C3 pharm Dx [package insert]. Carpinteria, CA: Dako; 2016.

Ratcliffe MJ, Sharpe A, Midha A, Barker C, Scott M, Scorer P, et al. Agreement between programmed cell death ligand-1 diagnostic assays across multiple protein expression cut-offs in non-small cell lung cancer. Clin Cancer Res. 2017 Jan 10. [Epub ahead of print].

Reck M, Popat S, Reinmuth N, De Ruysscher D, Kerr KM, Peters S; ESMO Guidelines Working Group. Metastatic non-small-cell lung cancer (NSCLC): ESMO clinical practice guidelines for diagnosis, treatment and follow-up. Ann Oncol. 2014;25 Suppl 3:iii27-39.

Reck M, Rodríguez-Abreu D, Robinson AG, Hui R, Csőszi T, Fülöp A, et al. Pembrolizumab versus chemotherapy for PD-L1-positive non-small-cell lung cancer. N Engl J Med. 2016;375(19):1823-33.

Rittmeyer A, Barlesi F, Waterkamp D, Park K, Ciardiello F, von Pawel J, et al. Atezolizumab versus docetaxel in patients with previously treated non-small-cell lung cancer (OAK): a phase 3, open-label, multicentre randomised controlled trial. Lancet. 2017;389(10066):255-265.

Rizvi NA, Hellmann MD, Brahmer JR, Juergens RA, Borghaei H, Gettinger S, et al. Nivolumab in combination with platinum-based doublet chemotherapy for first-line treatment of advanced non-small-cell lung cancer. J Clin Oncol. 2016;34:2969-79.

Roach C, Zhang N, Corigliano E, Jansson M, Tolad G, Ponto G, et al. Development of a companion diagnostic PD-L1 immunohistochemistry assay for pembrolizumab therapy in non-small-cell lung cancer. Appl Immunohistochem Mol Morphol. 2016;24:392–7.

Rosell R, Gatzemeier U, Betticher DC, Keppler U, Macha HN, Pirker R, et al. Phase III randomised trial comparing paclitaxel/carboplatin with paclitaxel/cisplatin in patients with advanced non-small-cell lung cancer: a cooperative multinational trial. Ann Oncol. 2002;13:1539-49.

Scagliotti GV, De Marinis F, Rinaldi M, Crinò L, Gridelli C, Ricci S, et al. Phase III randomized trial comparing three platinum-based doublets in advanced non-small-cell lung cancer. J Clin Oncol. 2002;20:4285-91.

Schiller JH, Harrington D, Belani CP, Langer C, Sandler A, Krook J, et al. Comparison of four chemotherapy regimens for advanced non-small-cell lung cancer. N Engl J Med. 2002;346:92-8.

Shimizu T, Seto T, Hirai F, Takenoyama M, Nosaki K, Tsurutani J, et.al. Phase 1 study of pembrolizumab (MK-3475; anti-PD-1 monoclonal antibody) in Japanese patients with advanced solid tumors. Invest New Drugs (2016);34:347-54.

Shimizu T, Yokoi T, Tamaki T, Kibata K, Inagaki N, Nomura S. Comparative analysis of carboplatin and paclitaxel combination chemotherapy schedules in previously untreated patients with advanced non-small cell lung cancer. Oncol Lett. 2013;5:761-7.

Siegel RL, Miller KD, Jemal A. Cancer statistics, 2016. CA Cancer J Clin. 2016;66:7-30.

Slovin SF, Higano CS, Hamid O, Tejwani S, Harzstark A, Alumkal JJ, et al. Ipilimumab alone or in combination with radiotherapy in metastatic castration-resistant prostate cancer: results from an open-label, multicenter phase I/II study. Ann Oncol. 2013;24:1813-21.

Socinski M, Creelan B, Horn L, Reck M, Paz-Are L, Steins M, et al. CheckMate 026: A phase 3 trial of nivolumab vs investigator’s choice (IC) of platinum-based doublet chemotherapy (PT-DC) as first-line therapy for stage IV/recurrent programmed death ligand 1 (PD-L1)-positive NSCLC. Annals of Oncology. 2016;27 Suppl 6:LBA7_PR.

Topalian SL, Hodi FS, Brahmer JR, Gettinger SN, Smith DC, McDermott DF, et al. Safety, activity, and immune correlates of anti–PD-1 antibody in cancer. N Engl J Med. 2012;366:2443-54.

Vinay DS, Ryan EP, Pawelec G, Talib WH, Stagg J, Elkord E, et al. Immune evasion in cancer: mechanistic basis and therapeutic strategies. Semin Cancer Biol. 2015;35:S185-98.

Weber JS, Yang JC, Atkins MB, Disis ML. Toxicities of immunotherapy for the practitioner. J Clin Oncol. 2015;33:2092-9.

Wolchok JD, Neyns B, Linette G, Negrier S, Lutzky J, Thomas L, et al. Ipilimumab monotherapy in patients with pretreated advanced melanoma: a randomised, double-blind, multicentre, phase 2, dose-ranging study. Lancet Oncol. 2010;11:155-64.

YERVOY [package insert]. Princeton, NJ: Bristol-Myers Squibb Company; 2017.

YERVOY [Summary of Product Characteristics]. Princeton, NJ: Bristol-Myers Squibb Company; 2016.

Zhao X, Suryawanshi S, Hruska M, Feng Y, Wang X, Shen J, et al. Assessment of nivolumab benefit-risk profile of a 240-mg flat dose relative to a 3-mg/kg dosing regimen in patients with advanced tumors. Ann Oncol. 2017a; doi: 10.1093/annonc/mdx235.

Zhao X, Feng Y, Wang X, Young TC, Maier S, Bello S, et al. Tumor growth dynamic (TGD) modeling and safety analysis of nivolumab (nivo) plus ipilimumab (ipi) in first-line (1L) patients (PTS) with non-small cell lung cancer (NSCLC). ASPCT 2017b Annual Meeting.

Zimmer L, Goldinger SM, Hofmann L, et al. Neurological, respiratory, musculoskeletal, cardiac and ocular side-effects of anti-PD-1 therapy. Eur J Cancer 2016;60 210e225.

VV-RIM-00023209-1.0 Approved - 29 Aug 2017 GMT-5:00

22. INVESTIGATOR'S AGREEMENT

I have read the attached protocol: A Randomized, Phase 3, Open-Label Study of Combinations of REGN2810 (Anti-PD-1 Antibody), Ipilimumab (Anti-CTLA-4 Antibody), and Platinum-Based Doublet Chemotherapy in First-Line Treatment of Patients With Advanced or Metastatic Non-Small Cell Lung Cancer With Tumors Expressing PD-L1 <50% and agree to abide by all the provisions set forth therein.

I agree to comply with the current International Council for Harmonisation Guideline for Good Clinical Practice and the laws, rules, regulations, and guidelines of the community, country, state, or locality relating to the conduct of the clinical study.

I also agree that persons debarred from conducting or working on clinical studies by any court or regulatory agency will not be allowed to conduct or work on studies for the sponsor or a partnership in which the sponsor is involved. I will immediately disclose it in writing to the sponsor if any person who is involved in the study is debarred, or if any proceeding for debarment is pending, or, to the best of my knowledge, threatened.

This document contains confidential information of the sponsor, which must not be disclosed to anyone other than the recipient study staff and members of the IRB/EC. I agree to ensure that this information will not be used for any purpose other than the evaluation or conduct of the clinical investigation without the prior written consent of the sponsor.

______________________________ ____________________

(Signature of Investigator) (Date)

____

(Printed Name)

VV-RIM-00023209-1.0 Approved - 29 Aug 2017 GMT-5:00

APPENDIX 1. FACTORS TO CONSIDER IN ASSESSING THE RELATIONSHIP OF ADVERSE EVENTS TO STUDY DRUG AND STUDY CONDUCT

Is there a reasonable possibility that the event may have been caused by the study drug or study conduct?

No:

- Due to external causes such as environmental factors or other treatment(s) being administered
- Due to the patient's disease state or clinical condition
- Do not follow a reasonable temporal sequence following the time of administration of the dose of study drug
- Do not reappear or worsen when dosing with study drug is resumed
- Are not a suspected response to the study drug based upon pre-clinical data or prior clinical data

Yes:

- Could not be explained by environmental factors or other treatment(s) being administered
- Could not be explained by the patient's disease state or clinical condition
- Follow a reasonable temporal sequence following the time of administration of the dose of study drug
- Resolve or improve after discontinuation of study drug
- Reappear or worsen when dosing with study drug is resumed
- Are known or suspected to be a response to the study drug based upon pre-clinical data or prior clinical data

NOTE: This list is not exhaustive.

VV-RIM-00023209-1.0 Approved - 29 Aug 2017 GMT-5:00

APPENDIX 2. RESPONSE EVALUATION CRITERIA IN SOLID TUMORS: RECIST GUIDELINE (VERSION 1.1)

This appendix has been excerpted from the revised Response Evaluation Criteria in Solid Tumors (RECIST) guideline (version 1.1; Eisenhauer 2009). For full details pertaining to the RECIST 1.1 criteria, please refer to the publication.

1. Assessment of Tumor Burden Measurable Disease at Baseline

Overall tumor burden must be assessed at baseline and will be used as a comparator for subsequent measurements. Tumor lesions will be characterized as measurable or non-measurable as follows:

Response and progression will be evaluated in this study using the international criteria proposed by the revised RECIST guideline (version 1.1; Eisenhauer 2009). Changes in the largest diameter (unidimensional measurement) of the tumor lesions and the shortest diameter in the case of malignant lymph nodes are used in the RECIST criteria.

1.1. Measurable disease

Tumor lesions must be accurately measured in at least one dimension (longest diameter in the plane of measurement is to be recorded) with a minimum size of

- 10 mm by CT scan (CT scan slice thickness no greater than 5 mm)
- 10 mm caliper measurement by clinical exam (lesions which cannot be accurately measured with calipers should be recorded as non-measurable)
- 20 mm by chest X-ray
- **Malignant lymph nodes:** To be considered pathologically enlarged and measurable, a lymph node must be ≥15 mm (≥1.5 cm) in short axis when assessed by CT scan (CT scan slice thickness recommended to be no greater than 5 mm [0.5 cm]). At baseline and in follow-up, only the short axis will be measured and followed.

1.2. Nonmeasurable disease

All other lesions, including small lesions (longest diameter <10 mm or pathological lymph nodes with ≥10 to <15 mm short axis), are considered nonmeasurable disease. Bone lesions, leptomeningeal disease, ascites, pleural/pericardial effusions, lymphangitis cutis/pulmonitis, inflammatory breast disease, and abdominal masses (not followed by CT, MRI, or positron emission tomography [PET]) are considered as nonmeasurable.

1.2.1. Special Considerations

Bone lesions:

- Bone scan, PET scan or plain films are not considered adequate imaging techniques to measure bone lesions. However, these techniques can be used to confirm the presence or disappearance of bone lesions.
- Lytic bone lesions or mixed lytic-blastic lesions, with identifiable soft tissue components, that can be evaluated by cross sectional imaging techniques such as CT

or MRI can be considered as measurable lesions if the soft tissue component meets the definition of measurability described above.

- Blastic bone lesions are non-measurable.

Cystic lesions:

Cystic lesions that meet the criteria for radiographically defined simple cysts should not be considered as malignant lesions (neither measurable nor nonmeasurable) since they are, by definition, simple cysts. “Cystic lesions” thought to represent cystic metastases can be considered as measurable lesions, if they meet the definition of measurability described above. However, if noncystic lesions are present in the same patient, these are preferred for selection as target lesions.

Lesions with prior local treatment:

- Tumor lesions situated in a previously irradiated area, or in an area subjected to other locoregional therapy, are usually not considered measurable unless there has been demonstrated progression in the lesion.

1.3. Methods of Assessment

All measurements must be recorded in metric notation, using calipers if clinically assessed. All baseline evaluations should be performed as closely as possible to the beginning of treatment and never more than 4 weeks before the beginning of the treatment.

The same method of assessment and the same technique should be used to characterize each identified and reported lesion at baseline and during follow-up. Imaging-based evaluation is preferred to evaluation by clinical examination unless the lesion(s) being followed cannot be imaged but are assessable by clinical exam.

- **Clinical lesions**. Clinical lesions will only be considered measurable when they are superficial and ≥10 mm (≥1 cm) in diameter as assessed using calipers (eg, skin nodules).
- **Chest X-ray**. Chest CT is preferred over chest X-ray, particularly when progression is an important endpoint, since CT is more sensitive than X-ray, particularly in identifying new lesions. Lesions on chest X-ray may be considered measurable if they are clearly defined and surrounded by aerated lung.
- **CT and MRI**. CT is the best currently available and reproducible method to measure lesions selected for response assessment. This guideline has defined measurability of lesions on CT scan based on the assumption that CT slice thickness is 5mm or less. When CT scans have slice thickness greater than 5 mm, the minimum size for a measurable lesion should be twice the slice thickness. MRI is also acceptable in certain situations (e.g. for body scans).
- **PET-CT**. At present, the low dose or attenuation correction CT portion of a combined PET-CT is not always of optimal diagnostic CT quality for use with RECIST measurements. However, if the site can document that the CT performed as part of a PET-CT is of identical diagnostic quality to a diagnostic CT (with intravenous and oral contrast), then the CT portion of the PET-CT can be used for

RECIST measurements and can be used interchangeably with conventional CT in accurately measuring cancer lesions over time. Note, however, that the PET portion of the CT introduces additional data that may bias an investigator if it is not routinely or serially performed.

- **Ultrasound**. Ultrasound is not useful in assessment of lesion size and should not be used as a method of measurement.
- **Endoscopy, laparoscopy**. The utilization of these techniques for objective tumor evaluation is not advised.
- **Tumor markers**. Tumor markers alone cannot be used to assess response.
- **Cytology, histology**. These techniques can be used to differentiate between PRs and CRs in rare cases (eg, residual lesions in tumor types, such as germ cell tumors, where known residual benign tumors can remain).

1.4. Baseline Documentation of Target and Non-Target Lesions

Target lesions: When more than one measurable lesion is present at baseline all lesions up to a maximum of 5 lesions total (and a maximum of 2 lesions per organ) representative of all involved organs should be identified as target lesions and will be recorded and measured at baseline. Target lesions should be selected on the basis of their size (lesions with the longest diameter), be representative of all involved organs, and should lend themselves to reproducible repeated measurements.

Lymph nodes merit special mention since they are normal anatomical structures which may be visible by imaging even if not involved by tumor. Pathological nodes which are defined as measurable and may be identified as target lesions must meet the criterion of a short axis of ≥15 mm by CT scan. Only the short axis of these nodes will contribute to the baseline sum. All other pathological nodes (those with short axis ≥10 mm but <15 mm) should be considered non-target lesions. Nodes that have a short axis <10 mm are considered non-pathological and should not be recorded or followed.

A sum of the diameters (longest for non-nodal lesions, short axis for nodal lesions) for all target lesions will be calculated and reported as the baseline sum diameters. The baseline sum diameters will be used as reference to further characterize any objective tumor regression in the measurable dimension of the disease.

Non-target lesions: All other lesions (or sites of disease) including pathological lymph nodes should be identified as non-target lesions and should also be recorded at baseline. Measurements of these lesions are not required, but the presence, absence, or, in rare cases, unequivocal progression of each should be noted throughout follow-up. In addition, it is possible to record multiple non-target lesions involving the same organ as a single item on the case record form (eg, ‘multiple enlarged pelvic lymph nodes’ or ‘multiple liver metastases’).

1.5. Response Criteria

This section provides the definitions of the criteria used to determine objective tumor response for target and non-target lesions.

CONFIDENTIAL

VV-RIM-00023209-1.0 Approved - 29 Aug 2017 GMT-5:00

1.5.1. Evaluation of Target Lesions

- **Complete Response (CR):** Disappearance of all target lesions. Any pathological lymph nodes (whether target or non-target) must have reduction in short axis to <10 mm (<1 cm).
- **Partial Response (PR):** At least a 30% decrease in the sum of the diameters of target lesions, taking as reference the baseline sum diameters.
- **Progressive Disease (PD):** At least a 20% increase in the sum of the diameters of target lesions, taking as reference the smallest sum on study (this includes the baseline sum if that is the smallest on study). In addition to the relative increase of 20%, the sum must also demonstrate an absolute increase of at least 5 mm (0.5 cm). (**Note:** the appearance of one or more new lesions is also considered progressions).
- **Stable Disease (SD):** Neither sufficient shrinkage to qualify for PR nor sufficient increase to qualify for progressive disease, taking as reference the smallest sum diameters while on study.

<u>Special notes on the assessment of target lesions:</u>

- Lymph nodes: Lymph nodes identified as target lesions should always have the actual short axis measurement recorded and should be measured in the same anatomical plane as the baseline examination, even if the nodes regress to below 10 mm on study. This means that when lymph nodes are included as target lesions, the ‘sum’ of lesions may not be zero even if CR criteria are met, since a normal lymph node is defined as having a short axis of <10 mm.
- Target lesions that become ‘too small to measure’: All lesions (nodal and non-nodal) recorded at baseline should have their actual measurements recorded at each subsequent evaluation, even when very small (eg, 2 mm). If the radiologist is able to provide an actual measure, that should be recorded, even if it is below 5 mm. However, when such a lesion becomes difficult to assign an exact measure to then: (i) if it is the opinion of the radiologist that the lesion has likely disappeared, the measurement should be recorded as 0 mm. (ii) if the lesion is believed to be present and is faintly seen but too small to measure, a default value of 5 mm should be assigned (note: in case of a lymph node believed to be present and faintly seen but too small to measure, a default value of 5 mm should be assigned in this circumstance as well). This default value is derived from the 5 mm CT slice thickness (but should not be changed with varying CT slice thickness).
- Lesions that split or coalesce on treatment: When non-nodal lesions ‘fragment’, the longest diameters of the fragmented portions should be added together to calculate the target lesion sum. Similarly, as lesions coalesce, a plane between them may be maintained that would aid in obtaining maximal diameter measurements of each individual lesion. If the lesions have coalesced such that they are no longer separable, the vector of the longest diameter in this instance should be the maximal longest diameter for the ‘coalesced lesion’.

1.5.2. Evaluation of Non-target Lesions

While some non-target lesions may actually be measurable, they need not be measured and instead should be assessed only qualitatively at the time points specified in the protocol.

- **Complete Response:** Disappearance of all nontarget lesions and normalization of tumor marker level. All lymph nodes must be nonpathological in size (<10 mm short axis).
- **Non-CR/Non-PD:** Persistence of one or more nontarget lesion(s) and/or maintenance of tumor marker level above the normal limits.
- **Progressive Disease:** Appearance of one or more new lesions and/or unequivocal progression of existing nontarget lesions. Unequivocal progression should not normally trump target lesion status. It must be representative of overall disease status change, not a single lesion increase.

Although a clear progression of "nontarget" lesions only is exceptional, the opinion of the treating physician should prevail in such circumstances, and the progression status should be confirmed at a later time by the review panel (or investigator).

1.6. New Lesions

The appearance of new malignant lesions denotes disease progression; therefore, some comments on detection of new lesions are important. There are no specific criteria for the identification of new radiographic lesions; however, the finding of a new lesion should be unequivocal: ie not attributable to differences in scanning technique, change in imaging modality or findings thought to represent something other than tumor (for example, some 'new' bone lesions may be simply healing or flare of pre-existing lesions). This is particularly important when the patient's baseline lesions show PR or CR. For example, necrosis of a liver lesion may be reported on a CT scan report as a 'new' cystic lesion, which it is not.

A lesion identified on a follow-up study in an anatomical location that was not scanned at baseline is considered a new lesion and will indicate disease progression. An example of this is the patient who has visceral disease at baseline and while on study has a CT or MRI brain ordered which reveals metastases. The patient's brain metastases are considered to be evidence of progressive disease even if he/she did not have brain imaging at baseline. If a new lesion is equivocal, for example because of its small size, continued therapy and follow-up evaluation will clarify if it truly represents new disease. If repeat scans confirm there is definitely a new lesion, then progression should be declared using the date of the initial scan.

While FDG-PET response assessments need additional study, it is sometimes reasonable to incorporate the use of FDG-PET scanning to complement CT scanning in assessment of progression (particularly possible 'new' disease). New lesions on the basis of FDG-PET imaging can be identified according to the following algorithm:

a. Negative FDG-PET at baseline, with a positive FDG-PET at follow-up is a sign of progressive disease based on a new lesion.
b. No FDG-PET at baseline and a positive FDG-PET at follow-up: If the positive FDG-PET at follow-up corresponds to a new site of disease confirmed by CT, this is progressive disease. If the positive FDG-PET at follow-up is not confirmed as a new

site of disease on CT, additional follow-up CT scans are needed to determine if there is truly progression occurring at that site (if so, the date of progressive disease will be the date of the initial abnormal FDG-PET scan). If the positive FDG-PET at follow-up corresponds to a preexisting site of disease on CT that is not progressing on the basis of the anatomic images, this is not progressive disease.

c. FDG-PET may be used to upgrade a response to a CR in a manner similar to a biopsy in cases where a residual radiographic abnormality is thought to represent fibrosis or scarring. The use of FDG-PET in this circumstance should be prospectively described in the protocol and supported by disease-specific medical literature for the indication. However, it must be acknowledged that both approaches may lead to false-positive CR due to limitations of FDG-PET and biopsy resolution/sensitivity.

Note: A "positive" FDG-PET scan lesion means one which is FDG avid with an uptake greater than twice that of the surrounding tissue on the attenuation corrected image.

1.7. Evaluation of Best Overall Response

The BOR is the best response recorded from the start of the treatment until the end of treatment taking into account any requirement for confirmation. The patient's best response assignment will depend on the findings of both target and non-target disease and will also take into consideration the appearance of new lesions.

Revised RECIST 1.1 (Eisenhauer 2009) is summarized in Table 7.

Table 7: Response According to Revised Response Evaluation Criteria in Solid Tumors (Version 1.1) in Patients with Target (and Non-Target) Lesions

Target Lesions	Non-target Lesions	New Lesions	Overall Response*
CR	CR	No	CR
CR	Non-CR/non-PD	No	PR
CR	Not evaluated	No	PR
PR	Non-CR/non-PD/not evaluated	No	PR
SD	Non-CR/non-PD/not evaluated	No	SD
PD	Any	Yes or no	PD
Any	PD*	Yes or no	PD
Any	Any	Yes	PD

Abbreviations: CR=complete response; PD=progressive disease; PR=partial response; SD=stable disease
*In exceptional circumstances, unequivocal progression in non-target lesions may be accepted as PD.
Note: Patients with a global deterioration of health status requiring discontinuation of treatment without objective evidence of disease progression at that time should be reported as "*symptomatic deterioration.*" Every effort should be made to document the objective progression even after discontinuation of treatment.

1.8. Missing Assessments and Unevaluable Designation

When no imaging/measurement is done at all at a particular time point, the patient is not evaluable (NE) at that time point. If only a subset of lesion measurements are made at an assessment, usually the case is also considered NE at that time point, unless a convincing

argument can be made that the contribution of the individual missing lesion(s) would not change the assigned time point response. This would be most likely to happen in the case of progressive disease. For example, if a patient had a baseline sum of 50 mm with 3 measured lesions and at follow-up only 2 lesions were assessed, but those gave a sum of 80 mm, the patient will have achieved progressive disease status, regardless of the contribution of the missing lesion.

1.9. Best Overall Response: All Time Points

The BOR is determined once all the data for the patient is known. Best response determination in studies where confirmation of CR or PR IS NOT required: Best response in these studies is defined as the best response across all time points (for example, a patient who has SD at first assessment, PR at second assessment, and progressive disease on last assessment has a BOR of PR). When SD is believed to be best response, it must also meet the protocol-specified minimum time from baseline. If the minimum time is not met when SD is otherwise the best time point response, the patient’s best response depends on the subsequent assessments. For example, a patient who has SD at first assessment, progressive disease at second and does not meet minimum duration for SD, will have a best response of progressive disease. The same patient lost to follow-up after the first SD assessment would be considered unevaluable.

2.0. Duration of overall response

The duration of overall response is measured from the time measurement criteria are first met for CR/PR (whichever is first recorded) until the first date that recurrent or progressive disease is objectively documented (taking as reference for progressive disease the smallest measurements recorded on study). The duration of overall CR is measured from the time measurement criteria are first met for CR until the first date that recurrent disease is objectively documented.

APPENDIX 3. RECOMMENDED DOSE MODIFICATION OR DISCONTINUATION AND SUPPORTIVE CARE GUIDELINES FOR SPECIFIC REGN2810 DRUG-RELATED ADVERSE EVENTS

CONFIDENTIAL

VV-RIM-00023209-1.0 Approved - 29 Aug 2017 GMT-5:00

Colitis Adverse Event Management

COLITIS CTCAE v4.03 Grade	REGN2810 Dosing Management	Action and Guidelines	Diagnostic Considerations
**Grade 1**<br>Bowel obstruction<br>Colitis<br>Colitis microscopic	No change in REGN2810 dose	• For diarrhea, treat symptomatically (loperamide, oral hydration, electrolyte substitution and ADA colitis diet). Endoscopy is recommended if symptoms persist.<br>• Grade 1 diarrhea that persist for >1 week should be treated with the addition of oral diphenoxylate hydrochloride and atropine sulfate four times daily and budesonide 9 mg daily	All attempts should be made to rule out other causes such as metastatic disease, bacterial or parasitic infection, viral gastroenteritis, or the first manifestation of an inflammatory bowel disease by examination for stool leukocytes, stool cultures, and a Clostridium difficile titer
**Grade 2**<br>Enterocolitis hemorrhagic<br>Gastrointestinal (GI) perforation<br>Necrotizing colitis<br>Diarrhea:<br>All patients who experience diarrhea should be advised to drink liberal quantities of clear fluids.<br>If sufficient oral fluid intake is not feasible, fluid and Electrolytes should be substituted via IV infusion.	Hold until ≤Grade 1.	• GI consultation and endoscopy is recommended to confirm or rule out colitis for grade 2 diarrhea that persists >1 week or grade 1-2 diarrhea with rectal bleeding (additional guidelines for the treatment of persistent colitis are provided below).<br>• Grade 2 diarrhea should be treated with the addition of oral diphenoxylate hydrochloride and atropine sulfate four times daily and budesonide 9 mg daily.<br>• Grade 2 diarrhea with diffuse ulceration and bleeding seen on endoscopy may require oral steroids with prolonged taper and represent an increased risk for the development of bowel perforation.<br>• When symptoms improve to grade 1 or less, steroid taper should be started and continued over no less than 4 weeks.<br>• In patients with Grade 2 enterocolitis, REGN2810 should be withheld and anti-diarrheal treatment should be started. If symptoms are persistent for more than one week, systemic corticosteroids should be initiated (e.g., 0.5 mg/kg/day of prednisone or equivalent). When symptoms improve to Grade 1 or less, corticosteroid taper should be started and continued over at least 1 month.	

CONFIDENTIAL

VV-RIM-00023209-1.0 Approved - 29 Aug 2017 GMT-5:00

Colitis Adverse Event Management

COLITIS<br>CTCAE v4.03 Grade	REGN2810 Dosing Management	Action and Guidelines	Diagnostic Considerations
**Grade 3-4**	Withhold REGN2810<br><br>Discontinue if unable to reduce corticosteroid dose to <10 mg per day prednisone equivalent within 12 weeks of toxicity	• Patients with Grade 3 enterocolitis, drug will be permanently discontinued and treatment with systemic corticosteroids should be initiated at a dose of 1 to 2 mg/kg/day of prednisone or equivalent. When symptoms improve to Grade 1 or less, corticosteroid taper should be started and continued over at least 1 month.<br><br>For Grade 3-4 diarrhea (or Grade 2 diarrhea that persists after initial steroid treatment),<br>• Rule out bowel perforation. Imaging with plain films or computed tomography (CT) can be useful.<br>• Consider consultation with gastroenterologist and confirmation biopsy with endoscopy.<br>• Treat with intravenous (IV) steroids (methylprednisolone 125 mg) followed by high-dose oral steroids (prednisone 1-2 mg/kg once per day or dexamethasone 4 mg every 4 hours). When symptoms improve to grade 1 or less, steroid taper should be started and continued over no less than 4 weeks. Taper over 6-8 weeks in patients with diffuse and severe ulceration and/or bleeding.<br>• If IV steroids followed by high-dose oral steroids does not reduce initial symptoms within 48-72 hours, consider treatment with infliximab at 5 mg/kg once every 2 weeks. Discontinue infliximab upon symptom relief and initiate a prolonged steroid taper over 45-60 days. If symptoms worsen during steroid reduction, initiate a retapering of steroids starting at a higher dose of 80 or 100 mg followed by a more prolonged taper and administer infliximab. CAUTION: infliximab is contraindicated in patients with bowel perforation or sepsis.<br>• If symptoms persist despite the above treatment a surgical consult should be obtained.	All attempts should be made to rule out other causes such as metastatic disease, bacterial or parasitic infection, viral gastroenteritis, or the first manifestation of an inflammatory bowel disease by examination for stool leukocytes, stool cultures, and a Clostridium difficile titer<br><br>If symptoms are persistent And/or severe, endoscopic evaluation should be considered

Endocrine Adverse Event Management

ENDOCRINE EVENTS CTCAE v4.03 Grade	REGN2810 Dosing Management	Action and Guidelines	Diagnostic Considerations
**Grade 1-2**<br>Hyperthyroidism<br>Hypothyroidism<br>Thyroid disorder<br>Thyroiditis	No change in REGN2810 dose	• Monitor thyroid function or other hormonal level tests and serum chemistries more frequently (every 3-6 weeks) until returned to baseline values.<br>• Replacement of thyroid hormone or thyroid suppression therapy as indicated.	All attempts should be made to rule out other causes such as brain metastases, sepsis, and/or infection. An endocrinology consultation is recommended
**Grade 3-4**<br>Hyperthyroidism<br>Hypothyroidism<br>Thyroid disorder<br>Thyroiditis	Hold until on stable replacement dose as determined by resolution of symptoms and normalization of hormone levels.	• Consider endocrine consultation.<br>• Rule out infection and sepsis with appropriate cultures and imaging.<br>• Replacement of thyroid hormone or thyroid suppression therapy as indicated.	

CONFIDENTIAL

VV-RIM-00023209-1.0 Approved - 29 Aug 2017 GMT-5:00

Endocrine Adverse Event Management (Cont)

ENDOCRINE EVENTS CTCAE v4.03 Grade	REGN2810 Dosing Management	Action and Guidelines	Diagnostic Considerations
**Grade 1-4**<br>Adrenal insufficiency<br>Hypophysitis<br>Hypopituitarism<br>Pan-hypopituitarism	Hold until on stable replacement dose	• Thyroid hormone and/or steroid replacement therapy to manage adrenal insufficiency.<br>• If Grade 1–2 hypophysitis is considered, pituitary gland imaging should be considered (magnetic resonance imaging [MRIs] with gadolinium and selective cuts of the pituitary can show enlargement or heterogeneity and confirm the diagnosis).<br>• Grade 3–4 hypophysitis with clinically significant adrenal insufficiency and hypotension, dehydration, and electrolyte abnormalities (such as hyponatremia and hyperkalemia) constitutes adrenal crisis. Hospitalization and IV methylprednisolone should be initiated.	All attempts should be made to rule out other causes such as brain metastases, sepsis, and/or infection. An endocrinology consultation is recommended

CONFIDENTIAL

VV-RIM-00023209-1.0 Approved - 29 Aug 2017 GMT-5:00

Pneumonitis Adverse Event Management

PNEUMONITIS<br>CTCAE v4.03 Grade	REGN2810 Dosing Management	Action and Guidelines	Diagnostic Considerations
**Grade 1**<br>Pneumonitis<br>Interstitial lung disease<br>Acute interstitial pneumonitis	Consider hold of therapy. REGN2810 may be continued with close monitoring.	• Radiologic findings should be followed on serial imaging studies at least every 3 weeks.<br>• Monitor for symptoms every 2–3 days.<br>• Consider pulmonary consultation and/or bronchoscopy if clinically indicated.	All attempts should be made to rule out other causes such as metastatic disease, bacterial or viral infection.
**Grade 2**<br>Pneumonitis<br>Interstitial lung disease<br>Acute interstitial pneumonitis	Hold REGN2810	**To rule out other causes such as infection:**<br>• Consider pulmonary consultation with bronchoscopy and biopsy/bronchoalveolar lavage (BAL).<br>• Consider pulmonary function tests.<br>• Follow radiologic findings on serial imaging studies every 1-3 days<br>**If the patient is determined to have study drug associated pneumonitis:**<br>• Monitor symptoms daily; consider hospitalization.<br>• Treat with systemic corticosteroids at a dose of 1–2 mg/kg/day prednisone or equivalent. When symptoms improve to Grade 1 or less, steroid taper should be started and continued over no less than 4 weeks.<br>• Treatment with REGN2810 may be resumed if the event improves to ≤ Grade 1 within 12 weeks and corticosteroids have been reduced to the equivalent of methylprednisolone 10 mg by mouth daily or less. Repeat chest imaging monthly as clinically indicated.<br>**For Grade 2 pneumonitis that improves to ≤Grade 1 within 12 weeks, the following rules should apply:**<br>• First episode of pneumonitis: May increase dosing interval by one week in subsequent cycles.<br>• Second episode of pneumonitis: Discontinue REGN2810 if upon rechallenge the patient develops a second episode of ≥ Grade 2 pneumonitis.	

Pneumonitis Adverse Event Management (Cont)

PNEUMONITIS CTCAE v4.03 Grade	REGN2810 Dosing Management	Action and Guidelines	Diagnostic Considerations
**Grade 3-4**<br>Pneumonitis<br>Interstitial lung disease<br>Acute interstitial pneumonitis	Discontinue REGN2810	• Consider pulmonary function tests with pulmonary consult.<br>• Bronchoscopy with biopsy and/or BAL is recommended.<br>• Treat with IV steroids (methylprednisolone 125 mg). When symptoms improve to grade 1 or less, a high-dose oral steroid (prednisone 1-2 mg/kg once per day or dexamethasone 4 mg every 4 hours) taper should be started and continued over no less than 4 weeks.<br>• Add prophylactic antibiotics for opportunistic infections<br>• If IV steroids followed by high-dose oral steroids does not reduce initial symptoms within 48-72 hours, treat with infliximab at 5 mg/kg once every 2 weeks. Discontinue infliximab upon symptom relief and initiate a prolonged steroid taper over 45-60 days. If symptoms worsen during steroid reduction, initiate a retapering of steroids starting at a higher dose of 80 or 100 mg followed by a more prolonged taper and administer infliximab.	All attempts should be made to rule out other causes such as metastatic disease, bacterial or viral infection.

Renal Adverse Event Management

RENAL EVENTS CTCAE v4.03 Grade	REGN2810 Dosing Management	Action and Guidelines	Diagnostic Considerations
**Grade 1**<br>Nephritis<br>Nephritis autoimmune	Consider withholding REGN2810 if event does not improve with symptomatic treatment	• Provide symptomatic treatment.<br>• Monitor creatinine weekly; when it returns to baseline, resume routine creatinine monitoring per protocol.	All attempts should be made to rule out other causes such as obstructive uropathy, progression of disease, or injury to other chemotherapy agents. A renal consultation is recommended.
**Grade 2**<br>Renal failure	Consider withholding REGN2810	• Systemic corticosteroids at a dose of 1-2 mg/kg/day of prednisone or equivalent may be indicated. When symptoms improve to Grade 1 or less, corticosteroid taper should be started and continued over at least 1 month.<br>• Consider prophylactic antibiotics for opportunistic infections.<br>• Consider renal biopsy.<br>• If elevations persist >7 days or worsen, treat as Grade 4.	
**Grade 3-4**<br>•Renal failure acute	Discontinue REGN2810	• Renal consultation with consideration of ultrasound and/or biopsy as appropriate.<br>• Monitor creatinine daily.<br>• Treat with systemic corticosteroids at a dose of 1–2 mg/kg prednisone or equivalent once per day.<br>• When symptoms improve to grade 1 or less, steroid taper should be started and continued over no less than 4 weeks.<br>• Discontinue REGN2810 if unable to reduce corticosteroid dose for irAEs to ≤10 mg.<br>• REGN2810 treatment may be restarted and the dose modified as specified in the protocol.	

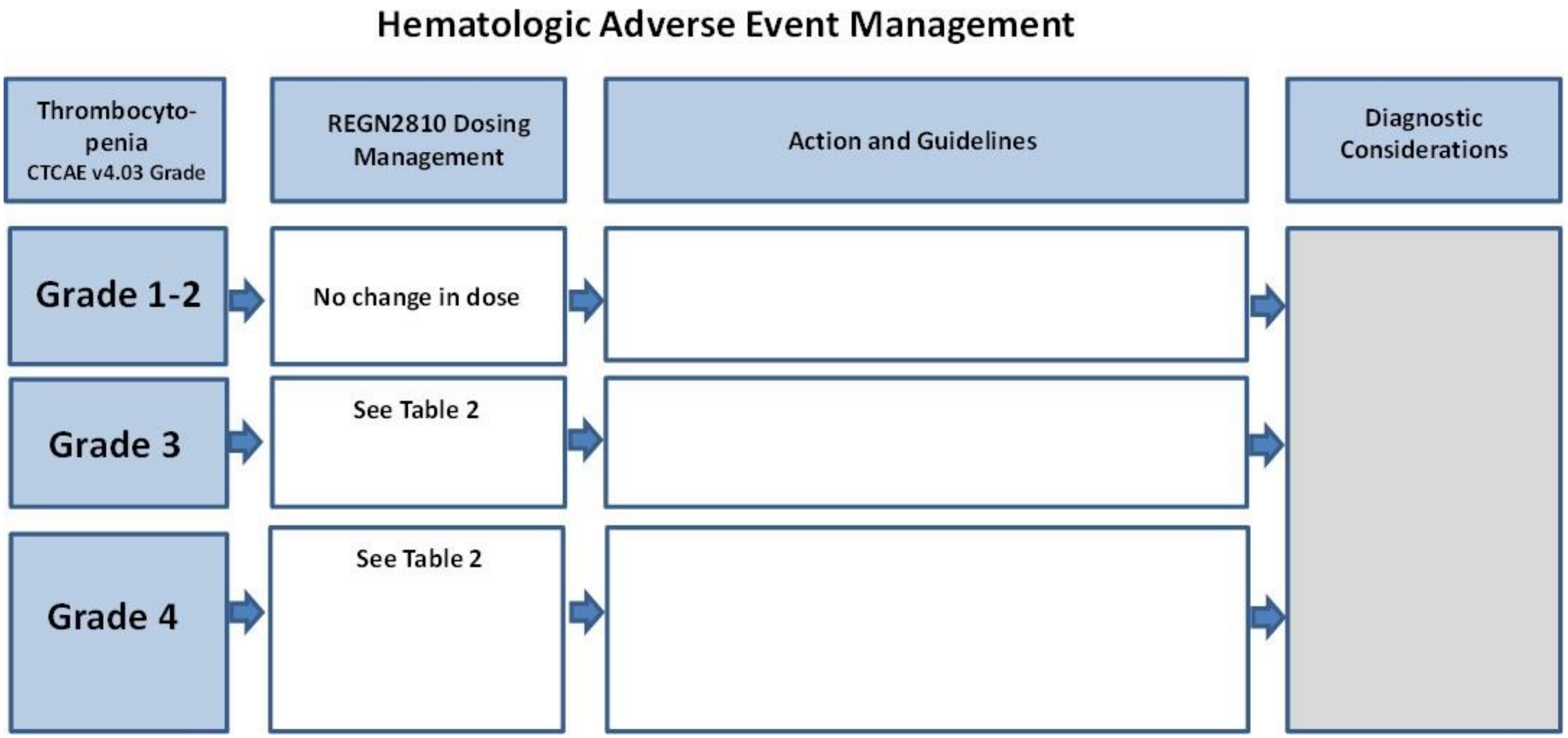

CONFIDENTIAL

VV-RIM-00023209-1.0 Approved - 29 Aug 2017 GMT-5:00

Hematologic Adverse Event Management (Cont)

Neutropenia CTCAE v4.03 Grade	REGN2810 Dosing Management	Action and Guidelines	Diagnostic Considerations
**Grade 1-3**	No change in dose	For neutropenia, see general guidelines on hematological toxicity in Table 2	
**Grade 4**	Hold until resolves to ≤Grade 1. May increase the dosing interval by 1 week. Discontinue if toxicities do not resolve within 12 weeks.	For neutropenia, see general guidelines on hematological toxicity in Table 2	

CONFIDENTIAL

VV-RIM-00023209-1.0 Approved - 29 Aug 2017 GMT-5:00

Dermatologic Adverse Event Management

SKIN EVENTS CTCAE v4.03 Grade	REGN2810 Dosing Management	Action and Guidelines	Diagnostic Considerations
**Grade 1-2**<br>Dermatitis exfoliative<br>Erythema multiforme<br>Stevens-Johnson syndrome<br>Toxic epidermal necrolysis	No change in dose	• Symptomatic treatment should be given such as topical glucocorticosteroids (eg, betamethasone 0.1% cream or hydrocortisone 1%) or urea-containing creams in combination with oral antipruritics (eg, diphenhydramine HCl or hydroxyzine HCl).<br>• Treatment with oral steroids is at investigator discretion for Grade 2 events.	All attempts should be made to rule out other causes such as metastatic disease, infection, or allergic dermatitis.
**Grade 3**<br>If considered to be immune related, ≥Grade 3 or result in dose modification or discontinuation:<br>Pruritus, Rash<br>Rash generalized<br>Rash maculo-papular<br>•	hold REGN2810	• Consider dermatology consultation and biopsy for confirmation of diagnosis.<br>• Treatment with oral steroids is recommended, starting with 1 mg/kg prednisone or equivalent once per day or dexamethasone 4 mg four times orally daily. When symptoms improve to Grade 1 or less, steroid taper should be started and continued over no less than 4 weeks.	
**Grade 4**	Permanently discontinue REGN2810	• Dermatology consultation and consideration of biopsy and clinical dermatology photograph.<br>• Initiate steroids at 1-2 mg/kg prednisone or equivalent. When symptoms improve to grade 1 or less, steroid taper should be started and continued over no less than 4 weeks.	

CONFIDENTIAL

VV-RIM-00023209-1.0 Approved - 29 Aug 2017 GMT-5:00

Hepatitis Adverse Event Management

Hepatitis CTCAE v4.03 Grade	REGN2810 Dosing Management	Action and Guidelines	Diagnostic Considerations
**Grade 1-2**<br>Hepatitis<br>Hepatitis, Autoimmune	Withhold REGN2810 if there is a treatment-emergent concurrent elevation of ALT and bilirubin that corresponds to an upward shift of 2 or more grades in both parameters.	• Monitor liver function tests more frequently until returned to baseline values.	All attempts should be made to rule out other causes such as metastatic disease, progressive liver disease, viral hepatitis, alternative drug toxicity, infectious causes and/or myositis.
**Grade 3-4**	Discontinue REGN2810 when AST or ALT >5.0 times ULN and/or total bilirubin >3.0 times ULN.	• Consider appropriate consultation and liver biopsy to establish etiology of hepatic injury, if necessary.<br>• Treat with high-dose IV glucocorticosteroids for 24–48 hours. When symptoms improve to grade 1 or less, a steroid taper with dexamethasone 4 mg every 4 hours or prednisone at 1–2 mg/kg should be started and continued over no less than 4 weeks.<br>• If serum transaminase levels do not decrease 48 hours after initiation of systemic steroids, oral mycophenolate mofetil 500 mg every 12 hours may be given. Infliximab is not recommended due to its potential for hepatotoxicity.<br>• Several courses of steroid tapering may be necessary as symptoms may worsen when the steroid dose is decreased	

Ophthalmologic (Uveitis) Adverse Event Management

Uveitis<br>CTCAE v4.03 Grade	REGN2810 Dosing Management	Action and Guidelines	Diagnostic Considerations
**Grade 1**<br>iritis, iridocyclitis	Discontinue REGN2810 if symptoms persist despite treatment with topical immunosuppressive therapy	• Evaluation by an ophthalmologist is strongly recommended.<br>• Treat with topical steroids such as 1% prednisolone acetate suspension and iridocyclitics.	All attempts should be made to rule out other causes such as metastatic disease, infection, or other ocular disease (*e.g.*, glaucoma or cataracts).
**Grade 2**	Discontinue REGN2810 if symptoms persist despite treatment with topical immunosuppressive therapy and do not improve to Grade 1 within the retreatment period OR requires systemic treatment.	• Evaluation by an ophthalmologist is strongly recommended.<br>• Treat with topical steroids such as 1% prednisolone acetate suspension and iridocyclitics.	
**Grade 3-4**	Discontinue REGN2810	• Treat with systemic corticosteroids such as prednisone at a dose of 1-2 mg/kg per day. When symptoms improve to ≤Grade 1, steroid taper should be started and continued over no less than 4 weeks	

Nausea and Vomiting Adverse Event Management

Nausea and Vomiting CTCAE v4.0 Grade	REGN2810 Dosing Management	Action and Guidelines	Diagnostic Considerations
**Grade 1**	No change in dose	• Nausea and vomiting should be treated aggressively, and consideration should be given in subsequent cycles to the administration of prophylactic antiemetic therapy according to standard institutional practice. Patients should be strongly encouraged to maintain liberal oral fluid intake.	
**Grade 2**	Hold until ≤Grade 1. Resume at same dose level. May increase dosing interval by 1 week if it takes more than 4 weeks for toxicities to resolve.		
**Grade 3**	Hold until <Grade 2. May increase dosing interval by 1 week for each occurrence. Discontinue if toxicities do not resolve within 12 weeks.		
**Grade 4**	Off protocol therapy		

VV-RIM-00023209-1.0 Approved - 29 Aug 2017 GMT-5:00

APPENDIX 4. REGN2810 PHARMACOKINETIC, IMMUNOGENICITY, AND BIOMARKER SAMPLING SCHEDULE

Study Visit	PK Sampling Time[a]	Anti-Drug Antibody Sampling Time[a]	Serum and Plasma Biomarker Samples Sampling Time[a]
Screening			• Collect on-study or archival (≤5 months old) tumor tissue sample
Cycle 1, day 1	• Pre-dose<br>• End of infusion	• Pre-dose	• Pre-dose<br>• Collect blood sample for DNA
Cycle 2, day 1	• Pre-infusion<br>• End of infusion		• Pre-infusion
Cycle 3, day 1	• Pre-infusion<br>• End of infusion	• Pre-infusion	
Cycle 4, day 1	• Pre-infusion<br>• End of infusion		• Pre-infusion
Cycle 5, day 1	• Pre-infusion<br>• End of infusion		
Cycle 6, day 1	• Pre-infusion<br>• End of infusion		
Cycle 9, day 1	• Pre-infusion<br>• End of infusion	• Pre-infusion	
Cycle 12, day 1	• Pre-infusion<br>• End of infusion		
Cycle 15, day 1	• Pre-infusion<br>• End of infusion	• Pre-infusion	
Cycle 18, day 1	• Pre-infusion<br>• End of infusion	• Pre-infusion	
Cycle 22, day 1	• Pre-infusion<br>• End of infusion	• Pre-infusion	
Cycle 26, day 1	• Pre-infusion<br>• End of infusion	• Pre-infusion	

Study Visit	PK Sampling Time[a]	Anti-Drug Antibody Sampling Time[a]	Serum and Plasma Biomarker Samples Sampling Time[a]
Cycle 30, day 1	• Pre-infusion<br>• End of infusion	• Pre-infusion	
Cycle 34, day 1	• Pre-infusion<br>• End of infusion	• Pre-infusion	
End of treatment	• Pre-infusion<br>• End of infusion	• Pre-infusion	
Follow-up visit 1	• Collect at visit	• Collect at visit	• Collect at visit
Follow-up visit 3	• Collect at visit	• Collect at visit	
At the time of RECIST 1.1-defined progressive disease			• Collect at visit[b]

Abbreviations: DNA=deoxyribonucleic acid; PK=pharmacokinetic; RECIST=Response Evaluation Criteria in Solid Tumors

[a] Pre-dose is defined as before the start of the first REGN2810 infusion. Pre-dose samples may be collected ≤72 hours prior to day 1 dosing. Pre-infusion is defined as before the start of subsequent REGN2810 infusions.

[b] A tumor biopsy should also be collected at this time point (optional).

Signature of Sponsor's Responsible Officers

(Scientific/Medical Monitor, Regulatory Representative, Clinical Study Team Lead, and Biostatistician)

To the best of my knowledge, this report accurately describes the conduct of the study and the data generated.

Study Title: A Randomized, Phase 3, Open-Label Study of Combinations of REGN2810 (Anti-PD-1 Antibody), Ipilimumab (Anti-CTLA-4 Antibody), and Platinum-Based Doublet Chemotherapy in First-Line Treatment of Patients With Advanced or Metastatic Non-Small Cell Lung Cancer With Tumors Expressing PD-L1 <50%

Protocol Number: R2810-ONC-16113

See appended electronic signature page

Sponsor's Responsible Scientific/Medical Monitor

See appended electronic signature page

Sponsor's Responsible Regulatory Representative

See appended electronic signature page

Sponsor's Responsible Clinical Study Team Lead

See appended electronic signature page

Sponsor's Responsible Biostatistician

Signature Page for VV-RIM-00023209 v1.0

Approval	[redacted]g-2017 19:23:20 GMT+0000
Approval	[redacted]-2017 19:46:08 GMT+0000
Approval	[redacted]17 20:41:45 GMT+0000
Approval	[redacted]000

Signature Page for VV-RIM-00023209 v1.0 Approved

IND Number: 134016
EudraCT Number: 2017-001311-36

Regeneron Pharmaceuticals, Inc.

Clinical Study Protocol

A TWO-PART RANDOMIZED, PHASE 3 STUDY OF COMBINATIONS OF CEMIPLIMAB (ANTI-PD-1 ANTIBODY) AND PLATINUM-BASED DOUBLET CHEMOTHERAPY IN FIRST-LINE TREATMENT OF PATIENTS WITH ADVANCED OR METASTATIC NON-SMALL CELL LUNG CANCER

**Compound:**	Cemiplimab (REGN2810 / anti-PD-1 mAb)
**Clinical Phase:**	3
**Protocol Number:**	R2810-ONC-16113
**Protocol Version:**	R2810-ONC-16113 Amendment 5
**Amendment 5 Date of Issue:**	*See appended electronic signature page*
**Amendment 4 Date of Issue:**	18 Jan 2019
**Amendment 3 JP Date of Issue:**	01 Nov 2018
**Amendment 3 Global Date of Issue:**	19 Jun 2018
**Amendment 2 VHP Date of Issue:**	15 Feb 2018
**Amendment 2 Date of Issue:**	25 Jan 2018
**Amendment 1 Date of Issue:**	06 Oct 2017
**Original Date of Issue:**	29 Aug 2017
**Scientific/Medical Monitor:**	[redacted]

Regeneron Pharmaceuticals, Inc.
777 Old Saw Mill River Road
Tarrytown, NY 10591

[redacted]

CLINICAL STUDY PROTOCOL SYNOPSIS

**Title**	A Two-Part Randomized, Phase 3 Study of Combinations of Cemiplimab (Anti-PD-1 Antibody) and Platinum-based Doublet Chemotherapy in First-line Treatment of Patients with Advanced or Metastatic Non-Small Cell Lung Cancer
**Site Locations**	Patients will be randomized at approximately 200 global study sites.
**Objectives**	<u>Primary Objectives</u><br>Part 1: To compare the overall survival (OS) of cemiplimab plus 4 cycles of platinum-based doublet chemotherapy (cemiplimab/chemo-f) and cemiplimab plust 2 cycles of platinum-based doublet chemotherapy (chemo-l) plus ipilimumab (cemiplimab/chemo-l/ipi) versus platinum-based doublet chemotherapy in the first-line treatment of patients with advanced squamous or non-squamous NSCLC with tumors expressing PD-L1 in <50% of tumor cells.<br>Part 2: To compare the OS of cemiplimab/chemo-f with placebo/chemo-f in the first-line treatment of patients with advanced squamous or non-squamous NSCLC irrespective of PD-L1 expression.<br>The key secondary objectives of the study are the following:<br>• Part 1: To compare the progression free survival (PFS) and overall response rate (ORR) of cemiplimab/chemo-f and cemiplimab/chemo-l/ipi versus chemo-f in the first-line treatment of patients with advanced squamous or non-squamous NSCLC and tumors expressing PD-L1 in <50% of tumor cells.<br>• Part 2: To compare the PFS and ORR of cemiplimab/chemo-f versus placebo/chemo-f in the first-line treatment of patients with advanced squamous or non-squamous NSCLC irrespective of PD-L1 expression.<br>The other secondary objectives are the following:<br>• Parts 1 and 2: To evaluate the safety and tolerability of cemiplimab/chemo-l/ipi and/or cemiplimab/chemo-f compared to platinum-based doublet chemotherapy or placebo/chemo-f<br>• Part 1 and 2: To evaluate the DOR of cemiplimab/chemo-l/ipi and/or cemiplimab/chemo-f compared to chemo-f or placebo/chemo-f in the first-line treatment of patients with advanced squamous or non-squamous NSCLC.<br>• Parts 1 and 2: To compare quality of life (QOL) in patients with advanced squamous or non-squamous NSCLC receiving cemiplimab/chemo-l/ipi and cemiplimab/chemo-f compared to platinum-based doublet chemotherapy or placebo/chemo-f<br>• Part 1: To evaluate the OS rate at 12 months, 18 months, and 24 months of cemiplimab/chemo-f and/or cemiplimab/chemo-l/ipi versus chemo-f in the

	first-line treatment of patients with advanced squamous or non-squamous NSCLC and tumors expressing PD-L1 in <50% of tumor cells.<br>• Part 2: To evaluate the OS rate at 12, 18, and 24 months of cemiplimab/chemo-f versus placebo/chemo-f in the first-line treatment of patients with advanced squamous or non-squamous NSCLC irrespective of PD-L1 expression.<br>• Parts 1 and 2: To assess immunogenicity as measured by anti-drug antibodies (ADAs) for cemiplimab<br>• Parts 1 and 2: To assess the predictive utility of baseline PD-L1 tumor expression levels on clinical response<br>• Part 1: To characterize the pharmacokinetics (PK) of cemiplimab when administered in combination with ipilimumab or in combination with platinum-based doublet chemotherapy.<br>• Part 2: To characterize the PK of cemiplimab when administered in combination with platinum-based doublet chemotherapy<br>• Parts 1 and 2: To conduct exposure-response (E-R) analyses for relevant biomarkers (exploratory PK/pharmacodynamic analyses) and E-R analyses for safety and efficacy endpoints, as appropriate<br>• Parts 1 and 2: Tumor mutation burden as assessed by the Foundation Medicine “FoundationOne®” panel, sample permitting
**Study Design**	This is a two-part, randomized, global, phase 3 study of cemiplimab/chemo-l/ipi and/or cemiplimab/chemo-f versus platinum-based doublet chemotherapy or placebo/chemo-f in the first-line treatment of patients with advanced squamous or non-squamous NSCLC.<br><br>Part 1 of the study will compare the efficacy and safety of cemiplimab/chemo-l/ipi and cemiplimab/chemo-f versus chemo-f in patients whose tumors express PD-L1 in <50% of tumor cells (measured using a PD-L1 IHC assay). Randomization in Part 1 will be 1:1:1 and prospectively stratified by histology (squamous versus non-squamous) and PD-L1 expression (<1% versus 1% to 24% versus 25% to 49% as measured using a PD-L1 immunohistochemistry [IHC] assay).<br><br>Part 2 of the study will compare the efficacy and safety of cemiplimab/chemo-f versus placebo/chemo-f in patients irrespective of PD-L1 expression. Randomization in Part 2 will be 2:1 and prospectively stratified by histology (squamous versus non- squamous) and PD-L1 expression (<1% versus 1% to 49% versus ≥50% as measured using a PD-L1 IHC assay). Patients with squamous NSCLC will be capped at 50% of the total sample size. At least 30% but no more than 40% of patients enrolled must have tumors that express PD-L1 in ≥50% of tumor cells. Enrollment of patients whose tumors express PD-L1 in <1% of tumor cells will be capped at 30%. (PD-L1 <50% is capped at 70%).

VV-RIM-00102568-2.0 Approved - 16 Apr 2020 GMT-5:00

	Part 1 consists of the following 3 periods: screening, treatment, and follow-up. After screening, eligible patients will be randomized to Treatment Arm A (platinum-based chemotherapy), Treatment Arm B (cemiplimab/chemo-f), or Treatment Arm C (cemiplimab/chemo-l/ipi). The length of the treatment period will be determined by the treatment arm to which a patient is randomized. Treatment may be discontinued early due to RECIST 1.1-defined progressive disease, withdrawal of consent, death, unacceptable toxicity, initiation of another anti-cancer treatment, or, for patients in Treatment Arms B and C, in specific instances of confirmed CR or PR. After discontinuing study treatment, patients will enter the follow-up period.<br><br>For Part 1 only, patients on Treatment Arm A who experience disease progression while on chemotherapy or after administration of chemotherapy will be offered the option to receive cemiplimab 350 mg Q3W for up to 108 weeks, provided they meet specific criteria. After the active portion of the study is complete, all patients will be followed for survival.<br><br>Part 2 consists of the following 3 periods: screening, treatment, and follow-up. After screening, eligible patients will be randomized to Treatment Arm A (placebo/chemo-f) or Treatment Arm B (cemiplimab/chemo-f). Treatment may be discontinued early due to RECIST 1.1-defined progressive disease, withdrawal of consent, death, unacceptable toxicity, or initiation of another anti-cancer treatment. After discontinuing study treatment, patients will enter the follow-up period. After the active portion of the study is complete, all patients will be followed for survival.
**Study Duration**	**Part 1**<br><br>The approximate duration of the active study assessments for each patient, excluding screening, will be 24 to 32 months. For Treatment Arms B and C, this encompasses 25 months of study treatment plus 7 months of follow-up. For patients in Treatment Arm A, this encompasses 4 cycles of study treatment (and pemetrexed maintenance, if appropriate) and radiographic tumor assessments for up to 2 years, if there is no disease progression; otherwise patients in Treatment Arm A advance to the 7 months follow-up period.<br><br>**Part 2**<br><br>The approximate duration of the active study assessments for each patient, excluding screening, will be 32 months. This encompasses 25 months of study treatment plus 7 months of follow-up. Patients will be unblinded to treatment at the time of progressive disease defined by RECIST1.1 criteria or discontinuation from study.

**Population**<br>**Sample Size:**	**Part 1**<br>Patients will be enrolled in Part 1 until Part 2 is activated at any given site. Part 1 halted patient randomization in August 2019 and randomized 323 by the end of August 2019.<br>**Part 2**<br>Approximately 450 patients will be randomized in Part 2 of the study. Enrollment of patients with squamous NSCLC histology will be capped at 50%.
**Target Population:**	Patients in this study will include men and women ≥18 years of age (≥20 years of age for Japanese patients), who are diagnosed with stage IIIB, IIIC, or stage IV non-squamous or squamous NSCLC.
**Treatments**<br>**Study Drug**<br>**Dose/Route/Schedule:**	Part 1: Cemiplimab administered at 350 mg as an intravenous (IV) infusion Q3W for 108 weeks in combination with platinum-based doublet chemotherapy Q3W administered IV for 4 cycles.
**Study Drug**<br>**Dose/Route/Schedule:**	Part 1: Cemiplimab administered at 350 mg as an IV infusion Q3W for 108 weeks in combination with platinum-based doublet chemotherapy Q3W administered IV for 2 cycles and ipilimumab administered IV over approximately 90 minutes at 50 mg Q6W for up to 4 doses.
**Reference Drug**<br>**Dose/Route/Schedule:**	Part 1: Platinum-based doublet chemotherapy administered IV Q3W for 4 cycles (followed by pemetrexed maintenance for those patients initially assigned to receive a pemetrexed-containing regimen).
**Reference Drug**<br>**Dose/Route/Schedule:**	Part 2: Placebo Q3W for 108 weeks as an IV infusion, and platinum-based doublet chemotherapy Q3W for 4 cycles as an IV infusion (followed by pemetrexed maintenance for those patients initially assigned to receive a pemetrexed-containing regimen) based on randomization.
**Study Drug**<br>**Dose/Route/Schedule:**	Part 2: Cemiplimab 350 mg Q3W for 108 weeks as an IV infusion, and platinum-based doublet chemotherapy Q3W for 4 cycles as an IV infusion (followed by pemetrexed maintenance for those patients initially assigned to receive a pemetrexed-containing regimen) based on randomization.

VV-RIM-00102568-2.0 Approved - 16 Apr 2020 GMT-5:00

**Endpoints**	Primary Endpoint<br>The primary endpoint for Part 1 and Part 2 of the study is OS.<br>Key Secondary Endpoints (Part 1 and Part 2)<br>For Part 1, the key secondary endpoints are PFS and ORR as assessed by IRC and investigator per RECIST 1.1. Progression free survival and ORR per IRC assessment will be performed hierarchically when analysis of OS is statistically significant.<br>For Part 2, the key secondary endpoints are PFS and ORR as assessed by IRC per RECIST 1.1.<br>Other Secondary or Exploratory Endpoints<br>Other secondary or exploratory endpoints will include the following:<br>DOR<br>BOR, as determined by the IRC or investigator per RECIST 1.1,<br>The safety and tolerability of study regimens measured by the incidence of TEAEs, dose-limiting toxicities (DLTs), serious adverse events (SAEs), deaths, and laboratory abnormalities<br>Overall survival rate at 12 months, 18 months, and 24 months.<br>Quality of life as measured by the European Organization for Research and Treatment of Cancer Quality of Life Questionnaire Core 30 (EORTC QLQ-C30) and Quality of Life Questionnaire Lung Cancer 13 (EORTC QLQ-LC13).

CONFIDENTIAL

VV-RIM-00102568-2.0 Approved - 16 Apr 2020 GMT-5:00

**Procedures and Assessments**	Procedures to be performed at screening will include informed consent; assessment of inclusion/exclusion criteria; recording of medical, oncology, and concomitant medications histories; recording of demographics; collection and testing of tumor tissue samples for PD-L1 assessment and for epidermal growth factor receptor and anaplastic lymphoma kinase mutations and C-ros oncogene receptor tyrosine kinase fusions; radiographic tumor assessment; tumor burden assessment; chest X-ray; serum pregnancy testing; 12-lead electrocardiogram; adverse event (AE) recording; physical examination, including vital signs, height, and weight assessments; Eastern Cooperative Oncology Group (ECOG) performance status assessment; and laboratory testing. Samples for an optional genomic sub-study may also be obtained.<br><br>During the treatment period, the following procedures will be performed to assess efficacy and safety: QOL measurement using validated patient questionnaires, physical examination, ECOG performance status assessment; vital signs; laboratory testing, including pregnancy testing for women of childbearing potential; recording of AEs and concomitant medications. Computed tomography or magnetic resonance imaging (or positron emission tomography) for radiographic tumor burden assessment and tumor burden assessment based on RECIST 1.1 criteria will be performed at pre-specified time points throughout the study.<br><br>Other assessments will include cemiplimab concentration measurement, cemiplimab ADA assessment, and biomarker assessments. Biomarker procedures will include the use of tumor tissue samples for validation of additional PD-L1 assays.<br><br>Survival data and treatment status will then be collected by phone, at office visit every 3 months, or at the time of pre-planned interim and final analyses, until the required number of events for the primary endpoint of the study has been reached.
**Statistical Plan**	The primary statistical hypotheses for Part 1 are that:<br><br>1) cemiplimab/chemo-f (Arm B) will prolong OS as compared with chemo-f (Arm A)<br><br>2) cemiplimab/chemo-l/ipi (Arm C) will prolong OS as compared with chemo-f (Arm A)<br><br>The primary statistical hypothesis for Part 2 is that:<br><br>1) cemiplimab/chemo-f will prolong OS as compared with platinum-based doublet chemotherapy<br><br><u>Justification of Sample Size</u><br><br><u>Part 1:</u><br><br>Enrollment in Part 1 at any individual site was stopped in August 2019 when the amended study authorizing Part 2 of the study received IRB/EC approval and all other Health Authority requirements were met. At that time, enrollment in Part 2 began. By the end of August 2019, Part 1 randomized a total of 323 patients with approximately 215 patients in each of the cemiplimab combination versus

	chemotherapy comparisons (cemiplimab/chemo-f vs. chemo-f [B + A ≈ 215] or cemiplimab/chemo-l/ipi vs. chemo-f [C + A ≈ 215]).<br><br>The sponsor assumes a median OS of 12 months for patients treated with chemotherapy , and a hazard ratio of 0.65 in OS for each cemiplimab combination versus the chemotherapy comparison. Patients will be randomized in a 1:1:1 ratio to the chemo-f arm (Arm A), the cemiplimab/chemo-f arm (Arm B) versus cemiplimab/chemo-l/ipi (Arm C). Under these assumptions and for each cemiplimab combination comparison, 125 deaths at interim analysis for OS and 151 deaths (cumulative 70% out of 215 randomized in each of the comparisons) at final analysis for OS are needed to yield approximately 74% power to detect statistical significance in OS by stratified Log-Rank testing procedure with an overall 2-sided type one error of 0.0499, given 2-sided type error of 0.0001 will be spent on the first administrative analysis of PFS and ORR per investigator assessment.<br><br>The sponsor assumes a median PFS of 6 months for patients treated with chemotherapy alone, and a hazard ratio of 0.6667 in PFS for each cemiplimab combination treatment arm versus the chemotherapy comparison. Under these assumptions and for each cemiplimab combination versus chemotherapy comparison, 161 PFS events (75% out of 215 randomized patients in each of the cemiplimab combination comparisons) are needed to yield approximately 73% power to detect statistical significance in PFS by stratified Log-Rank testing procedure with an overall 2-sided type one error of 0.0499.<br><br><u>Part 2:</u><br><br>Historically, in patients with stage IIIB or stage IV NSCLC treated with cisplatin or carboplatin plus paclitaxel Q3W, the median PFS has ranged from approximately 2.7 to 6.4 months .<br><br>The sponsor assumes a median OS of 12 months for patients treated with chemotherapy plus placebo, and a hazard ratio of 0.65 in OS between cemiplimab/chemo-f arm and placebo/chemo-f arm. Patients will be randomized in a 2:1 ratio to the cemiplimab/chemo-f arm (Treatment Arm B) versus the placebo/chemo-f arm (Treatment Arm A). Under these assumptions, 291 deaths are needed to yield approximately 93% power to detect statistical significance in OS at 2-sided 0.05 level between two treatment arms.<br><br>Considering an enrollment period of 14 months (11 patients per month for the first 4 months, 26 patients per month for month 5 to 8, 50 patients per month afterwards), an approximately 24-month follow-up period for OS after completion of enrollment, and 10% annual dropout, the enrollment of approximately 450 randomized patients is needed to obtain 291 deaths for the final analysis of OS.<br><br>The sponsor assumes a median PFS of 6 months for patients treated with chemotherapy plus placebo, and a hazard ratio of 0.6667 in PFS between cemiplimab/chemo-f arm and placebo/chemo-f arm . With these assumptions and

	at two-sided 0.05 level, the power for analysis of PFS will be 90% or more if it is performed after 288 or more PFS events are observed.<br><br>**Interim Analyses**<br><br><u>Part 1:</u><br><br>An interim analysis of OS will be performed when 125 deaths (83% of total OS events) have been observed in the comparison of cemiplimab/chemo-f vs. chemo-f.<br><br>The final analysis of OS will be performed when 151 deaths (cumulatively 70% out of 215 randomized patients) have been observed.<br><br>The Lan-DeMets O'Brien-Fleming alpha spending for analysis of OS is specified in the protocol.<br><br>Final analysis of PFS will be performed at 2-sided 0.0499 level when analysis of OS is statistically significant either at the interim analysis or at final analysis for OS. At the time of PFS analysis, it is estimated more than 161 PFS events have been observed in each of the cemiplimab comparison.<br><br><u>Part 2:</u><br><br>Two interim analyses of OS will be performed when 146 deaths (50% of total OS events) and 204 deaths (70% of total OS events) have been observed.<br><br>The final analysis of OS will be performed 291 deaths (cumulative) have been observed.<br><br>The alpha spending for analysis of OS is specified in the protocol.<br><br>Final analysis of PFS will be performed at 2-sided 0.05 level when analysis of OS is statistically significant either at interim analysis or at final analysis for OS.<br><br>Analysis of ORR will be performed when PFS is statistically significant.

CONFIDENTIAL

VV-RIM-00102568-2.0 Approved - 16 Apr 2020 GMT-5:00

LIST OF ABBREVIATIONS AND DEFINITIONS OF TERMS

ACTH	Adrenocorticotropic hormone
ADA(s)	Anti-drug antibody(ies)
ADL	Activities of daily living
AE	Adverse event
AESI	Adverse event of special interest
ALK	Anaplastic lymphoma kinase
ALT	Alanine aminotransferase
Anti-CTLA-4	Anti-cytotoxic T-lymphocyte-associated antigen 4
Anti-PD-1	Anti-programmed death-1
Anti-PD-L1	Anti-programmed death ligand 1
AST	Aspartate aminotransferase
ATC	Anatomical therapeutic chemical
AUC	Area under the curve
BOR	Best overall response
BUN	Blood urea nitrogen
C2P1	Cell Line 2 Process 1
CBC	Complete blood count
CI	Confidence interval
C_{eoi}	Concentration at end of infusion
Chemo-f	Chemo-full; 4 cycles of chemotherapy
Chemo-l	Chemo-limited; 2 cycles of chemotherapy
CHO	Chinese hamster ovary
CNS	Central nervous system
CR	Complete response
CRF	Case report form
CRO	Contract research organization
CSCC	Cutaneous squamous cell carcinoma
CT	Computed tomography
CTLA-4	Cytotoxic T-lymphocyte-associated protein 4
C_{trough}	Pre-infusion concentration
CTX	Cyclophosphamide
CV%	Variability in exposure
DLT	Dose-limiting toxicity

DNA	Deoxyribonucleic acid
DOR	Duration of response
EC	Ethics Committee
ECG	Electrocardiogram
ECOG	Eastern Cooperative Oncology Group
EDC	Electronic data capture
EGFR	Epidermal growth factor receptor
EORTC QLQ-C30	European Organization for Research and Treatment of Cancer Quality of Life Questionnaire Core 30
EORTC QLQ-LC13	European Organization for Research and Treatment of Cancer Quality of Life Questionnaire Lung Cancer 13
E-R	Exposure-response
EU	European Union
FAS	Full analysis set
FDA	Food and Drug Administration
FIH	First-in-human
GCP	Good Clinical Practice
GFR	Glomerular filtration rate
HBV	Hepatitis B virus
HCV	Hepatitis C virus
HIV	Human immunodeficiency virus
HR	Hazard ratio
ICF	Informed consent form
ICH	International Council for Harmonisation
IDMC	Independent Data Monitoring Committee
IHC	Immunohistochemistry
ipi	Ipilimumab
irAE	Immune-related adverse event
IRB	Institutional Review Board
IRC	Independent Review Committee
irTEAE	Immune-related treatment-emergent adverse event
IV	Intravenous
IVRS	Interactive voice response system
IWRS	Interactive web response system
JP	Japan

LDH	Lactate dehydrogenase
MedDRA	Medical Dictionary for Regulatory Activities
MRI	Magnetic resonance imaging
MTD	Maximum tolerated dose
N/A	Not applicable
NAb	Neutralizing anti-drug antibody
NCI-CTCAE	National Cancer Institute: Common Terminology Criteria for Adverse Events
NE	Not evaluable
NR	Not reported
NSCLC	Non-small cell lung cancer
ORR	Objective response rate
OS	Overall survival
PD	Progressive disease
PD-1	Programmed cell death-1
PD-L1	Programmed cell death ligand 1
PD-L2	Programmed cell death ligand 2
PE	Physical examination
PET	Positron emission tomography
PFS	Progression-free survival
PK	Pharmacokinetic(s)
PMDA	Pharmaceuticals and Medical Devices Agency
PPD	Purified protein derivative
PR	Partial response
PT	Preferred Term
PT/PTT/INR	Prothrombin time/Partial thromboplastin time/International normalized ratio
QOL	Quality of life
Q2W	Every 2 weeks
Q3W	Every 3 weeks
Q6W	Every 6 weeks
Q9W	Every 9 weeks
Q12W	Every 12 weeks
Q18W	Every 18 weeks
Q24W	Every 24 weeks
RBC	Red blood cell

RECIST 1.1	Response Evaluation Criteria in Solid Tumors version 1.1
Regeneron	Regeneron Pharmaceuticals, Inc.
RNA	Ribonucleic acid
ROS1	C-ros oncogene receptor tyrosine kinase
SAE	Serious adverse event
SAF	Safety analysis set
SAP	Statistical analysis plan
SD	Stable disease
SmPC	Summary of Product Characteristics
SOC	System organ class
T4	Thyroxine
TEAE	Treatment-emergent adverse event
t_{eoi}	Time of end of infusion
TSH	Thyroid-stimulating hormone
ULN	Upper limit of normal
US	United States
WBC	White blood cell

TABLE OF CONTENTS

AMENDMENT HISTORY 2
CLINICAL STUDY PROTOCOL SYNOPSIS 20
LIST OF ABBREVIATIONS AND DEFINITIONS OF TERMS 28
1. INTRODUCTION 39
2. STUDY OBJECTIVES 47
2.1. Primary Objectives 47
2.2. Secondary Objectives 47
2.2.1. Key Secondary Objectives 47
2.2.2. Other Secondary Objectives 48
3. HYPOTHESIS AND RATIONALE 49
3.1. Hypothesis 49
3.2. Rationale 49
3.2.1. Rationale for Study Design 49
3.2.2. Rationale for Endpoints and Objectives 49
3.2.3. Rationale for Cemiplimab Dose Selection 50
3.2.4. Rationale for Ipilimumab Dose Selection (Part 1 Only) 51
3.2.5. Rationale for Platinum-Based Chemotherapy as Comparator 51
4. STUDY VARIABLES 52
4.1. Demographic and Baseline Characteristics 52
4.2. Primary and Secondary Endpoints 52
4.2.1. Primary Endpoint (Part 1 and Part 2) 52
4.2.2. Key Secondary Endpoints (Part 1 and Part 2) 52
4.2.3. Other Secondary or Exploratory Endpoints 53
4.3. Pharmacokinetic Variables 53
4.4. Anti-Drug Antibody Variables 53
5. STUDY DESIGN 54
5.1. Study Description and Duration 54
5.1.1. Screening 57
5.1.2. Treatment Period 58
5.1.3. Follow-Up Period 61
5.1.4. Description of Study Stopping Rules (Part 1) 63

CONFIDENTIAL

VV-RIM-00102568-2.0 Approved - 16 Apr 2020 GMT-5:00

5.1.5. Dose-Limiting Toxicities (Part 1) 63
5.1.6. Maximum Tolerated Dose (Part 1 Only) 65
5.1.7. End of Study Definition 65
5.2. Planned Interim Analysis 65
5.3. Study Committees 65
5.3.1. Independent Review Committee 65
5.3.2. Independent Data Monitoring Committee 66
6. SELECTION, WITHDRAWAL, AND REPLACEMENT OF PATIENTS 66
6.1. Number of Patients Planned 66
6.2. Study Population 66
6.2.1. Inclusion Criteria 67
6.2.2. Exclusion Criteria 68
6.3. Premature Withdrawal from the Study 70
6.4. Replacement of Patients 71
7. STUDY TREATMENTS 71
7.1. Investigational and Reference Treatments 71
7.1.1. Cemiplimab or Placebo 71
7.1.2. Ipilimumab (Part 1 Only) 72
7.1.3. Platinum-based Doublet Chemotherapy 72
7.2. Pre-treatments 74
7.3. Dose Modification and Study Treatment Discontinuation Rules 74
7.3.1. Cemiplimab Plus Ipilimumab and Chemotherapy Combination Therapy (Part 1 Only) 74
7.3.2. Cemiplimab or Placebo as Combination Therapy with Chemotherapy 77
7.3.3. Cemiplimab Monotherapy 79
7.3.4. Immune-Related Adverse Events 79
7.3.5. Ipilimumab (Part 1 Only) 81
7.3.6. Platinum-Based Doublet Chemotherapy 81
7.3.7. Permanent Study Drug Discontinuation 82
7.4. Management of Acute Reactions 82
7.4.1. Acute Infusion Reactions 82
7.4.1.1. Interruption of the Infusion 83

7.4.1.2. Termination of the Infusion 84
7.5. Method of Treatment Assignment 84
7.5.1. Blinding 85
7.5.2. Emergency Unblinding (Part 2 Only) 85
7.6. Treatment Logistics and Accountability 86
7.6.1. Packaging, Labeling, and Storage 86
7.6.2. Supply and Disposition of Treatments 87
7.6.3. Treatment Accountability 87
7.6.4. Treatment Compliance 88
7.7. Concomitant Medications 88
7.7.1. Prohibited Medications and Procedures 88
7.7.2. Permitted Medications and Procedures 88
7.8. Disease Progression While Receiving Cemiplimab 89
7.9. Disease Progression While Receiving Chemotherapy Plus Placebo (Part 2) 89
7.10. Optional Cemiplimab Monotherapy (Part 1 Only) 89
7.11. Post-Study Treatments and Procedures 90
8. STUDY SCHEDULE OF EVENTS AND PROCEDURES 90
8.1. Schedule of Events 90
8.1.1. Footnotes for the Schedule of Events Table 110
8.1.2. Early Termination Visit 110
8.1.3. Unscheduled Visits 110
8.2. Study Procedures 110
8.2.1. Procedures Performed Only at the Screening/Baseline Visit 110
8.2.2. Efficacy Procedures 111
8.2.2.1. Radiographic Tumor Assessments 111
8.2.2.2. Tumor Burden Assessments 112
8.2.2.3. Quality of Life Questionnaires 113
8.2.2.4. Survival Data and Treatment Status Collection 113
8.2.3. Safety Procedures 113
8.2.3.1. Physical Examination 113
8.2.3.2. ECOG Performance Status 113
8.2.3.3. Weight and Height 113

8.2.3.4. Vital Signs 114
8.2.3.5. 12-Lead Electrocardiogram 114
8.2.3.6. Laboratory Testing....114
8.2.3.7. Concomitant Medication Recording....116
8.2.3.8. Adverse Event Recording....116
8.2.4. Pharmacokinetic and Anti-Drug Antibody Procedures 116
8.2.4.1. Drug Concentration Measurements and Samples....116
8.2.4.2. Anti-Drug Antibody Measurements and Samples....117
8.2.5. Biomarker Procedures 117
8.2.6. Future Biomedical Research....118
8.2.6.1. Genomics Sub-Study - Optional....118
9. SAFETY DEFINITIONS, REPORTING, AND MONITORING 118
9.1. Obligations of Investigator 118
9.2. Obligations of Sponsor 118
9.3. Definitions 119
9.3.1. Adverse Event....119
9.3.2. Serious Adverse Event....119
9.3.3. Adverse Events of Special Interest 120
9.3.3.1. Immune-Related Adverse Events 120
9.3.4. Infusion Reactions 121
9.4. Recording and Reporting Adverse Events....121
9.4.1. Adverse Events 121
9.4.2. Serious Adverse Events 121
9.4.3. Other Events that Require Accelerated Reporting to Sponsor 122
9.4.4. Reporting Adverse Events Leading to Withdrawal from the Study....122
9.4.5. Abnormal Laboratory, Vital Signs, or Electrocardiogram Results....122
9.4.6. Follow-up....122
9.5. Evaluation of Severity and Causality 123
9.5.1. Evaluation of Severity 123
9.5.2. Evaluation of Causality....123
9.6. Safety Monitoring....124
9.7. Investigator Alert Notification....124

10. STATISTICAL PLAN....124
10.1. Statistical Hypothesis....124
10.2. Justification of Sample Size....125
10.3. Analysis Sets....126
10.3.1. Efficacy Analysis Sets....126
10.3.2. Safety Analysis Set....126
10.3.3. Other Analysis Sets....126
10.4. Statistical Methods....126
10.4.1. Patient Disposition....127
10.4.2. Demography and Baseline Characteristics....127
10.4.3. Medical History....127
10.4.4. Prior Medications/Concomitant Medications....127
10.4.5. Efficacy Analyses....128
10.4.6. Safety Analyses....129
10.4.6.1. Adverse Events....130
10.4.6.2. Other Safety Analyses....130
10.4.6.3. Treatment Exposure....131
10.4.6.4. Treatment Compliance....131
10.4.7. Analysis of Drug Concentration Data....131
10.4.8. Analysis of Anti-Drug Antibody Data....131
10.4.9. Analysis of Biomarker Data....131
10.5. Multiplicity Considerations....131
10.6. Interim Analyses....133
10.7. Additional Statistical Data Handling Conventions....134
10.8. Statistical Considerations Surrounding the Premature Termination of a Study....135
11. DATA MANAGEMENT AND ELECTRONIC SYSTEMS....135
11.1. Data Management....135
11.2. Electronic Systems....135
12. STUDY MONITORING....136
12.1. Monitoring of Study Sites....136
12.2. Source Document Requirements....136
12.3. Case Report Form Requirements....136

CONFIDENTIAL

VV-RIM-00102568-2.0 Approved - 16 Apr 2020 GMT-5:00

13. AUDITS AND INSPECTIONS 136
14. ETHICAL AND REGULATORY CONSIDERATIONS 137
14.1. Good Clinical Practice Statement 137
14.2. Informed Consent 137
14.3. Patients' Confidentiality and Data Protection 138
14.4. Institutional Review Board/Ethics Committee 138
15. PROTOCOL AMENDMENTS 138
16. PREMATURE TERMINATION OF THE STUDY OR CLOSE-OUT OF A SITE 139
16.1. Premature Termination of the Study 139
16.2. Close-out of a Site 139
17. STUDY DOCUMENTATION 139
17.1. Certification of Accuracy of Data 139
17.2. Retention of Records 139
18. CONFIDENTIALITY 140
19. FINANCING AND INSURANCE 140
20. PUBLICATION POLICY 140
21. REFERENCES 140
22. INVESTIGATOR'S AGREEMENT 145
SIGNATURE OF SPONSOR'S RESPONSIBLE OFFICERS 169

LIST OF TABLES

Table 1: Guidelines for Platinum-Based Doublet Chemotherapy Regimens 73
Table 2: Cemiplimab or Placebo Guidelines for Temporary and Permanent Discontinuations for Toxicity 78
Table 3: General Cemiplimab Treatment Hold Guidelines for Immune-Related Adverse Events 81
Table 4: Schedule of Events: Screening Visit Assessments and Procedures 91
Table 5: Schedule of Events: Part 1 Treatment Period Assessments and Procedures 93
Table 6: Schedule of Events: Part 2 Treatment Period Assessments and Procedures 101
Table 7: Schedule of Events: Follow-Up Period Assessments and Procedures (Part 1 and Part 2) 107
Table 8: Summary of Decision Guidance for Part 1 Analysis 132

Table 9: Alpha Spending for Analysis of OS 133
Table 10: Alpha Spending for Analysis of OS 134
Table 11: Response According to Revised Response Evaluation Criteria in Solid Tumors (Version 1.1) in Patients with Target (and Non-Target) Lesions 152

LIST OF FIGURES

Figure 1: Study Flow Diagram – Part 1 55
Figure 2: Study Flow Diagram – Part 2 56

LIST OF APPENDICES

APPENDIX 1. FACTORS TO CONSIDER IN ASSESSING THE RELATIONSHIP OF ADVERSE EVENTS TO STUDY DRUG AND STUDY CONDUCT 146
APPENDIX 2. RESPONSE EVALUATION CRITERIA IN SOLID TUMORS: RECIST GUIDELINE (VERSION 1.1) 147
APPENDIX 3. RECOMMENDED DOSE MODIFICATION OR DISCONTINUATION AND SUPPORTIVE CARE GUIDELINES FOR SPECIFIC CEMIPLIMAB DRUG-RELATEDADVERSE EVENTS (SEE SECTION 7.5.2 FOR EMERGENCY UNBLINDING) 154
APPENDIX 4. CEMIPLIMAB OR PLACEBO PHARMACOKINETIC, IMMUNOGENICITY, AND BIOMARKER SAMPLING SCHEDULE 168

VV-RIM-00102568-2.0 Approved - 16 Apr 2020 GMT-5:00

1. INTRODUCTION

The original protocol design was a 3-arm study for first-line treatment of patients with non-small cell lung cancer (NSCLC) with a PD-L1+ score in their tumors of <50%, and with a primary endpoint of progression-free survival (PFS). Treatment Arm A was standard histology specific platinum-based chemotherapy (up to four cycles, or chemo-full [chemo-f]), Treatment Arm B was chemo-full plus cemiplimab, and Treatment Arm C was an abbreviated course of histology-specific platinum-based chemotherapy (2 cycles, or chemo-limited [chemo-l]) plus cemiplimab plus low dose ipilimumab (ipi). At Amendment 4, the protocol was updated so that patients enrolled per the original criteria were designated as enrolled in Part 1.

Part 2 of the study, which was introduced in Amendment 4, will enroll patients regardless of the level of PD-L1 staining in their tumors; patients will be randomized to chemo-f plus cemiplimab or chemo-f plus saline/dextrose placebo (placebo). In Amendment 4, Part 2 had co-primary endpoints of overall survival (OS) and PFS. In Amendment 5, the endpoints were updated to OS as primary endpoint and PFS as a key secondary endpoint. Further details of the changes in Part 2 are described below. The main reasons for instituting the changes in Amendment 4 are:

1. The desire to demonstrate the benefit of the addition of cemiplimab to chemotherapy regardless of the level of PD-L1 staining.
2. The desire to include a survival co-primary endpoint so that this study could support an application for registration.
3. The increasing recognition that the addition of CTLA-4 blockade to PD-1/PD-L1 blockade has not robustly improved benefit, and the desire to explore this combination with an additional limited course of chemotherapy in a more limited way.

In Amendment 5, the following interim analyses are introduced in the study in order to assess OS at earlier timepoints in Parts 1 and 2. In Part 1, an interim analysis at ~83% OS event is introduced. In Part 2, an interim analysis at 50% OS event is introduced in addition to the existing interim analysis at 70% OS event.

OS has been elevated to be the primary endpoint in Part 1 to potentially support a registration application based on the enrolled patients in Part 1 of the study. OS is considered the primary endpoint of choice for regulatory approval. PFS and objective response rate (ORR) will be assessed as key secondary endpoints.

Background on Lung Cancer

Lung cancer is one of the most commonly diagnosed cancers and is the leading cause of cancer-related mortality worldwide (Siegel 2016, Bray 2013). Non-small cell lung cancer accounts for 80% to 85% of all lung cancers and is composed of several histopathological subtypes, the most common of which are adenocarcinoma (40% to 60%) and squamous cell carcinoma (30%). In the past several decades, the incidence of adenocarcinoma has increased greatly, and adenocarcinoma has replaced squamous cell carcinoma as the most prevalent type of NSCLC (Dela Cruz, 2011). The majority of patients with NSCLC are often found to have advanced cancer at the time of diagnosis

Systemic therapy with platinum-based doublet chemotherapy regimens, with or without maintenance therapy, was until recently the standard of care for first-line treatment of patients

with advanced NSCLC whose tumors do not have an epidermal growth factor receptor (EGFR) mutation, an anaplastic lymphoma kinase (ALK)mutation, or a C-ros oncogene receptor tyrosine kinase (ROS1) fusion (Besse 2014, Ettinger 2016, Reck 2014). Despite chemotherapy, patients with metastatic NSCLC had a median overall survival (OS) of approximately 8 to 12 months, and a 5 year survival rate of approximately 18% (Leighl 2012, Siegel 2016). With the introduction of immunotherapy to NSCLC, this grim prognosis has recently improved.

It is recognized that a complex cross-talk between cancer cells and the host immune system exists that can both inhibit and enhance tumor growth (Vinay 2015). Tumors modulate and evade the host immune response through a number of mechanisms, including formation of an immune-suppressive environment within the tumor. Programmed cell death-1 (PD-1) is an important co-receptor expressed on the surface of activated T-cells that mediates immunosuppression. The binding of PD-1 to one of its ligands, programmed cell death ligand 1 (PD-L1) or programmed cell death ligand 2 (PD-L2), results in inhibition of a cytotoxic T-cell response. Increased expression of PD-L1 in the tumor microenvironment facilitates escape from the immune-surveillance mechanism (T-cell-induced anti-tumor activity). In contrast, blockade of this interaction results in an enhanced T-cell response with anti-tumor activity.

Rationale for Cemiplimab Monotherapy for the Treatment of NSCLC

In NSCLC, there is a wide range of PD-L1 expression levels in tumor cells (D'Incecco 2015, Kerr 2015), and a high level of expression has been correlated with poor patient prognosis and resistance to treatment (Creelan 2014). Blockade of the PD-1/PD-L1 T-cell checkpoint pathway has been shown to be an effective and well-tolerated approach to stimulating the immune response and has achieved significant objective responses in patients with NSCLC (Topalian 2012).

Initial studies demonstrated the efficacy of PD-1 inhibitors as monotherapy in second line treatment of NSCLC, generating great enthusiasm for these new agents and leading to approvals for both nivoljumab (OPDIVO®) and pembrolizumab (KEYTRUDA®). Programmed cell death-1/PD-L1 inhibitors have subsequently been evaluated in the first-line treatment of NSCLC. The phase 3 CheckMate 026 trial investigated the efficacy of first-line treatment with nivolumab as monotherapy compared to platinum-based doublet chemotherapy in patients with advanced NSCLC and PD-L1 positive tumors (defined as present in 1% or more tumor cells). A total of 541 patients were randomized 1:1 to nivolumab or chemotherapy. In 423 patients with 5% or greater PD-L1 expression, progression-free survival (PFS) was 4.2 months with nivolumab and 5.9 months with chemotherapy (hazard ration [HR]: 1.15 95%CI: 0.91 to 1.45, p=0.25). OS was 14.4 months for nivolumab versus 13.2 months for chemotherapy (HR: 1.02; 95% CI: 0.80 to 1.30). Among all treated patients, any serious treatment-related AEs were 71% and 18% with nivolumab, and 92% and 51% with chemotherapy, respectively (Socinski 2016).

In contrast to this negative trial, pembrolizumab was investigated as monotherapy in KEYNOTE-024, an open-label, phase 3 trial with 305 patients with previously untreated advanced NSCLC with PD-L1 expression on at least 50% of tumors. Patients were randomized to receive either pembrolizumab (at a fixed dose of 200 mg every 3 weeks [Q3W]) or the investigator's choice of platinum-based chemotherapy. Crossover from the chemotherapy group to the pembrolizumab group was permitted in the event of disease progression. Median PFS was

VV-RIM-00102568-2.0 Approved - 16 Apr 2020 GMT-5:00

10.3 months (95% confidence interval [CI]: 6.7 months to not reached) in the pembrolizumab group versus 6.0 months (95% CI: 4.2 to 6.2 months) in the chemotherapy group (hazard ratio [HR] for disease progression or death: 0.50; 95% CI: 0.37 to 0.68; p=0.0015. The estimated rate of OS at 6 months was 80.2% in the pembrolizumab group versus 72.4% in the chemotherapy group (hazard ratio for death, 0.60; 95% CI, 0.41 to 0.89; p=0.005). The response rate was higher in the pembrolizumab group than in the chemotherapy group (44.8% versus 27.8%), the median duration of response (DOR) was longer (not reached [range: 1.9+ to 14.5+ months] vs 6.3 months [range: 2.1+ to 12.6+]), and treatment-related AEs of any grade were less frequent (occurring in 73.4% vs 90.0% of patients), as were grade 3, 4, or 5 treatment-related AEs (26.6% vs 53.3%) (Reck 2016). In October 2016, the United States (US) Food and Drug Administration (FDA) approved pembrolizumab (KEYTRUDA®, Merck & Co., Inc.) for the treatment of patients with metastatic NSCLC, whose tumors express PD-L1 ≥50% as determined by the PD-L1 IHC 22C3 pharmDx 22C3 assay (Dako, North America, Inc.). This was the first FDA approval of a checkpoint inhibitor for first-line treatment of lung cancer. This approval also expanded the indication in second-line treatment of lung cancer to include all patients with PD-L1-expressing NSCLC.

In a more recently reported open-label, phase 3 trial, KEYNOTE-042, in which 1274 patients with previously untreated advanced NSCLC, without EGFR or ALK mutations and with a PD-L1 tumor proportion score (TPS) of ≥1% were randomized to receive either pembrolizumab or the investigator's choice of platinum-based chemotherapy. The primary endpoint of the study was OS. Pembrolizumab did provide a survival benefit vs chemotherapy in the overall patient population (TPS ≥1%). Median OS was 16.7 months (95% CI: 13.9 to 19.7) in the pembrolizumab group versus 12.1 months (95% CI: 11.3 to 13.3) in the chemotherapy group (HR for disease progression or death: 0.81; 95% CI: 0.71 to 0.93; p=0.0018). However, the benefit was driven by the PD-L1 high population (≥50% expression, HR=0.69). Pembrolizumab did not provide a survival benefit in the 1% to 49% PD-L1 population (HR=0.92). In patients with TPS ≥50%, the median OS was 20.0 months (95% CI: 15.4 to 24.9) in the pembrolizumab group versus 12.2 months (95% CI:10.4 to 14.2) in the chemotherapy group (HR for disease progression or death: 0.69; 95% CI: 0.56 to 0.85; p=0.0003). In patients with TPS ≥20%, median OS was 17.7 months (95% CI: 15.3 to 22.1) in the pembrolizumab group versus 13.0 months (95% CI: 11.6 to 15.3) in the chemotherapy group (HR for disease progression or death: 0.77; 95% CI: 0.64 to 0.92; p=0.0020). In patients with TPS ≥50%, median PFS was 7.1 months (95% CI: 5.9 to 9.0) in the pembrolizumab group versus 6.4 months (95% CI: 6.1 to 6.9) in the chemotherapy group (HR=0.81 [95% CI, 0.67-0.99]; p=0.0170), which did not meet protocol-specified significance boundary. The median DOR was 20.2 months (range: 2.1+ to 31.2+ months) in the pembrolizumab arm vs 8.3 months in the chemotherapy arm (range: 1.8+ to 28.1) for patients with TPS ≥50% (De Lima Lopes 2018).

The results of KEYNOTE-042 in the subset of patients with TPS ≥50%, are different from those of KEYNOTE-024. Progression free survival and OS curves show that rapidly progressing patients (patients that progress or die within 6 months) treated with chemotherapy may have better outcomes than patients treated with immunotherapy. After 6 months, the curves cross and the benefit of the immunotherapy becomes apparent. The reasons for these differing outcomes are uncertain, and potentially reflect the inclusion of patients with more advanced or aggressive

disease in KEYNOTE-042, but in both studies the clinical benefit of PD-1 inhibition was still demonstrated in the population with TPS ≥50%.

Rationale for PD-1/PD-L1 Inhibitors in Combination with Standard-of-Care Chemotherapy Regimens

Programmed cell death-1/PD-L1 inhibitors have also been investigated in combination with standard-of-care chemotherapy regimens.

Non-squamous NSCLC

Results with pembrolizumab in chemotherapy-naïve patients with non-squamous NSCLC in combination with carboplatin and pemetrexed (KEYNOTE-021, phase 2, Cohort G [n=123]) have shown ORR of 57% with pembrolizumab with chemotherapy and 32% with chemotherapy (estimated difference, 25%; 95% CI, 7%–41%; P = 0.0029). PFS was significantly improved with pembrolizumab vs chemotherapy (HR, 0.54; 95% CI, 0.33–0.88; P = 0.0067) with median (95% CI) PFS of 19.0 (8.5–Not Reported [NR]) months vs 8.9 (95% CI, 6.2–11.8) month. Median follow-up was 18.7 months (range, 0.8–29.0 months). The HR for OS was 0.59 (95% CI, 0.34–1.05; P = 0.0344). Median (95% CI) OS was not reached (22.8–NR) months for pembrolizumab with chemotherapy and 20.9 (14.9–NR) months in the chemotherapy arm. 18-month OS rate was 70% with pembrolizumab with chemotherapy and 56% with chemotherapy (Borghaei 2018). These results led to the accelerated approval of pembrolizumab in combination with carboplatin and pemetrexed as first-line treatment in patients with non-squamous NSCLC in the US in 2017. In a confirmatory double-blind phase 3 study, KEYNOTE-189, 616 patients with non-squamous NSCLC who were treatment-naive for metastatic disease were randomly assigned to receive platinum-based chemotherapy with or without pembrolizumab. After a median follow-up of 10.5 months, the estimated rate of OS at 12 months was 69.2% (95% confidence interval [CI], 64.1 to 73.8) in the pembrolizumab with chemotherapy arm versus 49.4% (95% CI, 42.1 to 56.2) in the chemotherapy arm (HR for death, 0.49; 95% CI, 0.38 to 0.64; P<.001). Improvement in OS was seen across all PD-L1 categories that were evaluated. Median PFS was 8.8 months (95% CI, 7.6 to 9.2) in the pembrolizumab with chemotherapy arm versus 4.9 months (95% CI, 4.7 to 5.5) in the chemotherapy arm (HR for disease progression or death, 0.52; 95% CI, 0.43 to 0.64; P<0.001). The addition of pembrolizumab to chemotherapy resulted in significantly longer OS and PFS than chemotherapy alone in non-squamous NSCLC patients with metastatic disease (Gandhi 2018, KEYTRUDA® 2015). **In August 2018, pembrolizumab in combination with pemetrexed and carboplatin was approved by the FDA as first-line treatment of patients with metastatic non-squamous NSCLC. In September 2018, pembrolizumab in combination with pemetrexed and carboplatin was approved by the EMA for the same indication.**

The randomized, phase 3, IMpower150 trial enrolled patients with non-squamous NSCLC, but also evaluated pairing immunotherapy with an anti-angiogenic agent, along with chemotherapy. The 1202 patients enrolled in IMpower 150 were equally distributed to receive atezolizumab plus carboplatin/paclitaxel (Arm A), atezolizumab plus carboplatin/paclitaxel plus bevacizumab (Arm B), or carboplatin/paclitaxel plus bevacizumab (Arm C). IMpower 150 met the co-primary endpoints of PFS and OS, and demonstrated a statistically significant and clinically meaningful benefit with the atezolizumab/bevacizumab/chemotherapy regimen versus bevacizumab/chemotherapy regimen. Median OS reached 19.4 months for Arm B versus

14.7 months for Arm C (HR 0.780, 95% CI [0.636, 0.956]; p = 0.0164), and an analysis of PFS showed a median duration of 8.3 months for Arm B versus 6.8 months for Arm C (HR 0.592, 95% CI [0.499, 0.703]; p < 0.0001). The OS benefit of atezolizumab/bevacizumab/chemotherapy regimen was maintained across all key subgroups evaluated, including all PD-L1 subgroups, although the results were not always statistically significant (Socinski 2016). **In December 2018, the FDA approved atezolizumab in combination with bevacizumab, paclitaxel and carboplatin in patients with metastatic non-squamous NSCLC.**

Squamous NSCLC

KEYNOTE-407, a randomized, blinded, phase 3 study of pembrolizumab plus platinum-based chemotherapy improved OS, PFS, response rates, and duration of response in treatment-naïve patients with advanced squamous cell NSCLC compared with chemotherapy alone irrespective of PD-L1 status. Patients were randomized to receive pembrolizumab plus carboplatin and paclitaxel or nanoparticle albumin-bound paclitaxel versus the same chemotherapy plus placebo. At the second interim analysis, with a median follow-up of 7.8 months, OS was significantly improved in the pembrolizumab-containing arm: median OS of 15.9 months versus 11.3 months in the chemotherapy arm (P = 0.0008). PFS also favored pembrolizumab with chemotherapy: median of 6.4 months vs 4.8 months with chemotherapy (p < 0.0001). PFS was better with the addition of pembrolizumab in all levels of PD-L1expression, but the reduction on the rate of disease progression was proportional to PD-L1 expression in tumor. The ORR was significantly higher in the pembrolizumab/chemotherapy arm: 59.4% vs 38%, respectively (p = 0.0004). The median duration of response was 7.7 months vs 4.8 months, respectively (Paz-Ares 2018). **In October 2018, FDA Approved pembrolizumab in combination with carboplatin and either paclitaxel or nab-paclitaxel for the first-line treatment of patients with metastatic squamous NSCLC.**

IMpower131 is a phase 3, open-label, randomized study evaluating atezolizumab in combination with carboplatin and nanoparticle albumin-bound -paclitaxel or atezolizumab in combination with carboplatin and paclitaxel versus chemotherapy (carboplatin and nanoparticle albumin-bound paclitaxel) alone in treatment-naïve patients with stage IV squamous NSCLC. The study enrolled 1021 people who were randomized equally to receive atezolizumab plus carboplatin and paclitaxel (Arm A), or atezolizumab plus carboplatin and nanoparticle albumin-bound paclitaxel (Arm B), or carboplatin and nanoparticle albumin-bound paclitaxel (Arm C, control arm). Outcomes for only two of the study arms, however, were reported at ASCO 2018: atezolizumab plus chemotherapy (carboplatin and nanoparticle albumin-bound paclitaxel), 343 patients; and chemotherapy (carboplatin and nanoparticle albumin-bound paclitaxel), 340 patients. In this study, 29% of all patients, regardless of PD-L1 expression, had a reduced risk of disease worsening or death, compared with those who received chemotherapy alone. There was a doubling of PFS benefit with this combination: At 12 months, cancer had not worsened in 24.7% patients receiving atezolizumab and chemotherapy, compared to 12% of those receiving chemotherapy alone. Improved PFS was observed in all groups of patients who received atezolizumab and chemotherapy, including those with PD-L1–negative tumors. OS data are not yet mature (Jotte 2018).

Rationale for Ipilimumab in Combination with PD-1/PD-L1 Inhibitors and 2 Cycles of Standard of Care Chemotherapy Regimens (Part 1)

A second type of immuno-oncology agent, anti-cytotoxic T-lymphocyte-associated antigen 4 (anti-CTLA-4), has also been demonstrated to be clinically active in advanced cancers. Ipilimumab, a human monoclonal anti-CTLA-4 immunotherapy, has demonstrated clinical activity as monotherapy in melanoma (Wolchok 2010, O'Day 2010) and prostate cancer (Slovin 2013) and is now approved in the US and EU for treatment of unresectable or metastatic melanoma and for the adjuvant treatment of melanoma (YERVOY® Package Insert, YERVOY SmPC). In lung cancer ipilimumab does not add benefit to standard of care chemotherapy (Govindan 2017). Combination immunotherapies that include an anti-CTLA-4 agent and an anti-PD-1/PD-L1 agent have the potential for additive or synergistic effects (reviewed in Buchbinder 2016).

Nivolumab as combination immunotherapy with ipilimumab was evaluated in CheckMate 012, a phase 1 study in treatment-naïve patients with advanced NSCLC (Hellmann 2017). Patients received nivolumab plus ipilimumab at one of 3 dose regimens or nivolumab monotherapy (monotherapy treatment group results not reported in Hellmann 2017). Confirmed response rates were 47% (95% CI: 31% to 64%) in patients receiving nivolumab every 2 weeks (Q2W) plus ipilimumab every 12 weeks (Q12W) and 38% (95% CI: 23% to 55%) in patients receiving nivolumab Q2W plus ipilimumab every 6 weeks (Q6W). Median DOR was not reached in either treatment group (median follow-up times of 12.8 months in the patients receiving nivolumab Q2W plus ipilimumab Q12W and 11.8 months in the patients receiving nivolumab Q2W plus ipilimumab Q6W. The greatest percentage of responses was noted in patients with tumors that expressed PD-L1. In patients whose tumors expressed PD-L1 in ≥1% of tumor cells, confirmed objective responses were achieved in 12 of 21 (57%) patients in the ipilimumab Q12W treatment group and 13 of 23 (57%) patients in the ipilimumab Q6W treatment group (Hellmann 2017). The 1-year survival rate in patients treated with nivolumab 3 mg/kg Q2W plus ipilimumab 1 mg/kg Q6W or Q12W was 100% in patients with tumors that expressed PD-L1 in ≥50% of tumor cells (n=13) and was 76% in all-comers (all patients regardless of PD-L1 status [n=77]), compared to 73% for patients receiving nivolumab monotherapy (n=52). One-year survival in patients with tumors that expressed PD-L1 in >1% of tumor cells was 91% in the nivolumab 3 mg/kg plus ipilimumab 1 mg/kg Q12W treatment group and 83% in the nivolumab 3 mg/kg plus ipilimumab 1 mg/kg Q6W treatment group (n=23 in each treatment group) compared to 73% in the nivolumab monotherapy treatment group (n=32) (Gettinger 2016). The improved efficacy in NSCLC patients whose tumors expressed PD-L1 contrasts to the phase 3 study in patients with melanoma (Larkin 2015), where addition of anti-CTLA-4 to anti-PD-1 provided benefit predominantly to those patients whose tumors had low baseline PD-L1 expression.

CheckMate 227 is an open-label, Phase 3, trial with more than 2500 treatment-naïve patients with stage IV or recurrent NSCLC, evaluating nivolumab-based regimens versus platinum-doublet chemotherapy. This study consists of three parts – Parts 1a and 1b and Part 2. Part 1a evaluates nivolumab plus low-dose ipilimumab (1 mg/kg every 6 weeks) versus chemotherapy in patients whose tumors express PD-L1. Part 1b evaluates nivolumab plus low-dose ipilimumab (1 mg/kg every 6 weeks) and nivolumab plus chemotherapy versus chemotherapy in patients whose tumors do not express PD-L1. Part 2 evaluates nivolumab plus chemotherapy versus chemotherapy in a broad population with a primary endpoint of OS. There are two co-primary endpoints in Part 1 for the nivolumab plus ipilimumab combination: OS in patients whose tumors express PD-L1 (assessed in patients enrolled in Part 1a) and PFS in patients with

VV-RIM-00102568-2.0 Approved - 16 Apr 2020 GMT-5:00

high tumor mutation burden (TMB), regardless of PD-L1 expression (assessed in patients enrolled across Parts 1a and 1b) (BMS Press Release, 2018). An analysis of Part 1 revealed that PFS among patients with a high TMB was significantly longer with nivolumab plus ipilimumab than with chemotherapy. The one-year PFS rate was 42.6% with nivolumab plus ipilimumab versus 13.2% with chemotherapy, and the median PFS was 7.2 months (95% confidence interval [CI], 5.5 to 13.2) versus 5.5 months (95% CI, 4.4 to 5.8) (HR for disease progression or death, 0.58; 97.5% CI, 0.41 to 0.81; P<0.001). The ORR was 45.3% with nivolumab plus ipilimumab and 26.9% with chemotherapy. The benefit of nivolumab plus ipilimumab over chemotherapy was broadly consistent within subgroups, including patients with a PD-L1 expression level of at least 1% and those with a level of less than 1% (Hellmann 2017).

Combination immunotherapy with durvalumab (anti-PD-L1) plus tremelimumab (anti-CTLA-4) is currently being evaluated in multiple cancer types, including urothelial carcinoma (NCT02516241), squamous cell carcinoma of the head and neck (NCT02551159), and renal cell carcinoma (RCC) (NCT02762006). In patients with NSCLC, the durvalumab plus tremelimumab combination demonstrated clinical activity, regardless of PD-L1 expression levels. Investigator-reported confirmed responses occurred in 23% of patients (95% CI: 9% to 44%) in the durvalumab plus tremelimumab combination therapy group, including 22% (95% CI: 3% to 60%) of patients with PD-L1-positive tumors (tumors that expressed PD-L1 on ≥25% of tumor cells) and 29% (95% CI: 8% to 58%) of patients with PD-L1-negative tumors (tumors that expressed PD-L1 on <25% of tumor cells) (Antonia 2016). However, a phase 3 trial of durvalumab in combination with tremelimumab (MYSTIC) failed to significantly improve PFS or OS over standard-of-care chemotherapy in PD-L1-positive patients with advanced NSCLC (AstraZeneca Press Release, 27 July 2017; AstraZeneca Press Release, 16 Nov 2018).

Rationale for Cemiplimab and Ipilimumab and limited (2 Cycles) Platinum-based Doublet Chemotherapy (Part 1)

The combination of cemiplimab and ipilimumab is accompanied by administration of the first 2 cycles of standard platinum doublet chemotherapy (on an every 3 weeks [Q3W] schedule).

The hypothesis for the proposed addition of 2 cycles of platinum-based chemotherapy is that initial tumor control mediated by chemotherapy will provide more time for an effective immune response to mature, and this immune response may, in fact, be enhanced by chemotherapy-induced cell death, and augmented antigen presentation. The rationale for limiting the chemotherapy to 2 cycles is to limit the risk of triple combination-related toxicities and to limit the interference of longer administration of the cytotoxic regimens with the immuno-oncology combination therapy. There are other trials exploring this strategy, for example CheckMate 9LA (NCT03215706). Limiting chemotherapy to 2 cycles obviates the need to separately study this arm to fulfill the combination rule as it would be considered a subtherapeutic regimen. Chemotherapy in combination with ipilimumab alone has been proven ineffective (AstraZeneca Press Release, 27 July 2017; AstraZeneca Press Release, 16 Nov 2018, Hellmann 2017), as such there is no need to study ipilimumab separately to fulfill the combination rule.

Cemiplimab Safety and Efficacy

Cemiplimab (the INN for REGN2810) is a human IgG4 monoclonal antibody (mAb) to the PD-1 receptor that blocks PD-1/PD-L1 mediated T cell inhibition. Cemiplimab is being evaluated in more than 20 Phase 1-3 clinical studies. Regeneron has initiated Phase 2 and Phase 3 trials of

cemiplimab in several indications: advanced or metastatic PD-L1 positive NSCLC, advanced cutaneous squamous cell carcinoma (CSCC), advanced basal cell carcinoma (BCC), and metastatic or recurrent cervical cancer after platinum-based therapy. In 2018, Cemiplimab was approved in the United States (US) by the FDA as cemiplimab-rwlc for the treatment of patients with metastatic cutaneous squamous cell carcinoma (CSCC) or locally advanced CSCC who are not candidates for curative surgery or curative radiation. It is under review for approval for the same indication in the European Union (EU).

As of 27 March 2018, 757 patients have been treated with cemiplimab either as monotherapy or in combination with radiotherapy and/or other cancer therapies. The safety profile of cemiplimab appears to be generally consistent across tumor types and dose of cemiplimab, and consistent with other PD-1 inhibitors.

Efficacy evaluation is currently based on phase 1 Study R2810-ONC-1423 dose escalation cohorts, selected expansion cohorts (CSCC and NSCLC), and a phase 2 study (R2810-ONC-1540) in CSCC. In study 1423, among 60 patients who received cemiplimab in dose escalation cohorts (1 mg/kg, 3 mg/kg, or 10 mg/kg every 2 weeks), the objective response rate was 18.3% (9 partial responses, 2 complete responses). Among 21 patients with NSCLC (one patient in dose escalation, 20 patients in expansion cohort 1 that studied cemiplimab 200 mg monotherapy every two weeks), the response rate according to independent central review was 29% (6 partial responses). Among 26 patients in the CSCC expansion cohorts of study 1423 (including both patients with locally advanced disease and those with metastatic disease), the response rate according to independent central review was 50% (13 partial responses). Study 1540 enrolled 59 patients with metastatic CSCC, and the response rate per independent central review was 48% (4 complete responses, 24 partial responses).

Expanded rationale for revising study to transition to a two-part study

Study R2810-ONC-16113 is a two-part randomized, global study. Under Amendment 4, the protocol was modified to include a "Part 2" of the study that will be conducted in a double-blind fashion in which patients with non-squamous and squamous NSCLC are randomized in a 2:1 fashion to cemiplimab plus platinum-based doublet chemotherapy (cemiplimab/chemo-f) or placebo plus platinum-based doublet chemotherapy (placebo/chemo-f). There are design elements that are different from Part 1 of the study that is enrolling patients in an open-label fashion at the time of this amendment. The primary reason for the introduction of Part 2 is the changing landscape of immunotherapy in NSCLC. KEYNOTE-189 and KEYNOTE-407 showed that the combination of PD-1 plus platinum doublet chemotherapy leads to superior outcomes for patients of all PD-L1 expression levels compared to monotherapy PD-1 inhibitors or platinum doublet chemotherapy alone. The role of adding CTLA-4 to PD-1 inhibitors and/or platinum doublet chemotherapy remains investigational at this time. Part 2 will therefore focus on the comparison of cemiplimab/chemo-f versus placebo/chemo-f for all PD-L1 expression levels.

The use of placebo in combination with standard chemotherapy will ensure the objectivity of assessment of disease progression and decisions to interrupt or discontinue therapy. A 2:1 randomization to cemiplimab/chemo-f to placebo/chemo-f will maximize the number of patients benefiting from the combination treatment and will minimize barriers to patient enrollment.

Part 1 stopped patient enrollment and by the end of August 2019, 323 patients were enrolled in Part 1. Under Amendment 5, Part 1 of the study will assess whether cemiplimab/chemo-f or

cemiplimab/chemo-l/ipi improves OS, PFS, or ORR over platinum-based doublet chemotherapy in patients with PD-L1 <50%.

In Part 1, OS is the primary endpoint. An interim analysis of OS will be performed when 125 (83%) OS events have been observed. Final OS will be conducted at approximately 151 OS events. Final analysis of PFS will be performed hierarchically when analysis of OS is statistically significant either at the interim analysis or at final analysis for OS. To extend the assessment of efficacy and safety to include never-smokers, the study population in Part 2 of the study will no longer be limited to previous and current smokers. Results of the PD-1 inhibitor/chemotherapy combination studies KEYNOTE-189 and KEYNOTE-407 have shown that never smokers equally benefit from the combination therapy, which had not been the case for monotherapy with PD-1 inhibitors.

In Part 2, OS is the primary endpoint. Two interim analyses of OS will be performed when 146 (50%) and 204 (70%) out of total 291 OS events have been observed. Final analysis of PFS will be performed hierarchically when analysis of OS is statistically significant either at the second interim analysis or at final analysis for OS.

2. STUDY OBJECTIVES

2.1. Primary Objectives

Part 1: To compare the OS of cemiplimab/chemo-f and cemiplimab/chemo-l/ipi versus platinum-based doublet chemotherapy in the first-line treatment of patients with advanced squamous or non-squamous NSCLC with tumors expressing PD-L1 in <50% of tumor cells.

Part 2: To compare the OS of cemiplimab/chemo-f with placebo/chemo-f in the first-line treatment of patients with advanced squamous or non-squamous NSCLC irrespective of PD-L1 expression.

2.2. Secondary Objectives

2.2.1. Key Secondary Objectives

The key secondary objectives of the study are the following:

- Part 1: To compare the PFS and ORR of cemiplimab/chemo-f and cemiplimab/chemo-l/ipi versus chemo-f in the first-line treatment of patients with advanced squamous or non-squamous NSCLC and tumors expressing PD-L1 in <50% of tumor cells.
- Part 2: To compare the PFS and ORR of cemiplimab/chemo-f versus placebo/chemo-f in the first-line treatment of patients with advanced squamous or non-squamous NSCLC irrespective of PD-L1 expression.

Fwrror

VV-RIM-00102568-2.0 Approved - 16 Apr 2020 GMT-5:00

2.2.2. Other Secondary Objectives

The other secondary objectives are the following:

- Parts 1 and 2: To evaluate the safety and tolerability of cemiplimab/chemo-l/ipi and/or cemiplimab/chemo-f compared to platinum-based doublet chemotherapy or placebo/chemo-f
- Parts 1 and 2: To evaluate the DOR of cemiplimab/chemo-l/ipi and/or cemiplimab/chemo-f compared to platinum-based doublet chemotherapy or placebo/chemo-f in the first-line treatment of patients with advanced squamous or non-squamous NSCLC.
- Parts 1 and 2: To compare QOL in patients with advanced squamous or non-squamous NSCLC receiving cemiplimab/chemo-l/ipi and/or cemiplimab/chemo-f compared to platinum-based doublet chemotherapy or placebo/chemo-f
- Part 1: To evaluate the OS rate at 12 months, 18 months, and 24 months of cemiplimab/chemo-f and cemiplimab/chemo-l/ipi versus chemo-f in the first-line treatment of patients with advanced squamous or non-squamous NSCLC with PD-L1 expression in <50% of tumor cells.
- Part 2: To evaluate the OS rate at 12, 18, and 24 months of cemiplimab/chemo-f versus placebo/chemo-f in the first-line treatment of patients with advanced squamous or non-squamous NSCLC irrespective of PD-L1 expression.
- Parts 1 and 2: To assess immunogenicity as measured by anti-drug antibodies (ADAs) for cemiplimab
- Parts 1 and 2: To assess the predictive utility of baseline PD-L1 tumor expression levels on clinical response
- Part 1: to characterize the pharmacokinetics (PK) of cemiplimab when administered in combination with ipilimumab or in combination with platinum-based doublet chemotherapy.
- Part 2: To characterize the PK of cemiplimab when administered in combination with platinum-based doublet chemotherapy
- Parts 1 and 2: To conduct exposure-response (E-R) analyses for relevant biomarkers (exploratory PK/pharmacodynamic analyses) and E-R analyses for safety and efficacy endpoints, as appropriate
- Parts 1 and 2: Tumor mutation burden as assessed by the Foundation Medicine "FoundationOne®" panel, sample permitting.

3. HYPOTHESIS AND RATIONALE

3.1. Hypothesis

The hypotheses of Part 1 and Part 2 are that cemiplimab/chemo-f and/or cemiplimab/chemo-l/ipi will prolong OS compared to platinum-based chemotherapy with advanced squamous or non-squamous NSCLC.

3.2. Rationale

3.2.1. Rationale for Study Design

See **Expanded rationale for revising study to transition to a two-part study in Section** 1.

Part 1

See Rationale for Ipilimumab in Combination with PD-1/PD-L1 Inhibitors and 2 Cycles of Standard of Care Chemotherapy Regimens in Section 1.

Because this is the first study to evaluate the combination of cemiplimab and ipilimumab, there will be an early review of data to ensure patient safety after the first 10 patients in the cemiplimab/chemo-l/ipi treatment arm have completed 4 weeks of follow-up following the first dose of cemiplimab/chemo-l/ipi.

Additionally, for Treatment Arms A and B, for patients with non-squamous NSCLC who are receiving a pemetrexed-containing doublet, pemetrexed maintenance is mandatory as maintenance therapy may provide benefit.

Patients in the chemotherapy Treatment Arm A will be given the option to receive cemiplimab monotherapy at the time of disease progression or after administration of chemotherapy, given that PD-1 inhibitors are now used for second-line treatment of NSCLC (Ettinger 2016).

The study population is limited to previous and current smokers as the benefit of PD-1 blockade has not been shown to the same extent in non-smokers, likely due to the lower mutational burden in this population (Reck 2016).

Part 2

See Rationale for PD-1/PD-L1 Inhibitors in Combination with Standard-of-care Chemotherapy Regimens in Section 1.

As in Part 1, patients with non-squamous NSCLC who receive a pemetrexed-containing doublet, pemetrexed maintenance is mandatory.

3.2.2. Rationale for Endpoints and Objectives

The primary objective of the Part 2 of this study is to compare the endpoint of OS of cemiplimab/chemo-f to placebo/chemo-f in non-squamous and squamous NSCLC patients irrespective of PD-L1 expression. OS has been recognized as the gold standard for demonstrating benefit of antineoplastic therapies in randomized clinical trials.

CONFIDENTIAL

VV-RIM-00102568-2.0 Approved - 16 Apr 2020 GMT-5:00

3.2.3. Rationale for Cemiplimab Dose Selection

The proposed cemiplimab dose of 350 mg Q3W given by intravenous (IV) route was selected in this study for patients with NSCLC to better align with the dosing schedule of planned concurrent therapies, namely with ipilimumab (50 mg Q6W for 4 doses) and with platinum-based doublet chemotherapy (day 1 or days 1 and 8, Q3W). The cemiplimab dose of 350 mg Q3W was also selected for an ongoing pivotal phase 3 study in patients with NSCLC.

In the first-in-human (FIH) study (study R2810-ONC-1423) the 3 mg/kg Q2W IV dose has shown anti-tumor activity and acceptable safety in NSCLC patients among other patient types; efficacy was also observed at the 1 mg/kg Q2W dose. As many standard chemotherapy treatments for NSCLC are dosed on a Q3W schedule, the clinical development strategy of cemiplimab coalesced around a Q3W treatment interval. The ongoing clinical development of cemiplimab also seeks to incorporate a fixed dosing paradigm. As such, a Q3W fixed dose regimen has been selected that is expected to provide a similar clinical efficacy and safety profile to that observed for the 3 mg/kg Q2W regimen.

The preference of a fixed dose over a body weight adjusted dose for anti-PD-1 monoclonal antibodies is supported by a wide safety margin (no maximum tolerated dose [MTD] observed), a flat E-R relationship for safety and efficacy over the therapeutic dosing range, and similar variability in exposure (CV%) after fixed and body weight adjusted dose (Freshwater 2017, Zhao 2017a). In fact, pembrolizumab and nivolumab were initially approved at a body-weight adjusted dose of 2 mg/kg Q3W and 3 mg/kg Q2W, respectively, and were subsequently approved by US FDA at fixed doses of 200 mg Q3W and 240 mg Q2W, respectively, for the treatment of melanoma and NSCLC (KEYTRUDA Package Insert, OPDIVO Package Insert).

A fixed IV cemiplimab dose of 350 mg Q3W was selected, based on population PK modeling and simulation and this dose provides exposure that is similar to 3 mg/kg Q2W IV regimen in study R2810-ONC-1423 (NCT02383212).

1. The variability in cemiplimab exposure was similar for body weight-adjusted doses as compared to fixed doses, therefore supporting fixed dose selection.
2. A 350 mg Q3W dose in a patient population with body weights varying around an average value of 80 kg resulted in similar (<20% difference) C_{trough}, area under the curve from time 0 to week 12 (AUC_{12W}), and peak concentration (C_{max}) at steady state compared to the 3 mg/kg Q2W dose used in the FIH study. Cemiplimab concentrations with 350 mg Q3W exceeded those observed with 1 mg/kg Q2W, a dose that demonstrated clinical efficacy in the FIH study, and C_{trough} values with 350 mg Q3W exceeded concentrations of ~5 to 20 mg/L, above which saturation of PD-1 target occupancy is expected to occur based on linearity assessment on single-dose pharmacokinetics in cynomolgus monkeys.

The 350 mg Q3W dose of cemiplimab is therefore used in the phase 3 studies in patients with NSCLC and across the cemiplimab program.

Some populations (eg, in Japan or other countries in Asia-Pacific Rim) have slightly lower average body weights (60 kg) compared to Western population (80 kg) (Shimizu 2016). Based on the effect of body weight on cemiplimab exposure, population PK modeling and simulations indicated that patients with average body weight of 60 kg, representing the Asian and/or

Japanese patient population, would have a slightly higher cemiplimab exposure (~16%) compared to a Western patient population via an average body weight of 80 kg, which is clinically irrelevant considering the flat exposure-response relationship for efficacy and safety. With the lack of any added safety signal in the existing clinical data at doses up to 10 mg/kg Q2W, the existing data support the use of the 350 mg Q3W treatment regimen for the global development of cemiplimab.

3.2.4. Rationale for Ipilimumab Dose Selection (Part 1 Only)

Data presented by Bristol-Meyers Squibb, Inc. demonstrated higher tumor shrinkage in patients treated with nivolumab at 3 mg/kg Q2W plus ipilimumab at 1 mg/kg Q6W or Q12W compared to nivolumab monotherapy (Zhao 2017b). The incidence of AEs was similar for nivolumab monotherapy and nivolumab plus ipilimumab at 1 mg/kg Q6W or Q12W, but was higher in treatment groups with more frequent and/or higher ipilimumab dosing. Based on the presented risk-benefit assessment (Zhao 2017b) and exposure-response analyses, the recommended dose of ipilimumab in NSCLC is 1 mg/kg Q6W, which is equivalent to approximately 50 mg, the dose proposed in the present study.

3.2.5. Rationale for Platinum-Based Chemotherapy as Comparator

Platinum-based doublet chemotherapy or placebo/chemo-f serve as the active comparator in this two-part study. There is no single "best" standard platinum-based doublet chemotherapy for squamous or non-squamous NSCLC. Randomized studies that have compared various regimens have not shown any difference in survival (Fossella 2003, Scagliotti 2002, Schiller 2002). Pemetrexed-based doublets are restricted to non-squamous NSCLC (ALIMTA® US Package Insert, ALIMTA European Union Summary of Product Characteristics).

The PARAMOUNT study is a large study of pemetrexed in advanced non-squamous NSCLC (Paz-Ares 2013). In this study, 939 patients with advanced non-squamous NSCLC were given 4 cycles of pemetrexed-cisplatin induction therapy. Of these patients, 539 patients with no disease progression and Eastern Cooperative Oncology Group (ECOG) performance statuses of 0 or 1 were then randomly assigned in a 2:1 ratio to receive maintenance pemetrexed (500 mg/m^2 on day 1 of each 21-day cycle; n=359) or placebo (n=180). The results demonstrated that maintenance pemetrexed resulted in a statistically significant 22% reduction in the risk of death (HR: 0.78; 95% CI: 0.64 to 0.96; p=0.0195; median OS: pemetrexed: 13.9 months, placebo: 11.0 months). Survival on pemetrexed consistently improved for all patient subgroups, including the induction response subgroups: complete/partial responders (n=234) OS HR: 0.81; 95% CI: 0.59 to 1.11; SD (n=285) OS HR: 0.76; 95% CI: 0.57 to 1.01. However, drug-related grade 3 to 4 toxicities of anemia, fatigue, and neutropenia were significantly higher in pemetrexed-treated patients. As such, the investigator in this study will decide, in consultation with an eligible patient, whether or not to include pemetrexed maintenance in the treatment regimen. A decision not to include pemetrexed maintenance will be documented in the case report form (CRF) (throughout this protocol, CRF refers to either a paper CRF or an electronic CRF [eCRF]).

Both cisplatin and carboplatin are used as standard-of-care chemotherapy agents, although carboplatin may be associated with fewer side effects. Given this and that clinical practice preferences will vary in this global study, investigators will be given the option to choose from

several approved treatment regimens. Administration of 4 cycles (chemo-f) of chemotherapy is standard; In Part 1 Arm C, patients will be administered chemotherapy for 2 cycles (chemo-l).

4. STUDY VARIABLES

4.1. Demographic and Baseline Characteristics

Baseline characteristics will include standard demography (eg, age, race, ethnicity, weight, gender, and height) and disease characteristics, including PD-L1 status and medical and oncology history.

4.2. Primary and Secondary Endpoints

4.2.1. Primary Endpoint (Part 1 and Part 2)

The primary endpoint for Part 1 and Part 2 of the study is OS. Overall survival is defined as the time from randomization to the date of death due to any cause. All deaths due to any cause occurring on or before cut-off date in the full analysis set (FAS) will be used in the OS analysis. If a patient is not known to have died or is lost to follow up at the time of analysis cutoff date, the one will be censored at the last date that the patient was known to be alive.

4.2.2. Key Secondary Endpoints (Part 1 and Part 2)

For Part 1, the key secondary endpoints are PFS and ORR as assessed by IRC and investigator per RECIST 1.1 (Eisenhauer 2009; see Appendix 2).

For Part 2, the key secondary endpoints are PFS and ORR as assessed by IRC per RECIST 1.1.

Progression-free survival is defined as the time from randomization to the date of the first documented tumor progression or death due to any cause, whichever occurred earlier. Patients will be censored according to the rules listed below:

1. Patients who do not have a documented tumor progression or death will be censored on the date of their last evaluable tumor assessment.
2. Patients who do not have a documented tumor progression or death before initiation of new anti-tumor therapy will be censored on the date of their last evaluable tumor assessment prior to or on the date of new anti-tumor therapy.
3. Patients who withdraw consent before taking any study treatment, therefore there is no post baseline tumor assessment, will be censored at the date of randomization.
4. Patients who do not have any evaluable tumor assessments after randomization and did not die will be censored on the date of randomization.

Objective response rate will be defined as the number of patients with a best overall response (BOR) of confirmed CR or PR divided by the number of patients in the efficacy analysis set. Patient(s) without baseline tumor assessment, or with either unknown or missing BOR will be included in the denominator and will be counted as non-responder(s).

VV-RIM-00102568-2.0 Approved - 16 Apr 2020 GMT-5:00

4.2.3. Other Secondary or Exploratory Endpoints

Other secondary or exploratory endpoints will include the following:

- DOR is defined as the time from date of first documented response of CR or PR to the date of first documented PD or death due to any cause, whichever occurred earlier. Patients continuing without PD or death due to any cause will use the same censoring rule as PFS as stated in Section 4.2.2.
- Best overall response is defined as the BOR, as determined by the IRC or investigator per RECIST 1.1, between the date of randomization and the date of the first objectively documented progression or death due to any cause, whichever occurred earlier. BOR of CR or PR must be confirmed by subsequent evaluations of overall response of CR or PR at time points at least 4 weeks apart. BOR of SD must have met the response SD criteria at least once ≥39 days (6 weeks*7 days/week -3 days) after randomization. BOR of (early) PD does not require confirmation. BOR for patients who do not have any post-baseline tumor assessment will be not evaluable (NE).
- The safety and tolerability of study regimens measured by the incidence of TEAEs, dose-limiting toxicities (DLTs), serious adverse events (SAEs), deaths, and laboratory abnormalities
- Overall survival rate at 12 months, 18 months, and 24 months.
- Quality of life as measured by the European Organization for Research and Treatment of Cancer Quality of Life Questionnaire Core 30 (EORTC QLQ-C30) and Quality of Life Questionnaire Lung Cancer 13 (EORTC QLQ-LC13).

4.3. Pharmacokinetic Variables

In patients randomized to the cemiplimab/chemo-f or cemiplimab/chemo-l/ipi treatment arms, cemiplimab concentrations in serum will be assessed at multiple time points throughout the treatment and follow-up periods.

Pharmacokinetic variables are cemiplimab concentrations in serum over time.

- C_{eoi} – concentration at end of infusion
- C_{trough} – pre-infusion concentration
- t_{eoi} – time of end of infusion

4.4. Anti-Drug Antibody Variables

The ADA variables will be measured in samples from patients randomized to the cemiplimab/chemo-f or cemiplimab/chemo-l/ipi treatment arms . Anti-drug antibody variables include status of ADA response and titer as follows:

- Treatment-boosted ADA response - defined as a positive response in the ADA assay after the first dose that is greater than or equal to 9-fold over baseline titer levels, when baseline results are positive

CONFIDENTIAL

VV-RIM-00102568-2.0 Approved - 16 Apr 2020 GMT-5:00

- Treatment-emergent ADA response - defined as a positive response in the ADA assay after the first dose when baseline results are negative or missing
  - The treatment-emergent ADA responses will be further categorized into persistent, indeterminate, and transient responses.
- Titer category is defined based on values as (titer value category):
  - Low (titer <1000)
  - Moderate (1000≤ titer ≤10,000)
  - High (titer >10,000)
- Neutralizing anti-drug antibody (NAb) activities for samples that are confirmed positive in the ADA assay

5. STUDY DESIGN

5.1. Study Description and Duration

This is a two-part, randomized, global phase 3 study of cemiplimab/chemo-l/ipi and/or cemiplimab/chemo-f versus platinum-based doublet chemotherapy or placebo/chemo-f in the first-line treatment of patients with advanced squamous or non-squamous NSCLC.

Part 1 of the study will compare the efficacy and safety of cemiplimab/chemo-l/ipi and cemiplimab/chemo-f versus platinum-based doublet chemotherapy in patients whose tumors express PD-L1 in <50% of tumor cells (measured using a PD-L1 IHC assay). A study flow diagram is presented in Figure 1.

Part 2 of the study will compare the efficacy and safety of cemiplimab/chemo-f versus placebo/chemo-f in patients irrespective of PD-L1 expression. Randomization in Part 2 will be 2:1 and stratified by histology and PD-L1 expression (measured using a PD-L1 IHC assay). Patients with squamous NSCLC will be capped at 50% of the total sample size. At least 30% but no more than 40% of patients enrolled must have tumors that express PD-L1 in ≥50% of tumor cells. Enrollment of patients whose tumors express PD-L1 in <1% of tumor cells will be capped at 30%. (PD-L1 <50% is capped at 70%). This approach is adopted to maintain an approximately equal distribution of patients in each PD-L1 stratum.

A study flow diagram is presented in Figure 2.

VV-RIM-00102568-2.0 Approved - 16 Apr 2020 GMT-5:00

Figure 1: Study Flow Diagram – Part 1

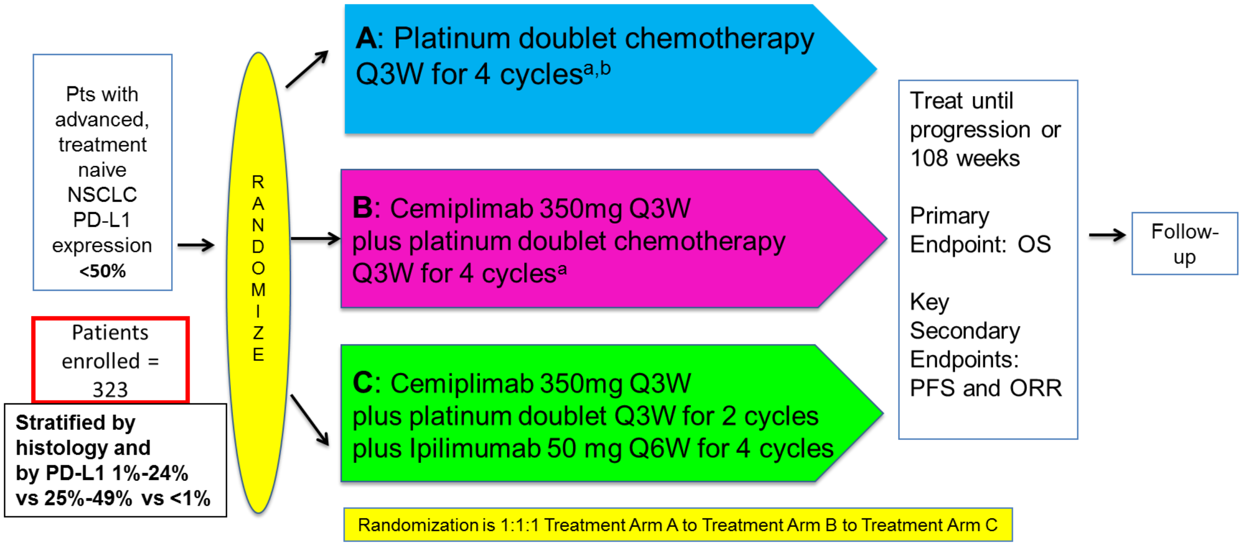

Abbreviations: NSCLC=non-small cell lung cancer; ORR=objective response rate; OS=overall survival; PD-L1=programmed death ligand 1; PFS=progression-free survival; Q3W=every 3 weeks; Q6W=every 6 weeks; W=weeks

[a] For Treatment Arms A and B: For patients with non-squamous NSCLC for whom the investigator chooses a pemetrexed-containing doublet, pemetrexed maintenance will be mandatory.

[b] optional cemiplimab 350 mg for up to 108 weeks at PD or after administration of chemotherapy

Part 1 consists of the following 3 periods: screening, treatment, and follow-up. After screening, eligible patients will be randomized to Treatment Arm A (chemo-f), Treatment Arm B (cemiplimab/chemo-f), or Treatment Arm C (cemiplimab/chemo-l/ipi). The length of the treatment period will determined by the treatment arm to which a patient is randomized. Treatment may be discontinued early due to RECIST 1.1-defined progressive disease, withdrawal of consent, death, unacceptable toxicity, initiation of another anti-cancer treatment, or, for patients in Treatment Arms B and C, in specific instances of confirmed CR or PR (Section 5.1.2). After discontinuing study treatment, patients will enter the follow-up period.

The approximate duration of the active study assessments for each patient, excluding screening, will be 24 to 32 months. For Treatment Arms B and C, this encompasses 25 months of study treatment plus 7 months of follow-up. For patients in Treatment Arm A, this encompasses 4 cycles of treatment (and pemetrexed maintenance, if appropriate) and radiographic tumor assessments for up to 2 years, if there is no disease progression; otherwise patients in Treatment Arm A advance to the 7 month follow up period.

For Part 1 only, patients on Treatment Arm A who experience disease progression while on chemotherapy or after administration of chemotherapy will be offered the option to receive cemiplimab 350 mg Q3W for up to 108 weeks, provided they meet specific criteria. After the active portion of the study is complete, all patients will be followed for survival.

Figure 2: Study Flow Diagram – Part 2

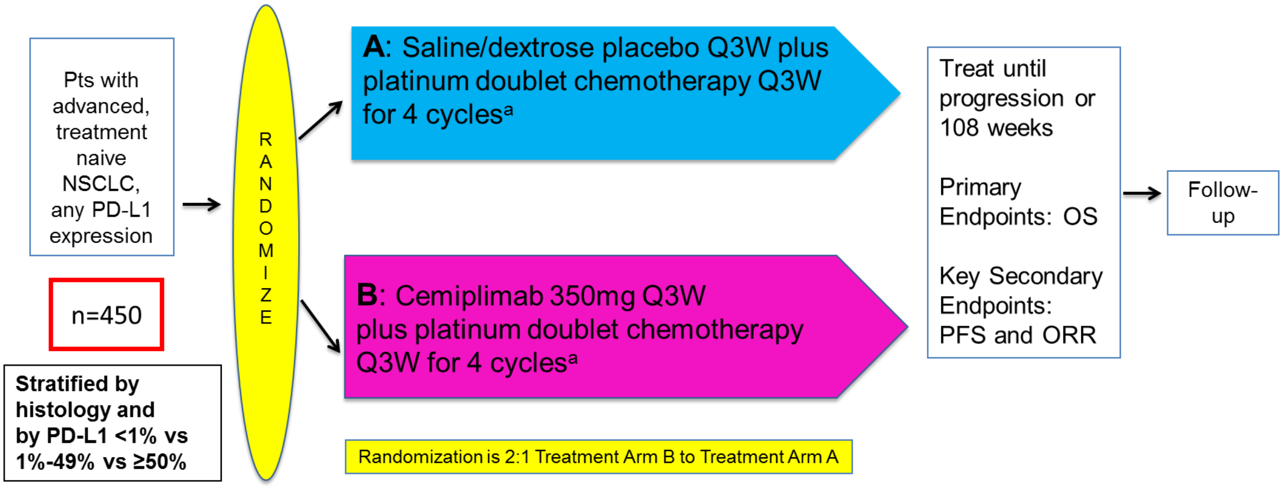

Abbreviations: NSCLC=non-small cell lung cancer; ORR=objective response rate; OS=overall survival; PD-L1=programmed death ligand 1; PFS=progression-free survival; Q3W=every 3 weeks; Q6W=every 6 weeks; W=weeks

[a] For Treatment Arms A and B: For patients with non-squamous NSCLC for whom the investigator chooses a pemetrexed-containing doublet, pemetrexed maintenance will be mandatory.

VV-RIM-00102568-2.0 Approved - 16 Apr 2020 GMT-5:00

Part 2 consists of the following 3 periods: screening, treatment, and follow-up. After screening, eligible patients will be randomized to Treatment Arm A (placebo/chemo-f) or Treatment Arm B (cemiplimab/chemo-f). Treatment may be discontinued early due to RECIST 1.1-defined progressive disease, withdrawal of consent, death, unacceptable toxicity, or initiation of another anti-cancer treatment (Section 5.1.2). After discontinuing study treatment, patients will enter the follow-up period. For Part 2, patients on Treatment Arm A who experience disease progression will enter follow up and other anticancer treatment should be considered at this time at the discretion of the investigator.

The approximate duration of the active study assessments for each patient, excluding screening, will be 32 months. This encompasses 25 months of study treatment plus 7 months of follow-up. Patients will be unblinded to treatment at the time of progressive disease defined by RECIST1.1 criteria or discontinuation from study.

5.1.1. Screening

Part 1

Patients will undergo a screening evaluation to determine their eligibility within 28 days prior to randomization (Table 4). PD-L1 expression in a tumor tissue sample (archival tissue, if ≤5 months old, or recently obtained on-study biopsy collected during screening; for collection instructions, refer to the laboratory manual) will be assessed using a PD-L1 IHC assay by a central laboratory (Section 8.2.5). The use of the PD-L1 IHC assays for decisions regarding treatment with cemiplimab is considered investigational. Patients whose tumors express PD-L1 in <50% of tumor cells will continue in screening, while those whose tumors express PD-L1 in ≥50% of tumor cells will be excluded from the study. Given that non-squamous (specifically adenocarcinoma) histology is more prevalent than squamous histology, it is predicted that approximately 70% of patients enrolling in the study will have non-squamous NSCLC and 30% of patients will have squamous NSCLC.

Tumor tissue samples will also be tested for EGFR mutations and ALK translocations as well as for ROS1 fusions, unless this testing has already been performed and the results are available. Patients whose tumors are positive for any of these mutations/fusions (by testing at screening or by previous results) will not be eligible for the study.

Baseline radiographic tumor assessments should also be performed within 28 days prior to randomization (Table 4). These assessments will not be reviewed by the IRC for eligibility assessment.

Informed consent must be obtained prior to any study-related procedures. Assessments performed as part of standard-of-care that fall within the screening window (28 days prior to randomization) but before informed consent is obtained may be used for screening and need not be repeated for enrollment eligibility. Randomization must occur within 3 days of initiating study treatment.

Part 2

Patients will undergo a screening evaluation to determine their eligibility within 28 days prior to randomization (Table 4). PD-L1 expression in a tumor tissue sample (archival tissue, if ≤5 months old, or recently obtained on-study biopsy collected during screening; for collection

VV-RIM-00102568-2.0 Approved - 16 Apr 2020 GMT-5:00

instructions, refer to the laboratory manual) will be assessed using a PD-L1 assay by a central laboratory (Section 8.2.5). The use of the PD-L1 assays for decisions regarding treatment with cemiplimab is considered investigational. Patients will be stratified based on the PD-L1 expression of their tumors. All patients will continue in screening. Enrollment of patients with squamous NSCLC will be capped at 50%. At least 30%, but no more than 40% of patients enrolled must have tumors that express PD-L1 in ≥50% of tumor cells (PD-L1 <50% is capped at 70%). Enrollment of patients whose tumors express PD-L1 in <1% of tumor cells will be capped at 30%.

Tumor tissue samples will also be tested for EGFR mutations and ALK translocations as well as for ROS1 fusions, unless this testing has already been performed and the results are available. Patients whose tumors are positive for any of these mutations/fusions (by testing at screening or by previous results) will not be eligible for the study.

Baseline radiographic tumor assessments should also be performed within 28 days prior to randomization (Table 4). These assessments will not be reviewed by the IRC for eligibility assessment.

Informed consent must be obtained prior to any study-related procedures. Assessments performed as part of standard-of-care that fall within the screening window (28 days prior to randomization) but before informed consent is obtained may be used for screening and need not be repeated for enrollment eligibility. Randomization must occur within 3 days of initiating study treatment.

5.1.2. Treatment Period

Part 1

Patients will be enrolled in Part 1 until Part 2 is opened. It is estimated that over 200 but no more than 360 patients will be randomized. Eligible patients will be randomized 1:1:1 to Treatment Arm A, Treatment Arm B, or Treatment Arm C.

Part 2

A total of approximately 450 patients will be randomized. Eligible patients will be randomized 2:1 to Treatment Arm B or Treatment Arm A.

Treatment Period: Treatment Arm A (Part 1)

- Treatment Arm A: platinum-based doublet chemotherapy Q3W for 4 cycles (followed by mandatory pemetrexed maintenance for those patients initially assigned to receive a pemetrexed-containing regimen)

Patients randomized to Treatment Arm A will receive a platinum-based doublet chemotherapy Q3W for 4 cycles (depending on patient tolerability and disease assessment) or until RECIST 1.1-defined progressive disease or early treatment discontinuation for another reason, including unacceptable toxicity, withdrawal of consent, death, or initiation of another anti-cancer treatment.

Following 4 cycles of chemotherapy, for patients with non-squamous NSCLC for whom the investigator chooses a pemetrexed-containing doublet, pemetrexed maintenance (according to

local prescribing information and practice guidelines) will be mandatory. Patients will enter the follow-up period after the last cycle of chemotherapy or pemetrexed maintenance therapy.

Treatment Period: Treatment Arm B (Part 1)

- Treatment Arm B: cemiplimab 350 mg Q3W for 108 weeks plus platinum-based doublet chemotherapy Q3W for 4 cycles (cemiplimab/chemo-f)

Patients randomized to Treatment Arm B will receive the following treatments in combination:

- Cemiplimab 350 mg as an IV infusion on day 1 of every treatment cycle (Q3W) for 108 weeks or until RECIST 1.1-defined progressive disease or early treatment discontinuation for another reason, including unacceptable toxicity, withdrawal of consent, death, or initiation of another anti-cancer treatment, or in specific instances of confirmed CR or PR
- Platinum-based doublet chemotherapy Q3W for 4 cycles (depending on patient tolerability and disease assessment)
  - The choice of platinum-based doublet chemotherapy will be at the discretion of the investigator from 1 of the options listed in Section 7.1.3 and is to be decided and documented prior to randomization.
  - Patients for whom the investigator chooses a pemetrexed-containing doublet, pemetrexed maintenance (according to local prescribing information and practice guidelines) will be mandatory.

Treatment Period: Treatment Arm C (Part 1)

- Treatment Arm C: cemiplimab 350 mg Q3W for 108 weeks plus platinum-based doublet chemotherapy for 2 cycles and ipilimumab 50 mg Q6W for up to 4 doses (cemiplimab/chemo-l/ipi)

Patients randomized to Treatment Arm C will receive the following treatments in combination:

- Cemiplimab 350 mg as an IV infusion on day 1 of every treatment cycle (Q3W) for 108 weeks or until RECIST 1.1-defined progressive disease or early treatment discontinuation for another reason, including unacceptable toxicity, withdrawal of consent, death, or initiation of another anti-cancer treatment, or in specific instances of confirmed CR or PR
- Platinum-based doublet chemotherapy Q3W for 2 cycles (depending on patient tolerability and disease assessment)
  - The choice of platinum-based doublet chemotherapy will be at the discretion of the investigator from one of the options listed in Section 7.1.3 and is to be decided and documented prior to randomization.
  - Patients in this arm will <u>not be</u> permitted to begin pemetrexed maintenance.
- Ipilimumab 50 mg fixed dose administered IV on day 1 of every other treatment cycle (ie, every 42 days or Q6W) for up to 4 doses.

VV-RIM-00102568-2.0 Approved - 16 Apr 2020 GMT-5:00

Treatment Period: Treatment Arms A and B (Part 2)

- Cemiplimab 350 mg or placebo Q3W for 108 weeks plus platinum-based doublet chemotherapy Q3W for 4 cycles (cemiplimab/chemo-f or placebo/chemo-f)

Patients randomized to Treatment Arms A or B will receive the following treatments in combination:

- Cemiplimab 350 mg or placebo as an IV infusion on day 1 of every treatment cycle (Q3W) for 108 weeks or until RECIST 1.1-defined progressive disease or early treatment discontinuation for another reason, including unacceptable toxicity, withdrawal of consent, or death
- Platinum-based doublet chemotherapy Q3W for 4 cycles (depending on patient tolerability and disease assessment)
  - The choice of platinum-based doublet chemotherapy will be at the discretion of the investigator from 1 of the options listed in Section 7.1.3 and is to be decided and documented prior to randomization.
  - Patients for whom the investigator chooses a pemetrexed-containing doublet, pemetrexed maintenance (according to local prescribing information and practice guidelines) will be mandatory.

Treatment Period: All Treatment Arms

Randomization will be stratified by histology (non-squamous versus squamous) and levels of PD-L1 expression (Part 1: <1% versus 1% to 24% versus 25% to <50%; Part 2: <1% versus 1% to 49% versus ≥50%).

Treatment should begin within 3 days of randomization. Details of the treatment regimens are provided in Section 7.1.

For the purposes of this study, a treatment cycle will be defined as 21 days or 3 weeks.

Laboratory results for safety assessments must be available prior to dosing on day 1 of each dosing cycle (Table 5).

Details of the treatment regimens are provided in Section 7.1.1, and details of dose modifications and study drug permanent and temporary discontinuation criteria are discussed in Section 7.3.

Radiographic tumor assessments will be obtained Q9W beginning at week 9 (day 63 ± 5 days) during year 1 and Q12W beginning at week 55 (first radiographic tumor assessment in year 2 performed at end of week 54) during year 2, until IRC-assessed RECIST 1.1-defined progressive disease, withdrawal of consent, death, or initiation of another anti-cancer treatment. See Section 8.2.2.1 for detailed timing of radiographic tumor assessments. Patients who discontinue for reasons other than progression who are not attending treatment visits may have radiographic tumor assessments between Q9W and Q12W until RECIST 1.1-defined progressive disease, withdrawal of consent, death, or initiation of another anti-cancer treatment.

Patients in Part 2 of the study will be unblinded to treatment at the time of progressive disease defined by RECIST1.1 criteria or discontinuation from the study.

Progressive disease will be defined using RECIST 1.1 criteria (Appendix 2). Investigators and the blinded IRC (Section 5.3.1) will assess response to therapy using RECIST 1.1 criteria. RECIST 1.1-defined progressive disease determined by the investigator will be used for clinical management of the patient. RECIST 1.1-based tumor burden assessments by the blinded IRC will be used for evaluation of efficacy endpoints.

Patients who experience RECIST 1.1-defined progressive disease on anti-PD-1 antibody therapy may continue treatment if the investigator judges the patient to be experiencing clinical benefit and if the patient has not completed the 108-week treatment period (Section 7.8). Alternatively, these patients may opt to initiate a new anti-cancer treatment. If a patient continues treatment beyond the initial determination of progressive disease, study assessments should continue as per Table 5. If on the next scheduled radiographic tumor assessment further progressive disease is confirmed (Section 7.8), therapy will be discontinued. Further progression will be defined as an additional 10% increase in tumor burden from the time of initial progressive disease.

Safety will be assessed through the occurrence of AEs, recording of concomitant medications, vital sign evaluation, physical examination, ECOG performance status, and laboratory analyses (Table 5).

To assess disease-related symptoms, patients will be asked to complete QOL questionnaires at time points specified in Table 5.

Details of the treatment regimens are provided in Section 7.1, and details of dose modifications and study drug permanent and temporary discontinuation criteria are discussed in Section 7.3.

Blood samples will be collected from patients in Treatment Arms B and C (Part 1) and both Treatment Arms (Part 2) to measure serum concentrations of cemiplimab and cemiplimab ADA titers. Blood samples will be collected to measure biomarkers associated with clinical response to cemiplimab including cytokines, circulating nucleic acids, and other potential biomarkers of interest (Table 5).

In Part 1, after at least 6 months (24 weeks) of treatment, a patient with confirmed CR may choose to stop cemiplimab treatment early and be followed for the duration of the study. A patient with a PR that has stabilized after 6 months and is no longer changing after 3 successive tumor assessments may also choose to stop cemiplimab treatment early and be followed for the duration of the study. In Part 2, since patients and the study team will be blinded, this is not an option.

5.1.3. Follow-Up Period

Patients who discontinue study treatment due to progressive disease should return to the clinic 14 to 30 days after the last study treatment to complete the end of study assessments (follow-up visit 1).

Patients who discontinue study treatment for a reason other than progressive disease should return to the clinic 14 to 30 days (±7 days) after the last cycle visit for follow-up visit 1 and then continue with follow-up visit 2 through follow-up visit 7.

Survival data will then be collected by phone or at an office visit every 3 months or at the time of pre-planned interim and final analyses, until death, loss to follow-up, or withdrawal of study consent.

Follow-up Period: Treatment Arm A (Part 1)

- Patients in Treatment Arm A will enter follow-up after completion of the last dose of chemotherapy (completion of 4 cycles) or completion of pemetrexed maintenance therapy.
- Patients who do not start pemetrexed maintenance and enter follow-up after 4 cycles of chemotherapy without RECIST 1.1-defined progressive disease will continue to have radiographic tumor assessments according to the same schedule as the other treatment arms, as outlined in Section 8.2.2.1.
- Patients that opt to be treated with other anti-cancer treatments will be expected to complete all follow-up assessments as specified in Table 7 .
- *Optional Cemiplimab Monotherapy (Part 1 only):* Patients who experience disease progression in Treatment Arm A will be offered the option to receive cemiplimab 350 mg Q3W for up to 108 weeks, provided they meet specific criteria (Section 7.10). Safety and efficacy assessments for these patients while receiving cemiplimab will be performed per Table 5. Patients on Treatment Arm A who receive optional cemiplimab 350 mg Q3W up to 108 weeks or discontinue from the treatment will enter follow-up. Follow-up assessments for these patients will be performed as specified in Table 7.

Follow-up Period: Treatment Arms B and C (Part 1)

- Patients in Treatment Arms B and C will enter the follow-up period after completion of the 108-week treatment period, at the time of RECIST 1.1-defined progressive disease, or when the decision is made to discontinue cemiplimab treatment.
- Patients who completed the treatment period without RECIST 1.1-defined progressive disease or who discontinued study treatment early for reasons other than RECIST 1.1-defined progressive disease should continue to have radiographic tumor assessments Q12W.
- Patients who discontinued cemiplimab treatment early (but after at least 6 months [24 weeks] of treatment) due to CR, PR and entered follow-up at that time who then have RECIST 1.1-defined progressive disease while in follow-up may be offered the option of retreatment with the original treatment for an additional 108 weeks. Study assessments for these patients will be performed as specified in Table 5.

Follow-up study assessments will be performed as specified in Table 7. Patients assigned to Treatment Arms B and C will have blood samples taken for PK and ADA testing as specified in Table 7

Follow-up Period: Treatment Arms A and B (Part 2)

- Patients in Treatment Arms A and B will enter the follow-up period after completion of the 108-week treatment period, at the time of RECIST 1.1-defined progressive disease, or when the decision is made to discontinue treatment.
- Patients in Part 2 of the study will be unblinded to treatment at the time of progressive disease defined by RECIST1.1 criteria or discontinuation from the study.

- Patients on Treatment Arm A who experience disease progression will enter follow up and other anticancer treatment should be considered at this time at the discretion of the investigator.
- Patients who completed the treatment period without RECIST 1.1-defined progressive disease or who discontinued study treatment early for reasons other than RECIST 1.1-defined progressive disease should continue to have radiographic tumor assessments Q12W.
- Follow-up study assessments will be performed as specified in Table 7. Patients assigned to Treatment Arms A and B will have blood samples taken for PK and ADA testing as specified in Table 7.

5.1.4. Description of Study Stopping Rules (Part 1)

Because this is the first study of the cemiplimab/chemo-l/ipi combination, safety data from the first 10 patients treated with cemiplimab/chemo-l/ipi in Treatment Arm C will be reviewed after completing 4 weeks of follow-up following the first dose of cemiplimab/chemo-l/ipi. The data will be reviewed at a meeting of the Independent Data Monitoring Committee (IDMC). If 2 or more DLTs occur in the first 10 patients treated with cemiplimab/chemo-l/ipi, enrollment for this treatment arm will be stopped temporarily. Enrollment in Treatment Arm C will be restarted only after a formal early safety review.

An additional safety review will be performed by the IDMC after the first 10 patients in the cemiplimab/ipi/chemo-l regimen have received all 4 doses of ipilimumab and have been followed for at least 6 weeks after the last dose. This analysis will include all patients who have been exposed to the combination treatment.

After this evaluation is performed, a frequency or severity of drug related adverse events causing discontinuation of treatment in ≥25% of treated patients will lead to a pause in further enrollment to Arm C pending review with the IDMC and the study steering committee.

The outcomes of these safety reviews will be a decision to do one of the following:

- Continue the study as planned after discussions with investigators and regulatory authorities
- Continue the study without the second cycle of chemotherapy
- Increase the interval for ipilimumab administration from 6 to 12 weeks in Treatment Arm C
- Discontinue Treatment Arm C

5.1.5. Dose-Limiting Toxicities (Part 1)

The DLT observation period for determination of safety is defined as 28 days starting with cycle 1 day 1, with the intent to monitor safety and tolerability of cemiplimab/chemo-l/ipi.

Any of the below outlined events occurring during the DLT observation period and considered to be at least possibly related to cemiplimab/chemo-l/ipi will qualify as a DLT.

A DLT is defined as follows:

VV-RIM-00102568-2.0 Approved - 16 Apr 2020 GMT-5:00

Non-Hematologic Toxicity

1. Grade ≥2 uveitis (considered as a potential irAE)
2. AST or ALT >5 times upper limit of normal (ULN) and/or total bilirubin >3 times ULN
3. Any grade ≥3 non-hematologic toxicity, with the exception of the following:
   a. Grade 3 nausea, vomiting, or diarrhea unless persistent (>7 days of duration) despite maximal supportive care measures, as prescribed by the treating physician
   b. Grade ≥3 laboratory abnormalities that are considered clinically insignificant and do not meet criteria for an AE
   c. Grade 3 infusion-related reactions that respond to medical management
   d. Grade 3 irAE (as defined by experience with other immunomodulatory drugs; see Section 7.3.4 for the description of common irAEs) other than uveitis that improves within 14 days to grade 2 or lower with medical management (including treatment with steroids)
   e. Any Grade 3 irAE which would result in permanent discontinuation of the cemiplimab/ipi/chemo-l (or permanent discontinuation of ipilimumab), as outlined in the "Dosing Discontinuation" subsection of Section 7.3.1 of the protocol, or which would result in permanent discontinuation of cemiplimab, as outlined in the guidelines in Appendix 3 of the protocol, should be considered a DLT. This specifically includes the following Grade 3 irAEs: pneumonitis, colitis/diarrhea, and nephritis

Hematologic Toxicity

1. Grade 4 neutropenia lasting more than 7 days
2. Grade 4 thrombocytopenia
3. Grade 3 thrombocytopenia with bleeding
4. Grade ≥3 febrile neutropenia (fever ≥38.5°C with absolute neutrophil count [ANC] <1.0 × 10^9/L) or grade ≥3 neutropenia with documented infection
5. Grade 4 anemia

The frequency, time to onset, and severity of toxicities, as well as the success of standard medical management and dosing interruptions/delays (Section 7.3.1), will be analyzed to determine if a given toxicity should be considered a DLT. Both irAEs and non-irAEs that meet the definition of a DLT will be considered to be DLTs.

In general, because there is no clinical experience with the cemiplimab/chemo-l/ipi combination, any AE that has been clearly described for other agents that block the PD-1/PD-L1 and/or CTLA-4 pathways or the combination will be treated initially as unexpected in this study. Such TEAEs will be monitored and especially considered on an ongoing basis to assess expectedness, including possible differences in event frequency or severity from that observed with other PD-1/PD-L1 and CTLA-4 blockers.

The TEAEs that appear to meet the DLT definition will be discussed between the sponsor and the investigator. The final decision of whether or not the TEAE meets the DLT definition will be based on a careful review of all relevant data and consensus between the Medical Monitor,

CONFIDENTIAL

VV-RIM-00102568-2.0 Approved - 16 Apr 2020 GMT-5:00

Clinical Development Lead, and the designated Risk Management Lead from the Pharmacovigilance and Risk Management department. The investigator may also be consulted.

Regardless of whether a patient remains on study treatment and/or continues to participate in study procedures, such an event will count as a DLT, if the event occurs during the DLT observation period.

5.1.6. Maximum Tolerated Dose (Part 1 Only)

If 2 or more of the first 10 patients from Treatment Arm C who are dosed with cemiplimab/chemo-l/ipi experience a DLT during the DLT monitoring period, the dosing interval for ipilimumab could be increased from Q6W to Q12W or the number of cycles of platinum doublet chemotherapy reduced from 2 to 1. If 2 or more DLTs occur in the first 10 patients treated in Treatment Arm C, enrollment to this treatment arm will be stopped temporarily and will be restarted only after the formal early safety review.

The MTD is defined as the dose level immediately below the level at which dosing is stopped due to the occurrence of 2 or more DLTs. If the study is not stopped due to the occurrence of a DLT, it will be considered that the MTD has not been determined.

Based on data with cemiplimab and other anti-PD-1 investigational compounds, it is possible that an MTD may not be defined in this study.

5.1.7. End of Study Definition

The end of study is defined as the last visit of the last patient.

5.2. Planned Interim Analysis

For administrative purposes, analyses will be performed for PFS and ORR per investigator assessment in Part 1 patients who were randomized by August 2019. This administrative analysis will be performed after all Part 1 patients who were randomized by August 2019 have had the opportunity for 3 tumor assessments.

An interim analysis for OS is planned for Part 1 of the study, and 2 interim analyses are planned for Part 2 of the study. Refer to Section 10.6 for details.

5.3. Study Committees

Two independent study committees will be utilized: an IRC (Section 5.3.1) and an IDMC (Section 5.3.2).

5.3.1. Independent Review Committee

A blinded IRC composed of members who are independent from the sponsor and the study investigators will review all available (de-identified) radiographic tumor assessments to determine tumor response based on RECIST 1.1 criteria. The IRC-determined tumor response will be used in the analysis of the PFS, ORR, and DOR endpoints. Details of the IRC responsibilities and procedures will be specified in the IRC charter.

5.3.2. Independent Data Monitoring Committee

An IDMC, composed of members who are independent from the sponsor and the study investigators, will monitor patient safety by conducting formal reviews of accumulated safety data that will be blinded by treatment arm; if requested, the IDMC may have access to the treatment allocation code, evaluate pre-planned interim analysis results which will be provided by an independent statistician, or any other requested data for the purposes of a risk-benefit assessment.

The IDMC will provide the sponsor with appropriate recommendations on the conduct of the clinical study to ensure the protection and safety of the patients enrolled in the study. The IDMC will also institute any measures that may be required for ensuring the integrity of the study results during the study execution.

All activities and responsibilities of the IDMC are described in the IDMC charter.

6. SELECTION, WITHDRAWAL, AND REPLACEMENT OF PATIENTS

6.1. Number of Patients Planned

Patients will be randomized at approximately 200 global sites in Part 1 and Part 2 of this study. The following countries enrolled patients only in Part 1 of the study: Austria, France, Ireland, Italy, South Korea, Lithuania, Slovakia, and the United States.

Part 1 Number of Patients Planned

Patients will be enrolled in Part 1 until Part 2 is activated at any given site. It is estimated that over 200, but no more than 360 patients will be randomized . Part 1 halted patient enrollment in August 2019 and randomized 323 by the end of August 2019.

Part 2 Number of Patients Planned

Approximately 450 patients will be randomized in Part 2 of the study. Enrollment of patients with squamous NSCLC histology will be capped at 50%.

6.2. Study Population

Patients in this study will include men and women ≥18 years of age (≥20 years of age for Japanese patients), who are diagnosed with stage IIIB, IIIC, or stage IV non-squamous or squamous NSCLC.

Part 1 is limited to patients whose tumors express PD-L1 in <50% of tumor cells (measured using a PD-L1 IHC assay). In Part 2, at least 30% but no more than 40% of patients enrolled must have tumors that express PD-L1 in ≥50% of tumor cells; enrollment of patients whose tumors express PD-L1 in <1% of tumor cells will be capped at 30% (PD-L1 <50% is capped at 70%). This approach is adopted to maintain an approximately equal distribution of patients in each PD-L1 stratum.

To be eligible for the study, patients must meet all of the inclusion criteria (Section 6.2.1) and none of the exclusion criteria (Section 6.2.2).

CONFIDENTIAL

VV-RIM-00102568-2.0 Approved - 16 Apr 2020 GMT-5:00

6.2.1. Inclusion Criteria

A patient must meet the following criteria to be eligible for inclusion in the study:

1. Men and women ≥18 years of age (≥20 years of age for Japanese patients).
2. Patients with histologically or cytologically documented squamous or non-squamous NSCLC with stage IIIB or IIIC disease who are not candidates for treatment with definitive concurrent chemoradiation, or patients with stage IV disease if they have not received prior systemic treatment for recurrent or metastatic NSCLC. The histologic diagnosis of NSCLC may be confirmed by a central laboratory.
   a. Patients who received adjuvant or neoadjuvant platinum-based doublet chemotherapy (after surgery and/or radiation therapy) and developed recurrent or metastatic disease more than 6 months after completing therapy are eligible.
3. Availability of an archival or on-study obtained formalin-fixed, paraffin-embedded tumor tissue sample.
   - Guidance on biopsy sites:

   a. Archival or fresh biopsies are acceptable
   b. If an archival biopsy is used, it has to be <5 months old
   c. The biopsy should be from a metastatic or recurrent site which has not previously been irradiated
   d. Exception: the primary lung tumor is still in place and the other metastatic sites are either not accessible (brain) or cannot be used (bone) or the biopsy would put the patient at risk
4. Part 1 only: Expression of PD-L1 in <50% of tumor cells determined by a commercially available PD-L1 IHC assay performed by the central laboratory.
5. At least 1 radiographically measurable lesion by computed tomography (CT) or magnetic resonance imaging (MRI) per RECIST 1.1 criteria (see Appendix 2). Target lesions may be located in a previously irradiated field if there is documented (radiographic) disease progression in that site.
6. ECOG performance status of ≤1.
7. Anticipated life expectancy of at least 3 months.
8. Adequate organ and bone marrow function as defined below:
   a. Hemoglobin ≥10.0 g/dL
   b. Absolute neutrophil count ≥1.5 × 10^9/L
   c. Platelet count ≥100,000/mm^3
   d. Glomerular filtration rate (GFR) >30 mL/min/1.73m^2
   e. Total bilirubin ≤1.5 × upper limit of normal (ULN) (if liver metastases ≤3 × ULN), with the exception of patients diagnosed with clinically confirmed Gilbert's syndrome
   f. Aspartate aminotransferase (AST) and alanine aminotransferase (ALT) ≤3 × ULN or ≤5 × ULN, if liver metastases
   g. Alkaline phosphatase ≤2.5 × ULN (or ≤5.0 × ULN, if liver or bone metastases)

   h. Not meeting criteria for Hy's law (ALT >3 × ULN and bilirubin >2 × ULN).

9. Willing and able to comply with clinic visits and study-related procedures.
10. Provide signed informed consent.
11. Able to understand and complete study-related questionnaires.

6.2.2. Exclusion Criteria

A patient who meets any of the following criteria will be excluded from the study:

1. Part 1 only: Patients who have never smoked, defined as smoking ≤100 cigarettes in a lifetime.
2. Active or untreated brain metastases or spinal cord compression. Patients are eligible if central nervous system (CNS) metastases are adequately treated and patients have neurologically returned to baseline (except for residual signs or symptoms related to the CNS treatment) for at least 2 weeks prior to enrollment. Patients must be off (immunosuppressive doses of) corticosteroid therapy (see exclusion criteria 7 for details on timing of discontinuation of steroids).
3. Patients with tumors tested positive for EGFR gene mutations, ALK gene translocations, or ROS1 fusions. All patients will have tumor evaluated for EGFR mutations, ALK rearrangement, and ROS1 fusions confirmed by a central laboratory.
4. Encephalitis, meningitis, or uncontrolled seizures in the year prior to enrollment.
5. History of interstitial lung disease (eg, idiopathic pulmonary fibrosis or organizing pneumonia), of active, noninfectious pneumonitis that required immune-suppressive doses of glucocorticoids to assist with management, or of pneumonitis within the last 5 years. A history of radiation pneumonitis in the radiation field is permitted as long as pneumonitis resolved ≥6 months prior to enrollment.
6. Ongoing or recent evidence of significant autoimmune disease that required treatment with systemic immunosuppressive treatments, which may suggest risk of immune-related treatment-emergent adverse events (irTEAEs). The following are not exclusionary: vitiligo, childhood asthma that has resolved, residual hypothyroidism that required only hormone replacement, or psoriasis that does not require systemic treatment.
7. Patients with a condition requiring corticosteroid therapy (>10 mg prednisone/day or equivalent) within 14 days of randomization. Physiologic replacement doses are allowed even if they are >10 mg of prednisone/day or equivalent, as long as they are not being administered for immunosuppressive intent. Inhaled or topical steroids are permitted, provided that they are not for treatment of an autoimmune disorder.
8. Exclusion criterion removed
9. Another malignancy that is progressing or requires treatment, with the exception of nonmelanomatous skin cancer that has undergone potentially curative therapy, in situ cervical carcinoma, or any other localized tumor that has been treated, and the patient is deemed to be in complete remission for at least 2 years prior to enrollment, and no additional therapy is required during the study period.

10. Known active hepatitis B (known positive result) or known hepatitis C (known positive result) and known quantitative hepatitis C virus (HCV) RNA results greater than the lower limits of detection of the assay). Uncontrolled infection with human immunodeficiency virus (HIV), hepatitis B virus (HBV) or HCV infection; or diagnosis of immunodeficiency.

    Exceptions:

    a. Patients with HIV who have controlled infection (undetectable viral load and CD4 count above 350 either spontaneously or on a stable antiviral regimen) are permitted.
    b. Patients with HBV (hepatitis B surface antigen positive) who have controlled infection (serum hepatitis B virus DNA PCR that is below the limit of detection AND receiving anti-viral therapy for hepatitis B) are permitted
    c. Patients who are HCV antibody positive (HCV Ab +) who have controlled infection (undetectable HCV RNA by PCR either spontaneously or in response to a successful prior course of anti-HCV therapy) are permitted.

11. Active infection requiring systemic therapy within 14 days prior to randomization.

12. Prior therapy with anti-PD-1 or anti-PD-L1. Prior exposure to other immunomodulatory or vaccine therapies such as anti-CTLA-4 antibodies is permitted, but the last dose of such an antibody should have been at least 6 months prior to the first dose of study drug.

13. Treatment-related immune-mediated AEs from immune-modulatory agents (including but not limited to anti-PD1/PD-L1 monoclonal antibodies, anti-CTLA4 monoclonal antibodies, and phosphatidylinositide 3-kinase inhibitors) that have not resolved to baseline at least 3 months prior to initiation of treatment with study therapy. Patients are excluded from treatment with cemiplimab if they experienced immune-mediated AEs related to prior treatment with a blocker of the PD-1/PD-L1 pathway that were grade 3 or 4 in severity and/or required discontinuation of the agent, regardless of time of occurrence.

14. Receipt of an investigational drug or device within 30 days of enrollment or within 5 half-lives of the investigational drug or therapy being studied (whichever is longer).

15. Receipt of a live vaccine within 30 days of planned start of study medication.

16. Major surgery or significant traumatic injury within 4 weeks prior to first dose.

17. Documented allergic or acute hypersensitivity reaction attributed to antibody treatments in general or to agents specifically used in the study.

18. Known psychiatric or substance abuse disorder that would interfere with participation with and/or the requirements of the study, including current use of any illicit drugs.

19. Pregnant or breastfeeding women.

20. Sexually active men and women of childbearing potential* who are unwilling to practice highly effective contraception prior to the initial dose/start of the first treatment, during the study, and for at least 6 months after the last dose. Highly effective contraceptive measures include stable use of combined (estrogen and progestogen containing) hormonal contraception (oral, intravaginal, transdermal) or progestogen-only hormonal contraception (oral, injectable, implantable) associated with inhibition of ovulation initiated 2 or more menstrual cycles prior to screening; intrauterine device (IUD);

intrauterine hormone-releasing system (IUS); bilateral tubal ligation; vasectomized partner; and or sexual abstinence†, ‡.

* Postmenopausal women must be amenorrheic for at least 12 months in order not to be considered of childbearing potential. Pregnancy testing and contraception are not required for women with documented hysterectomy or tubal ligation.

† Sexual abstinence is considered a highly effective method only if defined as refraining from heterosexual intercourse during the entire period of risk associated with the study treatments.

‡ Periodic abstinence (calendar, symptothermal, post-ovulation methods), withdrawal (coitus interruptus), spermicides only, and lactational amenorrhea method (LAM) are not acceptable methods of contraception. Female condom and male condom should not be used together.

Sexually active men and their partners must use highly effective contraception as described above. Contraception is not required for men with documented vasectomy.

21. Patients who are committed to an institution by virtue of an order issued either by the judicial or the administrative authorities will be excluded from this study.
22. Member of the clinical site study team and/or his/her immediate family, unless prior approval granted by the Sponsor.
23. Active or latent tuberculosis. Latency should be confirmed by purified protein derivative (PPD)/QuantiFERON testing according to local guidelines in high-risk individuals at the discretion of the investigator.
24. History of previous organ transplant, including stem cell allograft.
25. Meeting the comparator products contraindicated criteria as listed in local labeling. Investigators should review the current label in their local database.

6.3. Premature Withdrawal from the Study

A patient has the right to withdraw from the study at any time, for any reason, and without repercussion.

The investigator and/or sponsor have the right to withdraw a patient from the study if it is no longer in the interest of the patient to continue in the study, or if the patient's continuation in the study places the scientific outcome of the study at risk (eg, if a patient does not or cannot follow study procedures). An excessive rate of withdrawals would render the study uninterpretable; therefore, unnecessary withdrawal of patients should be avoided.

Patients who are withdrawn prematurely from the study will be asked to complete study assessments, as described in Section 8.1.2.

Rules for discontinuation of study treatment (permanent or temporary) are discussed in Section 7.3.7.

VV-RIM-00102568-2.0 Approved - 16 Apr 2020 GMT-5:00

6.4. Replacement of Patients

Patients prematurely discontinued from the study or study treatment will not be replaced.

7. STUDY TREATMENTS

7.1. Investigational and Reference Treatments

Patients will be randomized to receive one of the following treatment regimens:

Part 1:

- Treatment Arm A: platinum-based doublet chemotherapy Q3W for 4 cycles as an IV infusion (followed by pemetrexed maintenance for those patients initially assigned to receive a pemetrexed-containing regimen)
- Treatment Arm B: cemiplimab 350 mg Q3W for 108 weeks as an IV infusion and platinum-based doublet chemotherapy Q3W for 4 cycles as an IV infusion (cemiplimab/chemo-f) (followed by pemetrexed maintenance for those patients initially assigned to receive a pemetrexed-containing regimen)
- Treatment Arm C: cemiplimab 350 mg Q3W for 108 weeks as an IV infusion platinum-based doublet chemotherapy Q3W for 2 cycles and ipilimumab 50 mg Q6W for up to 4 doses as an IV infusion (cemiplimab/chemo-l/ipi)

Part 2:

- Treatment Arm A: placebo Q3W for 108 weeks as an IV infusion, and platinum-based doublet chemotherapy Q3W for 4 cycles as an IV infusion (followed by pemetrexed maintenance for those patients initially assigned to receive a pemetrexed-containing regimen) based on randomization.
- Treatment Arm B: cemiplimab 350 mg Q3W for 108 weeks as an IV infusion, and platinum based doublet chemotherapy Q3W for 4 cycles as an IV infusion (followed by pemetrexed maintenance for those patients initially assigned to receive a pemetrexed-containing regimen) based on randomization.

Patients will receive their assigned treatment for the treatment period (as noted above) or until RECIST 1.1-defined progressive disease or early treatment discontinuation for another reason.

Study treatment will be administered by the investigator or other designated study personnel.

7.1.1. Cemiplimab or Placebo

Cemiplimab (REGN2810) is manufactured by Regeneron Pharmaceuticals, Inc.

Cemiplimab will be supplied as a liquid in sterile, single-use vials. Each vial will contain cemiplimab at a concentration of 50 mg/mL. See Section 7.6 for details on packaging, labeling, and storage.

Cemiplimab or placebo (Part 2 only) will be administered in an outpatient setting as a 30-minute (±10 minutes) IV infusions. Instructions on cemiplimab dose and placebo infusion preparation

are provided in the pharmacy manual. Instructions on management of acute infusion reactions are provided in Section 7.4.1.

Placebo will be prepared by the unblinded pharmacist. See Section 7.6.1 for preparation of placebo infusion.

In Part 1, cemiplimab will be administered in combination with either platinum-based doublet chemotherapy (administered Q3W for 4 cycles; cemiplimab/chemo-f) or with platinum-based doublet chemotherapy (administered Q3W for 2 cycles) and ipilimumab (administered Q6W for up to 4 doses; cemiplimab/chemo-f/ipi) and then alone for the remainder of the treatment period.

In Part 2, cemiplimab or placebo will be administered in combination with platinum-based doublet chemotherapy (administered Q3W for 4 cycles; cemiplimab/chemo-f or placebo/chemo-f, respectively).

In Part 1, when administered in combination with ipilimumab and chemotherapy, infuse chemotherapy, then cemiplimab, then ipilimumab on the same day. Use separate infusion bags and filters for each infusion.

In Part 1, when administered in combination with ipilimumab, infuse cemiplimab first followed by ipilimumab on the same day. Use separate infusion bags and filters for each infusion.

When administered in combination with platinum-based doublet chemotherapy agents, the chemotherapy agents should be infused first, followed by cemiplimab, on the same day. Use separate infusion bags and filters for each infusion.

7.1.2. Ipilimumab (Part 1 Only)

Ipilimumab should be procured by the study sites as local commercial products in some countries and where allowed by local regulations; for other countries, Regeneron may provide the ipilimumab to the study sites. See Section 7.6 for details on packaging, handling, and storage.

Ipilimumab 50 mg fixed dose will be administered IV over approximately 90 minutes on day 1 every 42 days (Q6W) for up to 4 doses in combination with cemiplimab. Administration should adhere to the local prescribing information and practice guidelines.

Instructions on dose preparation are provided in the pharmacy manual. Instructions on management of acute infusion reactions are provided in Section 7.4.1.

Ipilimumab should be infused after cemiplimab on the same day. Use separate infusion bags and filters for each infusion.

7.1.3. Platinum-based Doublet Chemotherapy

Chemotherapy will be administered Q3W as outlined in Table 1 for 4 cycles or 2 cycles (Part 1, Arm C only) (depending on randomized treatment arm, patient tolerability, and disease assessment) or until RECIST 1.1-defined progressive disease or early treatment discontinuation for another reason. Chemotherapy will be administered and according to local prescribing information and practice guidelines.

The choice of chemotherapy will be one of the regimens shown in Table 1. The investigator may choose from one of these regimens provided that it is consistent with the local standard-of-care

(Table 1). Assignment of the chemotherapy choice must be made prior to randomization. Chemotherapy should be procured by the study sites as local commercial products in some countries and where allowed by local regulations; for other countries, Regeneron may provide the chemotherapy to the study sites. Preference should be given to regimens that are allowed by local regulations.

Note for patients receiving paclitaxel: Caution should be exercised when paclitaxel is concomitantly administered with known substrates (eg, midazolam, buspirone, felodipine, lovastatin, eletriptan, sildenafil, simvastatin, and triazolam), inhibitors (eg, atazanavir, clarithromycin, indinavir, itraconazole, ketoconazole, nefazodone, nelfinavir, ritonavir, saquinavir, and telithromycin), and inducers (eg, rifampin and carbamazepine) of CYP3A4).

Caution should also be exercised when paclitaxel is concomitantly administered with known substrates (eg, repaglinide and rosiglitazone), inhibitors (eg, gemfibrozil), and inducers (eg, rifampin) of CYP2C8.

Potential interactions between paclitaxel, a substrate of CYP3A4, and protease inhibitors (ritonavir, saquinavir, indinavir, and nelfinavir), which are substrates and/or inhibitors of CYP3A4, have not been evaluated in clinical trials (Paclitaxel Package Insert)

The suggested sequence of drug administration is platinum-based doublet chemotherapy agents followed by cemiplimab.

Table 1: Guidelines for Platinum-Based Doublet Chemotherapy Regimens

Option	Chemotherapy Regimen	Dosing Frequency	Maintenance Therapy
1	Paclitaxel 200 mg/m^2 IV plus carboplatin AUC of 5 or 6 mg/mL/minute IV	Day 1 every 21 days (Q3W) for 4 cycles<br>Calculate dose of carboplatin using the Calvert formula.	No maintenance therapy
2	Paclitaxel 200 mg/m^2 IV plus cisplatin 75 mg/m^2 IV	Day 1 every 21 days (Q3W) for 4 cycles	No maintenance therapy
3	Pemetrexed 500 mg/m^2 IV plus carboplatin AUC of 5 or 6 mg/mL/minute IV	Day 1 every 21 days (Q3W) for 4 cycles<br>Calculate dose of carboplatin using the Calvert formula.	**For Treatment Arms A and B only (Part 1 and Part 2):** Mandatory pemetrexed maintenance 500 mg/m^2 IV day 1 every 21 days; pemetrexed maintenance according to local prescribing information and practice guidelines.
4	Pemetrexed 500 mg/m^2 IV plus cisplatin 75 mg/m^2 IV	Day 1 every 21 days (Q3W) for 4 cycles	**For Treatment Arms A and B only (Part 1 and Part 2):** Mandatory pemetrexed maintenance 500 mg/m^2 IV day 1 every 21 days; pemetrexed maintenance according to local prescribing information and practice guidelines.

Abbreviations: AUC=area under the curve; IV=intravenous; N/A=not applicable

CONFIDENTIAL

VV-RIM-00102568-2.0 Approved - 16 Apr 2020 GMT-5:00

These are general guidelines. The dosages should be according to the local prescribing information and practice guidelines. Note that patients with GFR <50 mL/min/1.73m^2 may NOT received cisplatin-containing regimens.

7.2. Pre-treatments

Premedications should be procured by the study sites as local commercial products in some countries and where allowed by local regulations; for other countries, Regeneron may provide the premedications. Preference should be given to regimens that are allowed by local regulations.

Premedications are not required prior to the first administration of cemiplimab or placebo. Premedications will be allowed at subsequent doses depending on the need to manage any observed low grade infusion reactions (Section 7.4.1.1).

For chemotherapy, premedications should be administered in accordance with the local prescribing information and practice guidelines. In general, it is recommended that patients receive corticosteroids, diphenhydramine, and an H_2-receptor blocker prior to receipt of paclitaxel. It is recommended that patients receiving pemetrexed receive vitamin supplementation (folic acid and vitamin B12) and corticosteroids. Pretreatment with vitamin supplementation is to start within 3 days of randomization for patients with non-squamous NSCLC. Patients receiving cisplatin should be adequately hydrated prior to the infusion of therapy and receive highly effective combination antiemetic therapy.

7.3. Dose Modification and Study Treatment Discontinuation Rules

7.3.1. Cemiplimab Plus Ipilimumab and Chemotherapy Combination Therapy (Part 1 Only)

Dosing Delay Rules

Patients in Treatment Arm C who experience protocol-defined DLTs (see Section 5.1.5; either during or outside of the DLT observation period) will be required to temporarily discontinue treatment with cemiplimab/chemo-l/ipi.

In addition to DLTs, during the cemiplimab/chemo-l/ipi treatment period, administration of cemiplimab (and of ipilimumab, if an AE occurs on the day of a planned ipilimumab dosing), must be delayed due to the following AEs:

- Either febrile neutropenia or neutropenia <500 cells/mm³ for >1 week despite the use of growth factors
- Any grade ≥2 non-skin, drug-related AE, except for fatigue and laboratory abnormalities and except for AEs that require study treatment discontinuation (as listed below)
- Any grade 3 drug-related laboratory abnormality (except for lymphopenia, AST, ALT, or total bilirubin or asymptomatic lipase or amylase)
  - Grade 3 lymphopenia will not require a dose delay

- If the patient had a baseline AST, ALT, or total bilirubin level that was within normal limits, dosing should be delayed for a drug-related grade ≥2 toxicity
- If the patient had a baseline AST, ALT, or total bilirubin level that was within the grade 1 toxicity range, dosing should be delayed for a drug-related grade ≥3 toxicity
- Any grade 3 drug-related amylase or lipase abnormality that is not associated with symptoms or clinical manifestations of pancreatitis will not require a dose delay

- Any grade 3 skin drug-related AE
- Any AE, laboratory abnormality, or intercurrent illness that, in the judgment of the investigator, warrants delaying the dose of study treatment

Resumption of treatment may be at the initial dose regimen or the dosing interval for ipilimumab could be increased from Q6W to Q12W or the number of cycles of platinum-based doublet chemotherapy reduced from 2 to 1, based upon the discretion of the investigator and the sponsor. Dose modification of cemiplimab will not be permitted.

A repeat occurrence of the same DLT after resumption of treatment will require permanent discontinuation of cemiplimab/chemo-l/ipi.

Criteria for Restarting Cemiplimab Plus Ipilimumab and Chemotherapy Dosing

Patients may resume treatment with cemiplimab/chemo-l/ipi when the drug-related AE(s) resolve to grade ≤1 or baseline value, with the following exceptions:

- Patients may resume study treatment in the presence of grade 2 fatigue.
- Patients who have not experienced a grade 3 drug-related skin AE may resume study treatment in the presence of grade 2 skin toxicity.
- Patients with baseline grade 1 AST, ALT, or total bilirubin level who require dose delays for reasons other than a 2-grade shift in AST, ALT, or total bilirubin may resume study treatment in the presence of grade 2 AST, ALT, OR total bilirubin.
- Patients with AST, ALT, and/or total bilirubin values meeting discontinuation criteria should have study treatment permanently discontinued.
- Drug-related pulmonary toxicity, diarrhea, or colitis must resolve to baseline before study treatment is resumed.
- Drug-related endocrinopathies adequately controlled with only physiologic hormone replacement may resume study treatment.
- Any AE that meets the discontinuation rules below requires that the patient discontinue study treatment.

Dosing Discontinuation Rules

The following categories require permanent discontinuation of cemiplimab/chemo-l/ipi:

- Any grade 2 drug-related uveitis, eye pain, or blurred vision that does not respond to topical therapy and does not improve to grade 1 severity within the retreatment period, or that requires systemic treatment require study treatment discontinuation.

- Any grade 3 non-skin, drug-related AE lasting >7 days require study treatment discontinuation, with the following exceptions for laboratory abnormalities, drug-related uveitis, pneumonitis, bronchospasm, colitis, diarrhea, hypersensitivity reactions, infusion reactions, and endocrinopathies:
  - Grade 3 drug-related uveitis, pneumonitis, bronchospasm, colitis, diarrhea, hypersensitivity reaction, or infusion reaction of any duration require study treatment discontinuation
  - Grade 3 drug-related endocrinopathies adequately controlled with only physiologic hormone replacement do not require study treatment discontinuation
  - Grade 3 drug-related laboratory abnormalities do not require study treatment discontinuation except for the following:
    - Grade 3 drug-related thrombocytopenia >7 days or associated with bleeding requires study treatment discontinuation
    - Any drug-related liver function test abnormality that meets the following criteria requires study treatment discontinuation:
      - AST or ALT >5 to 10 × ULN for >2 weeks
      - AST or ALT >10 × ULN
      - Total bilirubin >5 × ULN
      - Concurrent AST or ALT >3 × ULN and total bilirubin >2 × ULN
  - Any grade 4 drug-related AE or laboratory abnormality requires study treatment discontinuation, except for the following events, which do not require study treatment discontinuation:
    - Grade 4 neutropenia ≤7 days
    - Grade 4 lymphopenia or leukopenia
    - Isolated grade 4 amylase or lipase abnormalities that are not associated with symptoms or clinical manifestations of pancreatitis
    - Isolated grade 4 electrolyte imbalances/abnormalities that are not associated with clinical sequelae and are corrected with supplementation/appropriate management within 72 hours of onset
    - Grade 4 drug-related endocrinopathy AEs, such as adrenal insufficiency, adrenocorticotropic hormone (ACTH) deficiency, hyperthyroidism, hypothyroidism, or glucose intolerance, that resolve or are adequately controlled with physiologic hormone replacement (corticosteroids, thyroid hormones) or glucose controlling agents, as applicable, may not require study treatment discontinuation per approval from the Medical Monitor

VV-RIM-00102568-2.0 Approved - 16 Apr 2020 GMT-5:00

- Any study treatment delay resulting in no dosing for >6 weeks (cemiplimab), >12 weeks (ipilimumab Q6W), or >18 weeks (ipilimumab Q12W, if dose was revised to Q12W due to a toxicity or another reason) requires study treatment discontinuation, with the exception of dosing delays to manage drug-related AEs, such as prolonged steroid tapers and with the exception of delays noted in the next bulleted item
- Study treatment delays resulting in no dosing for >6 weeks (cemiplimab), >12 weeks (ipilimumab Q6W), or >18 weeks (ipilimumab Q12W, if dose was revised to Q12W due to a toxicity or another reason) that occur for non-drug-related reasons are permitted and may not require study treatment discontinuation, if approved by the Medical Monitor
- Any AE, laboratory abnormality, or intercurrent illness, which, in the judgment of the investigator, presents a substantial clinical risk to the patient with continued cemiplimab or ipilimumab dosing, requires study treatment discontinuation.

If the investigator determines that an AE is definitely related to ipilimumab based on the Reference Safety Information in the Investigator's Brochure or based on local prescribing information and practice guidelines, the patient may discontinue treatment with ipilimumab and continue treatment with only cemiplimab.

Additional guidelines for discontinuation of ipilimumab are provided in Section 7.3.5.

7.3.2. Cemiplimab or Placebo as Combination Therapy with Chemotherapy

The following cemiplimab or placebo infusion hold guidelines should be followed for patients in

Part 1: Treatment Arm A opting for cemiplimab monotherapy, Treatment Arm B throughout the course of the study and for patients in Treatment Arm C following completion of ipilimumab dosing (when cemiplimab will be used alone).

Part 2: Treatment Arms A and B while patient is receiving an infusion of cemiplimab or placebo. **See Section 7.5.2 on Emergency Unblinding.**

Cemiplimab or placebo infusion may be held upon occurrence of a treatment-related AE at any time on the study. Resumption of infusion after resolution or stabilization of the condition is allowed at the discretion of the investigator and sponsor if resuming treatment is thought to be in the best interest of the patient, with the exception of the following categories:

- Patients with events that require infusion to be discontinued permanently or held for more than 84 days from the last scheduled dose
- Patients with grade ≥2 uveitis. Patients with grade 2 uveitis will generally be discontinued from infusion unless there is resolution to grade ≤1 as outlined in Appendix 3 AND discussion with and approval by the Medical Monitor. All patients with grade ≥3 uveitis will be permanently discontinued from infusion

Dose modification of cemiplimab or placebo infusion will not be permitted.

Guidelines for temporary discontinuations of infusion, including delays and interruptions, criteria for restarting, and permanent discontinuations for toxicity are outlined in Table 2 and Appendix 3.

VV-RIM-00102568-2.0 Approved - 16 Apr 2020 GMT-5:00

Table 2: Cemiplimab or Placebo Guidelines for Temporary and Permanent Discontinuations for Toxicity

Toxicity	Grade	Hold Treatment?	Restarting Criteria	Restarting Dose/Schedule	Discontinuation Criteria
**Hematological toxicity** (other than grade 3 thrombocytopenia >7 days or associated with bleeding)	1, 2, 3	No	N/A	N/A	N/A
	4	Yes	Toxicity resolves to grade 0 to 1 or baseline	Same dose and schedule	Toxicity does not resolve within 84 days of last infusion. *Permanent discontinuation should be considered for any severe or life-threatening event.*
Grade 3 thrombocytopenia >7 days or associated with bleeding	3	Yes	Toxicity resolves to grades 0 to 1 or baseline	Same dose and schedule	Toxicity does not resolve within 84 days of last infusion. *Permanent discontinuation should be considered for any severe or life-threatening event.*
**Nonhematological toxicity** *Note:* Exceptions to be treated as for grade 1 toxicity: • Grade 2 alopecia • Grade 2 fatigue • Clinically insignificant lab abnormality not meeting AE criteria	1	No	N/A	N/A	N/A
	2	Consider withholding for persistent symptoms	Toxicity resolves to grades 0 to 1 or baseline	*Clinical AE resolves within 4 weeks*: Same dose and schedule *Clinical AE does not resolve within 4 weeks:* Discontinue	Toxicity does not resolve within 84 days of last infusion.
	3	Yes	Toxicity resolves to grades 0 to 1 or baseline	Same dose and schedule	Toxicity does not resolve within 84 days of last infusion.
	4	Yes	N/A	N/A	Patient must be discontinued.

Abbreviations: AE=adverse event; irAE=immune-related adverse event; N/A=not applicable
For additional information regarding potential irAEs with a potential for irAEs, see Table 3 and Appendix 3.

Appendix 3 includes recommendations on the management of specific treatment-related AEs and when to delay and/or discontinue infusion. These guidelines are intended to be applied when the investigator determines the events to be treatment-related.

Additional reasons for infusion permanent discontinuation include the following:

- Infusion will be permanently discontinued in the event of pregnancy.
- In the event of an infusion reaction of grade 3 or greater severity during or directly following infusions, dosing should be stopped and the patient must be permanently discontinued from infusion. Infusion reactions are defined in Section 9.3.4.

VV-RIM-00102568-2.0 Approved - 16 Apr 2020 GMT-5:00

- In addition, infusion for any patient may be discontinued for other safety reasons or compliance issues at the discretion of the investigator or sponsor. A patient may choose to discontinue infusion or study participation at any time for any reason.

In Part 1 only, after at least 6 months (24 weeks) of treatment, patients in Treatment Arm C with confirmed CR may choose to stop cemiplimab treatment early and be followed for the duration of the study. A patient with a PR that has stabilized after 6 months and is no longer changing after 3 successive tumor assessments may also choose to stop cemiplimab treatment early and be followed for the duration of the study.

A patient who permanently discontinues infusion treatment should continue follow-up in the study without additional infusion treatment until RECIST 1.1-defined progressive disease, completion of all study assessments, or closure of the study (Section 6.3 and Section 8.1.2).

Guidelines for dose delays and discontinuation of chemotherapy are provided in Section 7.3.6.

7.3.3. Cemiplimab Monotherapy

Dose modification of cemiplimab monotherapy will not be permitted. For details see Section 7.3.2.

7.3.4. Immune-Related Adverse Events

Investigators must be extremely vigilant and be ready to intervene early in the management of irAEs because the onset of symptoms of irAEs (eg, pneumonitis) may be subtle. Immune-related TEAEs have been reported with cemiplimab and with other anti-PD-1 antibodies; these are considered consistent with the mechanism of action of anti-PD-1 antibodies.

An irTEAE can occur shortly after the first dose or several months after the last dose of treatment. All AEs of unknown etiology associated with drug exposure should be evaluated for a possible immune etiology. Efforts should be made to rule out neoplastic, infectious, metabolic, toxin or other etiologic causes prior to labeling an AE as an irTEAE. Suggested management guidelines are provided in Appendix 3 for certain anti-PD-1 irTEAEs including but not limited to:

- **Gastrointestinal events** (colitis, colitis microscopic, enterocolitis, enterocolitis hemorrhagic, gastrointestinal perforation, diarrhoea, stomatitis)
- **Pneumonitis events** (pneumonitis, acute interstitial pneumonitis)
- **Hepatic events** (ALT/AST increased, autoimmune hepatitis, transaminases increased)
- **Endocrine events** (autoimmune thyroiditis, blood thyroid stimulating hormone [TSH] increased, diabetic ketoacidosis, diabetes mellitus, hyperthyroidism, hypophysitis, hypopituitarism, hypothyroidism, thyroid disorder, thyroiditis, adrenal insufficiency, Type 1 diabetes mellitus)
- **Uveitis** (iritis, iridocyclitis, uveitis)
- **Renal events (**nephritis, autoimmune nephritis, tubulointerstitial nephritis)
- **Skin events** (dermatitis, dermatitis acneiform, dermatitis bullous, dermatitis exfoliative, dermatitis exfoliative generalized, exfoliative rash, pruritus, pruritus

generalized, rash, rash erythematous, rash generalized, rash macular, rash maculopapular, rash maculovesicular, rash morbilliform, rash papular, rash pruritic, rash rubelliform, rash scarlatiniform, rash vesicular, vitiligo)

- **Nervous System Events** (encephalitis, paraneoplastic encephalomyelitis, myasthenia gravis)

Based on the emerging safety profile of cemiplimab and other antibodies targeting the PD-1/PD-L1 axis (Weber 2015, Naidoo 2015), the following working case definitions are provided to help investigators distinguish irTEAEs from non-immune AEs. These case definitions pertain to the more commonly reported irTEAEs associated with PD-1 inhibition (Weber 2015, Naidoo 2015), and is not exhaustive of all possible irAEs. Clinical presentations of less common irAEs, including neurologic, musculoskeletal, cardiac, renal, and ocular events (Zimmer 2016, Hofmann 2016), should be reviewed in patients with concerning presentations.

The investigator should refer to the latest version of the Investigator's Brochure for further details and guidance. The case definitions have not been validated, and are intended only as guidance for investigators to help distinguish irTEAEs from non-immune AEs. Investigators' clinical judgment may include other factors when determining immune-relatedness. The case definitions for irAEs may evolve as clinical experience increases with cemiplimab and other antibodies targeting the PD-1/PD-L1 axis.

For Part 2, see Section 7.5.2 on Emergency Unblinding.

VV-RIM-00102568-2.0 Approved - 16 Apr 2020 GMT-5:00

Table 3: General Cemiplimab Treatment Hold Guidelines for Immune-Related Adverse Events

Severity	Withhold/Discontinue Cemiplimab Treatment?	Supportive Care
Grade 1	No action.	Provide symptomatic treatment.
Grade 2	May withhold treatment.	Consider systemic corticosteroids in addition to appropriate symptomatic treatment.
Grade 3<br>Grade 4	Withhold treatment.<br>Discontinue if unable to reduce corticosteroid dose to <10 mg per day prednisone equivalent within 12 weeks of toxicity.	For any severe (grade 3-4) irAE, if symptoms worsen or do not improve on adequate corticosteroids within 48 to 72 hours, consider adding additional immunosuppressive agents (to be selected from agents such as: infliximab, CTX, cyclosporine, and mycophenolate mofetil). Referral of the patient to a specialized unit for assessment and treatment should be considered.

Abbreviations: CTX=cyclophosphamide; irAE=immune-related adverse event

7.3.5. Ipilimumab (Part 1 Only)

Ipilimumab-related toxicities should be managed in accordance with local prescribing information and practice guidelines.

Ipilimumab should also be discontinued for the following reasons:

- Ipilimumab dosing will be permanently discontinued in the event of pregnancy.
- In the event of an infusion reaction of grade 3 or greater severity during or directly following infusions, dosing should be stopped and the patient must be permanently discontinued from ipilimumab treatment. Infusion reactions are defined in Section 9.3.4.
- Ipilimumab may be discontinued for other safety reasons or compliance issues at the discretion of the investigator or sponsor. A patient may choose to discontinue cemiplimab plus ipilimumab combination therapy or study participation at any time for any reason.
- Any reason listed in the local prescribing information and practice guidelines.

If a patient experiences a toxicity that is known to be associated only with ipilimumab therapy, ipilimumab will be discontinued but cemiplimab may be continued.

7.3.6. Platinum-Based Doublet Chemotherapy

Dose modification/reduction or temporary cessation of a given chemotherapy should be managed in accordance with the local prescribing guidelines for the specific chemotherapy agent.

Chemotherapy should be permanently discontinued for safety reasons, compliance issues, intolerance due to toxicity, or other reasons as provided by local prescribing information and practice guidelines and standard-of-care.

If a patient experiences a toxicity that is known to be associated with chemotherapy, chemotherapy treatment will be discontinued but cemiplimab treatment may be continued.

VV-RIM-00102568-2.0 Approved - 16 Apr 2020 GMT-5:00

A patient who permanently discontinues from chemotherapy should continue follow-up in the study without additional chemotherapy treatment until RECIST 1.1-defined progressive disease, completion of all study assessments, or closure of the study (see Section 6.3 and Section 8.1.2).

7.3.7. Permanent Study Drug Discontinuation

Patients who permanently discontinue from study drug and who do not withdraw from the study will be asked to return to the clinic for all remaining study visits per the visit schedule.

Patients who permanently discontinue from study drug and who opt to withdraw from the study will be asked to complete study assessments, per Section 8.1.2.

7.4. Management of Acute Reactions

For Part 2, see Section 7.5.2 on Emergency Unblinding.

7.4.1. Acute Infusion Reactions

Emergency equipment and medication for the treatment of infusion reactions must be available for immediate use. Vital signs should be closely monitored according to Table 4, Table 5, Table 6, and Table 7. All infusion reactions must be reported as AEs (as defined in Section 9.4.1) and graded using the grading scales as instructed in Section 9.5.1.

Acute infusion reactions are defined as any AE that occurs during the infusion or within 1 day after the infusion is completed. Emergency equipment and medication for the treatment of these potential adverse effects (eg, antihistamines, bronchodilators, IV saline, corticosteroids, acetaminophen, and/or epinephrine) must be available for immediate use. Infusion reactions must be graded according to the current version of the National Cancer Institute Common Terminology Criteria for Adverse Events (NCI-CTCAE) grading scale (Section 9.5.1).

In the event of an infusion reaction of grade 3 or greater severity during or directly following cemiplimab (or placebo), ipilimumab, or chemotherapy infusion, dosing must be stopped and the patient must be permanently discontinued from treatment. Infusion reactions are defined in Section 9.3.4.

Acute infusion reactions can include cytokine release syndrome, angioedema, or anaphylaxis, and differ from allergic/hypersensitive reactions, although some of the manifestations are common to both AEs. Signs and symptoms usually develop during or shortly after drug infusion and generally resolve completely within 24 hours of the completion of infusion.

Signs/symptoms may include:

- Allergic reaction/hypersensitivity (including drug fever)
- Arthralgia (joint pain)
- Bronchospasm
- Cough
- Dizziness
- Dyspnea (shortness of breath)

- Fatigue (asthenia, lethargy, malaise)
- Headache
- Hypertension
- Hypotension
- Myalgia (muscle pain)
- Nausea
- Pruritus/itching
- Rash/desquamation
- Rigors/chills
- Diaphoresis (sweating)
- Tachycardia
- Tumor pain (onset or exacerbation of tumor pain due to treatment)
- Urticaria (hives, welts, wheals)
- Vomiting

7.4.1.1. Interruption of the Infusion

The infusion should be interrupted if any of the following AEs are observed:

- Cough
- Rigors/chills
- Rash, pruritus (itching)
- Urticaria (hives, welts, wheals)
- Diaphoresis (sweating)
- Hypotension
- Dyspnea (shortness of breath)
- Vomiting
- Flushing

The reaction(s) should be treated symptomatically, and the infusion may be restarted at 50% of the original rate.

If investigators feel there is a medical need for treatment or discontinuation of the infusion other than described above, they should use clinical judgment to provide the appropriate response according to typical clinical practice.

For patients who experience infusion-related hypersensitivity reactions that are less than grade 3 and who plan to continue treatment, pre-medication will be required for retreatment.

CONFIDENTIAL

VV-RIM-00102568-2.0 Approved - 16 Apr 2020 GMT-5:00

For grade 1 symptoms (mild reaction; infusion interruption not indicated; intervention not indicated), the following prophylactic medications are recommended for future infusions: diphenhydramine 50 mg (or equivalent) and/or acetaminophen/paracetamol 325 to 1000 mg at least 30 minutes prior to subsequent cemiplimab infusions.

For grade 2 symptoms (moderate reaction that requires therapy or infusion interruption but for which symptoms resolve promptly with appropriate treatment such as antihistamines, nonsteroidal anti-inflammatory drugs, narcotics, corticosteroids, and/or IV fluids; prophylactic medications indicated ≤24 hours), the following prophylactic medications are recommended for future infusions: diphenhydramine 50 mg (or equivalent) and/or acetaminophen/paracetamol 325 to 1000 mg at least 30 minutes prior to subsequent cemiplimab infusions. If necessary, corticosteroids (up to 25 mg of hydrocortisone or equivalent) may be used.

See the local prescribing information and practice guidelines for management of infusion interruptions for ipilimumab and chemotherapy agents.

7.4.1.2. Termination of the Infusion

The infusion should be terminated and NOT restarted if any of the following AEs occur:

- Anaphylaxis
- Laryngeal/pharyngeal edema
- Severe bronchospasm
- Chest pain
- Seizure
- Severe hypotension
- Other neurological symptoms (confusion, loss of consciousness, paresthesia, paralysis, etc)
- Any other symptom or sign that, in the opinion of the investigator, warrants discontinuation of the infusion

In the event of an infusion reaction of grade 3 or greater severity during or directly following cemiplimab or ipilimumab dosing must be stopped and the patient must be permanently discontinued from treatment.

See the local prescribing information and practice guidelines for management of infusion termination for ipilimumab and chemotherapy agents.

7.5. Method of Treatment Assignment

Part 1 Method of Treatment Assignment

Each patient who signs the informed consent form (ICF) will be assigned a patient number and tracked centrally as described in the interactive voice response system (IVRS)/interactive web response system (IWRS) manual. It is estimated that over 200, but no more than 360 patients will be randomized in a 1:1:1 ratio according to a central randomization scheme provided by an IVRS/IWRS to the designated study pharmacist (or authorized designee). Patients will be

CONFIDENTIAL

VV-RIM-00102568-2.0 Approved - 16 Apr 2020 GMT-5:00

randomized after providing informed consent, after completing screening assessments, and after the investigator has verified patient eligibility. Randomization will be stratified by histology (non-squamous versus squamous) and levels of PD-L1 expression (<1% versus 1% to <25% versus 25% to <50%).

Patients will be randomized 1:1:1 to receive platinum-based doublet chemotherapy, cemiplimab/chemo-f, or cemiplimab/chemo-l/ipi.

The choice of chemotherapy will be at the discretion of the investigator and is to be decided and documented prior to randomization.

Part 2 Method of Treatment Assignment

Each patient who signs the informed consent form (ICF) will be assigned a patient number and tracked centrally as described in the interactive voice response system (IVRS)/interactive web response system (IWRS) manual. Patients will be randomized after providing informed consent, after completing screening assessments, and after the investigator has verified patient eligibility. Approximately 450 patients will be randomized in a 2:1 ratio to receive cemiplimab/chemo-f or placebo/chemo-f. Randomization will be stratified by histology (non-squamous versus squamous) and levels of PD-L1 expression (<1% versus 1% to <49% versus ≥50%).

The choice of chemotherapy will be at the discretion of the investigator and is to be decided and documented prior to randomization.

7.5.1. Blinding

Part 1 Open-Label

Treatment for Part 1 of the study is open-label. To reduce bias, endpoint assessments will be performed by an IRC blinded to treatment assignment.

Part 2 Double Blind

Treatment for Part 2 of the study will be double-blinded with the exception of an unblinded pharmacist at each site. Sites will receive open label cemiplimab, which will be blinded by the unblinded pharmacist at each site. Patients, the principal investigators, and study site personnel (apart from the unblinded investigative site pharmacist) will remain blinded to all randomization assignments throughout the study. The Regeneron Medical Director, Study Monitor, and any other Regeneron and contract research organization (CRO) personnel who are in regular contact with the study site will remain blinded to all patient randomization assignments.

Selected individuals not involved in the conduct of the study may have access to unblinded data as needed for safety review or other data review.

7.5.2. Emergency Unblinding (Part 2 Only)

- Unblinding of treatment assignment for a patient may be necessary due to a medical emergency or any other significant medical event (eg, pregnancy). If unblinding is required:
  - Only the Investigator will make the decision to unblind the treatment assignment.
  - Only the affected patients will be unblinded.

- The designated study pharmacist(s)/designee at the study site will provide the treatment assignment to the Investigator. If there is no study pharmacist, the individual at the site fulfilling that role, or the Investigator for the site will unblind the treatment assignment.
- The Investigator will notify Regeneron and/or designee as soon as possible after unblinding the treatment assignment.

Treatment assignment is not to be provided to site personnel, other than the unblinded study pharmacist (when applicable), at any time during the conduct of the study, except in the case of a true emergency. In the event that there is no study pharmacist, the individual at the site fulfilling that role will be the only unblinded member of the site personnel.

7.6. Treatment Logistics and Accountability

7.6.1. Packaging, Labeling, and Storage

Part 1 Packaging, Labeling, and Storage

In Part 1, cemiplimab, ipilimumab, and platinum-based chemotherapy are open-label.

Cemiplimab

Cemiplimab will be supplied as a liquid in sterile, single-use vials that will display the product lot number on the label. Each vial will contain cemiplimab at a concentration of 50 mg/mL. Cemiplimab will be refrigerated at the site at a temperature of 2°C to 8°C. The temperature of the storage refrigerator should be checked and recorded at least daily as prescribed in the pharmacy manual. Further storage instructions will be provided in the pharmacy manual.

A pharmacist or other qualified individual will be identified at each site to prepare cemiplimab for administration. Details on storage and preparation for drug product for IV administration will be provided in the pharmacy manual.

Platinum-Based Doublet Chemotherapy

A pharmacist or other qualified individual will be identified at each site to prepare platinum-based doublet chemotherapy for administration per local guidelines.

Ipilimumab

Instructions on storage will be provided in the pharmacy manual. A pharmacist or other qualified individual will be identified at each site to prepare ipilimumab for administration. Detailed preparation and administration instructions will be provided to the sites in the pharmacy manual.

Ipilimumab will be refrigerated at the site at a temperature of 2°C to 8°C, and refrigerator temperature will be logged daily.

Part 2 Packaging, Labeling, and Storage

In Part 2, chemotherapy agents and cemiplimab are supplied open-label; however, cemiplimab or placebo will be prepared by an unblinded pharmacist at the investigative site and administered in a blinded fashion.

VV-RIM-00102568-2.0 Approved - 16 Apr 2020 GMT-5:00

Cemiplimab

Cemiplimab will be supplied as a liquid in sterile, single-use vials that will display the product lot number on the label. Each vial will contain cemiplimab at a concentration of 50 mg/mL. Cemiplimab will be refrigerated at the site at a temperature of 2°C to 8°C. The temperature of the storage refrigerator should be checked and recorded at least daily as prescribed in the pharmacy manual. Further storage instructions will be provided in the pharmacy manual.

The Sponsor, Investigator and patient will not know whether the treatment administered contains cemiplimab or placebo. The study site's unblinded pharmacist or the individual at the site fulfilling that role will obtain each patient's study identification number and study drug assignment from IVRS/IWRS and prepare the solutions for infusion. The unblinded pharmacist will provide the site staff with ready-to-use **blinded cemiplimab or saline/dextrose infusion solutions that look identical for administration** at scheduled infusion visits. Details on preparation for drug product for IV administration will be provided in the pharmacy manual.

In Arm B, if a patient qualifies for cemiplimab monotherapy after initial 1.1-RECIST-defined progression of disease, cemiplimab will be given open-label.

Platinum-Based Doublet Chemotherapy

A pharmacist or other qualified individual will be identified at each site to prepare platinum-based doublet chemotherapy for administration, per local guidelines.

7.6.2. Supply and Disposition of Treatments

Cemiplimab

Study drug will be shipped at a temperature of 2ºC to 8ºC to the investigator or designee at regular intervals or as needed during the study. At specified time points during the study (eg, interim site monitoring visits), at the site close-out visit, and following drug reconciliation and documentation by the site monitor, all opened and unopened study drug will be destroyed or returned to the sponsor or designee.

Ipilimumab

Open-label ipilimumab will be supplied locally or may be provided by Regeneron.

Platinum-Based Doublet Chemotherapy

Open-label platinum-based doublet chemotherapy agents will be supplied locally or may be provided by Regeneron.

7.6.3. Treatment Accountability

All drug accountability records must be kept current.

The investigator must be able to account for all opened and unopened study drug. These records should contain the dates, quantity, and study medication

- Dispensed to each patient,
- Returned from each patient (if applicable), and
- Disposed of at the site or returned to the sponsor or designee.

All accountability records must be made available for inspection by the sponsor and regulatory agency inspectors; anonymized photocopies must be provided to the sponsor at the conclusion of the study.

7.6.4. Treatment Compliance

All treatments will be administered at the study site, and administration will be recorded on the eCRF. All dosing records for each patient will be kept by the site.

All drug compliance records must be kept current and made available for inspection by the sponsor and regulatory agency inspectors.

7.7. Concomitant Medications

Any treatment administered from the time of informed consent until 90 days after the last study treatment will be considered concomitant medication. This includes medications that were started before the study and are ongoing during the study, as well as any therapies started in the follow-up period to treat a study-drug-related AE. All concomitant treatments must be recorded in the study eCRF with the generic name, dose, dose unit, frequency, indication, and start/stop date, as appropriate.

7.7.1. Prohibited Medications and Procedures

While participating in this study, a patient may not receive any investigational drug or treatment for treatment of a tumor other than ipilimumab (Part 1 only), cemiplimab, or study specified chemotherapy regimens.

Treatment with idelalisib, bevacizumab, or necitumumab is not one of the protocol–defined treatment options. If the treating physician believes that treatment with one of these 3 medications is required for a patient considering enrollment to the study and study-specified treatment options are not sufficient, they should not enroll in the study.

Any other medication that is considered necessary for the patient's welfare and is not expected to interfere with the evaluation of the cemiplimab may be given at the discretion of the investigator.

7.7.2. Permitted Medications and Procedures

It is recommended that patients do not receive concomitant systemic corticosteroids such as hydrocortisone, prednisone, prednisolone, or dexamethasone at any time throughout the study, except in the case of a life-threatening emergency and/or to treat an irAE.

Physiologic replacement doses of systemic corticosteroids are permitted, even if >10 mg/day prednisone equivalents. A brief course of corticosteroids for prophylaxis (eg, contrast dye allergy) or for treatment of non-autoimmune conditions (eg, delayed-type hypersensitivity reaction caused by contact allergen) is permitted.

Treatments for bone metastases (eg, bisphosphonates, denosumab) are permitted.

Pemetrexed maintenance therapy should be given according to local prescribing information and practice guidelines.

7.8. Disease Progression While Receiving Cemiplimab

It is recognized that a minority of patients treated with immunotherapy may derive clinical benefit despite initial evidence of progressive disease. Patients treated on Arm B or Arm C (cemiplimab combination regimen) of Part 1 and Arm B of Part 2 will be permitted to continue treatment beyond initial RECIST 1.1-defined progressive disease if the investigator perceives the patient to be experiencing clinical benefit, the patient has not completed the 108-week treatment period and the patient meets the criteria listed below. Patients in Part 2 will be unblinded at the time of treatment discontinuation for initial RECIST 1.1-defined progressive disease or discontinuation from the study.

- Investigator assessed no rapid disease progression.
- Patient continues to meet all other study eligibility criteria.
- Patient is tolerant of cemiplimab and has a stable performance status.
- Treatment beyond progression will not delay an imminent intervention to prevent serious complications of disease progression.

Patients may be required to sign a separate ICF per local regulatory requirements.

Imaging should be performed within 9 weeks of the initial assessment of progressive disease to determine whether there has been a decrease in the original tumor size or continued progressive disease. In these patients, further progression will be defined as an additional 10% increase in tumor burden from the time of initial progressive disease; this includes an increase in the sum of all target lesions and/or the development of new lesions. If further progressive disease is confirmed, cemiplimab must be discontinued and other anti-cancer therapy considered, if appropriate.

If a patient continues treatment with cemiplimab beyond the initial determination of progressive disease, study assessments should continue as per Table 5.

7.9. Disease Progression While Receiving Chemotherapy Plus Placebo (Part 2)

Patients treated in Part 2 will be unblinded at the time of treatment discontinuation for initial RECIST 1.1 defined progressive disease or discontinuation from the study. Patients in Treatment Arm A will discontinue study treatment and enter follow-up. Other anti-cancer treatment should be considered at this time at the discretion of the investigator.

7.10. Optional Cemiplimab Monotherapy (Part 1 Only)

Patients treated on Treatment Arm A, who experience RECIST 1.1-defined progressive disease, will be permitted to receive optional cemiplimab 350 mg Q3W for up to 108 weeks, provided that they meet the following criteria:

- IRC confirms investigator assessment of disease progression
- Investigator assesses use of a PD-1 inhibitor as an appropriate second-line treatment

VV-RIM-00102568-2.0 Approved - 16 Apr 2020 GMT-5:00

Patients who continue to meet all other study eligibility criteria, as defined in the inclusion/exclusion criteria (Section 6.2), should continue with assessments described in Table 7 except for PK and ADA assessments.

Intolerance to chemotherapy is not a reason for optional cemiplimab monotherapy.

Note: Optional cemiplimab monotherapy for patients on Treatment Arm A is only supplied by the Sponsor for patients enrolled during Part 1 of the study.

7.11. Post-Study Treatments and Procedures

Patients will be contacted quarterly (telephone is acceptable), or at the time of pre-planned analyses, for survival status and treatment status (subsequent anticancer systemic therapy), if available, until death, loss to follow-up, or study termination by the sponsor.

8. STUDY SCHEDULE OF EVENTS AND PROCEDURES

8.1. Schedule of Events

Study assessments and procedures are presented in Table 4 for screening, in Table 5 and Table 6 for the treatment period, and in Table 7 for the follow-up period.

CONFIDENTIAL

VV-RIM-00102568-2.0 Approved - 16 Apr 2020 GMT-5:00

Table 4: Schedule of Events: Screening Visit Assessments and Procedures

Procedure	Screening Visit (within 28 days prior to randomization)	Notes
**Eligibility Assessments**		
Informed Consent	X	Informed consent must be obtained prior to any study-related procedures. Assessments performed as part of standard-of-care that fall within the screening window (28 days prior to randomization) but before informed consent is obtained may be used for screening and need not be repeated for enrollment eligibility.
Inclusion/Exclusion Criteria	X	Eligibility of the patient must be confirmed prior to randomization. See Section 6.2.
Collection of Tumor Tissue Sample for PD-L1 Assessment	X	• Samples should be collected as described in the laboratory manual. See Section 8.2.5.<br>• Samples (archival tissue, if ≤5 months old, or recently obtained on-study biopsy collected during screening) will be tested for PD-L1 by a central laboratory.<br>• Samples will also be tested for EGFR mutations, ALK translocations, and ROS1 fusions by a central laboratory, unless this testing has been performed and the test results are available from other Regeneron NSCLC immunotherapy studies.
Medical/Oncology History	X	
Demographics	X	
**Efficacy Assessments**		
Baseline Radiographic Tumor Assessment	X	• CT or MRI (or PET) should be performed within 28 days of randomization.<br>• CT or MRI of the brain with contrast (unless contraindicated) should be performed in patients with a known history of treated brain metastasis, if not performed in the prior 60 days. Additional sites of known disease (including CNS) should be imaged at screening.<br>• The same imaging modality should be used throughout the study. If PET is used at baseline, it should be used throughout the study.
Baseline Tumor Burden Assessment	X	Tumor burden assessment using RECIST 1.1 criteria
**Safety Assessments**		Assessments performed as part of standard-of-care treatment that fall within the screening window (28 days prior to randomization) but before informed consent is obtained may be used for screening, and need not be repeated for enrollment eligibility.
Complete Physical Examination	X	
ECOG Performance Status	X	

CONFIDENTIAL

VV-RIM-00102568-2.0 Approved - 16 Apr 2020 GMT-5:00

Procedure	Screening Visit (within 28 days prior to randomization)	Notes
Weight	X	
Height	X	
Vital Signs	X	• Vital signs include temperature, seated BP, heart rate, respiration rate.<br>• BP and heart rate should be measured prior to obtaining any blood samples.
12-Lead ECG	X	A 12-lead ECG should be acquired at screening and as clinically indicated thereafter, per the discretion of the investigator.
Chest X-ray	X	
Laboratory Tests:<br>CBC with differential<br>Serum Chemistry<br>PT/PTT/INR<br>TSH<br>Tuberculosis	X	• Measure free T4 if TSH is outside the normal range.<br>• TSH (and free T4 if TSH is abnormal) must be tested ≤72 hours prior to dosing ipilimumab, and the results must be reviewed prior to dosing ipilimumab.<br>• Latency should be confirmed by purified protein derivative (PPD)/QuantiFERON testing according to local guidelines in high-risk individuals at the discretion of the investigator
Serum Pregnancy Test	X	Women of childbearing potential must have a serum pregnancy test performed within the 72 hours prior to administration of the first dose of study drug.
Prior/Concomitant Medication Recording	X	
Adverse Event Recording	X	Assess using current version of NCI-CTCAE.
**Genomics Sub-study**		
Genomics Sub-Study Consent (Optional)	X	DNA consent should be obtained during the screening period but may be obtained any time throughout the study

Abbreviations: ALK=anaplastic lymphoma kinase; BP=blood pressure; CBC=complete blood count; CNS=central nervous system; CT=computerized tomography; DNA=deoxyribonucleic acid; ECG=electrocardiogram; EGFR=epidermal growth factor receptor; ECOG=Eastern Cooperative Oncology Group; MRI=magnetic resonance imaging; PD-L1=programmed cell death ligand 1; PET=positron emission tomography; PT=prothrombin time; PTT=partial thromboplastin time; INR=international normalized ratio; RECIST 1.1=Response Evaluation Criteria in Solid Tumors version 1.1; ROS1=C-ros oncogene receptor tyrosine kinase; T4= thyroxine; TSH=thyroid-stimulating hormone

Table 5: Schedule of Events: Part 1 Treatment Period Assessments and Procedures

Study Procedure	Year 1				Year 2 (Starting at Cycle 19)			Notes
	Day 1, Cycle 1	Day 1, Each Cycle (Every 21 Days ±3 Days)	Day 1, Every Other Cycle (Every 42 Days ±3 Days)	Every 3 Cycles (9 Weeks [63 Days] ±5 Days)	Day 1, Each Cycle (Every 21 Days ±3 Days)	Every 3 Cycles (9 Weeks [63 Days] ±5 Days)	Every 4 Cycles (12 Weeks ±5 Days)	
Randomization	X							Treatment should be initiated within 3 days of randomization.
**Study Treatment Administration**								
**Treatment Arm A**								
Paclitaxel 200 mg/m^2 IV plus carboplatin AUC of 5 or 6 mg/mL/min IV<br>OR<br>Paclitaxel 200 mg/m^2 IV plus cisplatin 75 mg/m^2 IV<br>OR<br>Pemetrexed 500 mg/m^2 IV plus carboplatin AUC of 5 or 6 mg/mL/min IV (non-squamous histology only)<br>OR<br>Pemetrexed 500 mg/m^2 IV plus cisplatin 75 mg/m^2 IV (non-squamous histology only)	X	X	• Record start and stop infusion times.<br>• Administer 4 cycles.<br>• Calculate dose of carboplatin using the Calvert formula.<br>• Note that patients with GFR <50 mL/min/1.73m^2 may NOT receive cisplatin-containing regimens.<br>• Pemetrexed maintenance is mandatory for Treatment Arm A; pemetrexed maintenance should be according to local prescribing information and practice guidelines.<br>• For patients on the Treatment Arm A, who experience disease progression during or after administration of chemotherapy will be offered the option to receive cemiplimab 350 mg Q3W for up to 108 weeks.					

CONFIDENTIAL

VV-RIM-00102568-2.0 Approved - 16 Apr 2020 GMT-5:00

<table>
<tr><th rowspan="2">Study Procedure</th><th colspan="4">Year 1</th><th colspan="3">Year 2 (Starting at Cycle 19)</th><th rowspan="2">Notes</th></tr>
<tr><th>Day 1, Cycle 1</th><th>Day 1, Each Cycle (Every 21 Days ±3 Days)</th><th>Day 1, Every Other Cycle (Every 42 Days ±3 Days)</th><th>Every 3 Cycles (9 Weeks [63 Days] ±5 Days)</th><th>Day 1, Each Cycle (Every 21 Days ±3 Days)</th><th>Every 3 Cycles (9 Weeks [63 Days] ±5 Days)</th><th>Every 4 Cycles (12 Weeks ±5 Days)</th></tr>
<tr><td colspan="9">Treatment Arm B</td></tr>
<tr><td>Cemiplimab 350 mg</td><td>X</td><td>X</td><td></td><td></td><td>X</td><td></td><td></td><td>• Record start and stop infusion times.
• Infuse cemiplimab after chemotherapy.</td></tr>
<tr><td>Paclitaxel 200 mg/m^2 IV plus carboplatin AUC of 5 or 6 mg/mL/min IV
OR
Paclitaxel 200 mg/m^2 IV plus cisplatin 75 mg/m^2 IV
OR
Pemetrexed 500 mg/m^2 IV plus carboplatin AUC of 5 or 6 mg/mL/min IV (non-squamous histology only)
OR
Pemetrexed 500 mg/m^2 IV plus cisplatin 75 mg/m^2 IV (non-squamous histology only)</td><td>X</td><td>X</td><td colspan="6">• Record start and stop infusion times.
• Administer 4 cycles.
• Calculate dose of carboplatin using the Calvert formula.
• Note that patients with GFR <50 mL/min/1.73m^2 may NOT receive cisplatin-containing regimens.
• Pemetrexed maintenance is mandatory for Treatment Arm B; pemetrexed maintenance should be according to local prescribing information and practice guidelines.</td></tr>
</table>

CONFIDENTIAL

VV-RIM-00102568-2.0 Approved - 16 Apr 2020 GMT-5:00

Study Procedure	Year 1				Year 2 (Starting at Cycle 19)			Notes
	Day 1, Cycle 1	Day 1, Each Cycle (Every 21 Days ±3 Days)	Day 1, Every Other Cycle (Every 42 Days ±3 Days)	Every 3 Cycles (9 Weeks [63 Days] ±5 Days)	Day 1, Each Cycle (Every 21 Days ±3 Days)	Every 3 Cycles (9 Weeks [63 Days] ±5 Days)	Every 4 Cycles (12 Weeks ±5 Days)	
**Treatment Arm C**								
Cemiplimab 350 mg	X	X			X			• Record start and stop infusion times.<br>• Infuse chemotherapy first, then cemiplimab followed by ipilimumab on the same day.
Paclitaxel 200 mg/m^2 IV plus carboplatin AUC of 5 or 6 mg/mL/min IV<br>OR<br>Paclitaxel 200 mg/m^2 IV plus cisplatin 75 mg/m^2 IV<br>OR<br>Pemetrexed 500 mg/m^2 IV plus carboplatin AUC of 5 or 6 mg/mL/min IV (non-squamous histology only)<br>OR<br>Pemetrexed 500 mg/m^2 IV plus cisplatin 75 mg/m^2 IV (non-squamous histology only)	X	X						• Record start and stop infusion times.<br>• Administer 2 cycles.<br>• Calculate dose of carboplatin using the Calvert formula.<br>• Note that patients with GFR <50 mL/min/1.73m^2 may NOT receive cisplatin-containing regimens.
Ipilimumab 50 mg	X		X					• Record start and stop infusion times.<br>• Administer Q6W for up to 4 doses.<br>• Infuse after cemiplimab on the same day.

CONFIDENTIAL

VV-RIM-00102568-2.0 Approved - 16 Apr 2020 GMT-5:00

Study Procedure	Year 1				Year 2 (Starting at Cycle 19)			Notes
	**Day 1, Cycle 1**	**Day 1, Each Cycle (Every 21 Days ±3 Days)**	**Day 1, Every Other Cycle (Every 42 Days ±3 Days)**	**Every 3 Cycles (9 Weeks [63 Days] ±5 Days)**	**Day 1, Each Cycle (Every 21 Days ±3 Days)**	**Every 3 Cycles (9 Weeks [63 Days] ±5 Days)**	**Every 4 Cycles (12 Weeks ±5 Days)**	
**Efficacy Assessments**								
Radiographic Tumor Assessment				X			X	• For schedule, see Section 8.2.2.1.<br>• Image with contrast (unless contraindicated) the chest/abdomen/pelvis and other areas being monitored.<br>• Regardless of when patients in Treatment Arm A enter the follow-up period, the radiographic tumor assessment schedule outlined in Section 8.2.2.1 should be followed, until RECIST 1.1-defined progressive disease, withdrawal of consent, death, or initiation of another anti-cancer treatment.<br>• For patients who have RECIST 1.1-defined progressive disease while receiving cemiplimab or after completion of cemiplimab treatment, imaging should be performed within 9 weeks of the original tumor progression.
Tumor Burden Assessment				X			X	Tumor burden assessment per RECIST 1.1 criteria
Quality of Life Questionnaires	X	X		X		X		Complete prior to any study procedures. Complete on day 1 of every cycle for the first 6 doses and then on day 1 of every 3 cycles.

Study Procedure	Year 1				Year 2 (Starting at Cycle 19)			Notes
	Day 1, Cycle 1	Day 1, Each Cycle (Every 21 Days ±3 Days)	Day 1, Every Other Cycle (Every 42 Days ±3 Days)	Every 3 Cycles (9 Weeks [63 Days] ±5 Days)	Day 1, Each Cycle (Every 21 Days ±3 Days)	Every 3 Cycles (9 Weeks [63 Days] ±5 Days)	Every 4 Cycles (12 Weeks ±5 Days)	
**Safety Assessments**								
Physical Examination	X	X			X			A PE may be performed ≤72 hours prior to dosing on the day 1 visit of each cycle. A complete PE is to be performed prior to the first dose. A limited PE should be performed at all other visits, but a complete PE may be performed, if indicated. PE definitions are provided in Section 8.2.3.1.
ECOG Performance Status	X	X			X			
Vital Signs (Seated Blood Pressure, Heart Rate, Respiratory Rate, Temperature)	X	X			X			At cycle 1 day 1 and on all subsequent treatment days, vital signs will be collected prior to infusion of treatment. Vital signs must also be obtained approximately 15 minutes (±10 minutes) after completion of the infusion. See Section 8.2.3.4.
Weight	X	X	X	X	X	X	X	Body weight should be collected at each treatment visit prior to dosing.
12-Lead ECG	(X)	(X)			(X)			A 12-lead ECG should be acquired as clinically indicated, per the discretion of the investigator.
Hematology (CBC With Differential)	X	X			X			• Blood samples may be collected ≤72 hours prior to dosing on the day 1 visit of each cycle.<br>• Results must be obtained/reviewed prior to dosing.<br>• Screening laboratory examinations performed within 7 days of cycle 1

CONFIDENTIAL

VV-RIM-00102568-2.0 Approved - 16 Apr 2020 GMT-5:00

Study Procedure	Year 1				Year 2 (Starting at Cycle 19)			Notes
	Day 1, Cycle 1	Day 1, Each Cycle (Every 21 Days ±3 Days)	Day 1, Every Other Cycle (Every 42 Days ±3 Days)	Every 3 Cycles (9 Weeks [63 Days] ±5 Days)	Day 1, Each Cycle (Every 21 Days ±3 Days)	Every 3 Cycles (9 Weeks [63 Days] ±5 Days)	Every 4 Cycles (12 Weeks ±5 Days)	
								day 1 do not need to be repeated for this visit, unless clinically indicated.
Serum Chemistry	X	X	X (ACTH must be tested in Treatment Arm C; only prior to ipilimumab dosing)		X			• Blood sample may be collected ≤72 hours prior to dosing on the day 1 visit of each cycle.<br>• Results must be obtained/reviewed prior to dosing.<br>• Screening laboratory examinations performed within 7 days of cycle 1 day 1 do not need to be repeated for this visit, unless clinically indicated.
Coagulation Testing	(X)	(X)			(X)			As clinically indicated
Pregnancy Testing	X	X			X			Women of childbearing potential must have a negative serum pregnancy test within 72 hours prior to study treatment administration on cycle 1 day 1 and a negative urine pregnancy test prior to study treatment administration on day 1 of each subsequent treatment cycle, or more frequently per local standard.
TSH	X		X (Arm B TSH at screening and Q9W. Arm C screening and before the 4 ipilimumab doses Q6W, then Q9W)	X (Treatment Arm B) and (Treatment Arm C after ipilimumab dosing)		X		• For **Treatment Arm B**, TSH (and free T4 if TSH is abnormal) at screening and every 9 weeks.<br>• For **Treatment C**, TSH (and free T4 if TSH is abnormal) **must** be tested ≤72 hours prior to dosing ipilimumab and the results must be reviewed prior to dosing ipilimumab.

CONFIDENTIAL

VV-RIM-00102568-2.0 Approved - 16 Apr 2020 GMT-5:00

Study Procedure	Year 1				Year 2 (Starting at Cycle 19)			Notes
	Day 1, Cycle 1	Day 1, Each Cycle (Every 21 Days ±3 Days)	Day 1, Every Other Cycle (Every 42 Days ±3 Days)	Every 3 Cycles (9 Weeks [63 Days] ±5 Days)	Day 1, Each Cycle (Every 21 Days ±3 Days)	Every 3 Cycles (9 Weeks [63 Days] ±5 Days)	Every 4 Cycles (12 Weeks ±5 Days)	
								• For all treatment arms, may be obtained again, as clinically indicated.<br>• Measure free T4 if TSH is abnormal.
Concomitant Medication Recording	X	X			X			Continuous collecting concomitant medicine information. For detail see Section 7.7
Adverse Event Recording	X	X			X			Continuous collecting AE information. Assess using current version of NCI-CTCAE. See Section 9.4.1
**PK Drug Concentration/ Anti-Drug Antibody Procedures**								For footnotes, see Section 8.1.1
**Treatment Arms B and C:** Pharmacokinetic Drug Concentration Measurements and Samples	X	X					X	Collect samples as described in Appendix 4.
**Treatment Arms B and C:** Anti-Drug Antibody Measurements and Samples	X	Collect samples as described in Appendix 4						
**Biomarker Procedures**								
Serum Biomarker Sample	X	X						Collect samples as described in Appendix 4
Plasma Biomarker Sample	X	X						Collect samples as described in Appendix 4
Genomics Sub-Study: Blood Sample for Germline DNA (Optional)	X							Collect the blood sample for DNA at day 1 of cycle 1. If consent is not obtained during screening, it can be

Study Procedure	Year 1				Year 2 (Starting at Cycle 19)			Notes
	Day 1, Cycle 1	Day 1, Each Cycle (Every 21 Days ±3 Days)	Day 1, Every Other Cycle (Every 42 Days ±3 Days)	Every 3 Cycles (9 Weeks [63 Days] ±5 Days)	Day 1, Each Cycle (Every 21 Days ±3 Days)	Every 3 Cycles (9 Weeks [63 Days] ±5 Days)	Every 4 Cycles (12 Weeks ±5 Days)	
								obtained at any other visit prior to collection of the sample.
**Tumor Biopsy**								
Tumor Biopsy (Optional)		(X)			(X)			If possible, a tumor biopsy should be collected at the time of RECIST 1.1-defined progressive disease (optional) as described in the laboratory manual.

Parentheses indicate assessments which are optional.

Abbreviations: cemiplimab/chemo-f=cemiplimab in combination with platinum-based doublet chemotherapy; cemiplimab/chemo-l/ipi=cemiplimab in combination with initial platinum-based doublet chemotherapy and ipilimumab; ACTH=adrenocorticotropic hormone; ADA=anti-drug antibody; CBC=complete blood count; AUC=area under the curve; DNA=deoxyribonucleic acid; ECG=electrocardiogram; ECOG=Eastern Cooperative Oncology Group; GFR=glomerular filtration rate; IV=intravenous; NCI-CTCAE=National Cancer Institute Common Terminology Criteria for Adverse Events; PD=progressive disease; PE=physical examination; PK=pharmacokinetic; Q3W=every 3 weeks; Q6W=every 6 weeks; Q9W=every 9 weeks; Q12W=every 12 weeks; Q18W=every 18 weeks; Q24W=every 24 weeks; RECIST 1.1=Response Evaluation Criteria in Solid Tumors version 1.1; T4=thyroxine; TSH=thyroid-stimulating hormone.

CONFIDENTIAL

VV-RIM-00102568-2.0 Approved - 16 Apr 2020 GMT-5:00

Table 6: Schedule of Events: Part 2 Treatment Period Assessments and Procedures

Study Procedure	Year 1				Year 2 (Starting at Cycle 19)			Notes
	Day 1, Cycle 1	Day 1, Each Cycle (Every 21 Days ±3 Days)	Day 1, Every Other Cycle (Every 42 Days ±3 Days)	Every 3 Cycles (9 Weeks [63 Days] ±5 Days)	Day 1, Each Cycle (Every 21 Days ±3 Days)	Every 3 Cycles (9 Weeks [63 Days] ±5 Days)	Every 4 Cycles (12 Weeks ±5 Days)	
Randomization	X							Treatment should be initiated within 3 days of randomization.
**Study Treatment Administration**								
Cemiplimab 350 mg or placebo	X	X			X			• Record start and stop infusion times.<br>• Infuse cemiplimab or placebo after chemotherapy.
Paclitaxel 200 mg/m^2 IV plus carboplatin AUC of 5 or 6 mg/mL/min IV<br>OR<br>Paclitaxel 200 mg/m^2 IV plus cisplatin 75 mg/m^2 IV<br>OR<br>Pemetrexed 500 mg/m^2 IV plus carboplatin AUC of 5 or 6 mg/mL/min IV (non-squamous histology only)<br>OR<br>Pemetrexed 500 mg/m^2 IV plus cisplatin 75 mg/m^2 IV (non-squamous histology only)	X	X	• Record start and stop infusion times.<br>• Administer 4 cycles.<br>• Calculate dose of carboplatin using the Calvert formula.<br>• Note that patients with GFR <50 mL/min/1.73m^2 may NOT receive cisplatin-containing regimens.<br>• Pemetrexed maintenance is mandatory if pemetrexed is selected as part of chemotherapy; pemetrexed maintenance should be according to local prescribing information and practice guidelines.					

CONFIDENTIAL

VV-RIM-00102568-2.0 Approved - 16 Apr 2020 GMT-5:00

Study Procedure	Year 1				Year 2 (Starting at Cycle 19)			Notes
	Day 1, Cycle 1	Day 1, Each Cycle (Every 21 Days ±3 Days)	Day 1, Every Other Cycle (Every 42 Days ±3 Days)	Every 3 Cycles (9 Weeks [63 Days] ±5 Days)	Day 1, Each Cycle (Every 21 Days ±3 Days)	Every 3 Cycles (9 Weeks [63 Days] ±5 Days)	Every 4 Cycles (12 Weeks ±5 Days)	
**Efficacy Assessments**								
Radiographic Tumor Assessment				X			X	• For schedule, see Section 8.2.2.1.<br>• Image with contrast (unless contraindicated) the chest/abdomen/pelvis and other areas being monitored.<br>• Regardless of when patients enter the follow-up period, the radiographic tumor assessment schedule outlined in Section 8.2.2.1 should be followed, until RECIST 1.1-defined progressive disease, withdrawal of consent, death, or initiation of another anti-cancer treatment.<br>• For patients who have RECIST 1.1-defined progressive disease while receiving cemiplimab or placebo or after completion of cemiplimab or placebo treatment, imaging should be performed within 9 weeks of the original tumor progression.
Tumor Burden Assessment				X			X	Tumor burden assessment per RECIST 1.1 criteria
Quality of Life Questionnaires	X	X		X		X		Complete prior to any study procedures. Complete on day 1 of every cycle for the first 6 doses and then on day 1 of every 3 cycles.

VV-RIM-00102568-2.0 Approved - 16 Apr 2020 GMT-5:00

Study Procedure	Year 1				Year 2 (Starting at Cycle 19)			Notes
	Day 1, Cycle 1	Day 1, Each Cycle (Every 21 Days ±3 Days)	Day 1, Every Other Cycle (Every 42 Days ±3 Days)	Every 3 Cycles (9 Weeks [63 Days] ±5 Days)	Day 1, Each Cycle (Every 21 Days ±3 Days)	Every 3 Cycles (9 Weeks [63 Days] ±5 Days)	Every 4 Cycles (12 Weeks ±5 Days)	
**Safety Assessments**								
Physical Examination	X	X			X			A PE may be performed ≤72 hours prior to dosing on the day 1 visit of each cycle. A complete PE is to be performed prior to the first dose. A limited PE should be performed at all other visits, but a complete PE may be performed, if indicated. PE definitions are provided in Section 8.2.3.1.
ECOG Performance Status	X	X			X			
Vital Signs (Seated Blood Pressure, Heart Rate, Respiratory Rate, Temperature)	X	X			X			At cycle 1 day 1 and on all subsequent treatment days, vital signs will be collected prior to infusion of treatment. Vital signs must also be obtained approximately 15 minutes (±10 minutes) after completion of the infusion. See Section 8.2.3.4.
Weight	X	X	X	X	X	X	X	Body weight should be collected at each treatment visit prior to dosing.
12-Lead ECG	(X)	(X)			(X)			A 12-lead ECG should be acquired as clinically indicated, per the discretion of the investigator.
Hematology (CBC With Differential)	X	X			X			• Blood samples may be collected ≤72 hours prior to dosing on the day 1 visit of each cycle.<br>• Results must be obtained/reviewed prior to dosing.<br>• Screening laboratory examinations performed within 7 days of cycle 1 day 1 do not need to be repeated for this visit, unless clinically indicated.

CONFIDENTIAL

VV-RIM-00102568-2.0 Approved - 16 Apr 2020 GMT-5:00

Study Procedure	Year 1				Year 2 (Starting at Cycle 19)			Notes
	Day 1, Cycle 1	Day 1, Each Cycle (Every 21 Days ±3 Days)	Day 1, Every Other Cycle (Every 42 Days ±3 Days)	Every 3 Cycles (9 Weeks [63 Days] ±5 Days)	Day 1, Each Cycle (Every 21 Days ±3 Days)	Every 3 Cycles (9 Weeks [63 Days] ±5 Days)	Every 4 Cycles (12 Weeks ±5 Days)	
Serum Chemistry	X	X	X (ACTH must be tested in Treatment Arm C; only prior to ipilimumab dosing)		X			• Blood sample may be collected ≤72 hours prior to dosing on the day 1 visit of each cycle.<br>• Results must be obtained/reviewed prior to dosing.<br>• Screening laboratory examinations performed within 7 days of cycle 1 day 1 do not need to be repeated for this visit, unless clinically indicated.
Coagulation Testing	(X)	(X)			(X)			As clinically indicated
Pregnancy Testing	X	X			X			Women of childbearing potential must have a negative serum pregnancy test within 72 hours prior to study treatment administration on cycle 1 day 1 and a negative urine pregnancy test prior to study treatment administration on day 1 of each subsequent treatment cycle, or more frequently per local standard.
TSH	X			X		X		• TSH (and free T4 if TSH is abnormal) at screening and every 9 weeks.<br>• May be obtained again, as clinically indicated.<br>•
Concomitant Medication Recording	X	X			X			Continuous collecting concomitant medicine information. For detail see Section 7.7

Study Procedure	Year 1				Year 2 (Starting at Cycle 19)			Notes
	Day 1, Cycle 1	Day 1, Each Cycle (Every 21 Days ±3 Days)	Day 1, Every Other Cycle (Every 42 Days ±3 Days)	Every 3 Cycles (9 Weeks [63 Days] ±5 Days)	Day 1, Each Cycle (Every 21 Days ±3 Days)	Every 3 Cycles (9 Weeks [63 Days] ±5 Days)	Every 4 Cycles (12 Weeks ±5 Days)	
Adverse Event Recording	X	X			X			Continuous collecting AE information. Assess using current version of NCI-CTCAE. See Section 9.4.1
**PK Drug Concentration/ Anti-Drug Antibody Procedures**								For footnotes, see Section 8.1.1
Pharmacokinetic Drug Concentration Measurements and Samples	X	X					X	Collect samples as described in Appendix 4.
Anti-Drug Antibody Measurements and Samples	X	Collect samples as described in Appendix 4.						
**Biomarker Procedures**								
Serum Biomarker Sample	X	X						Collect samples as described in Appendix 4
Plasma Biomarker Sample	X	X						Collect samples as described in Appendix 4
Genomics Sub-Study: Blood Sample for Germline DNA (Optional)	X							Collect the blood sample for DNA at day 1 of cycle 1. If consent is not obtained during screening, it can be obtained at any other visit prior to collection of the sample.
**Tumor Biopsy**								
Tumor Biopsy (Optional)		(X)			(X)			If possible, a tumor biopsy should be collected at the time of RECIST 1.1-defined progressive disease (optional) as described in the laboratory manual.

CONFIDENTIAL

VV-RIM-00102568-2.0 Approved - 16 Apr 2020 GMT-5:00

Parentheses indicate assessments which are optional.
Abbreviations: cemiplimab/chemo-f=cemiplimab in combination with platinum-based doublet chemotherapy; ACTH=adrenocorticotropic hormone; ADA=anti-drug antibody; CBC=complete blood count; AUC=area under the curve; DNA=deoxyribonucleic acid; ECG=electrocardiogram; ECOG=Eastern Cooperative Oncology Group; GFR=glomerular filtration rate; IV=intravenous; NCI-CTCAE=National Cancer Institute Common Terminology Criteria for Adverse Events; PD=progressive disease; PE=physical examination; PK=pharmacokinetic; Q3W=every 3 weeks; Q6W=every 6 weeks; Q9W=every 9 weeks; Q12W=every 12 weeks; Q18W=every 18 weeks; Q24W=every 24 weeks; RECIST 1.1=Response Evaluation Criteria in Solid Tumors version 1.1; T4=thyroxine; TSH=thyroid-stimulating hormone.

Table 7: Schedule of Events: Follow-Up Period Assessments and Procedures (Part 1 and Part 2)

Study Procedure	Follow-up Visit 1/End of study visit for patients who discontinued treatment due to PD	Follow-up Visits 2-7	Notes
	**14 to 30 Days (±7 days) after the last study treatment if treatment is discontinued due to PD as an end of study visit OR Last Cycle Visit + 14 to 30 Days (±7 Days) if treatment is discontinued for any other reason**	**Prior Follow-up Visit + 28 Days (± 7 Days)**	
**Efficacy Assessments**			
Radiographic Tumor Assessment		X	**Part 1**<br>• Radiographic assessments for patients in **Treatment Arms B and C** should be according to the schedule outlined in Section 8.2.2.1.<br>• Regardless of when patients in Treatment Arm A enter the follow-up period, the radiographic tumor assessment schedule outlined in Section 8.2.2.1 should be followed, until RECIST 1.1-defined progressive disease, withdrawal of consent, death, or initiation of another anti-cancer treatment.<br>**Part 2**<br>• Radiographic assessments should be according to the schedule outlined in Section 8.2.2.1
Tumor Burden Assessment		X	Tumor burden assessment using RECIST 1.1 criteria
Quality of Life Questionnaires	X		Complete prior to any study procedures.
Survival Data and Treatment Status Collection	X	X	Every 3 months, until the required number of events for the primary endpoint of the study has been reached, or prior to pre-planned interim and final analyses. May be performed by phone contact
**Safety Assessments**			
Physical Examination	X	X	A limited PE may be performed but a complete PE should be performed when clinically indicated
ECOG Performance Status	X	X	
Weight	X		

CONFIDENTIAL

VV-RIM-00102568-2.0 Approved - 16 Apr 2020 GMT-5:00

Study Procedure	Follow-up Visit 1/End of study visit for patients who discontinued treatment due to PD	Follow-up Visits 2-7	Notes
Vital Signs	X	X	• Vital signs including temperature, seated BP, RR, heart rate<br>• BP and heart rate should be measured prior to obtaining any blood samples.
Hematology (CBC With Differential)	X		Collect at follow-up visit 1 and then as clinically indicated.
Serum Chemistry	X		Collect at follow-up visit 1 and then as clinically indicated.
Coagulation Testing			Collect as clinically indicated.
Pregnancy Test (urine)	X		Women of childbearing potential only.
Concomitant Medication Recording	X	X	See Section 7.7
	**14 to 30 Days (±7 days) after the last study treatment if treatment is discontinued due to PD as an end of study visit OR Last Cycle Visit + 14 to 30 Days (±7 Days) if treatment is discontinued for any other reason**	**Prior Follow-up Visit + 28 Days (± 7 Days)**	
Adverse Event Recording	X	X	Continuous collecting AE information. Assess using current version of NCI-CTCAE. See Section 9.4.1
**PK Drug Concentration/ Anti-Drug Antibody Samples (blood)**			
**Treatment Arm A (Part 2 only) and Treatment Arms B and C:**<br>Pharmacokinetic Drug Concentration Measurements and Samples	X	X	Samples will be collected at follow-up visits as described in Appendix 4. Samples will only be collected from patients on Treatment Arm A during Part 2 of the study. No samples will be collected from patients on Treatment Arm A (Part 2) after unblinding.

Study Procedure	Follow-up Visit 1/End of study visit for patients who discontinued treatment due to PD	Follow-up Visits 2-7	Notes
**Treatment Arm A (Part 2 only) and Treatment Arms B and C:**<br>Anti-Drug Antibody Measurements and Samples	X	X	Samples will be collected at follow-up visits as described in Appendix 4. Samples will only be collected from patients on Treatment Arm A during Part 2 of the study. No samples will be collected from patients on Treatment Arm A (Part 2) after unblinding.
**Biomarker Samples**			
Serum Biomarker Sample	X		Samples will be collected at follow-up visit 1 and at the time of progressive disease.
Plasma Biomarker Sample	X		Samples will be collected at follow-up visit 1 and at the time of progressive disease.
**Tumor Biopsy**			
Tumor Biopsy (Optional)			A tumor biopsy should be collected at the time of RECIST 1.1-defined progressive disease (optional) as described in the laboratory manual.

Abbreviations: CBC=complete blood count; ECOG=Eastern Cooperative Oncology Group; NCI-CTCAE=National Cancer Institute Common Terminology Criteria for Adverse Events; PD=progressive disease; PE=physical examination; PK=pharmacokinetic; Q12W=every 12 weeks; RECIST 1.1=Response Evaluation Criteria in Solid Tumors version 1.1

8.1.1. Footnotes for the Schedule of Events Table

Footnotes for the Schedule of Events Tables are as follows:

12. Pre-dose is defined as before the start of the first cemiplimab infusion (specific to PK drug concentration and ADA samples in Appendix 4). Pre-dose samples may be collected ≤72 hours prior to day 1 dosing.
13. Pre-infusion is defined as before the start of subsequent cemiplimab infusions (specific to PK drug concentration and ADA samples in Appendix 4).

8.1.2. Early Termination Visit

Patients who withdraw from the study during the treatment period will be asked to return to the clinic to complete follow-up visit 1 study assessments (Table 7) as an early termination visit. Patients who withdraw from the study during the follow-up period will be asked to return to the clinic to complete visits of the follow-up period as indicated in Table 7.

Patients who discontinue study treatment due to progressive disease should return to the clinic 14 to 30 days after the last study treatment to complete the end of study assessments (follow-up visit 1).

Patients who discontinue study treatment for a reason other than progressive disease should return to the clinic 14 to 30 days (±7 days) after the last cycle visit for follow-up visit 1 and then continue with follow-up visit 2 through follow-up visit 7.

8.1.3. Unscheduled Visits

All attempts should be made to keep patients on the study schedule. Unscheduled visits may be necessary to repeat testing following abnormal laboratory results, for follow-up of AEs, or for any other reason, as warranted. In response to adverse event of special interests (AESIs), such as anaphylaxis or hypersensitivity, ADA samples may be collected closer to the event, based on the judgment of the investigator and/or Medical Monitor.

8.2. Study Procedures

8.2.1. Procedures Performed Only at the Screening/Baseline Visit

Informed consent must be obtained prior to any study-related procedures. Assessments performed as part of standard-of-care that fall within the screening window (28 days prior to randomization) but before informed consent is obtained may be used for screening and need not be repeated for enrollment eligibility.

The following procedures will be performed for the sole purpose of determining study eligibility or characterizing the baseline population:

- Collection of tumor tissue sample for PD-L1 assessment by the central laboratory
  - A formalin-fixed, paraffin-embedded tissue block or unstained slide of tumor tissue sample (archival tissue, if ≤5 months old, or recently obtained, on-study tumor biopsy collected at screening) must be provided. Tumor biopsies should be of sufficient size to ensure an adequate amount of tissue for analysis (excisional,

incisional, or core needle; fine needle aspirates are not acceptable). Complete instructions on the collection, processing, handling, and shipment of all samples will be provided in the laboratory manual.

  – Tumor tissue samples will also be tested for EGFR mutations and ALK translocations as well as for ROS1 fusions by a central laboratory, unless this testing has already been performed and the results are available from other Regeneron NSCLC immunotherapy studies.

- Baseline radiographic tumor assessment of the chest, abdomen, pelvis, and all other known or suspected sites of disease by CT or MRI (or positron emission tomography). The same imaging modality should be used throughout the study. If PET is used at baseline, it should be used throughout the study.
- Baseline tumor burden assessment
- Baseline weight and height
- Tuberculosis testing
- Serum pregnancy test in women of childbearing potential within 72 hours prior to administration of the first study treatment administration
- Baseline 12-Lead ECG and chest X-ray
- Sample for a genomics sub-study (optional)

For the complete list of procedures performed at screening to determine eligibility, including those that are used throughout the study (ie, not only for determining eligibility), see Table 4.

8.2.2. Efficacy Procedures

8.2.2.1. Radiographic Tumor Assessments

High-resolution CT with contrast and contrast-enhanced MRI are the preferred imaging modalities for assessing radiographic tumor response. In patients whom contrast is strictly contraindicated, non-contrast scans will suffice. The chest, abdomen, and pelvis must be imaged along with any other known or suspected sites of disease. If more than 1 imaging modality is used at screening, the most accurate imaging modality according to RECIST 1.1 should be used when recording data. The same imaging modality used at screening should be used for all subsequent assessments.

At screening, CT or MRI of the brain with contrast (unless contraindicated) should be performed in patients with a known history of treated brain metastasis, if not performed in the prior 60 days. Additional sites of known disease (including CNS) should be imaged at screening.

After the baseline tumor assessment, radiographic tumor assessments will be obtained in all patients Q9W beginning at week 9 (day 63 ±5 days) during year 1 and Q12W beginning at week 55 (first radiographic tumor assessment in year 2 performed at end of week 54) during year 2, until IRC-assessed RECIST 1.1-defined progressive disease, withdrawal of consent, death, or initiation of another anti-cancer treatment. Patients who discontinue for reasons other than progression who are not attending treatment visits may have radiographic tumor

VV-RIM-00102568-2.0 Approved - 16 Apr 2020 GMT-5:00

assessments between Q9W and Q12W until RECIST 1.1-defined progressive disease, withdrawal of consent, death, or initiation of another anti-cancer treatment.

Radiographic tumor assessments on treatment will occur at the following time points:

End of week 9 ±5 days (end of cycle 3)
End of week 18 ±5 days (end of cycle 6)
End of week 27 ±5 days (end of cycle 9)
End of week 36 ±5 days (end of cycle 12)
End of week 45 ±5 days (end of cycle 15)
End of week 54 ±5 days (end of cycle 18)
End of week 66 ±5 days (end of cycle 22)
End of week 78 ±5 days (end of cycle 26)
End of week 90 ±5 days (end of cycle 30)
End of week 102 ±5 days (end of cycle 34)

In the follow-up period, radiographic assessments for all patients who have not experienced progressive disease should be performed Q9W in year 1 and Q12W in year 2, post original treatment or until RECIST 1.1-defined progressive disease, withdrawal of consent, death, or initiation of another anti-cancer treatment.

Tumor assessments should be performed even if dosing is interrupted. Weeks are in reference to the calendar week and should not be adjusted due to dosing delays/interruptions.

Patients in Treatment Arms B and C of Part 1 and Treatment Arm B of Part 2 who are receiving treatment beyond RECIST 1.1-defined progressive disease, subsequent imaging should be performed within 9 weeks of the initial tumor assessment of progression.

For Arm A patients receiving optional cemiplimab monotherapy during Part 1 of the study, radiographic assessments should be performed Q12W.

8.2.2.2. Tumor Burden Assessments

Tumor measurements will be performed in accordance with RECIST 1.1 criteria (Eisenhauer 2009; Appendix 2) and should be done by the same investigator or radiologist for each assessment, to the extent feasible.

Investigators and the blinded IRC (Section 5.3.1) will assess response to therapy using RECIST 1.1 criteria. RECIST 1.1-defined progressive disease determined by the investigator will be used for clinical management of the patient. RECIST 1.1-based tumor burden assessments by the blinded IRC will be used for evaluation of efficacy endpoints.

CONFIDENTIAL

VV-RIM-00102568-2.0 Approved - 16 Apr 2020 GMT-5:00

8.2.2.3. Quality of Life Questionnaires

Patient-reported outcomes will be measured at the frequency indicated in Table 5, Table 6, and Table 7 using the following validated patient self-administered questionnaires: EORTC QLQ-C30 and EORTC QLQ-LC13 (Bergman 1994, Bjordal 2000). Patients will be asked to complete these questionnaires prior to any study procedures being performed at a given study visit (during the treatment and follow-up periods).

8.2.2.4. Survival Data and Treatment Status Collection

Every effort will be made to collect survival data and treatment status (subsequent anticancer systemic therapy) on all patients, including patients who withdraw from the study for any reason but have not withdrawn consent to collect survival information, as indicated in Table 7. Patients will be contacted quarterly (telephone is acceptable) or at the time of pre-planned interim or final analyses for survival status and treatment status (subsequent anticancer systemic therapy) until the required number of events for the primary endpoint of the part of the study (part 1 or part 2) on which the patient is enrolled has been reached. If the death of a patient is not reported, the date of the last patient contact in this study will be used in the determination of the patient's last known date alive.

8.2.3. Safety Procedures

8.2.3.1. Physical Examination

A complete or limited PE will be performed at the visits specified in Table 4, Table 5, Table 6, and Table 7. Care should be taken to examine and assess any abnormalities that may be present, as indicated by the patient's medical history.

Complete PE will be performed prior to the first dose on cycle 1 day 1, or in other visits if indicated, and will include examination of the skin, head, eyes, nose, throat, neck, joints, lungs, heart, pulse, abdomen (including liver and spleen), lymph nodes, and extremities, as well as a brief neurologic examination.

Limited physical examination will include, at least, examination of the lungs, heart, abdomen, and skin.

8.2.3.2. ECOG Performance Status

Eastern Cooperative Oncology Group performance status will be measured at a frequency indicated in Table 4, Table 5, and Table 6, and Table 7.

8.2.3.3. Weight and Height

Body weight measurements will be obtained at screening according to Table 4, at treatment according to Table 5 and Table 6, and at follow-up according to Table 7. Weight should be obtained with the patient wearing undergarments or very light clothing, with no shoes, and with an empty bladder. The same scale should be used throughout the study. The use of calibrated balance scales is recommended, if possible. Self-reported weights are not acceptable.

Height should be measured at screening; self-reported heights are not acceptable.

CONFIDENTIAL

VV-RIM-00102568-2.0 Approved - 16 Apr 2020 GMT-5:00

8.2.3.4. Vital Signs

Vital signs, including temperature, seated blood pressure, heart rate, and respiratory rate, will be collected at time points according to Table 4, Table 5, Table 6, and Table 7. Vital signs should be performed before blood is drawn during visits requiring blood draws.

Blood pressure should be measured in the same arm at all study visits (when feasible) and after the patient has been resting quietly in the seated position for at least 5 minutes.

At cycle 1 day 1 and on all subsequent treatment days, vital signs will be collected prior to infusion of treatment. Vital signs should also be obtained approximately 15 minutes (±10 minutes) after completion of the infusion.

8.2.3.5. 12-Lead Electrocardiogram

A standard 12-lead ECG will be performed at screening and when clinically indicated, per the discretion of the investigator, while during the active treatment period (Table 4, Table 5, and Table 6). Electrocardiograms (ECGs) should be performed before blood is drawn during visits requiring blood draws.

The patient should be relaxed and in a recumbent position for at least 5 minutes before recording an ECG. The ECG will be reviewed by the investigator or an authorized designee at the site and will be available for comparison with subsequent ECGs. The ECG tracing will be retained with the source.

Any ECG finding that is judged by the investigator as a clinically significant change (worsening) compared with the baseline value will be considered an AE, recorded, and monitored.

8.2.3.6. Laboratory Testing

Hematology, chemistry, and pregnancy testing samples will be analyzed by the site's local laboratory.

Samples for laboratory testing will be collected at time points according to according to Table 4 , Table 5, Table 6, and Table 7. Tests will include the following:

Hematology (CBC with Differential)

Hemoglobin	Differential:
Hematocrit	Neutrophils
Red blood cells	Lymphocytes
White blood cells	Monocytes
Red cell indices	Basophils
Platelet count	Eosinophils

For hematology, blood samples may be collected ≤72 hours prior to dosing on the day 1 visit of each cycle. Results must be obtained/reviewed prior to dosing.

Serum Chemistry

Sodium	Total protein, serum	Total bilirubin
Potassium	Creatinine	Magnesium
Chloride	Blood urea nitrogen [a]	Phosphorus
Bicarbonate [b]	Aspartate aminotransferase	Uric acid
Calcium	Alanine aminotransferase	Adrenocorticotropic hormone[c]
Glucose	Alkaline phosphatase	
Albumin	Lactate dehydrogenase	

[a] The urea test is acceptable instead of blood urea nitrogen at centers where this is commonly used instead of the blood urea nitrogen.
[b] The partial pressure of carbon dioxide (PCO_2) test is an acceptable test at centers where this is commonly used instead of the bicarbonate test.
[c] Adrenocorticotropic hormone (ACTH) will be included only for the 4 cycles when ipilimumab is administered and only in Treatment Arm C.

For chemistry, blood sample may be collected ≤72 hours prior to dosing on the day 1 visit of each cycle. Results must be obtained/reviewed prior to dosing. For Treatment Arm C, ACTH should be tested during the 4 cycles ipilimumab is administered.

Other Laboratory Tests

Coagulation Tests: Prothrombin time/partial thromboplastin time/international normalized ratio will be analyzed by the site's local laboratory. Testing will be performed at screening and then as clinically indicated.

Thyroid Function Tests:

Thyroid-stimulating hormone will be analyzed by the site's local laboratory.

For Treatment Arm C (Part 1), TSH will be tested at screening, before the 4 ipilimumab doses every 6 weeks then Q9W, and as clinically indicated. If TSH is outside of normal range, a free thyroxine (T4) should be measured at the investigative site's local laboratory. Thyroid function test (and free T4 if TSH is abnormal) must be tested ≤72 hours prior to dosing ipilimumab, and the results must be reviewed prior to dosing ipilimumab, and on cycles when ipilimumab is not dosed.

For Treatment Arm B (Part 1), TSH will be tested at screening, Q9W and as clinically indicated. If TSH is outside of normal range, a free T4 should be measured at the investigative site's local laboratory.

For Treatment Arm A (Part 1), TSH is tested at screening and as clinically indicated. If TSH is outside normal range, a free T4 should be measured at the investigative site's local laboratory.

For Part 2, TSH will be tested at screening, Q9W and as clinically indicated. If TSH is outside of normal range, a free T4 should be measured at the investigative site's local laboratory.

Tuberculosis Testing: Latency should be confirmed by purified protein derivative (PPD)/QuantiFERON testing according to local guidelines in high-risk individuals at the discretion of the investigator.

Pregnancy Testing

Women of childbearing potential must have a negative serum pregnancy test within the 72 hours prior to study treatment administration on cycle 1 day 1 and a negative urine pregnancy test prior to study treatment administration on day 1 of each subsequent treatment cycle, or more frequently, per local standard.

Abnormal Laboratory Values and Laboratory Adverse Events

- All laboratory values must be reviewed by the investigator or an authorized designee.
- Significantly abnormal test results that occur after start of treatment must be repeated to confirm the nature and degree of the abnormality. When necessary, appropriate ancillary investigations should be initiated. If the abnormality fails to resolve or cannot be explained by events or conditions unrelated to the study medication or its administration, the Medical Monitor must be consulted.
- The clinical significance of an abnormal test value, within the context of the disease under study, must be determined by the investigator.

Criteria for reporting laboratory values as an AE are provided in Section 9.4.5.

8.2.3.7. Concomitant Medication Recording

Concomitant medication will be collected at time points according to Table 4, Table 5, Table 6, and Table 7. See Section 7.7 for details on recording concomitant medications.

8.2.3.8. Adverse Event Recording

Adverse events will be collected at time points according to according to Table 4, Table 5, Table 6, and Table 7. See Section 9.4 for details on recording and reporting AEs.

8.2.4. Pharmacokinetic and Anti-Drug Antibody Procedures

In addition to the procedures detailed below, blood samples will also be taken to measure drug concentrations/ADA, as appropriate, in case of AESIs.

8.2.4.1. Drug Concentration Measurements and Samples

Blood samples will be collected from patients in Treatment Arms B and C in Part 1 and both Treatment Arms in Part 2 (analysis will be limited to Treatment Arm B of Part 2). Cemiplimab concentrations in the sera of patients randomized to Treatment Arm B (Parts 1 and 2) and Treatment Arm C (Part 1) will be measured using a validated enzyme-linked immunosorbent assay method at visits and time points indicated in Table 5, Table 6, Table 7, and.The actual time of each blood draw must be recorded. Pre-dose is defined as before the start of the first cemiplimab infusion. Pre-dose samples may be collected ≤72 hours prior to day 1 dosing. Pre-infusion is defined as before the start of subsequent cemiplimab infusions.

In addition, measurement of ipilimumab concentrations in serum may be considered in the future in the PK samples of patients randomized to Treatment Arm C.

Any unused samples collected for drug concentration measurements may be used for exploratory biomarker research.

VV-RIM-00102568-2.0 Approved - 16 Apr 2020 GMT-5:00

8.2.4.2. Anti-Drug Antibody Measurements and Samples

Anti-drug antibody samples for cemiplimab immunogenicity assessments will be collected from patients randomized Treatment Arms B and C in Part 1 and both Treatment Arms in Part 2 (analysis will be limited to Treatment Arm B of Part 2) prior to dosing at time points listed in Table 5, Table 6, during the follow-up period as shown in Table 7 and as indicated in Any unused samples collected for immunogenicity assessments may be used for exploratory research or to investigate unexpected AEs. If necessary, these samples may also be used for ADA assessments of ipilimumab.

8.2.5. Biomarker Procedures

For biomarker assessments, a formalin-fixed, paraffin-embedded tissue block or unstained slides of tumor tissue biopsy samples (archival tissue if ≤5 months old, or recently obtained biopsy collected during on-study (if this is medically challenging, agreement to provide an older biopsy can be obtained by agreement with the sponsor Medical Director) must be provided. Tumor tissue biopsy samples should be of sufficient size to ensure an adequate amount of tissue for analysis (excisional, incisional, or core needle; fine needle aspirates are not acceptable). Complete instructions on the collection, processing, handling, and shipment of all samples will be provided in the laboratory manual.

With the use of the collected tumor tissue samples, the following biomarker-based stratification strategies will be implemented in this study to determine study eligibility:

- Tumor tissue biopsy samples will be tested by a central laboratory for EGFR mutations, ALK translocations, as well as for ROS1 fusions for determination of study eligibility. This testing does not need to be repeated if the results are available from other Regeneron NSCLC immunotherapy studies. The results must indicate that the patient is negative for EGFR mutations, ALK translocations, and ROS1 fusions for enrollment in this study.
- A PD-L1 assay will be utilized as the clinical trial assay to assess PD-L1 expression levels in recently obtained tumor tissue samples (on-study biopsy collected at screening visit or archival tissue, if ≤5 months old) in order to qualify patients for enrollment. The <50% PD-L1 positivity cut-off will be used as an inclusion criterion for Part 1 of this study.

After completion of PD-L1 expression analysis, the remaining tumor tissue samples may be used to study the biomarkers associated with clinical response to cemiplimab including but not limited to whole exome sequencing of tumor genome and tumor mutational load.

Biomarker serum and plasma samples will be collected from all patients enrolled in this study at multiple visits and time points indicated in Table 5, Table 6, Table 7, and to study the potential pharmacodynamics or predictive biomarkers of response to cemiplimab including, but not limited to, cytokines and circulating tumor nucleic acids (refer to the laboratory manual). Samples will be collected prior to drug administration.

If possible, a tumor tissue biopsy sample should be obtained at the time of RECIST 1.1-defined progressive disease to obtain information on mechanism of resistance.

VV-RIM-00102568-2.0 Approved - 16 Apr 2020 GMT-5:00

8.2.6. Future Biomedical Research

The biomarker samples unused for study-related research, as well as unused PK and ADA samples, will be stored for up to 15 years after the final date of the database lock. The unused samples may be utilized for future biomedical research of lung carcinoma and other diseases. No additional samples will be collected for future biomedical research. After 15 years, any residual samples will be destroyed.

8.2.6.1. Genomics Sub-Study - Optional

Patients who agree to participate in the genomics sub-study will be required to sign a separate genomics sub-study ICF before collection of the samples. Patients are not required to participate in the genomics sub-study in order to enroll in the primary study. Samples for DNA extraction should be collected on day 1/baseline (predose), but may be collected at any study visit.

DNA samples for the genomics sub-study will be double-coded as defined by the International Council for Harmonisation (ICH) guideline E15. Sub-study samples will be stored for up to 15 years after the final date of the database lock and may be used for research purposes. The purpose of the genomic analyses is to identify genomic associations with clinical or biomarker response, other clinical outcome measures and possible AEs. In addition, associations between genomic variants and prognosis or progression of other diseases may also be studied. These data may be used or combined with data collected from other studies to identify and validate genomic markers related to the study drug or other diseases. Analyses may include sequence determination or single nucleotide polymorphism studies of candidate genes and surrounding genomic regions. Other methods, including whole-exome sequencing, whole-genome sequencing, and DNA copy number variation may also be performed. The list of methods may be expanded to include novel methodology that may be developed during the course of this study or sample storage period.

9. SAFETY DEFINITIONS, REPORTING, AND MONITORING

For the purposes of this section, study treatment refers to cemiplimab, platinum-based doublet chemotherapy agents and ipilimumab.

9.1. Obligations of Investigator

The investigator must promptly report to the Institutional Review Board (IRB)/Ethics Committee (EC) all unanticipated problems involving risks to patients according to local regulations.

9.2. Obligations of Sponsor

During the course of the study, the sponsor will report in an expedited manner all SAEs that are both unexpected and at least reasonably related to the study drug (suspected, unexpected, serious adverse reactions), to the health authorities, IRB/ECs as appropriate, and to the investigators.

Any AE not listed as an expected event in the Reference Safety Information section of the cemiplimab Investigator's Brochure or in the reference safety documents of the other study drugs will be considered as unexpected.

CONFIDENTIAL

VV-RIM-00102568-2.0 Approved - 16 Apr 2020 GMT-5:00

In addition, the sponsor will report all other SAEs to the health authorities, according to local regulations, if this is applicable to the country requirements.

At the completion of the study, the sponsor will report all safety observations made during the conduct of the study in the clinical study report to health authorities and IRB/ECs, as appropriate.

9.3. Definitions

9.3.1. Adverse Event

An AE is any untoward medical occurrence in a patient administered a study drug which may or may not have a causal relationship with the study drug. Therefore, an AE is any unfavorable and unintended sign (including abnormal laboratory finding), symptom, or disease which is temporally associated with the use of a study drug, whether or not considered related to the study drug.

An AE also includes any worsening (ie, any clinically significant change in frequency and/or intensity) of a pre-existing condition that is temporally associated with the use of the study drug.

Progression of underlying malignancy will not be considered an AE if it is clearly consistent with the typical progression pattern of the underlying cancer (including time course, affected organs, etc.). Clinical symptoms of progression may be reported as AEs if the symptom cannot be determined as exclusively due to the progression of the underlying malignancy, or does not fit the expected pattern of progression for the disease under study.

If there is any uncertainty about an AE being due only to progression of the underlying malignancy, it should be reported as an AE or SAE as outlined in Section 9.3.2.

9.3.2. Serious Adverse Event

An SAE is any untoward medical occurrence that at any dose:

- Results in **death** – includes all deaths, even those that appear to be completely unrelated to study drug (eg, a car accident in which a patient is a passenger).
- Is **life-threatening** – in the view of the investigator, the patient is at immediate risk of death at the time of the event. This does not include an AE that had it occurred in a more severe form, might have caused death.
- Requires in-patient **hospitalization** or **prolongation of existing hospitalization**. In-patient hospitalization is defined as admission to a hospital or an emergency room for longer than 24 hours. Prolongation of existing hospitalization is defined as a hospital stay that is longer than was originally anticipated for the event, or is prolonged due to the development of a new AE as determined by the investigator or treating physician.
- Results in persistent or significant **disability/incapacity** (substantial disruption of one's ability to conduct normal life functions).
- Is a **congenital anomaly/birth defect**.

VV-RIM-00102568-2.0 Approved - 16 Apr 2020 GMT-5:00

- Is an **important medical event** - Important medical events may not be immediately life-threatening or result in death or hospitalization, but may jeopardize the patient or may require intervention to prevent one of the other serious outcomes listed above (eg, intensive treatment in an emergency room or at home for allergic bronchospasm; blood dyscrasias or convulsions that do not result in hospitalization; or development of drug dependency or drug abuse).

Hospitalization or death due solely to manifestations consistent with typical progression of underlying malignancy will not be considered as an SAE.

Serious adverse events must be reported as directed in Section 9.4.2.

9.3.3. Adverse Events of Special Interest

An AESI (serious or non-serious) is one of scientific and medical concern specific to the sponsor's product or program, for which ongoing monitoring and rapid communication by the investigator to the sponsor can be appropriate. Such an event might warrant further investigation in order to characterize and understand it. Depending on the nature of the event, rapid communication by the study sponsor to other parties (eg, regulators) might also be warranted. All AESI, serious and non-serious, must be reported within 24 hours of identification using the same reporting process as for SAE reporting, per Section 9.4.2. Adverse events of special interest for this study include the following:

- Any AE that meets the DLT criteria (defined in Section 5.1.5)
- Grade 2 or greater infusion-related reactions
- Grade 2 or greater allergic/hypersensitivity reactions
- Grade 3 or greater irAEs
- irAEs of any grade in patients previously treated with phosphatidylinositol 3-kinase (PI3-K) inhibitor

Note: An irAE can occur shortly after the first dose or several months after the last dose of study treatment. All AEs of unknown etiology associated with cemiplimab exposure should be evaluated to determine possible immune etiology. If an irAE is suspected, efforts should be made to rule out neoplastic, infectious, metabolic, toxin, or other etiologic causes prior to labeling an AE as an irAE. Detailed guidance of management of irAEs is provided in Section 7.3.3 and Appendix 3. The recommendations in Section 7.3.3 and Appendix 3 should be seen as guidelines, and the treating physician should exercise clinical judgment based on the symptoms and condition of the individual patient. For any AE that is of a type known to be potentially immune-related (eg, rash, colitis, elevated transaminases, endocrine, or pneumonitis) but is deemed not to be an irAE by the investigator, the sponsor may request additional information.

9.3.3.1. Immune-Related Adverse Events

Detailed guidance of management of irAEs is provided in Section 7.3.3 and Appendix 3.

Note regarding irAEs: For any AE that is of a type known to be potentially immune-related (eg, rash, colitis, elevated transaminases, endocrine, or pneumonitis) but is deemed not to be an irAE by the investigator, the sponsor may request additional information.

9.3.4. Infusion Reactions

Infusion-related reactions are known to occur with protein therapeutic infusions and have been observed in cemiplimab studies. Acute infusion reactions are defined as any AEs that occur during the infusion or within 1 day after the infusion is completed. Signs and symptoms usually develop during or shortly after drug infusion and generally resolve completely within 24 hours of completion of infusion.

All infusion reactions must be reported as AEs (defined in Section 9.4.1) and graded using the grading scales as instructed in Section 9.5.1.

9.4. Recording and Reporting Adverse Events

9.4.1. Adverse Events

The investigator (or designee) will seek information on AEs at each patient contact, and record all AEs that occur from the time the informed consent is signed until 90 days after the end of study treatment. After informed consent has been obtained but prior to initiation of study treatment, only the following categories of AEs should be reported on the AE eCRF:

- SAEs
- Non-SAEs associated with a protocol-mandated intervention (eg, AEs related to an invasive procedure such as a biopsy)

Other AEs that occur prior to first treatment should be reported on the medical history eCRF.

All AEs after initiation of study treatment and until 90 days after the last study treatment, regardless of relationship to study treatment, will be reported on the AE eCRF. Additionally, any SAE or other AE of concern that the investigator believes may be related to study treatment and that occurs later than 90 days after last study treatment should be reported.

Information on follow-up for AEs is provided in Section 9.4.6. Laboratory, vital signs, or ECG abnormalities are to be recorded as AEs as outlined in Section 9.4.5.

9.4.2. Serious Adverse Events

All SAEs, regardless of assessment of causal relationship to study drug, must be reported to the sponsor (or designee) within 24 hours of becoming aware of the event.

Information not available at the time of the initial report must be documented in a follow-up report. Substantiating data such as relevant hospital or medical records and diagnostic test reports may also be requested.

In the event that the investigator is informed of an SAE that occurs more than 90 days after the last dose of study treatment, only those SAEs or other AEs of concern deemed by the investigator to be related to study treatment will be reported to the sponsor. The investigator

VV-RIM-00102568-2.0 Approved - 16 Apr 2020 GMT-5:00

should make every effort to obtain follow-up information on the outcome of any treatment-related SAE and/or until the event is considered chronic and/or stable.

9.4.3. Other Events that Require Accelerated Reporting to Sponsor

The following events also require reporting to the sponsor (or designee) within 24 hours of learning of the event:

Symptomatic Overdose of Study Drug: Accidental or intentional overdose of at least 2 times the intended dose of study drug within the intended therapeutic window, if associated with an AE.

Pregnancy: Although pregnancy is not considered an AE, it is the responsibility of the investigator to report to the sponsor (or designee), within 24 hours of identification, any pregnancy occurring in a female or female partner of a male, during the study or within 180 days of the last dose of study drug. Any complication of pregnancy affecting a female study patient or female partner of a male study patient, and/or fetus and/or newborn that meets the SAE criteria must be reported as an SAE. Outcome for all pregnancies should be reported to the sponsor.

9.4.4. Reporting Adverse Events Leading to Withdrawal from the Study

All AEs that lead to a patient's withdrawal from the study must be reported to the sponsor's Medical Monitor within 30 days.

9.4.5. Abnormal Laboratory, Vital Signs, or Electrocardiogram Results

The criteria for determining whether an abnormal objective test finding should be reported as an AE include:

- the test result is associated with accompanying symptoms, and/or
- the test result requires additional diagnostic testing or medical/surgical intervention, and/or
- the test result leads to a change in dosing (outside of protocol-stipulated dose adjustments), discontinuation from the study, significant additional concomitant drug treatment, or other therapy

Contact the Medical Monitor in the event the investigator feels that an abnormal test finding should be reported as an AE, although it does not meet any of the above criteria.

Repeating an abnormal test, in the absence of any of the above conditions, does not constitute an AE. Any abnormal test result that is determined to be an error does not require reporting as an AE.

Evaluation of severity of laboratory abnormalities will be assessed according to the scale outlined in Section 9.5.1.

9.4.6. Follow-up

Information for any non-SAE that starts during the treatment period or within 90 days after last dose of study treatment will be collected from the time of the event until resolution of the event, or until the patient's last study visit, whichever comes first.

Serious adverse event information will be collected until the event is resolved or considered chronic and/or stable.

9.5. Evaluation of Severity and Causality

9.5.1. Evaluation of Severity

The severity of AEs (including test findings classified as AEs) will be graded using the current version of the NCI-CTCAE grading system. Adverse events not listed in the NCI-CTCAE will be graded according to the following scale:

1 (Mild): Mild; asymptomatic or mild symptoms; clinical or diagnostic observations only; intervention not indicated

2 (Moderate): Moderate; minimal, local or noninvasive intervention indicated; limiting age-appropriate instrumental Activities of Daily Living (ADL)*

3 (Severe): Severe or medically significant but not immediately life-threatening; hospitalization or prolongation of hospitalization indicated; disabling; limiting self-care ADL**

4 (Life–threatening): Life-threatening consequences; urgent intervention indicated

5 (Death): Death related to AE

*Instrumental ADL refer to preparing meals, shopping for groceries or clothes, using the telephone, managing money, etc.

**Self-care ADL refer to bathing, dressing and undressing, feeding self, using the toilet, taking medications, and not bedridden.

If a laboratory value is considered as an AE, its severity should be based on the degree of physiological impairment the value indicates.

9.5.2. Evaluation of Causality

Relationship of Adverse Events to Study Drug:

The relationship of AEs to study drug will be assessed by the investigator and will be a clinical decision based on all available information. The following question will be addressed:

Is there a reasonable possibility that the AE may have been caused by the study drug?

The possible answers are:

Not Related: There is no reasonable possibility that the event may have been caused by the study drug

Related: There is a reasonable possibility that the event may have been caused by the study drug

A list of factors to consider when assessing the relationship of AEs to study drug is provided in Appendix 1.

- The investigator should justify the causality assessment of each SAE.

VV-RIM-00102568-2.0 Approved - 16 Apr 2020 GMT-5:00

Relationship of Adverse Events to Study Conduct:

The relationship of AEs to study conduct will be assessed by the investigator and will be a clinical decision based on all available information. The following question will be addressed:

Is there a reasonable possibility that the AE may have been caused by study conduct?

The possible answers are:

Not Related: There is no reasonable possibility that the event may have been caused by study conduct

Related: There is a reasonable possibility that the event may have been caused by study conduct

A list of factors to consider when assessing the relationship of AEs to study conduct is provided in Appendix 1.

The investigator should justify the causality assessment of each SAE.

9.6. Safety Monitoring

The investigator will monitor the safety of the study patient at his/her site(s) as per the requirements of this protocol and consistent with current Good Clinical Practice (GCP). Any questions or concerns should be discussed with the sponsor in a timely fashion. The sponsor will monitor the safety data from across all study sites. The Medical Monitor will have primary responsibility for the emerging safety profile of the compound but will be supported by other departments (eg, Pharmacovigilance and Risk Management and Biostatistics and Data Management). Safety monitoring will be performed on an ongoing basis (eg, individual review of SAEs) and on a periodic cumulative aggregate basis.

9.7. Investigator Alert Notification

Regeneron (or designee) will inform all investigators participating in this clinical study, as well as in any other clinical study using the same investigational drug, of any SAE that meets the relevant requirements for expedited reporting (an AE that is serious, unexpected based on the Investigator's Brochure or this protocol, and has a reasonable suspected causal relationship to the medicinal/study drug).

10. STATISTICAL PLAN

This section provides the basis for the statistical analysis plan (SAP) for the study. The SAP may be revised during the study to accommodate amendments to the clinical study protocol and to make changes to adapt to unexpected issues in study execution and data that may affect the planned analyses. The final SAP will be issued before the database is locked.

Analysis variables are listed in Section 4.

10.1. Statistical Hypothesis

The primary statistical hypotheses for Part 1 are that:

1. cemiplimab/chemo-f (Arm B) will prolong OS as compared with chemo-f (Arm A)

VV-RIM-00102568-2.0 Approved - 16 Apr 2020 GMT-5:00

2. cemiplimab/chemo-l/ipi (Arm C) will prolong OS as compared with chemo-f (Arm A)

The primary statistical hypothesis for Part 2 is that:

1. cemiplimab/chemo-f will prolong OS as compared with placebo/chemo-f.

10.2. Justification of Sample Size

See **the Expanded rationale for revising study to transition to a two part study** in **Section** 1.

Part 1:

Part 1 halted patient enrollment in August 2019 and by the end of August 2019, Part 1 randomized a total of 323 patients with approximately 215 patients in each of cemiplimab combination treatment arm versus the chemotherapy comparison (cemiplimab/chemo-f vs. chemo-f or cemiplimab/chemo-l/ipi vs. chemo-f). The sponsor assumes Part 1 has the same hypothesis assumption for PFS and OS as assumed for Part 2.

Specifically, the sponsor assumes that median OS of 12 months for patients treated with chemotherapy, and a hazard ratio of 0.65 in OS for each cemiplimab combination versus the chemotherapy comparison. Patients will be randomized in a 1:1:1 ratio to the chemo-f arm (Arm A), the cemiplimab/chemo-f arm (Arm B) versus cemiplimab/chemo-l/ipi (Arm C). Under these assumptions and for each cemiplimab combination versus the chemotherapy comparison, 151 deaths (70% out of 215 randomized patients each of the cemiplimab combination comparisons) are needed to yield approximately 74% power to detect statistical significance in OS by stratified Log-Rank testing procedure with an overall 2-sided type one error of 0.0499, given 2-sided type error of 0.0001 will be spent on first administrative analysis of PFS and ORR per investigator assessment.

The sponsor assumes a median PFS of 6 months for patients treated with chemotherapy alone, and a hazard ratio of 0.6667 in PFS for each cemiplimab combination treatment arm versus the chemotherapy comparison. Under these assumptions and for each cemiplimab combination treatment arm versus the chemotherapy comparison, 161 PFS events (75% out of 215 randomized patients in each of the cemiplimab combination comparisons) are needed to yield approximately 73% power to detect statistical significance in PFS by stratified Log-Rank testing procedure with an overall 2-sided type one error of 0.0499.

Part 2:

Historically, in patients with stage IIIB or stage IV NSCLC treated with cisplatin or carboplatin plus paclitaxel Q3W, the median PFS has ranged from approximately 2.7 to 6.4 months (El-Shenshawy 2012, Rosell 2002, Scagliotti 2002, Schiller 2002, Shimizu 2013, Socinski 2016); the median OS has ranged from approximately 11.3 to 20.9 months (Socinski 2016, De Lima Lopes 2018, Borghaei 2017, Brahmer 2017, Gandhi 2018, Paz-Ares 2018).

Based on this historical data, the sponsor assumes a median OS of 12 months for patients treated with chemotherapy plus placebo, and a hazard ratio of 0.65 in OS between cemiplimab/chemo-f arm and placebo/chemo-f arm. Patients will be randomized in a 2:1 ratio to the cemiplimab/chemo-f arm (Treatment Arm B) versus the placebo/chemo-f arm (Treatment Arm A). Under these assumptions, 291 deaths are needed to yield approximately 93% power to detect

CONFIDENTIAL

VV-RIM-00102568-2.0 Approved - 16 Apr 2020 GMT-5:00

statistical significance in OS at a 2-sided Type I error level or 0.05 between the two treatment arms.

Considering an enrollment period of 14 months (11 patients per month for the first 4 months, 26 patients per month for month 5 to 8, 50 patients per month afterwards), an approximately 24-month follow-up period for OS after completion of enrollment, and 10% annual dropout, the enrollment of approximately 450 randomized patients is needed to obtain 291 deaths for the final analysis of OS.

Based on these same studies, the sponsor assumes a median PFS of 6 months for patients treated with chemotherapy alone, and a hazard ratio of 0.6667 in PFS between the cemiplimab/chemo-f arm and placebo/chemo-f arm. . With these assumptions and Type I error at two-sided 0.05 level, the power for analysis of PFS will be 90% or more if it is performed after 288 or more PFS events are observed.

10.3. Analysis Sets

10.3.1. Efficacy Analysis Sets

The full analysis set (FAS) will be defined separately for Part 1 and Part 2 of this study. The FAS includes all randomized patients and will be the intention-to-treat population. The FAS is based on the treatment allocation (as randomized). All efficacy endpoints will be analyzed using the FAS.

10.3.2. Safety Analysis Set

The safety analysis set (SAF) will be defined separately for Part 1 and Part 2 of this study. The SAF includes all randomized patients who received any study drug; it is based on the treatment received (as treated). Treatment administration and all clinical safety variables will be analyzed using the SAF.

10.3.3. Other Analysis Sets

The PK population includes all randomized patients (safety population) who receive cemiplimab and who have at least 1 non-missing cemiplimab concentration assay result following the first dose of cemiplimab up to the end of the study.

The ADA analysis set includes all treated patients who received any study drug and had at least 1 non-missing post-baseline ADA assay result following the first dose of study drug.

The DLT analysis set includes all patients treated with cemiplimab/chemo-l/ipi who are DLT evaluable, defined as the patients who completed the DLT observation period and those patients who discontinued early due to the development of a DLT. This population will be used for the assessment of DLTs. The patients will be analyzed as treated.

10.4. Statistical Methods

For continuous variables, descriptive statistics will include the following information: the number of patients reflected in the calculation (n), mean, median, standard deviation, minimum, and maximum.

For categorical or ordinal data, frequencies and percentages will be displayed for each category.

The descriptive summary of time-to-event data will include median time to event and the corresponding 95% CI using the Kaplan-Meier method.

All analyses for each study part will be performed separately.

10.4.1. Patient Disposition

The following will be provided:

- The total number of screened patients: met the inclusion criteria regarding the target indication and signed the ICF
- The total number of patients in the FAS
- The total number of patients in the SAF
- The total number of patients who discontinued treatment and the reasons for treatment discontinuation
- The total number of patients who discontinued the study and the reasons for discontinuation
- A listing of patients treated but not randomized, patients randomized but not treated, and patients randomized but not treated as randomized
- A listing of patients prematurely discontinued from treatment and study, along with reasons for discontinuation

10.4.2. Demography and Baseline Characteristics

Demographic and baseline characteristics will be summarized descriptively by treatment arm, and by all patients combined.

10.4.3. Medical History

Medical history will be summarized by primary system organ class (SOC) and preferred term (PT) for each treatment arm, with the table sorted by decreasing frequency of SOC, followed by PT, based on the overall incidence between treatment arms.

10.4.4. Prior Medications/Concomitant Medications

Number and proportion of patients taking prior/concomitant medication will be summarized by decreasing frequency of anatomical therapeutic chemical (ATC) level 2 and ATC level 4 according to the current version of the World Health Organization Drug Dictionary, based on the overall incidence between treatment arms.

Listings of pre-treatment medication and concomitant medications will include generic name, ATC levels 2 and 4, indication, the study day onset, the study end date (defined similarly as for study onset day), ongoing status, dose, frequency, and route.

For medications that are started before treatment, the study day onset is defined as the date of medication start to date of the first dose of treatment; for medications that are started on or after treatment, the study day onset is defined as the date of medication start to date of the first dose + 1.

10.4.5. Efficacy Analyses

For the time-to-event endpoints (PFS and OS), the non-parametric Kaplan-Meier method will be used to estimate the survival curves. The treatment difference in survival will be assessed by the stratified Log-Rank test. A stratified Cox proportional hazard model with Efron's method of tie handling will be used to assess the magnitude of the treatment difference (ie, the hazard ratio). The hazard ratio and its 95% confidence interval from the stratified Cox model with a single treatment covariate will be reported. The same stratification factors used for randomization per IWRS will be applied to both the stratified logrank test and the stratified Cox model.

ORR will be analyzed using the Cochran-Mantel-Haenszel test stratified by stratification factors at randomization per IWRS. Objective response rate and the corresponding exact 95% CI will be calculated by Clopper-Pearson method for each treatment arm.

Overall survival rate at a landmark (12 months, 18 months, and 24 months) will be summarized using Kaplan-Meier method for each treatment arm.

DOR will be summarized for confirmed responders (CR or PR) using Kaplan-Meier method for each treatment arm .

The change in EORTC QLQ-C30 and EORTC QLQ-LC13 scores from first assessment to the end of the study will be summarized descriptively at each post-baseline time point and compared using a mixed effects model, if appropriate.

Part 1:

An administrative analysis for PFS and ORR (both per investigator assessment using RECIST 1.1) will be performed in patients who were randomized before the end of August 2019.

For the efficacy claim purpose, the analyses of primary and key secondary endpoints will be tested in the following order:

- OS comparison for cemiplimab/chemo-f vs. chemo-f
- OS comparison for cemiplimab/chemo-l/ipi vs. chemo-f
- PFS per IRC assessment comparison for cemiplimab/chemo-f vs. chemo-f
- PFS per IRC assessment comparison for cemiplimab/chemo-l/ipi vs. chemo-f
- ORR per IRC assessment comparison for cemiplimab/chemo-f vs. chemo-f
- ORR per IRC assessment comparison for cemiplimab/chemo-l/ipi vs. chemo-f

An interim analysis and final analysis for OS will be performed when 125 (83% out of total OS events, 58% out of 215 randomized patients) and 151 (70% out of 215 randomized patients) deaths are observed in the comparison of cemiplimab/chemo-f vs. chemo-f.

VV-RIM-00102568-2.0 Approved - 16 Apr 2020 GMT-5:00

If the analyses of OS are statistically significant for both cemiplimab combination vs. chemotherapy comparisons (in the order of cemiplimab/chemo-f vs. chemo-f and cemiplimab/chemo-l/ipi vs. chemo-f), the key secondary endpoint PFS per IRC assessment will be analyzed using the same statistical method and strategy as used in the analyses of OS. If analyses of PFS are statistically significant, ORR per IRC assessment will be analyzed following the same analysis order as OS and PFS.

Part 2:

The primary and key secondary endpoints will be tested in the following order: OS, PFS, and ORR.

The analyses of primary endpoint of OS will be performed as follows: 1) two interim analyses for OS will be performed when approximately 146 (50% out of total OS events) and 204 (70% out of total OS events) deaths are observed; 2) final analysis for OS will be performed when approximately 291 deaths are observed.

If analysis of OS is statistically significant, the key secondary endpoint PFS will be analyzed using the same statistical method as used in analysis of OS. If analysis of PFS is statistically significant, the key secondary endpoint of ORR will be analyzed.

Subgroup Analyses

Part 2:

To determine the consistency of treatment effect across various demographic and baseline subgroups, the estimate of the between-group treatment effect (with a nominal 95% CI) for the primary and key secondary endpoints will be estimated and plotted within each category of the following subgroup variables:

- Age category (≤65 versus >65 years)
- Gender (female, male)
- Race (white, non-white)
- Histology (squamous, non-squamous)
- PD-L1 expression levels (<1% versus 1% to <50% versus >=50%)
- ECOG status (0 versus 1)
- Geographic region of enrolling site
- Ethnicity

10.4.6. Safety Analyses

Safety observations and measurements, including drug exposure, AEs, laboratory data, vital signs, and ECOG performance status, will be summarized and presented in tables and listings.

Dose-limiting toxicities observed during the DLT evaluation period will be summarized by treatment arm and will be assessed using the DLT analysis set.

10.4.6.1. Adverse Events

Definitions

For safety variables, 3 observation periods are defined:

- The pre-treatment period is defined as the time from signing the ICF to before the first dose of study drug
- The on-treatment period is defined as the day from the first dose of study drug to the day of the last dose of study drug plus 90 days or 1 day before patients receive their first dose of new anti-cancer systemic therapy, whichever is earlier.
- The post-treatment period is defined as the time after follow-up visit 1.

Treatment-emergent adverse events are defined as those developed or worsened during the on-treatment period and any TEAEs during the post-treatment period.

Analysis

All AEs reported in this study will be coded using the currently available version of the Medical Dictionary for Regulatory Activities (MedDRA). Coding will be to lowest-level terms. The verbatim text, the PT, and the primary SOC will be listed.

Summaries of all TEAEs by treatment arm will include the following:

- The number (n) and percentage (%) of patients with at least 1 TEAE by SOC and PT
- TEAEs by severity (graded according to the current version of the NCI-CTCAE), presented by SOC and PT
- TEAEs leading to death
- TEAEs related to study drug, presented by SOC and PT
- AESI

Deaths and other SAEs will be listed and summarized by treatment arm.

Events of NCI-CTCAE grade 3 and grade 4 severity will be summarized by treatment arm.

Treatment-emergent adverse events leading to permanent treatment discontinuation will be listed and summarized by treatment arm.

10.4.6.2. Other Safety Analyses

Vital Signs

Vital signs (temperature, heart rate, seated blood pressure, and respiration rate) will be summarized by baseline and change from baseline to each scheduled assessment time with descriptive statistics.

Laboratory Tests

Laboratory test results will be listed, and the number and percentage of patients with NCI-CTCAE grade 3 or grade 4 laboratory values will be summarized by laboratory test. Shift tables may be generated if applicable.

CONFIDENTIAL

VV-RIM-00102568-2.0 Approved - 16 Apr 2020 GMT-5:00

10.4.6.3. Treatment Exposure

Treatment duration, number of doses, dose intensity, and number of cycles administered will be summarized by treatment arm.

10.4.6.4. Treatment Compliance

Treatment compliance, i will be summarized by treatment arm. The analysis methods will be detailed in the SAP.

10.4.7. Analysis of Drug Concentration Data

Cemiplimab concentrations in serum will be reported over time as individual values with descriptive statistics. The PK data in this study may be included in a population PK analysis that will be presented in a separate report.

Blood samples for analysis of ipilimumab concentrations will be stored for possible future analysis, the results of which may be reported in the same way as for cemiplimab concentrations.

10.4.8. Analysis of Anti-Drug Antibody Data

The ADA variables described in Section 4.4 will be summarized using descriptive statistics in the ADA analysis set of the cemiplimab treatment arms (Treatment Arms B and C). Frequency tables of the proportion of patients with treatment-emergent, treatment-boosted, persistent ADA response, and NAb status in the NAb assay will be presented as absolute occurrence (n) and percentage of patients (%), presented by treatment arms.

Plots of cemiplimab concentrations will be examined, and the influence of ADAs on individual concentration-time profiles may be evaluated. Assessment of impact of ADA on safety and efficacy may be provided.

10.4.9. Analysis of Biomarker Data

Biomarker analyses in this study will be exploratory in nature, and results will be summarized in a separate report. Detailed description of statistical methods that will be used for biomarker data analyses will be provided in a separate Biomarker Analytical Plan.

10.5. Multiplicity Considerations

The statistical analyses of the 2 parts will be conducted independently and summarized separately. Therefore, statistical control of overall type I error for the whole study is not planned. The familywise 2-sided type error of 0.05 is controlled within each part of study.

Part 1:

For the 2 cemiplimab combinations versus standard of care chemotherapy comparisons, familywise type-I error rate of 0.05 is controlled by allocating 0.0001 to the administrative analyses of PFS and ORR per investigator assessment (0.00005 for each endpoint) and 0.0499 to the hierarchical testing of the OS, PFS, and ORR per IRC in the order of cemi/chemo-f vs. chemo-f and cemi/chemo-l/ipi vs. chemo-f for each endpoint.

Specifically, PFS per investigator assessment and ORR per investigator assessment will be controlled at nominal 2-sided 0.00005 level respectively in patients who were randomized up to the end of August 2019. The multiplicity across OS, PFS per IRC assessment, and ORR per IRC assessment will be controlled at two-sided 0.0499 level by the hierarchical approach. That is, the analysis of PFS will be performed at two-sided 0.0499 level only if analyses of OS are statistically significant for both cemiplimab comparisons, and the analyses of ORR will be performed at two-sided 0.0499 level only if the analyses of PFS are statistically significant.

The type I error for the interim analysis of OS at approximately 125 events and final analysis of OS at approximately 151 events is controlled at two-sided 0.0499 level using an α-spending function according to Lan-DeMets (O'Brien 1979, Gordon Lan 1983) in the intent-to-treat (ITT) population. The exact nominal p-values needed to declare statistical significance at the time of these analyses for OS will depend on the actual number of OS events at the time of the analysis. The method of adjustment will be detailed in the study SAP.

All other statistical comparisons will be exploratory in nature and, therefore, not controlled for multiplicity and should be interpreted accordingly.

Table 8: Summary of Decision Guidance for Part 1 Analysis

Analysis	Endpoint (projected 2-sided alpha allocation)	Testing Steps
Administrative Look	ORR per investigator assessment in patients randomized by the end of August 2019 (0.00005)	1a. ORR: Arm B vs. Arm A<br>1b. ORR: Arm C vs. Arm A
	PFS per investigator assessment in patients randomized by the end of August 2019 (0.00005), will be tested in parallel of ORR per investigator assessment analyses	1a'. PFS: Arm B vs. Arm A<br>1b'. PFS: Arm C vs. Arm A
Efficacy Analysis	OS in ITT (0.0499)	2a. OS: Arm B vs. Arm A<br>2b. OS: Arm C vs. Arm A
	PFS per IRC in ITT (0.0499), only if the analyses of OS (Steps 2a and 2b) at either second interim analysis or final OS analysis statistically significant	2c. PFS: Arm B vs. Arm A<br>2d. PFS: Arm C vs. Arm A
	ORR in ITT, only if OS and PFS analyses are statistically significant (0.0499)	2e. ORR: Arm B vs. Arm A<br>2f. ORR: Arm C vs. Arm A

Part 2:

The family-wise type I error across the test of primary and key secondary endpoints and the repeated testing of OS in the 2 interim analyses and final analyses in Part 2 is controlled at 2-sided 0.05 level.

The multiplicity between analyses of OS, PFS, and ORR will be controlled at 2-sided 0.05 level by the hierarchical approach. That is, the analysis of PFS will be performed at 2-sided 0.05 level only if analysis of OS is statistically significant, and the analysis of ORR will be performed at 2-sided 0.05 level only if analysis of PFS is statistically significant.

The type I error for the 2 interim analyses of OS at 146 (50%) and 204 (70%) and final analysis of OS is controlled at 2-sided 0.05 level using an α-spending function according to Lan-DeMets (O'Brien 1979, Gordon Lan 1983). The exact nominal p-values needed to declare statistical significance at the time of these analyses for OS will depend on the actual number of OS events at the time of the analysis. The method of adjustment will be detailed in study SAP.

All other statistical comparisons will be exploratory in nature and, therefore, not controlled for multiplicity and should be interpreted accordingly.

10.6. Interim Analyses

Part 1:

For administrative purposes, analyses will be performed for PFS and ORR per investigator assessment in Part 1 patients who were randomized by August 2019. This administrative analysis will be performed after all Part 1 patients who were randomized by August 2019 have had the opportunity for 3 tumor assessments.

An interim analysis of OS will be performed when 125 deaths (83% of total OS events) have been observed in the comparison of cemiplimab/chemo-f vs. chemo-f.

The final analysis of OS will be performed when 151 deaths (cumulatively 70% out of 215 randomized patients) have been observed.

The Lan-DeMets alpha spending for analysis of OS is specified in Table 9 .

Table 9: Alpha Spending for Analysis of OS

		**Value**
Interim Analysis for OS	Z	2.205
Death =125	Alpha (2-sided[a])	0.02745
Final Analysis for OS<br>Deaths=151	Z	2.034
	Alpha (2-sided)	0.04195

[a] As a two-sided test is used at interim, the superiority or futility of cemiplimab treatment will be claimed if the statistical boundary is crossed.

Final analysis of PFS will be performed at 2-sided 0.0499 level when analysis of OS is statistically significant either at the interim analysis or at final analysis for OS. At the time of

CONFIDENTIAL

VV-RIM-00102568-2.0 Approved - 16 Apr 2020 GMT-5:00

PFS analysis, it is estimated more than 161 PFS events have been observed in each of the cemiplimab comparison.

Part 2:

Two interim analyses of OS will be performed when 146 deaths (50% of total OS events) and 204 deaths (70% of total OS events) have been observed.

The final analysis of OS will be performed 291 deaths (cumulative) have been observed.

The alpha spending for analysis of OS is specified in Table 10.

Table 10: Alpha Spending for Analysis of OS

		**Value**
First Interim Analysis for OS	Z	2.958
Death =146	Alpha (2-sided[a])	0.00310
Second Interim Analysis for OS Deaths=204	Z	2.465
	Alpha (2-sided[a])	0.01370
Final Analysis for OS	Z	2.002
	Alpha (2-sided)	0.04528

[a] As a two-sided test is used at interim, the superiority or futility of cemiplimab treatment will be claimed if the statistical boundary is crossed.

Final analysis of PFS will be performed at 2-sided 0.05 level when analysis of OS is statistically significant either at interim or final analysis for OS. At the time of PFS analysis, if more than 288 PFS events have been observed, the analysis of PFS will have at least 90% power at two-sided 0.05 level.

Analysis of ORR will be performed when PFS is statistically significant.

10.7. Additional Statistical Data Handling Conventions

The following analysis and data conventions will be followed:

Definition of baseline:

- Unless otherwise specified, the last assessment before the initial administration of cemiplimab or placebo will be considered the baseline evaluation.

General rules for handling missing data:

- Unless otherwise specified, there will be no imputations for missing data.
- The pattern of missing data and potential prognostic factors for missing data (QOL, clinical neurologic assessment and mental status) will be examined to guide the use of proper statistical models.
- If the start date of an AE or concomitant medication is incomplete or missing, it will be assumed to have occurred on or after the intake of study drug except if an incomplete date (eg, month and year) clearly indicates that the event started prior to treatment. If

the partial date indicates the same month or year of the intake of study drug date, then the start date by the study drug intake date will be imputed; otherwise, the missing day or month by the first day or the first month will be imputed.

Visit windows:

- Assessments taken outside of protocol allowable windows will be displayed according to the eCRF assessment recorded by the investigator.

Unscheduled assessments:

- Extra assessments (laboratory data or vital signs associated with non-protocol-defined clinical visits or obtained in the course of investigating or managing AEs) will be included in the listings, but not in the summaries. If more than 1 laboratory value is available for a given visit, the first observation will be used in summaries, and all observations will be presented in listings.

10.8. Statistical Considerations Surrounding the Premature Termination of a Study

The study is expected to end after the last visit of the last patient.

If the study is terminated prematurely, only those parameters required for the development program and/or reporting to regulatory authorities will be summarized. Investigator and sponsor responsibilities surrounding the premature termination of a study are presented in Section 16.1.

11. DATA MANAGEMENT AND ELECTRONIC SYSTEMS

11.1. Data Management

A data management plan specifying all relevant aspects of data processing for the study (including data validation, cleaning, correcting, and releasing) will be maintained and stored at Regeneron.

A medical coding plan will specify the processes and the dictionary used for coding. All data coding (eg, AEs, baseline findings, medication, medical history/surgical history) will be done using internationally recognized and accepted dictionaries.

The eCRF data for this study will be collected using an electronic data capture (EDC) tool. User training must be documented before the user is granted access to the EDC system.

11.2. Electronic Systems

Electronic systems that may be used to process and/or collect data in this study will include the following:

- IVRS/IWRS system – randomization, study drug supply
- EDC system – data capture
- Statistical Analysis System (SAS) – statistical review and analysis

- Pharmacovigilance safety database
- ARGUS safety database

12. STUDY MONITORING

12.1. Monitoring of Study Sites

The study monitor and/or designee (eg, contract research organization [CRO] monitor) will visit each site prior to enrollment of the first patient, and periodically during the study.

12.2. Source Document Requirements

Investigators are required to prepare and maintain adequate and accurate patient records (source documents).

The investigator must keep all source documents on file with the case report form (CRF) (throughout this protocol, CRF refers to either a paper CRF or an electronic CRF [eCRF]). Case report forms and source documents must be available at all times for inspection by authorized representatives of the sponsor and regulatory authorities.

12.3. Case Report Form Requirements

Study data obtained in the course of the clinical study will be recorded on eCRFs within the EDC system by trained site personnel. All required eCRFs must be completed for each and every patient enrolled in the study. After review of the clinical data for each patient, the investigator must provide an electronic signature. A copy of each patient eCRF casebook is to be retained by the investigator as part of the study record and must be available at all times for inspection by authorized representatives of the sponsor and regulatory authorities.

Corrections to the eCRF will be entered in the eCRF by the investigator or an authorized designee. All changes, including details regarding the date and person performing corrections, will be available via the audit trail, which is part of the EDC system. For corrections made via data queries, a reason for any alteration must be provided.

13. AUDITS AND INSPECTIONS

This study may be subject to a quality assurance audit or inspection by the sponsor or regulatory authorities. Should this occur, the investigator is responsible for the following:

- Informing the sponsor of a planned inspection by the authorities as soon as notification is received, and authorizing the sponsor's participation in the inspection
- Providing access to all necessary facilities, study data, and documents for the inspection or audit
- Communicating any information arising from inspection by the regulatory authorities to the sponsor immediately
- Taking all appropriate measures requested by the sponsor to resolve the problems found during the audit or inspection

Documents subject to audit or inspection include, but are not limited to, all source documents, eCRFs, medical records, correspondence, ICFs, IRB/EC files, documentation of certification and quality control of supporting laboratories, and records relevant to the study maintained in any supporting pharmacy facilities. Conditions of study material storage are also subject to inspection. In addition, representatives of the sponsor may observe the conduct of any aspect of the clinical study or its supporting activities both within and outside of the investigator's institution.

In all instances, the confidentiality of the data must be respected.

14. ETHICAL AND REGULATORY CONSIDERATIONS

14.1. Good Clinical Practice Statement

It is the responsibility of both the sponsor and the investigator(s) to ensure that this clinical study will be conducted in accordance with the ethical principles that have their origin in the Declaration of Helsinki, and that are consistent with the ICH guidelines for GCP and applicable regulatory requirements.

14.2. Informed Consent

The principles of informed consent are described in ICH guidelines for GCP.

The ICF used by the investigator must be reviewed and approved by the sponsor prior to submission to the appropriate IRB/EC. A copy of the IRB/EC-approved ICF and documentation of approval must be provided to the sponsor before study drug will be shipped to the study site.

It is the responsibility of the investigator or designee (if acceptable by local regulations) to obtain written informed consent from each patient prior to his/her participation in the study and after the aims, methods, objectives, and potential hazards of the study have been explained to the patient in language that he/she can understand. The ICF should be signed and dated by the patient and by the investigator or authorized designee who reviewed the ICF with the patient.

- Patients who can write but cannot read will have the ICF read to them before signing and dating the ICF.
- Patients who can understand but who can neither write nor read will have the ICF read to them in presence of an impartial witness, who will sign and date the ICF to confirm that informed consent was given.

The original ICF must be retained by the investigator as part of the patient’s study record, and a copy of the signed ICF must be given to the patient.

If new safety information results in significant changes in the risk-benefit assessment, the ICF must be reviewed and updated appropriately. All study patients must be informed of the new information and provide their written consent if they wish to continue in the study. The original signed revised ICF must be maintained in the patient’s study record and a copy must be given to the patient.

A written informed consent should be obtained for treatment beyond radiologic disease progression, acknowledging that this practice is not considered standard in the treatment of cancer.

14.3. Patients' Confidentiality and Data Protection

The investigator must take all appropriate measures to ensure that the anonymity of each study patient will be maintained. Patients should be identified by a patient identification number, only, on eCRFs or other documents submitted to the sponsor. Documents that will not be submitted to the sponsor (eg, signed ICF) must be kept in strict confidence.

The patient's and investigator's personal data, which may be included in the sponsor database, will be treated in compliance with all applicable laws and regulations. The sponsor shall take all appropriate measures to safeguard and prevent access to this data by any unauthorized third party.

14.4. Institutional Review Board/Ethics Committee

An appropriately constituted IRB/EC, as described in ICH guidelines for GCP, must review and approve:

- The protocol, ICF, and any other materials to be provided to the patients (eg, advertising) before any patient may be enrolled in the study
- Any amendment or modification to the study protocol or ICF before implementation, unless the change is necessary to eliminate an immediate hazard to the patient, in which case the IRB/EC should be informed as soon as possible
- Ongoing studies on an annual basis or at intervals appropriate to the degree of risk

In addition, the IRB/EC should be informed of any event likely to affect the safety of patients or the continued conduct of the clinical study.

A copy of the IRB/EC approval letter with a current list of the IRB/EC members and their functions must be received by the sponsor prior to shipment of drug supplies to the investigator. The approval letter should include the study number and title, the documents reviewed, and the date of the review.

Records of the IRB/EC review and approval of all study documents (including approval of ongoing studies) must be kept on file by the investigator.

15. PROTOCOL AMENDMENTS

The sponsor may not implement a change in the design of the protocol or ICF without an IRB/EC-approved amendment. Regulatory approvals will also be sought as required by regulatory guidance.

VV-RIM-00102568-2.0 Approved - 16 Apr 2020 GMT-5:00

16. PREMATURE TERMINATION OF THE STUDY OR CLOSE-OUT OF A SITE

16.1. Premature Termination of the Study

The sponsor has the right to terminate the study prematurely. Reasons may include efficacy, safety, or futility, among others. Should the sponsor decide to terminate the study, the investigator(s) will be notified in writing.

16.2. Close-out of a Site

The sponsor and the investigator have the right to close-out a site prematurely.

Investigator's Decision

The investigator must notify the sponsor of a desire to close-out a site in writing, providing at least 30 days' notice. The final decision should be made through mutual agreement with the sponsor. Both parties will arrange the close-out procedures after review and consultation.

Sponsor's Decision

The sponsor will notify the investigator(s) of a decision to close-out a study site in writing. Reasons may include the following, among others:

- The investigator has received all items and information necessary to perform the study, but has not enrolled any patient within a reasonable period of time
- The investigator has violated any fundamental obligation in the study agreement, including but not limited to, breach of this protocol (and any applicable amendments), breach of the applicable laws and regulations, or breach of any applicable ICH guidelines
- The total number of patients required for the study are enrolled earlier than expected

In all cases, the appropriate IRB/EC and Health Authorities must be informed according to applicable regulatory requirements, and adequate consideration must be given to the protection of the patients' interests.

17. STUDY DOCUMENTATION

17.1. Certification of Accuracy of Data

A declaration assuring the accuracy and content of the data recorded on the eCRF must be signed electronically by the investigator. This signed declaration accompanies each set of patient final eCRF that will be provided to the sponsor.

17.2. Retention of Records

The investigator must retain all essential study documents, including ICFs, source documents, investigator copies of eCRFs, and drug accountability records for at least 15 years following the completion or discontinuation of the study, or longer, if a longer period is required by relevant

regulatory authorities. The investigator must consult with the sponsor before discarding or destroying any essential study documents following study completion or discontinuation. Records must be destroyed in a manner that ensures confidentiality.

If the investigator's personal situation is such that archiving can no longer be ensured, the investigator must inform the sponsor and the relevant records will be transferred to a mutually agreed-upon destination.

18. CONFIDENTIALITY

Confidentiality of information is provided as a separate agreement.

19. FINANCING AND INSURANCE

Financing and insurance information is provided as a separate agreement.

20. PUBLICATION POLICY

The publication policy is provided as a separate agreement.

21. REFERENCES

ALIMTA [package insert]. Indianapolis, IN: Eli Lilly and Company; 2013.

ALIMTA [European Union Summary of Product Characteristics]. Indianapolis, IN: Eli Lilly and Company; 2013.

Antonia S, Goldberg SB, Balmanoukian A, Chaft JE, Sanborn RE, Gupta A, et al. Safety and antitumour activity of durvalumab plus tremelimumab in non-small cell lung cancer: a multicentre, phase 1b study. Lancet Oncol. 2016;17(3):299-308.

Bergman B, Aaronson NK, Ahmedzai S, Kaasa S, Sullivan M. The EORTC QLQ-LC13: a modular supplement to the EORTC Core Quality of Life Questionnaire (QLQ-C30) for use in lung cancer clinical trials. EORTC Study Group on Quality of Life. Eur J Cancer. 1994;30A(5):635-42.

Besse B, Adjei A, Baas P, Meldgaard P, Nicolson M, Paz-Ares L, et al. 2nd ESMO consensus conference on lung cancer: non-small-cell lung cancer first-line/second and further lines of treatment in advanced disease. Ann Oncol. 2014;25:1475-84.

Bjordal K, de Graeff A, Fayers PM, Hammerlid E, van Pottelsberghe C, Curran D, et al. A 12 country field study of the EORTC QLQ-C30 (version 3.0) and the head and neck cancer specific module (EORTC QLQ-H;N35) in head and neck patients. EORTC Quality of Life Group. Eur J Cancer. 2000;36(14):1796-807.

Borghaei et al. Updated results from KEYNOTE-021 cohort G: A randomized, phase 2 study of pemetrexed and carboplatin (PC) with or without pembrolizumab (pembro) as first-line therapy for advanced nonsquamous NSCLC. ESMO 2017 Congress, September 2017.

Borghaei H, Langer CJ, Gadgeel S, Papadimitrakopoulou VA, Patnaik A, Powell SF, et al. 24-Month Overall Survival from KEYNOTE-021 Cohort G: Pemetrexed and Carboplatin with or

without Pembrolizumab as First-Line Therapy for Advanced Nonsquamous Non-Small Cell Lung Cancer. J Thorac Oncol 2018.

Brahmer, JR et al. 2017. Updated Analysis of KEYNOTE-024: Pembrolizumab Versus Platinum-based Chemotherapy for Advanced NSCLC With PD-L1≥50%. International Association for the Study of Lung Cancer 18th World COnference on Lung Cancer. Yokohama, Japan.

Bray F, Ren JS, Masuyer E, Ferlay J. Estimates of global cancer prevalence for 27 sites in the adult population in 2008. Int J Cancer. 2013;132:1133-45.

Buchbinder EI, Desai A. CTLA-4 and PD-1 pathways: similarities, differences, and implications of their inhibition. Am J Clin Oncol. 2016;39:98-106.

Creelan BC. Update on immune checkpoint inhibitors in lung cancer. Cancer Control. 2014;21:80-9.

Dela Cruz CS, Tanoue LT, Matthay RA. Lung cancer: epidemiology, etiology, and prevention. Clin Chest Med 2011; 32(4):605-44.

D'Incecco A, Andreozzi M, Ludovini V, Rossi E, Capodanno A, Landi L, et al. PD-1 and PD-L1 expression in molecularly selected non-small-cell lung cancer patients. Br J Cancer. 2015;112:95-102.

De Lima Lopes G, Wu Y, Sadowski S, Zhang J, Rangwala R, et al. P2.43: Pembrolizumab vs platinum-based chemotherapy for PD-L1+ NSCLC: phase 3, randomized, open-label KEYNOTE-042: track: immunotherapy. J Thorac Oncol. 2018;11(10S): S244-S245.

Eisenhauer EA, Therasse P, Bogaerts J, Schwartz LH, Sargent D, Ford R, et al. New response evaluation criteria in solid tumours: revised RECIST guideline (version 1.1). Eur J Cancer. 2009;45:228-47.

El-Shenshawy HM, Taema S, El-Zahaf E, El-Beshbeshi W, Eldeen DS, Fathy A. Advanced non-small cell lung cancer in elderly patients: the standard every 3-weeks versus weekly paclitaxel with carboplatin. Chest. 2012;61:485-93.

Ettinger DS, Wood DE, Akerley W, Bazhenova LA, Borghaei H, Camidge DR, et al. NCCN Guidelines insights: non-small cell lung cancer, version 4.2016. J Natl Compr Canc Netw. 2016;14:255-64.

Fossella F, Pereira JR, von Pawel J, Pluzanska A, Gorbounova V, Kaukel E, et al. Randomized, multinational, phase III study of docetaxel plus platinum combinations versus vinorelbine plus cisplatin for advanced non-small-cell lung cancer: the TAX 326 study group. J Clin Oncol. 2003;21:3016-24.

Freshwater T, Kondic A, Ahamadi M, Li CH, de Greef R, de Alwis D, Stone JA, et al. Evaluation of dosing strategy for pembrolizumab for oncology indications. J Immunother Cancer. 2017;16(5):43.

Gandhi L, Rodríguez-Abreu D, et al. Pembrolizumab plus Chemotherapy in Metastatic Non-Small-Cell Lung Cancer. N Engl J Med. 2018 May 31;378(22):2078-2092. doi: 10.1056/NEJMoa1801005. Epub 2018 Apr 16.

VV-RIM-00102568-2.0 Approved - 16 Apr 2020 GMT-5:00

Gettinger S, Rizvi NA, Chow LQ, Borghaei H, Brahmer J, Ready N, et al. First-line nivolumab monotherapy and nivolumab plus ipilimumab in patients with advanced NSCLC: Long-term outcomes from CheckMate 012. IASLC 17th World Congress. 2016;Abstract OA03.01.

Gordon Lan KK, Demets DL, Discrete sequential boundaries for clinical trials. Biometrika 1983; 70(3):659-63

Govindan R, Szczesna A, et al. Phase III Trial of Ipilimumab Combined With Paclitaxel and Carboplatin in Advanced Squamous Non-Small-Cell Lung Cancer. J Clin Oncol. 2017 Oct 20;35(30):3449-3457. doi: 10.1200/JCO.2016.71.7629. Epub 2017 Aug 30.

Hellmann MD, Rizvi NA, Goldman JW, Gettinger SN, Borghaei H, Brahmer JR, et al. Nivolumab plus ipilimumab as first-line treatment for advanced non-small-cell lung cancer (CheckMate 012): results of an open-label, phase 1, multicohort study. Lancet Oncol. 2017;18:31-41.

Hofmann L, Forschner A, Loquai C, Goldinger SM, Zimmer L, Uqurel S, et al. Cutaneous, gastrointestinal, hepatic, endocrine, and renal side-effects of anti-PD-1 therapy. Eur J Cancer 2016;60:190-209.

Jotte RM, et al. IMpower131: Primary PFS and safety analysis of a randomized phase III study of atezolizumab + carboplatin + paclitaxel or nab-paclitaxel vs carboplatin + nab-paclitaxel as 1L therapy in advanced squamous NSCLC. 2018 ASCO Annual Meeting. Abstract LBA9000.

Kerr KM, Tsao MS, Nicholson AG, Yatabe Y, Wistuba II, Hirsch FR; IASLC Pathology Committee. Programmed death-ligand 1 immunohistochemistry in lung cancer: in what state is this art? J Thorac Oncol. 2015;10:985-9.

KEYTRUDA [package insert]. Whitehouse Station, NY: Merck & Co; 2015.

Larkin J, Chiarion-Sileni V, Gonzalez R, Grob JJ, Cowey CL, Lao CD, et al. Combined nivolumab and ipilimumab or monotherapy in untreated melanoma. N Engl J Med. 2015;373:23-34.

Leighl NB. Treatment paradigms for patients with metastatic non-small-cell lung cancer: first-, second-, and third-line. Curr Oncol. 2012;19:S52-8.

AstraZeneca Press Release, 27 July 2017. https://www.astrazeneca.com/media-centre/press-releases/2017/astrazeneca-reports-initial-results-from-the-ongoing-mystic-trial-in-stage-iv-lung-cancer-27072017.html.

AstraZeneca Press Release, 16 November 2018. https://www.astrazeneca.com/media-centre/press-releases/2018/astrazeneca-provides-update-on-the-phase-iii-mystic-trial-of-imfinzi-and-tremelimumab-in-stage-iv-non-small-cell-lung-cancer16112018.html.

Naidoo J, Page DB, Li BT, Connell LC, Schindler K, Lacouture ME, et al. Toxicities of the anti-PD-1 and anti-PD-L1 immune checkpoint antibodies. Annals of Oncology (2015) 26: 2375-2391.

O'Day SJ, Maio M, Chiarion-Sileni V, Gajewski TF, Pehamberger H, Bondarenko IN, et al. Efficacy and safety of ipilimumab monotherapy in patients with pretreated advanced melanoma: a multicenter single-arm phase II study. Ann Oncol. 2010;21:1712-7.

VV-RIM-00102568-2.0 Approved - 16 Apr 2020 GMT-5:00

O'Brien PC, Fleming TR. A multiple testing procedure for clinical trials. Biometrics 1979; 35(3):549-56

OPDIVO [package insert]. Princeton, NJ: Bristol-Myers Squibb Company; 2015.

Paz-Ares LG, de Marinis F, Dediu M, Thomas M, Pujol JL, Bidoli P, et al. PARAMOUNT: Final overall survival results of the phase III study of maintenance pemetrexed versus placebo immediately after induction treatment with pemetrexed plus cisplatin for advanced non-squamous non-small-cell lung cancer. J Clin Oncol. 2013;31:2895-902.

Paz-Ares LG, et al. Phase 3 study of carboplatin-paclitaxel/nab-paclitaxel with or without pembrolizumab for patients with metastatic squamous non-small cell lung cancer. 2018 ASCO Annual Meeting. Abstract 105.

Reck M, Popat S, Reinmuth N, De Ruysscher D, Kerr KM, Peters S; ESMO Guidelines Working Group. Metastatic non-small-cell lung cancer (NSCLC): ESMO clinical practice guidelines for diagnosis, treatment and follow-up. Ann Oncol. 2014;25 Suppl 3:iii27-39.

Reck M, Rodríguez-Abreu D, Robinson AG, Hui R, Csőszi T, Fülöp A, et al. Pembrolizumab versus chemotherapy for PD-L1-positive non-small-cell lung cancer. N Engl J Med. 2016;375(19):1823-33.

Roach C, Zhang N, Corigliano E, Jansson M, Tolad G, Ponto G, et al. Development of a companion diagnostic PD-L1 immunohistochemistry assay for pembrolizumab therapy in non-small-cell lung cancer. Appl Immunohistochem Mol Morphol. 2016;24:392–7.

Rosell R, Gatzemeier U, Betticher DC, Keppler U, Macha HN, Pirker R, et al. Phase III randomised trial comparing paclitaxel/carboplatin with paclitaxel/cisplatin in patients with advanced non-small-cell lung cancer: a cooperative multinational trial. Ann Oncol. 2002;13:1539-49.

Scagliotti GV, De Marinis F, Rinaldi M, Crinò L, Gridelli C, Ricci S, et al. Phase III randomized trial comparing three platinum-based doublets in advanced non-small-cell lung cancer. J Clin Oncol. 2002;20:4285-91.

Schiller JH, Harrington D, Belani CP, Langer C, Sandler A, Krook J, et al. Comparison of four chemotherapy regimens for advanced non-small-cell lung cancer. N Engl J Med. 2002;346:92-8.

Shimizu T, Seto T, Hirai F, Takenoyama M, Nosaki K, Tsurutani J, et.al. Phase 1 study of pembrolizumab (MK-3475; anti-PD-1 monoclonal antibody) in Japanese patients with advanced solid tumors. Invest New Drugs (2016);34:347-54.

Shimizu T, Yokoi T, Tamaki T, Kibata K, Inagaki N, Nomura S. Comparative analysis of carboplatin and paclitaxel combination chemotherapy schedules in previously untreated patients with advanced non-small cell lung cancer. Oncol Lett. 2013;5:761-7.

Siegel RL, Miller KD, Jemal A. Cancer statistics, 2016. CA Cancer J Clin. 2016;66:7-30.

Slovin SF, Higano CS, Hamid O, Tejwani S, Harzstark A, Alumkal JJ, et al. Ipilimumab alone or in combination with radiotherapy in metastatic castration-resistant prostate cancer: results from an open-label, multicenter phase I/II study. Ann Oncol. 2013;24:1813-21.

Socinski M, Creelan B, Horn L, Reck M, Paz-Are L, Steins M, et al. CheckMate 026: A phase 3 trial of nivolumab vs investigator's choice (IC) of platinum-based doublet chemotherapy (PT-DC) as first-line therapy for stage IV/recurrent programmed death ligand 1 (PD-L1)-positive NSCLC. Annals of Oncology. 2016;27 Suppl 6:LBA7_PR.

Topalian SL, Hodi FS, Brahmer JR, Gettinger SN, Smith DC, McDermott DF, et al. Safety, activity, and immune correlates of anti–PD-1 antibody in cancer. N Engl J Med. 2012;366:2443-54.

Vinay DS, Ryan EP, Pawelec G, Talib WH, Stagg J, Elkord E, et al. Immune evasion in cancer: mechanistic basis and therapeutic strategies. Semin Cancer Biol. 2015;35:S185-98.

Weber JS, Yang JC, Atkins MB, Disis ML. Toxicities of immunotherapy for the practitioner. J Clin Oncol. 2015;33:2092-9.

Wolchok JD, Neyns B, Linette G, Negrier S, Lutzky J, Thomas L, et al. Ipilimumab monotherapy in patients with pretreated advanced melanoma: a randomised, double-blind, multicentre, phase 2, dose-ranging study. Lancet Oncol. 2010;11:155-64.

YERVOY [package insert]. Princeton, NJ: Bristol-Myers Squibb Company; 2017.

YERVOY [Summary of Product Characteristics]. Princeton, NJ: Bristol-Myers Squibb Company; 2016.

Zhao X, Suryawanshi S, Hruska M, Feng Y, Wang X, Shen J, et al. Assessment of nivolumab benefit-risk profile of a 240-mg flat dose relative to a 3-mg/kg dosing regimen in patients with advanced tumors. Ann Oncol. 2017a; doi: 10.1093/annonc/mdx235.

Zhao X, Feng Y, Wang X, Young TC, Maier S, Bello S, et al. Tumor growth dynamic (TGD) modeling and safety analysis of nivolumab (nivo) plus ipilimumab (ipi) in first-line (1L) patients (PTS) with non-small cell lung cancer (NSCLC). ASPCT 2017b Annual Meeting.

Zimmer L, Goldinger SM, Hofmann L, et al. Neurological, respiratory, musculoskeletal, cardiac and ocular side-effects of anti-PD-1 therapy. Eur J Cancer 2016;60 210e225.

22. INVESTIGATOR'S AGREEMENT

I have read the attached protocol: A Two-Part Randomized, Phase 3, Study of Combinations of Cemiplimab (Anti-PD-1 Antibody) And Platinum-Based Doublet Chemotherapy in First-Line Treatment of Patients with Advanced or Metastatic Non-Small Cell Lung Cancer

I agree to comply with the current International Council for Harmonisation Guideline for Good Clinical Practice and the laws, rules, regulations, and guidelines of the community, country, state, or locality relating to the conduct of the clinical study.

I also agree that persons debarred from conducting or working on clinical studies by any court or regulatory agency will not be allowed to conduct or work on studies for the sponsor or a partnership in which the sponsor is involved. I will immediately disclose it in writing to the sponsor if any person who is involved in the study is debarred, or if any proceeding for debarment is pending, or, to the best of my knowledge, threatened.

This document contains confidential information of the sponsor, which must not be disclosed to anyone other than the recipient study staff and members of the IRB/EC. I agree to ensure that this information will not be used for any purpose other than the evaluation or conduct of the clinical investigation without the prior written consent of the sponsor.

___________________________________ ___________________________

(Signature of Investigator) (Date)

____

(Printed Name)

VV-RIM-00102568-2.0 Approved - 16 Apr 2020 GMT-5:00

APPENDIX 1. FACTORS TO CONSIDER IN ASSESSING THE RELATIONSHIP OF ADVERSE EVENTS TO STUDY DRUG AND STUDY CONDUCT

Is there a reasonable possibility that the event may have been caused by the study drug or study conduct?

No:

- Due to external causes such as environmental factors or other treatment(s) being administered
- Due to the patient's disease state or clinical condition
- Do not follow a reasonable temporal sequence following the time of administration of the dose of study drug
- Do not reappear or worsen when dosing with study drug is resumed
- Are not a suspected response to the study drug based upon pre-clinical data or prior clinical data

Yes:

- Could not be explained by environmental factors or other treatment(s) being administered
- Could not be explained by the patient's disease state or clinical condition
- Follow a reasonable temporal sequence following the time of administration of the dose of study drug
- Resolve or improve after discontinuation of study drug
- Reappear or worsen when dosing with study drug is resumed
- Are known or suspected to be a response to the study drug based upon pre-clinical data or prior clinical data

NOTE: This list is not exhaustive.

APPENDIX 2. RESPONSE EVALUATION CRITERIA IN SOLID TUMORS: RECIST GUIDELINE (VERSION 1.1)

This appendix has been excerpted from the revised Response Evaluation Criteria in Solid Tumors (RECIST) guideline (version 1.1; Eisenhauer 2009). For full details pertaining to the RECIST 1.1 criteria, please refer to the publication.

1. Assessment of Tumor Burden Measurable Disease at Baseline

Overall tumor burden must be assessed at baseline and will be used as a comparator for subsequent measurements. Tumor lesions will be characterized as measurable or non-measurable as follows:

Response and progression will be evaluated in this study using the international criteria proposed by the revised RECIST guideline (version 1.1; Eisenhauer 2009). Changes in the largest diameter (unidimensional measurement) of the tumor lesions and the shortest diameter in the case of malignant lymph nodes are used in the RECIST criteria.

1.1. Measurable disease

Tumor lesions must be accurately measured in at least one dimension (longest diameter in the plane of measurement is to be recorded) with a minimum size of

- 10 mm by CT scan (CT scan slice thickness no greater than 5 mm)
- 10 mm caliper measurement by clinical exam (lesions which cannot be accurately measured with calipers should be recorded as non-measurable)
- 20 mm by chest X-ray
- **Malignant lymph nodes:** To be considered pathologically enlarged and measurable, a lymph node must be ≥15 mm (≥1.5 cm) in short axis when assessed by CT scan (CT scan slice thickness recommended to be no greater than 5 mm [0.5 cm]). At baseline and in follow-up, only the short axis will be measured and followed.

1.2. Nonmeasurable disease

All other lesions, including small lesions (longest diameter <10 mm or pathological lymph nodes with ≥10 to <15 mm short axis), are considered nonmeasurable disease. Bone lesions, leptomeningeal disease, ascites, pleural/pericardial effusions, lymphangitis cutis/pulmonitis, inflammatory breast disease, and abdominal masses (not followed by CT, MRI, or positron emission tomography [PET]) are considered as nonmeasurable.

1.2.1. Special Considerations

Bone lesions:

- Bone scan, PET scan or plain films are not considered adequate imaging techniques to measure bone lesions. However, these techniques can be used to confirm the presence or disappearance of bone lesions.

- Lytic bone lesions or mixed lytic-blastic lesions, with identifiable soft tissue components, that can be evaluated by cross sectional imaging techniques such as CT or MRI can be considered as measurable lesions if the soft tissue component meets the definition of measurability described above.
- Blastic bone lesions are non-measurable.

Cystic lesions:

Cystic lesions that meet the criteria for radiographically defined simple cysts should not be considered as malignant lesions (neither measurable nor nonmeasurable) since they are, by definition, simple cysts. "Cystic lesions" thought to represent cystic metastases can be considered as measurable lesions, if they meet the definition of measurability described above. However, if noncystic lesions are present in the same patient, these are preferred for selection as target lesions.

Lesions with prior local treatment:

- Tumor lesions situated in a previously irradiated area, or in an area subjected to other locoregional therapy, are usually not considered measurable unless there has been demonstrated progression in the lesion.

1.3. Methods of Assessment

All measurements must be recorded in metric notation, using calipers if clinically assessed. All baseline evaluations should be performed as closely as possible to the beginning of treatment and never more than 4 weeks before the beginning of the treatment.

The same method of assessment and the same technique should be used to characterize each identified and reported lesion at baseline and during follow-up. Imaging-based evaluation is preferred to evaluation by clinical examination unless the lesion(s) being followed cannot be imaged but are assessable by clinical exam.

- **Clinical lesions**. Clinical lesions will only be considered measurable when they are superficial and ≥10 mm (≥1 cm) in diameter as assessed using calipers (eg, skin nodules).
- **Chest X-ray**. Chest CT is preferred over chest X-ray, particularly when progression is an important endpoint, since CT is more sensitive than X-ray, particularly in identifying new lesions. Lesions on chest X-ray may be considered measurable if they are clearly defined and surrounded by aerated lung.
- **CT and MRI**. CT is the best currently available and reproducible method to measure lesions selected for response assessment. This guideline has defined measurability of lesions on CT scan based on the assumption that CT slice thickness is 5mm or less. When CT scans have slice thickness greater than 5 mm, the minimum size for a measurable lesion should be twice the slice thickness. MRI is also acceptable in certain situations (e.g. for body scans).
- **PET-CT**. At present, the low dose or attenuation correction CT portion of a combined PET-CT is not always of optimal diagnostic CT quality for use with RECIST measurements. However, if the site can document that the CT performed as part of a

VV-RIM-00102568-2.0 Approved - 16 Apr 2020 GMT-5:00

PET-CT is of identical diagnostic quality to a diagnostic CT (with intravenous and oral contrast), then the CT portion of the PET-CT can be used for RECIST measurements and can be used interchangeably with conventional CT in accurately measuring cancer lesions over time. Note, however, that the PET portion of the CT introduces additional data that may bias an investigator if it is not routinely or serially performed.

- **Ultrasound**. Ultrasound is not useful in assessment of lesion size and should not be used as a method of measurement.
- **Endoscopy, laparoscopy**. The utilization of these techniques for objective tumor evaluation is not advised.
- **Tumor markers**. Tumor markers alone cannot be used to assess response.
- **Cytology, histology**. These techniques can be used to differentiate between PRs and CRs in rare cases (eg, residual lesions in tumor types, such as germ cell tumors, where known residual benign tumors can remain).

1.4. Baseline Documentation of Target and Non-Target Lesions

Target lesions: When more than one measurable lesion is present at baseline all lesions up to a maximum of 5 lesions total (and a maximum of 2 lesions per organ) representative of all involved organs should be identified as target lesions and will be recorded and measured at baseline. Target lesions should be selected on the basis of their size (lesions with the longest diameter), be representative of all involved organs, and should lend themselves to reproducible repeated measurements.

Lymph nodes merit special mention since they are normal anatomical structures which may be visible by imaging even if not involved by tumor. Pathological nodes which are defined as measurable and may be identified as target lesions must meet the criterion of a short axis of ≥15 mm by CT scan. Only the short axis of these nodes will contribute to the baseline sum. All other pathological nodes (those with short axis ≥10 mm but <15 mm) should be considered non-target lesions. Nodes that have a short axis <10 mm are considered non-pathological and should not be recorded or followed.

A sum of the diameters (longest for non-nodal lesions, short axis for nodal lesions) for all target lesions will be calculated and reported as the baseline sum diameters. The baseline sum diameters will be used as reference to further characterize any objective tumor regression in the measurable dimension of the disease.

Non-target lesions: All other lesions (or sites of disease) including pathological lymph nodes should be identified as non-target lesions and should also be recorded at baseline. Measurements of these lesions are not required, but the presence, absence, or, in rare cases, unequivocal progression of each should be noted throughout follow-up. In addition, it is possible to record multiple non-target lesions involving the same organ as a single item on the case record form (eg, ‘multiple enlarged pelvic lymph nodes’ or ‘multiple liver metastases’).

1.5. Response Criteria

This section provides the definitions of the criteria used to determine objective tumor response for target and non-target lesions.

1.5.1. Evaluation of Target Lesions

- **Complete Response (CR):** Disappearance of all target lesions. Any pathological lymph nodes (whether target or non-target) must have reduction in short axis to <10 mm (<1 cm).
- **Partial Response (PR):** At least a 30% decrease in the sum of the diameters of target lesions, taking as reference the baseline sum diameters.
- **Progressive Disease (PD):** At least a 20% increase in the sum of the diameters of target lesions, taking as reference the smallest sum on study (this includes the baseline sum if that is the smallest on study). In addition to the relative increase of 20%, the sum must also demonstrate an absolute increase of at least 5 mm (0.5 cm). (**Note:** the appearance of one or more new lesions is also considered progressions).
- **Stable Disease (SD):** Neither sufficient shrinkage to qualify for PR nor sufficient increase to qualify for progressive disease, taking as reference the smallest sum diameters while on study.

Special notes on the assessment of target lesions:

- Lymph nodes: Lymph nodes identified as target lesions should always have the actual short axis measurement recorded and should be measured in the same anatomical plane as the baseline examination, even if the nodes regress to below 10 mm on study. This means that when lymph nodes are included as target lesions, the 'sum' of lesions may not be zero even if CR criteria are met, since a normal lymph node is defined as having a short axis of <10 mm.
- Target lesions that become 'too small to measure': All lesions (nodal and non-nodal) recorded at baseline should have their actual measurements recorded at each subsequent evaluation, even when very small (eg, 2 mm). If the radiologist is able to provide an actual measure, that should be recorded, even if it is below 5 mm. However, when such a lesion becomes difficult to assign an exact measure to then: (i) if it is the opinion of the radiologist that the lesion has likely disappeared, the measurement should be recorded as 0 mm. (ii) if the lesion is believed to be present and is faintly seen but too small to measure, a default value of 5 mm should be assigned (note: in case of a lymph node believed to be present and faintly seen but too small to measure, a default value of 5 mm should be assigned in this circumstance as well). This default value is derived from the 5 mm CT slice thickness (but should not be changed with varying CT slice thickness).
- Lesions that split or coalesce on treatment: When non-nodal lesions 'fragment', the longest diameters of the fragmented portions should be added together to calculate the target lesion sum. Similarly, as lesions coalesce, a plane between them may be maintained that would aid in obtaining maximal diameter measurements of each individual lesion. If the lesions have coalesced such that they are no longer separable, the vector of the longest diameter in this instance should be the maximal longest diameter for the 'coalesced lesion'.

1.5.2. Evaluation of Non-target Lesions

While some non-target lesions may actually be measurable, they need not be measured and instead should be assessed only qualitatively at the time points specified in the protocol.

- **Complete Response:** Disappearance of all nontarget lesions and normalization of tumor marker level. All lymph nodes must be nonpathological in size (<10 mm short axis).
- **Non-CR/Non-PD:** Persistence of one or more nontarget lesion(s) and/or maintenance of tumor marker level above the normal limits.
- **Progressive Disease:** Appearance of one or more new lesions and/or unequivocal progression of existing nontarget lesions. Unequivocal progression should not normally trump target lesion status. It must be representative of overall disease status change, not a single lesion increase.

Although a clear progression of "nontarget" lesions only is exceptional, the opinion of the treating physician should prevail in such circumstances, and the progression status should be confirmed at a later time by the review panel (or investigator).

1.6. New Lesions

The appearance of new malignant lesions denotes disease progression; therefore, some comments on detection of new lesions are important. There are no specific criteria for the identification of new radiographic lesions; however, the finding of a new lesion should be unequivocal: ie not attributable to differences in scanning technique, change in imaging modality or findings thought to represent something other than tumor (for example, some 'new' bone lesions may be simply healing or flare of pre-existing lesions). This is particularly important when the patient's baseline lesions show PR or CR. For example, necrosis of a liver lesion may be reported on a CT scan report as a 'new' cystic lesion, which it is not.

A lesion identified on a follow-up study in an anatomical location that was not scanned at baseline is considered a new lesion and will indicate disease progression. An example of this is the patient who has visceral disease at baseline and while on study has a CT or MRI brain ordered which reveals metastases. The patient's brain metastases are considered to be evidence of progressive disease even if he/she did not have brain imaging at baseline. If a new lesion is equivocal, for example because of its small size, continued therapy and follow-up evaluation will clarify if it truly represents new disease. If repeat scans confirm there is definitely a new lesion, then progression should be declared using the date of the initial scan.

While FDG-PET response assessments need additional study, it is sometimes reasonable to incorporate the use of FDG-PET scanning to complement CT scanning in assessment of progression (particularly possible 'new' disease). New lesions on the basis of FDG-PET imaging can be identified according to the following algorithm:

a. Negative FDG-PET at baseline, with a positive FDG-PET at follow-up is a sign of progressive disease based on a new lesion.
b. No FDG-PET at baseline and a positive FDG-PET at follow-up: If the positive FDG-PET at follow-up corresponds to a new site of disease confirmed by CT, this is progressive disease. If the positive FDG-PET at follow-up is not confirmed as a new

site of disease on CT, additional follow-up CT scans are needed to determine if there is truly progression occurring at that site (if so, the date of progressive disease will be the date of the initial abnormal FDG-PET scan). If the positive FDG-PET at follow-up corresponds to a preexisting site of disease on CT that is not progressing on the basis of the anatomic images, this is not progressive disease.

c. FDG-PET may be used to upgrade a response to a CR in a manner similar to a biopsy in cases where a residual radiographic abnormality is thought to represent fibrosis or scarring. The use of FDG-PET in this circumstance should be prospectively described in the protocol and supported by disease-specific medical literature for the indication. However, it must be acknowledged that both approaches may lead to false-positive CR due to limitations of FDG-PET and biopsy resolution/sensitivity.

Note: A "positive" FDG-PET scan lesion means one which is FDG avid with an uptake greater than twice that of the surrounding tissue on the attenuation corrected image.

1.7. Evaluation of Best Overall Response

The BOR is the best response recorded from the start of the treatment until the end of treatment taking into account any requirement for confirmation. The patient's best response assignment will depend on the findings of both target and non-target disease and will also take into consideration the appearance of new lesions.

Revised RECIST 1.1 (Eisenhauer 2009) is summarized in Table 11.

Table 11: Response According to Revised Response Evaluation Criteria in Solid Tumors (Version 1.1) in Patients with Target (and Non-Target) Lesions

Target Lesions	Non-target Lesions	New Lesions	Overall Response*
CR	CR	No	CR
CR	Non-CR/non-PD	No	PR
CR	Not evaluated	No	PR
PR	Non-CR/non-PD/not evaluated	No	PR
SD	Non-CR/non-PD/not evaluated	No	SD
PD	Any	Yes or no	PD
Any	PD*	Yes or no	PD
Any	Any	Yes	PD

Abbreviations: CR=complete response; PD=progressive disease; PR=partial response; SD=stable disease
*In exceptional circumstances, unequivocal progression in non-target lesions may be accepted as PD.
Note: Patients with a global deterioration of health status requiring discontinuation of treatment without objective evidence of disease progression at that time should be reported as "*symptomatic deterioration.*" Every effort should be made to document the objective progression even after discontinuation of treatment.

1.8. Missing Assessments and Unevaluable Designation

When no imaging/measurement is done at all at a particular time point, the patient is not evaluable (NE) at that time point. If only a subset of lesion measurements are made at an

assessment, usually the case is also considered NE at that time point, unless a convincing argument can be made that the contribution of the individual missing lesion(s) would not change the assigned time point response. This would be most likely to happen in the case of progressive disease. For example, if a patient had a baseline sum of 50 mm with 3 measured lesions and at follow-up only 2 lesions were assessed, but those gave a sum of 80 mm, the patient will have achieved progressive disease status, regardless of the contribution of the missing lesion.

1.9. Best Overall Response: All Time Points

The BOR is determined once all the data for the patient is known. Best response determination in studies where confirmation of CR or PR IS NOT required: Best response in these studies is defined as the best response across all time points (for example, a patient who has SD at first assessment, PR at second assessment, and progressive disease on last assessment has a BOR of PR). When SD is believed to be best response, it must also meet the protocol-specified minimum time from baseline. If the minimum time is not met when SD is otherwise the best time point response, the patient's best response depends on the subsequent assessments. For example, a patient who has SD at first assessment, progressive disease at second and does not meet minimum duration for SD, will have a best response of progressive disease. The same patient lost to follow-up after the first SD assessment would be considered unevaluable.

2.0. Duration of overall response

The duration of overall response is measured from the time measurement criteria are first met for CR/PR (whichever is first recorded) until the first date that recurrent or progressive disease is objectively documented (taking as reference for progressive disease the smallest measurements recorded on study). The duration of overall CR is measured from the time measurement criteria are first met for CR until the first date that recurrent disease is objectively documented.

VV-RIM-00102568-2.0 Approved - 16 Apr 2020 GMT-5:00

APPENDIX 3. RECOMMENDED DOSE MODIFICATION OR DISCONTINUATION AND SUPPORTIVE CARE GUIDELINES FOR SPECIFIC CEMIPLIMAB DRUG-RELATEDADVERSE EVENTS (SEE SECTION 7.5.2 FOR EMERGENCY UNBLINDING)

CONFIDENTIAL

VV-RIM-00102568-2.0 Approved - 16 Apr 2020 GMT-5:00

COLITIS CTCAE v4.0 Grade	Cemiplimab Dosing Management	Action and Guidelines	Diagnostic Considerations
**Grade 1**	No change in cemiplimab dose	• For diarrhea, treat symptomatically (loperamide, oral hydration, electrolyte substitution and ADA colitis diet). Endoscopy is recommended if symptoms persist.<br>• Grade 1 diarrhea that persist for >1 week should be treated with the addition of oral diphenoxylate hydrochloride and atropine sulfate four times daily and budesonide 9 mg daily	All attempts should be made to rule out other causes such as metastatic disease, bacterial or parasitic infection, viral gastroenteritis, or the first manifestation of an inflammatory bowel disease by examination for stool leukocytes, stool cultures, and a Clostridium difficile titer<br><br>If symptoms are persistent And/or severe, endoscopic evaluation should be considered
**Grade 2**	Hold until ≤Grade 1. Resume cemiplimab at same dose level.<br><br>May increase dosing interval by 1 week if it takes more than 4 weeks for toxicities to resolve.	• GI consultation and endoscopy is recommended to confirm or rule out colitis for grade 2 diarrhea that persists >1 week or grade 1-2 diarrhea with rectal bleeding (additional guidelines for the treatment of persistent colitis are provided below).<br>• Grade 2 diarrhea should be treated with the addition of oral diphenoxylate hydrochloride and atropine sulfate four times daily and budesonide 9 mg daily.<br>• Grade 2 diarrhea with diffuse ulceration and bleeding seen on endoscopy may require oral steroids with prolonged taper and represent an increased risk for the development of bowel perforation.<br>• When symptoms improve to grade 1 or less, steroid taper should be started and continued over no less than 4 weeks.<br>• In patients with Grade 2 enterocolitis, cemiplimab should be withheld and anti-diarrheal treatment should be started. If symptoms are persistent for more than one week, systemic corticosteroids should be initiated (e.g., 0.5 mg/kg/day of prednisone or equivalent). When symptoms improve to Grade 1 or less, corticosteroid taper should be started and continued over at least 1 month.	

CONFIDENTIAL

VV-RIM-00102568-2.0 Approved - 16 Apr 2020 GMT-5:00

Colitis Adverse Event Management

COLITIS CTCAE v4.0 Grade	Cemiplimab Dosing Management	Action and Guidelines	Diagnostic Considerations
Grade 3-4	Withhold cemiplimab<br><br>Discontinue if unable to reduce corticosteroid dose to <10 mg per day prednisone equivalent within 12 weeks of toxicity	• Patients with Grade 3 enterocolitis, drug will be permanently discontinued and treatment with systemic corticosteroids should be initiated at a dose of 1 to 2 mg/kg/day of prednisone or equivalent. When symptoms improve to Grade 1 or less, corticosteroid taper should be started and continued over at least 1 month.<br><br>For Grade 3-4 diarrhea (or Grade 2 diarrhea that persists after initial steroid treatment),<br>• Rule out bowel perforation. Imaging with plain films or computed tomography (CT) can be useful.<br>• Consider consultation with gastroenterologist and confirmation biopsy with endoscopy.<br>• Treat with intravenous (IV) steroids (methylprednisolone 125 mg) followed by high-dose oral steroids (prednisone 1-2 mg/kg once per day or dexamethasone 4 mg every 4 hours). When symptoms improve to grade 1 or less, steroid taper should be started and continued over no less than 4 weeks. Taper over 6-8 weeks in patients with diffuse and severe ulceration and/or bleeding.<br>• If IV steroids followed by high-dose oral steroids does not reduce initial symptoms within 48-72 hours, consider treatment with infliximab at 5 mg/kg once every 2 weeks. Discontinue infliximab upon symptom relief and initiate a prolonged steroid taper over 45-60 days. If symptoms worsen during steroid reduction, initiate a retapering of steroids starting at a higher dose of 80 or 100 mg followed by a more prolonged taper and administer infliximab. CAUTION: infliximab is contraindicated in patients with bowel perforation or sepsis.<br>• If symptoms persist despite the above treatment a surgical consult should be obtained.<br>• Permanent discontinuation of cemiplimab for grade 4 colitis	All attempts should be made to rule out other causes such as metastatic disease, bacterial or parasitic infection, viral gastroenteritis, or the first manifestation of an inflammatory bowel disease by examination for stool leukocytes, stool cultures, and a Clostridium difficile titer<br><br>If symptoms are persistent And/or severe, endoscopic evaluation should be considered

CONFIDENTIAL

VV-RIM-00102568-2.0 Approved - 16 Apr 2020 GMT-5:00

Endocrine Adverse Event Management

ENDOCRINE EVENTS CTCAE v4.0 Grade	Cemiplimab Dosing Management	Action and Guidelines	Diagnostic Considerations
**Grade 1-2**<br>Hyperthyroidism<br>Hypothyroidism<br>Thyroid disorder<br>Thyroiditis	No change in cemiplimab dose	• Monitor thyroid function or other hormonal level tests and serum chemistries more frequently (every 3-6 weeks) until returned to baseline values.<br>• Replacement of thyroid hormone or thyroid suppression therapy as indicated.	All attempts should be made to rule out other causes such as brain metastases, sepsis, and/or infection. An endocrinology consultation is recommended
**Grade 3-4**<br>Hyperthyroidism<br>Hypothyroidism<br>Thyroid disorder<br>Thyroiditis	Hold until on stable replacement dose as determined by resolution of symptoms and normalization of hormone levels.	• Consider endocrine consultation.<br>• Rule out infection and sepsis with appropriate cultures and imaging.<br>• Replacement of thyroid hormone or thyroid suppression therapy as indicated.	

CONFIDENTIAL

VV-RIM-00102568-2.0 Approved - 16 Apr 2020 GMT-5:00

Endocrine Adverse Event Management (Cont)

ENDOCRINE EVENTS CTCAE v4.0 Grade	Cemiplimab Dosing Management	Action and Guidelines	Diagnostic Considerations
**Grade 1-4**<br>Adrenal insufficiency<br>Hypophysitis<br>Hypopituitarism<br>Pan-hypopituitarism	Hold until on stable replacement dose as determined by resolution of symptoms and normalization of hormone levels	• Thyroid hormone and/or steroid replacement therapy to manage adrenal insufficiency.<br>• If Grade 1–2 hypophysitis is considered, pituitary gland imaging should be considered (magnetic resonance imaging [MRIs] with gadolinium and selective cuts of the pituitary can show enlargement or heterogeneity and confirm the diagnosis).<br>• Grade 3–4 hypophysitis with clinically significant adrenal insufficiency and hypotension, dehydration, and electrolyte abnormalities (such as hyponatremia and hyperkalemia) constitutes adrenal crisis. Hospitalization and IV methylprednisolone should be initiated.	All attempts should be made to rule out other causes such as brain metastases, sepsis, and/or infection. An endocrinology consultation is recommended

Pneumonitis Adverse Event Management

PNEUMONITIS CTCAE v4.0 Grade	Cemiplimab Dosing Management	Action and Guidelines	Diagnostic Considerations
**Grade 1**	**Consider hold of therapy. cemiplimab may be continued with close monitoring.**	• **Radiologic findings should be followed on serial imaging studies at least every 3 weeks.**<br>• **Monitor for symptoms every 2–3 days.**<br>• **Consider pulmonary consultation and/or bronchoscopy if clinically indicated.**	**All attempts should be made to rule out other causes such as metastatic disease, bacterial or viral infection.**
**Grade 2**	**Hold cemiplimab**	**To rule out other causes such as infection:**<br>• Consider pulmonary consultation with bronchoscopy and biopsy/bronchoalveolar lavage (BAL).<br>• Consider pulmonary function tests.<br>• Follow radiologic findings on serial imaging studies every 1-3 days<br>**If the patient is determined to have study drug associated pneumonitis:**<br>• Monitor symptoms daily; consider hospitalization.<br>• Treat with systemic corticosteroids at a dose of 1–2 mg/kg/day prednisone or equivalent. When symptoms improve to Grade 1 or less, steroid taper should be started and continued over no less than 4 weeks.<br>• Treatment with cemiplimab may be resumed if the event improves to ≤ Grade 1 within 12 weeks and corticosteroids have been reduced to the equivalent of methylprednisolone 10 mg by mouth daily or less. Repeat chest imaging monthly as clinically indicated.<br>**For Grade 2 pneumonitis that improves to ≤Grade 1 within 12 weeks, the following rules should apply:**<br>• First episode of pneumonitis: May increase dosing interval by one week in subsequent cycles.<br>• Second episode of pneumonitis: Discontinue cemiplimab if upon rechallenge the patient develops a second episode of ≥ Grade 2 pneumonitis.	

CONFIDENTIAL

VV-RIM-00102568-2.0 Approved - 16 Apr 2020 GMT-5:00

Pneumonitis Adverse Event Management (Cont)

PNEUMONITIS CTCAE v4.0 Grade	Cemiplimab Dosing Management	Action and Guidelines	Diagnostic Considerations
Grade 3-4	Discontinue cemiplimab	• Consider pulmonary function tests with pulmonary consult.<br>• Bronchoscopy with biopsy and/or BAL is recommended.<br>• Treat with IV steroids (methylprednisolone 125 mg). When symptoms improve to grade 1 or less, a high-dose oral steroid (prednisone 1-2 mg/kg once per day or dexamethasone 4 mg every 4 hours) taper should be started and continued over no less than 4 weeks.<br>• Add prophylactic antibiotics for opportunistic infections<br>• If IV steroids followed by high-dose oral steroids does not reduce initial symptoms within 48-72 hours, treat with infliximab at 5 mg/kg once every 2 weeks. Discontinue infliximab upon symptom relief and initiate a prolonged steroid taper over 45-60 days. If symptoms worsen during steroid reduction, initiate a retapering of steroids starting at a higher dose of 80 or 100 mg followed by a more prolonged taper and administer infliximab.	All attempts should be made to rule out other causes such as metastatic disease, bacterial or viral infection.

CONFIDENTIAL

VV-RIM-00102568-2.0 Approved - 16 Apr 2020 GMT-5:00

Renal Adverse Event Management

RENAL EVENTS CTCAE v4.0 Grade	Cemiplimab Dosing Management	Action and Guidelines	Diagnostic Considerations
Grade 1	Consider withholding cemiplimab if event does not improve with symptomatic treatment	• Provide symptomatic treatment.<br>• Monitor creatinine weekly; when it returns to baseline, resume routine creatinine monitoring per protocol.	All attempts should be made to rule out other causes such as obstructive uropathy, progression of disease, or injury to other chemotherapy agents. A renal consultation is recommended.
Grade 2	Consider withholding cemiplimab	• Systemic corticosteroids at a dose of 1-2 mg/kg/day of prednisone or equivalent may be indicated. When symptoms improve to Grade 1 or less, corticosteroid taper should be started and continued over at least 1 month.<br>• Consider prophylactic antibiotics for opportunistic infections.<br>• Consider renal biopsy.<br>• If elevations persist >7 days or worsen, treat as Grade 4.	
Grade 3-4	Discontinue cemiplimab	• Renal consultation with consideration of ultrasound and/or biopsy as appropriate.<br>• Monitor creatinine daily.<br>• Treat with systemic corticosteroids at a dose of 1–2 mg/kg prednisone or equivalent once per day.<br>• When symptoms improve to grade 1 or less, steroid taper should be started and continued over no less than 4 weeks.<br>• Discontinue cemiplimab if unable to reduce corticosteroid dose for irAEs to ≤10 mg.<br>• cemiplimab treatment may be restarted and the dose modified as specified in the protocol.	

CONFIDENTIAL

VV-RIM-00102568-2.0 Approved - 16 Apr 2020 GMT-5:00

Hematologic Adverse Event Management

Thrombocytopenia CTCAE v4.0 Grade	Cemiplimab Dosing Management	Action and Guidelines	Diagnostic Considerations
Grade 1-2	No change in dose		
Grade 3	No change in dose unless bleeding or >7 days	• Grade 3 drug-related thrombocytopenia >7 days or associated with bleeding requires discontinuation.	
Grade 4	Hold cemiplimab until resolves to ≤ Grade 1. May increase the dosing interval by 1 week.	• Grade 4 drug-related thrombocytopenia >7 days or associated with bleeding requires discontinuation.	

Hematologic Adverse Event Management (Cont)

Neutropenia CTCAE v4.0 Grade	Cemiplimab Dosing Management	Action and Guidelines	Diagnostic Considerations
**Grade 1-3**	No change in dose		
**Grade 4**	Hold until resolves to ≤Grade 1. May increase the dosing interval by 1 week. Discontinue if toxicities do not resolve within 12 weeks.		

Dermatologic Adverse Event Management

SKIN EVENTS CTCAE v4.0 Grade	Cemiplimab Dosing Management	Action and Guidelines	Diagnostic Considerations
Grade 1-2	No change in dose	• Symptomatic treatment should be given such as topical glucocorticosteroids (eg, betamethasone 0.1% cream or hydrocortisone 1%) or urea-containing creams in combination with oral antipruritics (eg, diphenhydramine HCl or hydroxyzine HCl).<br>• Treatment with oral steroids is at investigator discretion for Grade 2 events.	All attempts should be made to rule out other causes such as metastatic disease, infection, or allergic dermatitis.
Grade 3	hold cemiplimab	• Consider dermatology consultation and biopsy for confirmation of diagnosis.<br>• Treatment with oral steroids is recommended, starting with 1 mg/kg prednisone or equivalent once per day or dexamethasone 4 mg four times orally daily. When symptoms improve to Grade 1 or less, steroid taper should be started and continued over no less than 4 weeks.	
Grade 4	Permanently discontinue cemiplimab	• Dermatology consultation and consideration of biopsy and clinical dermatology photograph.<br>• Initiate steroids at 1-2 mg/kg prednisone or equivalent. When symptoms improve to grade 1 or less, steroid taper should be started and continued over no less than 4 weeks.	

CONFIDENTIAL

VV-RIM-00102568-2.0 Approved - 16 Apr 2020 GMT-5:00

Hepatitis Adverse Event Management

Hepatitis CTCAE v4.0 Grade	Cemiplimab Dosing Management	Action and Guidelines	Diagnostic Considerations
Grade 1-2	Withhold cemiplimab if there is a treatment-emergent concurrent elevation of ALT and bilirubin that corresponds to an upward shift of 2 or more grades in both parameters.	• Monitor liver function tests more frequently until returned to baseline values.	All attempts should be made to rule out other causes such as metastatic disease, progressive liver disease, viral hepatitis, alternative drug toxicity, infectious causes and/or myositis.
Grade 3-4	Discontinue cemiplimab when AST or ALT >5.0 times ULN and/or total bilirubin >3.0 times ULN.	• Consider appropriate consultation and liver biopsy to establish etiology of hepatic injury, if necessary.<br>• Treat with high-dose IV glucocorticosteroids for 24–48 hours. When symptoms improve to grade 1 or less, a steroid taper with dexamethasone 4 mg every 4 hours or prednisone at 1–2 mg/kg should be started and continued over no less than 4 weeks.<br>• If serum transaminase levels do not decrease 48 hours after initiation of systemic steroids, oral mycophenolate mofetil 500 mg every 12 hours may be given. Infliximab is not recommended due to its potential for hepatotoxicity.<br>• Several courses of steroid tapering may be necessary as symptoms may worsen when the steroid dose is decreased	

CONFIDENTIAL

VV-RIM-00102568-2.0 Approved - 16 Apr 2020 GMT-5:00

Ophthalmologic (Uveitis) Adverse Event Management

Uveitis CTCAE v4.0 Grade	Cemiplimab Dosing Management	Action and Guidelines	Diagnostic Considerations
Grade 1	Discontinue cemiplimab if symptoms persist despite treatment with topical immunosuppressive therapy	• Evaluation by an ophthalmologist is strongly recommended.<br>• Treat with topical steroids such as 1% prednisolone acetate suspension and iridocyclitics.	All attempts should be made to rule out other causes such as metastatic disease, infection, or other ocular disease (*e.g.*, glaucoma or cataracts).
Grade 2	Discontinue cemiplimab if symptoms persist despite treatment with topical immunosuppressive therapy and do not improve to Grade 1 within the retreatment period OR requires systemic treatment.	• Evaluation by an ophthalmologist is strongly recommended.<br>• Treat with topical steroids such as 1% prednisolone acetate suspension and iridocyclitics.	
Grade 3-4	Discontinue cemiplimab	• Treat with systemic corticosteroids such as prednisone at a dose of 1-2 mg/kg per day. When symptoms improve to ≤Grade 1, steroid taper should be started and continued over no less than 4 weeks	

CONFIDENTIAL

VV-RIM-00102568-2.0 Approved - 16 Apr 2020 GMT-5:00

Nausea and Vomiting Adverse Event Management

Nausea and Vomiting CTCAE v4.0 Grade	Cemiplimab Dosing Management	Action and Guidelines	Diagnostic Considerations
**Grade 1**	No change in dose	• Nausea and vomiting should be treated aggressively, and consideration should be given in subsequent cycles to the administration of prophylactic antiemetic therapy according to standard institutional practice. Patients should be strongly encouraged to maintain liberal oral fluid intake.	
**Grade 2**	Hold until ≤Grade 1. Resume at same dose level. May increase dosing interval by 1 week if it takes more than 4 weeks for toxicities to resolve.		
**Grade 3**	Hold until <Grade 2. May increase dosing interval by 1 week for each occurrence. Discontinue if toxicities do not resolve within 12 weeks.		
**Grade 4**	Off protocol therapy		

CONFIDENTIAL

VV-RIM-00102568-2.0 Approved - 16 Apr 2020 GMT-5:00

APPENDIX 4. CEMIPLIMAB OR PLACEBO PHARMACOKINETIC, IMMUNOGENICITY, AND BIOMARKER SAMPLING SCHEDULE

Study Visit	PK Sampling Time[a]	Anti-Drug Antibody Sampling Time[a]	Serum and Plasma Biomarker Samples Sampling Time[a]
Screening			• Collect on-study or archival (≤5 months old) tumor tissue sample
Cycle 1, day 1	• Pre-dose<br>• End of infusion[c]	• Pre-dose	• Pre-dose<br>• Collect blood sample for DNA
Cycle 2, day 1	• Pre-infusion<br>• End of infusion		• Pre-infusion
Cycle 3, day 1	• Pre-infusion<br>• End of infusion		
Cycle 4, day 1	• Pre-infusion<br>• End of infusion		• Pre-infusion
Cycle 9, day 1	• Pre-infusion	• Pre-infusion	
Cycle 18, day 1	• Pre-infusion<br>• End of infusion	• Pre-infusion	
Cycle 26, day 1	• Pre-infusion		
Cycle 30, day 1	• Pre-infusion<br>• End of infusion		
Cycle 34, day 1	• Pre-infusion		
End of treatment	• Pre-infusion<br>• End of infusion	• Pre-infusion	
Follow-up visit 1	• Collect at visit		• Collect at visit
Follow-up visit 2	• Collect at visit		
Follow-up visit 3	• Collect at visit	• Collect at visit	
At the time of RECIST 1.1-defined progressive disease			• Collect at visit[b]

Abbreviations: DNA=deoxyribonucleic acid; PK=pharmacokinetic; RECIST=Response Evaluation Criteria in Solid Tumors

[a] Pre-dose is defined as before the start of the first cemiplimab infusion. Pre-dose samples may be collected ≤72 hours prior to day 1 dosing. Pre-infusion is defined as before the start of subsequent cemiplimab infusions.

[b] If possible a tumor biopsy should also be collected at this time point (optional)

[c] End of infusion is defined as within 10 minutes after the end of the cemiplimab infusion

CONFIDENTIAL

VV-RIM-00102568-2.0 Approved - 16 Apr 2020 GMT-5:00

SIGNATURE OF SPONSOR'S RESPONSIBLE OFFICERS

(**Scientific/Medical Monitor, Regulatory Representative, Clinical Study Team Lead, and Biostatistician**)

To the best of my knowledge, this report accurately describes the conduct of the study and the data generated.

Study Title: A Two-Part Randomized, Phase 3, Study of Combinations of Cemiplimab (Anti-PD-1 Antibody) And Platinum-Based Doublet Chemotherapy in First-Line Treatment of Patients with Advanced or Metastatic Non-Small Cell Lung Cancer

Protocol Number: R2810-ONC-16113

Protocol Version: R2810-ONC-16113 Amendment 5

See appended electronic signature page

Sponsor's Responsible Scientific/Medical Monitor

See appended electronic signature page

Sponsor's Responsible Regulatory Representative

See appended electronic signature page

Sponsor's Responsible Clinical Study Team Lead

See appended electronic signature page

Sponsor's Responsible Biostatistician

VV-RIM-00102568-2.0 Approved - 16 Apr 2020 GMT-5:00

Signature Page for VV-RIM-00102568 v2.0

ESig Approval	[redacted]-2020 20:55:48 GMT+0000
ESig Approval	[redacted]20 20:58:21 GMT+0000
ESig Approval	[redacted]000
ESig Approval	[redacted]-2020 22:31:31 GMT+0000

Signature Page for VV-RIM-00102568 v2.0 Approved

AMENDMENT HISTORY

Amendment 5

Change	Rationale for Change	Sections Changed
Updated primary endpoint of Part 1 from objective response rate (ORR) to overall survival (OS), made ORR and progression free survival (PFS) key secondary endpoints, and made characterization of duration of response (DOR), pharmacokinetics (PK), and quality of life (QOL) secondary objectives instead of exploratory objectives.<br><br>Added an interim analysis at 83% OS event to Part 1.	The number of patients enrolled at time of halting enrollment into Part 1 of the study may allow Part 1 to support a potential registration application.<br><br>The interim analysis is to allow an earlier assessment of OS in Part 1.	Clinical Study Protocol Synopsis: Objectives, Study Design, Procedures & Assessments, Endpoints, Statistical Plan<br><br>Section 1 Introduction<br><br>Section 2.1 Primary Objectives<br><br>Section 2.2.1 Key Secondary Objectives<br><br>Section 2.2.2 Exploratory Objectives (section deleted)<br><br>Section 3.2.2 Rationale for Endpoints and Objectives<br><br>Section 4.2.2 Key Secondary Endpoint (Part 2) and Primary Endpoint (Part 1)<br><br>Figure 1 Study Flow Diagram – Part 1<br><br>Section 5.2 Planned Interim Analysis<br><br>Section 10.1 Statistical Hypothesis<br><br>Section 10.2 Justification of Sample Size<br><br>Section 10.4.5 Efficacy Analyses<br><br>Section 10.5 Multiplicity Considerations<br><br>Section 10.6 Interim Analyses
Added an interim analysis at 50% OS events to Part 2, in addition to the planned interim analysis at 70% OS events.	To allow an earlier assessment of OS.	Section 5.2 Planned Interim Analysis<br><br>Section 10.6 Interim Analyses

CONFIDENTIAL

VV-RIM-00102568-2.0 Approved - 16 Apr 2020 GMT-5:00

Change	Rationale for Change	Sections Changed
Clarified that best overall response (BOR) is not the key secondary endpoint, but a variable used to assess the key secondary endpoint for Part 2, ORR.	Clarification for consistency with the Section 10 (Statistics) of the protocol.	Clinical Study Protocol Synopsis: Endpoints<br>Section 4.2.2 Key Secondary Endpoints<br>Figure 1 Study Flow Diagram – Part 1<br>Figure 2 Study Flow Diagram – Part 2
Updated PFS to key secondary endpoint for Part 2 instead of co-primary endpoint.	Health Authority feedback. There is a hierarchical testing plan in place, so these designations are appropriate for these endpoints; the analysis plan is unchanged.	Clinical Study Protocol Synopsis: Objectives, Endpoints<br>Section 2.1 Primary Objectives<br>Section 2.2.1 Key Secondary Objectives<br>Section 3.2.2 Rationale for Endpoints and Objectives<br>Section 4.2.1 Primary Endpoint (Part 1 and Part 2)<br>Section 4.2.2 Key Secondary Endpoints<br>Figure 2 Study Flow Diagram – Part 2
Removed amylase and lipase testing.	This testing is not routinely done unless clinically indicated to confirm pancreatitis. It is only valuable when done as clinically indicated.	Table 4 Schedule of Events: Screening Visit Assessments and Procedures<br>Table 5 Schedule of Events: Part 1 Treatment Period Assessments and Procedures<br>Table 6 Schedule of Events: Part 2 Treatment Period Assessments and Procedures<br>Section 8.2.3.6 Laboratory Testing

Change	Rationale for Change	Sections Changed
Included cautionary note regarding the concurrent use of strong inducers or inhibitors of CYP2C8 and CYP3A4 with paclitaxel per approved product labeling for paclitaxel.	Health Authority feedback	Section 7.1.3 Platinum-based Doublet Chemotherapy
Updated language to allow patients to be contacted for follow-up at the time of pre-planned interim and final analyses.	To confirm patient survival if necessary before database lock	Table 7 Follow-up Period Assessments and Procedures (Part 1 and Part 2)
Personnel changes	To reflect changes in study oversight	Title Page
Minor editorial changes	Correction of typographical, grammatical, and formatting errors.	Throughout the document

Amendment 4

Change	Sections Changed
Updated the title so that it describes the revised study design.	Title Page: Study Title<br>Clinical Study Protocol Synopsis: Title<br>Section 22 Investigator's Agreement<br>Signature of Sponsor's Responsible Officers: Study Title
Updated the Introduction with relevant data from recent clinical trials and provided rationale for protocol treatment regimens, utilizing information that was previously in other sections of the protocol, to provide a more complete background and rationale for the protocol. Subheadings were added to provide clarity.	Section 1 Introduction<br>Section 3.2 Rationale
Objectives were added for Part 2. The co-primary objectives for Part 2 are to compare	Clinical Study Protocol Synopsis: Objectives<br>Section 1 Introduction

CONFIDENTIAL

VV-RIM-00102568-2.0 Approved - 16 Apr 2020 GMT-5:00

**Change**	**Sections Changed**
overall survival (OS) and progression free survival (PFS) of cemiplimab/chemo-f with placebo/chemo-f in the first-line treatment of patients with advanced squamous or non-squamous non-small cell lung cancer (NSCLC) irrespective of PD-L1 expression, with a planned interim analysis for OS.	Section 2.1 Primary Objectives<br>Section 3.2.2 Rationale for Endpoints and Objectives<br>Section 5.2 Planned Interim Analysis<br>Section 10.1 Statistical Hypothesis<br>Section 10.4.5 Efficacy Analysis<br>Section 10.6 Interim Analysis (Part 2 Only)
The Objectives and Endpoints were modified to differentiate between Part 1 and Part 2.	Clinical Study Protocol Synopsis: Objectives<br>Clinical Study Protocol Synopsis: Endpoints<br>Section 2 Study Objectives<br>Section 3.2.1 Rationale for Study Design<br>Section 4.2 Primary and Secondary Endpoints<br>Section 5.1 Study Description and Duration
A section for Exploratory Objectives was added to capture many of the objectives for Part 1, which are now descriptive.	Section 2.2.2 Exploratory Objectives (Added)
The Hypothesis Section has been updated to describe the hypothesis for Part 2 of the study, as there is no longer a statistical hypothesis for Part 1, which will be analyzed descriptively.	Section 3.1 Hypothesis
Part 2 randomization was added to the protocol. Randomization is 2:1 cemiplimab plus chemotherapy to saline/dextrose placebo (placebo) plus chemotherapy.	Section 3.2.1 Rationale for Study Design<br>Section 5.1.2 Treatment Period<br>Section 7.1.1 Cemiplimab or Placebo<br>Section 7.5 Method of Treatment Assignment<br>Section 10.2 Justification of Sample Size
The Rationale for Cemiplimab Dose Selection was updated with relevant information and clarifying language to keep section current.	Section 3.2.3 Rationale for Cemiplimab Dose Selection
Removed the statement that chemotherapy is currently recommended as a first-line treatment for advanced or metastatic NSCLC	Section 3.2.5 Rationale for Platinum-Based Chemotherapy as Comparator

CONFIDENTIAL

VV-RIM-00102568-2.0 Approved - 16 Apr 2020 GMT-5:00

Change	Sections Changed
in patients with PD-L1 expression <50% since this is no longer accurate in the US, or in many countries world-wide.	
Language about chemotherapy administration was updated to be consistent with changes made in Amendment 3 Global. Patients on Treatment Arms A and B will receive 4 cycles of chemotherapy (not up to 6), and Patients on Treatment Arm C will receive only 2 cycles.	Section 3.2.5 Rationale for Platinum-Based Chemotherapy as Comparator
Pharmacokinetic Variables and Anti-Drug Variables sections were updated to clarify that only cemiplimab is being measured over time and that blood samples will be collected only from patients on arms receiving cemiplimab (or placebo).	Section 4.3 Pharmacokinetic Variables<br>Section 4.4 Anti-Drug Variables<br>Table 7 Schedule of Events: Follow-Up Period Assessments and Procedures<br>Section 8.2.4.1 Drug Concentration Measurements and Samples<br>Section 8.2.4.2 Anti-Drug Antibody Measurements and Samples
The description of the study was changed to align with updated objectives, updated Figure 1 to visually convey changes to the Part 1 study design, and added a new figure to visually represent the study design for Part 2.	Section 5.1 Study Description and Duration<br>Figure 1 Study Flow Diagram – Part 1<br>Figure 2 Study Flow Diagram – Part 2
PD-L1 stratification is <1% versus 1% to 49% versus ≥50%.	Section 5.1.2 Treatment period<br>Section 10.4.5 Efficacy Analysis
In Part 2, enrollment of patients with squamous NSCLC will be capped at 50% of the total sample size.	Clinical Study Protocol Synopsis: Study Design<br>Section 5.1 Study Description and Duration
In Part 2, PD-L1 expression quotas are as follows:<br>• At least 30% but no more than 40% of patients enrolled must have tumors that express PD-L1 in ≥50% of tumor cells	Clinical Study Protocol Synopsis: Study Design<br>Clinical Study Protocol Synopsis: Population<br>Section 5.1 Study Description and Duration<br>Section 6.2 Study Population

Change	Sections Changed
• Enrollment of patients whose tumors express PD-L1 in <1% of tumor cells will be capped at 30%<br>• Enrollment of patients with tumors that express PD-L1 in <50% of tumor cells is capped at 70%.<br>This approach is adopted to maintain an approximately equal distribution of patients in each PD-L1 stratum.	
The study is double-blinded for Part 2, except for an unblinded pharmacist at each site (study team, site investigative team, and patient will be blinded).	Section 5.1 Study Description and Duration<br>Section 7.5.1 Blinding<br>Section 7.5.2 Emergency Unblinding (Part 2 Only)<br>Section 7.6.1 Packaging, Labeling, and Storage
Modified language describing the PD-L1 assay that will be used to assess PD-L1 expression levels for the study.	Section 5.1.1 Screening<br>Section 8.2.5 Biomarker Procedures
The enrollment goal for Part 2 was added and the justification of the sample size was updated, based on statistical considerations and power calculations for primary objectives and endpoints.	Section 6.1 Number of Patients Planned<br>Section 7.5 Method of Treatment Assignment<br>Section 10.2 Justification of Sample Size
The study population for Part 1 was updated to approximately 200 but no more than 360 patients, as fewer patients are expected to enroll with the addition of Part 2.	Section 6.1 Number of Patients Planned<br>Section 7.5 Method of Treatment Assignment
Tuberculosis testing requirements were updated to state that testing should be done only for high risk individuals at the discretion of the investigator, for consistency across NSCLC program.	Section 6.2.2 Exclusion Criteria, #23<br>Table 4 Schedule of Events: Screening Visit Assessments<br>Section 8.2.3.6 Laboratory Testing
Exclusion criterion #8, "previous treatment with idelalisib at any time" was removed to	Section 6.2.2 Exclusion Criteria, #8

Change	Sections Changed
align with inclusion/exclusion criteria across the NSCLC program.	
Never smokers are no longer excluded.	Clinical Study Protocol Synopsis: Population<br>Section 6.2.2 Exclusion Criteria
Language was added to describe a placebo comparator will be used for cemiplimab in Treatment Arm A, to accommodate the double-blinded design for Part 2.	Section 7.1 Investigational and Reference Treatments<br>Section 7.1.1 Cemiplimab or Placebo<br>Section 7.2 Pre-Treatments<br>Section 7.3.2 Cemiplimab or Placebo as Combination Therapy with Chemotherapy<br>Table 2 Cemiplimab or Placebo Guidelines for Temporary and Permanent Discontinuations for Toxicity<br>Section 7.4.1 Acute Infusion Reactions<br>Section 10.7 Additional Statistical Data Handling Conventions
Because Part 2 is double-blinded, language was added which describes that patients will be unblinded at the time of treatment discontinuation in order to allow patients initially randomized to Treatment Arm B (cemiplimab) the option to cemiplimab beyond progression, as permitted by the study.	Section 7.8 Disease Progression While Receiving Cemiplimab
A new section was added, describing the treatment options at the time of disease progression for patients who are randomized to Treatment Arm A (placebo) during Part 2 of the study	Section 7.9 Disease Progression While Receiving Chemotherapy Plus Placebo (Part 2)
Added clarification that cemiplimab monotherapy for patients on Treatment Arm A is only supplied by the Sponsor for patients enrolled during Part 1 of the study.	Section 7.10 Optional Cemiplimab Monotherapy (Part 1 Only)<br>Section 8.2.2.1 Radiographic Tumor Assessments

CONFIDENTIAL

VV-RIM-00102568-2.0 Approved - 16 Apr 2020 GMT-5:00

Change	Sections Changed
Updated Schedule of Events tables and Appendix 4 so that they are aligned.	Table 5 Schedule of Events (Part 1) Treatment Period Assessments and Procedures<br>Appendix 4 Cemiplimab or Placebo Pharmacokinetic Immunogenicity, and Biomarker Sampling Schedule
A “weight” assessment was added to the Schedule of Events to clarify expectations for study conduct.	Table 5 Schedule of Events (Part 1) Treatment Period Assessments and Procedures
Added international normalized ratio (INR) to prothrombin time (PT)/partial thromboplastin time (PTT) lab assessments to align with Common Terminology Criteria for Adverse Events (CTCAE) grading.	Table 4 Schedule of Events: Screening Visit Assessments<br>Section 8.2.3.6 Laboratory Testing
Added Schedule of Events for Part 2.	Table 6 Schedule of Events: Part 2 Treatment Period Assessments and Procedures (Table Added)
Updated Schedule of Events for follow-up assessments to describe differences between assessments in Part 1 and Part 2.	Table 7 Schedule of Events: Follow-Up Period Assessments and Procedures
Added clarifying language about collecting survival data and treatment status.	Section 8.2.2.4 Survival Data and Treatment Status Collection
Described thyroid-stimulating hormone (TSH) testing requirements for patients on Part 2 of the study.	Section 8.2.3.6 Laboratory Testing
Clarified that there is no longer a statistical hypothesis for Part 1, removed the existing statistical hypothesis, and added a new one to align with co-primary objectives and endpoints for part 2.	Section 10.1 Statistical Hypothesis
Described requirements for stopping enrollment to Part 1 and beginning enrollment in Part 2 of the protocol on a site-by-site basis.	Section 10.2 Justification of Sample Size

VV-RIM-00102568-2.0 Approved - 16 Apr 2020 GMT-5:00

Change	Sections Changed
Clarified that analysis sets will be defined separately for Part 1 and Part 2 of the study and updated the description of each analysis set to align with plans for each part of the study.	Section 10.3.1 Efficacy Analysis Sets<br>Section 10.3.2 Safety Analysis Sets<br>Section 10.4.5 Efficacy Analyses<br>Section 10.4.5.1 Subgroup Analyses
Updated and redefined Multiplicity Considerations to align with new statistical analysis plan for study based on design updates in this amendment.	Section 10.5 Multiplicity Considerations
The figure representing the hierarchical testing strategy for primary and key secondary endpoints was removed, as the analysis depicted will not be used with the addition of Part 2 to the study.	Section 10.5 Multiplicity Considerations
Added section to describe the planned interim analysis for OS in Part 2.	Section 10.6 Interim Analysis (Part 2 Only) (Section Added)
Editorial updates to correct typographical and grammatical errors.	Throughout the protocol

Amendment 3 Japan

Change	Sections Changed
Updated inclusion criterion #1 to "Men and women ≥18 years of age (≥20 years of age for Japanese patients)" to be consistent with Japanese regulations.	Clinical Study Protocol Synopsis: Population<br>Section 6.2 Study Population<br>Section 6.2.1 Inclusion Criteria #1
Updated items in Table of Contents to align with company SOP.	Table of Contents

CONFIDENTIAL

VV-RIM-00102568-2.0 Approved - 16 Apr 2020 GMT-5:00

Amendment 3 Global

Change	Sections Changed
Description of Treatment Arm A and B revised to add the clarification that pemetrexed maintenance is changed from optional to mandatory for patients for whom the investigator chooses a pemetrexed-containing doublet, and pemetrexed maintenance (according to local prescribing information and practice guidelines). Description of Treatment Arm C aligned with Section 5.1 Study Design, to clarify that no pemetrexed maintenance is allowed.<br><br>Clarified that for patients on the Treatment Arm A, who experience disease progression during or after administration of chemotherapy will be offered the option to receive cemiplimab 350 mg Q3W for up to 108 weeks, provided they meet specific criteria. No pharmacokinetic or anti-drug antibody assessments will be performed for these patients while receiving optional cemiplimab monotherapy.<br><br>Clarified that in the follow-up period, radiographic assessments for all patients who have not experienced progressive disease should be performed Q9W in year 1 and Q12W in year 2, post original treatment or until RECIST 1.1-defined progressive disease, withdrawal of consent, death, or initiation of another anti-cancer treatment. For Arm B and Arm C patients receiving treatment beyond RECIST 1.1-defined progressive disease, subsequent imaging should be performed within 9 weeks of the initial tumor assessment of progression. For Arm A patients receiving optional cemiplimab monotherapy, radiographic assessments should be performed Q12W.<br><br>Clarified that for Treatment Arm B and C, patients in follow-up may be offered retreatment up to an additional 108 weeks.	Clinical Study Protocol Synopsis: Study Design<br><br>Section 3.2.1 Rationale for Study Design<br><br>Section 5.1 Study Duration and Description<br><br>Section 5.1.2 Treatment Period<br><br>Section 5.1.3 Follow-up<br><br>Section Investigational and Reference Treatment<br><br>Section 7.3.2 Cemiplimab as Combination Therapy with Chemotherapy<br><br>Section 7.3.3 Cemiplimab Monotherapy<br><br>Section 7.8 Disease Progression While Receiving Cemiplimab<br><br>Section 7.10 Optional Cemiplimab Monotherapy<br><br>Table 1 Guidelines for Platinum-Based Doublet Chemotherapy Regimens<br><br>Table 5 Schedule of Events: Treatment Period Assessments and Procedures<br><br>Table 7 Schedule of Events: Follow-Up Period Assessments and Procedures<br><br>Section 8.2.2.1 Radiographic Tumor Assessments

CONFIDENTIAL

VV-RIM-00102568-2.0 Approved - 16 Apr 2020 GMT-5:00

**Change**	**Sections Changed**
Clarified that Treatment Arm B and C patients in follow-up who are offered a retreatment up to an additional 108 weeks may be required to sign a separate informed consent form (ICF) per local regulatory requirements<br>Clarified in Table 5 that there will be a continuous collection of concomitant medicine and AE information.	
Other Secondary Objective revised as follows:<br>Changed from: To assess the predictive utility of baseline PD-L1 tumor expression levels on indicators of clinical response using PD-L1 assays other than the clinical trial assay.<br>New text: To assess the predictive utility of baseline PD-L1 tumor expression levels on indicators of clinical response.<br>Added the following in the list of Other Secondary Objectives: Tumor mutation burden to be assessed using the Foundation Medicine "FoundationOne®" panel, sample permitting.	Clinical Study Protocol Synopsis: Objectives<br>Section 2.2 Secondary Objectives<br>Section 2.2.2 Other Secondary Objectives
Study flow diagram revised to reflect the changes in this amendment.	Figure 1 Study Flow Diagram
Gemcitabine option for platinum based doublet was deleted and chemotherapy was changed from 4 to 6 cycles to 4 cycles.	Table 1 Guidelines for Platinum-Based Doublet Chemotherapy Regimens<br>Section 3.2.5 Rationale for Platinum-Based Chemotherapy as Comparator
Inclusion criterion #2 revised to add stage IIIC patient eligibility for the study and clarified that histologic diagnosis of non-small cell lung cancer (NSCLC) may be confirmed by the central laboratory.<br>Guidance on tumor biopsy sites added to the inclusion criterion #3.	Clinical Study Protocol Synopsis: Study Population<br>Section 6.2 Study Population<br>Section 6.2.1 Inclusion Criteria #2 and #3

**Change**	**Sections Changed**
Clarified that all patients will have tumor evaluated for epidermal growth factor receptor (EGFR) mutations, anaplastic lymphoma kinase (ALK) rearrangement, and C-ros oncogene receptor tyrosine kinase (ROS1) fusions confirmed by a central laboratory (exclusion criterion #3)<br>Exclusion criteria revised based on a health authority feedback<br>The following text was added to exclusion criterion #10: Known active hepatitis B (known positive result) or hepatitis C (known positive result) and known quantitative hepatitis C virus (HCV) RNA results greater than the lower limits of detection of the assay).<br>Clarified that tuberculosis testing is for potential patients in tuberculosis-endemic areas (exclusion criterion #23).<br>Following Criteria added:<br>Criteria added: #24. History of previous organ transplant, including stem cell allograft<br>#25. Meeting the comparator products contraindicated criteria as listed in local labeling. Investigators should review the current label in their local database.	Section 6.2.2 Exclusion Criteria #3, #10, #20, #23, #24 and #25
Deleted "standard-of-care" wording from when mentioned in regards to platinum based chemotherapy(ies).	Section 7 Study Treatments<br>Throughout the protocol
Revised the following text: Cemiplimab is manufactured by Regeneron Pharmaceuticals, Inc.	Section 7.1.1 Cemiplimab
Clarified that premedications are not required prior to the first administration of cemiplimab. And pretreatment with vitamin supplementation is to start within 3 days of	Section 7.2 Pre-treatments

VV-RIM-00102568-2.0 Approved - 16 Apr 2020 GMT-5:00

Change	Sections Changed
randomization for patients with non-squamous NSCLC.	
The text describing cemiplimab revised to say: Each vial will contain cemiplimab at a concentration of 50 mg/mL.	Section 7.1 Investigational and Reference Treatment<br>Section 7.6.1 Packaging, Labeling, and Storage
Any treatment administered from the time of informed consent until 90 days (instead of 30 days) after the last study treatment will be considered concomitant medication.<br>Following text deleted: Optional pemetrexed maintenance therapy is only permitted for patients with non-squamous NSCLC in Treatment Arm A for whom the investigator chooses a pemetrexed-containing doublet.	Section 7.7 Concomitant Medications<br>Section 7.7.2 Permitted Medications and Procedures
Clarified that tumor tissue samples will also be tested for EGFR mutations and ALK translocations as well as for ROS1 fusions by a central laboratory, unless this testing has already been performed and the results are available from other Regeneron NSCLC immunotherapy studies.<br>The following text was deleted: Additional testing may be employed to determine PD L1 expression levels by utilizing another PD-L1 immunohistochemistry (IHC) assay in the same tissue specimens. This approach may provide a better understanding of the performance of PD L1 expression level as a predictive biomarker of response to REGN2810. Of special interest is PD L1 expression across different cell types including tumor cells, stroma cells, and infiltrating immune cells.<br>The following text was revised as: If possible a tumor tissue biopsy sample should be obtained at the time of RECIST 1.1-defined progressive disease.	Section 8.2.1 Procedures Performed only at Screening/Baseline Visit<br>Section 8.2.5 Biomarker Procedures<br>Table 4 Schedule of Events: Screening Visit Assessments and Procedures

Change	Sections Changed
Following text deleted: Brain scans during the treatment and follow-up periods should be performed as clinically indicated except for patients with a history of metastases, who should have surveillance imaging approximately every 18 weeks for year 1 and every 24 weeks for year 2 or sooner, if indicated.<br>Electrocardiogram (ECG) text revised to delete: Heart rate will be recorded from the ventricular rate, and the PR, QRS, RR, QT, and QT corrected for Bazett's formula intervals will also be recorded.	Section 8.2.2.1 Radiographic Tumor Assessments<br>Section 8.2.3.5 12-Lead Electrocardiogram
Addition of text, based on request by the regulatory authority to evaluate ADA in ipilimumab, as follows:<br>If necessary, these samples may also be used for ADA assessments of ipilimumab.	Section 8.2.4.2 Anti-Drug Antibody Measurements and Samples
The following text was added: irAEs of any grade in patients previously treated with PI3-Kinase inhibitor.	Section 9.3.3 Adverse Events of Special Interest
Acute infusion reactions are defined as any adverse event (AE) that occurs during the infusion or within 1 day hours after the infusion is completed, instead of within 2 hours.	Section 7.4.1 Acute Infusion Reactions<br>Section 9.3.4 Infusion Reactions
Changed REGN2810 to the generic name, cemiplimab, and minor editorial changes	Throughout the protocol
Pharmacokinetic (PK)/anti-drug antibody (ADA) sample collection time points revised. Pharmacokinetics and immunogenicity of cemiplimab are now well understood and described in more than 500 patients from various studies using population PK modeling. Therefore, sparse PK/ADA sampling can be applied to describe PK characteristics in individual patients and in	Appendix 4 Cemiplimab Pharmacokinetic, Immunogenicity, and Biomarker Sampling Schedule

VV-RIM-00102568-2.0 Approved - 16 Apr 2020 GMT-5:00

Change	Sections Changed
the overall population of patients with NSCLC.	

Amendment 2 VHP

Change	Sections Changed
The current text of Key Secondary Endpoint “A patient who has not died will be censored at the last known date of contact" has been revised to "A patient who is lost to follow-up will be censored at the last date that the patient was known to be alive", following European Union (EU) regulatory review.	Section 4.2.2 Key Secondary Endpoint
The current text : “cemiplimab C2P1 drug product is supplied as a sterile liquid solution of 5.6 or 7.4 mL in a 10 or 20 mL glass vial for IV administration” is revised to “cemiplimab C2P1 drug product is supplied as a sterile liquid solution of 5.6 mL in a 10 mL glass vial for IV administration.”	Section 7.1.1 Cemiplimab
The current text : “Each vial will contain a withdrawable volume of 5 or 7 mL of cemiplimab at a concentration of 50 mg/mL” revised to “Each vial will contain a withdrawable volume of 5 mL of cemiplimab at a concentration of 50 mg/mL.” as requested following EU regulatory review.	Section 7.1.1 Cemiplimab<br>Section 7.6.1 Packaging, Labeling, and Storage
Revised the protocol to include regular thyroid function testing for Arm B (cemiplimab + SOC) as requested following EU regulatory review.	Table 5 Schedule of Events: Treatment Period Assessments and Procedures<br>Section 8.2.3.6 Laboratory Testing.

VV-RIM-00102568-2.0 Approved - 16 Apr 2020 GMT-5:00

Amendment 2

**Change**	**Sections Changed**
Removed "pembrolizumab" from the protocol, because it is not part of the study, as requested following European Union (EU) regulatory review.	Clinical Study Protocol Synopsis: Study Design<br>Section 7.4.1.2 Termination of the Infusion
Clarified that treatment with pemetrexed is chosen by the investigator (patients are not randomized to it), as requested following EU regulatory review.	Section 3.2.1 Rationale for Study Design<br>Figure 1 Study Flow Diagram (footnotes)<br>Section 5.1.2 Treatment Period<br>Section 7.7.2 Permitted Medications and Procedures
Clarified that the use of the PD-L1 IHC 22C3 pharmDx assay for decisions regarding treatment with cemiplimab is considered investigational	Clinical Study Protocol Synopsis: Target Population<br>Section 3.2.2 Rationale for Endpoints and Objectives<br>Section 5.1.1 Screening<br>Section 6.2 Study Population
Revised the exclusion criteria concerning human immunodeficiency virus (HIV), hepatitis B virus (HBV) and hepatitis C virus (HCV) to clarify that patients with uncontrolled infection are excluded, but patients with controlled infection are permitted, as requested following EU regulatory review.	Section 6.2.2 Exclusion Criteria (combined criteria #10 and #11, and deleted previous #11)
Added tuberculosis screening, as requested following EU regulatory review.	Section 6.2.2 Exclusion Criteria (new criterion number 23)<br>Table 4 Schedule of Events: Screening Visit Assessments and Procedures<br>Section 8.2.1 Procedures Performed Only at the Screening/Baseline Visit<br>Section 8.2.3.6 Laboratory Testing
Added testing for amylase and lipase, as requested following EU regulatory review.	Table 4 Schedule of Events: Screening Visit Assessments and Procedures

VV-RIM-00102568-2.0 Approved - 16 Apr 2020 GMT-5:00

Change	Sections Changed
	Table 5 Schedule of Events: Treatment Period Assessments and Procedures<br>Section 8.2.3.6 Laboratory Testing
Added that testing for thyroid-stimulating hormone will be every 9 weeks after treatment with ipilimumab is complete, as requested following EU regulatory review.	Table 5 Schedule of Events: Treatment Period Assessments and Procedures<br>Section 8.2.3.6 Laboratory Testing

Amendment 1

Change	Sections Changed
The following text added:<br>The study population is limited to previous and current smokers as the benefit of PD-1 blockade has not been shown in non-smokers, likely due to the lower mutational burden in this population.	Section 3.2.1 Rationale for Study Design
Enrollment to Arm C does not need to be halted pending the results of this additional safety evaluation, but stopping criteria related to this evaluation was incorporated into the protocol.	Section 5.1.4 Description of Study Stopping Rules
The following changes were implemented:<br>• Revised AST or ALT >5 times upper limit of normal (ULN) and/or total bilirubin >3 times ULN as a DLT criterion.<br>• Added Grade 4 anemia<br>• DLT criteria include all Grade 3 immune-related AEs (irAEs). Any Grade 3 irAE which would result in permanent discontinuation of the cemiplimab/ipi/chemo-l (or permanent discontinuation of ipilimumab), as outlined in the "Dosing Discontinuation" subsection of Section 7.3.1 of the protocol, or which would result in permanent discontinuation of cemiplimab, as outlined in the	Section 5.1.5 Dose Limiting Toxicities

VV-RIM-00102568-2.0 Approved - 16 Apr 2020 GMT-5:00

Change	Sections Changed
guidelines in Appendix 3 of the protocol, will be considered a DLT. This specifically includes the following Grade 3 irAEs: pneumonitis, colitis/diarrhea, nephritis.	
Inclusion criterion #2 revised to specify as follows:<br>Patients with histologically or cytologically documented squamous or non-squamous NSCLC with stage IIIB disease who are not candidates for treatment with definitive concurrent chemoradiation or patients with stage IV disease if they have not received prior systemic treatment for recurrent or metastatic NSCLC are eligible.	Inclusion Criteria Section 6.2.1 #2
Exclusion criterion #3 modified to add a statement indicating that all patients should have tumor evaluated for EGFR mutations, ALK rearrangement, and ROS1 fusions.<br>Exclusion criterion #5 modified as: A history of radiation pneumonitis in the radiation field is permitted as long as pneumonitis resolved ≥6 months prior to enrollment.	Exclusion Criteria Section 6.2.2 #3, and #5
A written informed consent should be obtained for treatment beyond radiologic disease progression, acknowledging that this practice is not considered standard in the treatment of cancer.	Section 14.2 Informed Consent
The diagram for colitis adverse event management in Appendix 3 has been modified to require permanent discontinuation of cemiplimab for grade 4 colitis	Appendix 3 Recommended Dose Modification or Discontinuation and Supportive Care Guidelines for Specific cemiplimab Drug Related Adverse

STATISTICAL ANALYSIS PLAN
VERSION: 1.0

Clinical Study Protocol Title: A Two-Part Randomized, Phase 3 Study of Combinations of Cemiplimab (Anti-PD-1 Antibody) and Platinum-Based Doublet Chemotherapy in First-Line Treatment of Patients with Advanced or Metastatic Non-Small Cell Lung Cancer

Compound: Cemiplimab (REGN2810 / anti-PD-1 mAb)

Protocol Number: R2810-ONC-16113

Clinical Phase: Phase 3

Sponsor: Regeneron Pharmaceuticals, Inc.

Study Biostatistician: [redacted]

Clinical Trial Manager: [redacted]

Study Medical Director: [redacted]

Version/Date: Original Statistical Analysis Plan / Apr 17 , 2020

Document's type	Document Reference	Effective Date	
Standard	BDM-STD-STA4-2.1	July 2, 2013	

The approval signatures below indicate that these individuals have reviewed the Statistical Analysis Plan (SAP) and agreed on the planned analysis defined in this document for reporting.

Study Biostatistician

See appended electronic signature page

[redacted]

Study Medical Director

See appended electronic signature page

[redacted]

Head of BDM or designee

See appended electronic signature page

[redacted]

Document's type	Document Reference	Effective Date	
Standard	BDM-STD-STA4-2.1	July 2, 2013	

TABLE OF CONTENTS

ABBREVIATIONS AND DEFINITION 6
1. OVERVIEW 9
1.1. Background/Rationale 9
1.2. Study Objectives 11
1.2.1. Primary Objectives 11
1.2.2. Secondary Objectives 11
1.2.3. Modifications from the Statistical Section in the Final Protocol 12
1.2.4. Revision History for SAP Amendments 12
2. INVESTIGATION PLAN 13
2.1. Study Design and Randomization 13
2.2. Sample Size and Power Considerations 13
2.3. Study Plan 14
3. ANALYSIS POPULATIONS 16
3.1. The Full Analysis Set (FAS) 16
3.2. The Safety Analysis Set (SAF) 16
3.3. The PK Set (PKS) 16
3.4. The Anti-Drug Antibody Set (ADA) 16
3.5. The Dose Limiting Toxicity Set (DLT) 16
4. ANALYSIS VARIABLES 17
4.1. Demographic and Baseline Characteristics 17
4.2. Baseline Tumor Characteristics 17
4.3. Medical History 17
4.4. Prior / Concomitant Medication and Procedure 18
4.5. Treatment Exposure 18
4.6. Efficacy Variable 19
4.6.1. Primary Efficacy Variable (Parts 1 and 2) 19
4.6.2. Key Secondary Efficacy Variable (Parts 1 and 2) 19
4.6.3. Other Secondary Efficacy Variable(s) 20
4.7. Safety Variables 20
4.7.1. Adverse Events and Serious Adverse Events 20
4.7.2. Adverse Events of Special Interest 21

4.7.3. Laboratory Safety Variables 21
4.7.4. Vital Signs 22
4.7.5. 12-Lead Electrocardiography (ECG) 22
4.7.6. Physical Examination Variables 22
4.8. Pharmacokinetic and Anti-Drug Antibody Variables (ADA) 22
4.9. Quality-of-Life Variables 23
5. STATISTICAL METHODS 24
5.1. Patient Disposition 24
5.2. Protocol Deviations 24
5.3. Demographics and Baseline Characteristics 24
5.4. Medical History 25
5.5. Pre-treatment and Concomitant Medications/Procedures 25
5.5.1. Prior-treatment Medication/Procedure 25
5.5.2. Concomitant Medications/Procedures 25
5.5.3. New anti-cancer Medications/Procedures 25
5.6. Extent of Study Treatment Exposure and Compliance 25
5.6.1. Measurement of Compliance 25
5.6.2. Exposure to Investigational Product 26
5.7. Analyses of Efficacy Variables 26
5.7.1. Primary Statistical Hypothesis 26
5.7.2. Primary and Key Secondary Efficacy Analyses 27
5.7.3. Subgroup Analysis 28
5.7.4. Adjustment for Multiple Comparison 28
5.8. Analysis of Safety Data 30
5.8.1. Adverse Events 31
5.8.2. Clinical Laboratory Measurements 32
5.8.3. Analysis of Vital Signs 32
5.8.4. Analysis of 12-Lead ECG 33
5.8.5. Physical Exams 33
5.9. Analysis of Pharmacokinetic and Antibody Data 33
5.9.1. Analysis of Pharmacokinetic Data 33
5.9.2. Analysis of Anti-Drug Antibody Data 33

5.9.3. Analysis of Exploratory Biomarker Data 33
6. DATA CONVENTIONS 34
6.1. Definition of Baseline for Efficacy/Safety Variables 34
6.2. Data Handling Convention for Efficacy Variables 34
6.3. Data Handling Convention for Missing Data 34
6.4. Visit Windows 34
6.5. Unscheduled Assessments 35
7. INTERIM ANALYSIS 36
8. SOFTWARE 37
9. REFERENCES 38

LIST OF TABLES

Table 1: Summary of Decision Guidance for Part 1 Analysis 29
Table 2: Alpha Spending for Analysis of OS in Part 1 36
Table 3: Alpha Spending for Analysis of OS in Part 2 36

ABBREVIATIONS AND DEFINITION

ACTH	Adrenocorticotropic hormone
ADA	Anti-drug antibody
ADL	Activities of daily living
AE	Adverse event
AESI	Adverse event of special interest
ALK	Anaplastic lymphoma kinase
ALT	Alanine aminotransferase
Anti-CTLA-4	Anti-cytotoxic T-lymphocyte-associated antigen 4
Anti-PD-1	Anti-programmed death 1
Anti-PD-L1	Anti-programmed death ligand 1
AST	Aspartate aminotransferase
ATC	Anatomical therapeutic chemical
BOR	Best overall response
BUN	Blood urea nitrogen
CI	Confidence interval
C_{eoi}	Concentration at end of infusion
Chemo-f	Chemo-full; 4 cycles of chemotherapy
Chemo-l	Chemo-limited; 2 cycles of chemotherapy
CSR	Clinical study report
CNS	Central nervous system
CR	Complete response
CRF	Case report form
CT	Computed tomography
DoR	Duration of response
ECG	Electrocardiogram
ECOG	East Cooperative Oncology Group
EGFR	Epidermal growth factor receptor
EORTC QLQ-C30	European Organization for Research and Treatment of Cancer Quality of<br>Life Questionnaire Core 30
EORTC QLQ-LC13	European Organization for Research and Treatment of Cancer Quality of<br>Life Questionnaire Lung Cancer 13

EOS	End of study
EOT	End of treatment
FAS	Full analysis set
GFR	Glomerular filtration rate
HR	Hazard ratio
ICH	International Conference on Harmonisation
irAE	Immune-related adverse event
ipi	Ipilimumab
irAE	Immune-related adverse event
IRB	Institutional Review Board
IWRS	Interactive web response system
LDH	Lactate dehydrogenase
MAA	Marketing authorization application
MedDRA	Medical Dictionary for Regulatory Activities
NCI-CTCAE	National Cancer Institute-Common Terminology Criteria for Adverse Events
NE	Not evaluable
NR	Not reported
NSCLC	Non-small cell lung cancer
ORR	Objective response rate
OS	Overall survival
PD	Progression Disease
PFS	Progression-free survival
PK	Pharmacokinetics
PR	Partial response
PT	Preferred term
QOL	Quality of life
Q2W	Every 2 weeks
Q3W	Every 3 weeks
Q6W	Every 6 weeks
Q9W	Every 9 weeks
Q12W	Every 12 weeks
Q18W	Every 18 weeks
Q24W	Every 24 weeks

RECIST	Response Evaluation Criteria in Solid Tumors
RNA	Ribonucleic acid
ROS1	C-ros oncogene receptor tyrosine kinase
ROW	Rest of world
SAE	Serious adverse event
SAF	Safety analysis set
SAP	Statistical analysis plan
SAS	Statistical Analysis Systems (software)
SD	Stable disease
SI	Standard international
SOC	System organ class
TEAE	Treatment-emergent adverse event
t_{eoi}	Time of end of infusion
TSH	Thyroid-stimulating hormone
ULN	Upper limit of normal
WHODD	World Health Organization drug dictionary
WBC	White blood cell

1. OVERVIEW

The purpose of this statistical analysis plan (SAP) is to ensure the credibility of the study results by pre-specifying the statistical approaches for the analysis of study data prior to database lock. The SAP is intended to be a comprehensive and detailed description of the strategy and statistical methods to be used in the analysis of data for the R2810-ONC-16113 study.

There are two parts in Study R2810-ONC-16113. Part 1 includes first line non-small cell lung cancer (NSCLC) patients whose tumors express PD-L1 in <50% of tumor cells. Part 2 includes first line NSCLC patients irrespective of PD-L1 expression . Although enrolled in the same clinical trial, the statistical analyses of the two parts will be conducted independently and summarized separately, except for safety data analyses may be combined at a later stage when deemed appropriate.

This plan may be revised during the study to accommodate protocol amendments and/or to make changes to adapt to unexpected issues in study execution and/or data that affect planned analyses. The final plan, if revised, will document all changes and be issued prior to data lock.

1.1. Background/Rationale

Lung cancer is one of the most commonly diagnosed cancers and is the leading cause of cancer related mortality worldwide. NSCLC accounts for 80% to 85% of all lung cancers and is composed of several histopathological subtypes, the most common of which are adenocarcinoma (40% to 60%) and squamous cell carcinoma (30%). The majority of patients with NSCLC are often found to have advanced cancer at the time of diagnosis.

Systemic therapy with platinum-based doublet regimens, with or without maintenance therapy, was until recently the standard of care for first line treatment of patients with advanced NSCLC whose tumors do not have an epidermal growth factor receptor (EGFR) mutation, an anaplastic lymphoma kinase (ALK) mutation, or a C-ros oncogene receptor tyrosine kinase (ROS1) fusion. Despite chemotherapy therapy, patients with metastacic NSCLC have a median overall survival (OS) of up to 8 to 12 months and a 5 year survival rate of approximately 18%.

Programmed cell death-1/PD-L1 inhibitors have also been investigated in combination with standard-of-care chemotherapy regimens for patients with non-squamous and squamous NSCLC. An accelerated approval of pembrolizumab in combination with carboplatin and pemetrexed was granted for first-line non-squamous NSCLC patients in the US in 2017. In August 2018, pembrolizumab in combination with pemetrexed and carboplatin was approved by the FDA as first-line treatment of patients with metastatic non-squamous NSCLC. In September 2018, pembrolizumab in combination with pemetrexed and carboplatin was approved by the EMA for the same indication. In December 2018, the FDA approved atezolizumab in combination with bevacizumab, paclitaxel and carboplatin in patients with metastatic non-squamous NSCLC. In October 2018, FDA approved pembrolizumab in combination with carboplatin and either paclitaxel or nab-paclitaxel for the first-line treatment of patients with metastatic squamous NSCLC. These approvals changed landscape of immunotherapy in NSCLC.

Cemiplimab (REGN2810) is a human IgG4 monoclonal antibody (mAb) to the PD-1 receptor that blocks PD-1/PD-L1 mediated T cell inhibition. Cemiplimab is being evaluated in more than 20

Phase 1-3 clinical studies. Regeneron has initiated Phase 2 and Phase 3 trials of cemiplimab in several indications: advanced or metastatic PD-L1 positive NSCLC, advanced cutaneous squamous cell carcinoma (CSCC), advanced basal cell carcinoma (BCC), and metastatic or recurrent cervical cancer after platinum-based therapy. In 2018, Cemiplimab was approved in the United States (US) by the FDA as cemiplimab-rwlc for the treatment of patients with metastatic cutaneous squamous cell carcinoma (CSCC) or locally advanced CSCC who are not candidates for curative surgery or curative radiation. In August 2019, same indication was approved in the European Union (EU).

The original study design was a randomized, global, open-label, phase 3 study comparing cemiplimab plus 4 cycles of platinum based doublet chemotherapy (chemo-f) combination therapy (cemiplimab/chemo-f, Arm B) and cemiplimab plus 2 cycles of platinum based doublet chemotherapy (chemo-l) plus ipilimumab combination therapy (cemiplimab/chemo-l/ipi, Arm C) versus platinum-based doublet chemotherapy (Arm A) in the first-line treatment of patients with advanced or metastatic, squamous or non-squamous NSCLC whose tumors express PD-L1 in <50% of tumor cells and who have received no prior systemic treatment for their advanced disease. Patients were centrally randomized via interactive web response system (IWRS) in a 1:1:1 ratio to one of the three treatment arms. The main objective of the original study was to determine if cemiplimab /chemo-f or cemiplimab /chemo-l/ipi improves progression free survival (PFS) over standard-of-care platinum-based doublet chemotherapy in this patient population. Additional objectives included characterization of overall survival (OS), objective response rate (ORR), safety, pharmacokinetics (PK), and quality of life (QOL). Due to the increasing recognition that the addition of CTLA-4 blockade to PD-1/PD-L1 blockade has not robustly improved benefit, and the desire to explore cemiplimab/chemo-l/ipi in a more limited way, Protocol Amendment 4 was issued where Part 2 of the study was added.

Per Protocol Amendment 4, this study was modified to a two-part randomized, global study. Patients enrolled per the original criteria were designated as enrolled in Part 1. Part 1 halted patient enrollment in August 2019 and randomized 323 patients by the end of August 2019. Part 1 was to assess whether cemiplimab/chemo-f or cemiplimab/chemo-l/ipi improves ORR, PFS, or OS over platinum-based doublet chemotherapy in patients with PD-L1 <50%. Part 2 was added via Amendment 4 to compare the OS and PFS of cemiplimab/chemo-f with saline/dextrose placebo (placebo/chemo-f) in the 1st line treatment of patients with advanced NSCLC irrespective of PD-L1 expression. Part 2 is a double-blind placebo controlled 2-arm study for first-line NSCLC patients regardless of the level of PD-L1. Patients will be randomized in a 2:1 fashion to cemiplimab plus platinum-based doublet chemotherapy (cemiplimab/chemo-f) or placebo plus platinum-based doublet chemotherapy (placebo/chemo-f) to maximize the number of patients benefiting from the combination treatment and minimize barriers to patient enrollment.

Per Protocol Amendment 5, the primary objective of Part 1 is updated to compare if cemiplimab/ chemo-f and cemiplimab /chemo-l/ipi improve OS over platinum-based doublet chemotherapy. The key secondary objectives are updated to compare the PFS and ORR of cemiplimab/chemo-f and cemiplimab/chemo-l/ipi versus chemo-f in the first-line treatment of patients with advanced squamous or non-squamous NSCLC. Additionally, PFS is updated to key secondary endpoint in Part 2 instead of co-primary endpoint, as requested by the FDA.

1.2. Study Objectives

1.2.1. Primary Objectives

Part 1: The primary objective is to compare the OS of cemiplimab/chemo-f and cemiplimab/chemo-l/ipi versus chemo-f in the first-line treatment of patients with advanced squamous or non-squamous NSCLC with PD-L1 tumors express <50%.

Part 2: The primary objective is to compare the OS of cemiplimab/chemo-f with placebo/chemo-f in the first-line treatment of patients with advanced squamous or non-squamous NSCLC irrespective of PD-L1 tumors expression.

1.2.2. Secondary Objectives

The key secondary objectives of the study are the following:

- Part 1: To compare the PFS and ORR of cemiplimab/chemo-f and cemiplimab/chemo-l/ipi versus chemo-f in the first-line treatment of patients with advanced squamous or non-squamous NSCLC with tumors express PD-L1 in <50% of tumor cells .
- Part 2: To compare the PFS and ORR of cemiplimab/chemo-f versus placebo/chemo-f in the first-line treatment of patients with advanced squamous or non-squamous NSCLC irrespective of PD-L1 expression.

The other secondary objectives of the study are the following:

- Parts 1 and 2: To evaluate the safety and tolerability of cemiplimab/chemo-l/ipi and/or cemiplimab/chemo-f compared to platinum-based doublet chemotherapy or placebo/chemo-f
- Parts 1 and 2: To evaluate the duration of response (DoR) of cemiplimab/chemo-l/ipi and/or cemiplimab/chemo-f compared to platinum-based doublet chemotherapy or placebo/chemo-f in the first-line treatment of patients with advanced squamous or non-squamous NSCLC.
- Parts 1 and 2: To compare QoL in patients with advanced squamous or non-squamous NSCLC receiving cemiplimab/chemo-l/ipi and/or cemiplimab/chemo-f compared to platinum-based doublet chemotherapy or placebo/chemo-f
- Part 1: To evaluate the OS rate at 12, 18, and 24 months of cemiplimab/chemo-f and cemiplimab/chemo-l/ipi versus chemo-f in the first-line treatment of patients with advanced squamous or non-squamous NSCLC with PD-L1 expression in <50% of tumor cells.
- Part 2: To evaluate the OS rate at 12, 18, and 24 months of cemiplimab/chemo-f versus placebo/chemo-f in the first-line treatment of patients with advanced squamous or non-squamous NSCLC irrespective of PD-L1 expression.
- Parts 1 and 2: To assess immunogenicity as measured by anti-drug antibodies (ADAs) for cemiplimab

- Parts 1 and 2: To assess the predictive utility of baseline PD-L1 tumor expression levels on clinical response
- Part 1: to characterize the PK of cemiplimab when administered in combination with ipilimumab or in combination with platinum-based doublet chemotherapy.
- Part 2: To characterize the PK of cemiplimab when administered in combination with platinum-based doublet chemotherapy
- Parts 1 and 2: To conduct exposure-response (E-R) analyses for relevant biomarkers (exploratory PK/pharmacodynamic analyses) and E-R analyses for safety and efficacy endpoints, as appropriate
- Parts 1and 2: Tumor mutation burden as assessed by the Foundation Medicine "FoundationOne®" panel, sample permitting.

1.2.3. Modifications from the Statistical Section in the Final Protocol

None.

1.2.4. Revision History for SAP Amendments

This is the first version of the SAP, based on the protocol R2810-ONC-16113 Amendment 5.

2. INVESTIGATION PLAN

2.1. Study Design and Randomization

This is a two-part, multicenter, global, randomized, phase 3 study. Part 1 is a multicenter, multinational, randomized, active-controlled, open-label, stratified (baseline histology [non-squamous versus squamous] and PD-L1 level [<1% versus 1% to 24% versus 25% to <50%]) study design. After a screening phase of up to 4 weeks, patients will be centrally randomized via interactive web response system (IWRS) in a 1:1:1 ratio to one of the three treatment arms and treated open-label for up to 108 weeks.

Part 2 is a multicenter, multinational, randomized, placebo-controlled, double-blind, 2-parallel-group, stratified (baseline histology [non-squamous versus squamous] and PD-L1 level [<1% versus 1% to 49% versus ≥ 50%]) study design. After a screening phase of up to 4 weeks, patients will be centrally randomized via interactive web response system (IWRS) in a 2:1 ratio to one of the two treatment arms and treated double blind up to 108 weeks.

2.2. Sample Size and Power Considerations

Part 1:

Enrollment in Part 1 at any individual site was stopped in August, 2019 when the amended study authorizing Part 2 of the study receives IRB/EC approval and all other Health Authority requirements were met. At that time, enrollment in Part 2 began. By the end of August 2019, Part 1 randomized a total of 323 patients with approximately 215 patients in each of cemiplimab combination versus the chemotherapy comparison (cemiplimab/chemo-f vs. chemo-f or cemiplimab/chemo-l/ipi vs. chemo-f). The sponsor assumes Part 1 has the same hypothesis assumptions for PFS and OS as assumed for Part 2.

The sponsor assumes a median OS of 12 months for patients treated with chemotherapy , and a hazard ratio of 0.65 in OS for each cemiplimab combination versus the chemotherapy comparison. Patients will be randomized in a 1:1:1 ratio to the chemo-f arm, the cemiplimab/chemo-f versus cemiplimab/chemo-l/ipi. Under these assumptions and for each cemiplimab combination comparison, 151 deaths (70% out of 215 randomized in one of the comparisons) are needed to yield approximately 74% power to detect statistical significance in OS by stratified Log-Rank testing procedure with an overall 2-sided type one error of 0.0499, given 2-sided type one error of 0.0001 will be spent on administrative analysis for PFS and ORR per invesstigator assessment.

The sponsor assumes a median PFS of 6 months for patients treated with chemotherapy alone, and a hazard ratio of 0.6667 in PFS for each cemiplimab combination versus the chemotherapy comparison. Under these assumptions and for each cemiplimab combination versus chemotherapy comparison, 161 PFS events (75% out of 215 randomized patients in one of the cemiplimab combination comparisons) are needed to yield approximately 73% power to detect statistical significance in PFS by stratified Log-Rank testing procedure with an overall 2-sided type one error of 0.0499 given 2-sided type one error of 0.0001 will be spent on administrative analyses for PFS and ORR per investigator assessment.

Part 2:

Historically, in patients with Stage IIIB or Stage IV NSCLC treated with cisplatin or carboplatin + paclitaxel Q3W, the median PFS has ranged from approximately 2.7 to 6.4 months (El-Shenshawy 2012, Rosell 2002, Scagliotti 2002, Schiller 2002, Shimizu 2013, Socinski 2016); the median OS has ranged from approximately 11.3 to 20.9 months (Socinski 2016, De Lima Lopes 2018, Borghaei 2017, Brahmer 2017, Gandhi 2018, Paz-Ares 2018).

Based on those historical data, the sponsor assumes a median OS of 12 months for patients treated with chemotherapy plus placebo, and a hazard ratio of 0.65 in OS between cemiplimab/chemo-f arm and placebo/chemo-f arm. Patients will be randomized in a 2:1 ratio to the cemiplimab/chemo-f arm (Treatment Arm B) versus the placebo/chemo-f arm (Treatment Arm A). Under these assumptions, 291 deaths are needed to yield approximately 93% power to detect statistical significance in OS at a 2-sided Type I error level or 0.05 between the two treatment arms.

Considering an enrollment period of 14 months (11 patients per month for the first 4 months, 26 patients per month for month 5 to 8, 50 patients per month afterwards), an approximately 24-month follow-up period for OS after completion of enrollment, and 10% annual dropout, the enrollment of approximately 450 randomized patients is needed to obtain 291 deaths for the final analysis of OS.

Based on above historical data, the sponsor assumes a median PFS of 6 months for patients treated with chemotherapy plus placebo, and a hazard ratio of 0.6667 in PFS between the cemiplimab/chemo-f arm and placebo/chemo-f arm. With these assumptions and at two-sided 0.05 alpha level, the power for analysis of PFS will be 90% or more if it is performed after 288 or more PFS events is observed.

EAST® 6.4.1 is used for sample size calculation.

2.3. Study Plan

Part 1:

Part 1 consists of the 3 periods: screening, treatment, and follow-up. Patients will undergo a screening evaluation within 28 days prior to randomization. After screening, eligible patients will be randomized to one of the three treatment arms. Treatment may be discontinued early due to RECIST 1.1-defined progressive disease , withdrawal of consent, death, unacceptable toxicity, initiation of another anti-cancer treatment, or, for patients in Treatment Arms B and C, in specific instances of confirmed complete response (CR) or partial response (PR). After discontinuing study treatment, patients will enter the follow-up period.

For patients on Treatment Arm A who experience disease progression while on chemotherapy or after administration of chemotherapy will be offered the option to receive cemiplimab 350 mg Q3W for up to 108 weeks, provided they meet specific criteria. After the active portion of the study is complete, all patients will be followed for survival.

Because this is the first study of cemiplimab /chemo-l/ipi, safety data from the first 10 patients treated with cemiplimab /chemo-l/ipi in Treatment Arm C will be reviewed after these patients have had 4 weeks of follow-up following the first dose of cemiplimab /chemo-l/ipi. The data will be reviewed at a meeting of the Independent Data Monitoring Committee (IDMC). If 2 or more dose-limiting toxicities occur in the first 10 patients treated in Treatment Arm C, enrollment for this treatment arm will be stopped temporarily and will be restarted only after a formal safety review.

The approximate duration of the active study assessments for each patient, excluding screening, will be 24 to 32 months. For Treatment Arms B and C, this encompasses 25 months of study treatment plus 7 months of follow-up. For patients in Treatment Arm A, this encompasses 4 cycles of treatment (and pemetrexed maintenance, if appropriate) and radiographic tumor assessments for up to 2 years, if there is no disease progression; otherwise patients in Treatment Arm A advance to the 7 months follow up period.

Part 2:

Part 2 consists of the 3 periods: screening, treatment, and follow-up. After screening, eligible patients will be randomized to Treatment Arm A (placebo/chemo-f) or Treatment Arm B (cemiplimab/chemo-f). Treatment may be discontinued early due to RECIST 1.1-defined progressive disease, withdrawal of consent, death, unacceptable toxicity, or initiation of another anti-cancer treatment. After discontinuing study treatment, patients will enter the follow-up period. Patients in Part 2 of the study will be unblinded to treatment at the time of progressive disease defined by RECIST1.1 criteria or discontinuation from the study. Patients on Treatment Arm A who experience disease progression will enter follow up and other anticancer treatment should be considered at this time at the discretion of the investigator. Patients will be unblinded to treatment at the time of progressive disease defined by RECIST1.1 criteria or discontinuation from study.

For the entire study, baseline radiographic tumor assessments should also be performed within 28 days prior to randomization. Radiographic tumor assessments will be obtained Q9W beginning at week 9 (day 63 ± 5 days) during year 1 and Q12W beginning at week 55 (first radiographic tumor assessment in year 2 performed at end of week 54) during year 2, until blinded independent review committee (IRC) assessed RECIST 1.1-defined progressive disease (PD), withdrawal of consent, death, or initiation of another anti-cancer treatment. Patients who discontinue for reasons other than progression who are not attending treatment visits may have radiographic tumor assessments between Q9W and Q12W until RECIST 1.1-defined progressive disease, withdrawal of consent, death, or initiation of another anti-cancer treatment.

For the entire study, radiographic tumor assessments will be assessed by Investigators (INV) and IRC using RECIST 1.1 criteria. Progressive disease per INV assessment will be used for clinical management. Tumor burden assessments per blinded IRC assessment will be used for evaluation of efficacy endpoints.

3. ANALYSIS POPULATIONS

In accordance with guidance from the International Conference of Harmonization of Technical Requirements for Registration of Pharmaceuticals for Human Use (ICH) guideline ICH E9 Statistical Principles for Clinical Trials (ICH, 1998), the following analysis populations will be used for relevant analyses.

3.1. The Full Analysis Set (FAS)

The FAS will be defined separately for Parts 1 and 2. The FAS includes all patients to whom study treatment has been assigned by randomization in each study part. FAS is essentially the intention to treat population (ITT). Per ITT principle, patients will be analyzed according to the treatment and stratification factors they have been assigned to during the randomization. FAS will be used for all baseline, demographic, and efficacy endpoints.

3.2. The Safety Analysis Set (SAF)

The study part specific SAF includes all randomized patients who received any study treatment (at least one dose of any component of study treatment in a combination therapy) in each study part. Patients will be analyzed according to the study treatment received. Treatment administration and all clinical safety variables will be analyzed using the SAF.

3.3. The PK Set (PKS)

The PK analysis set includes all randomized patients (safety population) who receive cemiplimab and who have at least 1 non-missing cemiplimab concentration assay result following the first dose of cemiplimab up to the end of the study.

3.4. The Anti-Drug Antibody Set (ADA)

The study part specific ADA analysis set includes all treated patients who received any study drug and have at least 1 non-missing post-baseline ADA assay result following the first dose of study drug.

3.5. The Dose Limiting Toxicity Set (DLT)

The DLT analysis set includes all Part 1 patients treated with cemiplimab/chemo-l/ipi who are DLT evaluable, defined as the patients who completed the DLT observation period and those patients who discontinued early due to the development of a DLT. This population will be used for the assessment of DLTs. The patients will be analyzed as treated.

4. ANALYSIS VARIABLES

4.1. Demographic and Baseline Characteristics

The following study part specific demographic variables will be summarized:

- Age at screening in years (quantitatively and quantitatively: <65, >=65)
- Sex (Male, Female)
- Race (White, Black, Asian, and Other)
- Ethnicity (Hispanic/Latino or not)
- Geographic region (Europe, Asia, and ROW)
- Weight (kg)
- Height (cm)
- ECOG performance status (0, 1)
- Smoking Status

4.2. Baseline Tumor Characteristics

The following study part specific analyses will be provided:

- Histology (non-squamous, squamous)
- Histologic grade (well differentiated, moderately differentiated, poorly, undifferentiated)
- Mutation status of Biomarkers (i.e. EGFR, ALK, ROS1, BRAF, NRAS, C-KIT)
- Level of PD-L1 expression (quantitative and qualitative variable [Part 1: <1% versus 1%-24% versus 25%-49%; Part 2: <1% versus 1% to 49% versus ≥ 50%])
- Cancer stage at screening (Part 1: Stage IIIB, IIIC, or Stage IV; Part 2: Stage IIIB versus Stage IV)
- TNM stage at initial diagnosis and screening
- Time from initial diagnosis to randomization in months (= [Date of randomization - Date of initial diagnosis]/30.4375)
- Time from most recent recurrence/relapse to randomization in months (= [Date of randomization - Date of most recent recurrence/relapse]/30.4375)

4.3. Medical History

The study part specific medical history will be coded to a Preferred Term (PT) and associated primary System Organ Class (SOC) according to the latest available version of Medical Dictionary for Regulatory Activities (MedDRA®).

4.4. Prior / Concomitant Medication and Procedure

Medications/Procedures will be recorded from the day of informed consent until the end of study (EOS) visit. Medications will be coded to the anatomical therapeutic chemical (ATC) level 2 (therapeutic main group) and ATC level 4 (chemical/therapeutic subgroup), according to the latest available version of WHO Drug Dictionary (WHODD).

Prior medications/procedures: medications taken or procedures performed prior to administration of the study drug, including prior cancer related systemic therapy, prior cancer related surgery, and prior cancer related radiotherapy.

Concomitant medications/procedures: medications taken or procedures performed from initiation of study treatment until 90 days after the last study treatment will be considered concomitant treatment. This includes medications that were started before the initiation of study treatment and are ongoing during the study, as well as any therapies started in the follow-up period to treat a study-drug-related AE. All concomitant treatments must be recorded in the study case report form (CRF) with the generic name, dose, dose unit, frequency, indication, and start/stop date, as appropriate.

4.5. Treatment Exposure

Cemiplimab

Duration of treatment exposure to cemiplimab (in weeks) is calculated as the minimum of

- [date of last dose – date of first dose + 21] / 7 or
- [date of clinical data cut-off or date of death – date of first dose + 1] / 7

The actual dose intensity = total dose received / duration of treatment exposure (week)

The relative dose intensity = actual dose intensity / planned dose intensity, where planned dose intensity (in week) = planned dose / 3.

Ipilimumab

Duration of treatment exposure to Ipilimumab (in weeks) is calculated as the minimum of

- [date of last dose – date of first dose + 42 days per Q6 weekly dosing schedule] / 7 or
- [date of clinical data cut-off or date of death – date of first dose + 1] / 7

The actual dose intensity = total dose received / duration of treatment exposure (week)

The relative dose intensity = actual dose intensity / planned dose intensity, where planned dose intensity (in week) = planned dose / 6.

Carboplatin, Cisplatin, Paclitaxel, and Pemetrexed

Duration of treatment exposure (in weeks) is calculated as the minimum of

- [date of last dose – date of first dose + 21 days] / 7 or
- [date of clinical data cut-off or date of death – date of first dose + 1] / 7

The actual dose intensity = total dose received / duration of treatment exposure (week)

The relative dose intensity = actual dose intensity / planned dose intensity, where planned dose intensity (in week) = planned dose / 3.

Gemcitabine

Duration of treatment exposure to Gemcitabine (in weeks) calculated as the minimum of

- [date of last dose – date of first dose + 7 or 14 days] / 7 , or
- [date of clinical data cut-off or date of death – date of first dose + 1] / 7

The actual dose intensity = total dose received / duration of treatment exposure (week)

The relative dose intensity = actual dose intensity / planned dose intensity, where planned dose intensity (in week) = planned dose / (3/2).

4.6. Efficacy Variable

4.6.1. Primary Efficacy Variable (Parts 1 and 2)

OS is defined as the time from the date of randomization to the date of death due to any cause. All deaths due to any cause occurring on or before cut-off date in the FAS will be used in the OS analysis. If a patient is not known to have died or is lost to follow up at the time of analysis cutoff date, the one will be censored at the last date that the patient was known to be alive.

4.6.2. Key Secondary Efficacy Variable (Parts 1 and 2)

The key secondary endpoints are PFS and ORR per RECIST 1.1.

PFS is defined as the time from randomization to the date of the first documented tumor progression, or death due to any cause, whichever occurred earlier. Patients will be censored according to the rules listed below:

- Patients who do not have a documented tumor progression or death will be censored on the date of their last evaluable tumor assessment.
- Patients who do not have a documented tumor progression or death before initiation of new anti-tumor therapy will be censored on the date of their last evaluable tumor assessment prior to or on the date of new anti-tumor therapy.
- Patients who withdraw consent before taking any study treatment, therefore there is no post baseline tumor assessment, will be censored at the date of randomization.
- Patients who do not have any evaluable tumor assessments after randomization and do not die will be censored on the date of randomization.

ORR is defined as the proportion of patients with a best over response (BOR) of confirmed complete response (CR) or partial response (PR) in the FAS. Patient(s) without baseline tumor assessment, or with either unknown or missing BOR will be included in the denominator and will be counted as non-responder(s).

4.6.3. Other Secondary Efficacy Variable(s)

DoR is defined as the time from date of first documented response of CR or PR to the date of first documented PD or death due to any cause, whichever occurred earlier. Patients continuing without PD or death due to any cause will used the same censoring rule as PFS as stated in Section 4.6.2.

OS rate at a landmark (12, 18, and 24 months) is defined as the K-M estimated probability of patients who survived due to any cause at the landmark after randomization. All deaths occurring on or before a landmark are counted as failures. If a patient is not known to have died or is lost to follow up at the landmark, the one will be censored at the last date that the patient was known to be alive.

BOR is defined as the best overall response as deterimined by the IRC or INV per RECIST 1.1, between the date of randomization and the date of first documented tumor progression or the date of subsequent anti-cancer therapy, whichever occurred earlier. BOR of CR or PR must be confirmed by a subsequent evaluation of overall response of CR or PR at time points at least 4 weeks apart. BOR of SD must have met the response SD criteria at least once ≥39 days (6 weeks*7 days/week -3 days) after randomization. BOR of (early) PD does not require confirmation. BOR for patients who do not have any post-baseline tumor assessment will be not evaluable (NE).

QoL are measured by the EORTC QLQ-C30 and EORTC QLQ-C13 (Appendix 1 and 3 of protocol, also details in Section 4.9).

4.7. Safety Variables

Patient safety will be assessed through the collection of reported adverse events (AEs), clinical laboratory data, vital signs, ECG and physical exam. Unless otherwise noted, the baseline value is defined as the last available value before the first dose of study treatment.

4.7.1. Adverse Events and Serious Adverse Events

An Adverse Event (AE) is any untoward medical occurrence in a patient or clinical investigation patient administered a pharmaceutical product and which does not necessarily have to have a causal relationship with this treatment. A Serious Adverse Event (SAE) is an AE that is classified as serious according to the criteria specified in the protocol.

The investigator (or designee) will seek information on AEs at each patient contact, and record all AEs that occur from the time the informed consent is signed until 90 days after the last dose of study drug, or until the patient commences another anticancer systemic therapy, whichever is earlier. Prior to initiation of study drug, only the following categories of AEs should be reported on the AE CRF:

- SAEs
- Non-SAEs associated with a protocol-mandated intervention (eg, AEs related to an invasive procedure such as a biopsy)

All AEs after initiation of study treatment and until 90 days after the last dose of study treatment, regardless of relationship to study treatment, will be reported on the AE CRF. Additionally, any SAE or AE that the investigator believes may be related to study drug and that occurs later than

90 days after last dose of study drug, or after the patient has commenced another anticancer systemic therapy (whichever is earlier) should be reported.

The relationship of AEs to study drug will be assessed by the investigator and be determined based on protocol specified criteria.

All adverse events are to be coded to a Preferred Term (PT) and associated primary System Organ Class (SOC) according to the latest available version of Medical Dictionary for Regulatory Activities (MedDRA).

Laboratory results, vital signs, or ECG abnormalities are to be recorded as AEs if they are medically relevant: symptomatic, requiring corrective therapy, leading to treatment discontinuation and/or fulfilling a seriousness criterion.

4.7.2. Adverse Events of Special Interest

An AE of special interest (AESI) must be reported within 24 hours of identification. AEs of special interest for this study include:

- Grade 2 or greater infusion-related reactions
- Grade 2 or greater allergic/hypersensitivity reactions
- Grade 3 immune-related AEs (irAEs)
- An irAE of any grade in a patient previously treated with a PI 3-K inhibitor

Note: An irAE can occur shortly after the first dose, several months after the last dose of treatment, or any time in-between. All AEs of unknown etiology associated with drug exposure should be evaluated to determine possible immune etiology. If an irAE is suspected, efforts should be made to rule out neoplastic, infectious, metabolic, toxin or other etiologic causes prior to labeling an AE as an irAE.

4.7.3. Laboratory Safety Variables

The clinical laboratory data consists of serum chemistry, hematology, and urinalysis. Clinical laboratory values will be converted to standard international (SI) units and grouped by function in summary tables. Conventional unit may be provided. Functions are defined as follows:

Blood Chemistry: Sodium; Phosphorus; Alanine aminotransferase (ALT); Potassium; Glucose; Aspartate aminotransferase (AST); Chloride; Albumin; Total bilirubin; Bicarbonate; Creatinine; Alkaline phosphatase (ALP); Calcium; Blood urea nitrogen (BUN); Lactate dehydrogenase (LDH); Magnesium; Uric acid; Total protein

Hematology: Hemoglobin; Hematocrit; Red blood cells (RBCs); White blood cells (WBCs); Red cells indices; Platelet count; Differential: Neutrophils, Lymphocytes, Monocytes, Basophils, Eosinophils

Other lab tests: Prothrombin time (PT); Partial thromboplastin time (PTT); Thyroid-stimulationg hormone (TSH); Free T4; Pregnancy test

4.7.4. Vital Signs

Vital signs will be collected according to Study Schedule of protocol: Body temperature (^{o}C); Resting systolic blood pressure and diastolic blood pressure (mmHg); Pulse rate (beats/minute); Respiratory rate (breaths/minute).

4.7.5. 12-Lead Electrocardiography (ECG)

A standard 12-lead ECG will be performed at time points according to Study Schedule of the protocol. The ECG strips or reports will be retained with the source. The ECG will be reviewed by the investigator (paper or electronic tracing) and will be available for comparison with subsequent ECGs by the investigator: PR Interval (msec); QRS Interval (msec); QT Interval (msec); Ventricular Rate (BPM)

Any ECG finding that is judged by the investigator as a clinically significant change (worsening) compared to the baseline value will be considered an AE, recorded, and monitored.

4.7.6. Physical Examination Variables

A thorough complete or limited physical examination will be performed at visits specified in Study Schedule of the protocol. Care should be taken to examine and assess any abnormalities that may be present, as indicated by the patient's medical history.

Complete physical examination will include examination of skin, head, eyes, nose, throat, neck, joints, lungs, heart, pulse, abdomen (including liver and spleen), lymph nodes, and extremities, as well as a brief neurologic examination.

Limited physical examination will include lungs, heart, abdomen, and skin.

4.8. Pharmacokinetic and Anti-Drug Antibody Variables (ADA)

Cemiplimab concentrations in serum of patients randomized to the cemiplimab treatment group will be assessed at multiple time points throughout the treatment and follow-up periods. PK variables may include, but are not limited to, the following:

- C_{trough} – pre-infusion concentration
- C_{eoi} – concentration at end-of-infusion
- t_{eoi} – time of end-of-infusion

Immunogenicity assessment will include the assessment of anti-drug antibody (ADA) and the assessment of neutralizing ADA (NAb). ADA variables will be measured in samples from patients randomized to the REGN2810 treatment group and will include status (positive or negative) and titer as follows:

- Total number of patients whose response in the ADA assay is negative at all times
- Total number of patients whose response in the ADA assay is positive at any time
- Pre-existing immunoreactivity – defined either as a positive ADA assay response at baseline with all post-treatment ADA results negative, or a positive assay response at baseline with all post-treatment ADA assay responses less than 9-fold over baseline titer levels

- Treatment emergent – defined as any positive response post-treatment when baseline results are negative
- Treatment boosted – defined as any post treatment positive ADA assay response that is greater than 9-fold over the baseline titer level when baseline is positive in the ADA assay.
- Titer category is defined based on values as (titer value category):
  - Low (titer < 1,000)
  - Moderate (1,000 ≤ titer ≤ 10,000)
  - High (titer > 10,000)

4.9. Quality-of-Life Variables

QOL are measured by the EORTC QLQ-C30 and EORTC QLQ-C13 (Appendix 1 and 3 of protocol).

The EORTC QLQ-C30 was developed to assess the quality of life of cancer patients. It contains 30 items and measures five functional dimensions (physical, role, emotional, cognitive, and social), three symptom items (fatigue, nausea/vomiting, and pain), six single items (dyspnea, sleep disturbance, appetite loss, constipation, diarrhea, and financial impact) and global health and quality of life. The global health and quality of life scale uses a 7-point scale scoring with anchors (1=very poor and 7=excellent); the other items are scored on a 4-point scale (1=not at all, 2=a little, 3= quite a bit, 4=very much).

The EORTC QLQ-LC13, a supplemental lung cancer-specific module used in combination with QLQ-C30, comprises multi-item and single-item measures of lung cancer-associated symptoms (cough, hemoptysis, dyspnea, and site-specific pain) and treatment -related symptoms (sore mouth, dysphagia, peripheral neuropathy and alopecia). It is scored on a 4-point scale (1=not at all, 2=a little, 3=quite a bit, 4=very much).

5. STATISTICAL METHODS

For continuous variables, descriptive statistics will include the following: the number of patients reflected in the calculation (n), mean, median, standard deviation, minimum, and maximum. In addition, 25th percentile and 75th percentile will be provided.

For categorical or ordinal endpoint, frequencies and percentages will be displayed for descriptive statistics. The denominator will be determined by the analysis set used for the summary.

All analyses for each study part will be performed separately.

5.1. Patient Disposition

The following will be summarized and listed by treatment arms based on part specific FAS:

- Number of screened patients: met the inclusion criteria regarding the target indication and signed the ICF
- Number of randomized patients: received a randomization number
- Number of patients in the SAF
- A listing of patients who were treated but not randomized, randomized but not treated, or randomized but not treated as randomized. Summary table will be provided if applicable.
- Number of patients who discontinued treatment and the primary reasons for treatment discontinuation
- Number of patients who discontinued study and the primary reasons for study discontinuation
- A listing of patients prematurely discontinued from study treatment and study, along with reasons for discontinuation

Listing of patient disposition will include dates of the first and the last study treatment administration, date of end of treatment and end of study visits, and reasons for treatment and study discontinuations.

5.2. Protocol Deviations

Protocol deviations will be recorded in a separate protocol deviation definition document which includes a listing of all patients with protocol deviations and the reasons for deviation. The major protocol deviations, such as violation of inclusion/exclusion criteria; post-enrollment deviations which will impact assessment of efficacy or safety endpoints, will be determined before database lock and be summarized by treatment group.

5.3. Demographics and Baseline Characteristics

Demographics and baseline characteristics variables will be summarized and listed by study part specific FAS. Summaries will be provided by treatment arm and for all patients by study part.

Assessment made before the first dose of study treatment will be used as baseline measurements for statistical analysis and reporting unless otherwise specified.

The part specific randomization stratification factors (histology and PD-L1 level) collected in the clinical database will be cross-classified, tabulated and listed against the stratification factors used to randomize patients (from the IWRS data) based on the FAS.

5.4. Medical History

Medical history will be summarized and listed based by study part specific FAS. Summaries will be provided by treatment arms and for all patients and will be sorted by decreasing frequency of SOC followed by PT.

Listing of medical history will include verbatim term, SOC, PT, and start and end dates.

5.5. Pre-treatment and Concomitant Medications/Procedures

5.5.1. Prior-treatment Medication/Procedure

Pre-treatment medications/procedures will be summarized and listed based on by study part specific FAS. Summaries will be provided by treatment arm and for all patients.

Number and proportion of patients with prior cancer related medication, prior cancer related surgery, and prior cancer related radiotherapy, will be provided, respectively. Prior cancer related medications will be summarized by therapy setting, and by ATC 2 and ATC 4.

Listing of prior cancer related medications will include verbatim term, ATC 2, ATC 4, start date, end date, therapy setting, best response, and date of progression. Listing of prior cancer related surgery will include date of surgery, procedure, reason for surgery/procedure, and surgery location. Listing of prior cancer related radiotherapy will include start date, end date, type of radiation therapy, site of radiation, intent of treatment, and total dose.

5.5.2. Concomitant Medications/Procedures

Concomitant medications/procedures will be summarized and listed by study part specific SAF. Summaries will be provided by treatment arm.

Concomitant medications will be summarized by ATC 2 and ATC 4, sorted by decreasing frequency of ATC2 followed by ATC 4 in investigational drug arm.

Listing of concomitant medications will include drug name (reported), ATC 2, ATC 4, start date, end date, indication, dose, frequency, and route.

5.5.3. New anti-cancer Medications/Procedures

New anti-cancer therapies will be listed by study part specific FAS.

5.6. Extent of Study Treatment Exposure and Compliance

5.6.1. Measurement of Compliance

Compliance with cemiplimab treatment will be calculated as follows:

- Treatment Compliance = (Number of doses of cemiplimab administered during treatment period) / (Number of doses of cemiplimab planned to be administered during treatment period) × 100%,

where temporary dose discontinuation is ignored.

Treatment compliance will be summarized and listed by study part specific FAS. The following will be summarized by treatment arm:

- Number of doses administered
- Number and percentage of patients who have <60%, >=60 to <80%, >=80 to <=100%, and >100% compliance will be summarized for each treatment arm, or by study drugs
- Wrong dose table (if applicable).

5.6.2. Exposure to Investigational Product

Exposure to Investigational Product will be summarized and listed based on SAF by study parts.

Duration of exposure will be summarized by treatment arm for each study part. For cemiplimab, each chemotherapy, and ipilimumab, the following will be summarized by treatment arm:

- Duration of exposure
- Number of doses administered
- Cumulative dose administered
- Actual dose intensity
- Relative dose intensity

Dose delays and dose interruptions will be summarized by treatment arm.

Listing of treatment exposure will include duration of exposure, number of doses administered, cumulative dose administered, actual dose intensity, and relative dose intensity. A listing of dose administration will be provided and will include date of administration, actual dose, dose delay, infusion interruption, and dose modification.

5.7. Analyses of Efficacy Variables

Efficacy variables will be summarized according to the assigned treatment arms per IWRS and listed based on FAS in each study part, unless otherwise specified.

5.7.1. Primary Statistical Hypothesis

Part 1:

- Cemiplimab/chemo-f (Arm B) will prolong OS as compared with platinum-based doublet chemotherapy (Arm A)
- Cemiplimab/chemo-l/ipi (Arm C) will prolong OS as compared with platinum-based doublet chemotherapy (Arm A)

Part 2:

- Cemiplimab/chemo-f will prolong OS as compared with platinum-based doublet chemotherapy;

5.7.2. Primary and Key Secondary Efficacy Analyses

For the time-to-event endpoints (PFS and OS), the non-parametric Kaplan-Meier method will be used to estimate the survival curves. The treatment difference in survival will be assessed by the stratified log-rank test. A stratified Cox proportional hazard model with Efron's method of tie handling will be used to assess the magnitude of the treatment difference (i.e., the hazard ratio). The hazard ratio and its 95% confidence interval from the stratified Cox model with a single treatment covariate will be reported. The part specific stratification factors used for randomization per IWRS will be applied to both the stratified log-rank test and the stratified Cox model.

ORR will be analyzed using the Cochran-Mantel-Haenszel (CMH) test stratified by part specific stratification factors at randomization per IWRS. Objective response rate and the corresponding exact 95% CI will be calculated by Clopper-Pearson method for each treatment arm.

Overall survival rate at a landmark (12 months, 18 months, 24 months) will be summarized using Kaplan-Meier method for each treatment arm.

DoR will be summarized for confirmed responders (CR or PR) using Kaplan-Meier method for each treatment arm .

The change in EORTC QLQ-C30 and EORTC QLQ-LC13 scores from first assessment to the end of the study will be summarized descriptively at each post-baseline time point and compared using a mixed effects model, if appropriate.

Part 1:

For administrative purpose, the administrative analysis for PFS and ORR (per INV assessment using RECIST 1.1) will be conducted in patients who were randomized before the end of August 2019.

For the efficacy claim purpose, the analyses of primary and key secondary endpoints will be tested in the following order:

- OS comparison for cemiplimab/chemo-f vs. chemo-f
- OS comparison for cemiplimab/chemo-l/ipi vs. chemo-f
- PFS per IRC assessment comparison for cemiplimab/chemo-f vs. chemo-f
- PFS per IRC assessment comparison for cemiplimab/chemo-l/ipi vs. chemo-f
- ORR per IRC assessment comparison for cemiplimab/chemo-f vs. chemo-f
- ORR per IRC assessment comparison for cemiplimab/chemo-l/ipi vs. chemo-f

An interim analysis and final analysis for OS will be performed when approximately 125 (83% out of total OS events, 58% out of 215 randomized patients) and approximately 151 (70% out of 215 randomized patients) deaths are observed in the comparison of cemiplimab/chemo-f vs. chemo-f.

If the analyses of OS are statistically significant for both cemiplimab combination vs. chemotherapy comparisons (in the order of cemi/chemo-f vs. chemo-f and cemi/chemo-l/ipi vs. chemo-f), the secondary endpoint PFS per IRC assessment will be analyzed using the same statistical methods and strategies as used in the analyses of OS. If analyses of PFS are statistically significant, ORR per IRC assessment will be analyzed following the same analysis order as OS and PFS.

Part 2:

The primary and key secondary endpoints will be tested in the following order: OS, PFS, and ORR.

The analyses of primary endpoint of OS will be performed as follows: 1) two interim analyses for OS will be performed when approximately 146 (50% out of total OS events) and 204 (70% out of total OS events) deaths are observed; 2) final analysis for OS will be performed when approximately 291 deaths are observed.

If analysis of OS is statistically significant, the key secondary endpoint PFS will be analyzed using the same statistical method as used in analysis of OS.

5.7.3. Subgroup Analysis

Descriptive subgroup analyses will be performed on the primary endpoint and key secondary endpoints to summarize the treatment effects across subpopulations in each study part. The subpopulation are defined by the following baseline or screening factors:

- Age (< 65 versus ≥ 65)
- Race (White versus Non-White)
- Gender (Male versus Female)
- Ethnicity (Hispanic/Latino versus Other)
- Histology (squamous, non-squamous)
- PD-L1 expression levels (<1% versus 1% to <50% versus >=50%)
- ECOG status (0 versus 1)
- Geographic region of enrolling site

As subgroup analyses may not have enough power for hypothesis tests, those analyses will be exploratory in nature.

5.7.4. Adjustment for Multiple Comparison

The statistical analyses of the two parts will be conducted independently and summarized separately. Therefore, statistical control of overall type I error for the whole study is not planned. The familywise two-sided type error of 0.05 is controlled within each part of study.

Part 1:

For the two cemiplimab combinations versus standard of care chemotherapy comparisons, familywise type-I error rate of 0.05 is controlled by allocating 0.0001 to the administrative analyses of PFS and ORR per investigator assessment (0.00005 for each endpoint) and 0.0499 to the hierarchical testing of the OS, PFS and ORR per IRC in the order of cemi/chemo-f vs. chemo-f and cemi/chemo-l/ipi vs. chemo-f for each endpoint (Table 1).

Specifically, PFS and ORR per INV assessment will be controlled at nominal two-sided 0.00005 level respectively in patients who were randomized up to the end of August 2019. The multiplicity across OS, PFS and ORR per IRC assessment will be controlled at two-sided 0.0499 level by the hierarchical approach. That is, the analysis of PFS will be performed at two-sided 0.0499 level only if analyses of OS are statistically significant for both cemi comparisons, and the analyses of ORR will be performed at two-sided 0.0499 level only if the analyses of PFS are statistically significant.

The type I error for the interim analysis of OS at approximately 125 events and final analysis of OS at approximately 151 events is controlled at two-sided 0.0499 level using an α-spending function according to Lan-DeMets O'Brien-Fleming (OBF, O'Brien 1979, Gordon Lan 1983). The exact nominal p-values needed to declare statistical significance at the time of these analyses for OS will depend on the actual number of OS events at the time of the analysis.

All other statistical comparisons will be exploratory in nature and, therefore, not controlled for multiplicity and should be interpreted accordingly.

Table 1: Summary of Decision Guidance for Part 1 Analysis

Analysis	Endpoint (projected 2-sided alpha allocation)	Testing Steps
Administrative Look	ORR per investigator assessment in patients randomized by the end of August 2019 (0.00005)	1a. ORR: Arm B vs. Arm A<br>1b. ORR: Arm C vs. Arm A
	PFS per investigator assessment in patients randomized by the end of August 2019 (0.00005), will be tested in parallel of ORR per investigator assessment analyses	1a'. PFS: Arm B vs. Arm A<br>1b'. PFS: Arm C vs. Arm A
Efficacy Analysis	OS in ITT (0.0499)	2a. OS: Arm B vs. Arm A<br>2b. OS: Arm C vs. Arm A
	PFS per IRC in ITT (0.0499), only if the analyses of OS (Steps 2a and 2b) at either 2nd interim analysis or final OS analysis statistically significant	2c. PFS: Arm B vs. Arm A<br>2d. PFS: Arm C vs. Arm A

Analysis	Endpoint (projected 2-sided alpha allocation)	Testing Steps
	ORR in ITT, only if OS and PFS analyses are statistically significant (0.0499)	2e. ORR: Arm B vs. Arm A<br>2f. ORR: Arm C vs. Arm A

Part 2:

The family-wise type I error across the test of primary and key secondary endpoints and the repeated testing of OS in the two interim analyses and final analyses in Part 2 is controlled at two-sided 0.05 level.

The multiplicity between analyses of OS, PFS, and ORR will be controlled at two-sided 0.05 level by the hierarchical approach. That is, the analysis of PFS will be performed at two-sided 0.05 level only if analysis of OS is statistically significant, and the analysis of ORR will be performed at two-sided 0.05 level only if analysis of PFS is statistically significant.

The type I error for the two interim analyses of OS at 146 (50%) and 204 (70%) and final analysis of OS is controlled at two-sided 0.05 level according to OBF alpha spending function. The exact nominal p-values needed to declare statistical significance at the time of these analyses for OS will depend on the actual number of OS events at the time of the analyses.

All other statistical comparisons will be exploratory in nature and, therefore, not controlled for multiplicity and should be interpreted accordingly.

5.8. Analysis of Safety Data

The analysis of safety and tolerance will be performed on the SAF in each study part, as defined in Section 3.2. The safety analysis will be based on the drug exposure, reported AEs and other safety information (clinical laboratory evaluations, vital signs and 12-lead ECG). The analysis will comprise the basis upon which conclusions will be drawn regarding the Cemiplimab. The AE of special interest will be determined by the list provided by medical monitors.

The on-treatment period to detect any event or abnormality is between the first dose of investigational product and within 90 days after the last dose of investigational product or to 1 day before patients receive another anti-cancer systemic therapy (including optional cemiplimab treatment and retreatment for Part 1 patients), whichever is earlier. The 90-day interval is chosen based on the half-life of the investigational product (approximately 5 times the half-life). Data collected outside this interval will be excluded from the estimation of descriptive statistics and identification of abnormalities for laboratory evaluations and vital signs.

Day 1 is the first day of investigational product, Day –1 is the day before, and there is no Day 0.

The summary of safety results will be presented for each treatment arm in tables and listings.

Dose limiting toxicities observed during the DLT evaluation period will be summarized by treatment arm and will be assessed using the DLT analysis set.

5.8.1. Adverse Events

The verbatim text, the preferred term (PT), and the primary system organ class (SOC) will be listed in subject listings. Summaries that include frequencies and proportions of patients reporting AEs will include the PTs and the SOCs.

Period of observation: The observation period will be divided into three segments: pre-treatment, on-treatment and follow up for Part 1 and Par 2 respectively.

- The pre-treatment period is defined as the time between when the patients give informed consent and the start of investigational product.
- The on-treatment period to detect any event or abnormality is defined as the time from first dose of investigational product up to 90 days after the last dose of investigational product or to 1 day before patients receive another anti-cancer systemic therapy (including optional cemiplimab treatment), whichever is earlier.

For Part 1 patients in the treatment arm B and C who received Cemiplimab retreatment:

- Start Cemiplimab less than or equal to 45 days of their last regular Cemiplimab treatment: the on-treatment period is defined as the time from the first dose of Cemiplimab up to 90 days after the last dose of Cemiplimab re-treatment or date cutoff date, whichever is earlier. That is, the re-treatment is considered as a part of the regular treatment in this case.
- Start Cemiplimab more than 45 days of their last regular Cemiplimab treatment: the on-treatment period is defined as the time from the first dose of Cemiplimab up to 90 days after the last dose of cemiplimab regular treatment, date cutoff date, or 1 day before patients receive their first dose of new anti-cancer systemic therapy (including retreatment of cemiplimab), whichever is earlier.
- The post-treatment period is defined as the time starting one day after the end of on-treatment period. The retreatment period is a subset of post treatment period (for Part 1 patients in arm B and arm C who received cemiplimab retreatment) and is defined as the time from the first dose of cemiplimab retreatment up to 90 days after the last dose of cemiplimab retreatment.
- **Pre-treatment AEs** are defined as AEs that developed or worsened during the pre-treatment period.
- **Treatment-emergent AEs** (TEAEs) are defined as AEs that developed or worsened during the on-treatment period and any treatment-related AEs that occur during the post-treatment period but prior to start of optional cemiplimab treatment (patients treated on Arm A in Part I) or retreatment (patients treated on Arm B or C in Part 1).
- **Post-treatment AEs** are defined as AEs that developed or worsened during post treatment period and are not considered drug related by the investigator.

The focus of adverse event reporting in the clinical study report will be on TEAEs. For details on handling missing data and partial dates, see Section 6.

Summaries of TEAEs will include: TEAEs, Treatment related TEAEs, Serious TEAEs, Treatment-related Serious TEAEs and treatment-emergent AESI. For TEAEs, the following will be summarized:

- The number and proportions of patients reporting at least 1 TEAE, presented by SOC and PT
- TEAEs by severity (CTCAE, latest available version), presented by SOC and PT
- TEAEs related to treatment, presented by SOC and PT
- TEAEs leading to permanent treatment discontinuation, presented by SOC and PT
- TEAEs leading to death, presented by SOC and PT

For each TEAE summary presented by SOC and PT, the summary table will be sorted by decreasing frequency of SOC and PT. For TEAE summary presented by PT, the summary table will be sorted by decreasing frequency of PT.

For AE listings, the following variables will be displayed:

- Verbatim Term, SOC, PT
- AE start date and end date (and corresponding study day)
- Relationship to study drug: unrelated or related
- Seriousness (Serious AE or not)
- National Cancer Institute-Common Terminology Criteria for Adverse Events (NCI-CTCAE) grade
- Action taken
- Treatment for AE: none, medication, procedure/surgery
- Outcome

5.8.2. Clinical Laboratory Measurements

Listings of laboratory values, normal ranges, grade, by dose cohort, date, and visit/cycle will be provided. For numeric lab variables and change from baseline to each visit/cycle will be summarized. Listings of abnormal lab values and clinically significant (Yes/No) by patient and visit/cycle will also be constructed.

Summary tables for worst laboratory values during on-treatment period with NCI CTCAE all grade and grade ≥ 3 will be generated. Shift tables from baseline to worst post-treatment NCI CTCAE grade during on-treatment period will be generated.

5.8.3. Analysis of Vital Signs

Vital signs (pulse, sitting blood pressures, and temperature) will be listed and summarized by Baseline and change from Baseline to each scheduled assessment time with descriptive statistics.

5.8.4. Analysis of 12-Lead ECG

ECG parameters (P-R interval ,QT interval, QTc interval, QRS interval, Ventricular rate and Heart rate) will be listed and summarized by Baseline and change from Baseline to each scheduled and collected assessment time.

ECG status (i.e. normal, abnormal) will be reported. Shift tables will be provided to present the post-baseline status according to the baseline status (normal or missing / abnormal) by treatment arm.

5.8.5. Physical Exams

Physical examination findings at baseline as well as post-treatment abnormal findings by body system and status (normal, abnormal and not done) will be provided with Listing.

5.9. Analysis of Pharmacokinetic and Antibody Data

5.9.1. Analysis of Pharmacokinetic Data

Concentrations of cemiplimab in serum will be measured at multiple time points throughout the study treatment and follow-up periods, and the PK variables will be determined.

Summaries of study drug concentrations will be presented by nominal time point (i.e., the time points specified in the protocol) and group. Pharmacokinetic variables, including C_{eoi}, C_{trough}, and t_{eoi}, will be presented as individual values with descriptive statistics.

5.9.2. Analysis of Anti-Drug Antibody Data

The ADA variables described in Section 4.4 will be summarized using descriptive statistics in the ADA analysis set of the REGN2810 treatment arms (Treatment Arms B and C). Frequency tables of the proportion of patients with treatment-emergent, treatment-boosted, persistent ADA response, and NAb status in the NAb assay will be presented as absolute occurrence (n) and percentage of patients (%), presented by treatment arms.

Plots of REGN2810 concentrations will be examined, and the influence of ADAs on individual concentration-time profiles may be evaluated. Assessment of impact of ADA on safety and efficacy may be provided.

5.9.3. Analysis of Exploratory Biomarker Data

Biomarker analyses in this study will be exploratory in nature and results will be summarized in a separate report. Detailed description of statistical methods that will be used for biomarker data analyses will be provided in a separate Biomarker Analytical Plan.

6. DATA CONVENTIONS

The following analysis conventions will be used in the statistical analysis.

6.1. Definition of Baseline for Efficacy/Safety Variables

Unless otherwise specified, the Baseline assessment for all measurements will be the latest available valid measurement taken prior to the administration of investigational product.

6.2. Data Handling Convention for Efficacy Variables

Patients who are deemed NE according to RECIST version 1.1. or unevaluable by the composite response criteria will be considered as not reaching PR/CR in calculating ORR, i.e. they are not considered as responders in the numerator of ORR, but they are counted in the denominator of ORR.

6.3. Data Handling Convention for Missing Data

For time to event endpoints, missing date imputation is absolutely restricted. Rules for handling missing data for primary and secondary efficacy variables are described in Sections 4.6.1 and Section 4.6.2.

No missing data imputation is planned in this study unless specified otherwise.

For categorical variables, patients with missing data are not included in calculations of percentages unless otherwise specified. When relevant, the number of patients with missing data is presented.

Medication missing/partial dates

To determine whether a medication is prior, concomitant or post-treatment medication, the missing medication start date is estimated as early as possible up to date of the first study treatment, and the missing medication end date is estimated as late as possible. If the medication start date is missing, the onset day will not be imputed in medication listings.

Adverse event

If the intensity of a TEAE is missing, it will be classified as "severe" in the frequency tables by intensity of TEAEs. If the assessment of relationship of a TEAE to the investigational product is missing, it will be classified as related to the investigational product.

Date of first / last study treatment

Date of first infusion is the first non-missing start date of dosing filled in the CRF "Investigational Product" module.

If a patient's date of the last dose is totally missing or unknown, his/her last visit date will be substituted.

6.4. Visit Windows

Assessments taken outside of protocol allowable windows will be displayed according to the case report form (CRF) assessment recorded by the investigator.

6.5. Unscheduled Assessments

Unscheduled visit measurements may be used to provide a measurement for a baseline or endpoint value if appropriate according to their definition. The measurements may also be used to determine abnormal laboratory or ECG values.

Unscheduled visit measurements may be used to provide a measurement for a baseline or endpoint value if appropriate according to their definition. The measurements may also be used to determine abnormal laboratory or ECG values.

The determination of baselines and values at the end of treatment for both efficacy and safety variables will be based on scheduled available assessments and unscheduled available assessments.

Extra assessments (laboratory data or vital signs associated with non-protocol clinical visits or obtained during investigating or managing adverse events) will be included in listings, but not summaries except for the endpoint determination. If more than one laboratory value is available for a given visit, the first observation will be used in summaries and all observations will be presented in listings.

7. INTERIM ANALYSIS

The tables below give an example of alpha spending. Actual alpha spending for each interim analysis will be calculated using O'Brien-Fleming alpha spending function based actual number of events included in the interim analysis .

Part 1:

For administrative purposes, analyses will be performed for PFS and ORR per investigator assessment in Part 1 patients who were randomized by August 2019. This administrative analysis will be performed after all Part 1 patients who were randomized by August 2019 have opportunity for three tumor assessments.

An interim analysis for OS is planned in addition to the final analysis. The OBF alpha spending for analyses of OS is specified in Table 2 .

Table 2: Alpha Spending for Analysis of OS in Part 1

		**Value**
Interim Analysis for OS<br>Death = ~ 125	Z	2.205
	Alpha (2-sided[a])	0.02745
Final Analysis for OS<br>Deaths= ~ 151	Z	2.034
	Alpha (2-sided)	0.04195

[a] as a two-sided test is used at interim, the superiority of cemiplimab treatment will be claimed if the statistical boundary is crossed.

Part 2:

Two interim analyses of OS are planned in addition to the final analysis. The OBF alpha spending for analysis of OS is specified in Table 3.

Table 3: Alpha Spending for Analysis of OS in Part 2

OS		Value
Interim Analysis<br>Death = ~146	Z	2.958
	Alpha (2-sided[a])	0.00310
Interim Analysis<br>Death = ~ 204	Z	2.465
	Alpha (2-sided[a])	0.01370
Final Analysis<br>Death = ~ 291	Z	2.002
	Alpha (2-sided)	0.04528

[a] as a two-sided test is used at interim, the superiority of cemiplimab treatment will be claimed if the statistical boundary is crossed.

8. SOFTWARE

All statistical analyses will be done using SAS Version 9.4 or above.

9. REFERENCES

ICH. (1998, February 5). ICH Harmonized tripartite guideline: Statistical principles for clinical trials (E9). International Conference on Harmonization of Technical Requirements for Registration of Pharmaceuticals for Human Use.

Borghaei et al. Updated results from KEYNOTE-021 cohort G: A randomized, phase 2 study of pemetrexed and carboplatin (PC) with or without pembrolizumab (pembro) as first-line therapy for advanced nonsquamous NSCLC. ESMO 2017 Congress, September 2017.

Brahmer, JR et al. 2017. Updated Analysis of KEYNOTE-024: Pembrolizumab Versus Platinum-based Chemotherapy for Advanced NSCLC With PD-L1≥50%. International Association for the Study of Lung Cancer 18th World COnference on Lung Cancer. Yokohama, Japan.

De Lima Lopes G, Wu Y, Sadowski S, Zhang J, Rangwala R, et al. P2.43: Pembrolizumab vs platinum-based chemotherapy for PD-L1+ NSCLC: phase 3, randomized, open-label KEYNOTE-042: track: immunotherapy. J Thorac Oncol. 2018;11(10S): S244-S245.

El-Shenshawy HM, Taema S, El-Zahaf E, El-Beshbeshi W, Eldeen DS, Fathy A. Advanced non-small cell lung cancer in elderly patients: the standard every 3-weeks versus weekly paclitaxel with carboplatin. Chest. 2012;61:485-93.

Gandhi L, Rodríguez-Abreu D, et al. Pembrolizumab plus Chemotherapy in Metastatic Non-Small-Cell Lung Cancer. N Engl J Med. 2018 May 31;378(22):2078-2092. doi: 10.1056/NEJMoa1801005. Epub 2018 Apr 16.

Gordon Lan KK, Demets DL, Discrete sequential boundaries for clinical trials. Biometrika 1983; 70(3):659-63

O'Brien PC, Fleming TR. A multiple testing procedure for clinical trials. Biometrics 1979; 35(3):549-56

Paz-Ares LG, et al. Phase 3 study of carboplatin-paclitaxel/nab-paclitaxel with or without pembrolizumab for patients with metastatic squamous non-small cell lung cancer. 2018 ASCO Annual Meeting. Abstract 105.

Rosell R, Gatzemeier U, Betticher DC, Keppler U, Macha HN, Pirker R, et al. Phase III randomised trial comparing paclitaxel/carboplatin with paclitaxel/cisplatin in patients with advanced non-small-cell lung cancer: a cooperative multinational trial. Ann Oncol. 2002;13:1539-49.

Scagliotti GV, De Marinis F, Rinaldi M, Crinò L, Gridelli C, Ricci S, et al. Phase III randomized trial comparing three platinum-based doublets in advanced non-small-cell lung cancer. J Clin Oncol. 2002;20:4285-91.

Schiller JH, Harrington D, Belani CP, Langer C, Sandler A, Krook J, et al. Comparison of four chemotherapy regimens for advanced non-small-cell lung cancer. N Engl J Med. 2002;346:92-8.

Shimizu T, Yokoi T, Tamaki T, Kibata K, Inagaki N, Nomura S. Comparative analysis of carboplatin and paclitaxel combination chemotherapy schedules in previously untreated patients with advanced non-small cell lung cancer. Oncol Lett. 2013;5:761-7.

Socinski M, Creelan B, Horn L, Reck M, Paz-Are L, Steins M, et al. CheckMate 026: A phase 3 trial of nivolumab vs investigator's choice (IC) of platinum-based doublet chemotherapy (PT-DC) as first-line therapy for stage IV/recurrent programmed death ligand 1 (PD-L1)-positive NSCLC. Annals of Oncology. 2016;27 Suppl 6:LBA7_PR.

Signature Page for VV-RIM-00106349 v2.0

ESig Approval	[redacted] 000

ESig Approval	[redacted] -2020 16:13:51 GMT+0000

Signature Page for VV-RIM-00106349 v2.0 Approved
