# Supplementary material for: Cemiplimab plus chemotherapy versus chemotherapy alone in non-small cell lung cancer: a randomized, controlled, double-blind phase 3 trial
Source: Nat Med. 2022 Aug 25;28(11):2374–80. doi: 10.1038/s41591-022-01977-y (PMC9671806; doi:10.1038/s41591-022-01977-y)
Supplement: Supplementary file 2 — Reporting Summary [file 41591_2022_1977_MOESM2_ESM.pdf]

nature research

Corresponding author(s): Miranda Gogishvili, MD

Last updated by author(s): July 22, 2022

Reporting Summary

Nature Research wishes to improve the reproducibility of the work that we publish. This form provides structure for consistency and transparency in reporting. For further information on Nature Research policies, see our Editorial Policies and the Editorial Policy Checklist.

Statistics

For all statistical analyses, confirm that the following items are present in the figure legend, table legend, main text, or Methods section.

n/a	Confirmed	
☐	☒	The exact sample size (*n*) for each experimental group/condition, given as a discrete number and unit of measurement
☐	☒	A statement on whether measurements were taken from distinct samples or whether the same sample was measured repeatedly
☐	☒	The statistical test(s) used AND whether they are one- or two-sided<br>*Only common tests should be described solely by name; describe more complex techniques in the Methods section.*
☐	☒	A description of all covariates tested
☐	☒	A description of any assumptions or corrections, such as tests of normality and adjustment for multiple comparisons
☐	☒	A full description of the statistical parameters including central tendency (e.g. means) or other basic estimates (e.g. regression coefficient) AND variation (e.g. standard deviation) or associated estimates of uncertainty (e.g. confidence intervals)
☐	☒	For null hypothesis testing, the test statistic (e.g. *F*, *t*, *r*) with confidence intervals, effect sizes, degrees of freedom and *P* value noted<br>*Give P values as exact values whenever suitable.*
☒	☐	For Bayesian analysis, information on the choice of priors and Markov chain Monte Carlo settings
☐	☒	For hierarchical and complex designs, identification of the appropriate level for tests and full reporting of outcomes
☐	☒	Estimates of effect sizes (e.g. Cohen's *d*, Pearson's *r*), indicating how they were calculated

Our web collection on statistics for biologists contains articles on many of the points above.

Software and code

Policy information about availability of computer code

Data collection	Clinical data were captured in the clincial database using the Medidata Rave Electronic Capture Data system (Version 2021.2.0).
Data analysis	All statistical analyses were performed using SAS Version 9.4 or above.

For manuscripts utilizing custom algorithms or software that are central to the research but not yet described in published literature, software must be made available to editors and reviewers. We strongly encourage code deposition in a community repository (e.g. GitHub). See the Nature Research guidelines for submitting code & software for further information.

Data

Policy information about availability of data

All manuscripts must include a data availability statement. This statement should provide the following information, where applicable:

- Accession codes, unique identifiers, or web links for publicly available datasets
- A list of figures that have associated raw data
- A description of any restrictions on data availability

Qualified researchers may request access to study documents (including the clinical study report, study protocol with any amendments, blank case report form, statistical analysis plan) that support the methods and findings reported in this manuscript. Individual anonymized participant data will be considered for sharing once the product and indication has been approved by major health authorities (e.g., FDA, EMA, PMDA, etc.), if there is legal authority to share the data and there is not a reasonable likelihood of participant re-identification. Submit requests to https://vivli.org/.

Field-specific reporting

Please select the one below that is the best fit for your research. If you are not sure, read the appropriate sections before making your selection.

☒ Life sciences ☐ Behavioural & social sciences ☐ Ecological, evolutionary & environmental sciences

For a reference copy of the document with all sections, see nature.com/documents/nr-reporting-summary-flat.pdf

Life sciences study design

All studies must disclose on these points even when the disclosure is negative.

Sample size	We estimated that a sample size of 450 randomized patients would yield approximately 93% power to detect a statistically significant difference in overall survival at a two-sided type 1 error level of 0.05 between the two treatment arms.
Data exclusions	There were no data exclusions
Replication	Not applicable for clinical data, as we are reporting pre-specified analysis of a phase III clinical trial.
Randomization	Randomization (2:1) was performed according to a central randomization scheme provided by an interactive web response system manual. Randomization was stratified by histology (non-squamous versus squamous) and levels of programmed cell death-ligand 1 (PD-L1) expression.
Blinding	Open label cemiplimab was blinded by an unblinded pharmacist at each site; patients, principal investigators, and other study site personnel remained blinded to all randomization.

Reporting for specific materials, systems and methods

We require information from authors about some types of materials, experimental systems and methods used in many studies. Here, indicate whether each material, system or method listed is relevant to your study. If you are not sure if a list item applies to your research, read the appropriate section before selecting a response.

Materials & experimental systems

n/a	Involved in the study	
☐	☒	Antibodies
☒	☐	Eukaryotic cell lines
☒	☐	Palaeontology and archaeology
☒	☐	Animals and other organisms
☐	☒	Human research participants
☐	☒	Clinical data
☒	☐	Dual use research of concern

Methods

n/a	Involved in the study	
☒	☐	ChIP-seq
☒	☐	Flow cytometry
☒	☐	MRI-based neuroimaging

Antibodies

Antibodies used	Cemiplimab; Regeneron Pharmaceuticals, Inc.
Validation	Cemiplimab-rwlc is a human programmed death receptor-1 (PD-1) blocking antibody. Cemiplimab-rwlc is a recombinant human IgG4 monoclonal antibody that binds to PD-1 and blocks its interaction with PD-L1 and PD-L2. Cemiplimab-rwlc is produced by recombinant DNA technology in Chinese hamster ovary (CHO) cell suspension culture. Cemiplimab-rwlc has an approximate molecular weight of 146 kDa.<br><br>For further information, please see the prescribing information, which may be found here: https://www.accessdata.fda.gov/drugsatfda_docs/label/2021/761097s007lbl.pdf

Human research participants

Policy information about studies involving human research participants

Population characteristics	Adult patients (both male and female) with stage III or IV non-small cell lung cancer and no actionable tumor mutations (eg, epidermal growth factor receptor, anaplastic lymphoma kinase, or ROS proto-oncogene 1 mutations). Sex and/or gender was not considered in the study design. Sex and/or gender of participants was determined based on self reporting to the treating physician and the study Sponsor was not involved in the process nor gave specific indications to the Investigators regarding the methodology used.

Recruitment

Patients were recruited at 74 study sites across 10 countries (primarily in Central and Eastern Europe). There was no selection bias in recruitment of patients in the trial.

Ethics oversight

Ethics Committee of Shanghai Pulmonary Hospital, No.507 Zhengmin Road, Yangpu District, Shanghai, 200433, China; Life Ethics Committee of Beijing Friendship Hospital, Capital Medical University, No.95 YongAn Road, Xicheng District, Beijing, 100050, China; Ethics Committee of Jinan Central Hospital, No.105 Jiefang Road, Jinan, Shandong, 250013, China; Ethics Committee of Affiliated Hospital of Hebei University, No. 648 Dongfeng east road, Lianchi Qu, Baoding Shi, Hebei Sheng, State Zip Code 071105, China; The Ethics Committee of Cancer Hospital of Xinjiang Medical University, No.789, Suzhou East Street, New urban area, Urumqi, Xinjiang, 830000, China; Ethics Committee of Clinical Trials of the First Affiliated Hospital, College of Medicine, Zhejiang University, No.79 Qingchun Road, Hangzhou, Zhejiang, 310003, China; Ethics Committee of the Second Affiliated Hospital of Zhejiang University School of Medicine, No.88 Jiefang Road, Shangcheng District, Hangzhou, Zhejiang, 310009, China; Institutional Review Board of Huadong Hospital, No.168 Yan'an Road West, Jing'an District, Shanghai, 200040, China; Clinical Trial Ethics Committee of the Henan Provincial Peoples Hospital, No.7, Weiwu Road, Zhengzhou, Henan, 450003, China; Ethics Committee of The Second Affiliated Hospital of Nanjing Medical University, No.121 Jiangjiayuan Road, Gulou district, Nanjing, Jiangsu, 210011, China; The First Affiliated Hospital of Guangzhou Medical University, No. 151, Yanjiang West Road, Yuexiu District, Guangzhou City, Guangdong Province, China, 510030; Ethics Committee of Xiangyang Central Hospital, No.136 Jinzhou Street, Xiangcheng, Xiangyang City, Hubei, 441021, China; Ethics Committee of the First Affiliated Hospital of Guangdong Pharmaceutical University, No. 19 Nonglinxia road, Yuexiu District, Guangzhou, Guangdong, 510000, China; Ethics Committee of Henan Cancer Hospital, No.127 Dongming Road, Zhengzhou, Henan, 450008, China; Good Clinical Practice Office of Hanghou First Peoples Hospital, Hangzhou Cancer Hospital No.34, Yanguan Xiang, Shangcheng district, Hangzhou City, Zhejiang Province, China, 310002; Anhui Provincial Cancer Hospital Clinical Trial Ethics Committee, No.107, Huanhu East Road, Shushan District, Hefei, Anhui, 230031, China; Ethics Committee of Fuzhou Pulmonary Hospital of Fujian, No.2 Hubian, Cangshan District, Fuzhou, Fujian, 350008, China; Medical Ethics Committee of Liaoning Cancer Hospital and Institute, No. 44, XiaoHeYan Road, Dadong District, Shenyang, Liaoning, 110042, China; Ethics Committee of Hunan Cancer Hospital, No.283, Tongzipo Road, Yuelu District, Changsha, Hunan, 410013, China; Ethics Committee of Linyi Cancer Hospital, Linyi Cancer Hospital, Intersection between Zhongsheng street and Zhicheng Road, Hedong District, Linyi City, Shangdong Province, 276000, China; Ethics Review Committee of Zhejiang Hospital, No.12 Lingyin Road, Xihu District, Hangzhou, Zhejiang Province, 310013, China; Local Ethics Committee of High Technology Medical Center University Clinic, 9 Tsinandali Street, 0144 Tbilisi, Georgia; Local Ethics Committee of Adjara Autonomic Republic, 118 Pushkin Street, 6000 Batumi, Georgia; Local Ethics Committee of JSC "Neo Medi", 12 Kristine Sharashidze Street, 0159 Tbilisi, Georgia; Local Ethics Committee of Institute of Clinical Oncology, 5 Lubliana Street, 0156 Tbilisi, Georgia; Local Ethics Committee of Research Institute of Clinical Medicine, 13 Tevdore Mgvdeli Street, 0112 Tbilisi, Georgia; LEC of "Multi-Profile Clinic Consilium Medulla", 6g Politkovskaia Steet, 0186 Tbilisi, Georgia; Hellenic Republic Ministry of Health National Ethic Committee (NEC) 284 Mesogion Avenue, 155 62 Cholargos, Greece; Medical Research & Ethics Committee, C/o Kompleks Institut Kesihatan Negara Blok A, No 1, Jalan Setia Murni U13/52, Seksyen U13, Bandar Setia Alam, 40170 Shah Alam, Selangor, Malaysia; Clinical Investigation Centre, 5th Floor, East Tower, University Malaya Medical Centre, 59100, Lembah Pantai, Kuala Lumpur, Malaysia; Research Ethics Committee, The National University of Malaysia, 1st Floor, Clinical Block, Hospital Canselor Tuanku Muhriz, Universiti Kebangsaan Malaysia Medical Centre, Jalan Yaacob Latif, Bandar Tun Razak, 56000 Cheras, Kuala Lumpur, Malaysia; Komisja Bioetyczna przy Okręgowej Izbie Lekarskiej w Lublinie Ul, Chmielna 4, 20-079 Lublin, Poland; Romania Academy of Medical Sciences, National Bioethics Committee for Medicines and Medical Devices, Sos. Stefan cel Mare nr. 19-21, Sector 2, Bucharest, Romania; Ethics Committee of State Budgetary Healthcare Institution of Sverdlovsk Region "Sverdlovsk Regional Oncology Dispensary", 29, Soboleva Street, Yekaterinburg, 620036, Russian Federation; Ethics Council at the Ministry of Healthcare of the Russian Federation 127994, Moscow, Rakhmanovskiy pereulok, 3; Ethics Committee at the Regional Budget Healthcare Institutio "Kursk Regional Scientific Clinical Centra n.a. G. E. Ostroverkhov" Ul, Eliseeva, 1 Kislino, 305524 Kursk Region, Kursk District, Russian Federation; Ethics Committee at City Clinical Oncologic Dispensary of St. Petersburg, 3/5, 2-ya Berezovaya alleya, Saint Petersburg, Russian Federation / 56, Prospekt Veteranov, Saint Petersburg, 197022 Russian Federation; Ethics Committee of Arkhangelsk Clinical Oncology Dispensary Bld.1, 145, Obvodniy Kanal Prospekt, Arkhangelsk, 163045 Russian Federation; Federal State Budgetary Institution "National Medical Research Centre of Oncology named after N.N. Petrov" of the Ministry of Healthcare of the Russian Federation 68, Leningradskaya Street., pos. Pesochny, Saint Petersburg, 197758, Russian Federation; Federal State Budgetary Educational Institution of Higher Education "National Research Ogarev Mordovia State University", Medical Institute, 26a, Ulyanov Street, 430032 Saransk, Republic of Mordovia, Russian Federation; Independent Ethics Committee of "Arte Med Assistance" LLC, Office 44, Lit. Ts, 27 prospekt Engelsa, 194156 St. Petersburg, Russian Federation; Federal State Budgetary Educational Institution of Higher Education "Siberian State Medical University" of Ministry of Healthcare of Russia, 15, Kotovskogo Street, 634034 Tomsk, Russian Federation; Ethics Committee at the State Budgetary Healthcare Institution of Kaluga Region "Kaluga Regional Clinical Oncology Dispensary", 2, Vishnevskogo Street., Kaluga, Kaluga Region, 248007, Russian Federation; Committee for Biomedical Ethics at the Research Institute of Oncology of Tomsk National Research Medical Centre ul. Savinykh, 12/1, 634028 Tomsk Region, Tomsk, Russian Federation; Ethics Committee of State Budgetary Healthcare Institution of Kemerovo Region "Regional Clinical Oncology Dispensary", 35, Volgogradskaya Street., Kemerovo, Kemerovo Region, 650036, Russian Federation; Ethics Committee at the Private Institution Educational Organisation of Higher Education Reaviz Medical University ul. Chkalova, d. 100, 443030 Samara, Russian Federation; Ethics Committee of Federal State Budgetary Institution "N. N. Blokhin National Medical Research Center of Oncology" of the Ministry of Health of the Russian Federation, 24, Kashirskoe shosse, Moscow, 115478, Russian Federation; Local Ethics Committee of "EVIMED" LLC Offices 10, 22, 9-v, Blyukhera Street., Chelyabinsk, 454048, Russian Federation; Ethics Committee of "Komanda" LLC, Room 37-N, Lit. A, Bld. 2, 19 Frunze Street., Saint-Petersburg, 196135, Russian Federation; Federal State Budgetary Educational Institution of Higher Education Bashkir State Medical University of the Ministry of Healthcare of the Russian Federation ul. Lenina 3, 450008 Ufa, Republic of Bashkortostan; Independent Interdisciplinary Ethics Committee on Ethical Review for Clinical Studies, 51, Leningradskiy Avenue, 125468 Moscow, Russian Federation; Ethics Committee at the State Budgetary Healthcare Institution "Leningrad Regional Clinical Oncology Dispensary", Liteyny prospect 37-39, 191014 St. Petersburg, Russian Federation; State Autonomous Healthcare Institution Republican Clinical Oncology Center of the Ministry of Healthcare of the Republic of Tatarstan, Sibirskiy trakt, 29, 420029 Kazan, Russian Federation; State Budgetary Healthcare Institution of Stavropol Region "Pyatigorsk Interdistrict Oncology Dispensary", 31, Kalinina Prospect, Pyatigorsk, Stavropol Territory, 357502, Russian Federation; Committee on Ethics Expertise of Clinical Studies of Regional Budgetary Healthcare Institution "Belgorod Oncology Dispensary", 1, Kuybysheva Street, 308010, Belgorod, Russian Federation; Ethics Committee of Budgetary Healthcare Institution of Omsk Region "Clinical Oncology Dispensary", Bld. 1, 9, Zavertyayeva Street, Omsk, 644013, Russian

Federation; Independent Interdisciplinary Ethics Committee on Ethical Review for Clinical Studies, 51, Leningradskiy Avenue, 125468 Moscow, Russian Federation; Ethics Committee in Human Research, Udonthani Cancer Hospital, 36 Moo 1, Udon-Khon Kean Road, Muang, Udonthani 41330, Thailand; Central Research Ethics Committee, 5th Floor, Building 2, The National Research Council of Thailand, Paholyothin Road, Lad Yao Sub-district, Chatuchak District, Bangkok 10900, Thailand; Naresuan University Institutional Review Board, 99 Moo 9 Tha Pho, Muang, Phitsanulok 65000, Thailand; Lopburi Cancer Hospital Ethics Committee for Human Research, 11/1 Paholyothin Road, Thalae Chup Son, Mueang, Lopburi 15000, Thailand; Institution Review Board, Royal Thai Army Medical Department, 5th floor Phramongkutklao wejvitya Building, Phramongkutklao College of Medicine, 317/5 Rajavithi Road, Rajathevee, Bangkok 10400, Thailand; The Ethics Committee of Lampang Cancer Hospital, 199 Moo12, Pichai, Muang, Lampang 52000, Thailand; Central Research Ethics Committee, 3rd Floor, Building 3, The National Research Council of Thailand, 196 Moo 5, Phaholyothin Road, Ladyao, Chatuchak, Bangkok 10900, Thailand; Human Research Ethics Committee Faculty of Medicine, Prince of Songkla University, 15 Karnjanavanit Road, Hat Yai, Songkhla 90110, Thailand; The Ethical Committee for Research in Human Subject, Chiangrai Prachanukroh Hospital, 1039 Sathanpayaban Road, Muang, Chiang Rai 57000, Thailand; Khon Kaen Hospital Institute Review Board in Human Research, 54, 56 Sri Chan Road, Naimaung sub-district, Muang district, Khon Kaen 40000, Thailand; Ankara University Faculty of Medicine Clinical Trials Ethics Committee, Ankara Üniversitesi Tıp Fakültesi Morfoloji Binası, 6100 Sıhhiye/Ankara, Turkey; Local ethics committee of MI “City Dnipropetrovsk Multi-field Clinical Hospital #4 of DRC”, 31, Blizhniaya Street, Dnipro, 49102, Ukraine; Ethics committee of Medical and Diagnostic Center of Private Enterprise of Private Production Company “Acinus”, 65, Velyka Perspektyvna Street, Kropyvnytskyi, 25006, Ukraine; Ethics committee of Municipal non-profit entrprise "Regional oncology center", 4, Lisoparkivska Street, Kharkiv, 61070, Ukraine; Ethics committee of Podilsky Regional Center of Oncology, 84, Khmelnytske Highway, Vinnytsia, 21029, Ukraine; Ethics committee of National Cancer Institute, 33/43, Lomonosova Street, Kyiv, 03022, Ukraine; Ethics Committee at Municipal Non-profit Enterprise “Transcarpathian Antitumor Center” of the Transcarpathian Regional Council, 2, Brodlakovych Street, Uzhorod, 88014, Ukraine.

Note that full information on the approval of the study protocol must also be provided in the manuscript.

Clinical data

Policy information about clinical studies

All manuscripts should comply with the ICMJE guidelines for publication of clinical research and a completed CONSORT checklist must be included with all submissions.

Clinical trial registration: NCT03409614

Study protocol: The trial protocol will be made available as part of the supplementary material.

Data collection: Data was collected between June 2019 and September 2020 at 74 sites in 10 countries, primarily in Central and Eastern Europe.

Outcomes: The primary endpoint was overall survival, defined as the time from randomization to the date of death.

Secondary endpoints included:
- Progression-free survival, defined as the time from randomization to the date of the first documented tumor progression as determined by the blinded independent review committee or death, whichever is earlier.
- Objective response rate, defined as the proportion of patients with a best overall response of confirmed complete response or partial response, per blinded independent review committee.
- Patient-reported outcomes (PROs) were measured as predefined secondary endpoints using multiple instruments, including the European Organization for Research and Treatment of Cancer Quality of Life Core 30 (EORTC QLQ-C30) questionnaire.
- To evaluate the safety and tolerability of cemiplimab plus chemotherapy versus placebo plus chemotherapy.

All efficacy endpoints were assessed in the intention-to-treat population. Safety was assessed in all randomized patients who received at least one dose of the assigned treatment.

Assessments:
- Baseline assessments included collection of tumor tissue samples for evaluation of PD-L1 expression; formalin-fixed, paraffin-embedded tumor samples were assessed at a central laboratory using the SP263 assay. Tumor tissue samples were also tested centrally for EGFR, ALK, and ROS1 mutations.
- Radiographic tumor assessments were obtained every 9 weeks for the first year and every 12 weeks thereafter until disease progression, withdrawal, death, or initiation of another anticancer treatment; responses were assessed by RECIST 1.1 criteria.
- PROs were measured using the EORTC QLQ-C30 questionnaire at baseline, the beginning of each treatment cycle for the first six doses, then at the start of every three cycles, and at the end of treatment.
- Adverse events and laboratory abnormalities were graded according to the National Cancer Institute Common Terminology Criteria for Adverse Events, version 4.03.
